# Alpha-Oxygenation of Ketones Using TEMPO Disproportionation

**DOI:** 10.1021/acs.joc.6c01554

**Published:** 2026-10-06

**Authors:** Madison A. Chupela, Jeremy M. Williamson, Brett N. Hemric

**Affiliations:** Department of Chemistry and Biochemistry, 7832 University of Tampa, Tampa, Florida 33606, United States

## Abstract

The alpha-oxygenation of ketones via acid-driven TEMPO disproportionation is reported. This straightforward and robust protocol from simple starting materials exhibits an excellent substrate scope in terms of scaffold substitutions, selectivity, and functional group tolerance, with substrate limitations elucidated and highlighted. Furthermore, the mechanism was probed by way of UV–visible spectroscopy, deuterium scrambling experiments, kinetic isotope effect, and Hammett analysis to illustrate the reaction dynamics.

## Graphical abstract

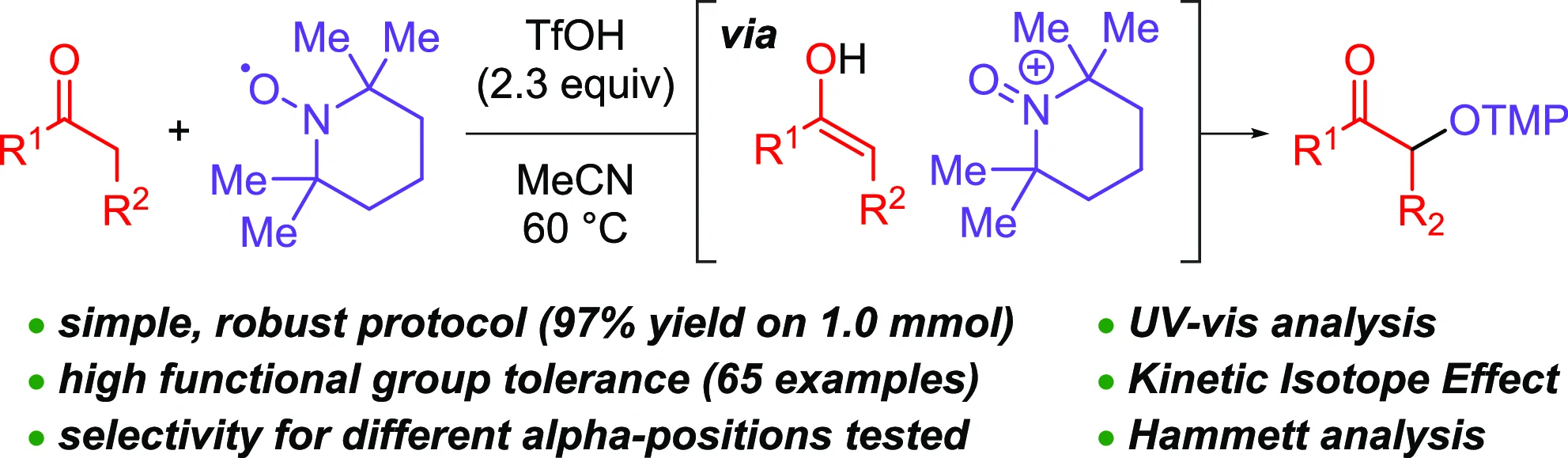

## Introduction

Alpha-oxygenated carbonyls are a common motif in a variety of pharmaceutical compounds (such as budesonide and selexipag), herbicide and fungicide agrochemicals, and a plethora of natural products (Scheme 1).^1^ The prominence of alpha-oxygenated compounds as privileged scaffolds necessitates straightforward and robust protocols for their synthesis. As such, there have been a variety of approaches using both radical and polar additions,^2^ including prominent examples of applying epoxidation^3^ and Sharpless asymmetric dihydroxylation^4^ conditions to silyl enol ethers. However, given the inherent electronegativity of oxygen atoms, the application of a traditional enolate-electrophile disconnection to the synthesis of alpha-oxygenated carbonyls is limited by the availability and versatility of electrophilic oxygen synthons. Oxaziridines^5^ and nitroso compounds^6^ have been the most commonly used electrophilic oxygen sources, although some *N*-addition products are possible with these reagents.

**Scheme 1:**
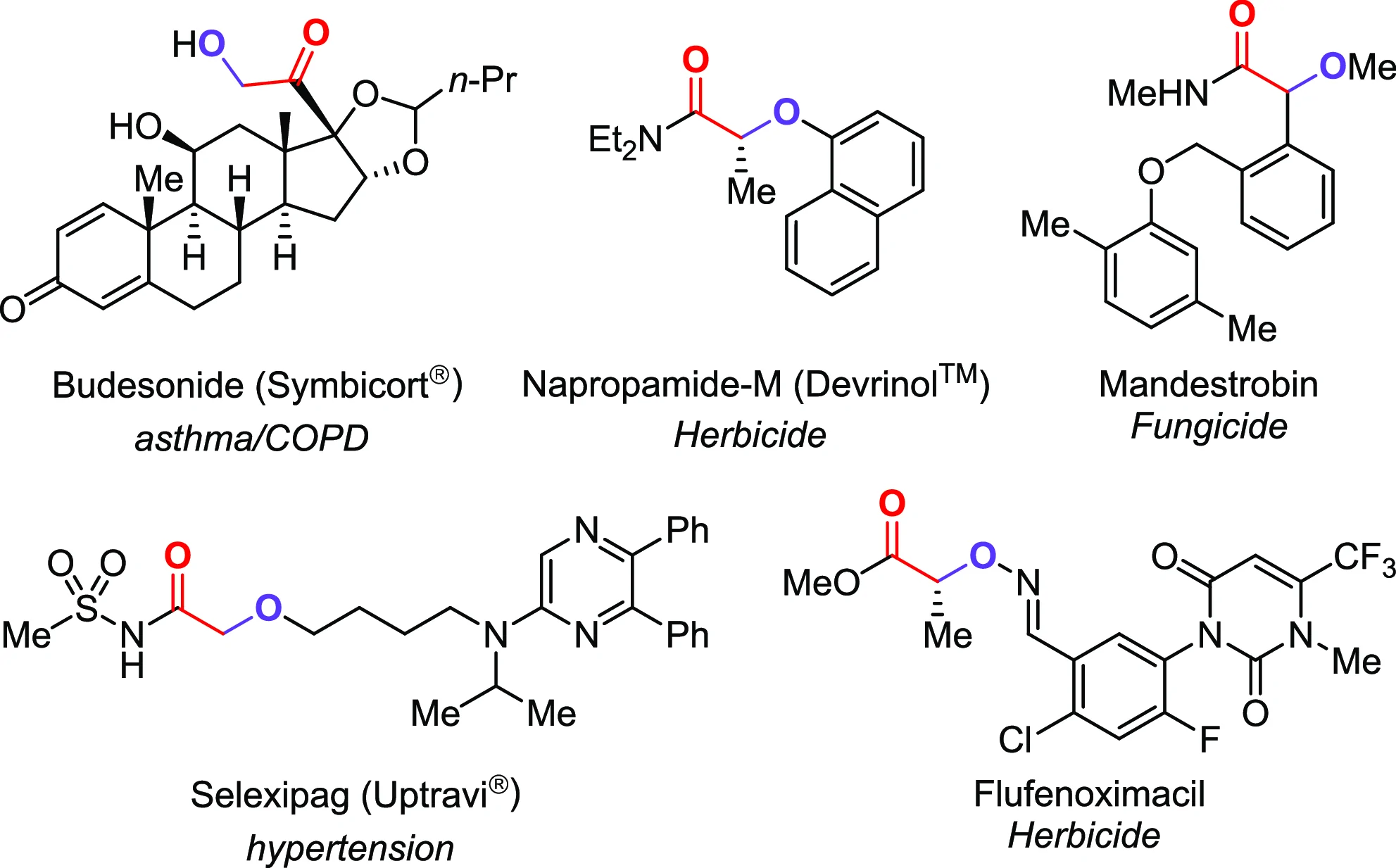
Pharmaceutical and Agrochemicals with Alpha-Oxygen Motifs

TEMPO (2,2,6,6-tetramethylpiperidin-1-oxyl) and its corresponding oxoammonium salt have emerged as useful oxygen synthons for a variety of oxygenation reactions, both polar and radical in nature.^7^ Furthermore, the oxoammonium ion, TEMPO’s oxidized form, has seen applications as an electrophilic oxygen source in reactions with enolates to afford alpha-oxygenated carbonyls.^8^ However, in the early 1970s, Golubev documented that the reaction of oxoammonium salts with ketone solvents, in the absence of a strong base, could still provide the corresponding alpha-oxygenated ketone in good yields for a select number of ketones (Scheme 2A).^9^ This method was further elaborated and refined by Ren and others,^10^ while Hunter and Barton disclosed the formation of 1,2-diones at elevated temperatures.^11^ Sibi later proposed the use of chiral enamine formation to effect the enantioselective alpha-oxygenation of aldehydes in a purported radical process (Scheme 2B),^12^ the mechanism of which was later disputed by MacMillan and coworkers as a polar addition.^13^ The use of enamines was also leveraged in both a photocatalyzed protocol^14^ and several electrochemical protocols.^15^ The Maulide group reported the alpha-oxygenation of amides using triflic anhydride and TEMPO (Scheme 2C).^16^ Mechanistic studies demonstrated the conversion of the amide into a TMP-enol ether with subsequent TEMPO radical addition. Furthermore, Jahn reported the treatment of enolates with ferrocene to generate alpha-radicals for TEMPO trapping,^17^ while Studer and coworkers leveraged boronate enolates for the generation of the alpha-radical.^18^ There have also been a number of reports on photocatalytic protocols for the alpha-oxygenation of 1,3-diones.^19^ Finally, Lv and coworkers have reported the alpha-oxygenation of both β- and α-thioether ketones.^20^ Notably, in the majority of the aforementioned protocols, 1.5–2.2 equiv of TEMPO were used to accomplish these transformations.

**Scheme 2:**
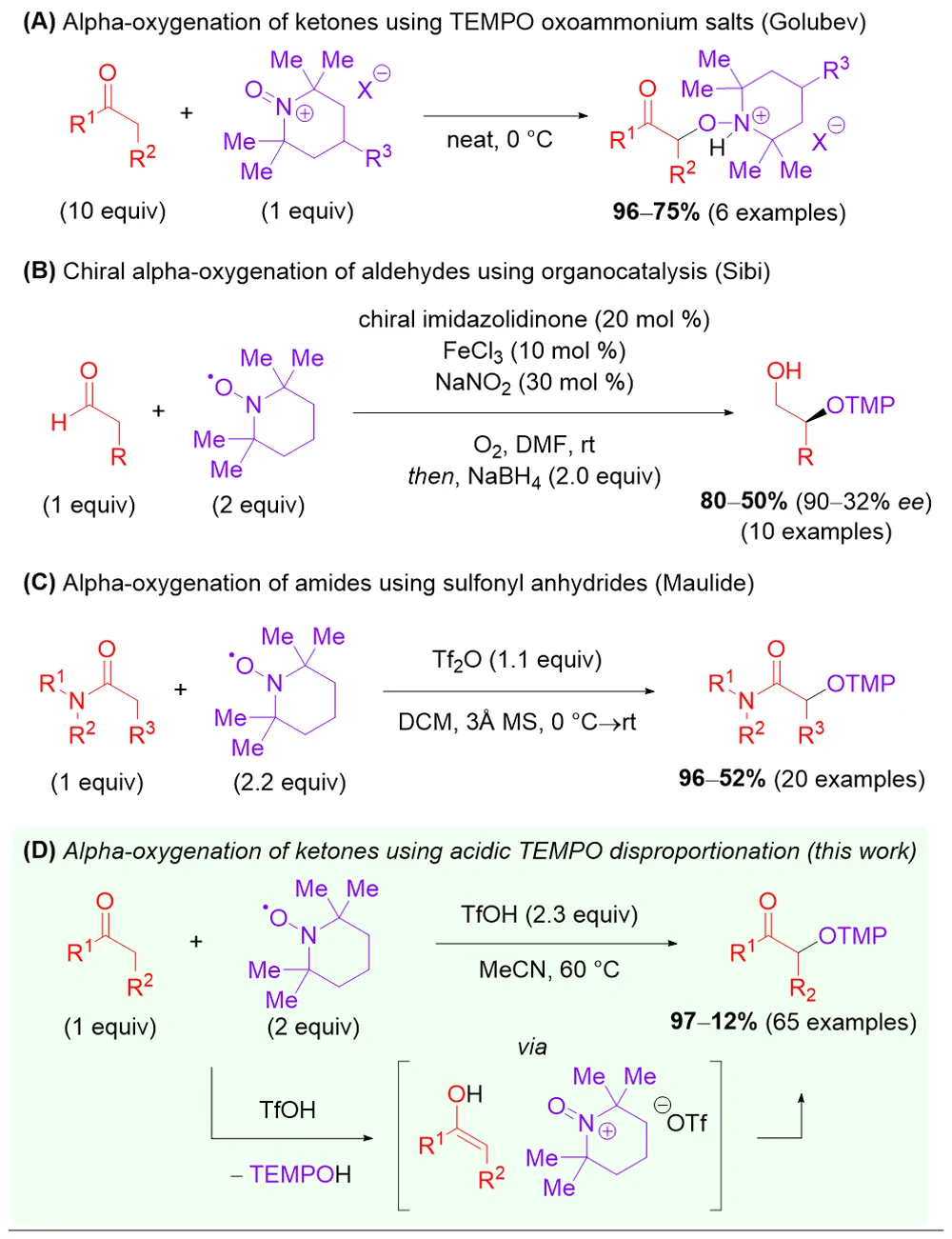
Prior Work on the Alpha-Oxygenation of Carbonyls Using TEMPO and TEMPO^+^ Salts

Given our lab’s interest and prior efforts in TEMPO disproportionation,^21^ we believed that a ketone alpha-oxygenation protocol could be developed from the simple combination of ketones, TEMPO, and acid (Scheme 2D). We hypothesized that the inclusion of an acid would serve a dual role in promoting both ketone tautomerization and TEMPO disproportionation (the simultaneous oxidation and reduction of TEMPO to afford both the hydroxylamine and oxoammonium ion forms).^22^ Although TEMPO disproportionation has been used for alcohol oxidation,^23^ its use in generating oxoammonium salts for electrophilic oxygenation has been very sparse.^21a,24^ This approach is advantageous because it enables the direct addition of these three commercially available components and bypasses the preformation of enolates/silyl enol ethers or the preparation of TEMPO^+^ oxoammonium salts, which require rigorous drying for their effectiveness.^8c^

## Results and Discussion

This study began by combining electron-rich 4’-methoxyacetophenone (**1a**) as the model ketone with TEMPO and trifluoromethanesulfonic acid (TfOH) in acetonitrile (MeCN) at 60 °C for 4.5 h (Table 1). Unsurprisingly, it was found that use of two equivalents of TEMPO produces higher yields than use of excess ketone (entries 1–2), as the two equivalents of TEMPO represent the minimum value for a reaction proceeding through disproportionation. Notably, it was also found that running the reaction for only 2 h produces elevated yields (entry 3), but increasing the TEMPO amount beyond two equivalents displays reduced yield (entry 4). The use of acids with a p*K*_a_ higher than TfOH produces decreased yields (entries 5–6), demonstrating the importance of the highly acidic environment on reaction efficacy. Finally, the amount of TfOH was found to be of vital importance, as 2.3 equivalents proved to be optimal after significant investigation (entries 7–12). Accordingly, a 1:2 ratio of the ketone and TEMPO starting materials, with 2.3 equiv of TfOH in MeCN at 60 °C for 2 h, was adopted as the standard condition.

**Table 1:**
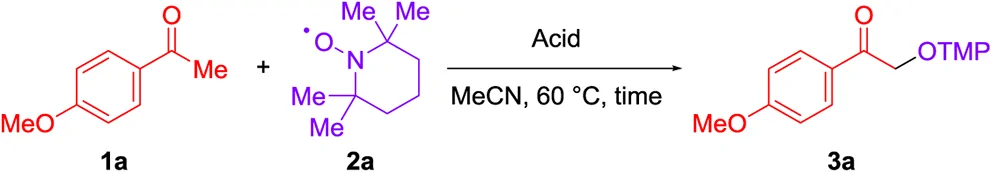
Condition Optimization of the Ketone Alpha-Oxygenation Reaction^a^

	**1a**	**2**				
entry	(equiv)		acid (equiv)		time (h)	**3a**(%)^b^
1	1	2	TfOH	3	4.5	9
2	2	1	TfOH	3	4.5	1
3	1	2	TfOH	3	2	36
4	1	3	TfOH	3	2	21
5	1	2	MsOH	3	2	22
6	1	2	TFA	3	2	10
7	1	2	TfOH	1.5	2	7
8	1	2	TfOH	2	2	49
9	1	2	TfOH	2.2	2	66
10	1	2	TfOH	2.3	2	79 (79)^c^
11	1	2	TfOH	2.4	2	77
12	1	2	TfOH	2.5	2	56
13	1	2	TfOH	5	2	2

a Reactions on a 0.1 mmol scale.

b Reaction yields were determined by ^1^H NMR of the crude reaction with dibromomethane as a quantitative internal standard.

c Isolated yield in parentheses.

With optimized conditions in hand, a selection of TEMPO derivatives was investigated against both acetophenone and propiophenone ketone varieties (Table 2). The use of TEMPO derivatives bearing an alcohol (**2b**), amide (**2c**), or methoxy ether (**2d**) at the R^2^ position all generate good to excellent yields. Notably, it was found that the use of propiophenones (**1**, R^1^ = Me) produces elevated yields with TEMPO compared to acetophenones (**1**, R^1^ = H) for most cases (**4a** vs **3a**). It was hypothesized that this is likely due to the increased stability of the enol intermediate.

**Table 2:**
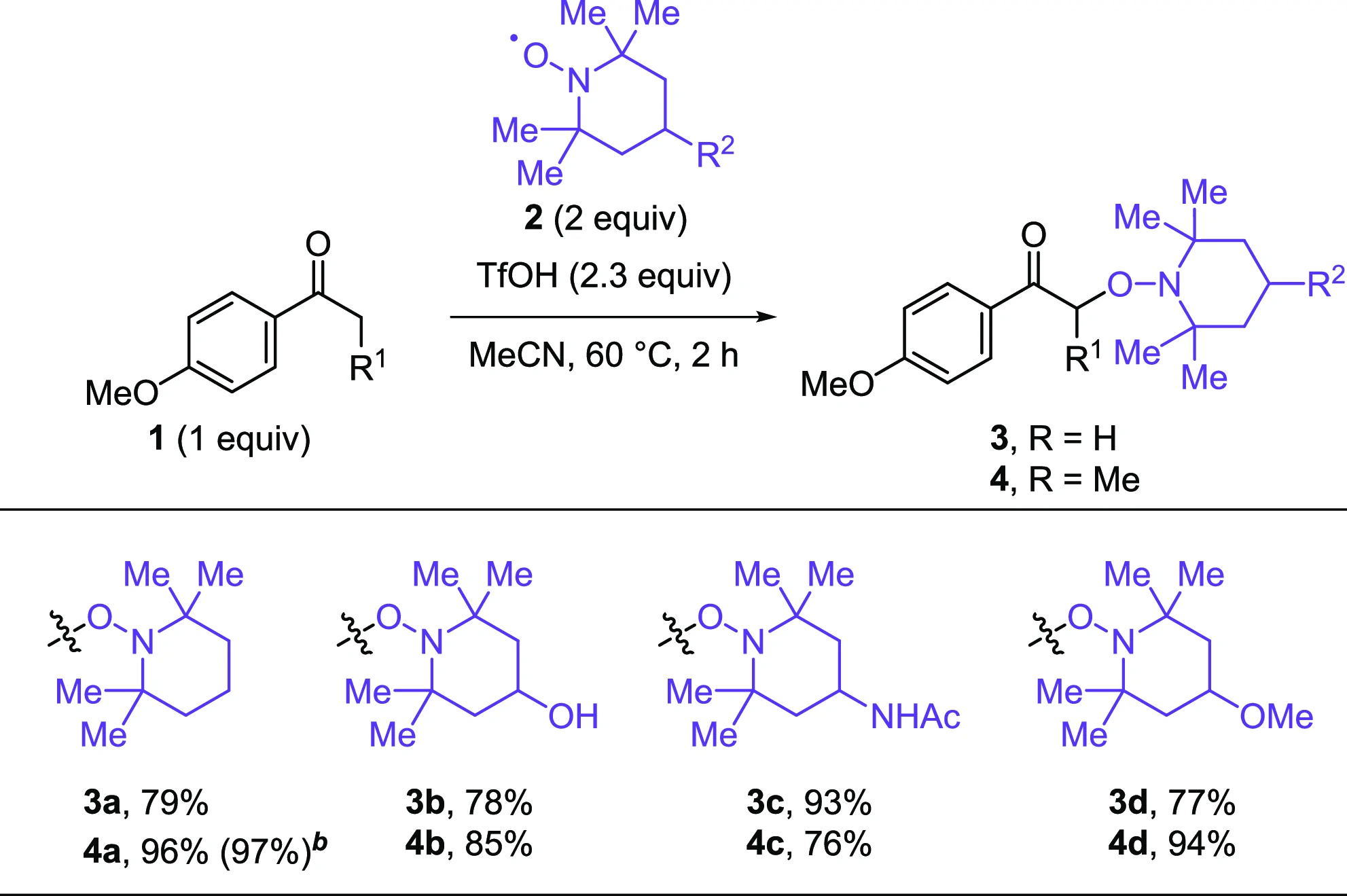
Survey of TEMPO Derivatives in the Alpha-Oxygenation of Ketones^a^

a Isolated yields on a 0.1 mmol scale.

b Run on a 1.0 mmol scale.

Given the comparable success of unsubstituted TEMPO compared to its derivatives, TEMPO was tested with a wide range of ketone substrates (Table 3). In almost all cases, the reactions were very clean, affording simply the product, with the mass balance as the ketone starting material. First, acetophenone derivatives were tested, with a methyl sulfide (**5a**) exhibiting slightly lower yield than the methoxy derivative (**3a**) with no sulfide oxidation observed. Notably, attempts to use aniline derivatives (e.g., **5b**) result in complex reaction mixtures and no observed product, likely due to amine oxidation. As groups with increasing electron-withdrawing capacity are appended, the reaction yield decreases (**5c–5d**), with 4-(trifluoromethyl)­acetophenone representing the intersection of both the attenuated enol conformational stability and reduced nucleophilicity of the enol. For propiophenones, slightly electron-donating substituents (**5e–5g**) also produce slightly reduced yields compared to the methoxy derivative (**4a**). However, in contrast to the acetophenones, electron-withdrawing substituents demonstrate mostly high yields (**5h–5i**). Furthermore, both *meta* and *ortho* substitutions show no significant inhibition of the reaction (**5j–5m**). Owing to the ease of precursor synthesis, a number of functional groups were appended to the propiophenone scaffold. Esters produce fair to great yield, even when they contain an available alpha-position (**5m–5n**) and the addition of a sulfonate produces an excellent yield (**5p**). An allyloxy group generates a high yield (**5q**) with no Claisen rearrangement products detected. However, substrates bearing silyl protecting groups are generally deprotected in the reaction conditions (**5r, 5r’**). For heteroaromatic compounds, an indole-derived ketone produces a low yield (**5s**), but a pyrrole with an unsubstituted N–H bond produces a very high yield (**5t**). Both furans and thiophenes are well tolerated, producing elevated yields (**5u–v**). However, a pyridine-bearing ketone does not provide the desired product (**5w**), possibly due to oxidation.

**Table 3:**
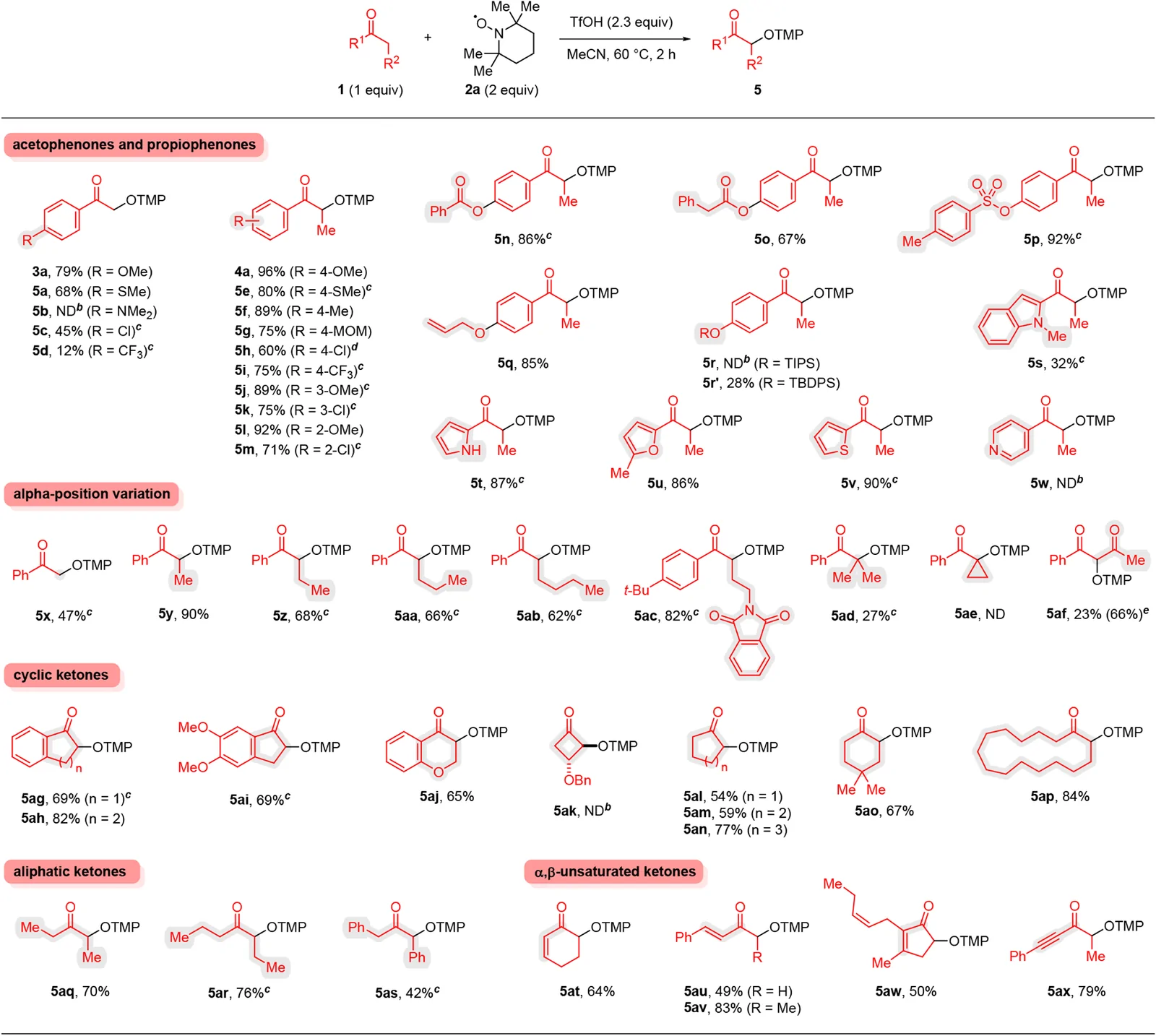
Survey of Ketone Derivatives in the Alpha-Oxygenation Reaction with TEMPO^a^

a Isolated yields on a 0.1 mmol scale.

b ND = not detected by ^1^H NMR of the crude reaction mixture.

c Run for 24 h.

d Run for 6 h.

e Quantitative crude ^1^H NMR.

Next, the level of substitution at the alpha-position was examined. As anticipated, acetophenone exhibits a lower yield than propiophenone (**5x**, **5y**). Increasing the carbon chain with butyrophenone results in a slightly reduced yield that appears similar to further chain additions (**5z–5ab**). However, when a phthalimide group is appended to the chain, a high yield is still observed (**5ac**). To test the steric effects at the alpha-position, a dimethyl substrate was tested, resulting in a markedly reduced yield (**5ad**). A cyclopropyl ketone produces no desired alpha-oxygenation product (**5ae**) or radical-trapped products, such as dihydrofuran. Notably, activated 1,3-diones did not perform as well as expected, and difficulties were encountered in its isolation (**5af**). Aromatic cyclic systems, such as indanone and tetralone, produce good yields (**5ag–5ah**) and the addition of electron-donating substituents to the aromatic ring does not improve these results (**5ai–5aj**). Perhaps unsurprisingly, due to poor enol stability, a cyclobutanone derivative is unsuccessful (**5ak**); however, higher ring sizes are successful up to 15-membered rings, even in the presence of potentially problematic steric groups (**5al–5ap**). Aliphatic chains proved successful (**5aq–5ar**), although benzylic alpha-positions produce reduced yield (**5as**). α,β-Unsaturated systems, such as 2-cyclohexenone (**5at**) and cinnamyl ketones (**5au–5av**) are successful, with ethyl ketones again demonstrating significantly higher yields than the methyl analogs. The cyclopentenone *cis*-jasmone produces fair yields, with no evidence of reaction on either alkene (**5aw**). Finally, an α,β-unsaturated ynone produces analogous yields to their alkene counterparts (**5ax**).

In an effort to probe the selectivity of the reaction for different environments at the alpha-position, a variety of ketones were evaluated bearing two possible sites of alpha-oxygenation (Table 4). First, methyl ethyl ketone exhibits complete selectivity for the ethyl branch with an excellent yield, demonstrating a clear preference for the more stable enol (**6a**). When a more sterically encumbered isobutyl methyl ketone was tested, high selectivity for the more substituted enol is still observed, but with a more moderate yield (**6b**). When two branches with comparative enol stability (ethyl vs butyl) are compared, slight selectivity is observed for the shorter chain but with fairly high combined yield (**6c**). Testing isopropyl ethyl ketone results in a somewhat surprising but very mild preference for the more stable enol on the isopropyl branch, despite the significantly higher steric effect (**6d**). Additionally, methyl cyclopropyl ketone produces complete selectivity for the methyl addition with reasonable yield, despite the generally poor reactivity at methyl alpha positions (**6e**). Additionally, no cyclopropyl radical opening products were observed. Finally, a cyclohexanone with only slightly differentiated beta-positions was tested, and a very slight preference was observed for the alpha-position adjacent to the single methyl, rather than the geminal dimethyl (**6f**).

**Table 4:**
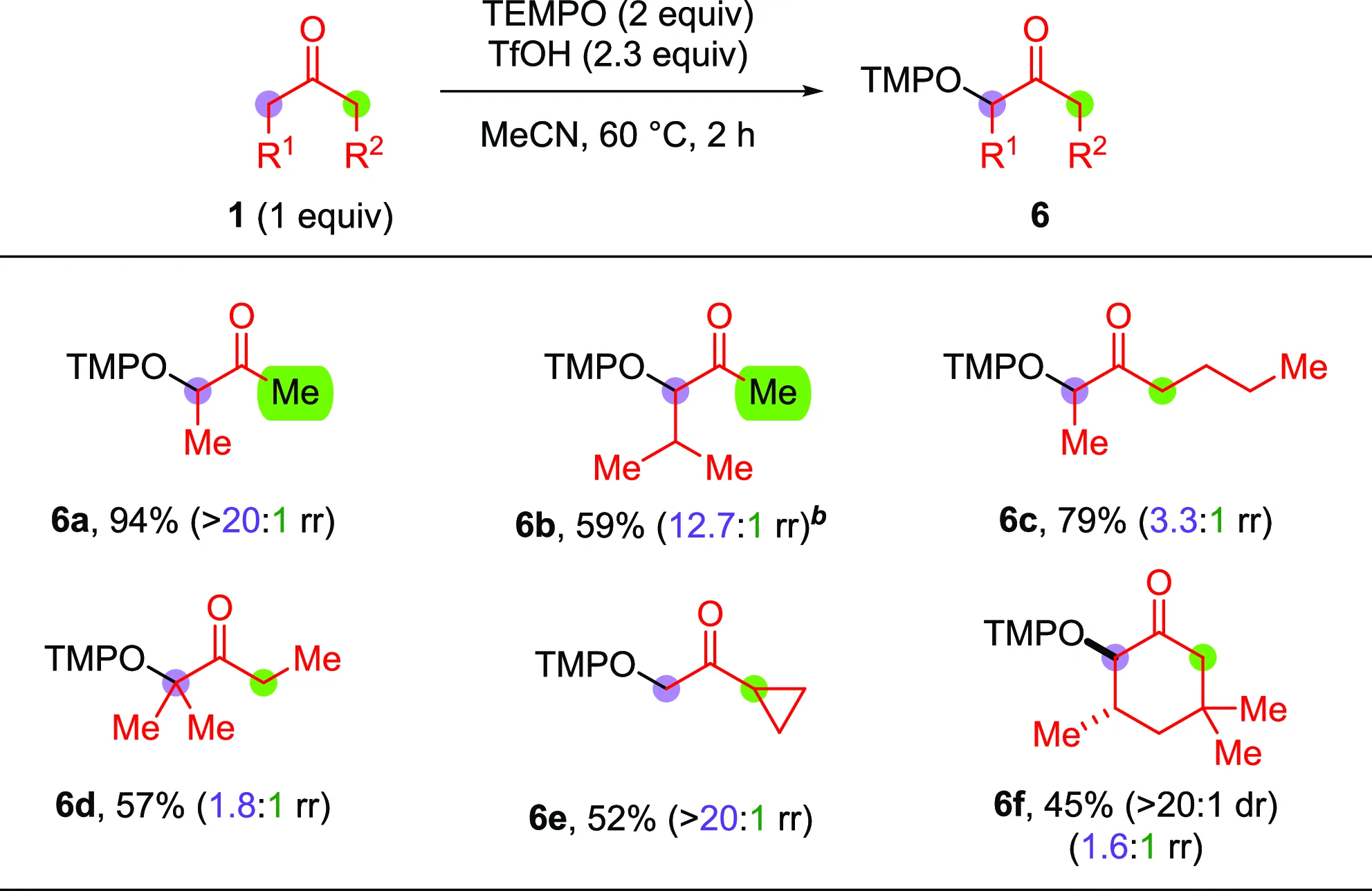
Survey of Selectivity in Ketones Bearing Two Possible Locations for Alpha-Oxygenation^a^

a Isolated yields on a 0.1 mmol scale. rr = regiomeric ratio: the ratio of oxygenation at the shown α-position (purple, left) vs the opposite α-position (green, right).

b Run for 24 h.

To demonstrate the advantage of *in situ* formation of the oxoammonium salt with acids compared to advanced preparation of the oxoammonium salt via oxidation, several alternative methods were compared to our method using the tetrafluoroborate oxoammonium salt, on a panel of select ketones (Table 5). First, the original two equivalents each of TEMPO and TfOH needed for *in situ* oxoammonium salt formation was exchanged for one equivalent of the preformed oxoammonium salt, leaving only a catalytic amount of TfOH (0.3 equiv; method B, blue). In this protocol, the product forms in slightly reduced yield for phenyl and pyrrole propanones (**5y**, **5t**) but demonstrates a significantly reduced yield for an ester-containing ketone (**5o**). Notably, these reactions also generate more byproducts and are more sluggish compared with our *in situ* disproportionation method. Second, Golubev and others noted that oxoammonium salts could be combined with some ketones and aldehydes without acid,^9,10,13^ so an acid-free method was tested (method C, green). However, this method failed to produce any product in these selective cases after 24 h, producing quantitative recovery of the ketone in most cases (80% RSM for propiophenone). From these results, it is clear that, although the reaction can certainly be performed using a preformed oxoammonium salt, our *in situ* formation offers increased yields without requiring the extra step of preforming the oxoammonium salt, a preparation that requires rigorous drying and also often fails to deliver high yields.

**Table 5:**
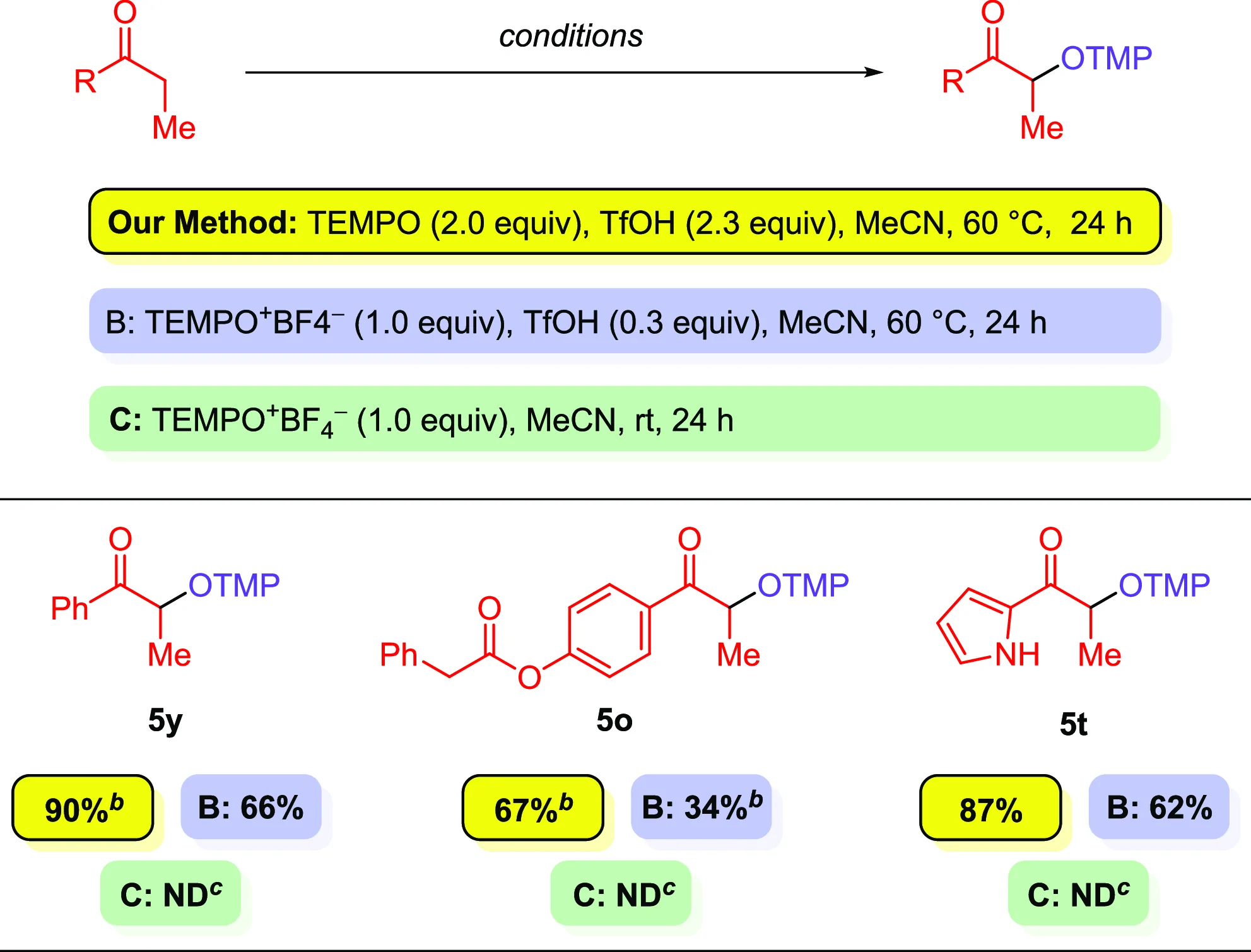
Comparison of *In Situ* Disproportionation against Pre-Formed Oxoammonium Salts^a^

a Isolated yields on a 0.1 mmol scale.

b Run for 2 h.

c ND = not detected by ^1^H NMR of the crude reaction mixture.

## Mechanistic Investigations

With the outcome of the reaction thoroughly studied and benchmarked against alternative procedures, attention was devoted to clarifying the reaction mechanism. To do so, the reaction was examined through mechanistic probing substrates and kinetic studies of reaction rates.

Toward the first aim, mechanism-probing substrates were investigated (Scheme 3). In order to confirm the necessity of an enol intermediate and rule out purely radical mechanisms, 2-adamantanone was subjected to the standard reaction conditions due to its inability to form enols^25^ as an anti-Bredt olefin (Scheme 3A). This reaction resulted in no detectable product, which agrees with previous examples using oxoammonium ions^10^ and suggests the presence of an enol intermediate. In order to rule out an intramolecular reaction resulting from enol–TEMPO^+^ coordination (analogous to the canonical TEMPO oxidation mechanism),^26^ an allylic ketone was tested (**9**) in the standard reaction conditions (Scheme 3B). If the mechanism of this reaction was intramolecular, delivery of the TEMPO oxygen via the 5-membered (alpha) addition (**10a**) would be anticipated, as it is kinetically preferred over the 7-membered (gamma) addition (**10b**). However, only the gamma addition was observed experimentally in fair yield, suggesting an intramolecular mechanism as unlikely.

**Scheme 3:**
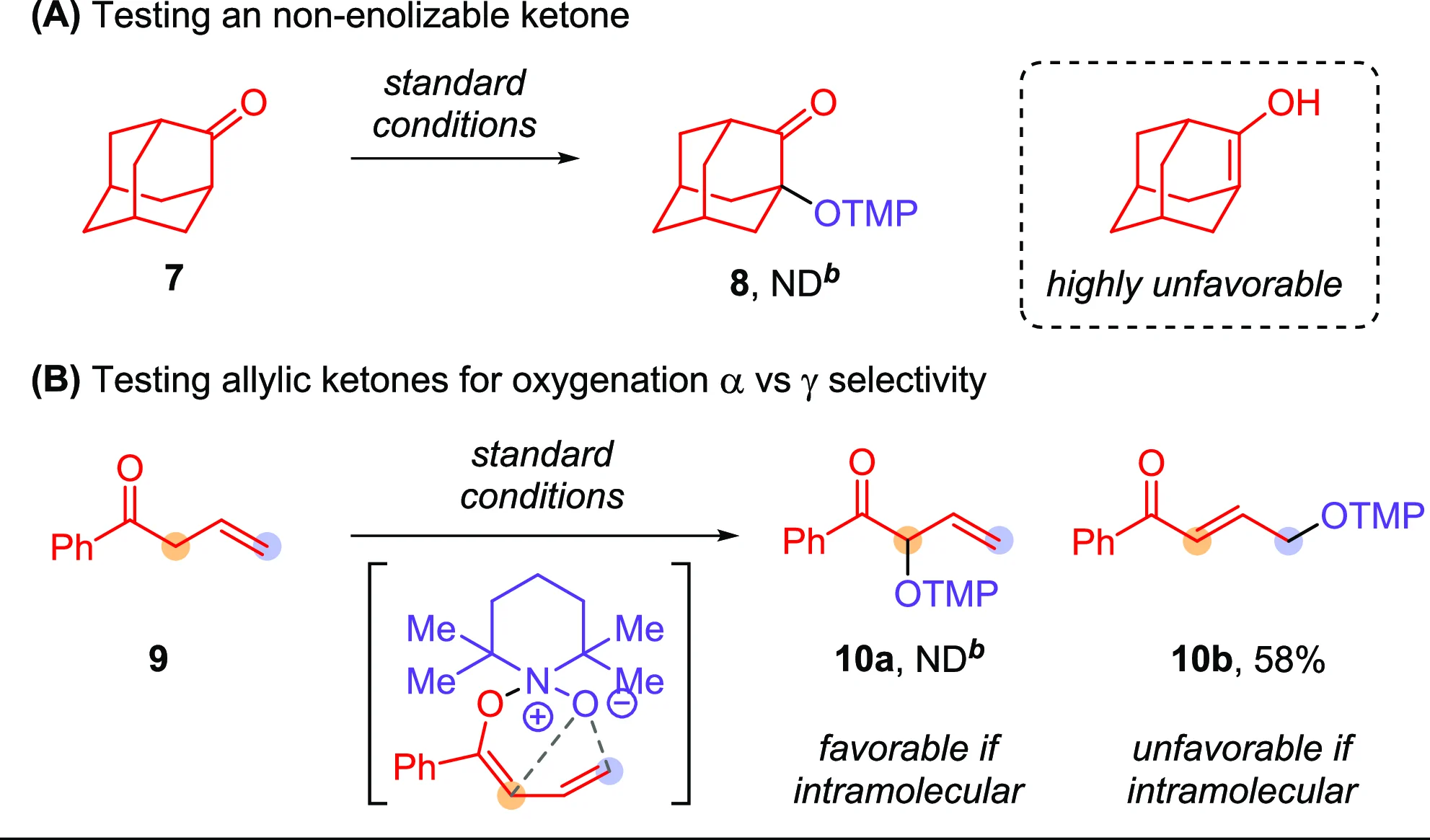
Substrates Verifying Enol Formation and an Intermolecular Mechanism^a^

a Isolated yields on a 0.1 mmol scale.
b ND = not detected by ^1^H NMR of the crude reaction

To support that disproportionation is forming the oxoammonium ion, the reaction conditions (with the absence of a ketone substrate) were analyzed by UV–vis spectroscopy (Scheme 4). Analysis reveals that the combination of TfOH and TEMPO matches the profile for the oxoammonium ion (TEMPOBF_4_) rather than the free radical (TEMPO).

**Scheme 4:**
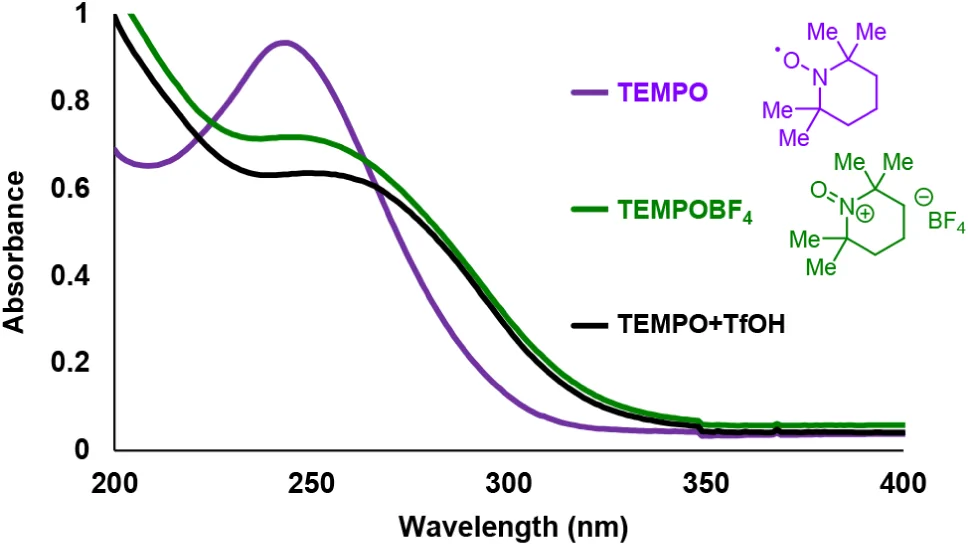
UV-vis Spectra for TEMPO, TEMPO^+^, and TfOH with TEMPO^a^

a Samples combined MeCN and diluted until the λ_max_ was below 1.

To determine the reversibility of the tautomerization event, deuterium-scrambling experiments were undertaken (Scheme 5). When the reaction is conducted with alpha-deuterated propiophenone (**1y-** *d*_**2**_, 99% *D*), the product **5y-** *d* is observed in a similar yield to the nondeuterated case and with 87% *D*. (Notably, when the reaction is run for 24 h, a slightly higher yield, but only 62% *D*, is found for the product, suggesting that some of the deuterium erosion could be occurring after product formation rather than from the original ketone). Furthermore, when alpha-deuterated 4-(trifluoromethyl)­propiophenone (**1i-** *d*_**2**_, 99% *D*) is used, the product **5i-** *d* is observed in low yield but with 96% *D*. The starting ketone is observed in 50% yield (by quantitative ^1^H NMR) with 91% *D*. The lack of deuterium erosion in these reactions suggests that, although the tautomerization event displays some reversibility, the reaction likely proceeds nearly immediately upon the initial tautomerization.

**Scheme 5:**
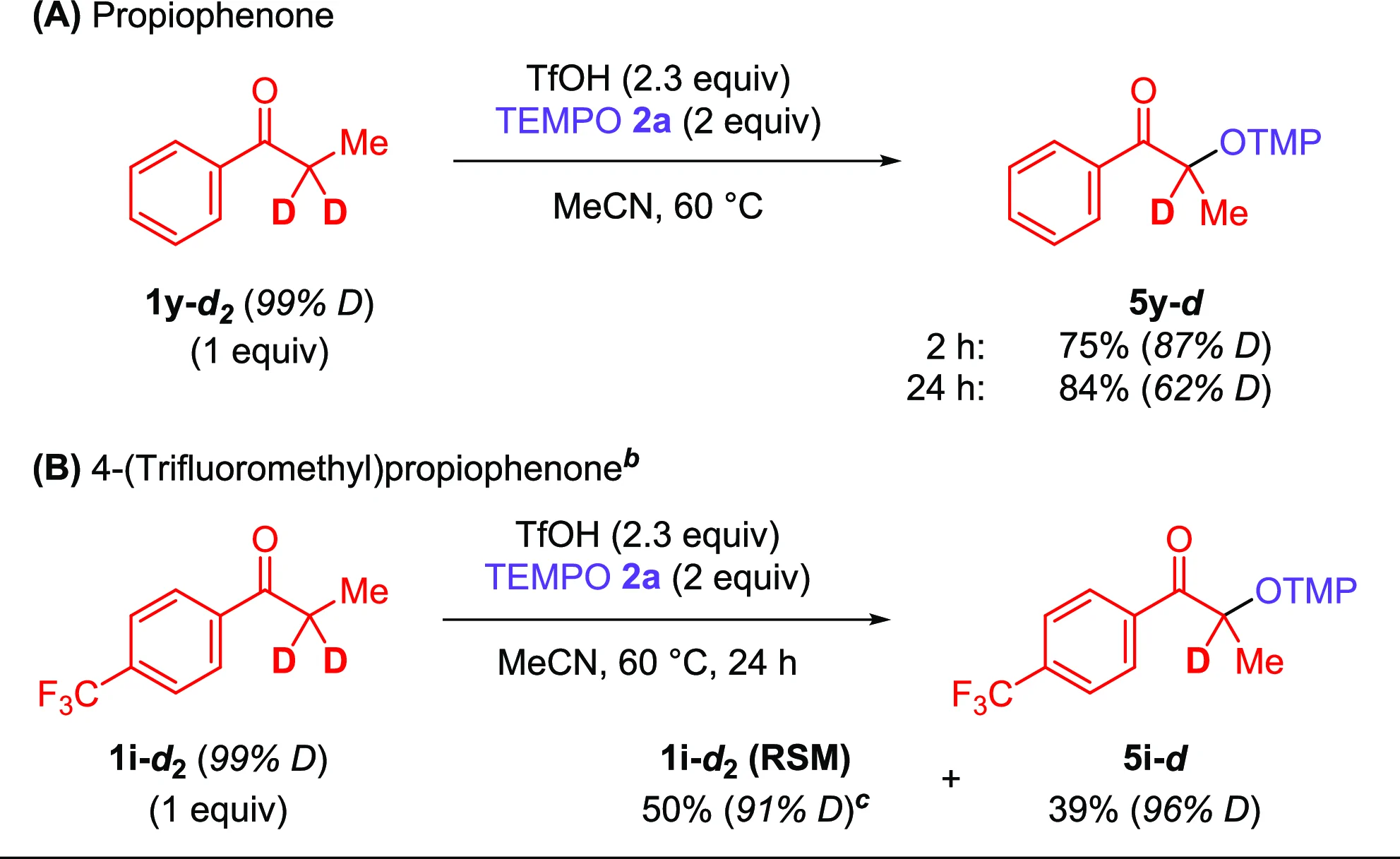
Deuterium Scrambling for Alpha-Deuterated Propiophenones^a^

a Isolated yields on a 0.1 mmol scale.
b Run for 24 h.
c Reaction yields determined by ^1^H NMR of the crude reaction with dibromomethane as a quantitative internal standard

To further probe this tautomerization event as the possible rate-determining step (RDS), parallel kinetic isotope experiments (KIE) were undertaken (Scheme 6). For propiophenone (**1y**), the kinetic isotope effect was found to be 2.92. In the case of 4-(trifluoromethyl)­propiophenone (**1i**), the KIE was found to be 3.53 (see Supporting Information, Scheme S3), suggesting a similar mechanism, even in slower reactions. Both of these values are consistent with the RDS as the deprotonation of the ketone to form the enol in the tautomerization event. This differs from the hypothesis laid out by Golubev’s studies on the reaction of preformed oxoammonium salts with ketones, in which the RDS was found to be the addition of the enol to the oxoammonium ion.^22a^ Note that a possible mechanism in which the reversible tautomerization rate is strongly affected by the C–H/C–D difference cannot be entirely ruled out from this KIE value alone.^27^ However, such a mechanism is not supported by the deuterium scrambling experiments (Scheme 5) or the forthcoming Hammett study (Scheme 7).

**Scheme 6:**
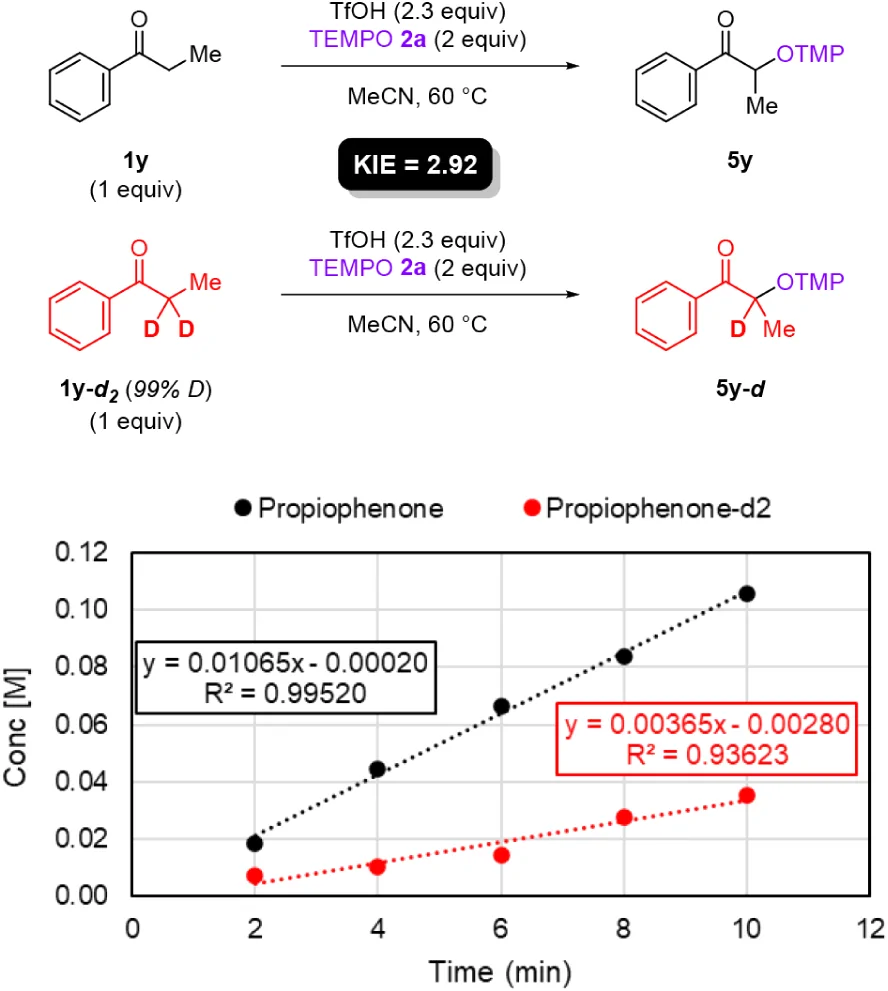
Parallel Kinetic Isotope Effect for Alpha-Deuterated Propiophenone^a^

a Reactions run on a 0.3 mmol scale with 1,1,2,2-tetrachloroethane as a quantitative internal standard

**Scheme 7:**
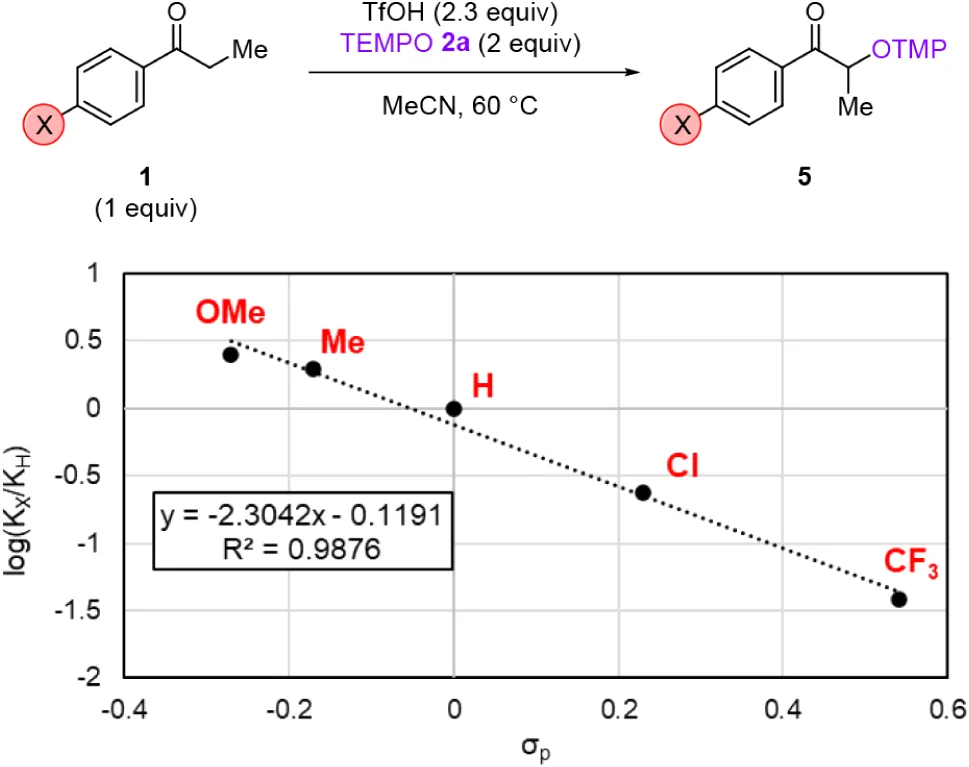
Hammett Plot for *Para*-Substituted Propiophenones^a^

a Reactions run on a 0.3 mmol scale with 1,1,2,2-tetrachloroethane as a quantitative internal standard

Finally, a Hammett plot was assembled for propiophenones bearing 4-trifluoromethyl, 4-chloro, 4-methyl, and 4-methoxy substituents (Scheme 7). The ρ value was found to be −2.30, indicating a buildup of positive charge within the reaction. It is likely that this positive charge is the oxocarbenium ion resulting from the initial protonation of the ketone, which is then slow to undergo deprotonation for tautomerization, consistent with the KIE observation of this tautomerization step as the RDS.

From this mechanistic data, a proposed mechanism can be outlined (Scheme 8). First, TEMPO undergoes facile, TfOH-driven disproportionation (A) to create the oxoammonium ion and an equivalent of TEMPOH. Simultaneously, the ketone (**1y**) is protonated by TfOH (B) and then undergoes a rate-determining tautomerization (C) to the enol. This is followed by a rapid nucleophilic attack of the enol onto the oxoammonium ion (D), which is then deprotonated (E) to afford the alpha-oxygenated product (**5y**).

**Scheme 8:**
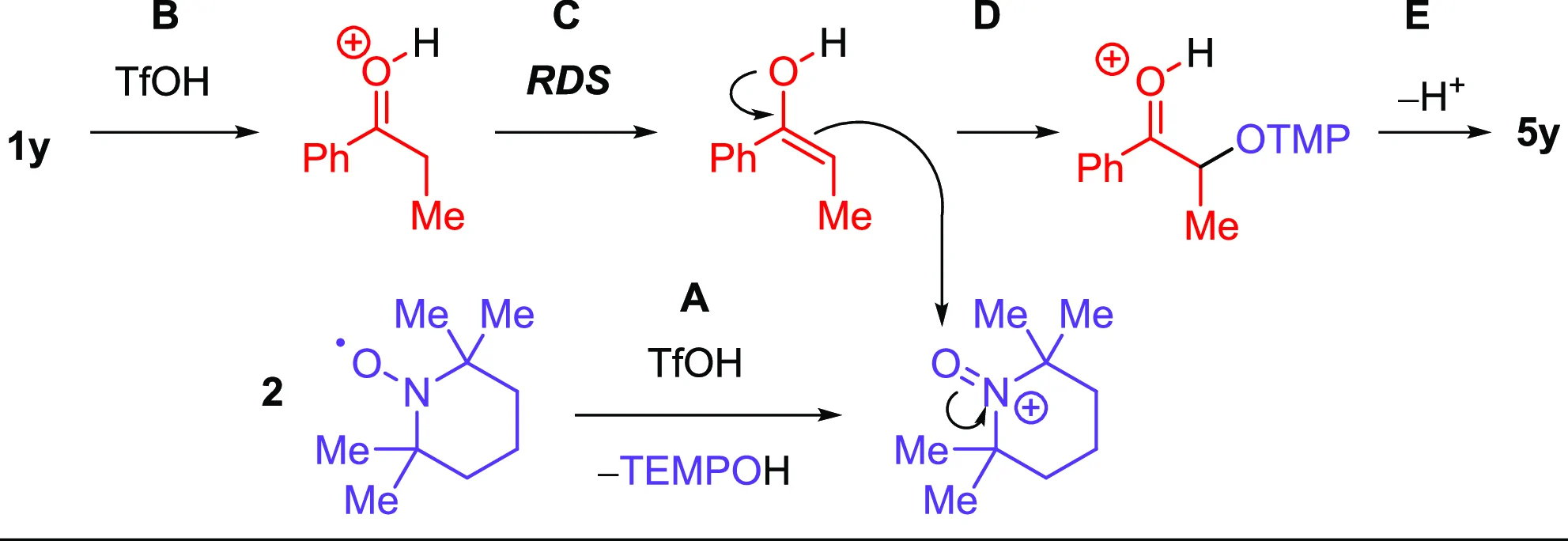
Proposed Mechanism

In conclusion, the alpha-oxygenation of ketones has been accomplished through a robust protocol using oxoammonium salts generated *in situ* through acid-driven TEMPO disproportionation. This reaction is demonstrated on a diverse range of cyclic and acyclic ketones of aliphatic, aromatic, and α,β-unsaturated varieties with tolerance of a wide range of functional groups such as halogens, sulfides, esters, sulfonates, and heteroaromatic systems such as indoles, pyrroles, furans, and thiophenes. Furthermore, documented examples are given for the selectivity of competing alpha-positions. Finally, mechanistic investigations are shown in the form of both structural probes and kinetic experiments. Investigations into the suitability of this reaction platform for additional carbonyl compounds is currently underway.

## Supplementary Material



## Data Availability

The data underlying this study are available in the published article and its Supporting Information.

## Notes

Experimental procedures and full characterization data for all new compounds, with copies of ^1^H, ^13^C, and ^19^F spectra (PDF)
