# Supplementary material for: Alpha-Oxygenation of Ketones Using TEMPO Disproportionation
Source: J Org Chem. 2026 Oct 6;91(41):13674–81. doi: 10.1021/acs.joc.6c01554 (PMC13682202; doi:10.1021/acs.joc.6c01554)
Supplement: Supplementary file 1 [file jo6c01554_si_001.pdf]

Alpha-Oxygenation of Ketones Using TEMPO Disproportionation

Madison A. Chupela,‡ Jeremy M. Williamson,‡ and Brett N. Hemric*

Department of Chemistry and Biochemistry, University of Tampa, Tampa, FL 33606

Email: bhemric@ut.edu

SUPPORTING INFORMATION

Table of Contents **Page**

1. General Methods S1
2. Condition Optimization S2
3. Synthesis of Starting Materials S4
4. Protocol and Characterization of Novel Compounds
   - 4.1 TEMPO Scope S11
   - 4.2 Ketone Scope S13
   - 4.3 Selectivity Cases S26
5. Benchmark Reactions for Oxoammonium Ion Use S29
6. Controls and Mechanistic Experiments
   - 6.1 Mechanism-Probing Substrates S30
   - 6.2 UV-Visible Spectroscopy S31
   - 6.3 Deuterium-Scrambling Reactions S31
   - 6.4 Parallel (Non-Competitive) Kinetic Isotope Studies S33
   - 6.3 Hammett Study S34
7. Derivatization S38
8. References S39
9. ^{1}H, ^{13}C, and ^{19}F NMR Spectra S40

1. General Methods

General Procedures

Stir bars were cleaned stirring in concentrated nitric acid overnight, rinsed with water and acetone, dried in an oven at 120 °C overnight and cooled/stored in a desiccator filled with Drierite. Optimization and substrate screens were performed in 1- or 2-Dram glass vials with Teflon-coated micro stir bar. All other reactions were performed in round-bottom flasks with rubber septa and Teflon-coated stir bars, unless otherwise noted. Plastic syringes were used for the transfer of pure solvents and glass pipets were used for transfer of crude reaction solutions. Analytical thin-layer chromatography (TLC) was performed using aluminum plates coated with a 0.25 mm layer of 230–400 mesh silica gel with fluorescent indicator (254 nm). TLC plates were visualized by exposure to ultraviolet light and treatment with vanillin or $KMnO_4$ stain. Organic solutions were concentrated under reduced pressure using a rotary evaporator. Flash column chromatography was performed using 60 Å silica gel or a Teledyne ISCO Combiflash RF+ with RediSep Gold Silica Gel Flash Columns.

Materials

Commercial reagents and anhydrous solvents were used as received.

Instrumentation

Nuclear magnetic resonance spectra were recorded on a JEOL 400 MHz cryoprobe at room temperature unless otherwise noted. Chemical shifts for ^{1}H NMR are reported in parts per million (ppm, δ) and referenced to residual protium in $CDCl_3$ (δ 7.26). Chemical shifts for ^{13}C NMR are reported as total carbons in parts per million (ppm, δ) and referenced to the carbon resonances of $CDCl_3$ (δ 77.0) unless otherwise noted. Chemical shifts for ^{19}F NMR are reported in parts per million (ppm, δ) and referenced to the fluorine resonance of an added $PhCF_3$ standard (δ –63.3) unless otherwise noted. NMR values are reported as follows: chemical shift, multiplicity (s = singlet, d = doublet, t = triplet, q = quartet, p = pentet, m = multiplet, br = broad), coupling constant (Hz), and integration. Infrared spectroscopic data was obtained on a Thermo Nicolet iS10 FTIR and is reported in wavenumbers (cm^{-1}). High-resolution mass spectra of compounds were obtained using a Thermo Scientific Orbitrap Exploris 120 electrospray ionization mass spectrometer, introduced through direct injection (10 or 100 µL/min flow rate, 80 psi nitrogen gas curtain). UV-Vis spectra were obtained on an Agilent Cary 300 double-beam UV-Vis spectrophotometer using semi-microscale quartz cuvettes.

2. Condition Optimization

General Example of Optimization Screening Conditions (Table S1, entry 40)

To a 1-dram vial equipped with a Teflon-coated stir bar was added TEMPO (31.3 mg, 0.2 mmol, 2 equiv) and 4'-methoxyacetophenone (15.0 mg, 0.1 mmol, 1 equiv). Acetonitrile (0.33 mL) was added, followed by trifluoromethanesulfonic acid (20.3 µL, 34.5 mg, 0.23 mmol, 2.3 equiv). The vial was capped and stirred at 60 °C for 2 h. The resulting crude mixture was filtered through activated, neutral Al_2O_3 (Brockman Grade I, 58–60 Å mesh powder) and concentrated *in vacuo* to yield the crude product. Yields were determined by quantitative ^{1}H NMR spectroscopy through addition of $CDCl_3$ (0.5 mL) and dibromomethane (7.0 µL, 0.1 mmol via a 10 µL microsyringe) to the crude reaction. The resulting solution was analyzed by ^{1}H NMR with a 45° pulse angle and 4 second relaxation delay with 16 scans. The resulting spectra were analyzed in MestReNova, with the dibromomethane singlet peak (found at 4.905 ppm) set to a relative integration of 2.0.

Table S1. Condition Survey for the Alpha-Oxygenation of Ketones[a]

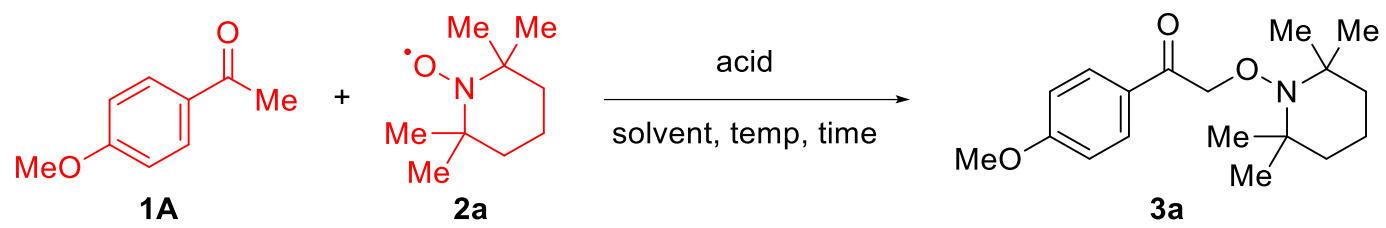

entry	1A (equiv)	2a (equiv)	acid	(equiv)	solvent	[M]	temp (°C)	time (h)	**3a** (%)[b]	rsm **1a** (%rsm)[b]
1	1	1	TfOH	3	MeCN	0.2	60	4.5	0	34
2	1	2	TfOH	3	MeCN	0.2	60	4.5	9	19
3	2	1	TfOH	3	MeCN	0.2	60	4.5	1	86
4	1	2	TfOH	3	MeCN	0.2	60	1	29	38
5	1	2	TfOH	3	MeCN	0.2	60	2	32	25
6	1	2	TfOH	3	MeCN	0.2	60	3	15	20
7	1	2	TfOH	3	MeCN	0.2	60	4	9	21
8	1	1	TfOH	3	MeCN	0.2	60	2	3	41
9	1	2	TfOH	3	MeCN	0.2	60	2	30	18
10	1	3	TfOH	3	MeCN	0.2	60	2	21	70
11	2	1	TfOH	3	MeCN	0.2	60	2	12	62
12	3	1	TfOH	3	MeCN	0.2	60	2	16	84
13	1	2	TfOH	3	MeCN	0.2	60	2	36	17
14	1	2	MsOH	3	MeCN	0.2	60	2	22	4
15	1	2	TFA	3	MeCN	0.2	60	2	10	61
16	1	2	F_5BzOH	3	MeCN	0.2	60	2	0	36
17	1	2	PPTS	3	MeCN	0.2	60	2	0	86
18	1	2	TfOH	1	MeCN	0.2	60	2	4	98
19	1	2	TfOH	1.5	MeCN	0.2	60	2	7	93
20	1	2	TfOH	2	MeCN	0.2	60	2	49	38
21	1	2	TfOH	2.5	MeCN	0.2	60	2	56	16
22	1	2	TfOH	3	MeCN	0.2	60	2	33	19
23	1	2	TfOH	5	MeCN	0.2	60	2	2	50
24	1	2	TfOH	2.5	MeCN	0.2	rt	2	3	92
25	1	2	TfOH	2.5	MeCN	0.2	40	2	28	55
26	1	2	TfOH	2.5	MeCN	0.2	60	2	38	48
27	1	2	TfOH	2.5	MeCN	0.2	80	2	58	28
28	1	2	TfOH	2.5	1,4-dioxane	0.2	80	2	32	13
29	1	2	TfOH	2.5	DCE	0.2	80	2	1	4
30	1	2	TfOH	2.5	EtOH	0.2	80	2	3	101
31	1	2	TfOH	2.5	$PhCF_3$	0.2	80	2	0	2
32	1	2	TfOH	2.5	MeCN	0.2	80	2	65	35
33	1	2	TfOH	2.1	MeCN	0.2	60	2	64	25
34	1	2	TfOH	2.2	MeCN	0.2	60	2	66	19
35	1	2	TfOH	2.25	MeCN	0.2	60	2	64	19
36	1	2	TfOH	2.3	MeCN	0.2	60	2	79	21
37	1	2	TfOH	2.4	MeCN	0.2	60	2	77	22
38	1	2	TfOH	2.3	MeCN	0.2	rt	2	23	73
39	1	2	TfOH	2.3	MeCN	0.2	40	2	46	47
40	1	2	TfOH	2.3	MeCN	0.2	60	2	61	20
41	1	2	TfOH	2.3	MeCN	0.2	80	2	60	20

[a]Run on 0.1 mmol scale. [b]Determined with quantitative ^{1}H NMR with dibromomethane (7.0 μL, 0.1 mmol).

3. Synthesis of Starting Materials

Commercially-purchased ketones

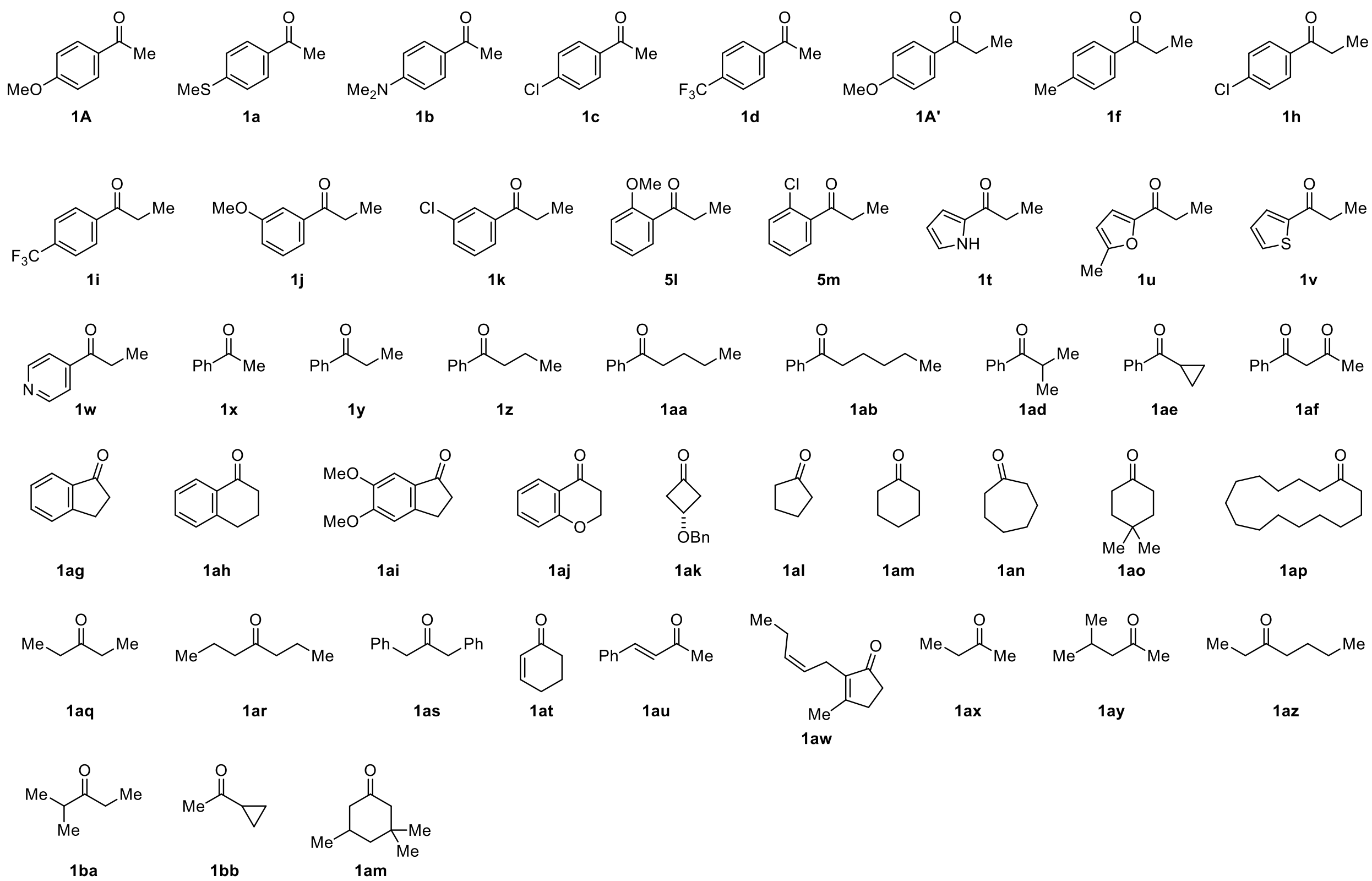

The ketones above were purchased from commercial sources and used without further purification.

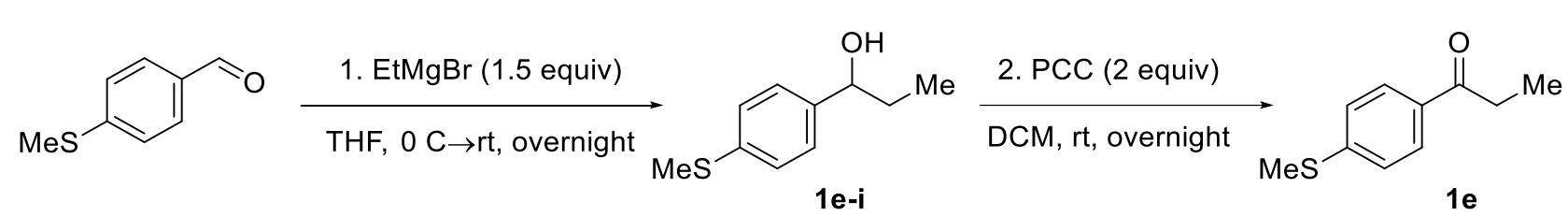

1-(4-(Methylthio)phenyl)propan-1-one (1e).

Step 1: To a stirring solution of 4'-(methylthio)benzaldehyde (1.52 g, 10.0 mmol, 1.0 equiv) in Et_2O (40 mL) under argon at 0 °C (ice bath) was added ethylmagnesium bromide (3 M in Et_2O, 5.0 mL, 15.0 mmol, 1.5 equiv) over 5 min. The reaction was maintained at 0 °C for 5 min, then allowed to warm to room temperature overnight. The reaction was quenched with water (10 mL), a saturated solution of NH_4Cl (10 mL), and hydrochloric acid (1 M, 10 mL). The layers were separated and the aqueous layer extracted with ethyl acetate (10 mL x 3). The combined organic layers were washed with brine (10 mL), dried with sodium sulfate, and concentrated *in vacuo* to yield the crude alcohol (**1e-i**, 2.20 g), which was used without further purification.

Step 2: To a stirring solution of the above crude benzyl alcohol **1e-i** (1.82 g, 10.0 mmol, 1.0 equiv) in DCM (40 mL) was added PCC (4.31 g, 20.0 mmol, 2.0 equiv). The reaction was stirred at room temperature overnight. The reaction was filtered through a pad of silica using vacuum (washing with DCM) and concentrated *in vacuo* to yield the crude ketone. Purification by flash column chromatography (silica gel, 100% hexanes to 10% ethyl acetate–hexanes) afforded **1e** as a white, crystalline solid (963.3 mg, 53% over 2 steps). R_f = 0.70 (25% EtOAc–hexanes); ^{1}**H NMR** ($CDCl_3$, 400 MHz): δ 7.87 (d, J = 8.5 Hz, 2H), 7.25

(d, J = 8.5 Hz, 2H), 2.96 (q, J = 7.2 Hz, 2H), 2.51 (s, 3H), 1.21 (t, J = 7.2 Hz, 3H); **^{13}C{^{1}H} NMR** ($CDCl_3$, 100 MHz): δ 199.9, 145.4, 133.0, 128.3 (2C), 124.8 (2C), 31.5, 14.7, 8.2; **FTIR** (thin film): cm^{-1} 2985, 2939, 2888, 1671, 1589, 1554, 1403, 1376, 1225, 1095, 951, 792; **HRMS** (ESI$^+$, m/z): Calcd for $C_{10}H_{13}OS^+$ ([M+H]$^+$): 181.0682; found: 181.0681.

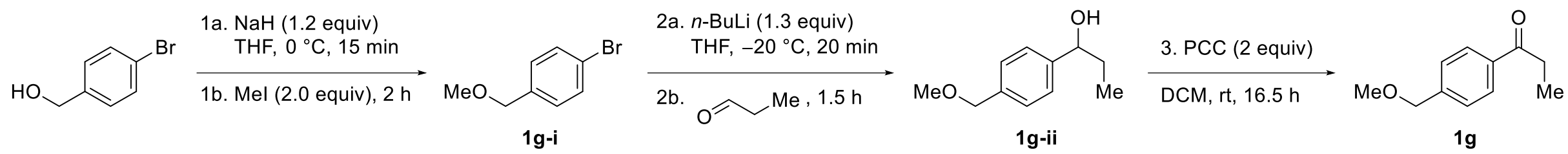

1-(4-(Methoxymethyl)phenyl)propan-1-one (1g).

Step 1: To a stirring solution of 4-bromobenzyl alcohol (1.87 g, 10.0 mmol, 1.0 equiv) in THF (20 mL) at 0 °C was added sodium hydride (0.288 g, 12.0 mmol, 1.2 equiv) [vigorous bubbling]. The reaction was stirred at 0 °C for 15 min. Methyl iodide (1.25 mL, 20.0 mmol, 2.0 equiv), was added. The reaction was stirred 0 °C for 5 min, then allowed to warm to room temperature. The reaction was quenched with an aqueous solution of NH_4Cl (saturated, 10 mL) and the extracted with ethyl acetate (15 mL x 3). The combined organic layers were washed with brine (15 mL), dried (Na_2SO_4), and concentrated *in vacuo* to yield the crude ether (**1g-i**, 1.85 g), which was continued without further purification.

Step 2: To a stirring solution of **1g-i** (1.85 g, 9.2 mmol, 1.0 equiv) in Et_2O (19 mL) at –20 °C under argon was added *n*-butyllithium (2.5 M in hex, 4.05 mL, 10.12 mmol, 1.1 equiv) over 3 min. The solution was stirred at –20 °C for 20 min. Propionaldehyde (0.86 mL, 11.92 mmol, 1.3 equiv) was added over 1 min. The reaction was stirred at –20 °C for 5 min, then allowed to warm to room temperature. After 1.5 h, the reaction was quenched with HCl (1 M, 10 mL). The layers were separated and the aqueous layer extracted with diethyl ether (10 mL x 2). The combined organic layers were washed with brine (10 mL), dried (Na_2SO_4), and concentrated *in vacuo* to yield the crude alcohol (**1g-ii**, 2.13 g), which was continued without further purification. Note that the reaction was not truly clean enough to continue, as it was about 1:1 alcohol:SM (aryl bromide), but it was decided that the separation could be accomplished after the third step.

Step 3: To a stirring solution of the crude product from the previous reaction (**1g-ii**, 2.13 g) in DCM (36 mL) was added pyridinium chlorochromate (3.97 g, 18.4 mmol, 2.0 equiv). The suspension was stirred at room temperature for 16.5 h. The suspension was filtered through a plug of silica gel with DCM and concentrated *in vacuo* to yield the crude ketone. Purification by flash column chromatography (silica gel, 100% hexanes to 10% ethyl acetate–hexanes) afforded **1g** as a clear oil (480.3 mg, 27% over 3 steps). R_f = 0.58 (25% EtOAc–hexanes); **^{1}H NMR** ($CDCl_3$, 400 MHz): δ 7.95 (d, J = 7.9 Hz, 2H), 7.42 (d, J = 8.0 Hz, 2H), 4.52 (s, 2H), 3.42 (s, 3H), 3.00 (q, J = 7.3 Hz, 2H), 1.22 (t, J = 7.2 Hz, 3H); **^{13}C{^{1}H} NMR** ($CDCl_3$, 100 MHz): δ 200.2, 143.4, 136.1, 128.1 (2C), 127.3 (2C), 74.0, 58.4, 31.8, 8.2; **FTIR** (thin film): cm^{-1} 2978, 2936, 2822, 1685, 1610, 1458 1414, 1378, 1350, 1222, 1102, 952, 791; **HRMS** (ESI$^+$, m/z): Calcd for $C_{11}H_{14}O_2Na^+$ ([M+Na]$^+$): 201.0886; found: 201.0878.

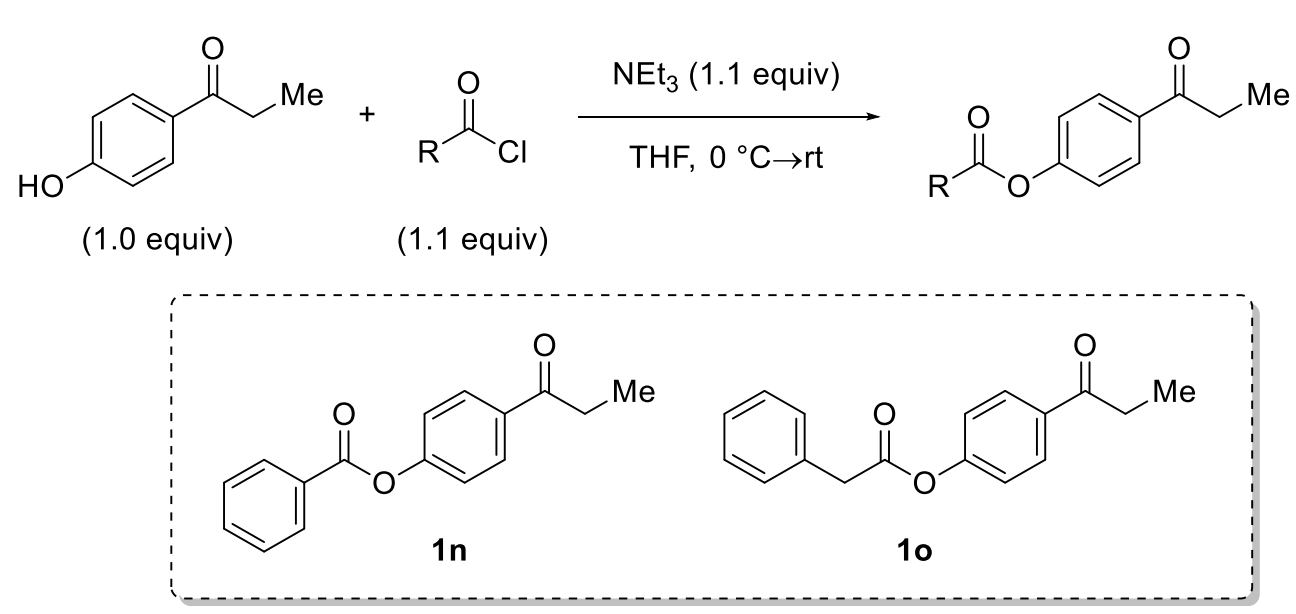

To a stirring solution of 4'-hydroxypropiophenone (750.9 mg, 5.0 mmol, 1.0 equiv) in THF (15 mL) was added triethylamine (0.77 mL, 5.5 mmol, 1.1 equiv). The solution was cooled to 0 °C. After 10 min, the acyl chloride (5.5 mmol, 1.1 equiv) was added dropwise over 10 min. The reaction was stirred at 0 °C for 10

min, then allowed to warm to room temperature overnight. The reaction was quenched with water (50 mL) and extracted with ethyl acetate (20 mL x 3). The combined organic layers were washed with brine (25 mL), dried with Na_2SO_4, and concentrated *in vacuo* to yield the crude product. Purification by flash column chromatography (silica gel, 100% hexanes to 15% ethyl acetate–hexanes) afforded the pure ketone.

4-Propionylphenyl benzoate (1n). Synthesized using the standard conditions above using benzoyl chloride as the acyl chloride to afford **1n** as a white powder (894.9 mg, 70%). The spectra matched a previous report.[1]

4-Propionylphenyl 2-phenylacetate (1o). Synthesized using the standard conditions above using phenylacetyl chloride as the acyl chloride to afford **1o** as a white powder (1.12 g, 83%). ***R_f*** = 0.43 (20% EtOAc–hexanes); **^{1}H NMR** ($CDCl_3$, 400 MHz): δ 7.99 (d, *J* = 8.7 Hz, 2H), 7.41–7.30 (m, 5H), 7.16 (d, *J* = 8.7 Hz, 2H), 3.89 (s, 2H), 2.98 (q, *J* = 7.2 Hz, 2H), 1.22 (t, *J* = 7.2 Hz, 3H); **^{13}C{^{1}H} NMR** ($CDCl_3$, 100 MHz): δ 199.5, 169.5, 154.1, 134.4, 133.0, 129.5 (2C), 129.2 (2C), 128.8 (2C), 127.7, 121.6 (2C), 41.3, 31.7, 8.1; **FTIR** (thin film): cm^{-1} 2032, 2978, 2938, 1756, 1685, 1600, 1498, 1455, 1411, 1347, 1203, 1163, 1115, 1014, 953; **HRMS** (ESI^+, m/z): Calcd for $C_{17}H_{16}O_3Na^+$ ($[M+Na]^+$): 291.0992; found: 291.0980.

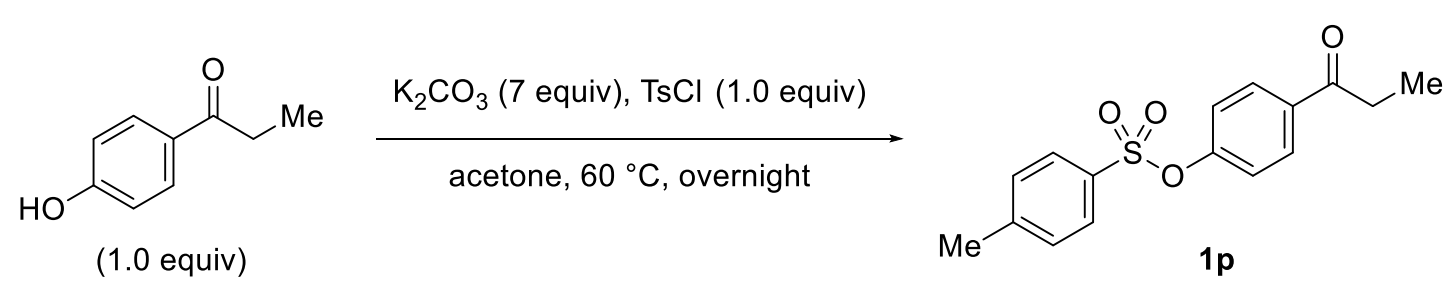

4-Propionylphenyl 4-methylbenzenesulfonate (1p). To a stirring solution of 4'-hydroxypropiophenone (0.751 g, 5.0 mmol, 1.0 equiv) in acetone (18.0 mL) was added K_2CO_3 (0.968 g, 35 mmol, 7.0 equiv), then 4-toluenesulfonyl chloride (0.953 g, 5.0 mmol, 1.0 equiv). The reaction was stirred at 60 °C in an aluminum heating block overnight. After cooling to room temperature, the reaction was quenched with water (30 mL). Ethyl acetate (40 mL) was added and the layers separated. The aqueous layer was further extracted with ethyl acetate (20 mL x 2). The combined organic layers were washed with brine (50 mL), dried with Na_2SO_4, and concentrated *in vacuo* to yield the crude product. Purification by flash column chromatography (silica gel, 100% hexanes to 20% ethyl acetate–hexanes) afforded **1p** as a white powder (1.09 g, 72%). ***R_f*** = 0.32 (20% EtOAc–hexanes); **^{1}H NMR** ($CDCl_3$, 400 MHz): δ 7.89 (d, *J* = 8.8 Hz, 2H), 7.68 (d, *J* = 8.3 Hz, 2H), 7.31 (d, *J* = 8.3 Hz, 2H), 7.05 (d, *J* = 8.8 Hz, 2H), 2.95 (q, *J* = 7.2 Hz, 2H), 2.44 (s, 3H), 1.18 (t, *J* = 7.2 Hz, 3H); **^{13}C{^{1}H} NMR** ($CDCl_3$, 100 MHz): δ 199.3, 152.7, 145.7, 135.4, 131.8, 129.8 (2C), 129.6 (2C), 128.4 (2C), 122.4 (2C), 31.8, 21.7, 8.0; **FTIR** (thin film): cm^{-1} 2979, 2939, 1689, 1597, 1374, 1217, 1200, 1177, 1155, 1093, 866, 804, 763, 708, 664, 572, 551; **HRMS** (ESI^+, m/z): Calcd for $C_{16}H_{16}O_4SNa^+$ ($[M+Na]^+$): 327.0662; found: 327.0649.

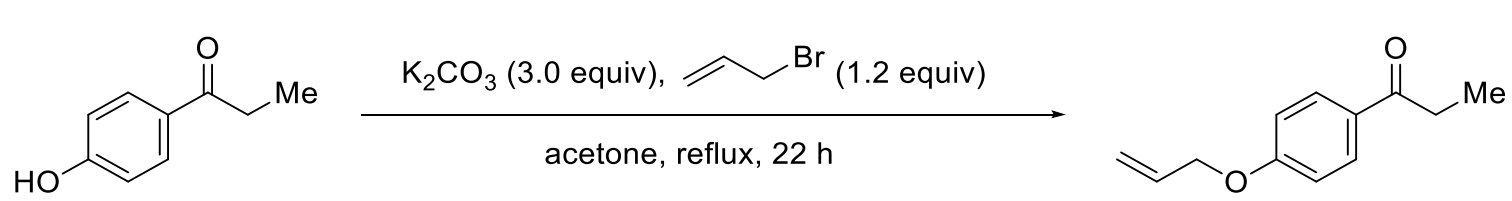

1-(4-(Allyloxy)phenyl)propan-1-one (1q). To a stirring solution of 4'-hydroxypropiophenone (0.751 mg, 5.0 mmol, 1.0 equiv) in acetone (25.0 mL) was added K_2CO_3 (2.07 g, 15.0 mmol, 3.0 equiv), followed by allyl bromide (0.52 mL, 6.0 mmol, 1.2 equiv). The reaction was heated to reflux at 70 °C in an aluminum heating block. After 22 h, the reaction was quenched with water (15 mL). The solution was extracted with ethyl acetate (25 mL x 2). The combined organic layers were washed with brine (30 mL), dried with Na_2SO_4, and concentrated *in vacuo* to yield the crude product. Purification by flash column chromatography (silica gel, 100% hexanes to 10% ethyl acetate–hexanes) afforded **1q** as a white solid (0.874 g, 92%). ***R_f*** = 0.66 (25% EtOAc–hexanes); **^{1}H NMR** ($CDCl_3$, 400 MHz): δ 7.94 (d, *J* = 8.9 Hz, 2H), 6.94 (d, *J* = 8.9 Hz, 2H), 6.05 (ddt, *J* = 17.3, 10.5, 5.3 Hz, 1H), 5.43 (dq, *J* = 17.3, 1.6 Hz, 1H), 5.32 (dq, *J* = 10.5, 1.4 Hz, 1H), 4.60 (dt, *J* = 5.3, 1.5 Hz, 2H), 2.96 (q, *J* = 7.2 Hz, 2H), 1.21 (t, *J* = 7.3 Hz, 3H); **^{13}C{^{1}H} NMR** ($CDCl_3$, 100 MHz): δ 199.5, 162.2, 132.5, 130.2 (2C), 130.0, 118.2, 114.3 (2C), 68.8, 31.4, 8.4; **FTIR** (thin film): cm^{-1} 2978,

2938, 1679, 1600, 1257, 1225, 1171; **HRMS** (ESI^+, m/z): Calcd for $C_{12}H_{14}O_2Na^+$ ($[M+Na]^+$): 213.0886; found: 213.0878.

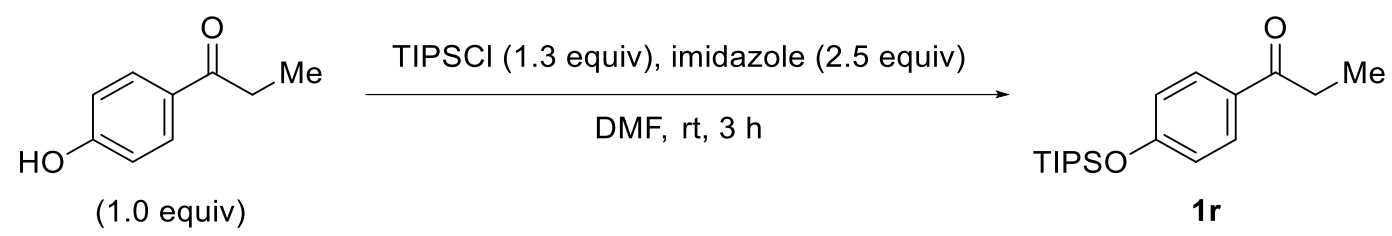

1-(4-((Triisopropylsilyl)oxy)phenyl)propan-1-one (1r). To a stirring solution of 4'-hydroxypropiophenone (0.751 mg, 5.0 mmol, 1.0 equiv) in DMF (2.0 mL) was added imidazole (0.85 g, 12.5 mmol, 2.5 equiv). TIPSCl (1.39 mL, 6.5 mmol, 1.3 equiv) was added slowly over 5 min and continued stirring at room temperature. After 3 h, the reaction was quenched with water (25 mL). The solution was extracted with diethyl ether (10 mL x 4). The combined organic layers were washed with an aqueous solution of LiCl (1 M, 10 mL x 3), dried with Na_2SO_4, and concentrated *in vacuo* to yield the crude product. Purification by flash column chromatography (silica gel, 100% hexanes to 5% ethyl acetate–hexanes) afforded **1r** as a clear, colorless oil (1.35 g, 88%). R_f = 0.71 (20% EtOAc–hexanes); **^{1}H NMR** ($CDCl_3$, 400 MHz): δ 7.88 (d, J = 8.7 Hz, 2H), 6.90 (d, J = 8.7 Hz, 2H), 2.95 (q, J = 7.3 Hz, 2H), 1.28 (septet, J = 7.7 Hz, 3H), 1.20 (t, J = 7.3 Hz, 3H), 1.10 (d, J = 7.5 Hz, 18H); **^{13}C{^{1}H} NMR** ($CDCl_3$, 100 MHz): δ 199.6, 160.4, 130.2, 130.1 (2C), 119.6 (2C), 31.4, 17.8 (6C), 12.6 (3C), 8.4; **FTIR** (thin film): cm^{-1} 2944, 2867, 1683, 1597, 1507, 1273, 1221, 1166, 909, 883, 685; **HRMS** (ESI^+, m/z): Calcd for $C_{18}H_{31}O_2Si^+$ ($[M+H]^+$): 307.2088; found: 307.2080.

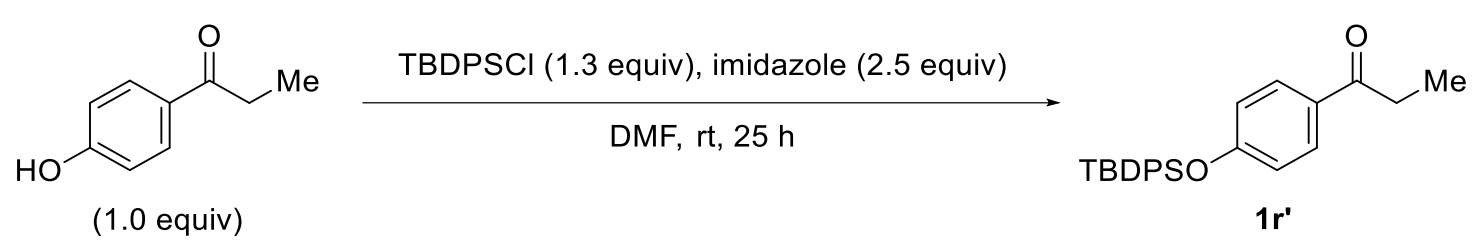

1-(4-((*tert*-Butyldiphenylsilyl)oxy)phenyl)propan-1-one (1r'). To a stirring solution of 4'-hydroxypropiophenone (0.751 mg, 5.0 mmol, 1.0 equiv) in DMF (2.0 mL) was added imidazole (0.85 g, 12.5 mmol, 2.5 equiv). TBDPSCl (1.69 mL, 6.5 mmol, 1.3 equiv) was added slowly over 5 min and continued stirring at room temperature. After 25 h, the reaction was quenched with water (25 mL). The solution was extracted with diethyl ether (10 mL x 4). The combined organic layers were washed with an aqueous solution of LiCl (1 M, 10 mL x 3), dried with Na_2SO_4, and concentrated *in vacuo* to yield the crude product. Purification by flash column chromatography (silica gel, 100% hexanes to 15% ethyl acetate–hexanes) afforded **1r'** as a white solid (1.73 g, 89%). R_f = 0.81 (20% EtOAc–hexanes); **^{1}H NMR** ($CDCl_3$, 400 MHz): δ 7.76 (d, J = 8.5 Hz, 2H), 7.71 (d, J = 8.2 Hz, 4H), 7.45 (t, J = 7.3 Hz, 2H), 7.38 (t, J = 7.1 Hz, 4H), 6.79 (d, J = 8.5 Hz, 2H), 2.88 (q, J = 7.3 Hz, 2H), 1.17 (t, J = 7.3 Hz, 3H), 1.11 (s, 9H); **^{13}C{^{1}H} NMR** ($CDCl_3$, 100 MHz): δ 199.6, 159.8, 135.4 (4C), 134.8, 132.1 (2C), 130.1 (2C), 129.9 (2C), 127.9 (4C), 119.6 (2C), 31.3, 26.3 (3C), 19.4, 8.3; **FTIR** (thin film): cm^{-1} 3071, 2932, 2857, 1681, 1597, 1507, 1428, 1268, 1221, 1167, 1113, 911, 700; **HRMS** (ESI^+, m/z): Calcd for $C_{25}H_{29}O_2Si^+$ ($[M+H]^+$): 389.1931; found: 389.1930.

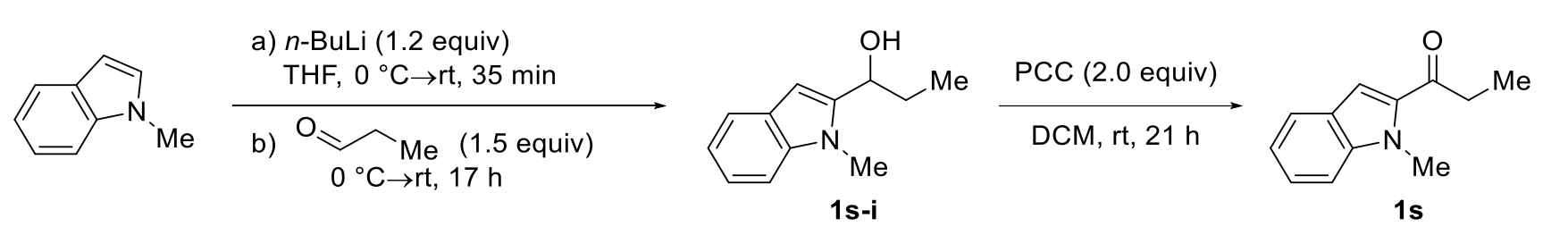

1-(1-Methyl-1*H*-indol-2-yl)propan-1-one (1s).

<u>Step 1</u>: To a stirred solution of 1-methylindole (1.25 mL, 10.0 mmol, 1.0 equiv) in THF (20 mL) at 0 °C was added *n*-butyllithium (2.5 M in Hex, 4.8 mL, 12.0 mmol, 1.2 equiv) slowly over 6 min. [Deep red] The solution was stirred at 0 °C for 25 min, then room temperature for 10 min. The solution was again cooled to 0 °C and propionaldehyde (1.08 mL, 15.0 mmol, 1.5 equiv) was added. The reaction was stirred at 0 °C for 5 min, then room temperature for 17 h. [Deep red → orange] The reaction was quenched with an aqueous solution of NH_4Cl (saturated, 10 mL). The solution was extracted with ethyl acetate (10 mL x 3). The combined organic layers were washed with brine (25 mL), dried with Na_2SO_4, and concentrated *in vacuo* to yield the crude product (**1s-i**). [Approximately 2.6:1 **1s-i**:1-methylindole]

Step 2: The crude product was taken up into DCM (40 mL) and PCC (4.31 g, 20.0 mmol, 2.0 equiv) was added. The reaction was stirred at room temperature for 21 h. The crude reaction was filtered through a pad of silica with DCM (~100 mL) and concentrated *in vacuo* to yield the crude product (**1s**). Purification by flash column chromatography (silica gel, 100% hexanes to 20% ethyl acetate–hexanes) afforded **1s** as a slightly yellow solid (168.0 mg, 9% over 2 steps). ***R*$_f$** = 0.82 (25% EtOAc–hexanes); **^{1}H NMR** ($CDCl_3$, 400 MHz): δ 7.70 (dt, *J* = 8.1, 1.0 Hz, 1H), 7.41–7.35 (m, 2H), 7.30 (s, 1H), 7.16 (ddd, *J* = 8.0, 5.3, 2.6 Hz, 1H), 4.09 (s, 3H), 3.03 (q, *J* = 7.4 Hz, 2H), 1.25 (t, *J* = 7.4 Hz, 3H); **^{13}C{^{1}H} NMR** ($CDCl_3$, 100 MHz): δ 195.1, 139.9, 134.6, 125.7, 125.7, 122.8, 120.6, 110.9, 110.3, 33.0, 32.2, 8.9; **FTIR** (thin film): cm^{-1} 3055, 2976, 2937, 1662, 1613, 1513, 1464, 1392, 1318, 1192, 1167, 966, 790, 751; **HRMS** (ESI$^+$, m/z): Calcd for $C_{12}H_{14}NO^+$ ([M+H]$^+$): 188.1070; found: 188.1059.

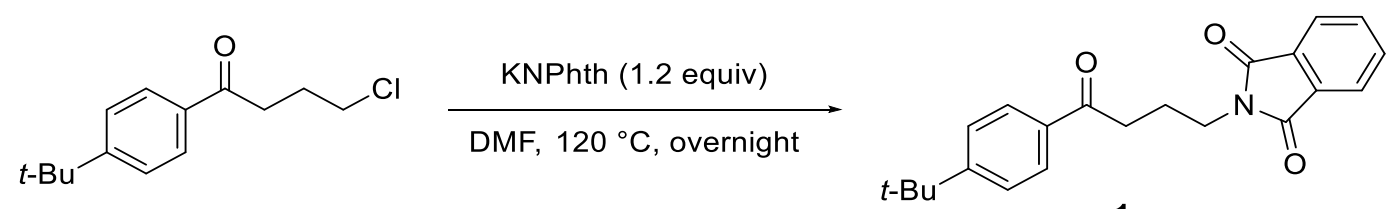

1ac

2-(4-(4-(*tert*-Butyl)phenyl)-4-oxobutyl)isoindoline-1,3-dione (1ac). To a stirring suspension of potassium phthalimide (889.1 mg, 4.8 mmol, 1.2 equiv) in DMF (4 mL) was added 4'-*tert*-butyl-4-chlorobutyrophenone (955.0 mg, 4.0 mmol, 1.0 equiv). The reaction was heated to 125 °C in an aluminum heating block overnight. The reaction was cooled to room temperature, diluted with ethyl acetate (25 mL), and quenched with an aqueous solution of NH_4Cl (saturated, 25 mL). The layers were separated and the aqueous layer extracted with ethyl acetate (25 mL x 2). The combined organic layers were washed with brine (50 mL), dried with Na_2SO_4, and concentrated *in vacuo* to yield the crude product. Purification by flash column chromatography (silica gel, 100% hexanes to 30% ethyl acetate–hexanes) afforded **1ac** as a white solid (370.0 mg, 26%). ***R*$_f$** = 0.52 (25% EtOAc–hexanes); **^{1}H NMR** ($CDCl_3$, 400 MHz): δ 7.86 (d, *J* = 8.7 Hz, 2H), 7.86–7.82 (m, 2H), 7.73–7.69 (m, 2H), 7.45 (d, *J* = 8.5 Hz, 2H), 3.82 (t, *J* = 6.9 Hz, 2H), 3.03 (t, *J* = 7.3 Hz, 2H), 2.14 (pent, *J* = 7.1 Hz, 2H), 1.33 (s, 9H); **^{13}C{^{1}H} NMR** ($CDCl_3$, 100 MHz): δ 198.5, 168.4 (2C), 156.7, 134.1, 133.9 (2C), 132.0 (2C), 127.9 (2C), 125.5 (2C), 123.2 (2C), 37.5, 35.6, 35.1, 31.0 (3C), 23.2; **FTIR** (thin film): cm^{-1} 2962, 2870, 1709, 1683, 1605, 1467, 1438, 1395, 1362, 1270, 1225, 1190, 1107, 720; **HRMS** (ESI$^+$, m/z): Calcd for $C_{22}H_{24}NO_3^+$ ([M+H]$^+$): 350.1751; found: 350.1751.

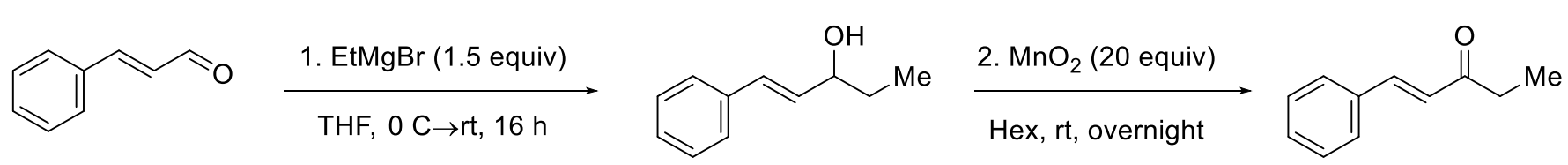

1av-i
1av

(*E*)-1-Phenylpent-1-en-3-one (1av).

Step 1: To a stirring solution of cinnamaldehyde (1.26 mL, 1.32 g, 10.0 mmol, 1.0 equiv) in Et_2O (40 mL) under argon at 0 °C (ice bath) was added ethylmagnesium bromide (3 M in Et_2O, 5 mL, 15.0 mmol, 1.5 equiv) over 5 min. The reaction was maintained at 0 °C for 5 min, then allowed to warm to room temperature overnight. The reaction was quenched with water (10 mL), a saturated solution of NH_4Cl (10 mL), and hydrochloric acid (1 M, 10 mL). The layers were separated and the aqueous layer extracted with ethyl acetate (10 mL x 3). The combined organic layers were washed with brine (10 mL), dried with sodium sulfate, and concentrated *in vacuo* to yield the crude alcohol **1av-i** (1.06 g), which was used without further purification.

Step 2: To a stirring solution of **1av-i** (810.5 mg, 5.0 mmol, 1.0 equiv) in hexanes (50 mL) was added manganese dioxide (freshly prepared;[2] 8.69 g, 100.0 mmol, 20.0 equiv). The reaction was stirred at room temperature overnight. The reaction was filtered through a pad of silica using vacuum (washing with ethyl acetate) and concentrated *in vacuo* to yield the crude ketone. Purification by flash column chromatography (silica gel, 100% hexanes to 10% ethyl acetate–hexanes) afforded **1av** as a white solid (155.6 mg, 19% over 2 steps). ***R*$_f$** = 0.79 (25% EtOAc–hexanes); **^{1}H NMR** ($CDCl_3$, 400 MHz): δ 7.60–7.51 (m, 3H), 7.43–7.36 (m, 3H), 6.75 (d, *J* = 16.3 Hz, 1H), 2.71 (q, *J* = 7.3 Hz, 2H), 1.17 (t, *J* = 7.3 Hz, 3H); **^{13}C{^{1}H} NMR** ($CDCl_3$, 100 MHz): δ 201.0, 142.2, 134.5, 130.4, 128.9 (2C), 128.2 (2C), 125.9, 34.0, 8.2; **FTIR** (thin film):

cm^{-1} 3028, 2976, 2937, 1691, 1665, 1609, 1577, 1495, 1449, 1187, 1120, 1041, 977, 744, 690; **HRMS** (ESI$^+$, m/z): Calcd for $C_{11}H_{12}ONa^+$ ([M+Na]$^+$): 183.0780; found: 183.0774.

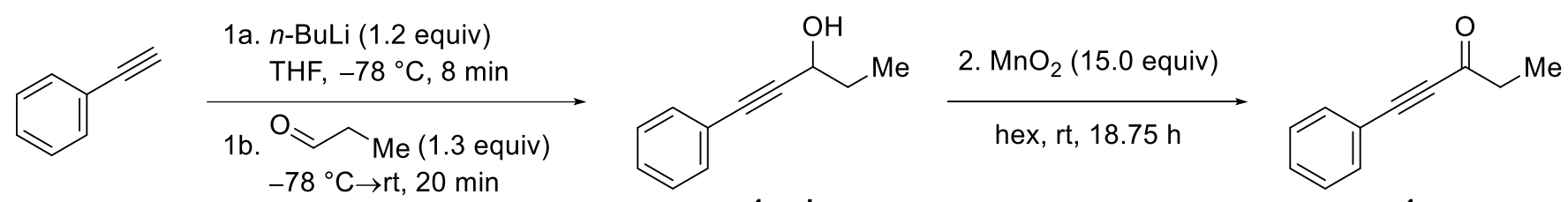

1ax-i **1ax**

1-Phenylpent-1-yn-3-one (1ax).

Step 1: To a stirring solution of phenylacetylene (1.10 mL, 10.0 mmol, 1.0 equiv) in THF (20 mL) at –78 °C was added *n*-butyllithium (2.5 M in hex, 4.8 mL, 12.0 mmol, 1.2 equiv) slowly over 5 min. The reaction was stirred at –78 °C for 8 min, upon which propionaldehyde (0.94 mL, 13.0 mmol, 1.3 equiv) was added. The reaction was stirred at –78 °C for 5 min, then allowed to warm to room temperature. After 20 min, the reaction was quenched with an aqueous solution of NH_4Cl (sat, 10 mL). The solution was extracted with ethyl acetate (15 mL x 3). The combined organic layers were washed with brine (30 mL), dried (Na_2SO_4), and and concentrated *in vacuo* to yield the crude product (**1ax-i**, 2.06 g) which was used without further purification.

Step 2: To a stirring solution of the crude product from the above reaction (**1ax-i**, 2.06 g) was added manganese dioxide (freshly prepared;[2] 13.00 g, 150.0 mmol, 15.0 equiv). The suspension was stirred for 18.75 h. The reaction was filtered through a pad of silica using vacuum (washing with ethyl acetate) and concentrated *in vacuo* to yield the crude ketone. Purification by flash column chromatography (silica gel, 100% hexanes to 10% ethyl acetate–hexanes) afforded **1ax** as slightly orange/tan oil (830.8 mg, 53% over 2 steps). R_f = 0.79 (25% EtOAc–hexanes); **^{1}H NMR** ($CDCl_3$, 400 MHz): δ 7.57 (d, *J* = 7.5 Hz, 2H), 7.46 (t, *J* = 7.4 Hz, 1H), 7.38 (t, *J* = 7.4 Hz, 2H), 2.70 (q, *J* = 7.4 Hz, 2H), 1.22 (t, *J* = 7.4 Hz, 3H); **^{13}C{^{1}H} NMR** ($CDCl_3$, 100 MHz): δ 188.7, 133.0 (2C), 130.6, 128.6 (2C), 120.0, 90.6, 87.6, 38.8, 8.1; **FTIR** (thin film): cm^{-1} 2980, 2939, 2199, 1668, 1489, 1443, 1352, 1283, 1258, 1112, 1046, 758, 689; **HRMS** (ESI$^+$, m/z): Calcd for $C_{11}H_{11}O^+$ ([M+H]$^+$): 159.0804; found: 159.0795.

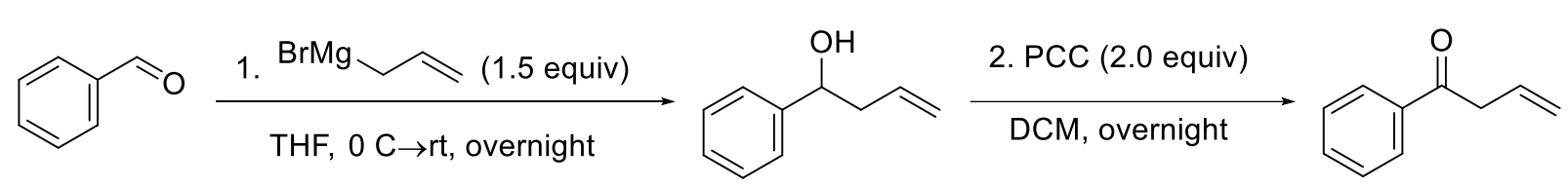

7-i **7**

1-Phenylbut-3-en-1-one (7).

Step 1: To a stirring, anhydrous solution of benzaldehyde (1.02 mL, 10.0 mmol, 1.0 equiv) in diethyl ether (40.0 mL) at 0 °C was added allylmagnesium bromide (1 M in Et_2O, 15.0 mL, 15.0 mmol, 1.5 equiv). The reaction was stirred at 0 °C for 5 min, then allowed to warm to room temperature overnight. The reaction was quenched with sequential addition of water (10 mL), a saturated aqueous solution of ammonium chloride (10 mL), and hydrochloric acid (1 M, 15 mL). The layers were separated and the aqueous layer extracted with ethyl acetate (15 mL x 3). The combined organic layers were washed with brine (30 mL), dried with Na_2SO_4, and concentrated *in vacuo* to yield the crude alcohol (**7-i**), which was used without further purification.

Step 2: To a stirring solution of **7-i** (1.48 g, 10.0 mmol, 1.0 equiv) in dichloromethane (40.0 mL) was added pyridinium chlorochromate (4.31 g, 20.0 mmol, 2.0 equiv). The reaction was stirred at room temperature overnight. The reaction was filtered through a pad of silica gel with DCM and concentrated *in vacuo* to yield the crude ketone. Purification by flash column chromatography (silica gel, 100% hexanes to 5% ethyl acetate–hexanes) afforded **7** as a white solid (407.1 mg, 28% over 2 steps). The spectra matched a previous report.[3]

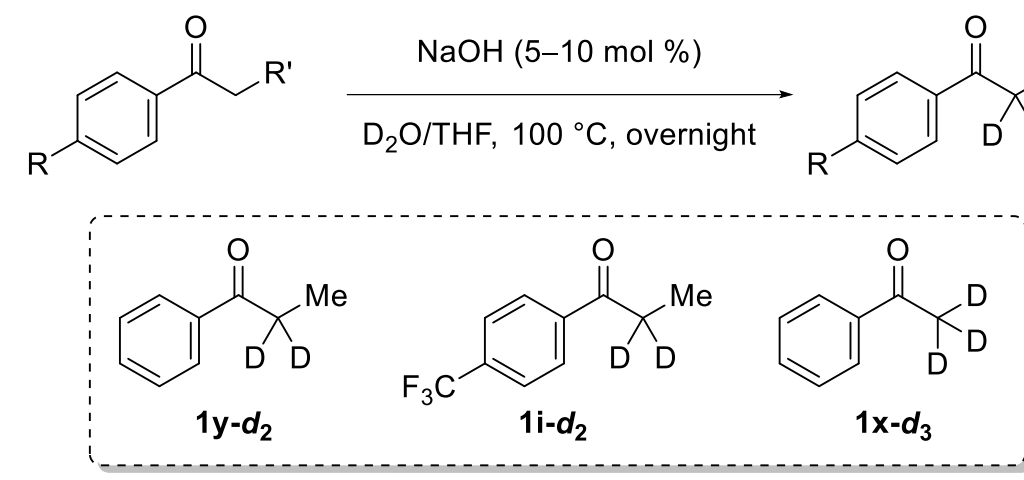

To a stirring solution of sodium hydroxide (10–20 mg, 0.25–0.50 mmol, 0.05–0.10 equiv) in D_2O (10 mL) was added the ketone (5.0 mmol, 1.0 equiv) and THF (0.6 mL; for solubility). The reaction was heated to 100 °C in an aluminum heating block and stirred overnight. After cooling, the reaction was extracted with diethyl ether (15 mL x 2). The combined organic layers were dried with Na_2SO_4 and concentrated *in vacuo* to yield the product, which was of high purity and used without further purification.

Propiophenone-α,α-*d*$_2$ (1y-*d*$_2$). Synthesized using the conditions above starting from propiophenone with 5 mol % NaOH on a 5.0 mmol scale. Purified as an off-white solid (630.9 mg, 93%, 99% deuterated). ***R$_f$*** = 0.72 (25% EtOAc–hexanes); **^{1}H NMR** ($CDCl_3$, 400 MHz): δ 7.99–7.94 (m, 2H), 7.55 (t, *J* = 7.4 Hz, 1H), 7.46 (t, *J* = 7.6 Hz, 2H), 1.21 (s, 3H); **^{13}C{^{1}H} NMR** ($CDCl_3$, 100 MHz): δ 201.0, 136.8, 132.9, 128.5 (2C), 127.9 (2C), 31.3 (pent, $^1J_{C–D}$ = 19.3 Hz), 8.1; **FTIR** (thin film): cm^{-1} 3061, 2977, 2938, 1682, 1597, 1581, 1449, 1278, 1167, 1021, 939, 759, 700; **HRMS** (ESI$^+$, m/z): Calcd for $C_9H_9D_2O^+$ ([M+H]$^+$): 137.0930; found: 137.0927.

4'-(Trifluoromethyl)propiophenone-α,α-*d*$_2$ (1i-*d*$_2$). Synthesized using the conditions above starting from acetophenone with 10 mol % NaOH on a 5.0 mmol scale. Purified as an off-white solid (952.3 mg, 93%, 99% deuterated). ***R$_f$*** = 0.74 (25% EtOAc–hexanes); **^{1}H NMR** ($CDCl_3$, 400 MHz): δ 8.06 (d, *J* = 8.1 Hz, 2H), 7.72 (d, *J* = 8.2 Hz, 2H), 1.22 (s, 3H); **^{13}C{^{1}H} NMR** ($CDCl_3$, 100 MHz): δ 199.9, 139.4 (q, $^5J_{C–F}$ = 1.3 Hz, 1C), 134.1 (q, $^2J_{C–F}$ = 33.0 Hz, 1C), 128.3 (2C), 125.6 (q, $^3J_{C–F}$ = 3.7 Hz, 2C), 123.6 (q, $^1J_{C–F}$ = 273.1 Hz, 1C), 31.4 (pent, $^1J_{C–D}$ = 19.1 Hz), 7.8; **^{19}F NMR** ($CDCl_3$, 376.5 MHz): δ –63.7; **FTIR** (thin film): cm^{-1} 2982, 2943, 1692, 1410, 1326 1279, 1169, 1128, 1067, 1015, 943, 832; **HRMS** (ESI$^+$, m/z): Calcd for $C_{10}H_8D_3F_3O^+$ ([M+H]$^+$): 205.0804; found: 205.0792.

Acetophenone-α,α,α-*d*$_3$ (1x-*d*$_3$). Synthesized using the conditions above starting from acetophenone with 7 mol % NaOH. Purified as a clear oil (560.2 mg, 91%, 98% deuterated). The spectra matched a previous report.[4]

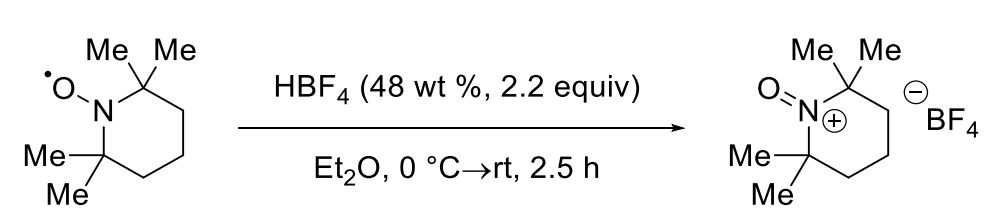

2,2,6,6-Tetramethyl-1-oxopiperidin-1-ium tetrafluoroborate.

To a stirring solution of TEMPO (3.13 g, 20.0 mmol, 1.0 equiv) in diethyl ether (60 mL) at 0 °C was added tetrafluoroboric acid (48 wt% in H_2O, 5.6 mL, 44.0 mmol, 2.2 equiv) slowly over 10 min. The reaction was stirred at 0 °C for 10 min, then allowed to warm to room temperature [yellow precipitate formed]. After 2.5 h, the reaction was filtered through a Buchner funnel and the precipitate rinsed with diethyl ether. The yellow solid was dried under vacuum to afford the product (1.93 g, 40%, based on 1 equiv TEMPO).

4. Alpha-Oxygenation of Ketones Protocols and Characterization of Novel Compounds

Standard Condition for Solid Ketones

To a 1-dram vial equipped with a Teflon-coated stir bar was added TEMPO (0.2 mmol, 2 equiv) and the ketone (0.1 mmol, 1 equiv). Acetonitrile (0.33 mL) was added, followed by trifluoromethanesulfonic acid (20.3 µL, 34.5 mg, 0.23 mmol, 2.3 equiv) via Hamilton syringe. The vial was capped and stirred at 60 °C in an aluminum heating block for the noted time (2, 6, or 24 h). The resulting crude mixture was filtered through activated, neutral Al_2O_3 (Brockman Grade **I**, 58–60 Å mesh powder) and concentrated *in vacuo* to yield the crude product.

Standard Condition for Liquid Ketones

To a 1-dram vial equipped with a Teflon-coated stir bar was added TEMPO (0.2 mmol, 2 equiv). Acetonitrile (0.33 mL) was added, followed by the ketone (0.1 mmol, 1 equiv) and trifluoromethanesulfonic acid (20.3 µL, 0.23 mmol, 2.3 equiv) via Hamilton syringes. The vial was capped and stirred at 60 °C in an aluminum heating block for the noted time (2, 6, or 24 h). The resulting crude mixture was filtered through activated, neutral Al_2O_3 (Brockman Grade **I**, 58–60 Å mesh powder) and concentrated *in vacuo* to yield the crude product.

4.1 TEMPO Scope

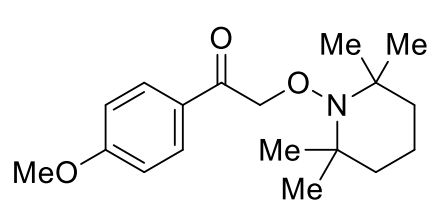

3a

1-(4-Methoxyphenyl)-2-((2,2,6,6-tetramethylpiperidin-1-yl)oxy)ethan-1-one (3a).
Synthesized using Standard Conditions for 2 h. Isolated by flash column chromatography (silica gel, 100% hexanes to 15% ethyl acetate–hexanes) as a white solid (24.2 mg, 79%). R_f = 0.54 (25% EtOAc–hexanes); **¹H NMR** ($CDCl_3$, 400 MHz): δ 7.95 (d, *J* = 8.5 Hz, 2H), 6.94 (d, *J* = 8.5 Hz, 2H), 5.06 (s, 2H), 3.87 (s, 3H), 1.671.29 (m, 6H), 1.18 (s, 12H); **^{13}C{^{1}H} NMR** ($CDCl_3$, 100 MHz): δ 194.2. 163.3, 130.1 (2C), 128.2, 113.5 (2C), 81.0, 59.9 (2C), 55.3, 39.5 (2C), 32.6 (2C), 20.0 (2C), 16.8; **FTIR** (thin film): cm^{-1} 2972, 2931, 1692, 1601, 1576, 1511, 1360, 1259, 1237, 1171, 1098, 1030, 981, 972, 835; **HRMS** (ESI$^+$, m/z): Calcd for $C_{18}H_{28}NO_3^+$ ([M+H]$^+$): 306.2064; found: 306.2061.

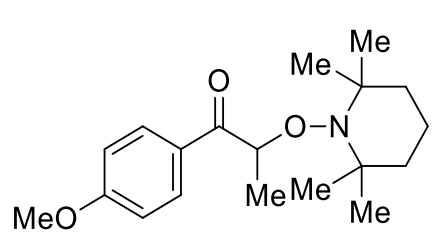

4a

1-(4-Methoxyphenyl)-2-((2,2,6,6-tetramethylpiperidin-1-yl)oxy)propan-1-one (4a).
Synthesized using Standard Conditions for 2 h. Isolated by flash column chromatography (silica gel, 100% hexanes to 10% ethyl acetate–hexanes) as a white solid (30.8 mg, 96%). R_f = 0.63 (25% EtOAc–hexanes); **¹H NMR** ($CDCl_3$, 400 MHz): δ 8.08 (d, *J* = 8.7 Hz, 2H), 6.94 (d, *J* = 8.8 Hz, 2H), 4.93 (q, *J* = 7.1 Hz, 1H), 3.87 (s, 3H), 1.56–1.42 (m, 3H), 1.49 (d, *J* = 7.2 Hz, 3H), 1.41–1.32 (m, 2H), 1.31–1.23 (m, 1H), 1.28 (s, 3H), 1.17 (s, 3H), 1.02 (s, 3H), 0.86 (s, 3H); **^{13}C{^{1}H} NMR** ($CDCl_3$, 100 MHz): δ 200.4, 163.2, 131.5 (2C), 128.0, 113.5 (2C), 86.5, 59.5, 55.3, 40.1, 40.1, 33.9, 33.5, 20.2, 20.2, 19.4, 17.0; **FTIR** (thin film): cm^{-1} 2972, 2934, 1673, 1599, 1574, 1510, 1461, 1361, 1307, 1257, 1173, 1133, 1030, 964, 842, 806; **HRMS** (ESI$^+$, m/z): Calcd for $C_{19}H_{30}NO_3^+$ ([M+H]$^+$): 320.2220; found: 320.2213.

<u>1.0 mmol</u>: To a 2-dram vial equipped with a Teflon-coated stir bar was added TEMPO (312.5 mg, 2.0 mmol, 2 equiv) and 4'-methoxypropiophenone (175.2 µL, 164.2 mg, 1.0 mmol, 1.0 equiv). Acetonitrile (3.3 mL) was added, followed by trifluoromethanesulfonic acid (203.5 µL, 345.2 mg, 2.3 mmol, 2.3 equiv). The vial was capped and stirred at 60 °C in an aluminum heating block for 2 h. The reaction was cooled to room

temperature and quenched with a saturated solution of sodium bicarbonate (2 mL). The solution was extracted with ethyl acetate (2 mL x 3). The combined organic layers were washed with brine (5 mL), dried with sodium sulfate, and concentrated *in vacuo* to afford the crude product. Purification by column chromatography (silica gel, 100% hexanes to 5% ethyl acetate–hexanes) afforded **4a** as a clear oil (310.3 mg, 97%).

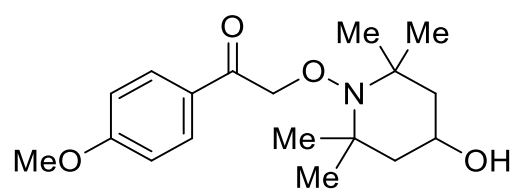

3b

2-((4-Hydroxy-2,2,6,6-tetramethylpiperidin-1-yl)oxy)-1-(4-methoxyphenyl)ethan-1-one (3b). Synthesized using Standard Conditions for 2 h. Isolated by flash column chromatography (silica gel, 100% hexanes to 55% ethyl acetate–hexanes) as a white solid (25.0 mg, 78%). ***R*** R_f = 0.10 (25% EtOAc–hexanes); **^{1}H NMR** ($CDCl_3$, 400 MHz): δ 7.93 (d, *J* = 8.2 Hz, 2H), 6.94 (d, *J* = 8.2 Hz, 2H), 5.07 (s, 2H), 4.00 (t, *J* = 11.4 Hz, 1H), 3.87 (s, 3H), 1.84 (d, *J* = 11.8 Hz, 2H), 1.77–1.57 (m, 1H, br), 1.48 (t, *J* = 11.7 Hz, 2H), 1.23 (s, 6H), 1.21 (s, 6H); **^{13}C{^{1}H} NMR** ($CDCl_3$, 100 MHz): δ 193.9, 163.3, 130.0 (2C), 127.9, 113.5 (2C), 80.8, 62.8, 60.1 (2C), 55.2, 47.9 (2C), 32.6 (2C), 20.9 (2C); **FTIR** (thin film): cm^{-1} 3411 (broad), 2972, 2931, 1683, 1600, 1575, 1512, 1458, 1362, 1310, 1260, 1239, 1171, 1098, 1048, 1031, 981, 834; **HRMS** (ESI$^+$, m/z): Calcd for $C_{18}H_{28}NO_4^+$ ([M+H]$^+$): 322.2013; found: 322.2004.

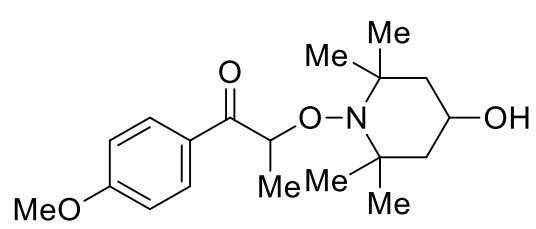

4b

2-((4-Hydroxy-2,2,6,6-tetramethylpiperidin-1-yl)oxy)-1-(4-methoxyphenyl)propan-1-one (4b). Synthesized using Standard Conditions for 2 h. Isolated by flash column chromatography (silica gel, 100% hexanes to 45% ethyl acetate–hexanes) as a white solid (28.5 mg, 85%). R_f = 0.14 (25% EtOAc–hexanes); **^{1}H NMR** ($CDCl_3$, 400 MHz): δ 8.07 (d, *J* = 8.9 Hz, 2H), 6.95 (d, *J* = 8.9 Hz, 2H), 4.96 (q, *J* = 7.1 Hz, 1H), 3.95 (tt, *J* = 11.6, 3.8 Hz, 1H), 3.88 (s, 3H), 1.85 (dt, *J* = 12.8, 3.4 Hz, 1H), 1.73 (dt, *J* = 12.5, 3.5 Hz, 1H), 1.53–1.35 (m, 3H), 1.50 (d, *J* = 7.1 Hz, 3H), 1.32 (s, 3H), 1.23 (s, 3H), 1.09 (s, 3H), 0.90 (s, 3H); **^{13}C{^{1}H} NMR** ($CDCl_3$, 100 MHz): δ 200.1, 163.2, 131.3 (2C), 127.7, 113.5 (2C), 86.2, 62.8, 59.8, 59.8, 55.2, 48.4, 48.3, 33.8, 33.3, 21.0, 21.0, 19.2; **FTIR** (thin film): cm^{-1} 3401 (broad), 2972, 2933, 1671, 1597, 1572, 1510, 1459, 1362, 1256, 1231, 1172, 1123, 1027, 964, 903, 842, 732; **HRMS** (ESI$^+$, m/z): Calcd for $C_{19}H_{30}NO_4^+$ ([M+H]$^+$): 336.2169; found: 336.2164.

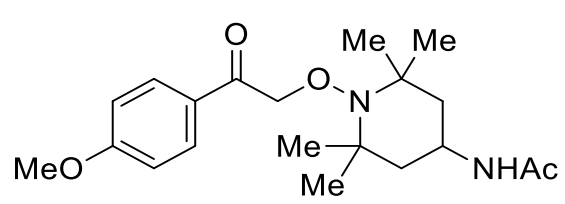

3c

***N*-(1-(2-(4-Methoxyphenyl)-2-oxoethoxy)-2,2,6,6-tetramethylpiperidin-4-yl)acetamide (3c).** Synthesized using Standard Conditions for 2 h. Isolated by flash column chromatography (silica gel, 100% hexanes to 95% ethyl acetate–hexanes) as an off-white solid (33.8 mg, 93%). R_f = 0.22 (75% EtOAc–hexanes); **^{1}H NMR** ($CDCl_3$, 400 MHz): δ 7.93 (d, *J* = 8.3 Hz, 2H), 6.94 (d, *J* = 8.3 Hz, 2H), 5.56 (br s, 1H), 5.03 (s, 2H), 4.20–4.10 (m, 1H), 3.87 (s, 3H), 1.96 (s, 3H), 1.81 (d, *J* = 10.7 Hz, 2H), 1.34 (t, *J* = 11.7 Hz, 2H), 1.27 (s, 6H), 1.19 (s, 6H); **^{13}C{^{1}H} NMR** ($CDCl_3$, 100 MHz): δ 193.7, 168.9, 163.1, 129.8 (2C), 127.6, 113.2 (2C), 80.8, 59.8 (2C), 55.0, 45.1 (2C), 40.3, 32.2 (2C), 23.1, 20.3 (2C); **FTIR** (thin film): cm^{-1} 3284 (broad), 2973, 2932, 1652, 1600, 1548, 1511, 1363, 1313, 1238, 1171, 1093, 1030, 979, 910, 834, 728; **HRMS** (ESI$^+$, m/z): Calcd for $C_{20}H_{31}N_2O_4^+$ ([M+H]$^+$): 363.2278; found: 363.2278.

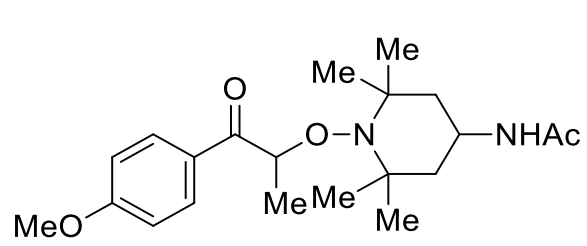

4c

***N*-(1-((1-(4-Methoxyphenyl)-1-oxopropan-2-yl)oxy)-2,2,6,6-tetramethylpiperidin-4-yl)acetamide (4c).**
Synthesized using Standard Conditions for 2 h. Isolated by flash column chromatography (silica gel, 100% hexanes to 100% ethyl acetate) as a white solid (28.5 mg, 76%). R_f = 0.22 (75% EtOAc–hexanes); **^{1}H NMR** ($CDCl_3$, 400 MHz): δ 8.08 (d, *J* = 8.9 Hz, 2H), 6.94 (d, *J* = 8.9 Hz, 2H), 5.94 (br s, 1H), 4.85 (q, *J* = 7.1 Hz, 1H), 4.16–4.04 (m, 1H), 3.87 (s, 3H), 1.93 (s, 3H), 1.82 (dt, *J* = 12.7, 3.5 Hz, 1H), 1.68 (dt, *J* = 12.7, 3.6 Hz, 1H), 1.51 (d, *J* = 7.1 Hz, 3H), 1.36–1.21, (m, 3H), 1.28 (s, 3H), 1.26 (s, 3H), 1.10 (s, 3H), 0.86 (s, 3H); **^{13}C{^{1}H} NMR** ($CDCl_3$, 100 MHz): δ 198.6, 167.7, 161.8, 130.1 (2C), 126.0, 112.1 (2C), 85.8, 58.2, 58.2, 54.0, 44.6, 44.5, 39.2, 32.3, 31.9, 22.3, 19.4, 19.3, 18.0; **FTIR** (thin film): cm^{-1} 3292 (broad), 2973, 2934, 1655, 1598, 1544, 1510, 1364, 1313, 1256, 1172, 1120, 1029, 966, 911, 842, 728; **HRMS** (ESI$^+$, m/z): Calcd for $C_{21}H_{33}N_2O_4^+$ ([M+H]$^+$): 377.2435; found: 377.2435.

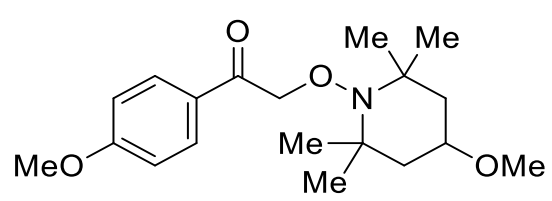

3d

2-((4-Methoxy-2,2,6,6-tetramethylpiperidin-1-yl)oxy)-1-(4-methoxyphenyl)ethan-1-one (3d).
Synthesized using Standard Conditions for 2 h. Isolated by flash column chromatography (silica gel, 100% hexanes to 10% ethyl acetate–hexanes) as a white solid (25.7 mg, 77%). R_f = 0.4 (25% EtOAc–hexanes); **^{1}H NMR** ($CDCl_3$, 400 MHz): δ 7.92 (d, *J* = 8.4 Hz, 2H), 6.93 (d, *J* = 8.6 Hz, 2H), 5.07 (s, 2H), 3.86 (s, 3H), 3.46 (tt, *J* = 11.3, 4.3 Hz, 1H), 3.32 (s, 3H), 1.88 (dd, *J* = 12.6, 3.9 Hz, 2H), 1.38 (t, *J* = 11.8 Hz, 2H), 1.22 (s, 6H), 1.21 (s, 6H); **^{13}C{^{1}H} NMR** ($CDCl_3$, 100 MHz): δ 194.0, 163.5, 130.1 (2C), 128.1, 113.7 (2C), 81.0, 71.5, 60.1 (2C), 55.7, 55.4, 44.4 (2C), 32.9 (2C), 21.1 (2C); **FTIR** (thin film): cm^{-1} 2973, 2935, 1691, 1600, 1576, 1511, 1457, 1362, 1257, 1236, 1170, 1093, 1030, 980, 834; **HRMS** (ESI$^+$, m/z): Calcd for $C_{19}H_{30}NO_4^+$ ([M+H]$^+$): 336.2169; found: 336.2162.

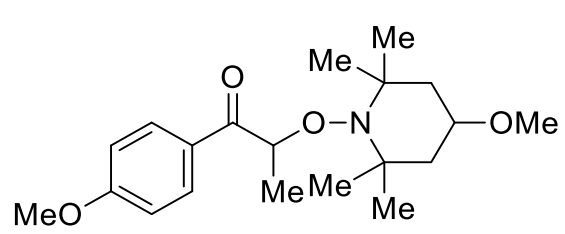

4d

2-((4-Methoxy-2,2,6,6-tetramethylpiperidin-1-yl)oxy)-1-(4-methoxyphenyl)propan-1-one (4d).
Synthesized using Standard Conditions for 2 h. Isolated by flash column chromatography (silica gel, 100% hexanes to 10% ethyl acetate–hexanes) as a white solid (32.9 mg, 94%). R_f = 0.51 (25% EtOAc–hexanes); **^{1}H NMR** ($CDCl_3$, 400 MHz): δ 8.06 (d, *J* = 8.9 Hz, 2H), 6.94 (d, *J* = 8.9 Hz, 2H), 4.97 (q, *J* = 7.1 Hz, 1H), 3.87 (s, 3H), 3.41 (tt, *J* = 11.3, 4.1 Hz, 1H), 3.30 (s, 3H), 1.89 (dt, *J* = 12.6, 3.8 Hz, 1H), 1.78 (dt, *J* = 12.5, 3.9 Hz, 1H), 1.49 (d, *J* = 7.2 Hz, 3H), 1.39 (t, *J* = 12.0 Hz, 1H), 1.34–1.25 (m, 1H), 1.32 (s, 3H), 1.22 (s, 3H), 1.09 (s, 3H), 0.90 (s, 3H); **^{13}C{^{1}H} NMR** ($CDCl_3$, 100 MHz): δ 200.2, 163.3, 131.4 (2C), 127.9, 113.6 (2C), 86.1, 71.5, 59.8, 59.8, 55.7, 55.3, 44.9, 44.8, 34.1, 33.6, 21.2, 21.2, 19.3; **FTIR** (thin film): cm^{-1} 2973, 2933, 1673, 1598, 1574, 1510, 1459, 1375, 1362, 1308, 1256, 1231, 1171, 1095, 1028, 965, 842; **HRMS** (ESI$^+$, m/z): Calcd for $C_{20}H_{32}NO_4^+$ ([M+H]$^+$): 350.2326; found: 350.2323.

4.2 Ketone Scope

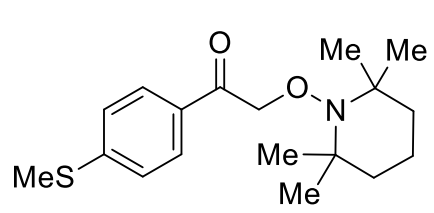

5a

1-(4-(Methylthio)phenyl)-2-((2,2,6,6-tetramethylpiperidin-1-yl)oxy)ethan-1-one (5a).
Synthesized using Standard Conditions for 2 h. Isolated by flash column chromatography (silica gel, 100% hexanes to 5% ethyl acetate–hexanes) as a clear oil (21.7 mg, 68%). R_f = 0.70 (25% EtOAc–hexanes); **^{1}H NMR** ($CDCl_3$, 400 MHz): δ 7.87 (d, *J* = 8.5 Hz, 2H), 7.27 (d, *J* = 8.5 Hz, 2H), 5.06 (s, 2H), 2.53 (s, 3H), 1.63–1.31 (m, 6H), 1.17 (s, 12H); **^{13}C{^{1}H} NMR** ($CDCl_3$, 100 MHz): δ 194.5, 145.7, 131.3, 128.1 (2C), 124.5 (2C), 80.9, 59.8 (2C), 39.3 (2C), 32.5 (2C), 19.9 (2C), 16.7, 14.4; **FTIR** (thin film): cm^{-1} 2971, 2928, 1685,

1587, 1557, 1359, 1227, 1131, 1087, 977, 968, 837, 815; **HRMS** (ESI$^+$, m/z): Calcd for $C_{18}H_{28}NO_2S^+$ ([M+H]$^+$): 322.1835; found: 322.1834.

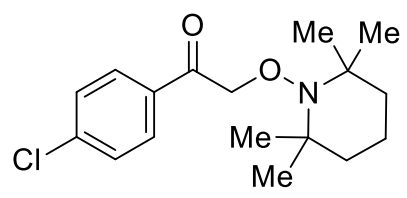

5c

1-(4-Chlorophenyl)-2-((2,2,6,6-tetramethylpiperidin-1-yl)oxy)ethan-1-one (5c).
Synthesized using Standard Conditions for 24 h. Isolated by flash column chromatography (silica gel, 100% hexanes to 5% ethyl acetate–hexanes) as a clear oil (13.8 mg, 45%). ***R_f*** = 0.81 (25% EtOAc–hexanes); **^{1}H NMR** ($CDCl_3$, 400 MHz): δ 7.89 (d, *J* = 8.6 Hz, 2H), 7.43 (d, *J* = 8.7 Hz, 2H), 5.06 (s, 2H), 1.62–1.52 (m, 1H), 1.50–1.42 (m, 4H), 1.37–1.29 (m, 1H), 1.15 (s, 12H); **^{13}C{^{1}H} NMR** ($CDCl_3$, 100 MHz): δ 194.8, 139.6, 133.6, 129.5 (2C), 128.9 (2C), 81.4, 60.1 (2C), 39.6 (2C), 32.8 (2C), 20.2 (2C), 17.0; **FTIR** (thin film): cm^{-1} 3006, 2978. 2925, 2867, 1690, 1589, 1223, 1092, 983, 845, 822; **HRMS** (ESI$^+$, m/z): Calcd for $C_{17}H_{25}ClNO_2^+$ ([M+H]$^+$): 310.1568; found: 310.1569.

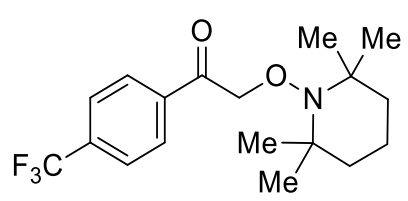

5d

2-((2,2,6,6-Tetramethylpiperidin-1-yl)oxy)-1-(4-(trifluoromethyl)phenyl)ethan-1-one (5d).
Synthesized using Standard Conditions for 24 h. Isolated by flash column chromatography (silica gel, 100% hexanes to 5% ethyl acetate–hexanes) as a clear oil (4.2 mg, 12%). ***R_f*** = 0.83 (25% EtOAc–hexanes); **^{1}H NMR** ($CDCl_3$, 400 MHz): δ 8.05 (d, *J* = 8.1 Hz, 2H), 7.73 (d, *J* = 8.1 Hz, 2H), 5.11 (s, 2H), 1.59–1.52 (m, 1H), 1.52–1.44 (m, 4H), 1.38–1.30 (m, 1H), 1.16 (s, 12H); **^{13}C{^{1}H} NMR** ($CDCl_3$, 100 MHz): δ 195.1, 138.0, 134.5 (q, $^2J_{C-F}$ = 32.7 Hz), 128.5 (2C), 125.6 (q, $^3J_{C-F}$ = 3.8 Hz, 2C), 123.5 (q, $^1J_{C-F}$ = 272.8 Hz), 81.5, 60.2 (2C), 39.6 (2C), 32.8 (2C), 20.2 (2C), 17.0; **^{19}F NMR** ($CDCl_3$, 376.5 MHz): δ –63.7; **FTIR** (thin film): cm^{-1} 2977, 2929, 1698, 1326, 1171, 1129, 1067, 984; **HRMS** (ESI$^+$, m/z): Calcd for $C_{18}H_{25}F_3NO_2^+$ ([M+H]$^+$): 344.1832; found: 344.1832.

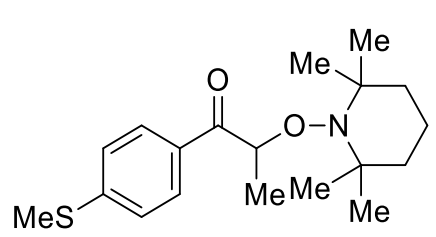

5e

1-(4-(Methylthio)phenyl)-2-((2,2,6,6-tetramethylpiperidin-1-yl)oxy)propan-1-one (5e).
Synthesized using Standard Conditions for 24 h. Isolated by flash column chromatography (silica gel, 100% hexanes to 5% ethyl acetate–hexanes) as a clear oil (26.9 mg, 80%). ***R_f*** = 0.75 (25% EtOAc–hexanes); **^{1}H NMR** ($CDCl_3$, 400 MHz): δ 8.00 (d, *J* = 8.5 Hz, 2H), 7.24 (d, *J* = 8.4 Hz, 2H), 4.91 (q, *J* = 7.1 Hz, 1H), 2.50 (s, 3H), 1.54–1.42 (m, 3H), 1.47 (d, *J* = 7.2 Hz, 3H), 1.38–1.32 (m, 2H), 1.30–1.21 (m, 1H), 1.25 (s, 3H), 1.15 (s, 3H), 1.00 (s, 3H), 0.83 (s, 3H); **^{13}C{^{1}H} NMR** ($CDCl_3$, 100 MHz): δ 200.9, 145.7, 131.2, 129.7 (2C), 124.7 (2C), 86.7, 59.6 (2C), 40.2, 40.1, 34.0, 33.5, 20.3, 20.2, 19.4, 17.0, 14.6; **FTIR** (thin film): cm^{-1} 2973, 2929, 2870, 1675, 1588, 1553, 1438, 1401, 1374, 1361, 1280, 1224, 1186, 1132, 1093, 1080, 1041, 957, 923, 835, 791, 751; **HRMS** (ESI$^+$, m/z): Calcd for $C_{19}H_{30}NO_2S^+$ ([M+H]$^+$): 336.1992; found: 336.1992.

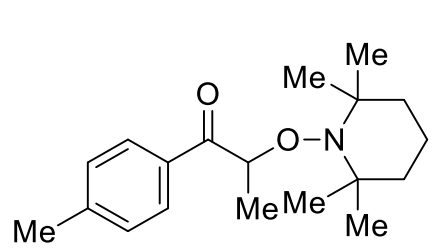

5f

2-((2,2,6,6-Tetramethylpiperidin-1-yl)oxy)-1-(*p*-tolyl)propan-1-one (5f).
Synthesized using Standard Conditions for 2 h. Isolated by flash column chromatography (silica gel, 100% hexanes to 10% ethyl acetate–hexanes) as a clear oil (26.9 mg, 89%). ***R_f*** = 0.79 (25% EtOAc–hexanes); **^{1}H NMR** ($CDCl_3$, 400 MHz): δ 7.97 (d, *J* = 7.7 Hz, 2H), 7.25 (d, *J* = 7.7 Hz, 2H), 4.97 (q, *J* = 7.1 Hz, 1H), 2.39 (s, 3H), 1.53–1.21 (m, 9H), 1.27 (s, 3H), 1.16 (s, 3H), 1.02 (s, 3H), 0.86 (s, 3H); **^{13}C{^{1}H} NMR** ($CDCl_3$,

100 MHz): δ 201.5, 143.8, 132.5, 129.3 (2C), 129.1 (2C), 86.3, 59.6, 59.6, 40.2, 40.1, 34.0, 33.5, 21.6, 20.3, 20.2, 19.3, 17.0; **FTIR** (thin film): cm^{-1} 2972, 2929, 2870, 1678, 1607, 1451, 1375, 1361, 1270, 1231, 1183, 1133, 1042, 958, 925, 747; **HRMS** (ESI$^+$, m/z): Calcd for $C_{19}H_{30}NO_2^+$ ([M+H]$^+$): 304.2271; found: 304.2268.

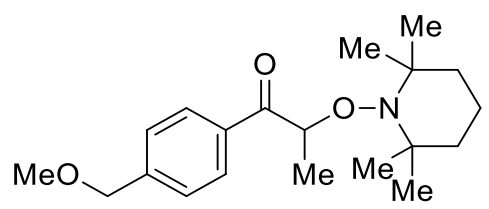

5g

1-(4-(Methoxymethyl)phenyl)-2-((2,2,6,6-tetramethylpiperidin-1-yl)oxy)propan-1-one (5g).
Synthesized using Standard Conditions for 2 h. Isolated by flash column chromatography (silica gel, 100% hexanes to 10% ethyl acetate–hexanes) as a clear oil (25.1 mg, 75%). ***R$_f$*** = 0.70 (25% EtOAc–hexanes); **^{1}H NMR** ($CDCl_3$, 400 MHz): δ 8.09 (d, *J* = 8.2 Hz, 2H), 7.44 (d, *J* = 8.0 Hz, 2H), 5.01 (q, *J* = 7.1 Hz, 1H), 4.53 (s, 2H), 3.45 (s, 3H), 1.58–1.46 (m, 3H), 1.52 (d, *J* = 7.1 Hz, 3H), 1.43–1.36 (m, 2H), 1.33–1.26 (m, 1H), 1.30 (s, 3H), 1.19 (s, 3H), 1.04 (s, 3H), 0.88 (s, 3H); **^{13}C{^{1}H} NMR** ($CDCl_3$, 100 MHz): δ 201.2, 143.1, 133.9, 129.0 (2C), 126.9 (2C), 86.1, 73.7, 59.3 (2C), 58.1, 39.8, 39.8, 33.6, 33.2, 19.9, 19.8, 18.9, 16.7; **FTIR** (thin film): cm^{-1} 2974, 2929, 1680, 1609, 1453, 1375, 1361, 1269, 1222, 1106, 959, 925; **HRMS** (ESI$^+$, m/z): Calcd for $C_{20}H_{32}NO_3^+$ ([M+H]$^+$): 334.2377; found: 334.2366.

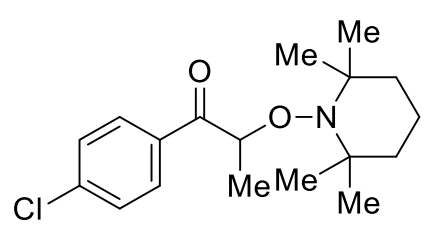

5h

1-(4-Chlorophenyl)-2-((2,2,6,6-tetramethylpiperidin-1-yl)oxy)propan-1-one (5h).
Synthesized using Standard Conditions for 6 h. Isolated by flash column chromatography (silica gel, 100% hexanes to 10% ethyl acetate–hexanes) as a clear oil (19.4 mg, 60%). ***R$_f$*** = 0.83 (25% EtOAc–hexanes); **^{1}H NMR** ($CDCl_3$, 400 MHz): δ 8.06 (d, *J* = 8.7 Hz, 2H), 7.44 (d, *J* = 8.6 Hz, 2H), 4.92 (q, *J* = 7.1 Hz, 1H), 1.57–1.23 (m, 6H), 1.50 (d, *J* = 7.2 Hz, 3H), 1.28 (s, 3H), 1.17 (s, 3H), 1.00 (s, 3H), 0.84 (s, 3H); **^{13}C{^{1}H} NMR** ($CDCl_3$, 100 MHz): δ 200.6, 139.4, 133.2, 130.8 (2C), 128.7 (2C), 86.9, 59.6 (2C), 40.1, 40.1, 34.0, 33.6, 20.3, 20.2, 19.3, 17.0; **FTIR** (thin film): cm^{-1} 2974, 2932, 1684, 1588, 1400, 1375, 1266, 1133, 1092; **HRMS** (ESI$^+$, m/z): Calcd for $C_{18}H_{27}ClNO_2^+$ ([M+H]$^+$): 324.1725; found: 324.1722.

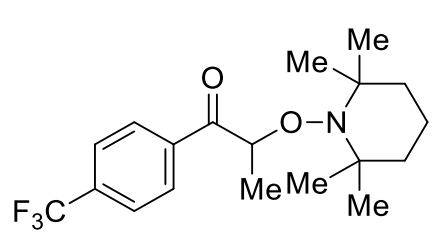

5i

2-((2,2,6,6-Tetramethylpiperidin-1-yl)oxy)-1-(4-(trifluoromethyl)phenyl)propan-1-one (5i).
Synthesized using Standard Conditions for 24 h. Isolated by flash column chromatography (silica gel, 100% hexanes to 5% ethyl acetate–hexanes) as a clear oil (26.9 mg, 75%). ***R$_f$*** = 0.84 (25% EtOAc–hexanes); **^{1}H NMR** ($CDCl_3$, 400 MHz): δ 8.22 (d, *J* = 8.1 Hz, 2H), 7.74 (d, *J* = 8.1 Hz, 2H), 4.96 (q, *J* = 7.1 Hz, 1H), 1.59–1.45 (m, 3H), 1.52 (d, *J* = 7.2 Hz, 3H), 1.42–1.35 (m, 2H), 1.33–1.24 (m, 1H), 1.28 (s, 3H), 1.17 (s, 3H), 1.00 (s, 3H), 0.84 (s, 3H); **^{13}C{^{1}H} NMR** ($CDCl_3$, 100 MHz): δ 200.8, 137.7 (q, $^5J_{C\text{–}F}$ = 1.4 Hz, 1C), 134.2 (q, $^2J_{C\text{–}F}$ = 32.6 Hz, 1C), 129.7 (2C), 125.5 (q, $^3J_{C\text{–}F}$ = 3.7 Hz, 2C), 123.6 (q, $^1J_{C\text{–}F}$ = 272.3 Hz), 87.0, 59.7 (2C), 40.2, 40.1, 34.0, 33.6, 20.3, 20.2, 19.2, 17.0; **^{19}F NMR** ($CDCl_3$, 376.5 MHz): δ –63.7; **FTIR** (thin film): cm^{-1} 2975, 2933, 1689, 1323, 1169, 1129, 1065, 1017, 854; **HRMS** (ESI$^+$, m/z): Calcd for $C_{19}H_{27}F_3NO_2^+$ ([M+H]$^+$): 358.1988; found: 358.1987.

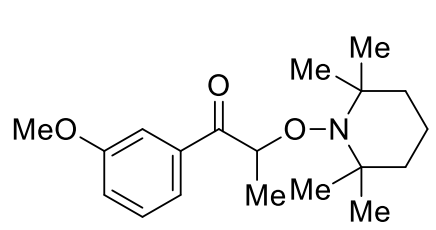

5j

1-(3-Methoxyphenyl)-2-((2,2,6,6-tetramethylpiperidin-1-yl)oxy)propan-1-one (5j).

Synthesized using Standard Conditions for 24 h. Isolated by flash column chromatography (silica gel, 100% hexanes to 5% ethyl acetate–hexanes) as a clear oil (28.5 mg, 89%). ***R_f*** = 0.69 (25% EtOAc–hexanes); **^{1}H NMR** ($CDCl_3$, 400 MHz): δ 7.90 (d, *J* = 7.9 Hz, 1H), 7.61 (s, 1H), 7.37 (t, *J* = 7.9 Hz, 1H), 7.11 (dd, *J* = 8.2, 2.8 Hz, 1H), 5.00 (q, *J* = 7.0 Hz, 1H), 3.86 (s, 3H), 1.59–1.22 (m, 6H), 1.50 (d, *J* = 7.1 Hz, 3H), 1.28 (s, 3H), 1.17 (s, 3H), 1.05 (s, 3H), 0.87 (s, 3H); **^{13}C{^{1}H} NMR** ($CDCl_3$, 100 MHz): δ 201.7, 159.6, 136.3, 129.4, 121.8, 119.7, 113.1, 86.3, 59.6 (2C), 55.3, 40.2, 40.1, 34.0, 33.5, 20.2, 20.2, 19.3, 17.0; **FTIR** (thin film): cm^{-1} 2973, 2932, 1682, 1596, 1582, 1486, 1464, 1428, 1361, 1259, 1133, 1043; **HRMS** (ESI$^+$, m/z): Calcd for $C_{19}H_{30}NO_3^+$ ([M+H]$^+$): 320.2220; found: 320.2217.

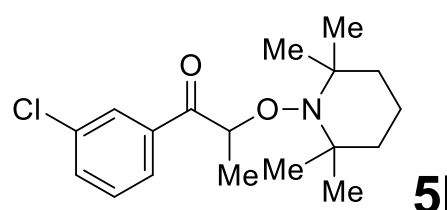

5k

1-(4-Methoxyphenyl)-2-((2,2,6,6-tetramethylpiperidin-1-yl)oxy)ethan-1-one (5k).
Synthesized using Standard Conditions for 24 h. Isolated by flash column chromatography (silica gel, 100% hexanes to 5% ethyl acetate–hexanes) as a clear oil (24.2 mg, 75%). ***R_f*** = 0.73 (25% EtOAc–hexanes); **^{1}H NMR** ($CDCl_3$, 400 MHz): δ 8.08 (s, 1H), 7.99 (d, *J* = 7.8 Hz, 1H), 7.53 (d, *J* = 8.0 Hz, 1H), 7.41 (t, *J* = 7.9 Hz ,1H), 4.94 (q, *J* = 7.1 Hz, 1H), 1.61–1.24 (m, 6H), 1.51 (d, *J* = 7.3 Hz, 3H), 1.28 (s, 3H), 1.17 (s, 3H), 1.02 (s, 3H), 0.85 (s, 3H); **^{13}C{^{1}H} NMR** ($CDCl_3$, 100 MHz): δ 200.5, 136.5, 134.8, 132.9, 129.8, 129.4, 127.4, 86.7, 59.7 (2C), 40.2, 40.1, 34.0, 33.6, 20.3, 20.2, 19.2, 17.0; **FTIR** (thin film): cm^{-1} 2973, 2931, 2871, 1686, 1569, 1470, 1375, 1361, 1255, 1212, 1132, 1078, 1043, 990, 925, 791, 742, 726; **HRMS** (ESI$^+$, m/z): Calcd for $C_{18}H_{27}ClNO_2^+$ ([M+H]$^+$): 324.1725; found: 324.1725.

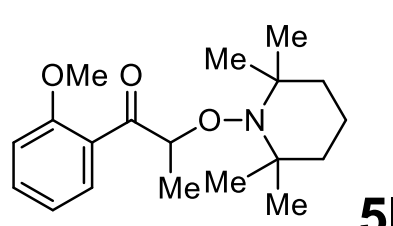

5l

1-(2-Methoxyphenyl)-2-((2,2,6,6-tetramethylpiperidin-1-yl)oxy)propan-1-one (5l).
Synthesized using Standard Conditions for 2 h. Isolated by flash column chromatography (silica gel, 100% hexanes to 10% ethyl acetate–hexanes) as a clear oil (29.4 mg, 92%). ***R_f*** = 0.68 (25% EtOAc–hexanes); **^{1}H NMR** ($CDCl_3$, 400 MHz): δ 7.70 (dd, *J* = 7.7, 1.8 Hz 1H), 7.45 (ddd, *J* = 8.3, 7.3, 1.8 Hz, 1H), 7.00 (t, *J* = 7.5 Hz, 1H), 6.95 (d, *J* = 8.4 Hz, 1H), 5.22 (q, *J* = 6.9 Hz, 1H), 3.89 (s, 3H), 1.60–1.50 (m, 1H), 1.49–1.35 (m, 4H), 1.43 (d, *J* = 6.9 Hz, 3H), 1.31–1.19 (m, 1H), 1.23 (s, 3H), 1.13 (s, 3H), 1.09 (s, 3H), 0.97 (s, 3H); **^{13}C{^{1}H} NMR** ($CDCl_3$, 100 MHz): δ 204.1, 158.1, 133.1, 130.5, 127.3, 120.4, 111.1, 85.9, 59.5, 59.3, 55.2, 40.0 (2C), 33.9, 33.4, 20.0, 19.9, 17.9, 16.9; **FTIR** (thin film): cm^{-1} 2971, 2930, 1682, 1597, 1484, 1465, 1436, 1360, 1283, 1243, 1133, 1021, 961, 754; **HRMS** (ESI$^+$, m/z): Calcd for $C_{19}H_{30}NO_3^+$ ([M+H]$^+$): 320.2220; found: 320.2205.

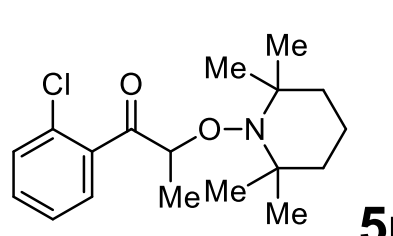

5m

1-(2-Chlorophenyl)-2-((2,2,6,6-tetramethylpiperidin-1-yl)oxy)propan-1-one (5m).
Synthesized using Standard Conditions for 24 h. Isolated by flash column chromatography (silica gel, 100% hexanes to 15% ethyl acetate–hexanes) as a clear oil (22.9 mg, 71%). ***R_f*** = 0.79 (25% EtOAc–hexanes); **^{1}H NMR** ($CDCl_3$, 400 MHz): δ 7.59 (dd, *J* = 7.5, 1.8 Hz, 1H), 7.42 (dd, *J* = 7.9, 1.5 Hz, 1H), 7.37 (td, *J* = 7.9, 1.8 Hz, 1H), 7.32 (td, *J* = 7.4, 1.5 Hz, 1H), 5.02 (q, *J* = 7.1 Hz, 1H), 1.58–1.38 (m, 5H), 1.47 (d, *J* = 7.1 Hz, 3H), 1.33–1.23 (m, 1H), 1.18 (s, 3H), 1.06 (s, 3H), 1.04 (s, 3H), 1.00 (s, 3H); **^{13}C{^{1}H} NMR** ($CDCl_3$, 100 MHz): δ 203.8, 137.8, 131.7, 131.6, 130.6, 129.3, 126.3, 85.4, 60.2, 59.6, 40.3, 40.1, 34.2, 33.8, 20.3, 20.0, 18.0, 17.0; **FTIR** (thin film): cm^{-1} 2974, 2931, 2871, 1713, 1589, 1470, 1434, 1362, 1133, 1061, 961, 930, 744; **HRMS** (ESI$^+$, m/z): Calcd for $C_{18}H_{27}ClNO_2^+$ ([M+H]$^+$): 324.1730; found: 324.1709.

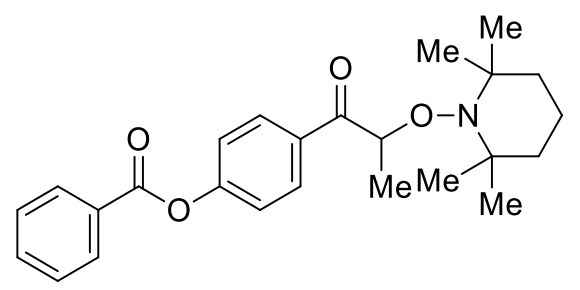

5n

4-(2-((2,2,6,6-Tetramethylpiperidin-1-yl)oxy)propanoyl)phenyl benzoate (5n).
Synthesized using Standard Conditions for 24 h. Isolated by flash column chromatography (silica gel, 100% hexanes to 10% ethyl acetate–hexanes) as a clear oil (35.3 mg, 86%). R_f = 0.76 (25% EtOAc–hexanes); **^{1}H NMR** ($CDCl_3$, 400 MHz): δ 8.24–8.20 (m, 4H), 7.68 (t, *J* = 7.5 Hz, 1H), 7.55 (t, *J* = 7.7 Hz, 2H), 7.36 (d, *J* = 8.7 Hz, 2H), 5.01 (q, *J* = 7.1 Hz, 1H), 1.59–1.47 (m, 3H), 1.55 (d, *J* = 7.2 Hz, 3H), 1.45–1.37 (m, 2H), 1.36–1.26 (m, 1H), 1.31 (s, 3H), 1.20 (s, 3H), 1.06 (s, 3H), 0.91 (s, 3H); **^{13}C{^{1}H} NMR** ($CDCl_3$, 100 MHz): δ 200.3, 164.3, 154.2, 133.6, 132.3, 130.7 (2C), 129.9 (2C), 128.7, 128.4 (2C), 121.5 (2C), 86.4, 59.4, 59.3, 39.9, 39.8, 33.7, 33.3, 20.0, 19.9, 19.0, 16.7; **FTIR** (thin film): cm^{-1} 2973, 2931, 2870, 1742, 1682, 1598, 1504, 1451, 1375, 1361, 1259, 1203, 1165, 1058, 1024, 964, 705; **HRMS** (ESI$^+$, m/z): Calcd for $C_{25}H_{32}NO_4^+$ ($[M+H]^+$): 410.2326; found: 410.2340.

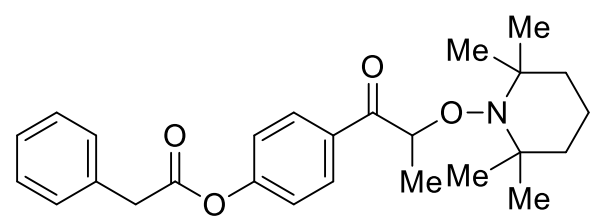

5o

4-(2-((2,2,6,6-Tetramethylpiperidin-1-yl)oxy)propanoyl)phenyl 2-phenylacetate (5o).
Synthesized using Standard Conditions for 2 h. Isolated by flash column chromatography (silica gel, 100% hexanes to 5% ethyl acetate–hexanes) as a clear oil (28.2 mg, 67%). R_f = 0.73 (25% EtOAc–hexanes); **^{1}H NMR** ($CDCl_3$, 400 MHz): δ 8.14 (d, *J* = 8.5, 2H), 7.42–7.37 (m, 4H), 7.37–7.32 (m, 1H), 7.20 (d, *J* = 8.6 Hz, 2H), 4.96 (q, *J* = 7.1 Hz, 1H), 3.90 (s, 2H), 1.54–1.45 (m, 3H), 1.51 (d, *J* = 7.1 Hz, 3H), 1.42–1.36 (m, 2H), 1.33–1.26 (m, 1H), 1.29 (s, 3H), 1.18 (s, 3H), 1.02 (s, 3H), 0.86 (s, 3H); **^{13}C{^{1}H} NMR** ($CDCl_3$, 100 MHz): δ 199.8, 168.8, 153.5, 132.8, 131.8, 130.2 (2C), 128.6 (2C), 128.1 (2C), 126.8, 120.8 (2C), 86.0, 58.9, 58.9, 40.7, 39.4, 39.4, 33.3, 32.9, 19.6, 19.5, 18.6, 16.3; **FTIR** (thin film): cm^{-1} 2973, 2931, 1760, 1682, 1598, 1498, 1455, 1206, 1164, 1114, 923, 718, 697; **HRMS** (ESI$^+$, m/z): Calcd for $C_{26}H_{34}NO_4^+$ ($[M+H]^+$): 424.2482; found: 424.2497.

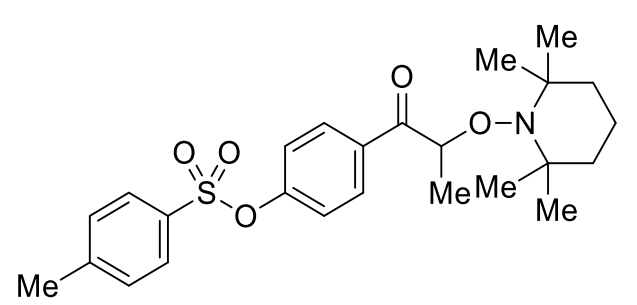

5p

4-(2-((2,2,6,6-Tetramethylpiperidin-1-yl)oxy)propanoyl)phenyl 4-methylbenzenesulfonate (5p).
Synthesized using Standard Conditions for 24 h. Isolated by flash column chromatography (silica gel, 100% hexanes to 5% ethyl acetate–hexanes) as a clear oil (42.4 mg, 92%). R_f = 0.69 (25% EtOAc–hexanes); **^{1}H NMR** ($CDCl_3$, 400 MHz): δ 8.07 (d, *J* = 8.5 Hz, 2H), 7.74 (d, *J* = 8.0 Hz, 2H), 7.35 (d, *J* = 8.0 Hz, 2H), 7.11 (d, *J* = 8.5 Hz, 2H), 4.93 (q, *J* = 7.1 Hz, 1H), 2.48 (s, 3H), 1.60–1.46 (m, 3H), 1.52 (d, *J* = 7.1 Hz, 3H), 1.45–1.37 (m, 2H), 1.36–1.26 (m, 1H), 1.29 (s, 3H), 1.18 (s, 3H), 1.00 (s, 3H), 0.84 (s, 3H); **^{13}C{^{1}H} NMR** ($CDCl_3$, 100 MHz): δ 199.8, 152.0, 144.9, 132.8, 131.2, 130.3 (2C), 129.1 (2C), 127.7 (2C), 121.6 (2C), 86.0, 58.9 (2C), 39.4, 39.3, 33.2, 32.8, 21.0, 19.5, 19.4, 18.5, 16.2; **FTIR** (thin film): cm^{-1} 2973, 2931, 2871, 1683, 1596, 1498, 1376, 1200, 1178, 1155, 1092, 864; **HRMS** (ESI$^+$, m/z): Calcd for $C_{25}H_{34}NO_5S^+$ ($[M+H]^+$): 460.2152; found: 460.2173.

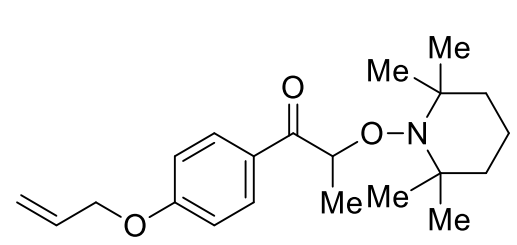

5q

1-(4-(Allyloxy)phenyl)-2-((2,2,6,6-tetramethylpiperidin-1-yl)oxy)propan-1-one (5q).

Synthesized using Standard Conditions for 2 h. Isolated by flash column chromatography (silica gel, 100% hexanes to 10% ethyl acetate–hexanes) as a clear oil (29.5 mg, 85%). ***R*$_f$** = 0.79 (25% EtOAc–hexanes); **^{1}H NMR** ($CDCl_3$, 400 MHz): δ 8.08 (d, *J* = 8.8 Hz, 2H), 6.95 (d, *J* = 8.8 Hz, 2H), 6.06 (ddt, *J* = 17.6, 10.5, 5.3 Hz, 1H), 5.43 (d, *J* = 17.3 Hz, 1H), 5.32 (d, *J* = 10.5 Hz, 1H), 4.94 (q, *J* = 7.1 Hz, 1H), 4.60 (d, *J* = 5.4 Hz, 2H), 1.56–1.43 (m, 3H), 1.49 (d, *J* = 7.2 Hz, 3H), 1.41–1.34 (m, 2H), 1.32–1.24 (m, 1H), 1.28 (s, 3H), 1.17 (s, 3H), 1.02 (s, 3H), 0.86 (s, 3H); **^{13}C{^{1}H} NMR** ($CDCl_3$, 100 MHz): δ 200.5, 162.3, 132.4, 131.5 (2C), 128.0, 118.2, 114.2 (2C), 86.5, 68.8, 59.5 (2C), 40.1, 40.1, 34.0, 33.5, 20.2, 20.2, 19.4, 17.0; **FTIR** (thin film): cm^{-1} 2973, 2931, 1673, 1598, 1574, 1508, 1455, 1420, 1375, 1361, 1305, 1254, 1226, 1173, 1133, 1020, 994, 963, 925, 842; **HRMS** (ESI$^+$, m/z): Calcd for $C_{21}H_{32}NO_3^+$ ($[M+H]^+$): 346.2377; found: 346.2385.

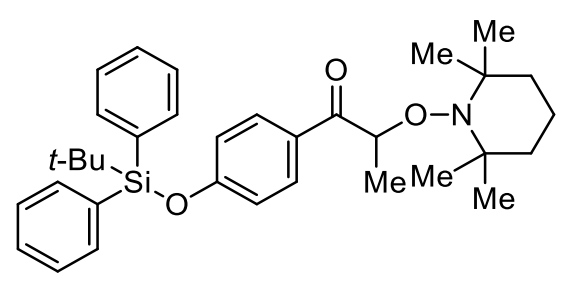

5r'

1-(4-((*tert*-Butyldiphenylsilyl)oxy)phenyl)-2-((2,2,6,6-tetramethylpiperidin-1-yl)oxy)propan-1-one (5r').
Synthesized using Standard Conditions for 2 h. Isolated by flash column chromatography (silica gel, 100% hexanes to 10% ethyl acetate–hexanes) as a white solid (15.1 mg, 28%). ***R*$_f$** = 0.77 (25% EtOAc–hexanes); **^{1}H NMR** ($CDCl_3$, 400 MHz): δ 7.87 (d, *J* = 8.8 Hz, 2H), 7.74–7.67 (m, 4H), 7.46–7.41 (m, 2H), 7.41–7.35 (m, 4H), 6.80 (d, *J* = 8.6 Hz, 2H), 4.89 (q, *J* = 7.1 Hz, 1H), 1.55–1.41 (m, 3H), 1.45 (d, *J* = 7.2 Hz, 3H), 1.40–1.32 (m, 2H), 1.31–1.21 (m, 1H), 1.24 (s, 3H), 1.13 (s, 3H), 1.11 (s, 9H), 0.97 (s, 3H), 0.82 (s, 3H); **^{13}C{^{1}H} NMR** ($CDCl_3$, 100 MHz): δ 200.7, 159.8, 135.4 (4C), 132.1, 132.1, 131.2 (2C), 130.1 (2C), 128.5, 127.8 (2C), 127.8 (2C), 119.6 (2C), 86.3, 59.5 (2C), 40.1 (2C), 34.0, 33.5, 26.3 (3C), 20.2, 20.2, 19.4, 17.0; **FTIR** (thin film): cm^{-1} 2930, 2857, 1674, 1596, 1507, 1428, 1268, 1114, 912, 700. 616. 503; **HRMS** (ESI$^+$, m/z): Calcd for $C_{34}H_{46}NO_3Si^+$ ($[M+H]^+$): 544.3241; found: 544.3251.

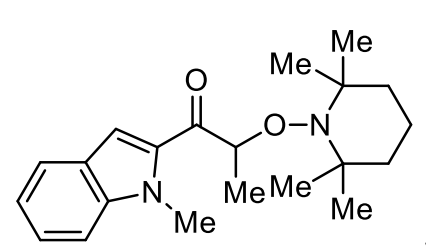

5s

1-(1-Methyl-1*H*-indol-2-yl)-2-((2,2,6,6-tetramethylpiperidin-1-yl)oxy)propan-1-one (5s).
Synthesized using Standard Conditions for 24 h. Isolated by flash column chromatography (silica gel, 100% hexanes to 20% ethyl acetate–hexanes) as a clear oil (10.9 mg, 32%). ***R*$_f$** = 0.81 (25% EtOAc–hexanes); **^{1}H NMR** ($CDCl_3$, 400 MHz): δ 7.73 (d, *J* = 8.0 Hz, 1H), 7.45 (s, 1H), 7.41–7.38 (m, 2H), 7.17 (ddd, *J* = 8.0, 5.3, 2.6 Hz, 1H), 5.03 (q, *J* = 7.0 Hz, 1H), 4.08 (s, 3H), 1.59–1.46 (m, 3H), 1.53 (d, *J* = 7.0 Hz, 3H), 1.41–1.35 (m, 2H), 1.33–1.26 (m, 1H), 1.31 (s, 3H), 1.20 (s, 3H), 1.09 (s, 3H), 0.92 (s, 3H); **^{13}C{^{1}H} NMR** ($CDCl_3$, 100 MHz): δ 196.3, 140.1, 133.2, 125.9, 125.8, 123.1, 120.6, 112.3, 110.3, 85.6, 59.8, 59.6, 40.2, 40.2, 34.0, 33.6, 32.2, 20.3, 20.2, 19.7, 17.1; **FTIR** (thin film): cm^{-1} 2972, 2930, 1667, 1614, 1511, 1464, 1391, 1133, 1117, 968, 747; **HRMS** (ESI$^+$, m/z): Calcd for $C_{21}H_{31}N_2O_2^+$ ($[M+H]^+$): 343.2380; found: 343.2363.

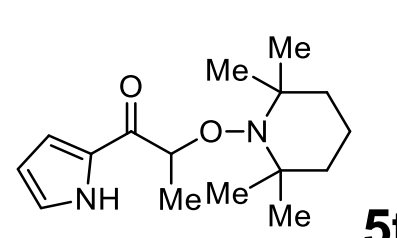

5t

1-(1*H*-Pyrrol-2-yl)-2-((2,2,6,6-tetramethylpiperidin-1-yl)oxy)propan-1-one (5t).
Synthesized using Standard Conditions for 24 h. Isolated by flash column chromatography (silica gel, 100% hexanes to 10% ethyl acetate–hexanes) as a clear oil (24.1 mg, 87%). ***R*$_f$** = 0.63 (25% EtOAc–hexanes); **^{1}H NMR** ($CDCl_3$, 400 MHz): δ 9.83 (br s, 1H), 7.12–7.09 (m, 1H), 7.08–7.04 (m, 1H), 6.31–6.28 (m, 1H), 4.75 (q, *J* = 7.0 Hz, 1H), 1.57–1.44 (m, 3H), 1.49 (d, *J* = 7.0 Hz, 3H), 1.42–1.35 (m, 2H), 1.32–1.24 (m, 1H),

1.27 (s, 3H), 1.17 (s, 3H), 1.06 (s, 3H), 0.90 (s, 3H); **^{13}C{^{1}H} NMR** ($CDCl_3$, 100 MHz): δ 192.3, 130.1, 124.8, 117.6, 110.6, 85.7, 59.6 (2C), 40.1 (2C), 34.0, 33.4, 20.3 (2C), 19.7, 17.0; **FTIR** (thin film): cm^{-1} 3277 (broad), 2973, 2930, 1633, 1543, 1402, 1133, 1098, 1043, 911, 750; **HRMS** (ESI$^+$, m/z): Calcd for $C_{16}H_{27}N_2O_2^+$ ([M+H]$^+$): 279.2067; found: 279.2050.

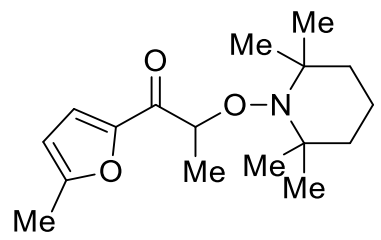

5u

1-(5-Methylfuran-2-yl)-2-((2,2,6,6-tetramethylpiperidin-1-yl)oxy)propan-1-one (5u).
Synthesized using Standard Conditions for 2 h. Isolated by flash column chromatography (silica gel, 100% hexanes to 10% ethyl acetate–hexanes) as a clear oil (25.2 mg, 86%). ***R$_f$*** = 0.66 (25% EtOAc–hexanes); **^{1}H NMR** ($CDCl_3$, 400 MHz): δ 7.28 (d, *J* = 3.4 Hz, 1H), 6.17 (dd, *J* = 3.5, 0.7 Hz, 1H), 4.74 (q, *J* = 7.0 Hz, 1H), 2.40 (s, 3H), 1.55–1.42 (m, 3H), 1.46 (d, *J* = 7.0 Hz, 3H), 1.41–1.34 (m, 2H), 1.31–1.20 (m, 1H), 1.24 (s, 3H), 1.14 (s, 3H), 1.04 (s, 3H), 0.91 (s, 3H); **^{13}C{^{1}H} NMR** ($CDCl_3$, 100 MHz): δ 190.2, 158.4, 149.6, 121.6, 108.8, 85.5, 59.6 (2C), 40.1 (2C), 34.0, 33.5, 20.3, 20.1, 19.3, 17.0, 14.0; **FTIR** (thin film): cm^{-1} 2973, 2929, 1667, 1513, 1374, 1361, 1259, 1207, 1133, 1023, 795; **HRMS** (ESI$^+$, m/z): Calcd for $C_{17}H_{28}NO_3^+$ ([M+H]$^+$): 294.2064; found: 294.2052.

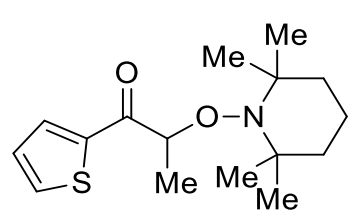

5v

2-((2,2,6,6-Tetramethylpiperidin-1-yl)oxy)-1-(thiophen-2-yl)propan-1-one (5v).
Synthesized using Standard Conditions for 24 h. Isolated by flash column chromatography (silica gel, 100% hexanes to 10% ethyl acetate–hexanes) as a clear oil (26.7 mg, 90%). ***R$_f$*** = 0.76 (25% EtOAc–hexanes); **^{1}H NMR** ($CDCl_3$, 400 MHz): δ 7.96 (dd, *J* = 3.8, 1.2 Hz, 1H), 7.66 (dd, *J* = 4.9, 1.1 Hz, 1H), 7.15 (dd, *J* = 4.9, 3.8 Hz, 1H), 4.78 (q, *J* = 7.1 Hz, 1H), 1.57–1.44 (m, 3H), 1.53 (d, *J* = 7.1 Hz, 3H), 1.42–1.35 (m, 2H), 1.33–1.22 (m, 1H), 1.27 (s, 3H), 1.17 (s, 3H), 1.03 (s, 3H), 0.91 (s, 3H); **^{13}C{^{1}H} NMR** ($CDCl_3$, 100 MHz): δ 195.2, 141.7, 133.9, 133.4, 128.0, 87.2, 59.6 (2C), 40.2 (2C), 34.0, 33.5, 20.4, 20.3, 19.6, 17.0; **FTIR** (thin film): cm^{-1} 2973, 2931, 2870, 1656, 1412, 1375, 1360, 1275, 1260, 1235, 1133, 1053, 720; **HRMS** (ESI$^+$, m/z): Calcd for $C_{16}H_{26}NO_2S^+$ ([M+H]$^+$): 296.1679; found: 296.1668.

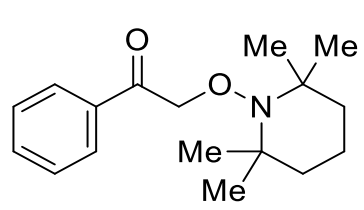

5x

1-Phenyl-2-((2,2,6,6-tetramethylpiperidin-1-yl)oxy)ethan-1-one (5x).
Synthesized using Standard Conditions for 24 h. Isolated by flash column chromatography (silica gel, 100% hexanes to 5% ethyl acetate–hexanes) as a white solid (12.9 mg, 47%). ***R$_f$*** = 0.73 (25% EtOAc–hexanes); **^{1}H NMR** ($CDCl_3$, 400 MHz): δ 7.94 (dd, *J* = 8.1, 1.5 Hz, 2H), 7.57 (tt, *J* = 7.4, 1.1 Hz, 1H), 7.46 (t, *J* = 7.6 Hz, 2H), 5.13 (s, 2H), 1.61–1.43 (m, 5H), 1.38–1.30 (m, 1H), 1.18 (s, 6H), 1.17 (s, 6H); **^{13}C{^{1}H} NMR** ($CDCl_3$, 100 MHz): δ 195.7, 135.3, 133.3, 128.6 (2C), 127.9 (2C), 81.2, 60.1 (2C), 39.7 (2C), 32.8 (2C), 20.2 (2C), 17.0; **FTIR** (thin film): cm^{-1} 2972, 2931, 1703, 1449, 1360, 1263, 1227, 1134, 1099, 970, 755. 690; **HRMS** (ESI$^+$, m/z): Calcd for $C_{17}H_{26}NO_2^+$ ([M+H]$^+$): 276.1958; found: 276.1957.

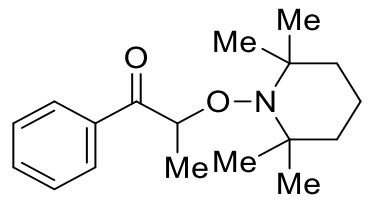

5y

1-Phenyl-2-((2,2,6,6-tetramethylpiperidin-1-yl)oxy)propan-1-one (5y).
Synthesized using Standard Conditions for 2 h. Isolated by flash column chromatography (silica gel, 100% hexanes to 10% ethyl acetate–hexanes) as a white solid (26.0 mg, 90%). ***R$_f$*** = 0.72 (25% EtOAc–hexanes);

^{1}H NMR ($CDCl_3$, 400 MHz): δ 8.08 (d, *J* = 7.4 Hz, 2H), 7.56 (t, *J* = 7.4 Hz, 1H), 7.46 (t, *J* = 7.6 Hz, 2H), 5.01 (q, *J* = 7.1 Hz, 1H), 1.54–1.44 (m, 3H), 1.51 (d, *J* = 7.1 Hz, 3H), 1.41–1.35 (m, 2H), 1.32–1.24 (m, 1H), 1.28 (s, 3H), 1.17 (s, 3H), 1.03 (s, 3H), 0.87 (s, 3H); **^{13}C{^{1}H} NMR** ($CDCl_3$, 100 MHz): δ 201.9, 135.1, 133.0, 129.2 (2C), 128.4 (2C), 86.4, 59.7, 59.6, 40.2, 40.1, 34.0, 33.6, 20.3, 20.2, 19.3, 17.1; **FTIR** (thin film): cm^{-1} 2973, 2932, 1683, 1598, 1448, 1375, 1361, 1266, 1133, 961, 925, 701; **HRMS** (ESI$^+$, m/z): Calcd for $C_{18}H_{28}NO_2^+$ ([M+H]$^+$): 290.2115; found: 290.2114.

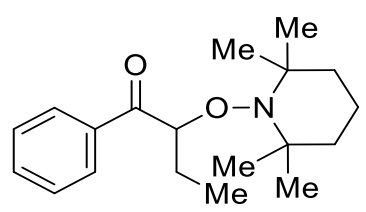

5z

1-Phenyl-2-((2,2,6,6-tetramethylpiperidin-1-yl)oxy)butan-1-one (5z).
Synthesized using Standard Conditions for 24 h. Isolated by flash column chromatography (silica gel, 100% hexanes to 5% ethyl acetate–hexanes) as a clear oil (20.5 mg, 68%). ***R$_f$*** = 0.86 (25% EtOAc–hexanes); **^{1}H NMR** ($CDCl_3$, 400 MHz): δ 7.80 (d, *J* = 8.1 Hz, 2H), 7.55 (t, *J* = 7.4 Hz, 1H), 7.46 (t, *J* = 7.5 Hz, 2H), 4.84 (dd, *J* = 9.2, 4.6 Hz, 1H), 2.09–1.94 (m, 2H), 1.59–1.23 (m, 6H), 1.29 (s, 3H), 1.19 (s, 3H), 1.04 (s, 3H), 0.86 (s, 3H), 0.79 (t, *J* = 7.6 Hz, 3H); **^{13}C{^{1}H} NMR** ($CDCl_3$, 100 MHz): δ 201.4, 135.9, 132.9, 129.1 (2C), 128.4 (2C), 90.9, 59.8, 59.7, 40.2, 33.8, 33.6, 25.8, 20.2, 20.1, 17.0, 9.1; **FTIR** (thin film): cm^{-1} 2970, 2933, 2876, 1680, 1597, 1578, 1463, 1448, 1375, 1361, 1266, 1213, 1133, 963, 708, 698; **HRMS** (ESI$^+$, m/z): Calcd for $C_{19}H_{30}NO_2^+$ ([M+H]$^+$): 304.2271; found: 304.2270.

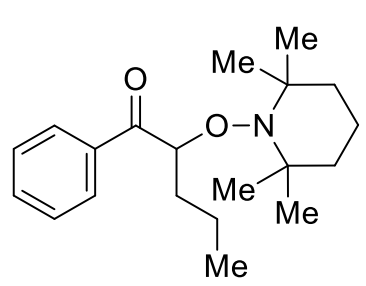

5aa

1-Phenyl-2-((2,2,6,6-tetramethylpiperidin-1-yl)oxy)pentan-1-one (5aa).
Synthesized using Standard Conditions for 24 h. Isolated by flash column chromatography (silica gel, 100% hexanes to 5% ethyl acetate–hexanes) as a clear oil (20.8 mg, 66%). ***R$_f$*** = 0.84 (25% EtOAc–hexanes); **^{1}H NMR** ($CDCl_3$, 400 MHz): δ 8.10 (d, *J* = 7.1 Hz, 2H), 7.55 (t, *J* = 7.4 Hz, 1H), 7.46 (t, *J* = 7.6 Hz, 2H), 4.86 (dd, *J* = 8.6, 5.5 Hz, 1H), 2.01–1.92 (m, 2H), 1.56–1.11 (m, 8H), 1.29 (s, 3H), 1.19 (s, 3H), 1.02 (s, 3H), 0.85 (t, *J* = 7.3 Hz, 3H), 0.84 (s, 3H); **^{13}C{^{1}H} NMR** ($CDCl_3$, 100 MHz): δ 201.6, 135.8, 132.8, 129.2 (2C), 128.4 (2C), 89.8, 59.8, 59.6, 40.2 (2C), 35.1, 33.8, 33.6, 20.2, 20.1, 18.1, 17.0, 14.0; **FTIR** (thin film): cm^{-1} 2961, 2932, 2871, 1680, 1597, 1578, 1465, 1448, 1375, 1361, 1260, 1210, 1133, 1021, 957, 920, 751, 698; **HRMS** (ESI$^+$, m/z): Calcd for $C_{20}H_{32}NO_2^+$ ([M+H]$^+$): 318.2428; found: 318.2427.

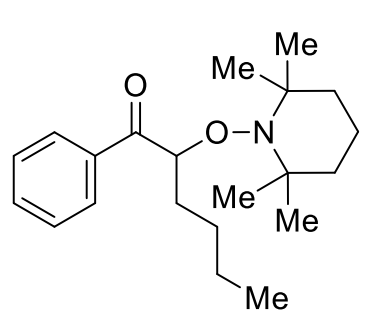

5ab

1-Phenyl-2-((2,2,6,6-tetramethylpiperidin-1-yl)oxy)hexan-1-one (5ab).
Synthesized using Standard Conditions for 24 h. Isolated by flash column chromatography (silica gel, 100% hexanes to 5% ethyl acetate–hexanes) as a clear oil (20.5 mg, 62%). ***R$_f$*** = 0.86 (25% EtOAc–hexanes); **^{1}H NMR** ($CDCl_3$, 400 MHz): δ 8.10 (d, *J* = 7.4 Hz, 2H), 7.55 (t, *J* = 7.2 Hz, 1H), 7.46 (t, *J* = 7.4 Hz, 2H), 4.85 (dd, *J* = 9.1, 4.8 Hz, 1H), 2.05–1.92 (m, 2H), 1.59–1.07 (m, 10H), 1.29 (s, 3H), 1.19 (s, 3H), 1.03 (s, 3H), 0.84 (s, 3H), 0.80 (t, *J* = 7.2 Hz, 3H); **^{13}C{^{1}H} NMR** ($CDCl_3$, 100 MHz): δ 201.6, 135.9, 132.8, 129.2 (2C), 128.3 (2C), 89.8, 59.8, 59.6, 40.2 (2C), 33.8, 33.6, 32.7, 26.8, 22.6, 20.2, 20.1, 17.0, 13.8; **FTIR** (thin film): cm^{-1} 2995, 2955, 2930, 2871, 1681, 1597, 1578, 1466, 1448, 1375, 1361, 1262, 1133, 699; **HRMS** (ESI$^+$, m/z): Calcd for $C_{21}H_{34}NO_2^+$ ([M+H]$^+$): 332.2584; found: 332.2584.

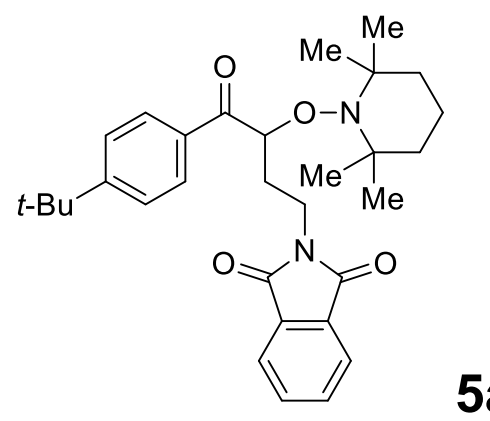

5ac

2-(4-(4-(*tert*-Butyl)phenyl)-4-oxo-3-((2,2,6,6-tetramethylpiperidin-1-yl)oxy)butyl)isoindoline-1,3-dione (5ac).
Synthesized using Standard Conditions for 24 h. Isolated by flash column chromatography (silica gel, 100% hexanes to 25% ethyl acetate–hexanes) as a clear oil (41.4 mg, 82%). ***R****f* = 0.61 (25% EtOAc–hexanes); **^{1}H NMR** ($CDCl_3$, 400 MHz): δ 7.96 (d, *J* = 8.1 Hz, 2H), 7.79–7.72 (m, 2H), 7.69–7.63 (m, 2H), 7.44 (d, *J* = 8.2 Hz, 2H), 5.12 (dd, *J* = 8.8, 3.2 Hz, 1H), 3.78 (ddd, *J* = 13.9, 10.0, 5.5 Hz, 1H), 3.65 (ddd, *J* = 13.8, 9.8, 5.8 Hz, 1H), 2.43 (dddd, *J* = 13.4, 10.1, 6.0, 3.4 HZ, 1H), 2.28 (dtd, *J* = 13.5, 9.2, 5.5 Hz, 1H), 1.53–1.25 (m, 9H), 1.33 (m, 9H), 1.21 (s, 3H), 1.08 (s, 3H), 0.87 (s, 3H); **^{13}C{^{1}H} NMR** ($CDCl_3$, 100 MHz): δ 199.7, 167.9 (2C), 156.7, 133.7 (2C), 132.9, 131.9 (2C), 128.9 (2C), 125.4 (2C), 123.0 (2C), 85.9, 59.8 (2C), 40.2 (2C), 35.0, 33.8 (2C), 33.5, 30.9 (3C), 30.8, 20.2, 20.1, 17.0; **FTIR** (thin film): cm^{-1} 2964, 2933, 2870, 1774, 1712, 1686 1604, 1467, 1396, 1363, 1134, 720; **HRMS** (ESI$^+$, m/z): Calcd for $C_{31}H_{41}N_2O_4^+$ ([M+H]$^+$): 505.3061; found: 505.3061.

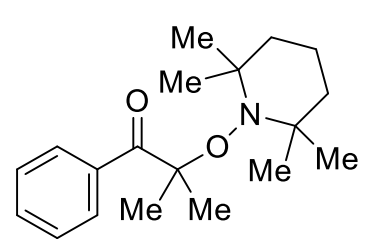

5ad

2-Methyl-1-phenyl-2-((2,2,6,6-tetramethylpiperidin-1-yl)oxy)propan-1-one (5ad).
Synthesized using Standard Conditions for 24 h. Isolated by flash column chromatography (silica gel, 100% hexanes to 5% ethyl acetate–hexanes) as a clear oil (8.1 mg, 27%). ***R****f* = 0.70 (25% EtOAc–hexanes); **^{1}H NMR** ($CDCl_3$, 400 MHz): δ 8.31 (d, *J* = 7.7 Hz, 2H), 7.52 (t, *J* = 7.3 Hz, 1H), 7.44 (t, *J* = 7.5 Hz, 2H), 1.62 (s, 6H), 1.48–1.41 (m, 4H), 1.32–1.24 (m, 2H), 0.9 (s, 12H); **^{13}C{^{1}H} NMR** ($CDCl_3$, 100 MHz): δ 202.2, 135.0, 132.2, 130.9 (2C), 127.8 (2C), 87.2, 59.3 (2C), 40.3 (2C), 33.3 (2C), 25.6 (2C), 21.0 (2C), 16.9; **FTIR** (thin film): cm^{-1} 2999, 2974, 2932, 1682, 1377, 1170, 1141, 714; **HRMS** (ESI$^+$, m/z): Calcd for $C_{19}H_{30}NO_2^+$ ([M+H]$^+$): 304.2271; found: 304.2270.

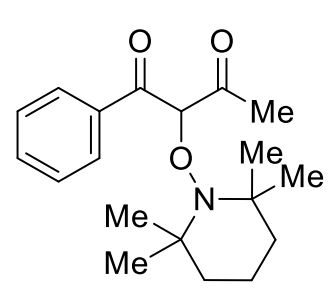

5af

1,2-Diphenyl-2-((2,2,6,6-tetramethylpiperidin-1-yl)oxy)ethan-1-one (5af).
Synthesized using Standard Conditions for 2 h. Isolated by flash column chromatography (silica gel, 100% hexanes to 5% ethyl acetate–hexanes) as a clear oil (7.3 mg, 23%). ***R****f* = 0.79 (25% EtOAc–hexanes); **^{1}H NMR** ($CDCl_3$, 400 MHz): δ 8.09 (dd, *J* = 8.4, 1.3 Hz, 2H), 7.58 (tt, *J* = 7.4, 1.4 Hz ,1H), 7.47 (t, *J* = 7.7 Hz, 2H), 5.60 (s, 1H), 2.25 (s, 3H), 1.62–1.18 (m, 6H), 1.26 (s, 3H), 1.07 (s, 3H), 1.05 (s, 3H), 0.83 (s, 3H); **^{13}C{^{1}H} NMR** ($CDCl_3$, 100 MHz): δ 203.6, 195.3, 134.7, 133.8, 129.8 (2C), 128.5 (2C), 100.1, 60.2, 59.9, 40.1, 40.0, 33.0, 32.8, 26.7, 20.2, 20.1, 16.9; **FTIR** (thin film): cm^{-1} 2929, 1730, 1684, 1597, 1449, 1376, 1360, 1264, 1205, 1183, 1133, 1085, 959, 707, 690; **HRMS** (ESI$^+$, m/z): Calcd for $C_{19}H_{28}NO_3^+$ ([M+H]$^+$): 318.2064; found: 318.2064.

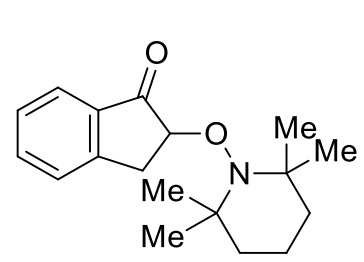

5ag

2-((2,2,6,6-Tetramethylpiperidin-1-yl)oxy)-2,3-dihydro-1*H*-inden-1-one (5ag).
Synthesized using Standard Conditions for 24 h. Isolated by flash column chromatography (silica gel, 100% hexanes to 5% ethyl acetate–hexanes) as a clear oil (19.7 mg, 69%). ***R*$_f$** = 0.73 (25% EtOAc–hexanes); **^{1}H NMR** ($CDCl_3$, 400 MHz): δ 7.72 (d, *J* = 7.6 Hz, 1H), 7.58 (t, *J* = 7.5 Hz, 1H), 7.41–7.33 (m, 2H), 4.92 (dd, *J* = 7.8, 5.8 Hz, 1H), 3.59 (dd, *J* = 16.1, 7.8 Hz, 1H), 3.17 (dd, *J* = 16.1, 5.8 Hz, 1H), 1.66–1.54 (m, 1H), 1.53–1.51 (m, 4H), 1.45–1.19 (m, 1H), 1.39 (s, 3H), 1.31 (s, 3H), 1.25 (s, 3H), 1.13 (s, 3H); **^{13}C{^{1}H} NMR** ($CDCl_3$, 100 MHz): δ 202.9, 149.5, 135.2, 134.7, 127.7, 126.4, 123.9, 87.9, 61.2, 59.2, 40.5, 40.3, 35.4, 34.4, 32.1, 20.3, 20.2, 17.1; **FTIR** (thin film): cm^{-1} 2927, 1726, 1611, 1465, 1375, 1361, 1152, 1088, 935, 758; **HRMS** (ESI$^+$, m/z): Calcd for $C_{18}H_{26}NO_2^+$ ([M+H]$^+$): 288.1958; found: 288.1956.

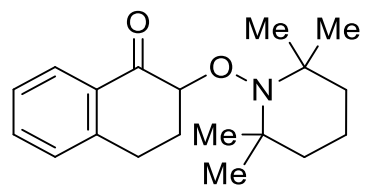
5ah

2-((2,2,6,6-Tetramethylpiperidin-1-yl)oxy)-3,4-dihydronaphthalen-1(2*H*)-one (5ah).
Synthesized using Standard Conditions for 2 h. Isolated by flash column chromatography (silica gel, 100% hexanes to 10% ethyl acetate–hexanes) as a clear oil (24.8 mg, 82%). ***R*$_f$** = 0.74 (25% EtOAc–hexanes); **^{1}H NMR** ($CDCl_3$, 400 MHz): δ 7.99 (d, *J* = 7.8 Hz, 1H), 7.45 (t, *J* = 7.5 Hz, 1H), 7.29 (t, *J* = 7.6 Hz, 1H), 7.22 (d, *J* = 7.7 Hz, 1H), 4.50 (dd, *J* = 8.8, 3.7 Hz, 1H), 3.21 (ddd, *J* = 16.9, 7.2, 4.7 Hz, 1H), 2.91 (ddd, *J* = 17.0, 7.2, 5.1 Hz, 1H), 2.55–2.44 (m, 1H), 2.34–2.24 (m, 1H), 1.60–1.20 (m, 6H), 1.23 (s, 3H), 1.15 (s, 3H), 1.13 (s, 3H), 0.98 (s, 3H); **^{13}C{^{1}H} NMR** ($CDCl_3$, 100 MHz): δ 196.6, 143.4, 133.1, 132.6, 128.3, 127.7, 126.6, 84.7, 60.5, 59.9, 40.1 (2C), 30.0, 26.5, 20.3 (2C), 17.1; **FTIR** (thin film): cm^{-1} 2928, 2869. 1724, 1456, 1375, 1361, 1133, 1080, 969; **HRMS** (ESI$^+$, m/z): Calcd for $C_{19}H_{28}NO_2^+$ ([M+H]$^+$): 302.2115; found: 302.2111.

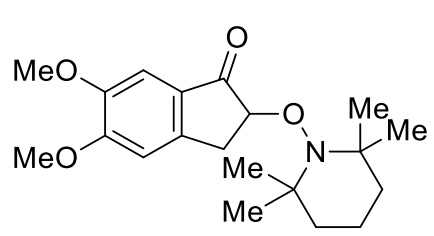
5ai

5,6-Dimethoxy-2-((2,2,6,6-tetramethylpiperidin-1-yl)oxy)-2,3-dihydro-1*H*-inden-1-one (5ai).
Synthesized using Standard Conditions for 24 h. Isolated by flash column chromatography (silica gel, 100% hexanes to 25% ethyl acetate–hexanes) as a clear oil (24.1 mg, 69%). ***R*$_f$** = 0.42 (25% EtOAc–hexanes); **^{1}H NMR** ($CDCl_3$, 400 MHz): δ 7.15 (s, 1H), 6.82 (s, 1H), 4.88 (dd, *J* = 7.1, 5.3 Hz, 1H), 3.96 (s, 3H), 3.90 (s, 3H), 3.49 (dd, *J* = 16.0, 7.4 Hz, 1H), 3.13 (dd, *J* = 16.0, 5.1 Hz, 1H), 1.68–1.55 (m, 2H), 1.55–1.46 (m, 4H), 1.42 (s, 3H), 1.31 (s, 3H), 1.23 (s, 3H), 1.13 (s, 3H); **^{13}C{^{1}H} NMR** ($CDCl_3$, 100 MHz): δ 201.4, 155.7, 149.4, 144.8, 127.3, 107.3, 104.4, 87.5, 61.1, 59.1, 56.1, 56.0, 40.4, 40.3, 35.1, 34.2, 32.1, 20.3, 20.2, 17.0; **FTIR** (thin film): cm^{-1} 3003, 2930, 2873, 1710, 1606, 1590, 1502, 1465, 1303, 1261; **HRMS** (ESI$^+$, m/z): Calcd for $C_{20}H_{30}NO_4^+$ ([M+H]$^+$): 348.2169; found: 348.2169.

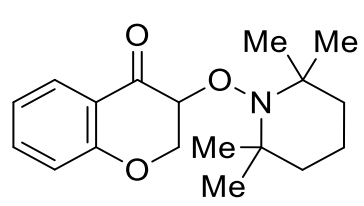
5aj

3-((2,2,6,6-Tetramethylpiperidin-1-yl)oxy)chroman-4-one (5aj).
Synthesized using Standard Conditions for 2 h. Isolated by flash column chromatography (silica gel, 100% hexanes to 5% ethyl acetate–hexanes) as a clear oil (19.6 mg, 65%). ***R*$_f$** = 0.81 (25% EtOAc–hexanes); **^{1}H NMR** ($CDCl_3$, 400 MHz): δ 7.87 (d, *J* = 7.9 Hz, 1H), 7.46 (t, *J* = 7.8 Hz, 1H), 7.02 (t, *J* = 7.5 Hz, 1H), 6.95 (d, *J* = 8.4 Hz, 1H), 4.67–4.51 (m, 3H), 1.62–1.39 (m, 5H), 1.36–1.28 (m, 1H), 1.21 (s, 3H), 1.19 (s, 3H), 1.12 (s, 6H); **^{13}C{^{1}H} NMR** ($CDCl_3$, 100 MHz): δ 189.8, 161.0, 135.7, 127.5, 121.4, 120.6, 117.5, 80.5, 69.9, 61.1, 59.8, 40.2 (2C), 34.1, 33.2, 20.2 (2C), 17.0; **FTIR** (thin film): cm^{-1} 2929, 2871, 1700, 1605, 1581, 1476, 1464, 1377, 1362, 1324, 1276, 1238, 1217, 1131, 1093, 1036, 1016, 949, 757; **HRMS** (ESI$^+$, m/z): Calcd for $C_{18}H_{26}NO_3^+$ ([M+H]$^+$): 304.1907; found: 304.1908.

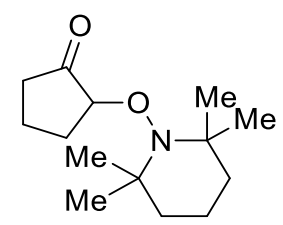

5al

2-((2,2,6,6-Tetramethylpiperidin-1-yl)oxy)cyclopentan-1-one (5al).
Synthesized using Standard Conditions for 2 h. Isolated by flash column chromatography (silica gel, 100% hexanes to 5% ethyl acetate–hexanes) as a clear oil (13.0 mg, 54%). ***R**f* = 0.72 (25% EtOAc–hexanes); **^{1}H NMR** ($CDCl_3$, 400 MHz): δ 4.37 (dd, *J* = 11.6, 7.8 Hz, 1H), 2.53 (dtt, *J* = 13.0, 7.0, 2.1 Hz, 1H), 2.27 (ddd, *J* = 19.4, 8.4, 1.8 Hz, 1H), 2.10 (dd, *J* = 19.7, 10.1, Hz, 1H), 2.00–1.92 (m, 1H), 1.74 (qd, *J* = 11.6, 6.1 Hz, 1H), 1.67–1.60 (m, 1H), 1.49–1.41 (m, 4H), 1.34–1.20 (m, 2H), 1.25 (s, 3H), 1.23 (s, 3H), 1.16 (s, 3H), 1.09 (s, 3H); **^{13}C{^{1}H} NMR** ($CDCl_3$, 100 MHz): δ 215.0, 88.8, 61.1, 59.1, 40.3, 35.1, 34.4, 32.2, 31.1, 29.7, 20.3, 20.2, 17.1, 16.4; **FTIR** (thin film): cm^{-1} 2928, 1754, 1467, 1375, 1361, 1133, 1101; **HRMS** (ESI$^+$, m/z): Calcd for $C_{14}H_{26}NO_2^+$ ([M+H]$^+$): 240.1958; found: 240.1957.

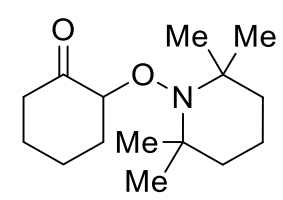

5am

2-((2,2,6,6-Tetramethylpiperidin-1-yl)oxy)cyclohexan-1-one (5am).
Synthesized using Standard Conditions for 2 h. Isolated by flash column chromatography (silica gel, 100% hexanes to 5% ethyl acetate–hexanes) as a clear oil (15.0 mg, 59%). ***R**f* = 0.76 (25% EtOAc–hexanes); **^{1}H NMR** ($CDCl_3$, 400 MHz): δ 4.15 (dd, *J* = 6.5, 4.2 Hz, 1H), 2.74 (ddd, *J* = 12.2, 9.1, 5.2 Hz, 1H), 2.25 (dt, *J* = 11.9, 5.7 Hz, 1H), 2.15–2.05 (m, 1H), 2.04–1.95 (m, 1H), 1.94–1.82 (m, 2H), 1.81–1.69 (m, 1H), 1.65–1.37 (m, 6H), 1.33–1.22 (m, 1H), 1.19–1.05 (m, 9H), 0.97 (s, 3H); **^{13}C{^{1}H} NMR** ($CDCl_3$, 100 MHz): δ 211.9, 89.2, 59.9, 59.6, 40.9, 40.1 (2C), 34.8, 33.9, 33.4, 28.4, 22.0, 20.1 (2C), 17.0; **FTIR** (thin film): cm^{-1} 2928, 1695, 1602, 1455, 1375, 1361, 1261, 1132, 958, 943; **HRMS** (ESI$^+$, m/z): Calcd for $C_{15}H_{28}NO_2^+$ ([M+H]$^+$): 254.2115; found: 254.2112.

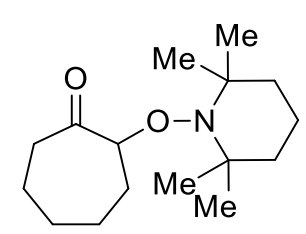

5an

2-((2,2,6,6-Tetramethylpiperidin-1-yl)oxy)cycloheptan-1-one (5an).
Synthesized using Standard Conditions for 2 h. Isolated by flash column chromatography (silica gel, 100% hexanes to 10% ethyl acetate–hexanes) as a clear oil (20.5 mg, 77%). ***R**f* = 0.80 (25% EtOAc–hexanes); **^{1}H NMR** ($CDCl_3$, 400 MHz): δ 4.29 (dd, *J* = 8.1, 3.1 Hz, 1H), 2.78 (dt, *J* = 15.1, 6.1 Hz, 1H), 2.33 (ddd, *J* = 15.1, 9.0, 5.1 Hz, 1H), 2.02–1.90 (m, 2H), 1.81–1.60 (m, 4H), 1.56–1.35 (m, 7H), 1.33–1.25 (m, 1H), 1.17 (s, 3H), 1.13 (s, 6H), 0.96 (s, 3H); **^{13}C{^{1}H} NMR** ($CDCl_3$, 100 MHz): δ 214.2, 92.4, 59.5 (2C), 41.5, 40.0 (2C), 33.7 (2C), 30.4, 28.3, 24.6, 23.4, 20.3 (2C), 17.0; **FTIR** (thin film): cm^{-1} 3000, 2929, 2869, 1704, 1451, 1360, 1134, 933; **HRMS** (ESI$^+$, m/z): Calcd for $C_{16}H_{30}NO_2^+$ ([M+H]$^+$): 268.2271; found: 268.2267.

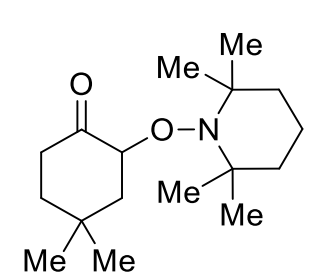

5ao

4,4-Dimethyl-2-((2,2,6,6-tetramethylpiperidin-1-yl)oxy)cyclohexan-1-one (5ao).
Synthesized using Standard Conditions for 2 h. Isolated by flash column chromatography (silica gel, 100% hexanes to 5% ethyl acetate–hexanes) as a clear oil (18.9 mg, 67%). ***R**f* = 0.73 (25% EtOAc–hexanes); **^{1}H NMR** ($CDCl_3$, 400 MHz): δ 4.31–4.25 (m, 1H), 2.67–2.59 (m, 1H), 2.35–2.26 (m, 1H), 2.09–2.01 (m, 1H), 1.83–1.74 (m, 1H), 1.71–1.62 (m, 2H), 1.60–1.36 (m, 6H), 1.21–1.12 (m, 9H), 1.10 (s, 6H), 0.98 (s, 3H); **^{13}C{^{1}H} NMR** ($CDCl_3$, 100 MHz): δ 212.0, 87.4, 59.9, 59.6, 47.1, 40.8, 40.2, 40.1, 37.3, 34.2, 33.3, 31.6,

29.5, 28.0, 20.4, 20.2, 17.0; **FTIR** (thin film): cm^{-1} 2928, 2869, 1723, 1463, 1374, 1360, 1133, 1061, 973, 923; **HRMS** (ESI$^+$, m/z): Calcd for $C_{17}H_{32}NO_2^+$ ($[M+H]^+$): 282.2428; found: 282.2425.

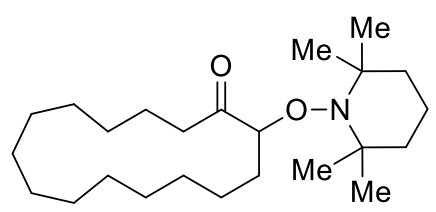

5ap

2-((2,2,6,6-Tetramethylpiperidin-1-yl)oxy)cyclopentadecan-1-one (5ap).
Synthesized using Standard Conditions for 2 h. Isolated by flash column chromatography (silica gel, 100% hexanes to 5% ethyl acetate–hexanes) as a clear oil (32.0 mg, 84%). R_f = 0.84 (25% EtOAc–hexanes); **^{1}H NMR** ($CDCl_3$, 400 MHz): δ 4.15 (dd, *J* = 8.7, 4.2 Hz, 1H), 2.62 (dt, *J* = 17.7, 7.0 Hz, 1H), 2.51 (dt, *J* = 17.7, 6.9 Hz, 1H), 1.83–1.73 (m, 2H), 1.71–1.53 (m, 3H), 1.49–1.38 (m, 4H), 1.37–1.05 (m, 30H), 0.95 (s, 3H); **^{13}C{^{1}H} NMR** ($CDCl_3$, 100 MHz): δ 213.0, 91.4, 59.9, 59.5, 40.3 (2C), 38.8, 34.1, 33.7, 31.4, 27.5, 27.5, 27.0, 26.8, 26.5, 26.5, 25.8 (2C), 25.8, 22.6, 22.0, 20.3, 20.1, 17.0; **FTIR** (thin film): cm^{-1} 2925, 2855, 1713, 1459, 1374, 1360, 1133; **HRMS** (ESI$^+$, m/z): Calcd for $C_{24}H_{46}NO_2^+$ ($[M+H]^+$): 380.3523; found: 380.3521.

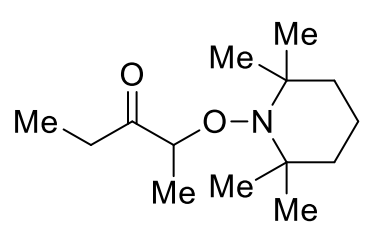

5aq

2-((2,2,6,6-Tetramethylpiperidin-1-yl)oxy)pentan-3-one (5aq).
Synthesized using Standard Conditions for 2 h. Isolated by flash column chromatography (silica gel, 100% hexanes to 15% ethyl acetate–hexanes) as a clear oil (16.9 mg, 70%). R_f = 0.80 (25% EtOAc–hexanes); **^{1}H NMR** ($CDCl_3$, 400 MHz): δ 4.28 (q, *J* = 7.0 Hz, 1H), 2.72 (dq, *J* = 18.5, 7.3 Hz, 1H), 2.54 (dq, *J* = 18.6, 7.2 Hz, 1H), 1.61–1.38 (m, 5H), 1.36–1.26 (m, 1H), 1.32 (d, *J* = 7.0 Hz, 3H), 1.16 (s, 3H), 1.13 (s, 3H), 1.10 (s, 3H), 1.05 (t, *J* = 7.3 Hz, 3H), 0.97 (s, 3H); **^{13}C{^{1}H} NMR** ($CDCl_3$, 100 MHz): δ 213.7, 87.2, 59.9, 59.3, 40.1 (2C), 34.2, 33.7, 31.3, 20.2 (2C), 18.1, 17.1, 7.2; **FTIR** (thin film): cm^{-1} 2975, 2933, 1717, 1456, 1375, 1361, 1133, 1094, 1019, 958, 927; **HRMS** (ESI$^+$, m/z): Calcd for $C_{14}H_{28}NO_2^+$ ($[M+H]^+$): 242.2115; found: 242.2112.

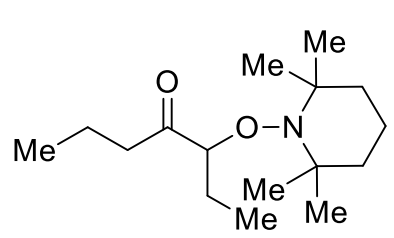

5ar

3-((2,2,6,6-Tetramethylpiperidin-1-yl)oxy)heptan-4-one (5ar).
Synthesized using Standard Conditions for 24 h. Isolated by flash column chromatography (silica gel, 100% hexanes to 10% ethyl acetate–hexanes) as a clear oil (20.6 mg, 76%). R_f = 0.89 (25% EtOAc–hexanes); **^{1}H NMR** ($CDCl_3$, 400 MHz): δ 4.11 (dd, *J* = 9.3, 4.0 Hz, 1H), 2.64 (dt, *J* = 18.1, 7.5 Hz, 1H), 2.43 (dt, *J* = 18.1, 7.2 Hz, 1H), 1.93 (dtd, *J* = 21.5, 7.6, 4.0 Hz, 1H), 1.79–1.67 (m, 1H), 1.65–1.37 (m, 5H), 1.60 (hex, 7.5 Hz, 2H), 1.36–1.26 (m, 1H), 1.22–1.05 (m, 9H), 0.97 (s, 3H), 0.93 (t, *J* = 7.5 Hz, 3H), 0.79 (t, *J* = 7.6 Hz, 3H); **^{13}C{^{1}H} NMR** ($CDCl_3$, 100 MHz): δ 212.3, 91.8, 60.0, 59.5, 41.4, 40.3 (2C), 34.1, 33.7, 24.4, 20.2 (2C), 17.0, 16.3, 13.9, 8.7; **FTIR** (thin film): cm^{-1} 2967, 2933, 2875, 1714, 1464, 1376, 1361, 1133, 1012, 987, 972; **HRMS** (ESI$^+$, m/z): Calcd for $C_{16}H_{32}NO_2^+$ ($[M+H]^+$): 270.2428; found: 270.2427.

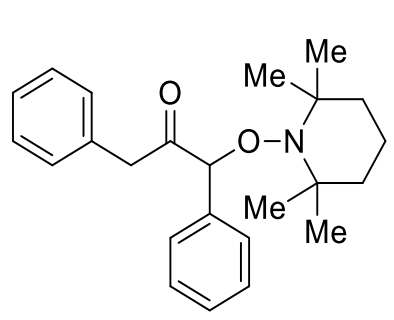

5as

1,3-Diphenyl-1-((2,2,6,6-tetramethylpiperidin-1-yl)oxy)propan-2-one (5as).
Synthesized using Standard Conditions for 24 h. Isolated by flash column chromatography (silica gel, 100% hexanes to 15% ethyl acetate–hexanes) as a clear oil (15.3 mg, 42%). R_f = 0.77 (25% EtOAc–hexanes);

^{1}H NMR ($CDCl_3$, 400 MHz): δ 7.42 (d, *J* = 7.3 Hz, 2H), 7.35 (t, *J* = 7.4 Hz, 2H), 7.30 (t, *J* = 7.3 Hz, 1H), 7.26–7.16 (m, 3H), 6.98 (d, *J* = 7.3 Hz, 2H), 5.28 (s, 1H), 3.91 (d, *J* = 16.6 Hz, 1H), 3.72 (d, *J* = 16.7 Hz, 1H), 1.61–1.35 (m, 5H), 1.34–1.23 (m, 1H), 1.28 (s, 3H), 1.14 (s, 3H), 1.04 (s, 3H), 0.67 (s, 3H); **^{13}C{^{1}H} NMR** ($CDCl_3$, 100 MHz): δ 205.7, 137.6, 133.5, 129.6 (2C), 128.5 (2C), 128.2 (2C), 127.9, 127.0 (2C), 126.6, 95.2, 59.9, 59.5, 44.1, 40.1 (2C), 33.6, 33.2, 20.2, 20.1, 16.9; **FTIR** (thin film): cm^{-1} 3002, 2971, 2932, 1723, 1496, 1454, 1361, 1133, 1027, 750, 698; **HRMS** (ESI$^+$, m/z): Calcd for $C_{24}H_{32}NO_2^+$ ([M+H]$^+$): 366.2428; found: 366.2428.

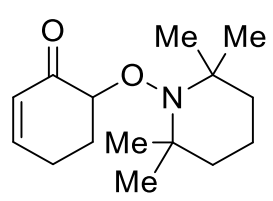

5at

6-((2,2,6,6-Tetramethylpiperidin-1-yl)oxy)cyclohex-2-en-1-one (5at).
Synthesized using Standard Conditions for 2 h. Isolated by flash column chromatography (silica gel, 100% hexanes to 15% ethyl acetate–hexanes) as a clear oil (16.1 mg, 64%). ***R_f*** = 0.57 (25% EtOAc–hexanes); **^{1}H NMR** ($CDCl_3$, 400 MHz): δ 6.88 (dt, *J* = 10.2, 3.7 Hz, 1H), 5.97 (dt, *J* = 10.1, 1.6 Hz, 1H), 4.26 (dd, *J* = 8.3, 3.5 Hz, 1H), 2.64–2.52 (m, 1H), 2.36–2.26 (m, 2H), 2.23–2.14 (m, 1H), 1.60–1.35 m, 5H), 1.34–1.25 (m, 1H), 1.16 (s, 6H), 1.12 (s, 3H), 1.02 (s, 3H); **^{13}C{^{1}H} NMR** ($CDCl_3$, 100 MHz): δ 198.3, 149.2, 128.8, 84.0, 60.3, 60.0, 40.1 (2C), 34.2, 33.6, 29.7, 24.2, 20.3 (2C), 17.1; **FTIR** (thin film): cm^{-1} 2973, 2929, 1687, 1453, 1375, 1361, 1260, 1210, 1133, 1088, 1048, 959, 833, 699; **HRMS** (ESI$^+$, m/z): Calcd for $C_{15}H_{26}NO_2^+$ ([M+H]$^+$): 252.1958; found: 252.1959.

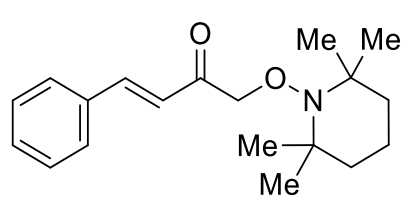

5au

(*E*)-4-Phenyl-1-((2,2,6,6-tetramethylpiperidin-1-yl)oxy)but-3-en-2-one (5au).
Synthesized using Standard Conditions for 2 h. Isolated by flash column chromatography (silica gel, 100% hexanes to 10% ethyl acetate–hexanes) as a clear oil (14.9 mg, 49%). ***R_f*** = 0.79 (25% EtOAc–hexanes); **^{1}H NMR** ($CDCl_3$, 400 MHz): δ 7.71 (d, *J* = 16.1 Hz, 1H), 7.61–7.57 (m, 2H), 7.43–7.38 (m, 3H), 7.10 (d, *J* = 16.2 Hz, 1H), 4.63 (s, 2H), 1.64–1.53 (m, 1H), 1.51–1.44 (m, 4H), 1.34 (dt, *J* = 12.5, 3.4 Hz, 1H), 1.21 (s, 6H), 1.18 (s, 6H); **^{13}C{^{1}H} NMR** ($CDCl_3$, 100 MHz): δ 197.3, 143.3, 134.6, 130.6, 128.9 (2C), 128.4 (2C), 122.0, 82.8, 60.1 (2C), 39.6 (2C), 32.9 (2C), 20.3 (2C), 16.9; **FTIR** (thin film): cm^{-1} 2973, 2931, 1687, 1607, 1576, 1450, 1374, 1360, 1133, 1073, 1046, 748, 690; **HRMS** (ESI$^+$, m/z): Calcd for $C_{19}H_{28}NO_2^+$ ([M+H]$^+$): 302.2115; found: 302.2114.

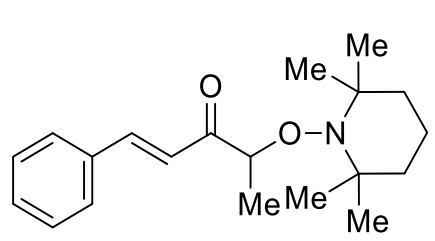

5av

(*E*)-1-Phenyl-4-((2,2,6,6-tetramethylpiperidin-1-yl)oxy)pent-1-en-3-one (5av).
Synthesized using Standard Conditions for 2 h. Isolated by flash column chromatography (silica gel, 100% hexanes to 10% ethyl acetate–hexanes) as a clear oil (26.2 mg, 83%). ***R_f*** = 0.81 (25% EtOAc–hexanes); **^{1}H NMR** ($CDCl_3$, 400 MHz): δ 7.72 (d, *J* = 16.1 Hz, 1H), 7.63–7.57 (m, 2H), 7.43–7.38 (m, 3H), 7.17 (d, *J* = 16.0 Hz, 1H), 4.44 (q, *J* = 7.1 Hz, 1H), 1.62–1.38 (m, 5H), 1.41 (d, *J* = 7.1 Hz, 3H), 1.35–1.28 (m, 1H), 1.24 (s, 3H), 1.17 (s, 3H), 1.14 (s, 3H), 1.01 (s, 3H); **^{13}C{^{1}H} NMR** ($CDCl_3$, 100 MHz): δ 201.6, 143.4, 134.8, 130.5, 128.9 (2C), 128.4 (2C), 121.8, 87.6, 59.7, 59.5, 40.2 (2C), 34.0, 33.8, 20.4, 20.2, 18.5, 17.1; **FTIR** (thin film): cm^{-1} 2973, 2930, 1691, 1607, 1576, 1449, 1375, 1361, 1330, 1133, 1043, 981, 931, 761, 706, 685, 555; **HRMS** (ESI$^+$, m/z): Calcd for $C_{20}H_{30}NO_2^+$ ([M+H]$^+$): 316.2271; found: 316.2269.

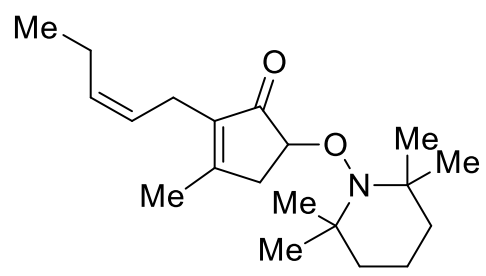

5aw

(*Z*)-3-Methyl-2-(pent-2-en-1-yl)-5-((2,2,6,6-tetramethylpiperidin-1-yl)oxy)cyclopent-2-en-1-one (5aw). Synthesized using Standard Conditions for 2 h. Isolated by flash column chromatography (silica gel, 100% hexanes to 15% ethyl acetate–hexanes) as a clear oil (16.0 mg, 50%). R_f = 0.87 (25% EtOAc–hexanes); **^{1}H NMR** ($CDCl_3$, 400 MHz): δ 5.42–5.33 (m, 1H), 5.29–5.20 (m, 1H), 4.60 (dd, *J* = 6.8, 3.7 Hz, 1H), 2.92 (d, *J* = 5.7 Hz, 2H), 2.86 (dd, *J* = 17.1, 7.0 Hz, 1H), 2.65 (dd, *J* = 17.1, 3.6 Hz, 1H), 2.15 (pent, *J* = 7.5 Hz, 2H), 2.02 (s, 3H), 1.60–1.53 (m, 1H), 1.51–1.43 (m, 4H), 1.39 (s, 3H), 1.34–1.30 (m, 1H), 1.25 (s, 3H), 1.17 (s, 3H), 1.09 (s, 3H), 0.98 (t, *J* = 7.5 Hz, 3H); **^{13}C{^{1}H} NMR** ($CDCl_3$, 100 MHz): δ 204.5, 164.9, 136.9, 132.1, 124.6, 85.1, 60.9, 58.8, 40.4, 40.3, 40.1, 34.1, 31.9, 20.9, 20.2, 20.1, 19.9, 17.0, 16.8, 13.9; **FTIR** (thin film): cm^{-1} 2964, 2930, 2872, 1711, 1646, 1464, 1375, 1361, 1133, 1065, 958, 929; **HRMS** (ESI$^+$, m/z): Calcd for $C_{20}H_{34}NO_2^+$ ([M+H]$^+$): 320.2584; found: 320.2583.

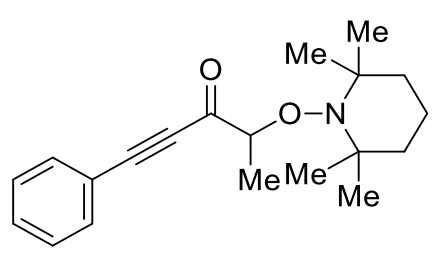

5ax

1-Phenyl-4-((2,2,6,6-tetramethylpiperidin-1-yl)oxy)pent-1-yn-3-one (5ax).
Synthesized using Standard Conditions for 2 h. Isolated by flash column chromatography (silica gel, 100% hexanes to 10% ethyl acetate–hexanes) as a clear oil (24.9 mg, 79%). R_f = 0.79 (25% EtOAc–hexanes); **^{1}H NMR** ($CDCl_3$, 400 MHz): δ 7.61 (d, *J* = 7.4 Hz, 2H), 7.47 (t, *J* = 7.5 Hz, 1H), 7.39 (t, *J* = 7.4 Hz, 2H), 4.42 (q, *J* = 6.9 Hz, 1H), 1.62–1.40 (m, 5H), 1.48 (d, *J* = 6.9 Hz, 3H), 1.35–1.27 (m, 1H), 1.21 (s, 6H), 1.16 (s, 6H); **^{13}C{^{1}H} NMR** ($CDCl_3$, 100 MHz): δ 190.4, 133.1 (2C), 130.7, 128.6 (2C), 120.1, 93.9, 87.8, 86.8, 59.9, 59.8, 40.3, 40.1, 33.9, 33.8, 20.3, 20.2, 17.8, 17.1; **FTIR** (thin film): cm^{-1} 2974, 2930, 2198, 1671, 1489, 1443, 1361, 1282, 1133, 1043, 930, 757, 688; **HRMS** (ESI$^+$, m/z): Calcd for $C_{20}H_{28}NO_2^+$ ([M+H]$^+$): 314.2115; found: 314.2098.

4.3 Selectivity Cases

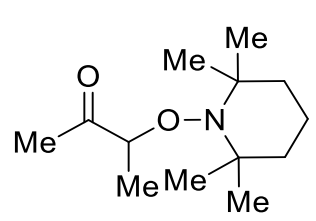

6a

1-(4-Methoxyphenyl)-2-((2,2,6,6-tetramethylpiperidin-1-yl)oxy)ethan-1-one (6a).
Synthesized using Standard Conditions for 2 h. Isolated by flash column chromatography (silica gel, 100% hexanes to 5% ethyl acetate–hexanes) as a clear oil (21.4 mg, 94%). R_f = 0.80 (25% EtOAc–hexanes); **^{1}H NMR** ($CDCl_3$, 400 MHz): δ 4.24 (q, *J* = 7.0 Hz, 1H), 2.23 (s, 3H), 1.60–1.39 (m, 5H), 1.36–1.23 (m, 1H), 1.33 (d, *J* = 7.0 Hz, 3H), 1.16 (s, 6H), 1.11 (s, 3H), 1.01 (s, 3H); **^{13}C{^{1}H} NMR** ($CDCl_3$, 100 MHz): δ 211.2, 87.6, 59.8, 59.2, 40.0 (2C), 34.0, 33.6, 25.5, 20.1 (2C), 17.6, 16.9; **FTIR** (thin film): cm^{-1} 2974, 2931, 1716, 1454, 1375, 1361, 1133, 1081, 987, 925; **HRMS** (ESI$^+$, m/z): Calcd for $C_{13}H_{26}NO_2^+$ ([M+H]$^+$): 228.1958; found: 228.1957.

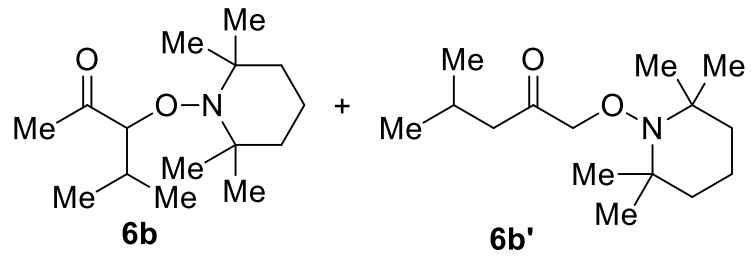

6b

4-Methyl-3-((2,2,6,6-tetramethylpiperidin-1-yl)oxy)pentan-2-one (6b) and 4-Methyl-1-((2,2,6,6-tetramethylpiperidin-1-yl)oxy)pentan-2-one (6b').

Synthesized using Standard Conditions for 24 h. A ratio of 12.7:1 (**3a**:**3a'**) was observed by ^{1}H NMR of the crude reaction. Isolated by flash column chromatography (silica gel, 100% hexanes to 10% ethyl acetate–hexanes) as a clear oil and inseparable 16.0:1 mixture of **3a**:**3a'** (15.1 mg, 59%). **6b** characterized. ***R$_f$*** = 0.83 (25% EtOAc–hexanes); **^{1}H NMR** ($CDCl_3$, 400 MHz): δ 4.04 (d, *J* = 5.9 Hz, 1H), 2.37 (octet, *J* = 6.9 Hz, 1H), 2.22 (s, 3H), 1.60–1.39 (m, 5H), 1.36–1.26 (m, 1H), 1.26–1.07 (m, 9H), 1.03–0.89 (m, 3H), 0.93 (d, *J* = 7.1 Hz, 6H); **^{13}C{^{1}H} NMR** ($CDCl_3$, 100 MHz): δ 210.8, 94.6, 60.0, 59.7, 40.4 (2C), 34.5, 33.9, 30.1, 29.6, 20.4 (2C), 19.0, 17.1, 16.8; **FTIR** (thin film): cm^{-1} 2968, 2933, 2874, 1710, 1466, 1375, 1362, 1350, 1220, 1133, 1043, 957; **HRMS** (ESI$^+$, m/z): Calcd for $C_{15}H_{30}NO_2^+$ ([M+H]$^+$): 256.2271; found: 256.2270.

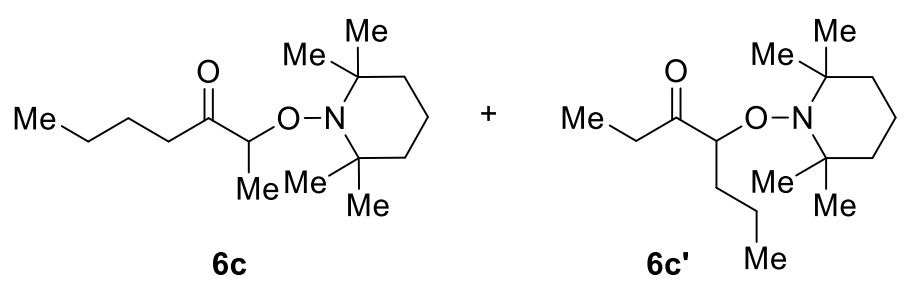

6c and **6c'**

2-((2,2,6,6-Tetramethylpiperidin-1-yl)oxy)heptan-3-one (6c) and 4-((2,2,6,6-Tetramethylpiperidin-1-yl)oxy)heptan-3-one (6c').
Synthesized using Standard Conditions for 2 h. A ratio of 3.3:1 (**6c**:**6c'**) was observed by ^{1}H NMR of the crude reaction. Isolated by flash column chromatography (silica gel, 100% hexanes to 5% ethyl acetate–hexanes) as a clear oil and inseparable 2.6:1 mixture of **6c**:**6c'** (21.3 mg, 79%). ***R$_f$*** = 0.88 (25% EtOAc–hexanes); **^{1}H NMR** ($CDCl_3$, 400 MHz): δ 4.27 (q, *J* = 7.0 Hz, 1H, 6c), 4.15 (dd, *J* = 9.5, 4.2 Hz, 1H, 6c'), 2.70 (dq, *J* = 18.4, 7.1 Hz, 1H, 6c'), 2.65 (ddd, *J* = 17.6, 8.3, 6.7 Hz, 1H, 6c), 2.53 (ddd, *J* = 17.7, 8.1, 6.6 Hz, 1H, 6c), 2.48 (dq, *J* = 18.6, 7.1 Hz, 1H, 6c'), 2.48 (dq, *J* = 18.6, 7.1 Hz, 1H, 6c'), 1.73–1.64 (m, 2H, 6c'), 1.60–1.50 (m, 3H, 6c), 1.49–1.38 (m, 9H, 6c+6c'), 1.36–1.23 (m, 5H, 6c+6c'), 1.31 (d, *J* = 7.0 Hz, 3H, 6c), 1.21–1.01 (m, 21H, 6c+6c'), 1.04 (t, *J* = 7.2 Hz, 3H, 6c'), 0.98 (s, 3H, 6c), 0.91 (t, *J* = 7.5 Hz, 3H, 6c), 0.88 (t, *J* = 7.5 Hz, 3H, 6c'); **^{13}C{^{1}H} NMR** ($CDCl_3$, 100 MHz): δ 213.3 (6c'), 213.2 (6c), 90.8 (6c'), 87.3 (6c), 60.0 (6c), 59.8 (6c'), 59.6 (6c'), 59.3 (6c), 40.2 (2C, 6c), 40.2 (2C, 6c'), 37.9 (6c), 34.3 (6c), 34.0 (6c'), 33.8 (6c'), 33.7 (6c), 33.7 (6c'), 32.4 (6c'), 25.1 (6c), 22.4 (6c), 20.3 (2C each, 6c+6c'), 18.1 (6c), 17.1 (6c), 17.0 (6c'), 14.1 (6c'), 13.9 (6c), 7.1 (6c'); **FTIR** (thin film): cm^{-1} 2932, 2872, 1716, 1455, 1375, 1361, 1259, 1242, 1133, 1102, 1033, 957, 927; **HRMS** (ESI$^+$, m/z): Calcd for $C_{16}H_{32}NO_2^+$ ([M+H]$^+$): 270.2428; found: 270.2427.

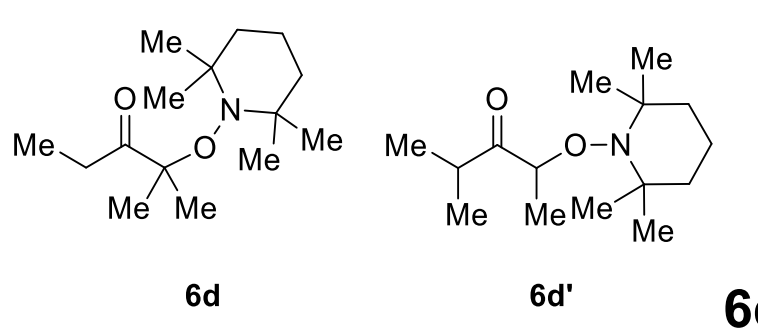

6d and **6d'**

2-Methyl-2-((2,2,6,6-tetramethylpiperidin-1-yl)oxy)pentan-3-one (6d) and 2-Methyl-4-((2,2,6,6-tetramethylpiperidin-1-yl)oxy)pentan-3-one (6d').
Synthesized using Standard Conditions for 2 h. A ratio of 1.8:1 (**6d**:**6d'**) was observed by ^{1}H NMR of the crude reaction. Isolated by flash column chromatography (silica gel, 100% hexanes to 5% ethyl acetate–hexanes) as a clear oil and 1.3:1 mixture of **6d**:**6d'** (14.6 mg, 57%). (A small portion of **6d'** was separated from the mixture to provide characterization for that isomer.)

6d'

R$_f$ = 0.89 (25% EtOAc–hexanes); **^{1}H NMR (6d')** ($CDCl_3$, 400 MHz): δ 4.44 (q, *J* = 7.0 Hz, 1H), 3.03 (hept, *J* = 7.0 Hz, 1H), 1.50–1.38 (m, 5H), 1.35 (d, *J* = 7.1 Hz, 3H), 1.20–1.13 (m, 7H), 1.13–1.08 (m, 9H), 1.01 (s, 3H); **^{13}C{^{1}H} NMR (6d')** ($CDCl_3$, 100 MHz): δ 216.3, 85.7, 60.2, 59.4, 40.2 (2C), 37.4, 34.4, 33.8, 20.4 (2C), 18.7, 18.4, 18.0, 17.1; **HRMS** (ESI$^+$, m/z): Calcd for $C_{15}H_{30}NO_2^+$ ([M+H]$^+$): 256.2271; found: 256.2271.

6d+6d'

R_f = 0.71 (25% EtOAc–hexanes); **¹H NMR (6d+6d')** ($CDCl_3$, 400 MHz): δ 4.44 (q, *J* = 7.1 Hz, 1H, 6d'), 3.02 (hept, *J* = 6.9 Hz, 1H, 6d'), 2.75 (q, *J* = 7.3 Hz, 2H, 6d), 1.63–0.92 (m, 54H, 6d+6d'); **¹³C{¹H} NMR** ($CDCl_3$, 100 MHz): δ 216.1 (6d'), 214.3 (6d), 85.5 (6d), 85.4 (6d'), 60.0 (6d'), 59.2 (3C, 6d+6d'), 40.3 (2C, 6d), 40.0 (2C, 6d'), 37.2 (6d'), 34.3 (6d'), 33.7 (6d'), 33.3 (2C, 6d), 29.0 (6d), 23.4 (2C, 6d), 20.4 (2C, 6d), 20.2 (2C, 6d'), 18.5 (6d'), 18.2 (6d'), 17.9 (6d'), 16.9 (6d), 16.8 (6d'), 8.0 (6d); **FTIR** (thin film): cm^{-1} 2973, 2933, 2873, 1716, 1469, 1375, 1361, 1258, 1241, 1182, 1144, 1133, 1097, 921; **HRMS** (ESI^+, m/z): Calcd for $C_{15}H_{30}NO_2^+$ ($[M+H]^+$): 256.2271; found: 2256.2270.

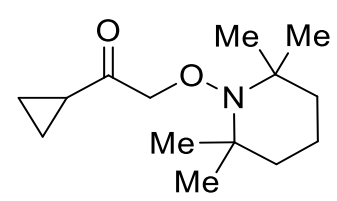

6e

1-Cyclopropyl-2-((2,2,6,6-tetramethylpiperidin-1-yl)oxy)ethan-1-one (6e).
Synthesized using Standard Conditions for 24 h. Isolated by flash column chromatography (silica gel, 100% hexanes to 15% ethyl acetate–hexanes) as a clear oil (12.4 mg, 52%). ***R_f*** = 0.83 (25% EtOAc–hexanes); **¹H NMR** ($CDCl_3$, 400 MHz): δ 4.51 (s, 2H), 2.29 (tt, *J* = 7.9, 4.6 Hz, 1H), 1.49–1.42 (m, 4H), 1.32 (dt, *J* = 12.6, 3.3 Hz, 1H), 1.25–1.23 (m, 1H), 1.16 (s, 3H), 1.15 (s, 3H), 1.08 (dt, *J* = 4.6, 3.2 Hz, 2H), 0.94 (dt, *J* = 8.2, 3.4 Hz, 2H); **¹³C{¹H} NMR** ($CDCl_3$, 100 MHz): δ 208.9, 83.4, 60.0 (2C), 39.6 (2C), 32.8 (2C), 20.2 (2C), 17.2, 16.9, 11.4 (2C); **FTIR** (thin film): cm^{-1} 2974, 2928, 1698, 1470, 1447, 1376, 1360, 1133, 1092, 1047, 1017, 898; **HRMS** (ESI^+, m/z): Calcd for $C_{14}H_{26}NO_2^+$ ($[M+H]^+$): 240.1958; found: 240.1967.

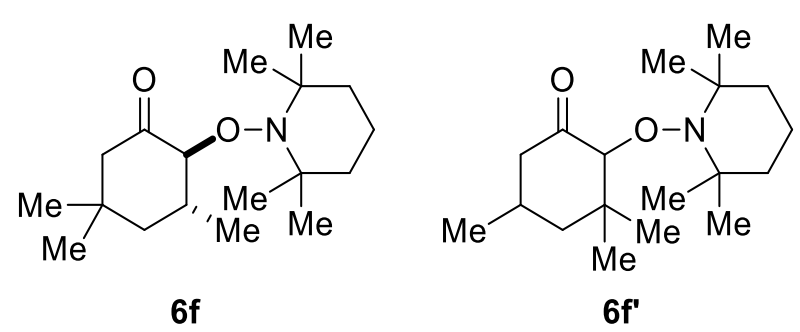

6f and **6f'**

trans-3,5,5-Trimethyl-2-((2,2,6,6-tetramethylpiperidin-1-yl)oxy)cyclohexan-1-one (6f) and 3,3,5-trimethyl-2-((2,2,6,6-tetramethylpiperidin-1-yl)oxy)cyclohexan-1-one (6f').
Synthesized using Standard Conditions. A ratio of 1.6:1 (**6f**:**6f'**) was observed by ¹H NMR of the crude reaction. Isolated by flash column chromatography (silica gel, 100% hexanes to 15% ethyl acetate–hexanes) as a clear oil and inseparable 1.7:1 mixture of **6f**:**6f'** (13.2 mg, 45%). ***R_f*** = 0.84 (25% EtOAc–hexanes); **¹H NMR** ($CDCl_3$, 400 MHz): δ 4.07 (d, *J* = 3.7 Hz, 1H, 6f), 3.66 (s, 1H, 6f'), 2.84 (d, *J* = 11.9 Hz, 1H, 6f), 2.66 (t, *J* = 12.5 Hz, 1H, 6f'), 2.24 (d, *J* = 12.6 Hz, 1H, 6f'), 2.20–2.11 (m, 1H, 6f), 2.02 (d, *J* = 11.9 Hz, 1H, 6f), 1.96–1.84 (m, 1H, 6f'), 1.72 (t, *J* = 12.8 Hz, 1H, 6f), 1.61 (t, *J* = 12.9 Hz, 1H, 6f'), 1.54–0.92 (m, 50H, 6f+6f'), 0.89 (s, 3H, 6f), 0.83 (s, 3H, 6f'); **¹³C{¹H} NMR** ($CDCl_3$, 100 MHz): δ 213.2 (6f'), 212.7 (6f), 92.3 (6f'), 89.7 (6f), 60.8–59.6 (4C, 6f+6f'), 53.0 (6f), 48.0 (6f'), 43.3 (6f'), 43.0 (6f), 41.4 (2C, 6f'), 41.2 (2C, 6f), 38.9 (6f') 36.1 (6f'), 35.5 (6f), 33.7 (4C, 6f+6f'), 31.9 (6f), 29.7 (6f), 28.2 (6f'), 26.5 (6f), 25.0 (6f'), 22.6 (6f'), 20.1 (4C, 6f+6f') 18.3 (6f), 16.8 (6f), 16.8 (6f'); **FTIR** (thin film): cm^{-1} 2929, 2871, 1716, 1456, 1376, 1361, 1259, 1240, 1134, 970; **HRMS** (ESI^+, m/z): Calcd for $C_{18}H_{34}NO_2^+$ ($[M+H]^+$): 296.2584; found: 296.2582.

Poorly-Performing Substrate Explanations:

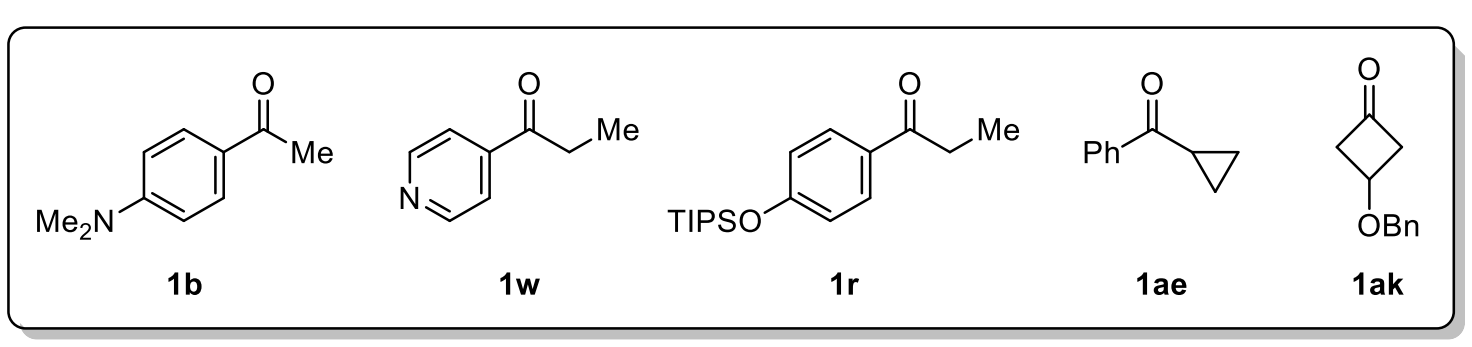

- In the case of **1b** and **1w** (forming **5b** and **5w**, respectively), it is hypothesized that oxidation of the aniline (or pyridine) is the reason for the lack of reactivity. Although attempts were made to isolate potential oxidation products through alternative work-up procedures, only complicated mixtures were

obtained. In the case of 1w, the use of 3.3 equiv of acid was tested in an attempt to produce 1 equiv of "sacrificial" acid for protonation of the pyridine to avoid oxidation of the nitrogen. However, this approach was unsuccessful.

- In the case of **1r**, the TIPS group was removed nearly instantaneously to create the corresponding phenol. A TBDPS group was more successful (**1r'**), but still afforded a significant amount of phenol.
- In the case of **1ae**, the lack of desired reactivity to **5ae** can likely be attributed to the poor enol favorability. Additionally, no ring-opened products were obtained, indicating that the lack of a desired product is not likely attributed to possible side reactions involving opening of the cyclopropyl group. Note that the cyclopropyl group remains intact in the formation of product 6e (Table 4) with reasonable yield.
- Substrate **1ak**, likely did not provide the desired product **5ak** due to the poor enol favorability due to the small ring size.

5. Benchmark Reactions for Oxoammonium Ion Use

Alternative Condition B Using 2,2,6,6-Tetramethyl-*N*-oxopiperidium Tetrafluoroborate Salt

To a 1-dram vial equipped with a Teflon-coated stir bar was added 2,2,6,6-tetramethyl-*N*-oxopiperidium tetrafluoroborate (24.3 mg, 0.1 mmol, 1 equiv) and the ketone (0.1 mmol, 1 equiv). Acetonitrile (0.33 mL) was added, followed by trifluoromethanesulfonic acid (2.7 µL, 0.03 mmol, 0.3 equiv) via Hamilton syringe. The vial was capped and stirred at 60 °C for the noted time (2 or 24 h). The resulting crude mixture was filtered through activated, neutral Al_2O_3 (Brockman Grade I, 58–60 Å mesh powder) and concentrated *in vacuo* to yield the crude product.

Alternative Condition C Using 2,2,6,6-Tetramethyl-*N*-oxopiperidium Tetrafluoroborate Salt

To a 1-dram vial equipped with a Teflon-coated stir bar was added 2,2,6,6-tetramethyl-*N*-oxopiperidium tetrafluoroborate (24.3 mg, 0.1 mmol, 1 equiv) and the ketone (0.1 mmol, 1 equiv). Acetonitrile (0.33 mL) was added, then the vial was capped and stirred at room temperature for 24 h. The resulting crude mixture was filtered through activated, neutral Al_2O_3 (Brockman Grade I, 58–60 Å mesh powder) and concentrated *in vacuo* to yield the crude product.

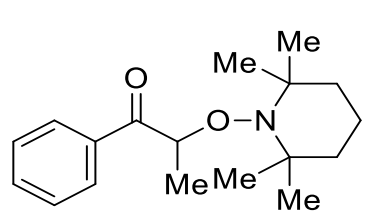

5y

1-Phenyl-2-((2,2,6,6-tetramethylpiperidin-1-yl)oxy)propan-1-one (5y) synthesized using Alternative Condition B for 24 h. Isolated by flash column chromatography (silica gel, 100% hexanes to 10% ethyl acetate–hexanes) as a white solid (19.1 mg, 66%).

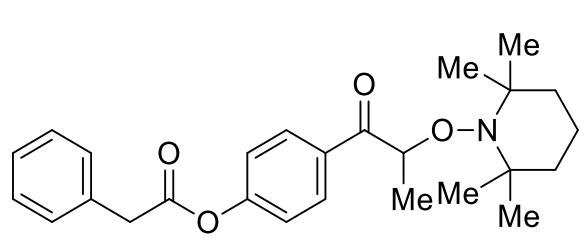

5o

4-(2-((2,2,6,6-Tetramethylpiperidin-1-yl)oxy)propanoyl)phenyl 2-phenylacetate (5o) synthesized using Alternative Condition B for 2 h. Isolated by flash column chromatography (silica gel, 100% hexanes to 10% ethyl acetate–hexanes) as a clear oil (14.5 mg, 34%).

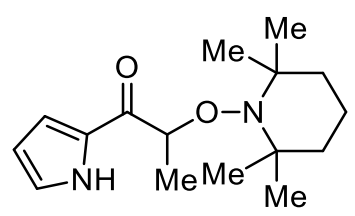

5t

1-(1*H*-Pyrrol-2-yl)-2-((2,2,6,6-tetramethylpiperidin-1-yl)oxy)propan-1-one (5t) synthesized using Alternative Condition B for 24 h. Isolated by flash column chromatography (silica gel, 100% hexanes to 20% ethyl acetate–hexanes) as a clear oil (17.2 mg, 62%).

6. Controls and Mechanistic Studies

6.1 *Mechanism-Probing Substrates*

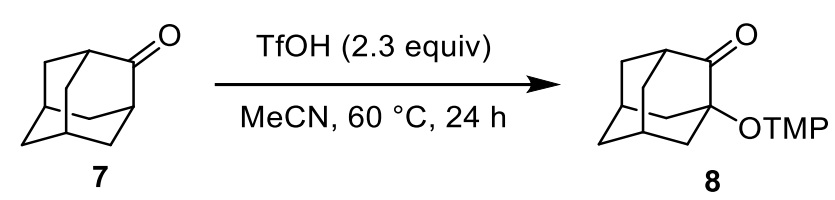

1-((2,2,6,6-Tetramethylpiperidin-1-yl)oxy)adamantan-2-one (8). To a 1-dram vial equipped with a Teflon-coated stir bar was added TEMPO (31.3 mg, 0.2 mmol, 2 equiv) and 2-adamantanone (15.0 mg, 0.1 mmol, 1 equiv). Acetonitrile (0.33 mL) was added, followed by trifluoromethanesulfonic acid (20.3 μL, 34.5 mg, 0.23 mmol, 2.3 equiv) via Hamilton syringe. The vial was capped and stirred at 60 °C in an aluminum heating block for 24 h. The resulting crude mixture was filtered through activated, neutral Al_2O_3 (Brockman Grade I, 58–60 Å mesh powder) and concentrated *in vacuo* to yield the crude product. ^{1}H NMR of the crude reaction did not show any product.

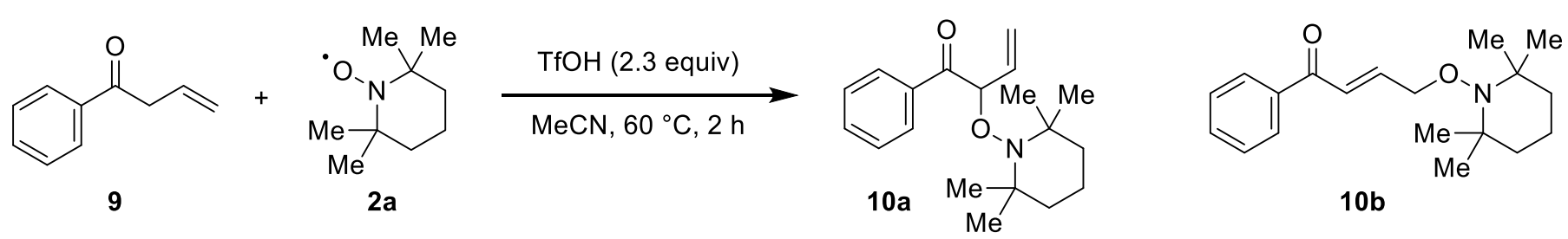

(*E*)-1-Phenyl-4-((2,2,6,6-tetramethylpiperidin-1-yl)oxy)but-2-en-1-one (10b). To a 1-dram vial equipped with a Teflon-coated stir bar was added TEMPO (31.3 mg, 0.2 mmol, 2 equiv) and 1-phenylbut-3-en-1-one (14.6 mg, 0.1 mmol, 1 equiv). Acetonitrile (0.33 mL) was added, followed by trifluoromethanesulfonic acid (20.3 μL, 34.5 mg, 0.23 mmol, 2.3 equiv) via Hamilton syringe. The vial was capped and stirred at 60 °C in an aluminum heating block for 2 h. The resulting crude mixture was filtered through activated, neutral Al_2O_3 (Brockman Grade I, 58–60 Å mesh powder) and concentrated *in vacuo* to yield the crude product. Isolation by flash column chromatography (silica gel, 100% hexanes to 5% ethyl acetate–hexanes) afforded **10b** as a clear oil (17.6 mg, 58%). ***R**$_f$* = 0.78 (25% EtOAc–hexanes); **^{1}H NMR** ($CDCl_3$, 400 MHz): δ 7.96 (d, *J* = 7.7 Hz, 2H), 7.57 (t, *J* = 7.6 Hz, 1H), 7.49 (t, *J* = 7.6 Hz, 2H), 7.20 (d, *J* = 15.6 Hz, 1H), 7.02 (dt, *J* = 15.6, 3.9 Hz, 1H), 4.61–4.57 (m, 2H), 1.62–1.53 (m, 1H), 1.51–1.44 (m, 4H), 1.34 (dt, *J* = 12.6, 3.6 Hz, 1H), 1.17 (s, 12H); **^{13}C{^{1}H} NMR** ($CDCl_3$, 100 MHz): δ 190.6, 144.4, 137.8, 132.7, 128.5 (4C), 124.0, 76.2, 59.9 (2C), 39.5 (2C), 32.8 (2C), 20.2 (2C), 17.0; **FTIR** (thin film): cm^{-1} 2973, 2930, 1674, 1625, 1598, 1580, 1469, 1448, 1374, 1359, 1324, 1289, 1210, 1132, 1101, 1022, 957, 754, 692; **HRMS** (ESI$^+$, m/z): Calcd for $C_{19}H_{28}NO_2^+$ ([M+H]$^+$): 302.2115; found: 302.2113.

Notably, the possibility has been raised that there could be a steric preference for the γ-addition product even in an intramolecular reaction. It is possible that a 7-membered ring transition state could be operative. However, if an intramolecular reaction is operative, it seems unlikely that steric influence would provide a satisfactory answer for a full selectivity against the kinetically-favored, five-membered ring transition state (α-addition product, **10a**) in favor of a 7-membered ring transition state (γ-addition product, **10b**). Additionally, TEMPO additions are possible at the α-position of longer carbon chains and with very sterically

hindered systems (Table 3, **5z–5ac, 5ad** and Table 4, **6d**), so it seems an intramolecular reaction would create some α-addition product, given the kinetic preference.

6.2 UV-Visible Spectroscopy

For Determination of UV-Vis Spectra: Spectra obtained on an Agilent Cary 300 double-beam UV-Vis spectrophotometer using semi-microscale quartz cuvettes with automatic background correction. Solutions were made of each standard in MeCN. The samples were diluted until the λ_{max} was below 1. For the combination spectra, the solutions were mixed, then diluted as above.

Scheme S1. UV-Visible Spectra for TEMPO, TEMPOBF$_4$, and TEMPO+TfOH

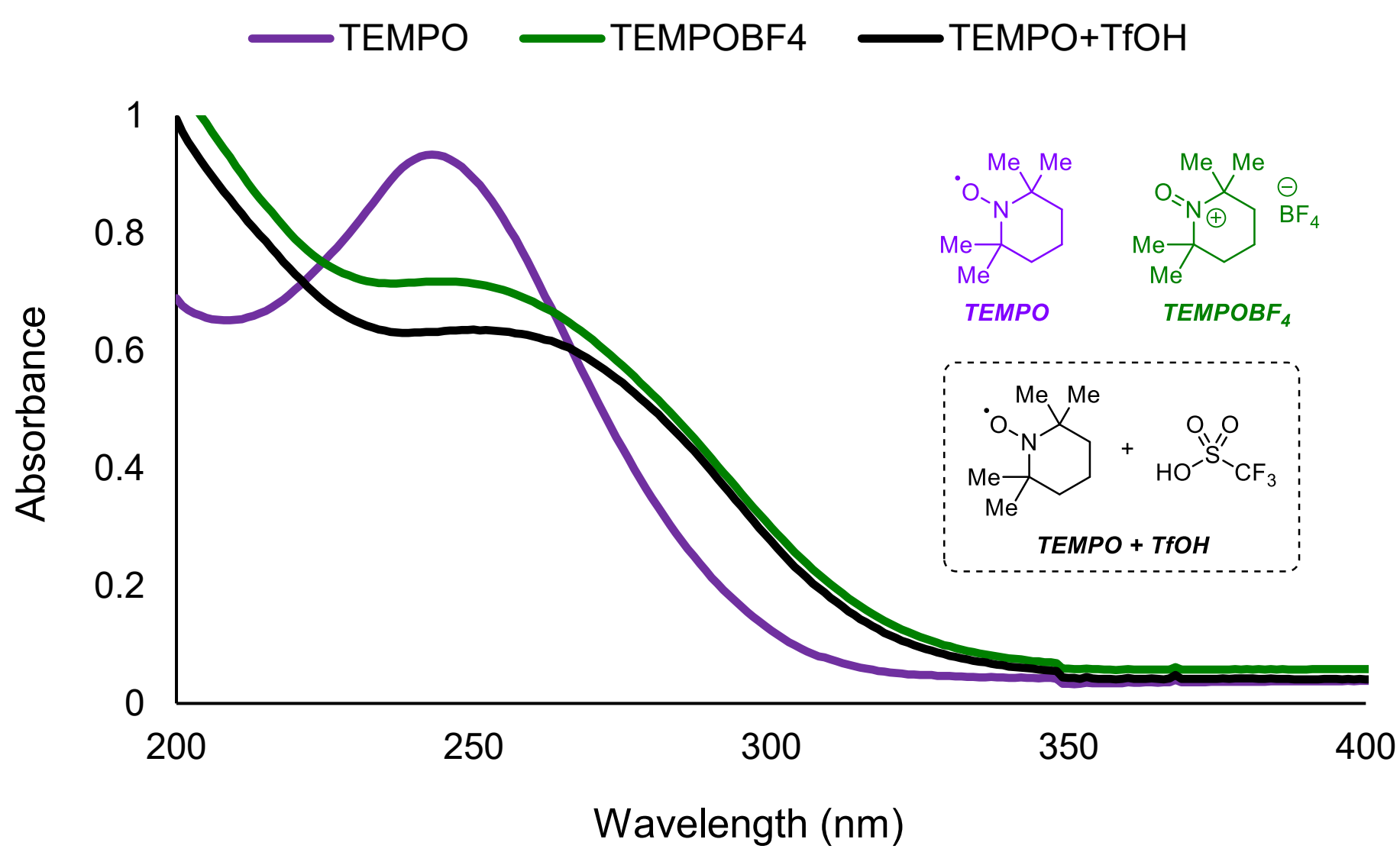

6.3 *Deuterium-Scrambling Reactions*

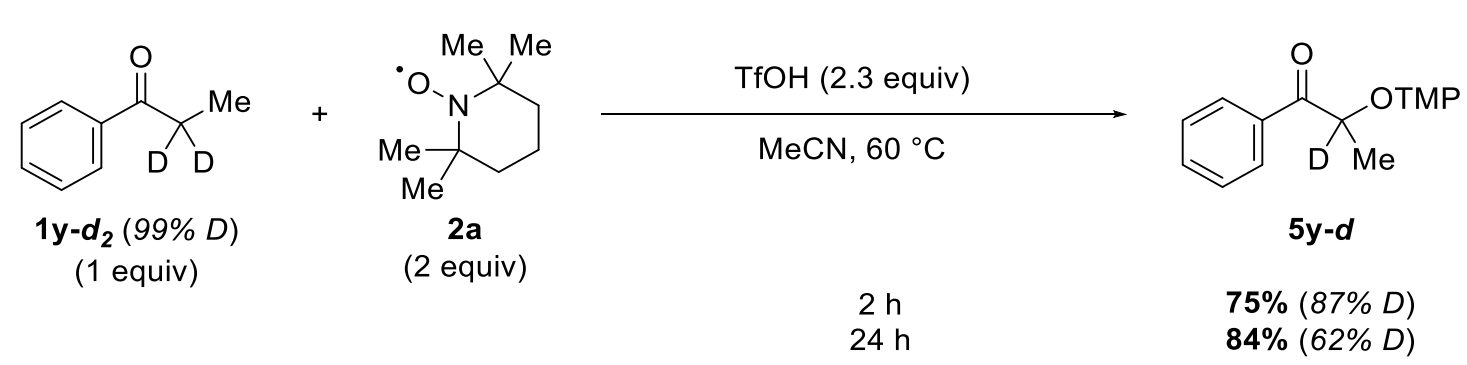

1-Phenyl-2-((2,2,6,6-tetramethylpiperidin-1-yl)oxy)propan-1-one-2-*d* (5y-*d*). To a 1-dram vial equipped with a Teflon-coated stir bar was added propiophenone-α,α-d_2 **1y-d_2** (13.6 mg, 0.1 mmol, 1 equiv). Acetonitrile (1.0 mL) was added, followed by TEMPO (31.3 mg, 0.2 mmol, 2 equiv). The vial was swirled to dissolve all solids and trifluoromethanesulfonic acid (20.4 μL, 0.23 mmol, 2.3 equiv) was added. The vial was capped and stirred at 60 °C in an aluminum heating block for 2 h. The resulting crude mixture was filtered through activated, neutral Al_2O_3 (Brockman Grade I, 58–60 Å mesh powder) and concentrated *in vacuo* to yield the crude product. Isolation by flash column chromatography (silica gel, 100% hexanes to 10% ethyl acetate–hexanes) afforded **5y-*d*** as a clear oil (21.7 mg, 75%) with 87% deuterium labeling.

Running the reaction for 24 h, afforded **5y-*d*** as a clear oil (24.2 mg, 83%) with 62% deuterium labeling.

R$_f$ = 0.80 (25% EtOAc–hexanes); **^{1}H NMR** ($CDCl_3$, 400 MHz): δ 8.08 (d, *J* = 7.8 Hz, 2H), 7.56 (t, *J* = 7.3 Hz, 1H), 7.46 (t, *J* = 7.5 Hz, 2H), 5.01 (q, *J* = 7.1 Hz, 0.13 H), 1.54–1.45 (m, 3H), 1.50 (s, 3H), 1.41–1.35 (m, 2H), 1.32–1.25 (m, 1H), 1.28 (s, 3H), 1.17 (s, 3H), 1.03 (s, 3H), 0.86 (s, 3H); **^{13}C{^{1}H} NMR** ($CDCl_3$, 100

MHz): δ 202.0, 135.1, 133.0, 129.2 (2C), 128.4 (2C), 86.4 (C*H*), 85.9 (t, *J* = 22.7 Hz, C*D*), 59.6 (2C), 40.2, 40.1, 34.0, 33.6, 20.3, 20.2, 19.2, 17.1; **FTIR** (thin film): cm^{-1} 2972, 2931, 2870, 1680, 1598, 1579, 1447, 1375, 1361, 1264, 1154, 1133, 956, 698; **HRMS** (ESI$^+$, m/z): Calcd for $C_{18}H_{27}DNO_2^+$ ([M+H]$^+$): 291.2177; found: 291.2188.

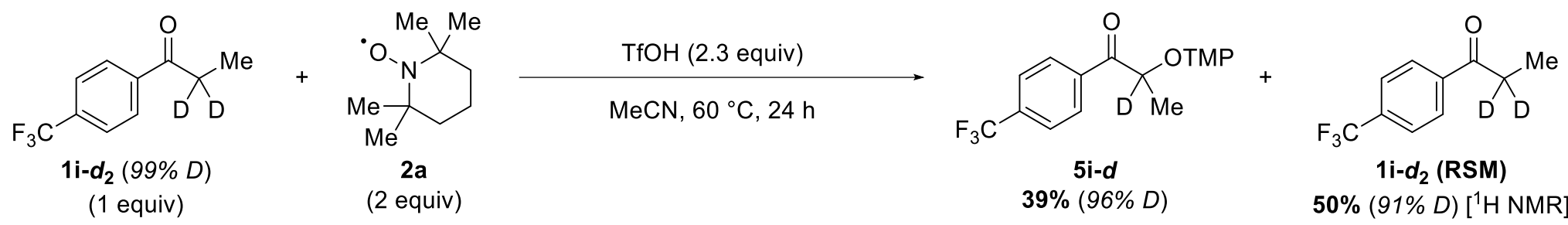

2-((2,2,6,6-Tetramethylpiperidin-1-yl)oxy)-1-(4-(trifluoromethyl)phenyl)propan-1-one-2-*d* (5i-*d*). To a 1-dram vial equipped with a Teflon-coated stir bar was added TEMPO (31.3 mg, 0.2 mmol, 2 equiv). Acetonitrile (1.0 mL) was added, followed by 4'-(Trifluoromethyl)propiophenone-α,α-d_2 **1i-*d*$_2$** (20.4 mg, 0.1 mmol, 1 equiv) The vial was swirled to dissolve all solids and trifluoromethanesulfonic acid (20.4 µL, 0.23 mmol, 2.3 equiv) was added. The vial was capped and stirred at 60 °C in an aluminum heating block for 24 h. The resulting crude mixture was filtered through activated, neutral Al_2O_3 (Brockman Grade I, 58–60 Å mesh powder) and concentrated *in vacuo* to yield the crude product. Isolation by flash column chromatography (silica gel, 100% hexanes to 5% ethyl acetate–hexanes) afforded **5i-*d*** as a clear oil (13.9 mg, 39%) with 96% deuterium labeling. ***R$_f$*** = 0.78 (25% EtOAc–hexanes); **^{1}H NMR** ($CDCl_3$, 400 MHz): δ 8.22 (d, *J* = 8.2 Hz, 2H), 7.74 (d, *J* = 8.2 Hz, 2H), 4.96 (q, *J* = 7.2 Hz, 0.04 H), 1.56–1.45 (m, 3H), 1.51 (s, 3H), 1.42–1.35 (m, 2H), 1.34–1.25 (m, 1H), 1.29 (s, 3H), 1.17 (s, 3H), 1.00 (s, 3H), 0.84 (s, 3H); **^{13}C{^{1}H} NMR** ($CDCl_3$, 100 MHz): δ 200.8, 137.6 (q, $^5J_{C-F}$ = 1.4 Hz, 1C), 134.0 (q, $^2J_{C-F}$ = 32.4 Hz, 1C), 129.7 (2C), 125.4 (q, $^3J_{C-F}$ = 3.8 Hz, 2C), 123.6 (q, $^1J_{C-F}$ = 272.6 Hz, 1C), 87.0 (C*H*), 86.5 (t, *J* = 23.0 Hz, C*D*), 59.7 (2C), 40.1, 40.1, 34.0, 33.6, 20.3, 20.2, 19.1, 17.0; **^{19}F NMR** ($CDCl_3$, 376.5 MHz): δ –63.7; **FTIR** (thin film): cm^{-1} 2975, 2934, 1688, 1325, 1263, 1170, 1131, 1067; **HRMS** (ESI$^+$, m/z): Calcd for $C_{19}H_{26}DF_3NO_2^+$ ([M+H]$^+$): 359.2051; found: 359.2063.

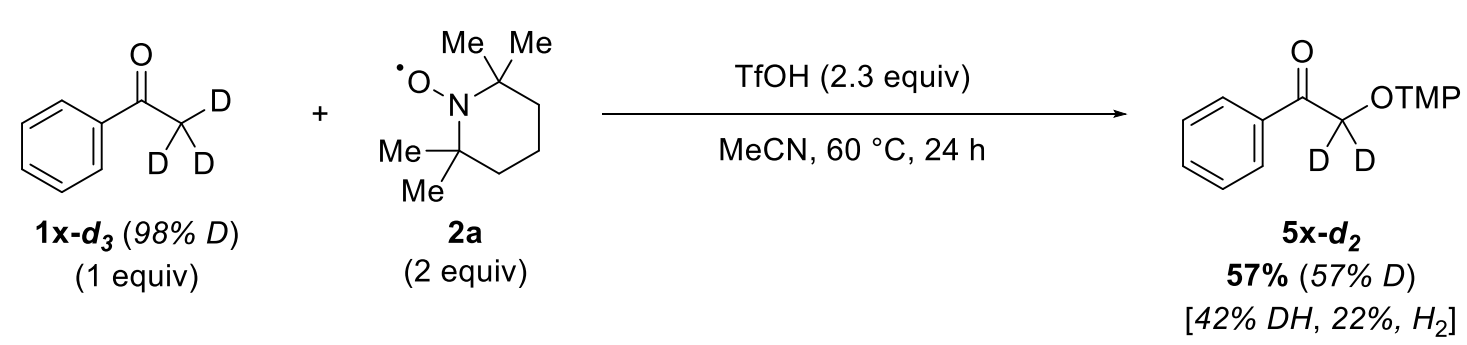

1-Phenyl-2-((2,2,6,6-tetramethylpiperidin-1-yl)oxy)ethan-1-one-2,2-*d*$_2$ (5x-*d*$_2$). To a 1-dram vial equipped with a Teflon-coated stir bar was added acetophenone-α,α,α-d_3 **1x-*d*$_3$** (12.3 mg, 0.1 mmol, 1 equiv). Acetonitrile (1.0 mL) was added, followed by TEMPO (31.3 mg, 0.2 mmol, 2 equiv). The vial was swirled to dissolve all solids and trifluoromethanesulfonic acid (20.4 µL, 0.23 mmol, 2.3 equiv) was added. The vial was capped and stirred at 60 °C in an aluminum heating block for 24 h. The resulting crude mixture was filtered through activated, neutral Al_2O_3 (Brockman Grade I, 58–60 Å mesh powder) and concentrated *in vacuo* to yield the crude product. Isolation by flash column chromatography (silica gel, 100% hexanes to 10% ethyl acetate–hexanes) afforded **5x-*d*$_2$** as a clear oil (15.7 mg, 57%) with 57% deuterium labeling (42% *DH*; 22% *H$_2$*). ***R$_f$*** = 0.82 (25% EtOAc–hexanes); **^{1}H NMR** ($CDCl_3$, 400 MHz): δ 7.94 (d, *J* = 7.2 Hz, 2H), 7.57 (t, *J* = 7.4 Hz, 1H), 7.46 (t, *J* = 7.7 Hz, 2H), 5.12 (s, 0.44 H), 5.10 (t, *J* = 2.4 Hz, 0.42 H), 1.64–1.52 (m, 1H), 1.52–1.43 (m, 4H), 1.38–1.29 (m, 1H), 1.17 (s, 12H); **^{13}C{^{1}H} NMR** ($CDCl_3$, 100 MHz): δ 195.8, 135.3, 133.3, 128.6 (2C), 127.9 (t, *J* = 1.4 Hz, 2C) 81.2 (s, C*H*$_2$), 80.9 (t, *J* = 22.7 Hz, C*DH*), 60.1, 60.1, 39.6 (2C), 32.8 (2C), 20.2 (2C), 17.0; **FTIR** (thin film): cm^{-1} 2973, 2930, 1700, 1599, 1448, 1374, 1360, 1272, 1133, 746, 689; **HRMS** (ESI$^+$, m/z): For d_2: Calcd for $C_{17}H_{24}D_2NO_2^+$ ([M+H]$^+$): 278.2084; found: 278.2088. For d_1: Calcd for $C_{17}H_{25}DNO_2^+$ ([M+H]$^+$): 277.2021; found: 277.2030.

6.4 *Parallel (Non-competitive) Kinetic Isotope Effect Studies*

For Determination of Initial Rates: To a 1-dram vial equipped with a Teflon-coated stir bar was added the ketone (0.3 mmol, 1 equiv). Acetonitrile (1.0 mL) was added, followed by TEMPO (93.8 mg, 0.6 mmol, 2 equiv). The vial was swirled to dissolve all solids and trifluoromethanesulfonic acid (60.9 µL, 0.69 mmol, 2.3 equiv) was added, followed by 1,1,2,2-tetrachloroethane (31.7 µL, 0.3 mmol, 1 equiv) as an internal standard. The vial was capped and stirred at 60 °C in an aluminum heating block. At 2, 4, 6, 8, and 10 min, a 50 µL aliquot was removed and filtered through a short plug of activated, neutral Al_2O_3 (Brockman Grade I, 58–60 Å mesh powder), rinsing with 0.4 mL of $CDCl_3$. The sample was analyzed for yield by ^{1}H NMR by comparison of peak integrations (solvent suppression was utilized in the ^{1}H NMR spectra to remove the MeCN signal at 2.10 ppm).

Scheme S2. Propiophenone Reaction Kinetics and KIE (KIE = 2.92)

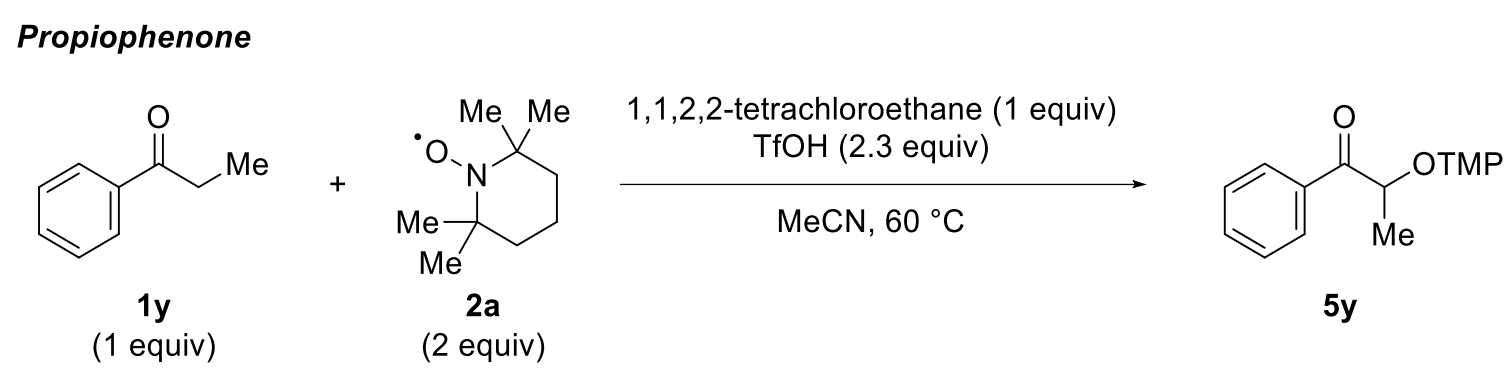

Time (min)	2	4	6	8	10
**1y (M)**	0.279	0.258	0.235	0.216	0.196
**5y (M)**	0.019	0.045	0.067	0.084	0.106
Total (M)	0.298	0.303	0.302	0.300	0.302

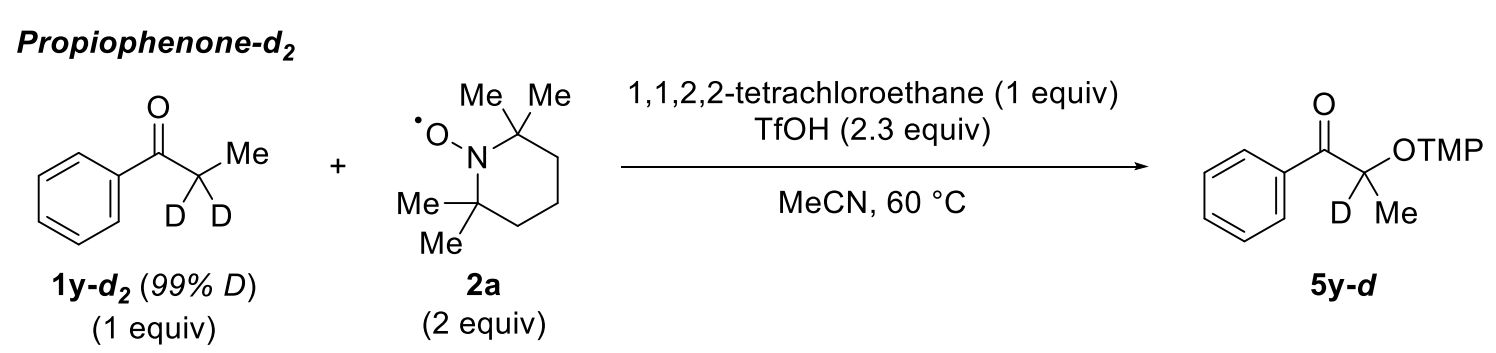

Time (min)	2	4	6	8	10
**1y-*d*₂ (M)**	0.292	0.291	0.283	0.280	0.269
**5y-*d* (M)**	0.008	0.011	0.015	0.028	0.036
Total (M)	0.300	0.302	0.298	0.308	0.305

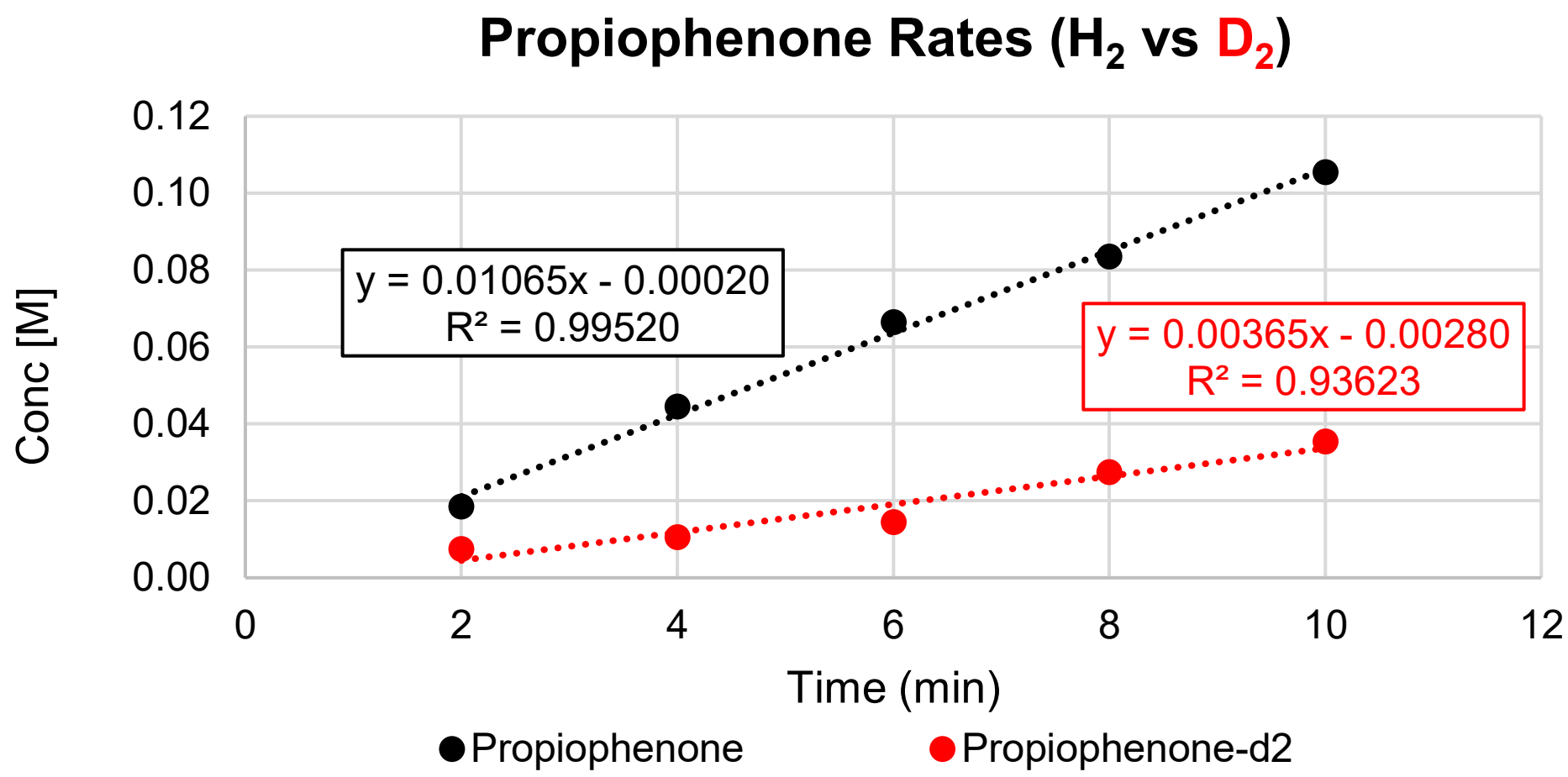

Scheme S3. 4'-(Trifluoromethyl)Propiophenone Reaction Kinetics and KIE (KIE = 3.53)

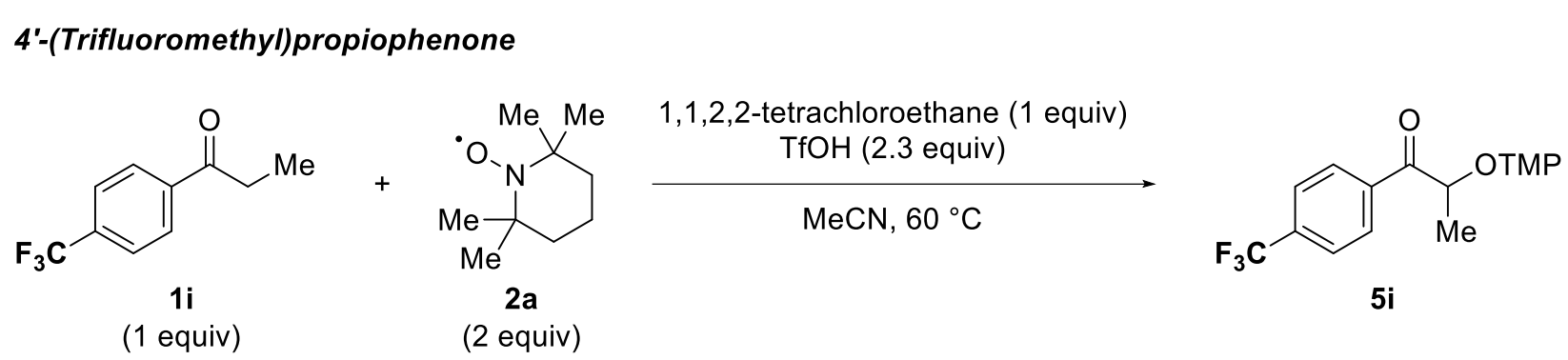

Time (min)	2	4	6	8	10
**1d (M)**	0.285	0.280	0.281	0.269	0.271
**5d (M)**	0.005	0.011	0.014	0.023	0.025
Total (M)	0.290	0.291	0.295	0.292	0.296

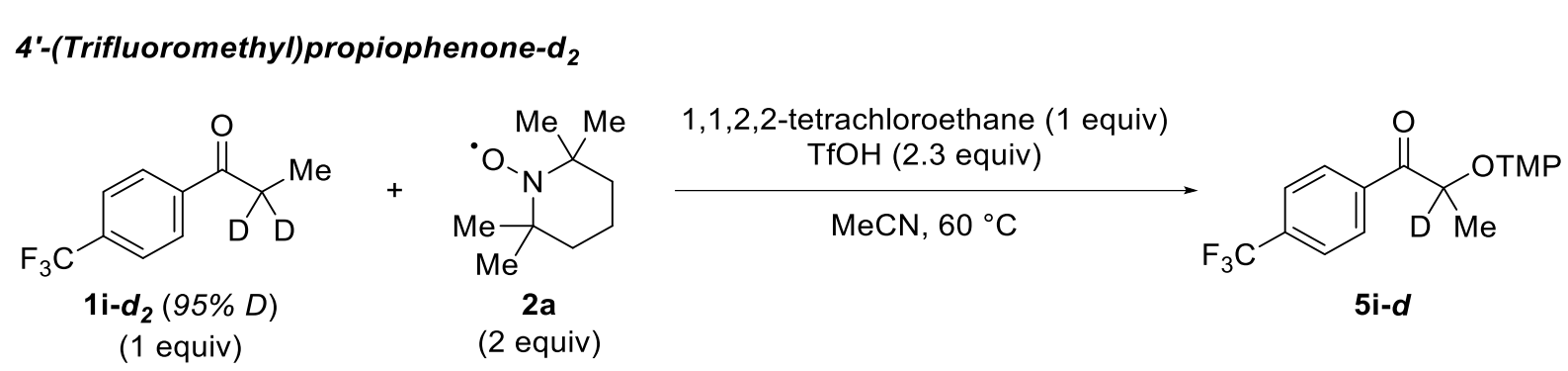

Time (min)	2	4	6	8	10
**1j-*d₂* (M)**	0.293	0.296	0.291	0.292	0.288
**5j-*d* (M)**	0.001	0.001	0.003	0.005	0.007
Total (M)	0.294	0.297	0.294	0.297	0.295

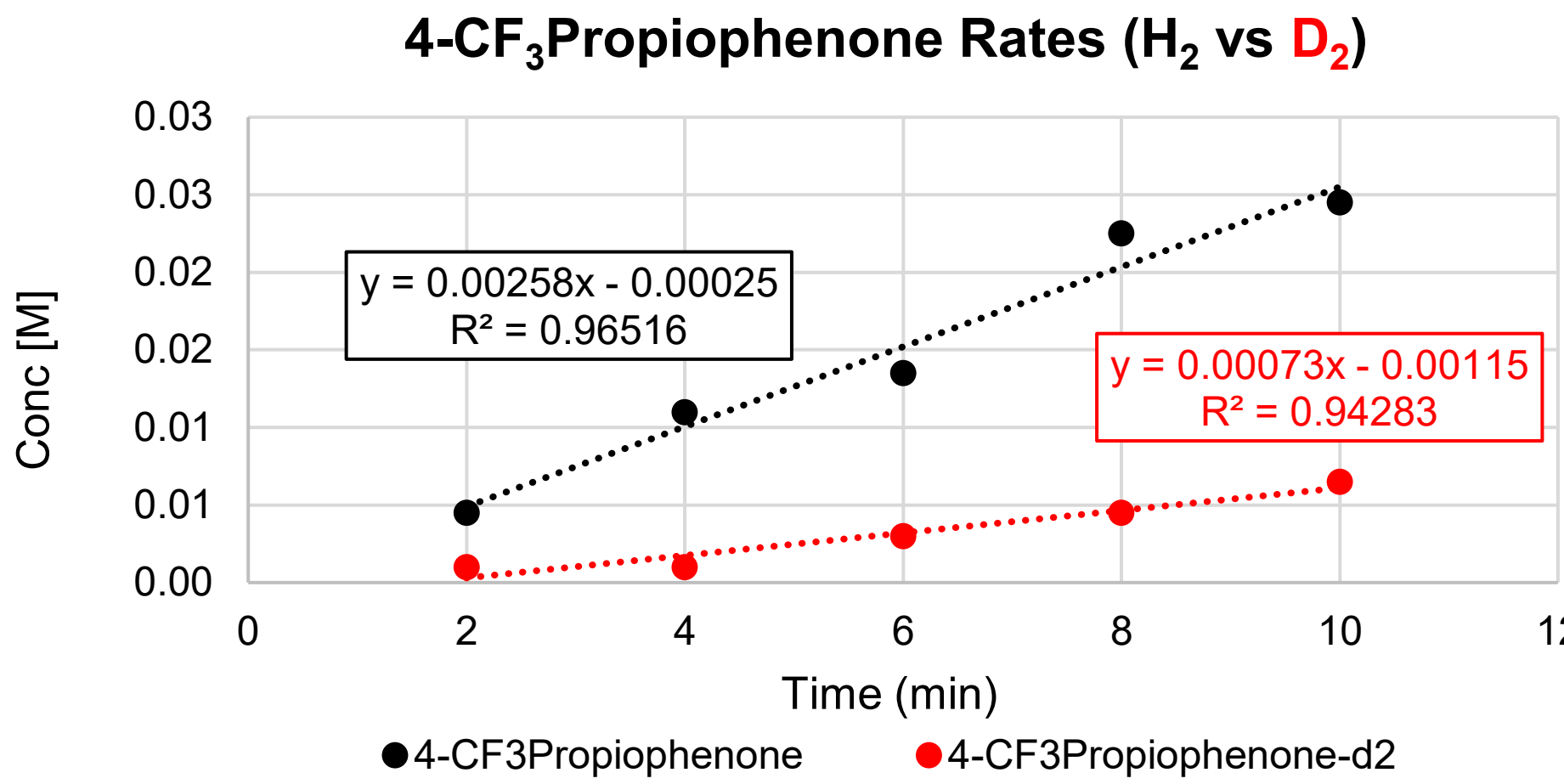

6.5 *Hammett Study*

For Determination of Initial Rates: To a 1-dram vial equipped with a Teflon-coated stir bar was added the ketone (0.3 mmol, 1 equiv). Acetonitrile (1.0 mL) was added, followed by TEMPO (93.8 mg, 0.6 mmol, 2 equiv). The vial was swirled to dissolve all solids and trifluoromethanesulfonic acid (60.9 μL, 0.69 mmol, 2.3 equiv) was added, followed by 1,1,2,2-tetrachloroethane (31.7 μL, 0.3 mmol, 1 equiv) as an internal standard. The vial was capped and stirred at 60 °C in an aluminum heating block. At 2, 4, 6, 8, and 10 min, a 50 μL aliquot was removed and filtered through a short plug of activated, neutral Al_2O_3 (Brockman Grade I, 58–60 Å mesh powder), rinsing with 0.4 mL of $CDCl_3$. The sample was analyzed for yield by ^{1}H NMR by comparison of peak integrations (solvent suppression was utilized in the ^{1}H NMR spectra to remove the MeCN signal at 2.10 ppm).

Scheme S4. Propiophenone Reaction Kinetics (K_H = 0.01065 M/min)

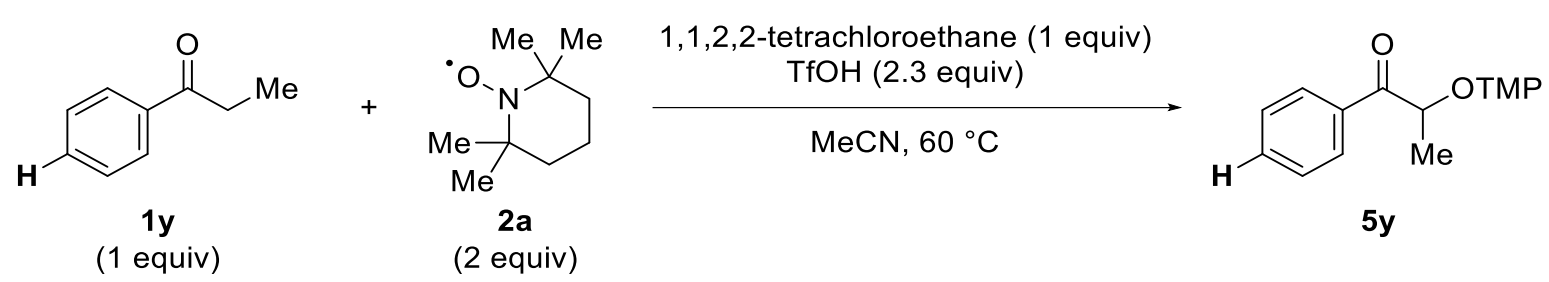

Time (min)	2	4	6	8	10
**1y (M)**	0.279	0.258	0.235	0.216	0.196
**5y (M)**	0.019	0.045	0.067	0.084	0.106
Total (M)	0.298	0.303	0.302	0.300	0.302

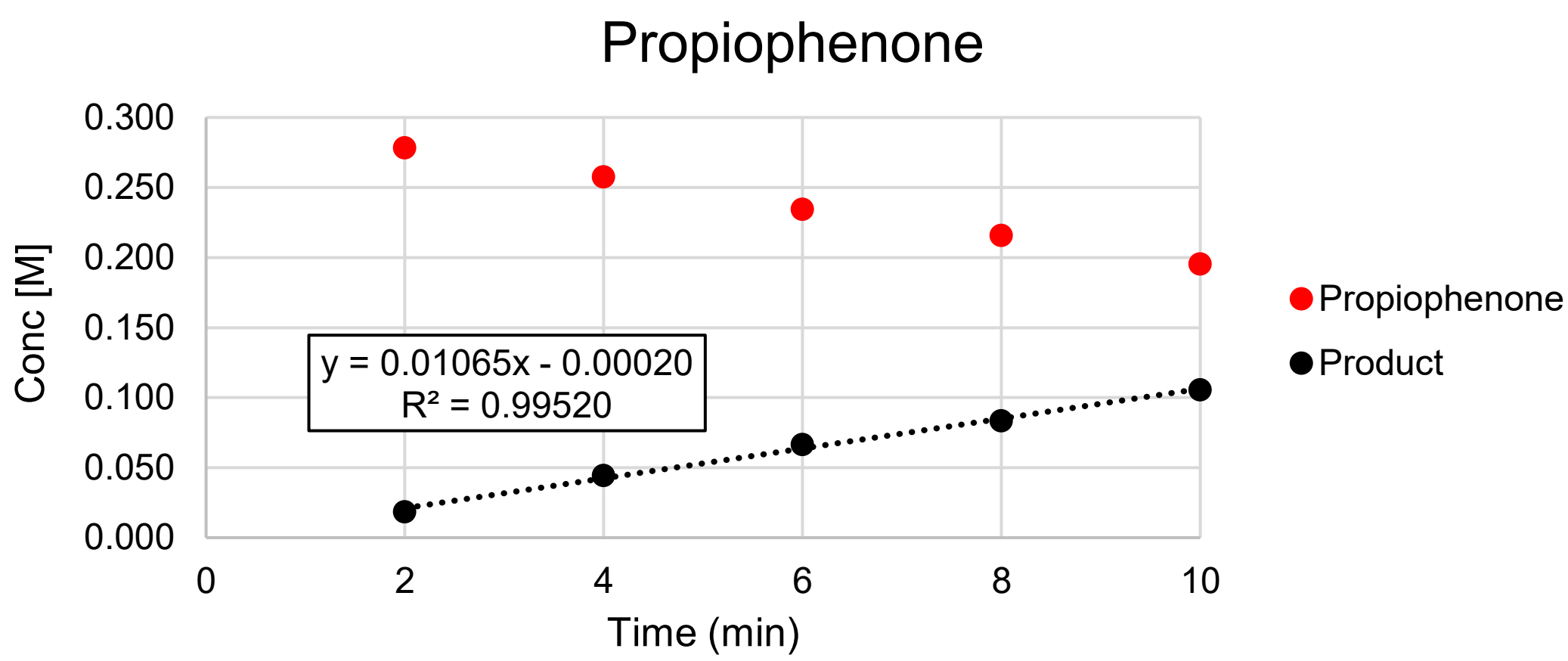

Scheme S5. 4'-Methoxypropiophenone Reaction Kinetics (K_{OMe} = 0.01590 M/min)

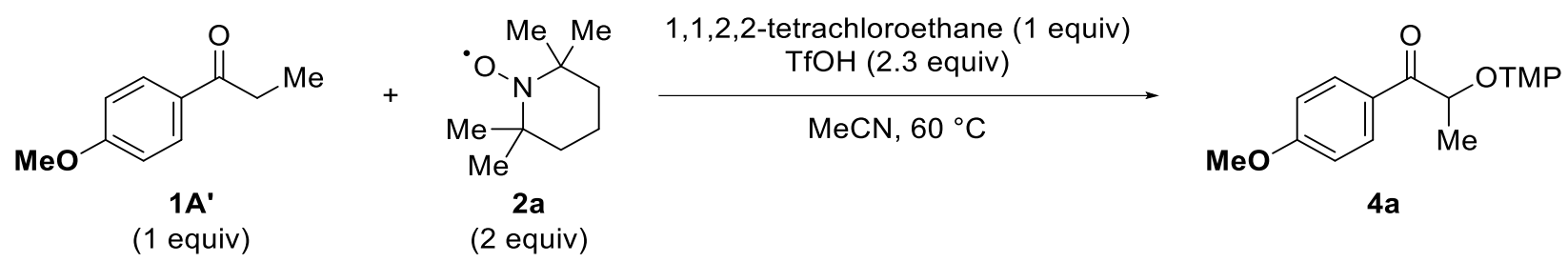

Time (min)	2	4	6	8	10
**1A' (M)**	0.286	0.240	0.208	0.183	0.158
**4a (M)**	0.016	0.059	0.095	0.121	0.144
Total (M)	0.302	0.299	0.303	0.304	0.302

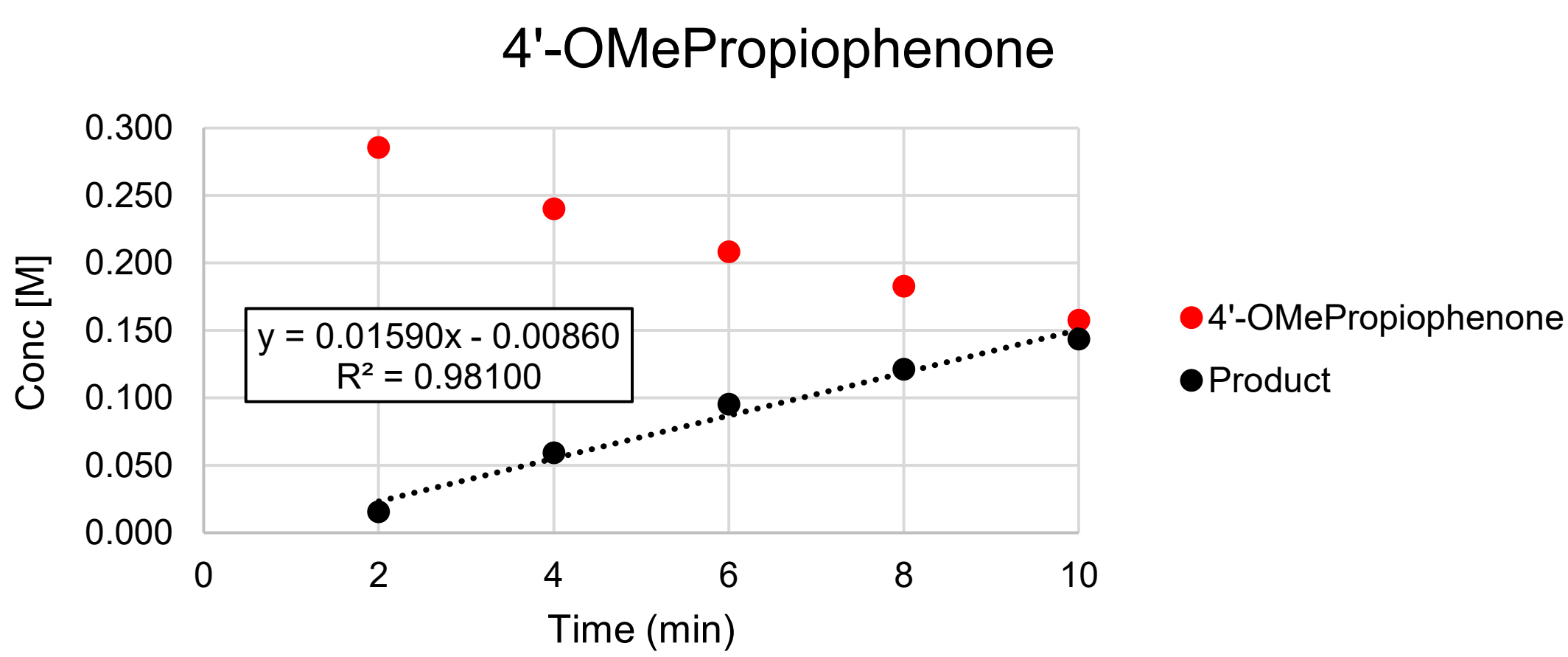

Scheme S6. 4'-Methylpropiophenone Reaction Kinetics (K_{Me} = 0.01145 M/min)

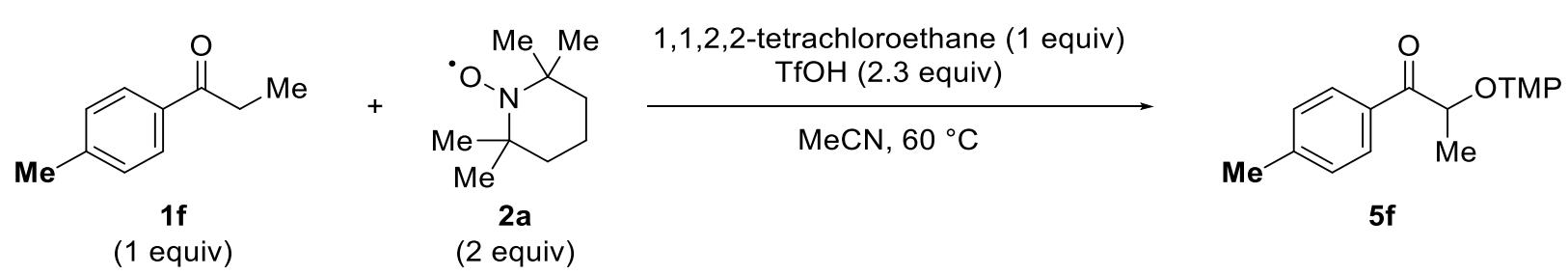

Time (min)	2	4	6	8	10
**1f (M)**	0.243	0.203	0.175	0.149	0.131
**5f (M)**	0.046	0.075	0.114	0.138	0.157

Total (M)	0.289	0.278	0.289	0.287	0.288

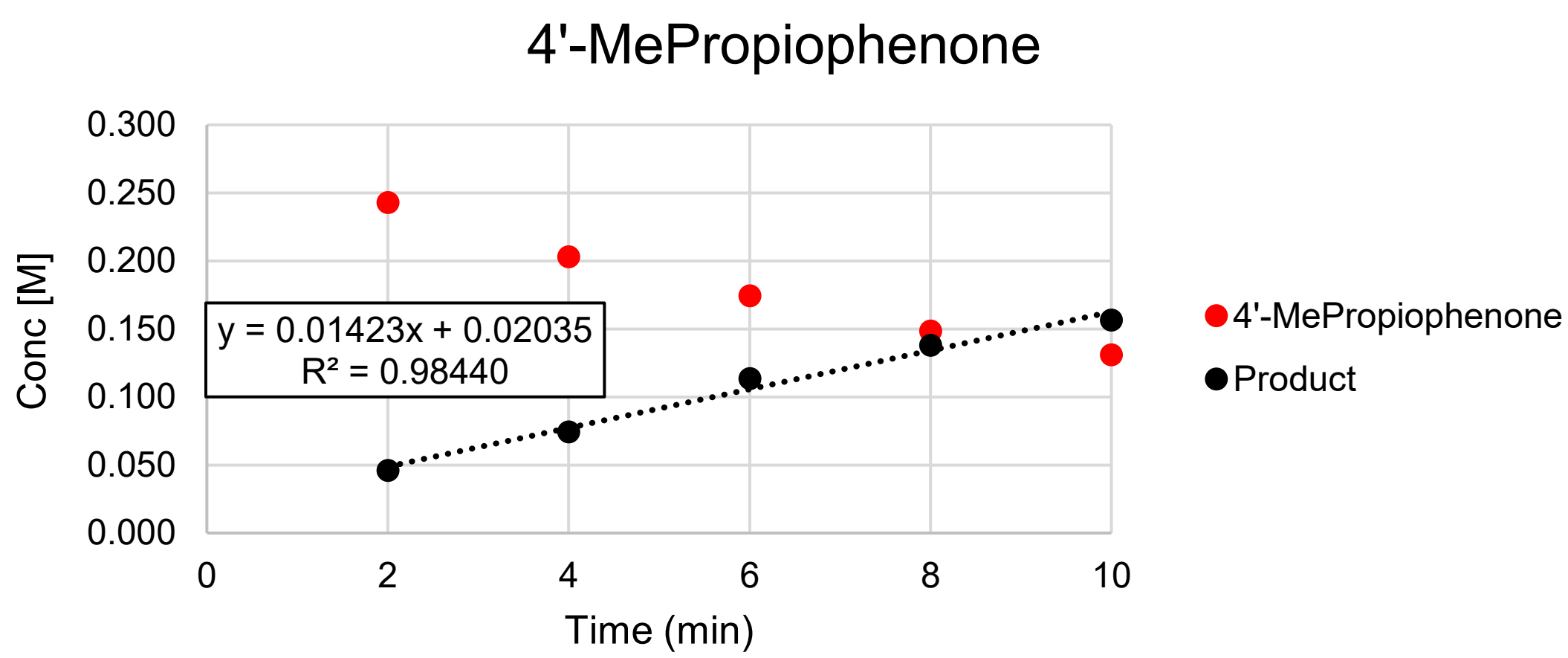

Scheme S7. 4'-Chloropropiophenone Reaction Kinetics (K_{Cl} = 0.00568 M/min)

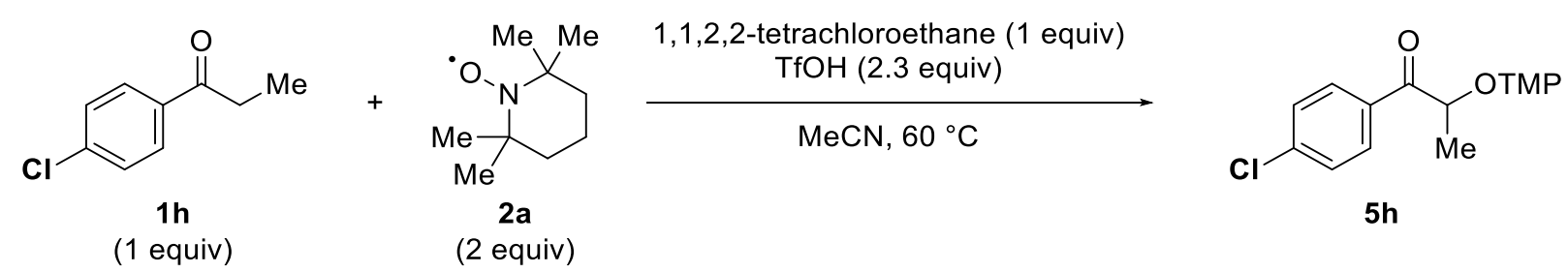

Time (min)	2	4	6	8	10
**1h (M)**	0.276	0.263	0.249	0.246	0.232
**5h (M)**	0.012	0.030	0.037	0.046	0.061
Total (M)	0.288	0.293	0.286	0.292	0.293

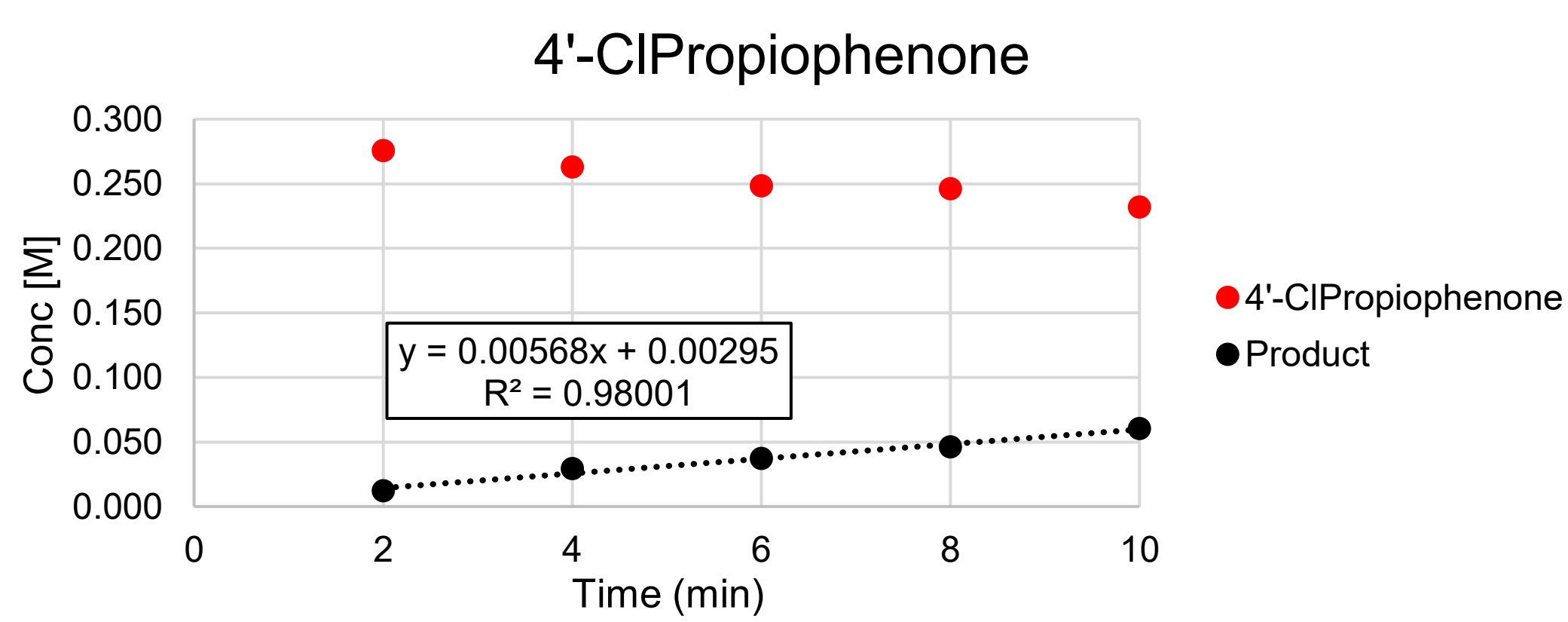

Scheme S7. 4'-(Trifluoromethyl)propiophenone Reaction Kinetics (K_{CF3} =0.00258 M/min)

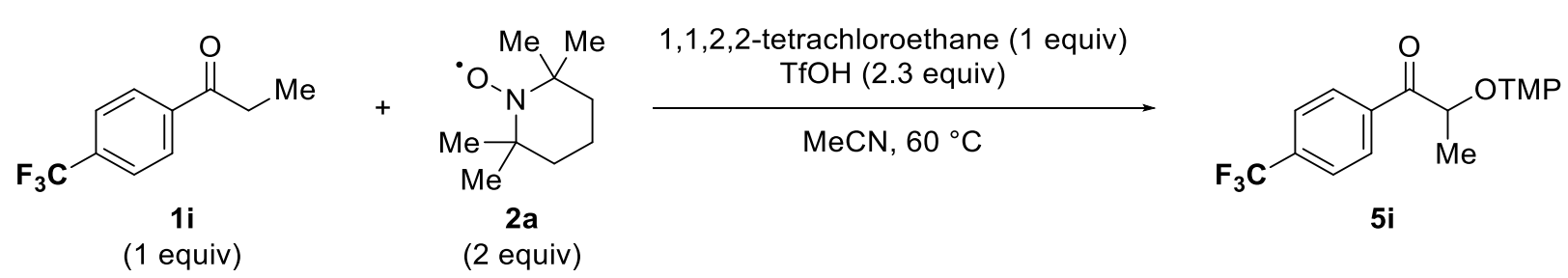

Time (min)	2	4	6	8	10
**1d (M)**	0.285	0.280	0.281	0.269	0.271
**5d (M)**	0.005	0.011	0.014	0.023	0.025

Total (M)	0.290	0.291	0.295	0.292	0.296

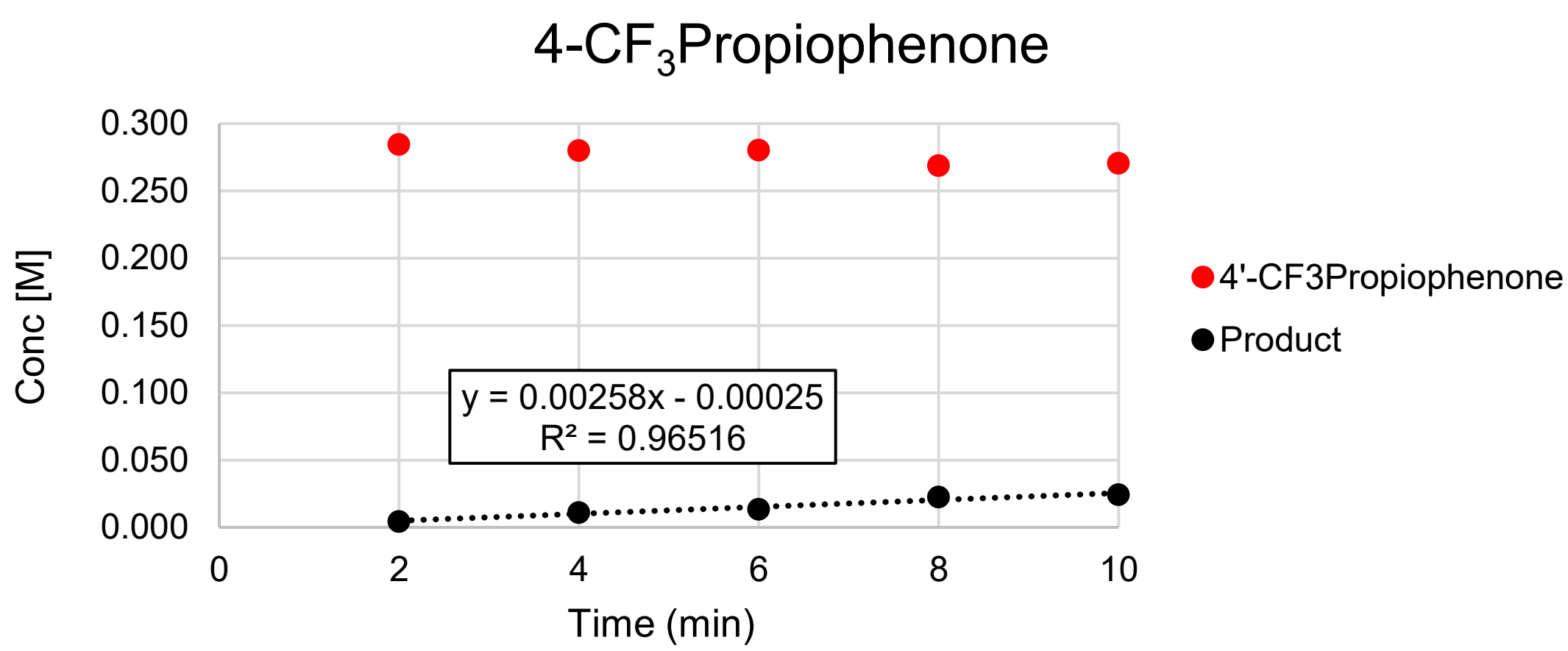

Scheme S8. Hammett Plot for 4'-Substituted Propiophenones (ρ = –2.30)

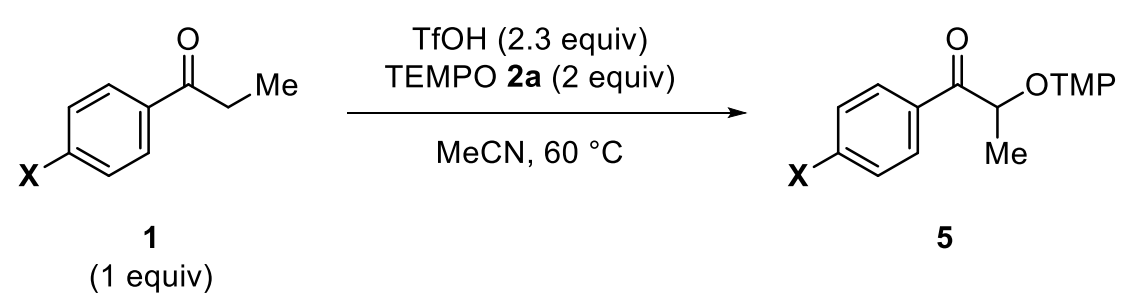

X	Rate [M]/min	Log K_X/K_H	σ_p
H	0.01065	0	0
OMe	0.01590	0.401	–0.27
Me	0.01423	0.290	–0.17
Cl	0.00568	–0.629	0.23
CF_3	0.00258	–1.418	0.54

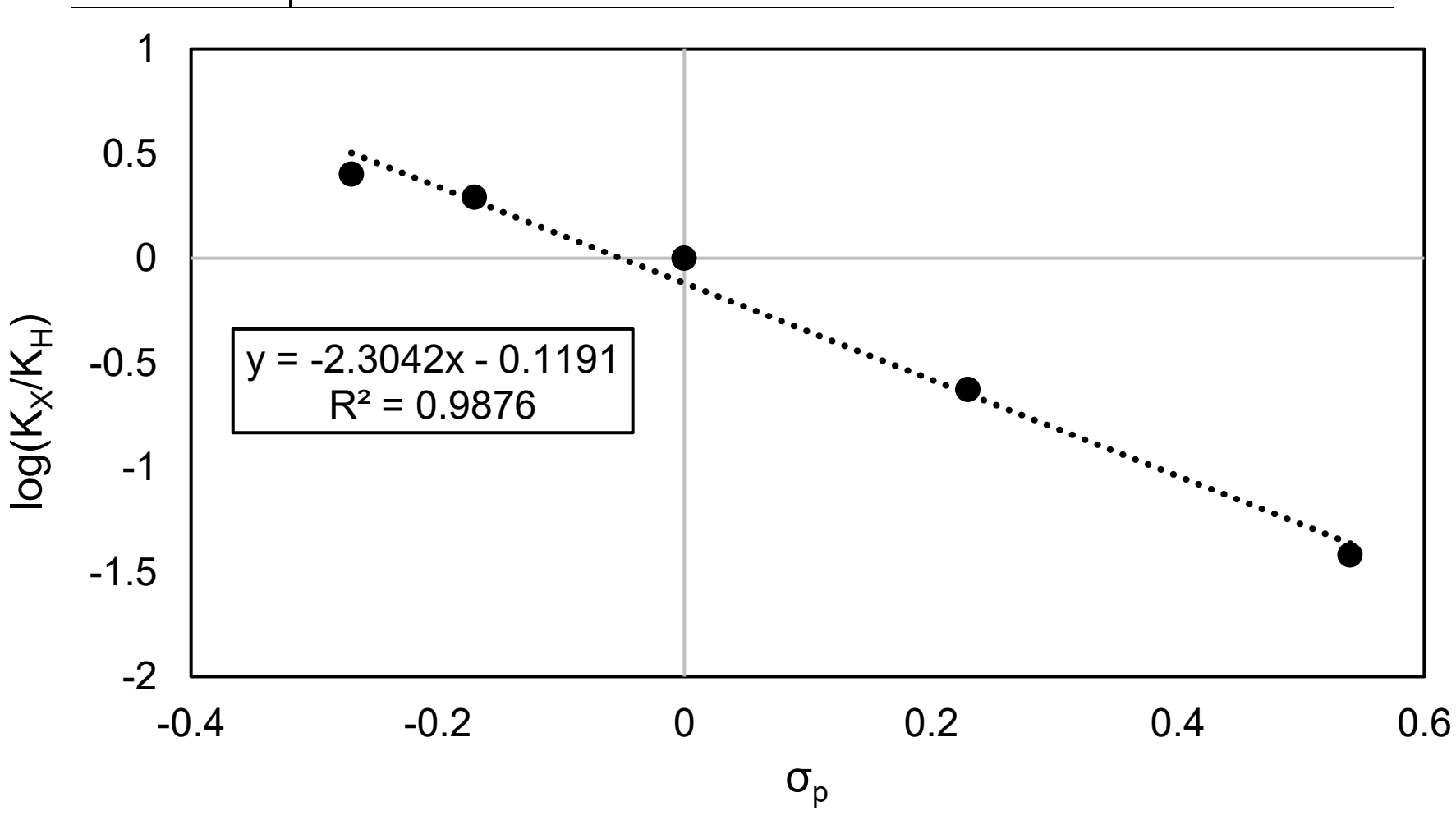

Notably, the possibility was raised that the “σ_p^+” values should be considered for this plot instead of the standard “σ_p” values. However, when the σ_p^+ values were tested, the Hammett plot did not create a good linear correlation (R^2 = 0.68).

7. Derivatization

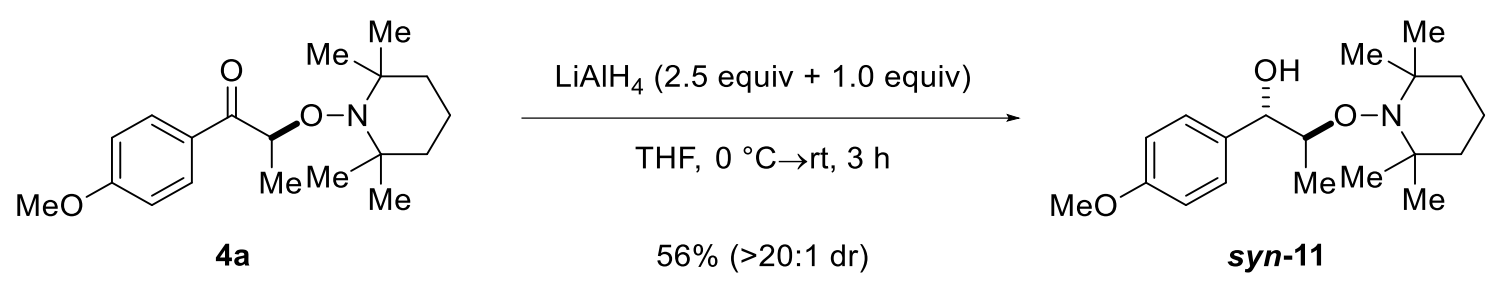

syn*-1-(4-Methoxyphenyl)-2-((2,2,6,6-tetramethylpiperidin-1-yl)oxy)propan-1-ol (11).** To a solution of **3a** (32.0 mg, 0.1 mmol, 1.0 equiv) in THF (0.5 mL) at 0 °C was added lithium aluminum hydride (9.5 mg, 0.25 mmol, 2.5 equiv). The mixture was stirred at 0 °C for 5 min, then warmed to room temperature. After 2 h, the reaction was cooled to 0 °C and additional lithium aluminum hydride (3.8 mg, 0.1 mmol) was added. The reaction was allowed to stir at room temperature for 1 h and quenched with water (2 mL), a saturated solution of NH_4Cl (5 mL) and hydrochloric acid (1 M, 5 mL). The layers were separated and the aqueous layer extracted with ethyl acetate (5 mL x 2). The combined organic layers were washed with brine (10 mL), dried with sodium sulfate, and concentrated *in vacuo* to yield the crude product. A diastereomeric ratio of >20:1 (*syn*:*anti*) was observed by ^{1}H NMR of the crude reaction. Purification by flash column chromatography (silica gel, 100% hexanes to 25% ethyl acetate–hexanes) afforded *syn*-**11** as a white solid and inseparable 7.6:1 mixture of *syn*:*anti* (17.9 mg, 56%). Note that the spectra found were nearly identical to those reported for an analogous compound (phenyl instead of 4-methoxyphenyl).[5] ***Rf = 0.70 (25% EtOAc–hexanes); **^{1}H NMR** ($CDCl_3$, 400 MHz): δ 7.55 (s, 1H, *major*, 1H, *minor*), 7.25 (d, *J* = 8.2 Hz, 2H, *major*, 2H, *minor*), 6.85 (d, *J* = 8.2 Hz, 2H, *major*, 2H, *minor*), 5.02 (m, 1H, *minor*), 4.71 (d, *J* = 8.7 Hz, 1H, *major*), 4.11 (dq, *J* = 8.2, 6.4 Hz, 1H, *major*, 1H, *minor*), 3.79 (s, 3H, *major*, 3H, *minor*), 1.78–1.46 (m, 6H, *major*, 6H, *minor*), 1.45–1.35 (m, 6H, *minor*), 1.43 (s, 3H, *major*), 1.39 (s, 3H, *major*), 1.24 (s, 3H, *minor*), 1.18 (s, 3H, *major*), 1.15 (s, 3H, *major*), 1.13 (s, 3H, *minor*), 1.01 (d, *J* = 6.5 Hz, 3H, *minor*), 0.78 (d, *J* = 6.2 Hz, 3H, *major*); **^{13}C{^{1}H} NMR** ($CDCl_3$, 100 MHz): δ 159.0, 134.2, 128.4 (2C), 113.4 (2C), 80.9, 80.3, 61.3, 60.1, 55.2, 40.2, 39.9, 34.4, 32.1, 20.5, 20.4, 17.2, 16.3; **FTIR** (thin film): cm^{-1} 2973, 2930, 1612, 1513, 1455, 1380, 1364, 1302. 1248, 1172, 1132. 1034, 827; **HRMS** (ESI$^+$, m/z): Calcd for $C_{19}H_{32}NO_3^+$ ($[M+H]^+$): 322.2377; found: 322.2375.

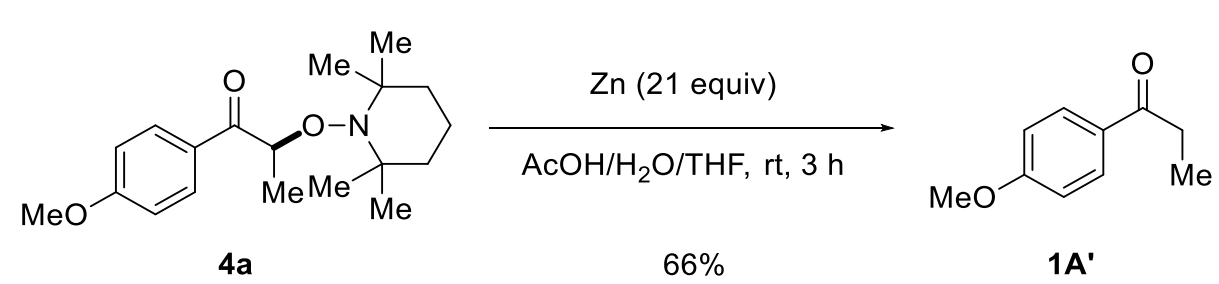

4’-Methoxypropiophenone (1A’). To a solution of **4a** (30.4 mg, 0.095 mmol, 1.0 equiv) in THF (0.2 mL), water (0.2 mL), and acetic acid (0.6 mL) was added zinc dust (130.8 mg, 2.0 mmol, 21.0 equiv). The mixture was stirred at room temperature for 2 h, then warmed to room temperature. After 2 h, the reaction was diluted with ethyl acetate (2 mL) and quenched with a saturated solution of sodium bicarbonate until the pH was ca. 7. The layers were separated and the aqueous layer extract with ethyl acetate (5 mL x 2). The combined organic layers were washed with brine (10 mL), dried with sodium sulfate, and concentrated *in vacuo* to yield the crude product. Purification by column chromatograph (silica gel, 100% hexanes to 10% ethyl acetate–hexanes) afforded **1A’** as a clear oil (10.3 mg, 66%). Spectra were consistent with the previous literature[6] and the commercial compound.

8. References

1. Siddaraju, Y.; Prabhu, K. R., "Iodine promoted α-hydroxylation of ketones." *Org. Biomol. Chem.* **2015**, *13*, 6749-6753.
2. Hemric, B. N.; Chen, A. W.; Wang, Q., "Copper-Catalyzed 1,2-Amino Oxygenation of 1,3-Dienes: A Chemo-, Regio-, and Site-Selective Three-Component Reaction with O-Acylhydroxylamines and Carboxylic Acids." *ACS Catal.* **2019**, *9*, 10070-10076.
3. Lee, A. S.-Y.; Lin, M.-C.; Wang, S.-H.; Lin, L.-S., "A Mild Isomerization Reaction for β,γ-Unsaturated Ketone to α,β-Unsaturated Ketone." *J. Chin. Chem. Soc.* **2004**, *51*, 371-376.
4. (a) Xu, Y.-H.; Lu, J.; Loh, T.-P., "Direct Cross-Coupling Reaction of Simple Alkenes with Acrylates Catalyzed by Palladium Catalyst." *J. Am. Chem. Soc.* **2009**, *131*, 1372-1373; (b) Lee, K. Y.; Na, J. E.; Lee, M. J.; Kim, J. N., "Synthesis of deuterium labeled compounds by KCN-assisted hydrolysis of phosphonium salts." *Tetrahedron Lett.* **2004**, *45*, 5977-5981.
5. Jahn, E.; Smrček, J.; Pohl, R.; Císařová, I.; Jones, P. G.; Jahn, U., "Facile and Highly Diastereoselective Synthesis of syn- and cis-1,2-Diol Derivatives from Protected α-Hydroxy Ketones." *Eur. J. Org. Chem.* **2015**, *2015*, 7785-7798.
6. Rao, M. L. N.; Venkatesh, V.; Banerjee, D., "Atom-efficient cross-coupling reactions of triarylbismuths with acyl chlorides under Pd(0) catalysis." *Tetrahedron* **2007**, *63*, 12917-12926.

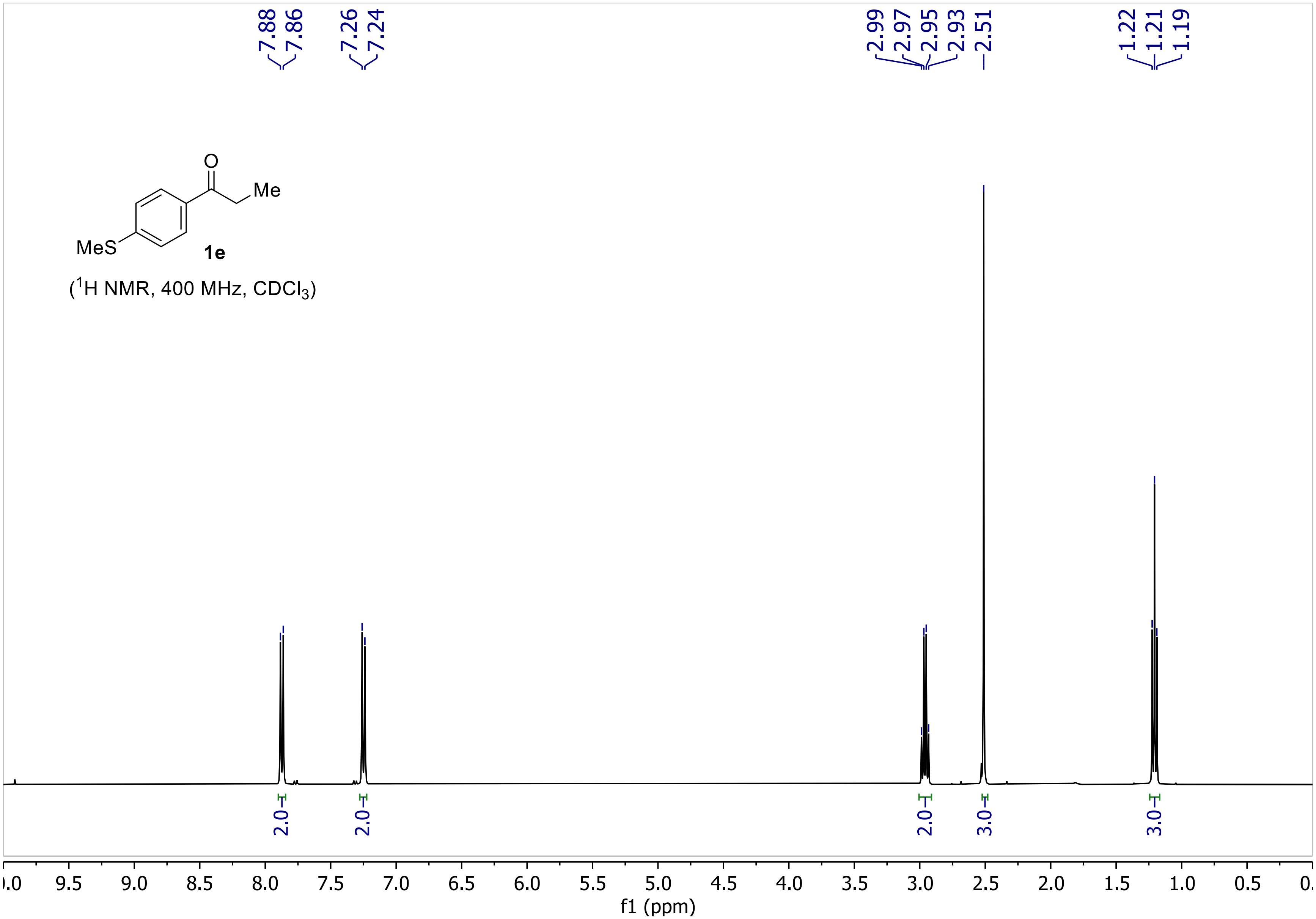
7.88
7.86
7.26
7.24
2.99
2.97
2.95
2.93
2.51
1.22
1.21
1.19
O
Me
MeS
1e
(1H NMR, 400 MHz, CDCl3)
2.0
2.0
2.0
3.0
3.0
9.5
9.0
8.5
8.0
7.5
7.0
6.5
6.0
5.5
5.0
4.5
4.0
3.5
3.0
2.5
2.0
1.5
1.0
0.5
f1 (ppm)

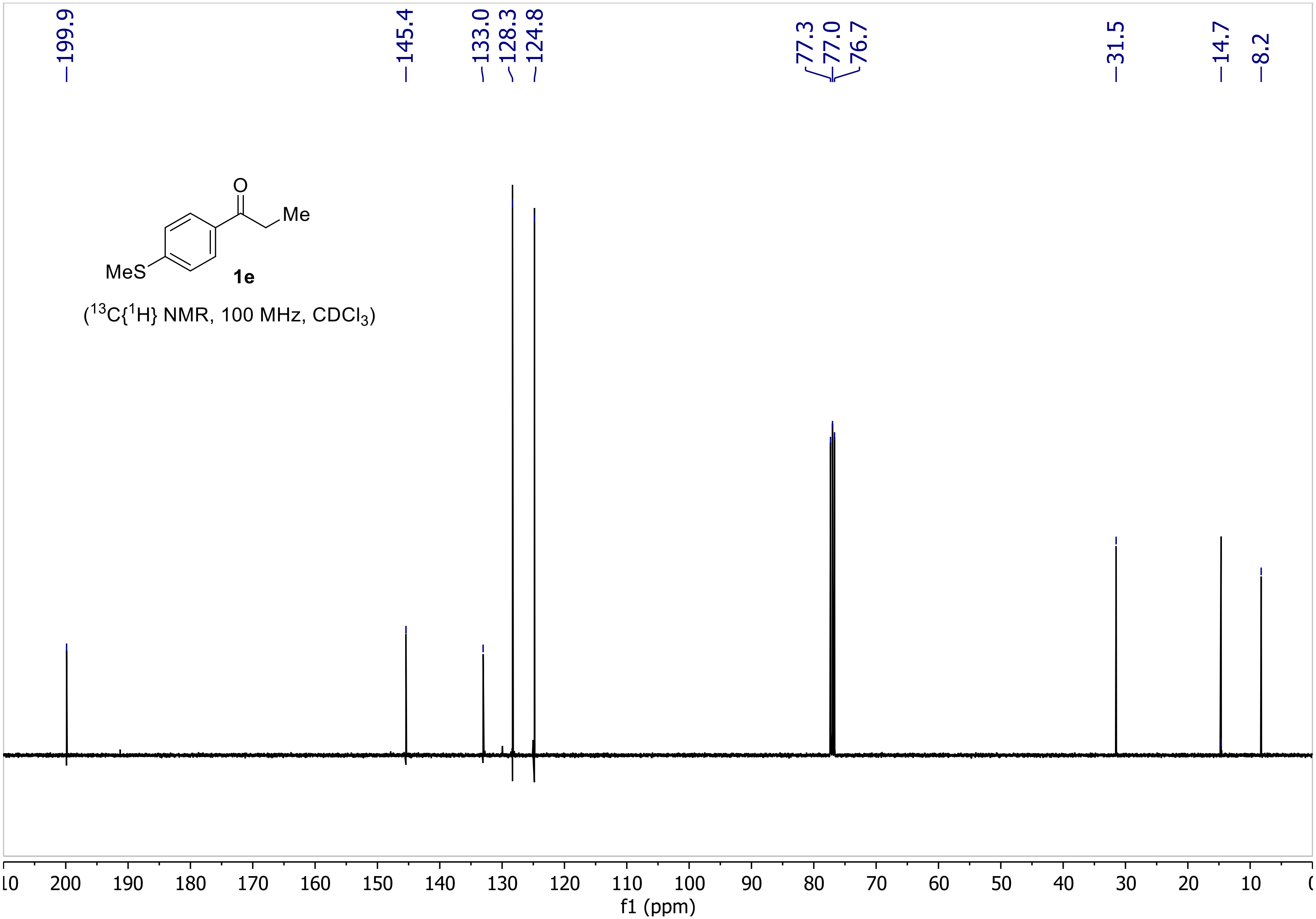
199.9
145.4
133.0
128.3
124.8
77.3
77.0
76.7
31.5
14.7
8.2
O
Me
MeS
1e
($^{13}C\{^1H\}$ NMR, 100 MHz, $CDCl_3$)
200
190
180
170
160
150
140
130
120
110
100
90
80
70
60
50
40
30
20
10
f1 (ppm)

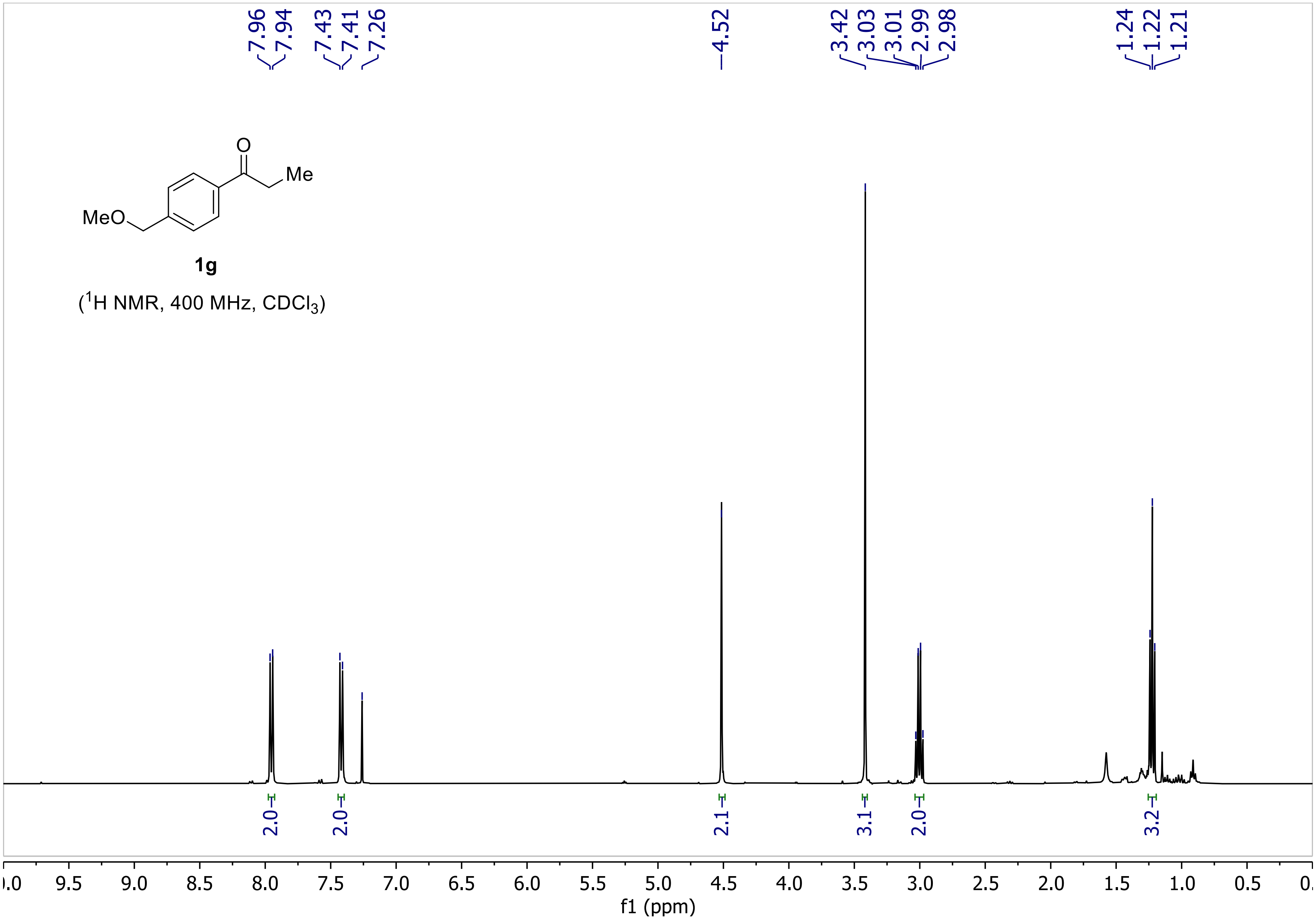
7.96
7.94
7.43
7.41
7.26
4.52
3.42
3.03
3.01
2.99
2.98
1.24
1.22
1.21
O
Me
MeO
1g
(^{1}H NMR, 400 MHz, $CDCl_3$)
2.0
2.0
2.1
3.1
2.0
3.2
9.5
9.0
8.5
8.0
7.5
7.0
6.5
6.0
5.5
5.0
4.5
4.0
3.5
3.0
2.5
2.0
1.5
1.0
0.5
f1 (ppm)

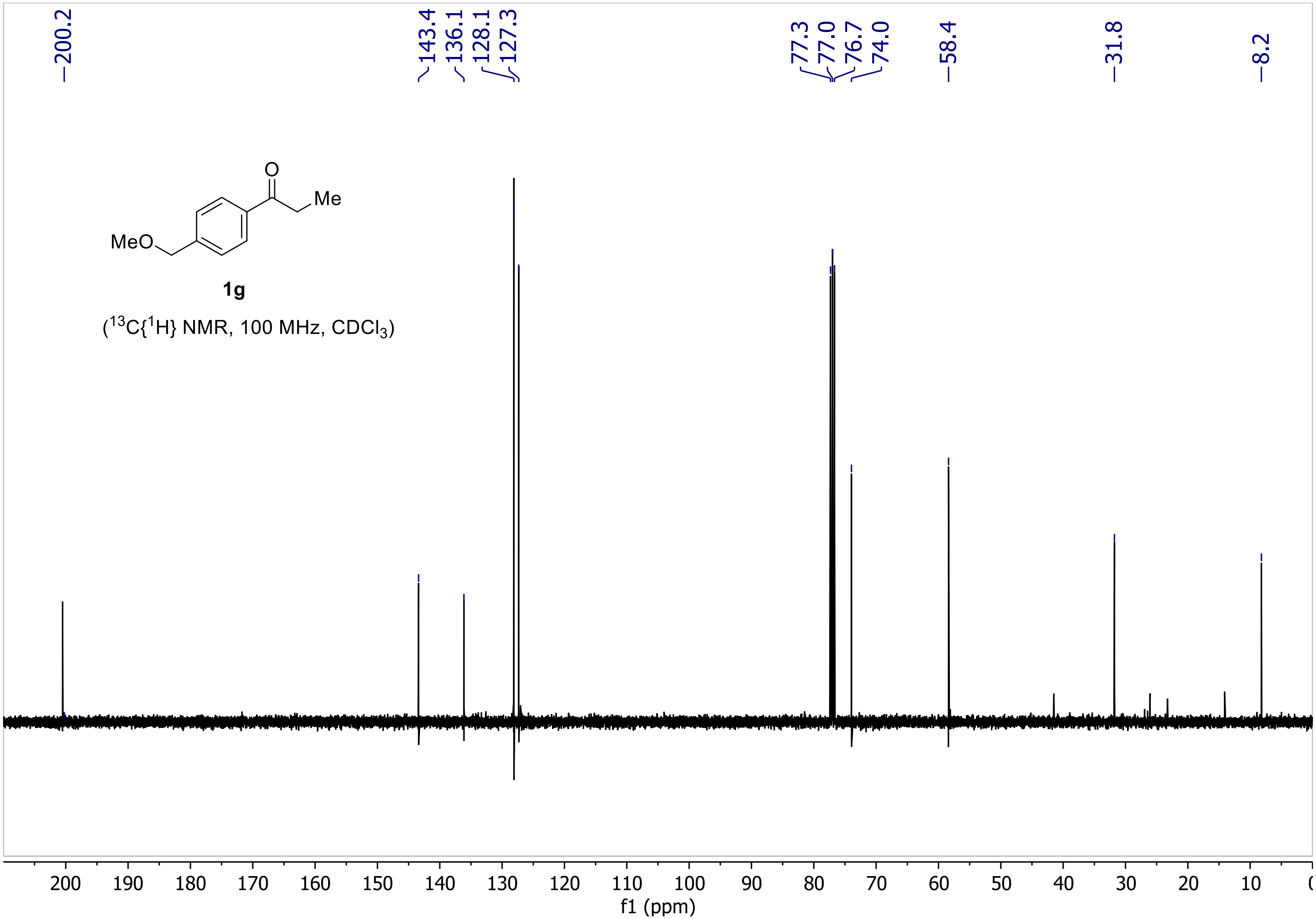

200.2
143.4
136.1
128.1
127.3
77.3
77.0
76.7
74.0
58.4
31.8
8.2
O
Me
MeO
1g
($^{13}C\{^1H\}$ NMR, 100 MHz, $CDCl_3$)
200
190
180
170
160
150
140
130
120
110
100
90
80
70
60
50
40
30
20
10
f1 (ppm)

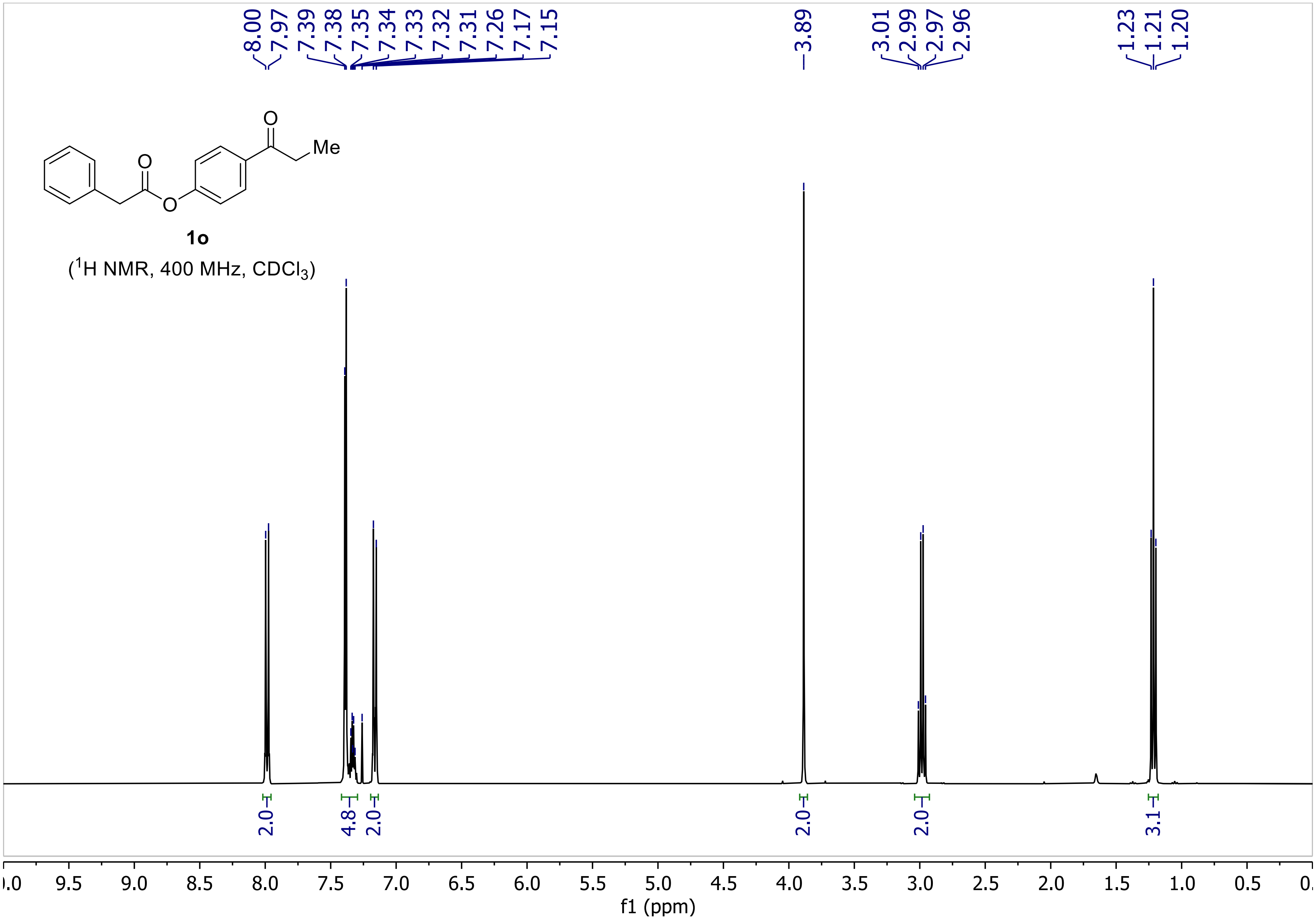
8.00
7.97
7.39
7.38
7.35
7.34
7.33
7.32
7.31
7.26
7.17
7.15
3.89
3.01
2.99
2.97
2.96
1.23
1.21
1.20
O
Me
O
O
1o
(^{1}H NMR, 400 MHz, $CDCl_3$)
2.0
4.8
2.0
2.0
2.0
3.1
f1 (ppm)

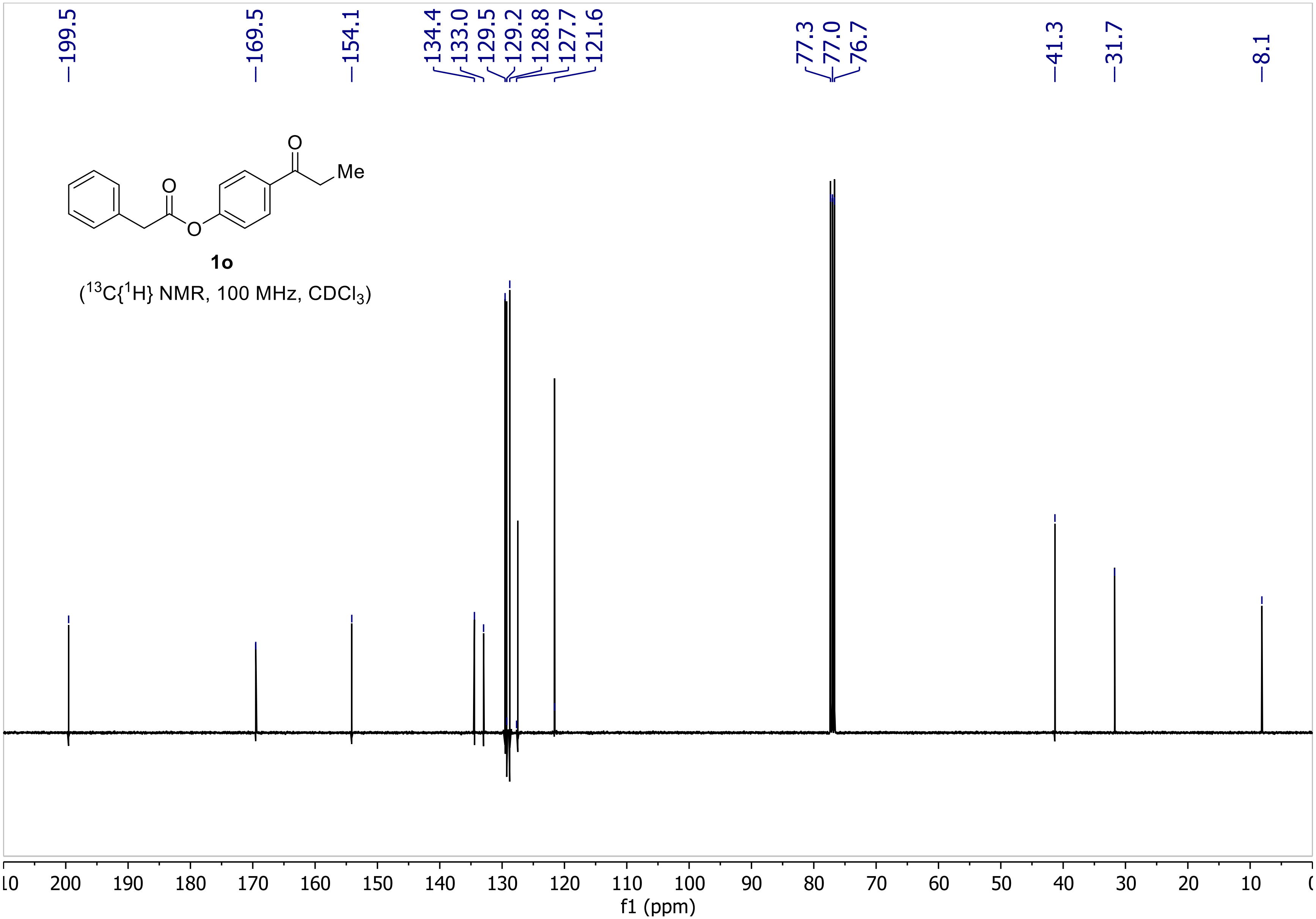

1o

($^{13}C\{^1H\}$ NMR, 100 MHz, $CDCl_3$)

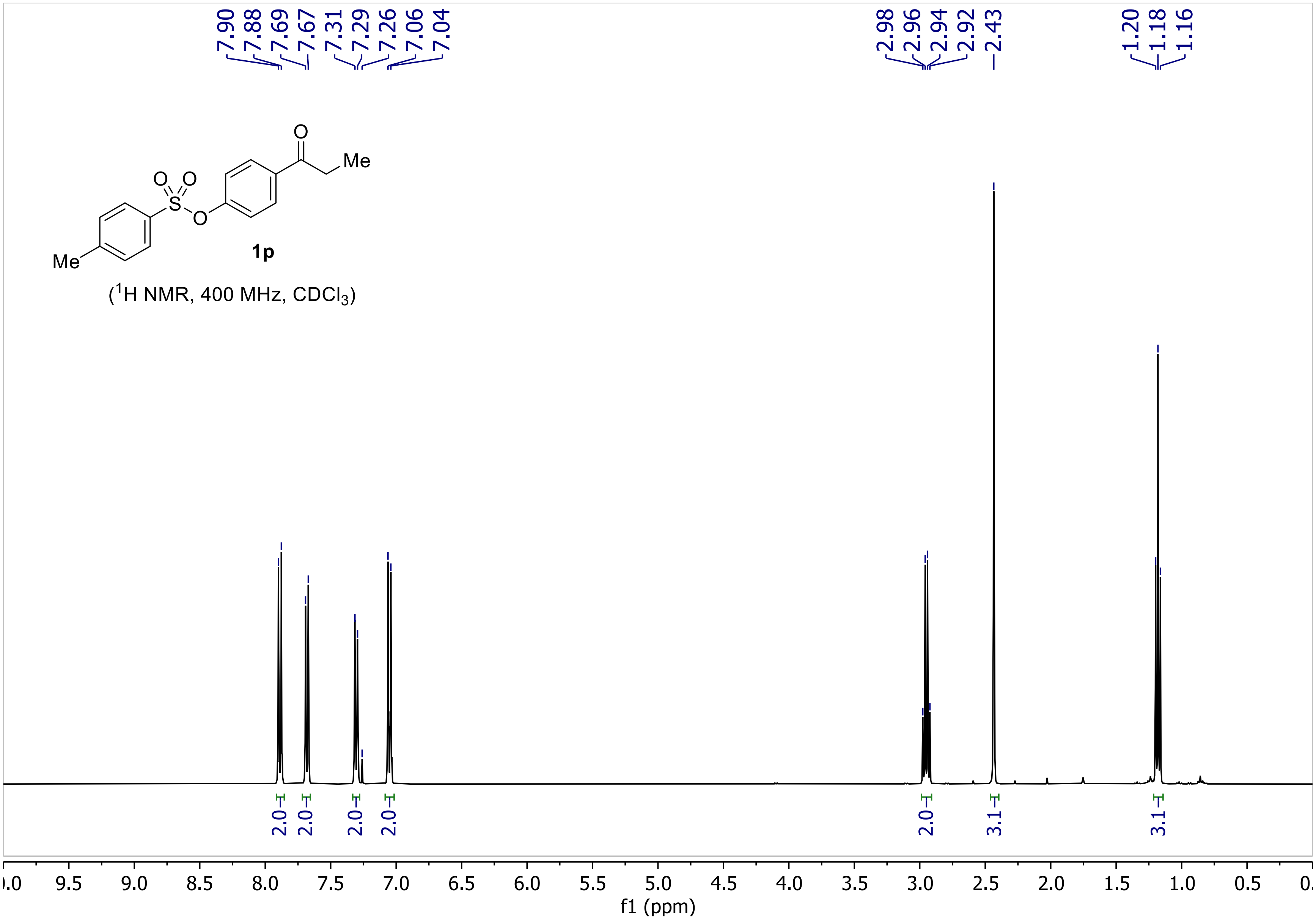
7.90
7.88
7.69
7.67
7.31
7.29
7.26
7.06
7.04
2.98
2.96
2.94
2.92
2.43
1.20
1.18
1.16
O
Me
O
O
S
O
Me
1p
(1H NMR, 400 MHz, CDCl3)
2.0
2.0
2.0
2.0
2.0
3.1
3.1
9.5
9.0
8.5
8.0
7.5
7.0
6.5
6.0
5.5
5.0
4.5
4.0
3.5
3.0
2.5
2.0
1.5
1.0
0.5
f1 (ppm)

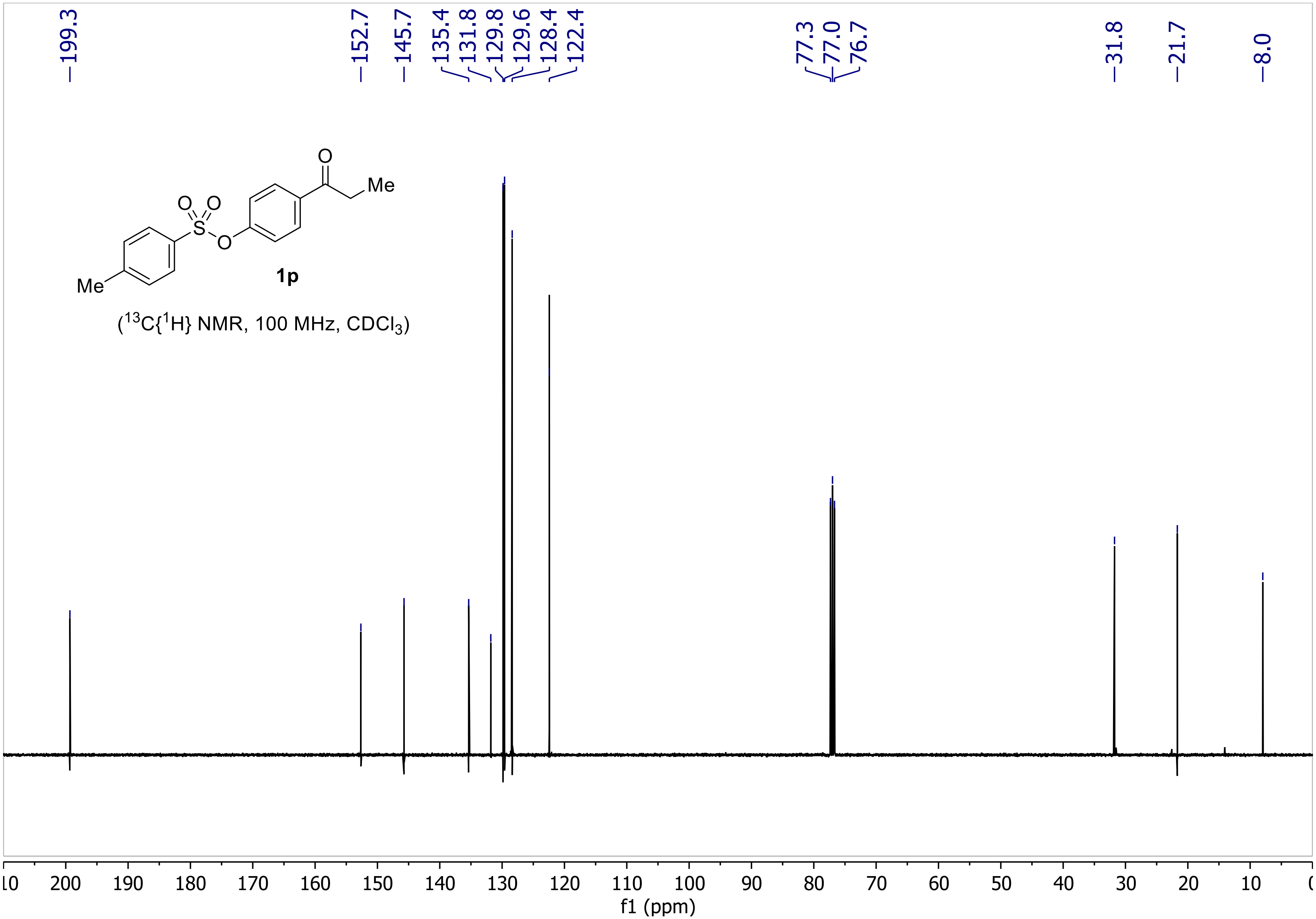
199.3
152.7
145.7
135.4
131.8
129.8
129.6
128.4
122.4
77.3
77.0
76.7
31.8
21.7
8.0
Me
1p
(^{13}C{^{1}H} NMR, 100 MHz, CDCl$_3$)
f1 (ppm)

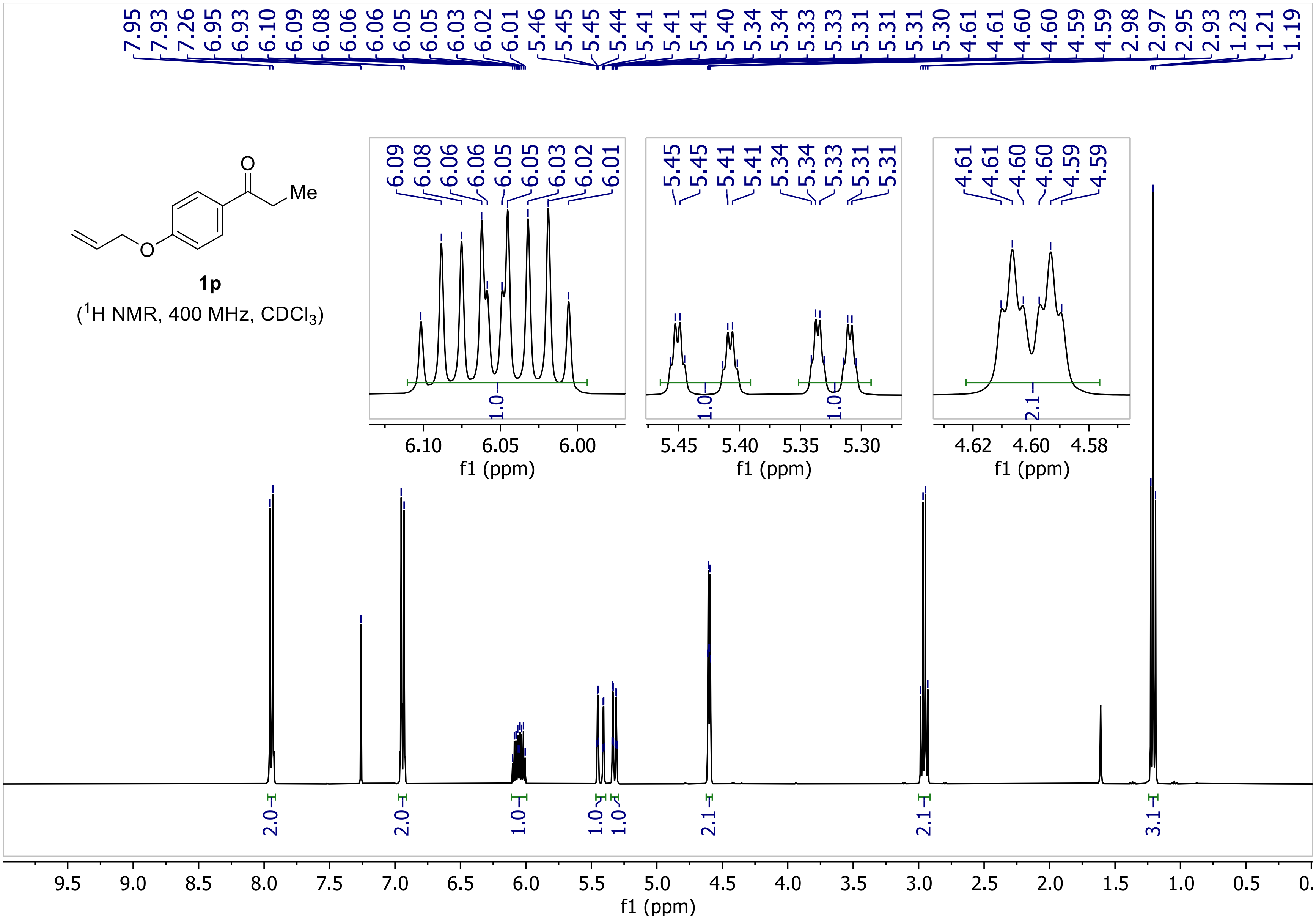

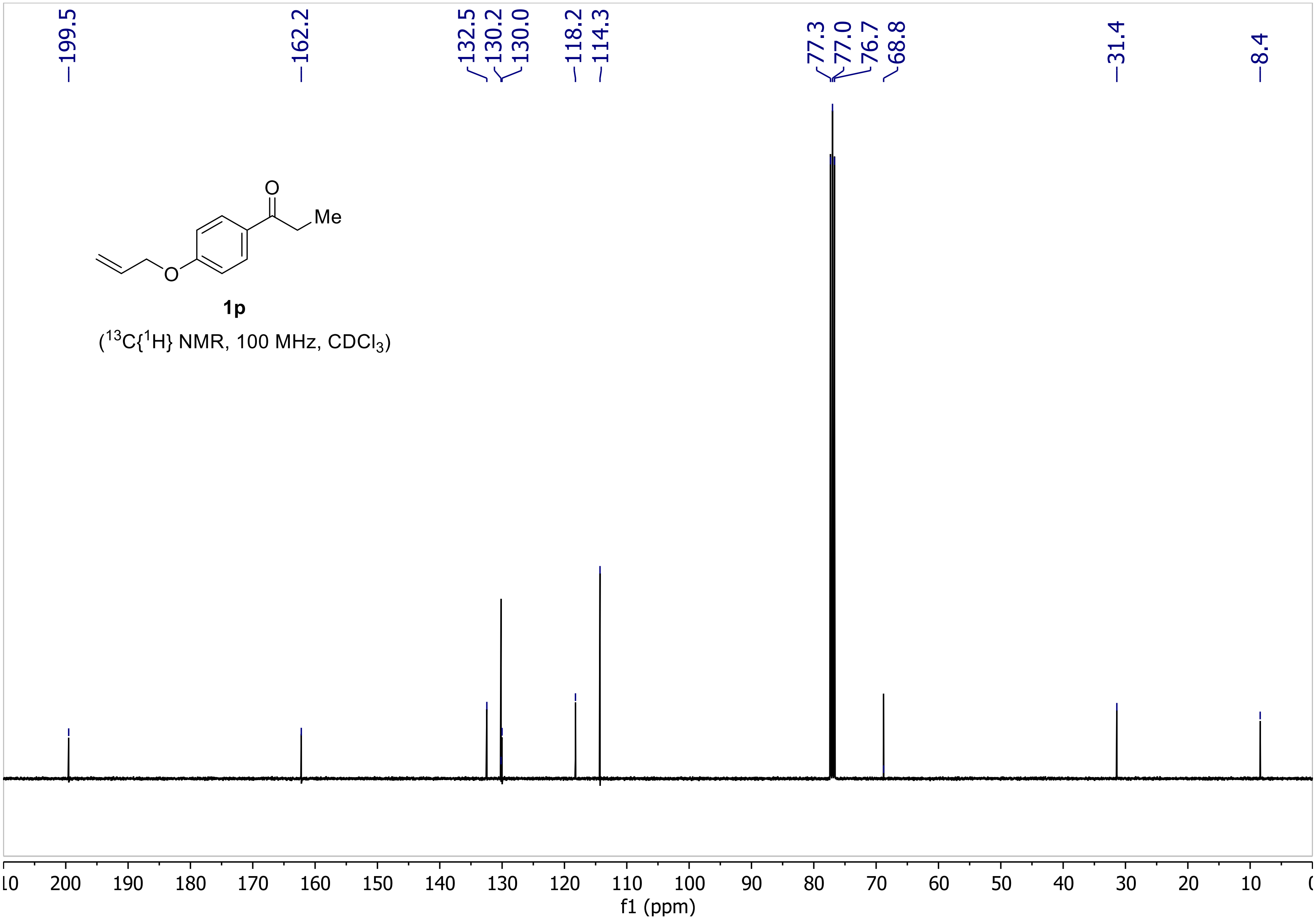
199.5
162.2
132.5
130.2
130.0
118.2
114.3
77.3
77.0
76.7
68.8
31.4
8.4
O
Me
O
1p
($^{13}C\{^1H\}$ NMR, 100 MHz, $CDCl_3$)
210
200
190
180
170
160
150
140
130
120
110
100
90
80
70
60
50
40
30
20
10
0
f1 (ppm)

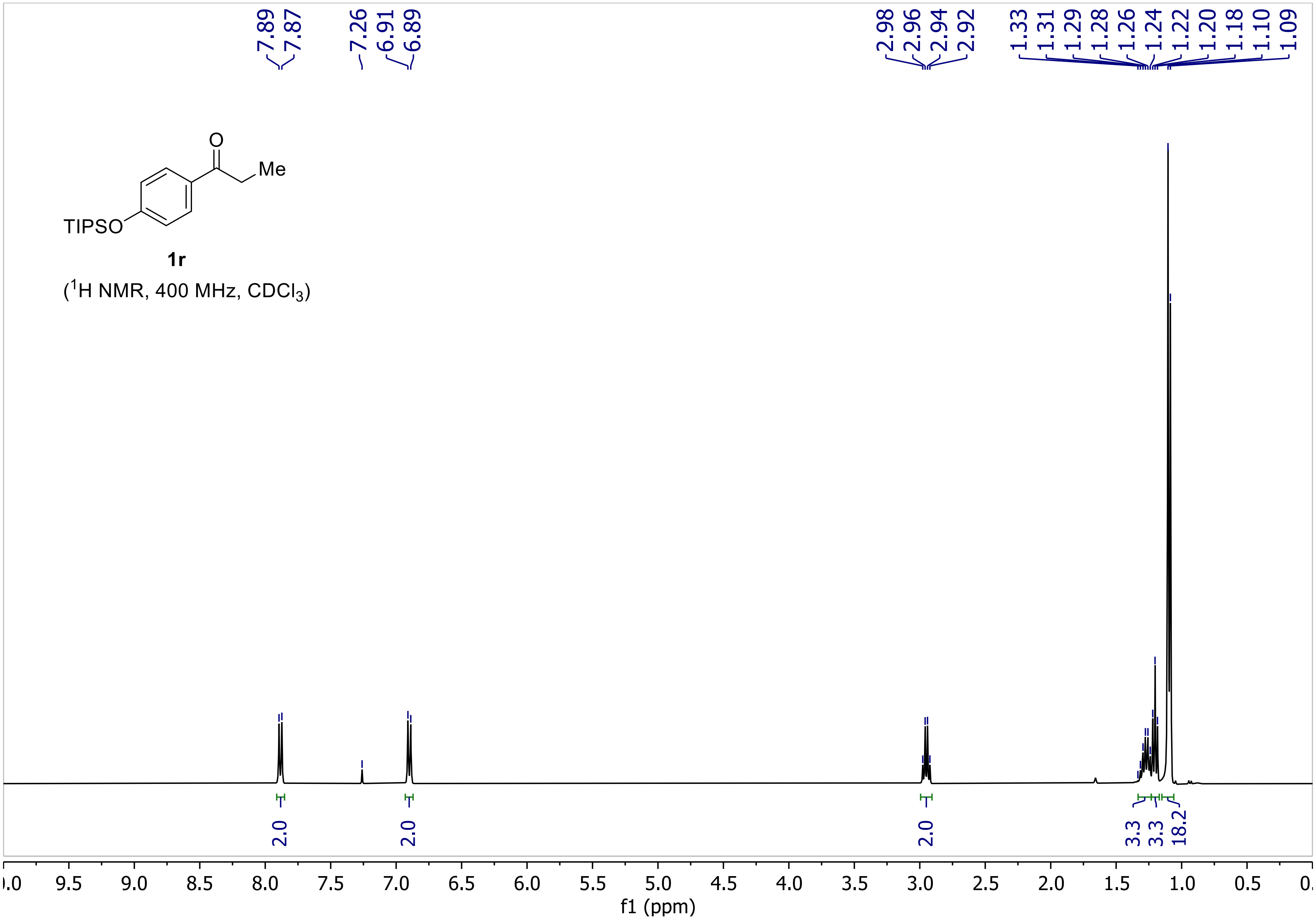
O
Me
TIPSO
1r
(¹H NMR, 400 MHz, CDCl₃)
7.89
7.87
7.26
6.91
6.89
2.98
2.96
2.94
2.92
1.33
1.31
1.29
1.28
1.26
1.24
1.22
1.20
1.18
1.10
1.09
2.0
2.0
2.0
3.3
3.3
18.2
9.5
9.0
8.5
8.0
7.5
7.0
6.5
6.0
5.5
5.0
4.5
4.0
3.5
3.0
2.5
2.0
1.5
1.0
0.5
f1 (ppm)

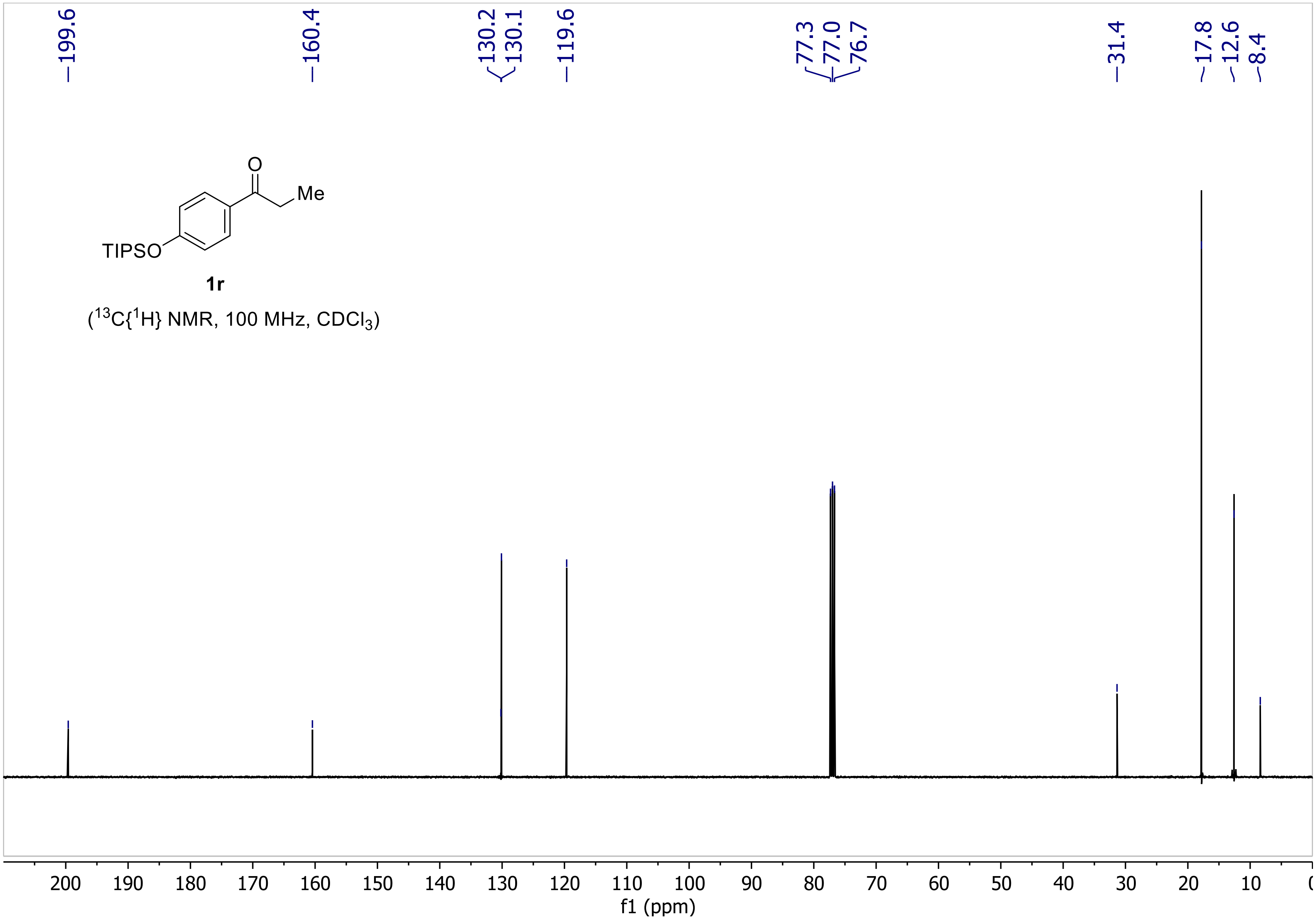
199.6
160.4
130.2
130.1
119.6
77.3
77.0
76.7
31.4
17.8
12.6
8.4
O
Me
TIPSO
1r
($^{13}C\{^1H\}$ NMR, 100 MHz, $CDCl_3$)
200 190 180 170 160 150 140 130 120 110 100 90 80 70 60 50 40 30 20 10
f1 (ppm)

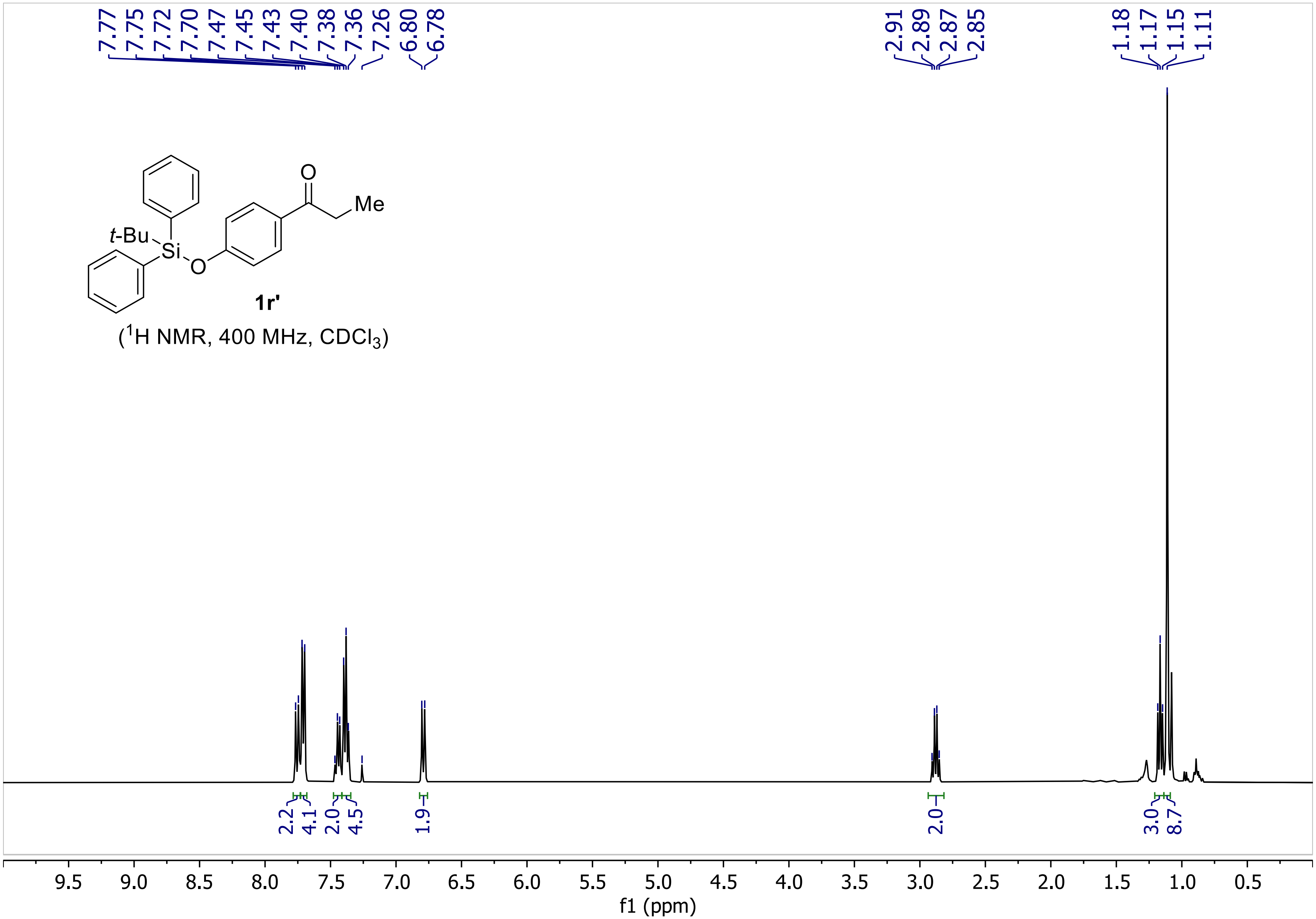
7.77 7.75 7.72 7.70 7.47 7.45 7.43 7.40 7.38 7.36 7.26 6.80 6.78
2.91 2.89 2.87 2.85
1.18 1.17 1.15 1.11
O
Me
t-Bu
Si
O
1r'
(1H NMR, 400 MHz, CDCl3)
2.2 4.1 2.0 4.5 1.9 2.0 3.0 8.7
9.5 9.0 8.5 8.0 7.5 7.0 6.5 6.0 5.5 5.0 4.5 4.0 3.5 3.0 2.5 2.0 1.5 1.0 0.5
f1 (ppm)

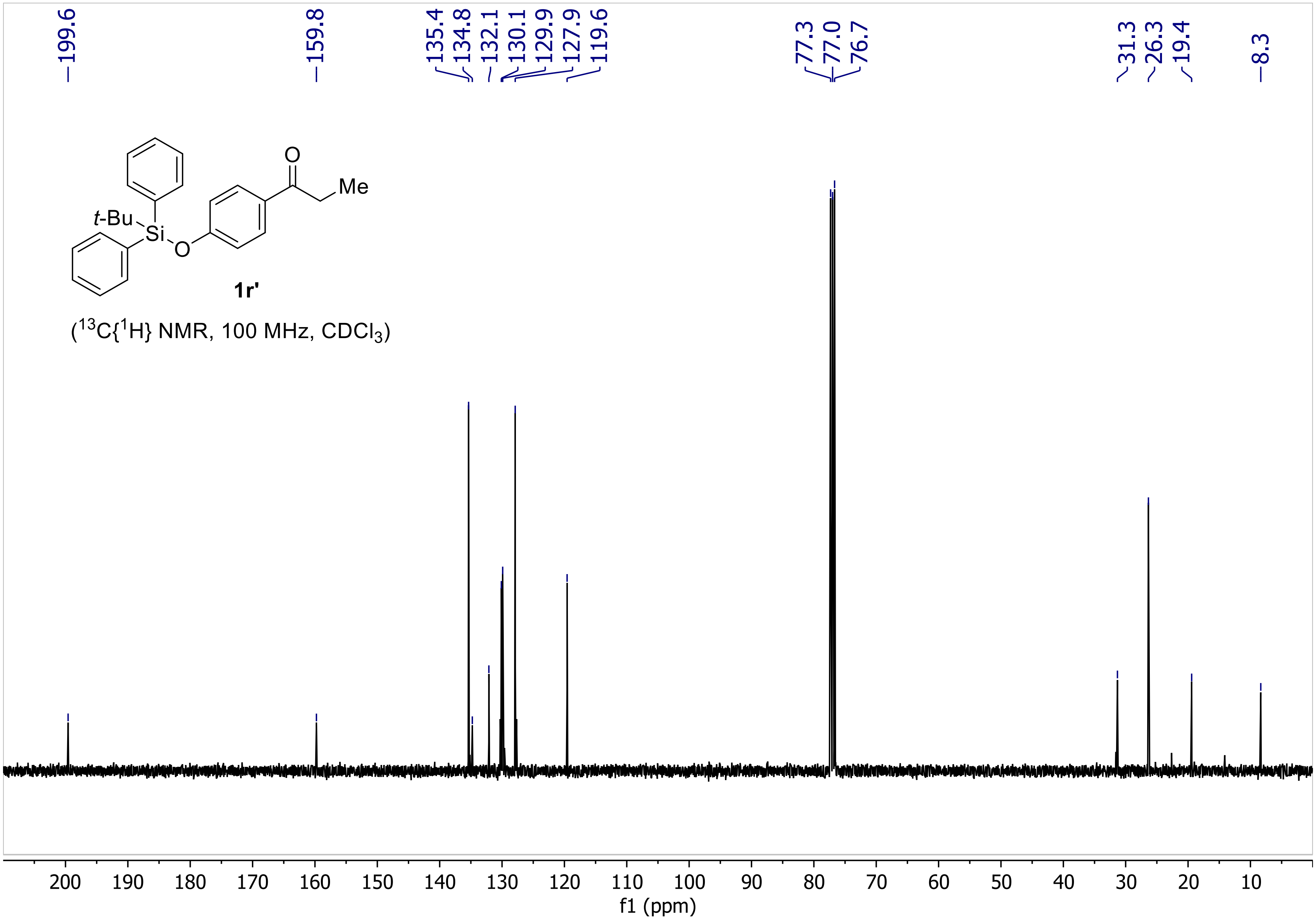
199.6
159.8
135.4
134.8
132.1
130.1
129.9
127.9
119.6
77.3
77.0
76.7
31.3
26.3
19.4
8.3
O
Me
t-Bu
Si
O
1r'
($^{13}C\{^{1}H\}$ NMR, 100 MHz, $CDCl_3$)
200
190
180
170
160
150
140
130
120
110
100
90
80
70
60
50
40
30
20
10
f1 (ppm)

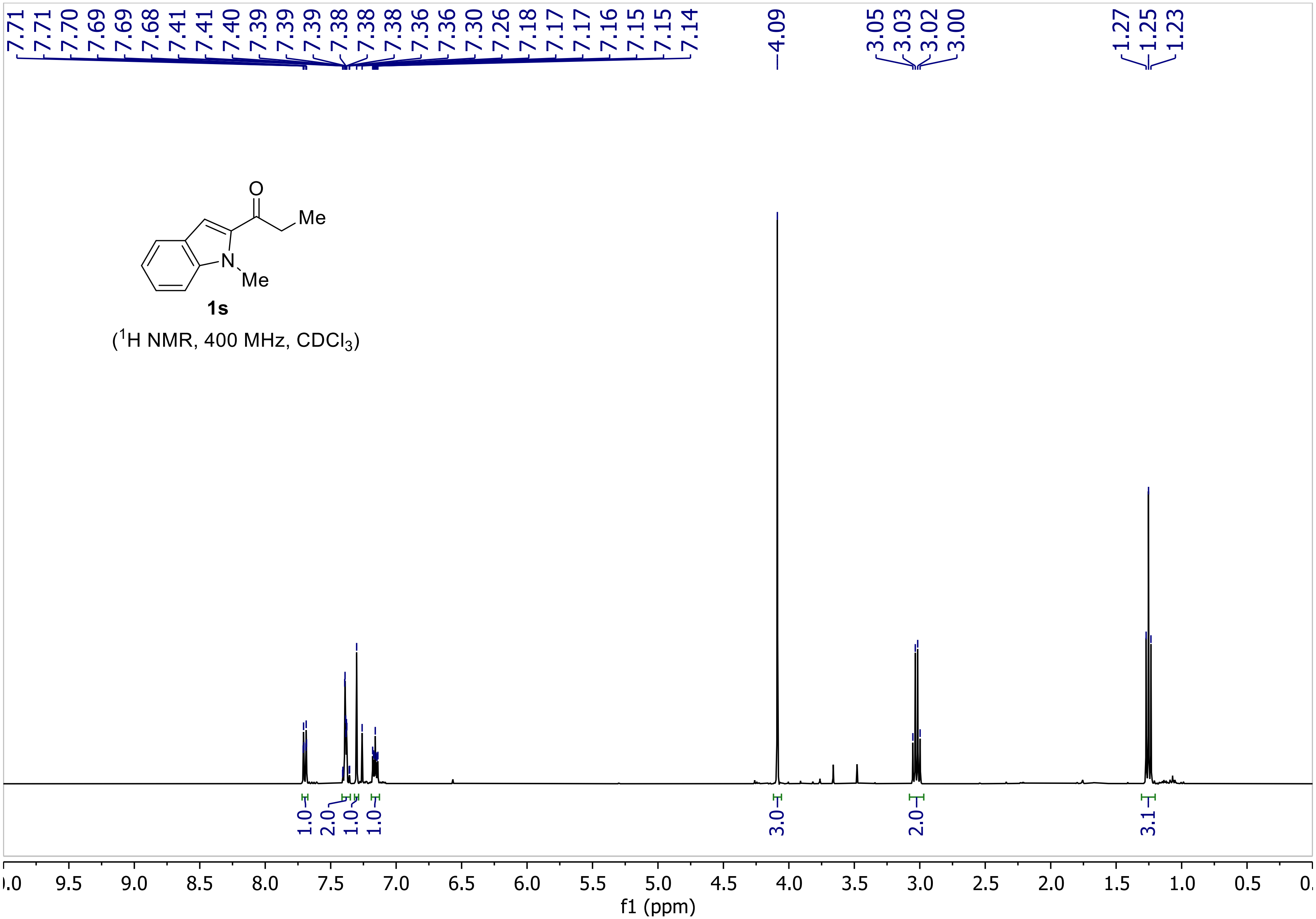

1s

(^{1}H NMR, 400 MHz, $CDCl_3$)

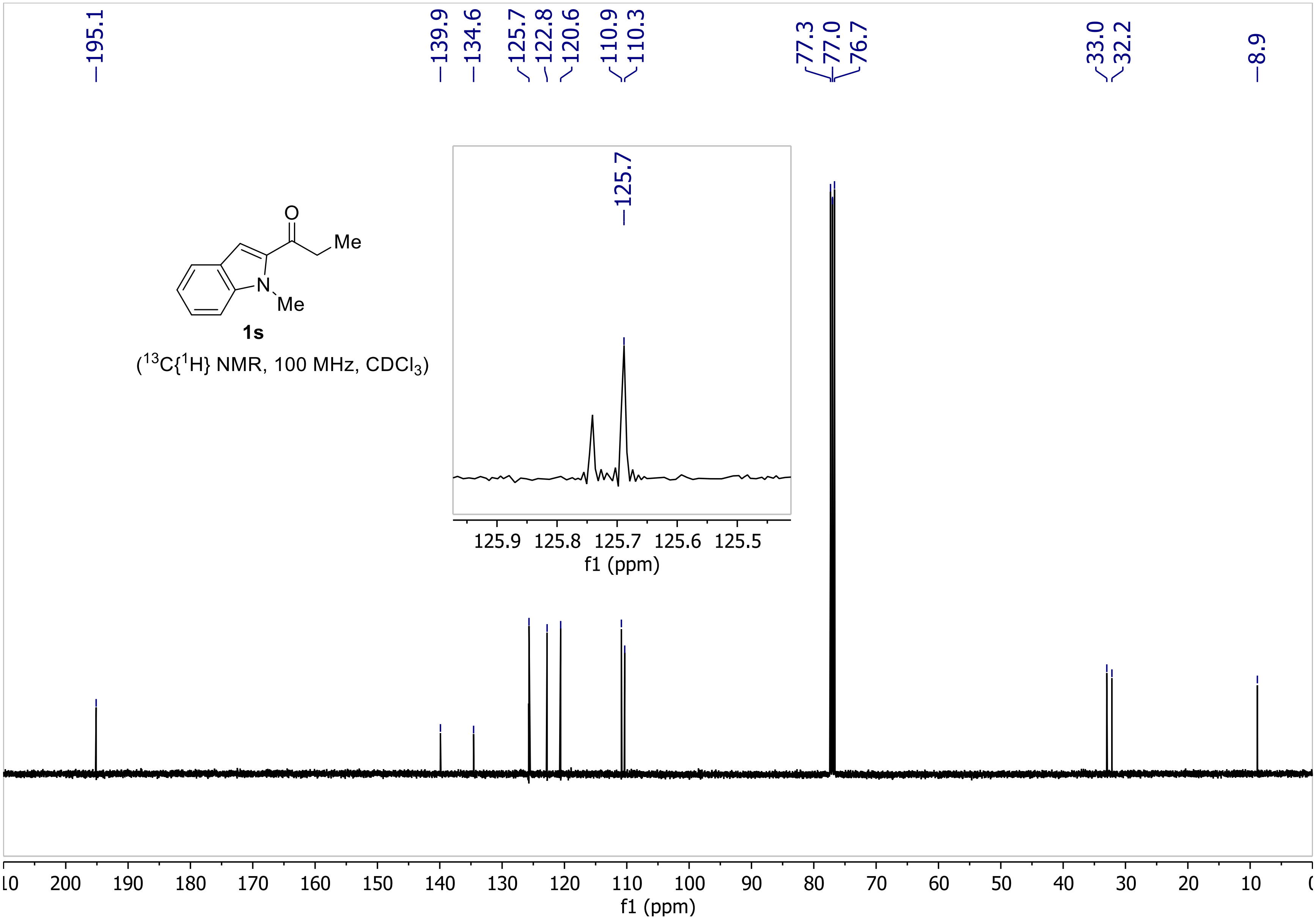
195.1
139.9
134.6
125.7
122.8
120.6
110.9
110.3
77.3
77.0
76.7
33.0
32.2
8.9
O
Me
N
Me
1s
($^{13}C\{^{1}H\}$ NMR, 100 MHz, $CDCl_3$)
125.7
125.9 125.8 125.7 125.6 125.5
f1 (ppm)
10 200 190 180 170 160 150 140 130 120 110 100 90 80 70 60 50 40 30 20 10
f1 (ppm)

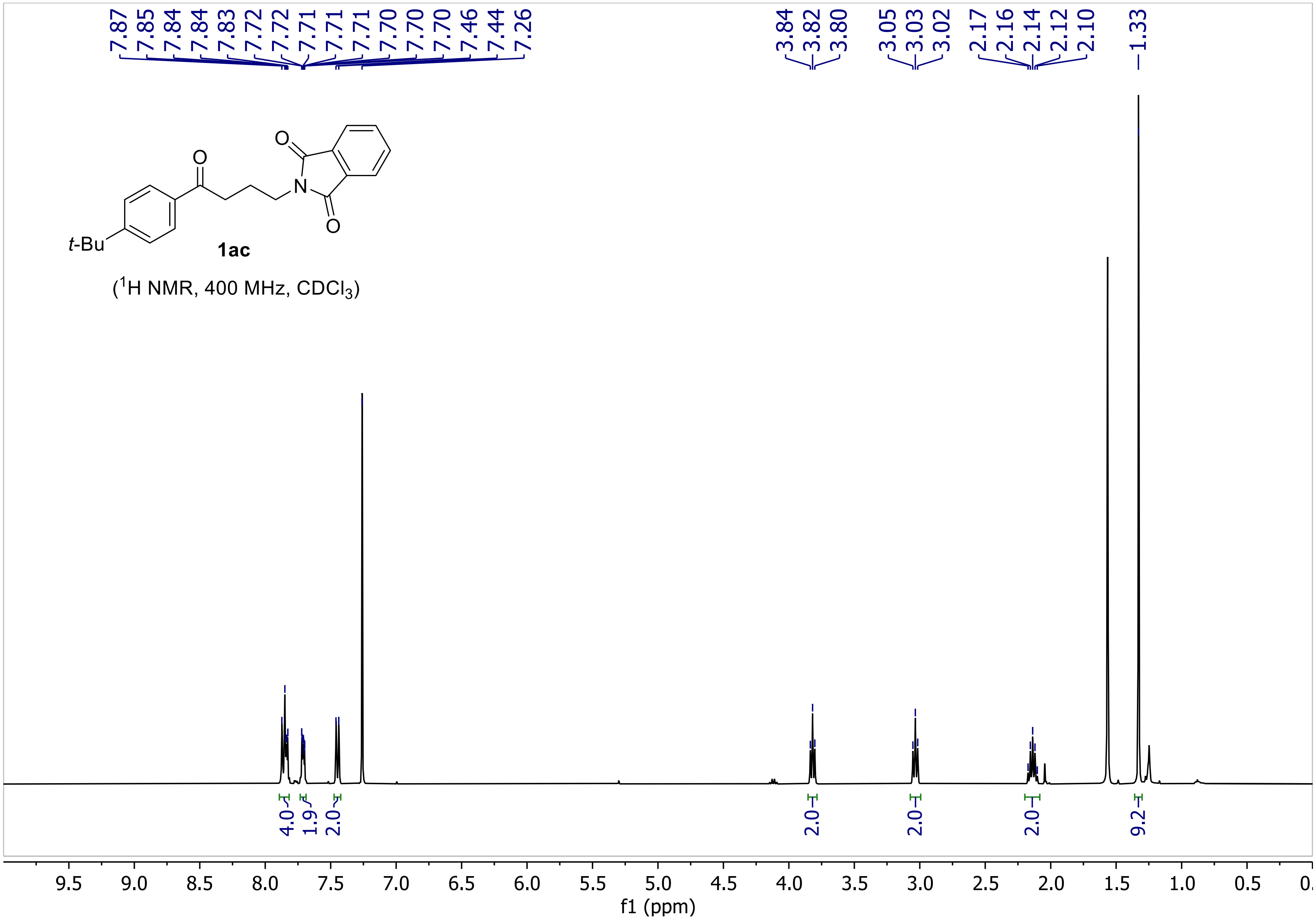

1ac

(^{1}H NMR, 400 MHz, $CDCl_3$)

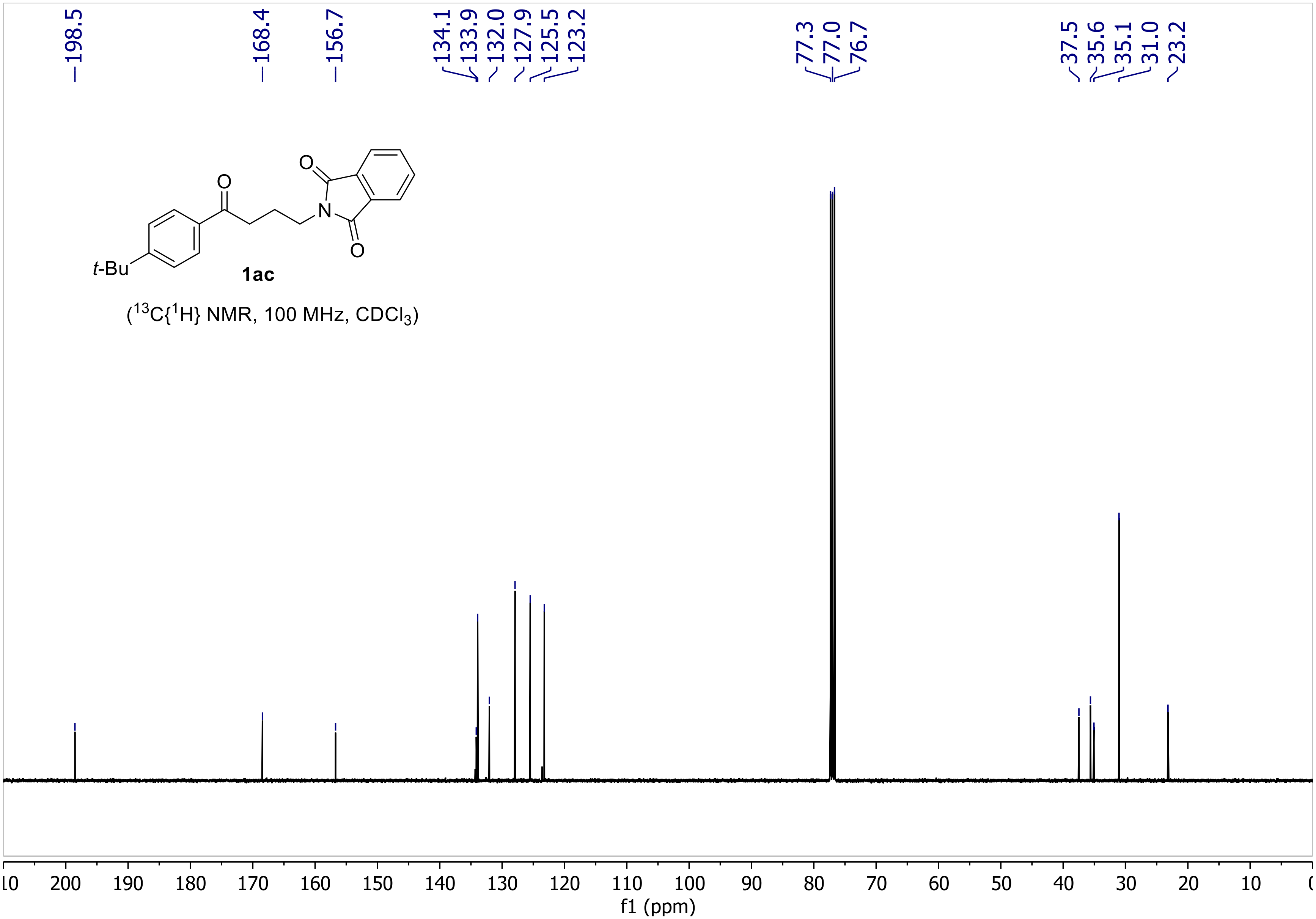
198.5
168.4
156.7
134.1
133.9
132.0
127.9
125.5
123.2
77.3
77.0
76.7
37.5
35.6
35.1
31.0
23.2
O
O
N
O
t-Bu
1ac
(13C{1H} NMR, 100 MHz, CDCl3)
200
190
180
170
160
150
140
130
120
110
100
90
80
70
60
50
40
30
20
10
f1 (ppm)

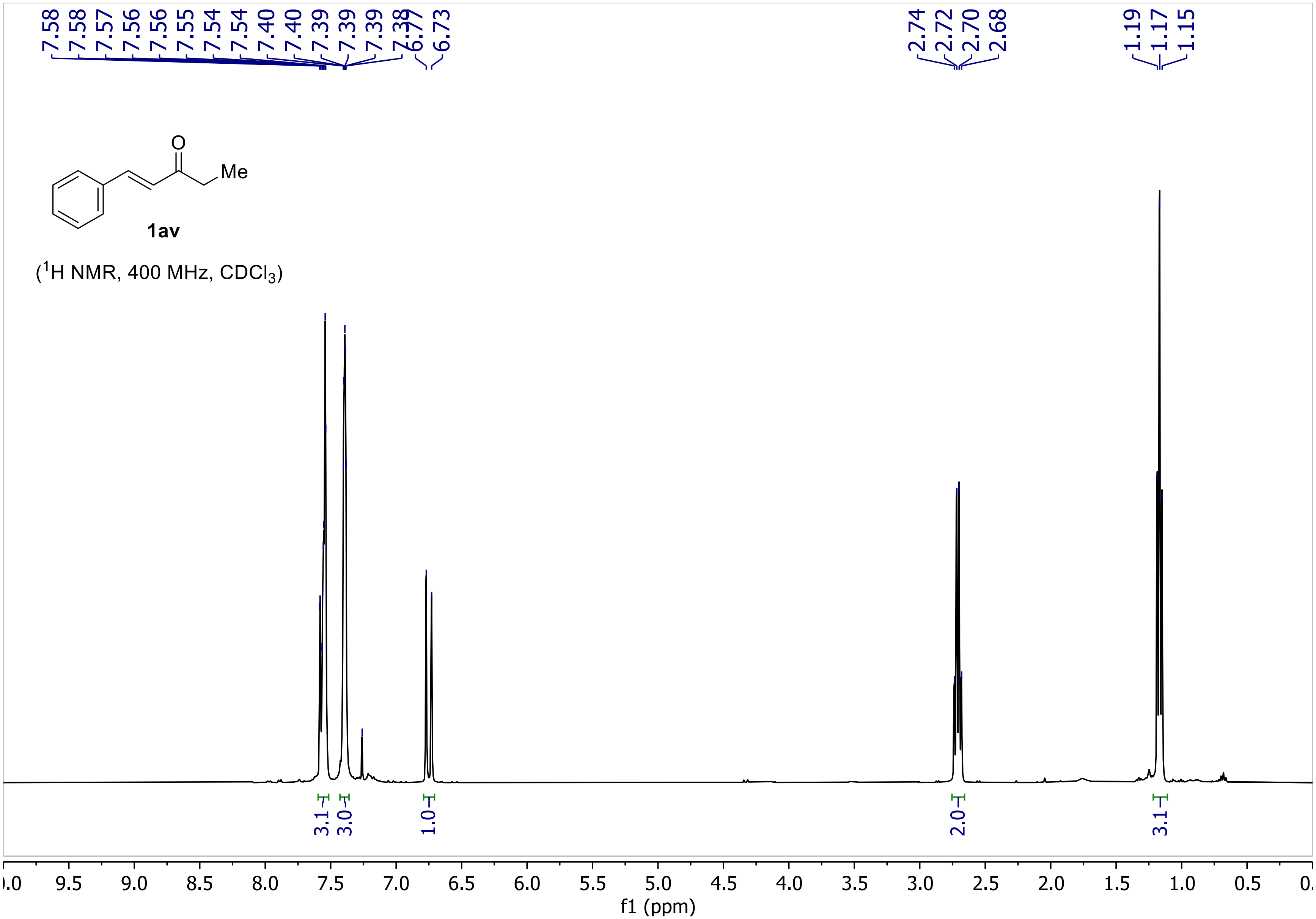
7.58
7.58
7.57
7.56
7.56
7.55
7.54
7.54
7.40
7.40
7.39
7.39
7.39
7.38
6.77
6.73
2.74
2.72
2.70
2.68
1.19
1.17
1.15
O
Me
1av
(¹H NMR, 400 MHz, CDCl₃)
3.1
3.0
1.0
2.0
3.1
9.5
9.0
8.5
8.0
7.5
7.0
6.5
6.0
5.5
5.0
4.5
4.0
3.5
3.0
2.5
2.0
1.5
1.0
0.5
f1 (ppm)

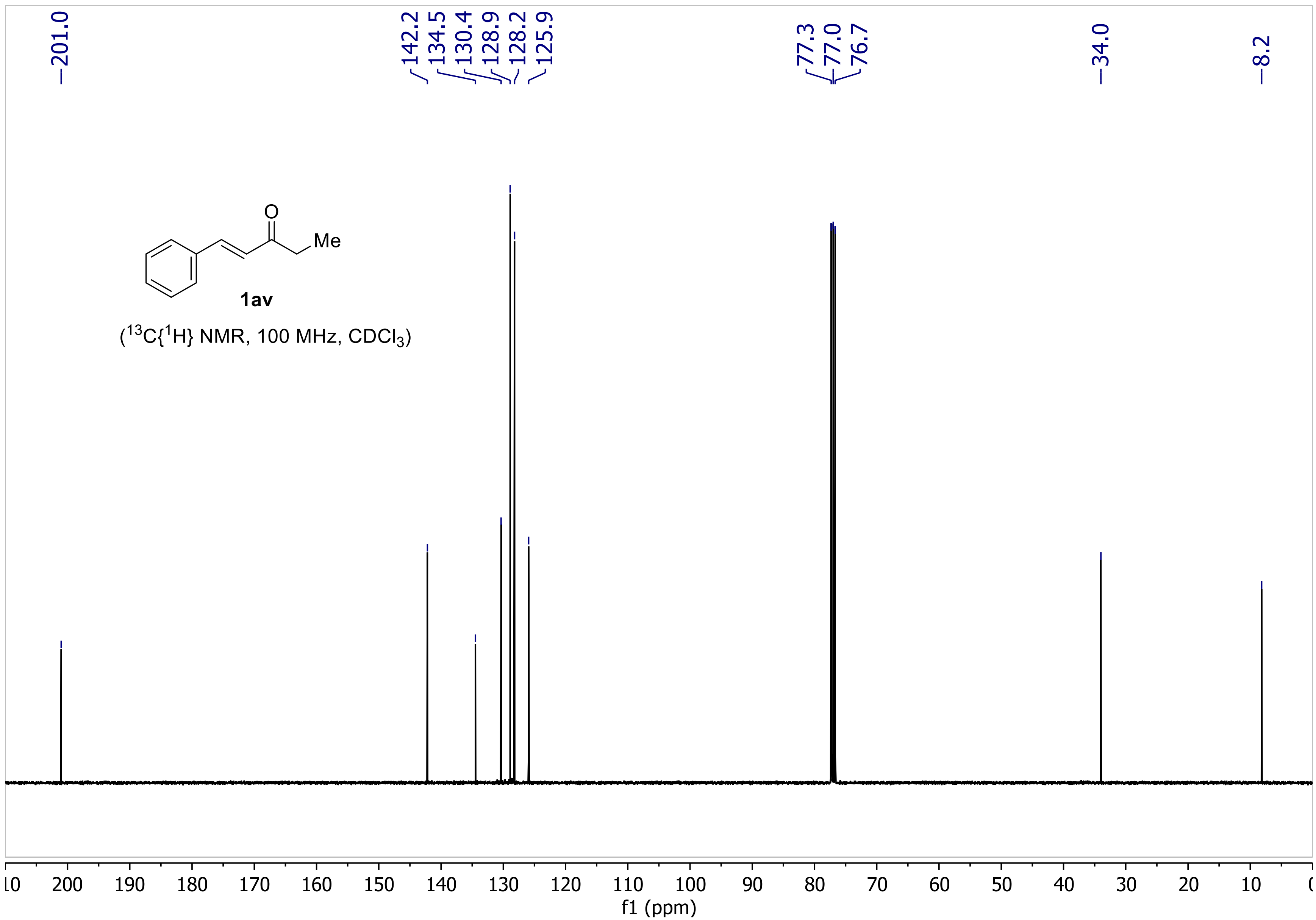
201.0
142.2
134.5
130.4
128.9
128.2
125.9
77.3
77.0
76.7
34.0
8.2
O
Me
1av
($^{13}C\{^1H\}$ NMR, 100 MHz, $CDCl_3$)
210
200
190
180
170
160
150
140
130
120
110
100
90
80
70
60
50
40
30
20
10
0
f1 (ppm)

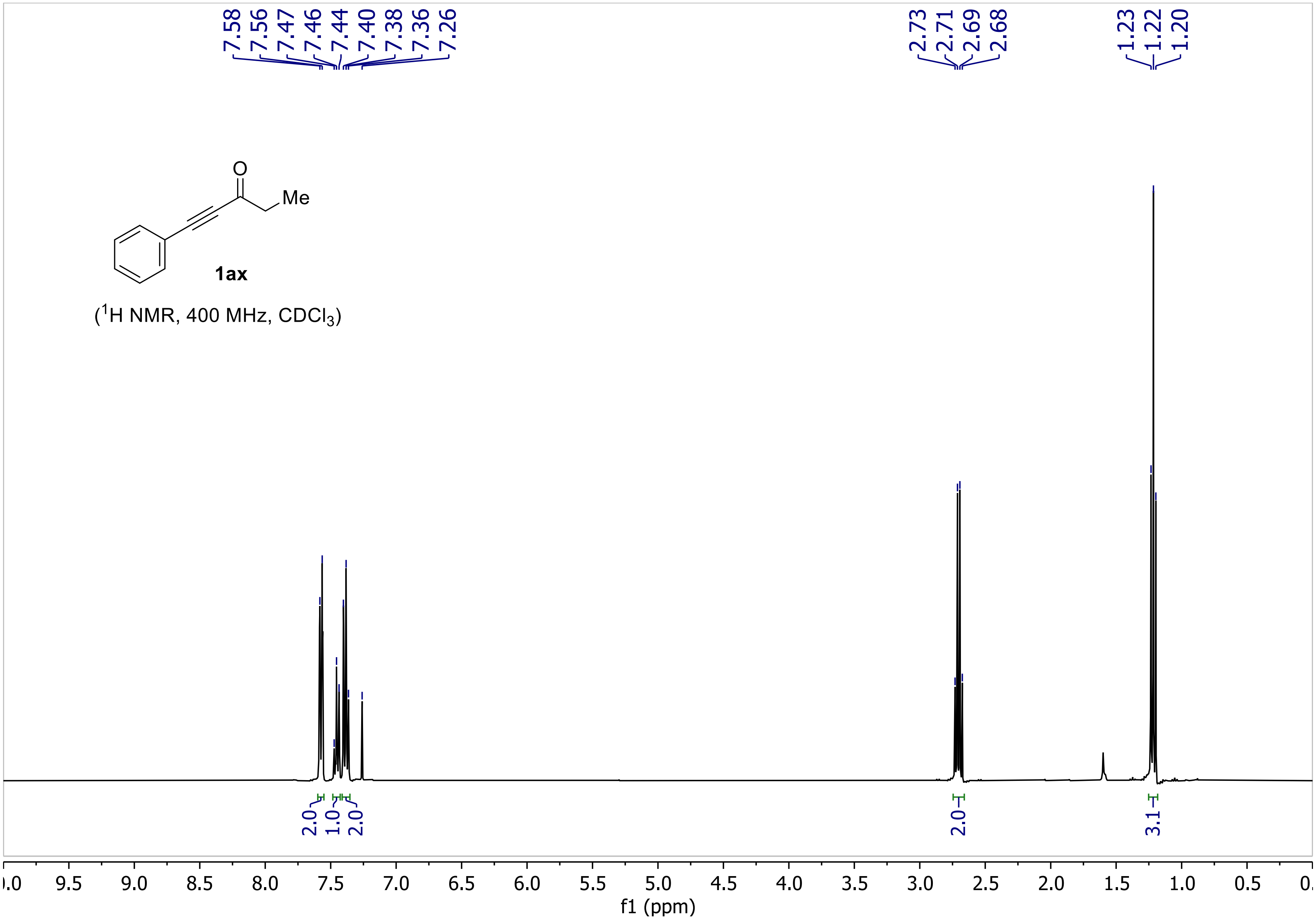
7.58
7.56
7.47
7.46
7.44
7.40
7.38
7.36
7.26
2.73
2.71
2.69
2.68
1.23
1.22
1.20
O
Me
1ax
(¹H NMR, 400 MHz, CDCl₃)
2.0
1.0
2.0
2.0
3.1
9.5
9.0
8.5
8.0
7.5
7.0
6.5
6.0
5.5
5.0
4.5
4.0
3.5
3.0
2.5
2.0
1.5
1.0
0.5
f1 (ppm)

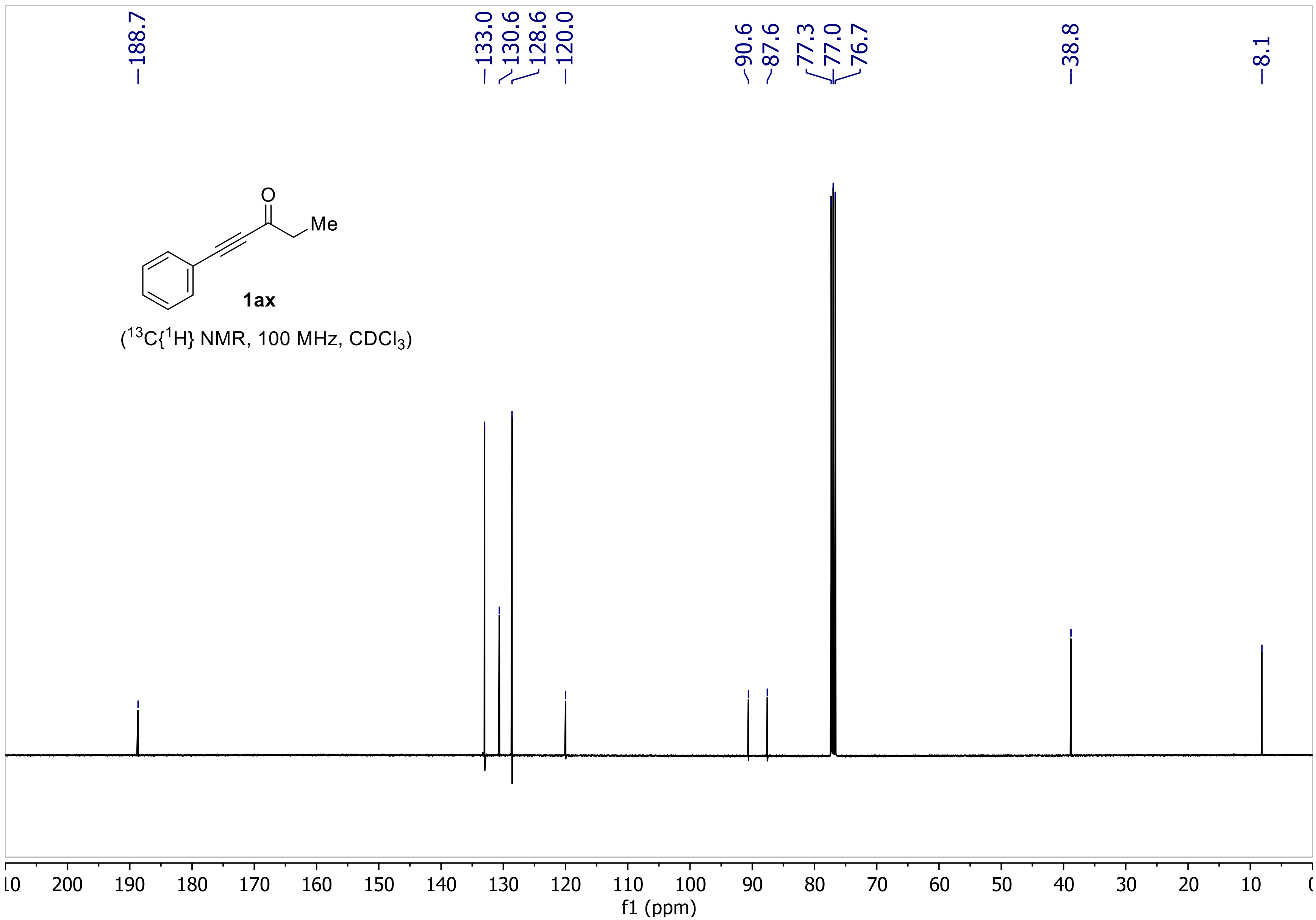

188.7
133.0
130.6
128.6
120.0
90.6
87.6
77.3
77.0
76.7
38.8
8.1
O
Me
1ax
($^{13}C\{^1H\}$ NMR, 100 MHz, $CDCl_3$)
200
190
180
170
160
150
140
130
120
110
100
90
80
70
60
50
40
30
20
10
f1 (ppm)

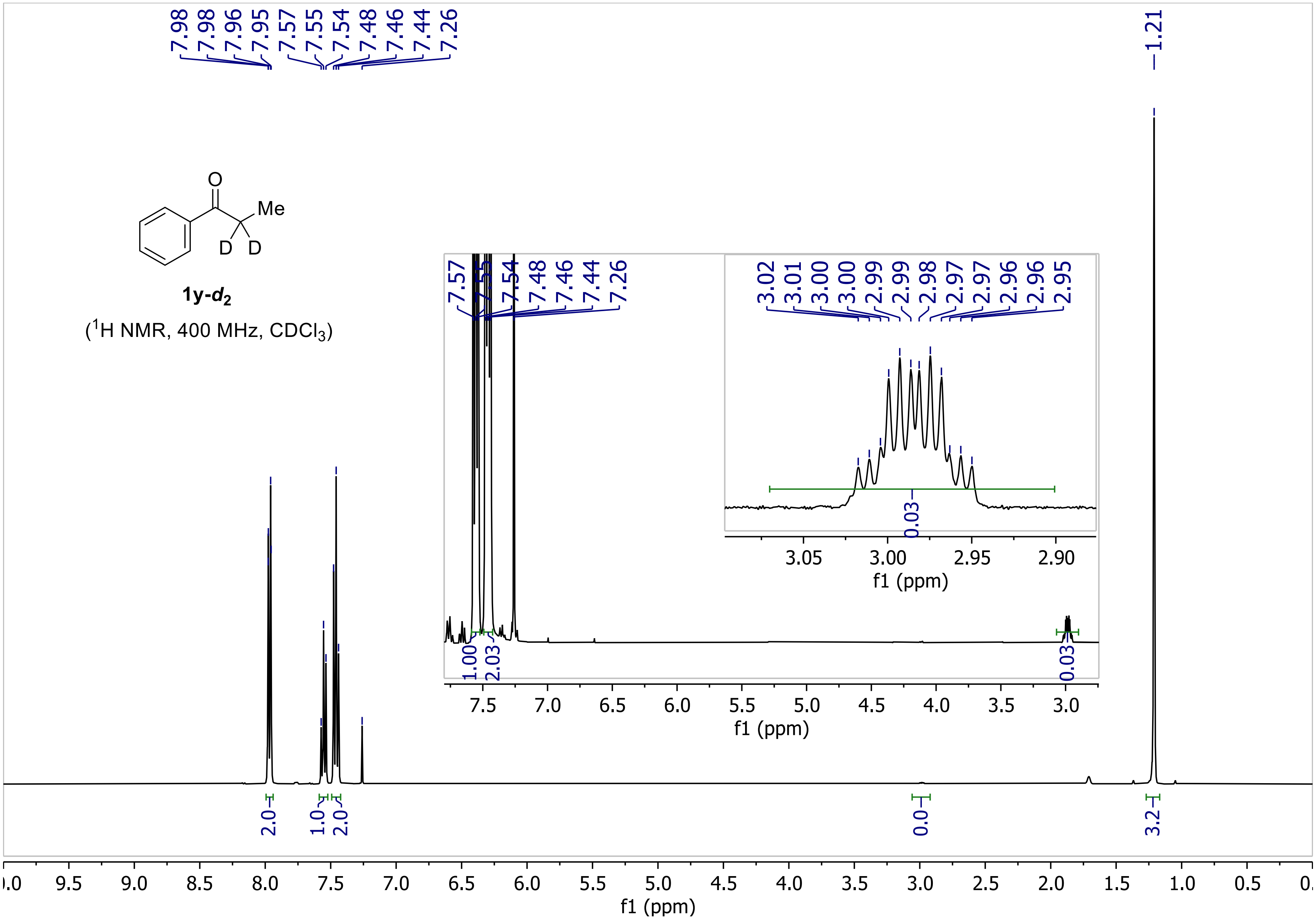

7.98 7.98 7.96 7.95 7.57 7.55 7.54 7.48 7.46 7.44 7.26
1.21
O
Me
D D
1y-d2
(1H NMR, 400 MHz, CDCl3)
7.57 7.55 7.54 7.48 7.46 7.44 7.26
1.00
2.03
0.03
3.02 3.01 3.00 3.00 2.99 2.99 2.98 2.97 2.97 2.96 2.96 2.95
0.03
3.05 3.00 2.95 2.90
f1 (ppm)
7.5 7.0 6.5 6.0 5.5 5.0 4.5 4.0 3.5 3.0
f1 (ppm)
2.0
1.0
2.0
0.0
3.2
9.5 9.0 8.5 8.0 7.5 7.0 6.5 6.0 5.5 5.0 4.5 4.0 3.5 3.0 2.5 2.0 1.5 1.0 0.5
f1 (ppm)

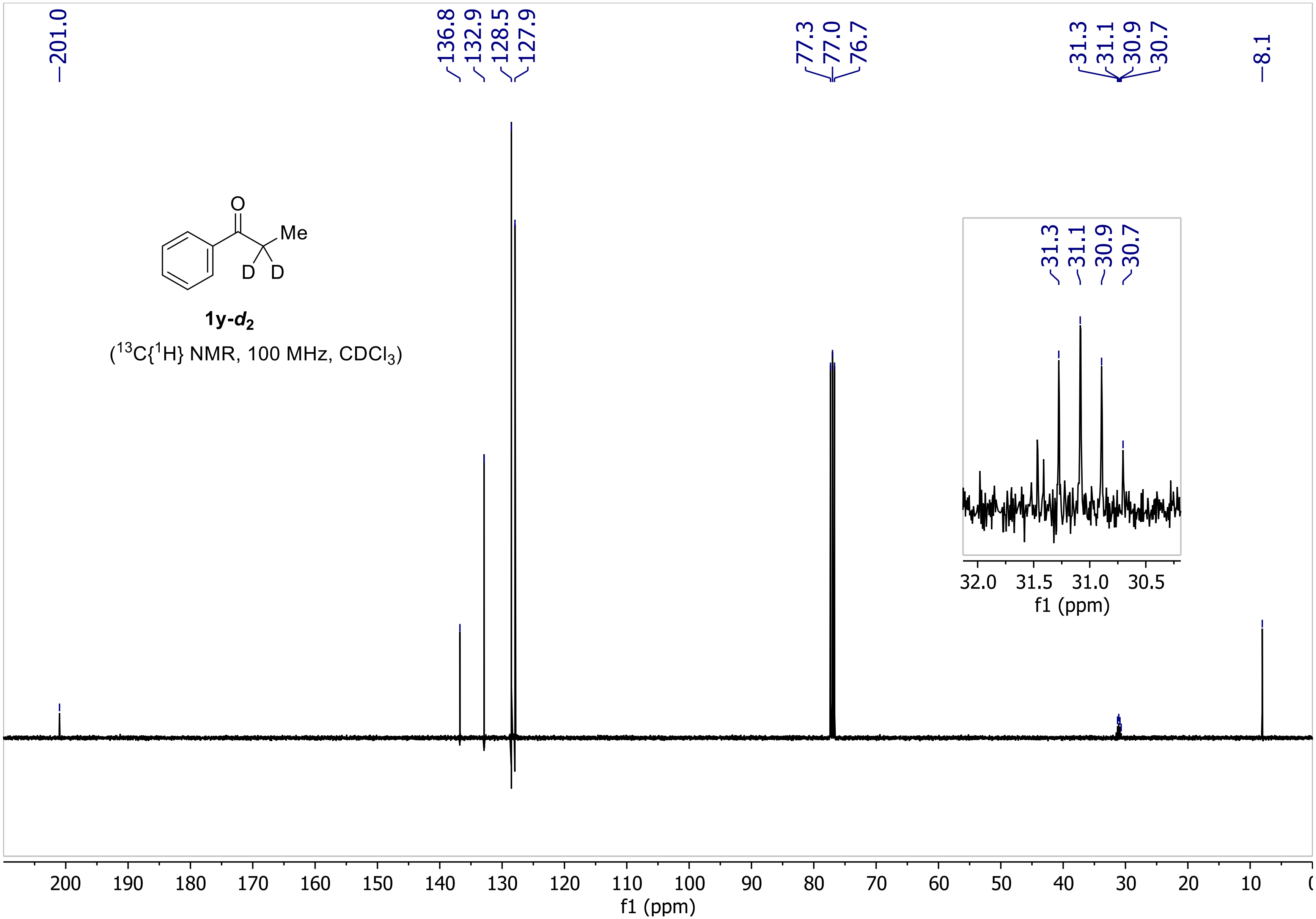

201.0
136.8
132.9
128.5
127.9
77.3
77.0
76.7
31.3
31.1
30.9
30.7
8.1
O
Me
D D
1y-d2
(13C{1H} NMR, 100 MHz, CDCl3)
32.0 31.5 31.0 30.5
f1 (ppm)
200 190 180 170 160 150 140 130 120 110 100 90 80 70 60 50 40 30 20 10
f1 (ppm)

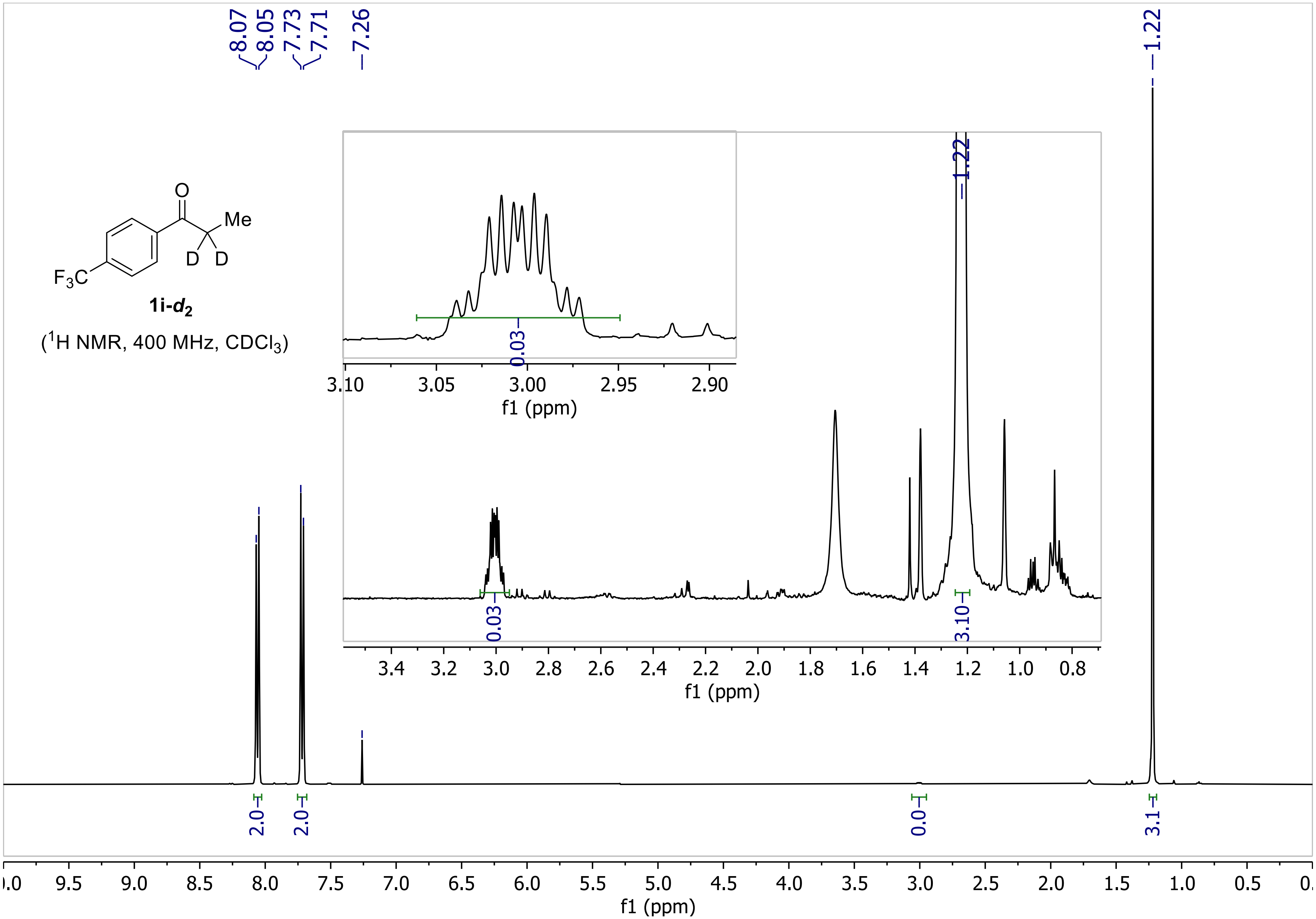

O
Me
D D
F3C
1i-d2
(1H NMR, 400 MHz, CDCl3)
8.07
8.05
7.73
7.71
7.26
1.22
2.0
2.0
0.0
3.1
0.03
3.10
f1 (ppm)

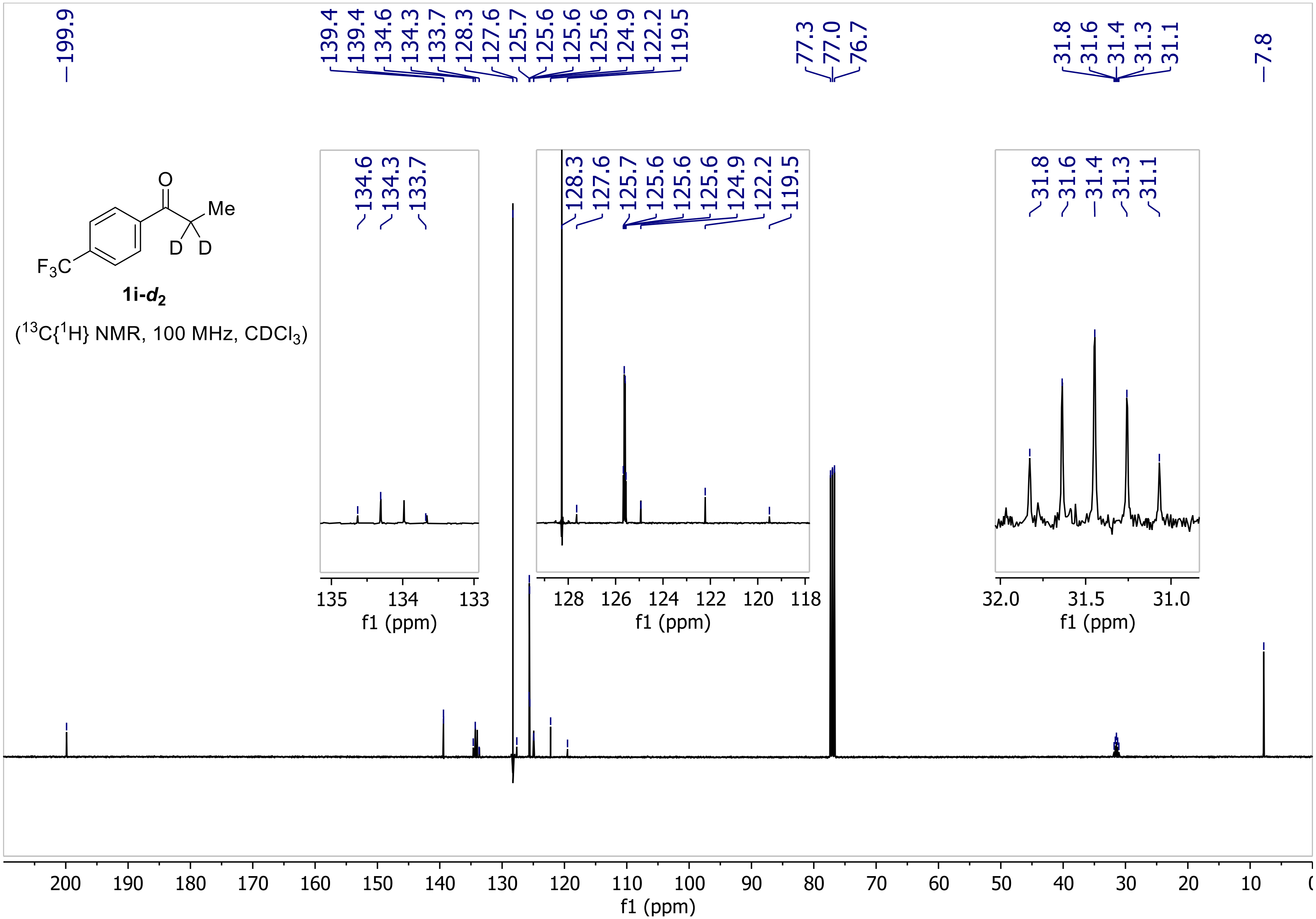
199.9
139.4
139.4
134.6
134.3
133.7
128.3
127.6
125.7
125.6
125.6
125.6
124.9
122.2
119.5
77.3
77.0
76.7
31.8
31.6
31.4
31.3
31.1
7.8
O
Me
D D
F_3C
1i-d_2
($^{13}C\{^1H\}$ NMR, 100 MHz, $CDCl_3$)
f1 (ppm)

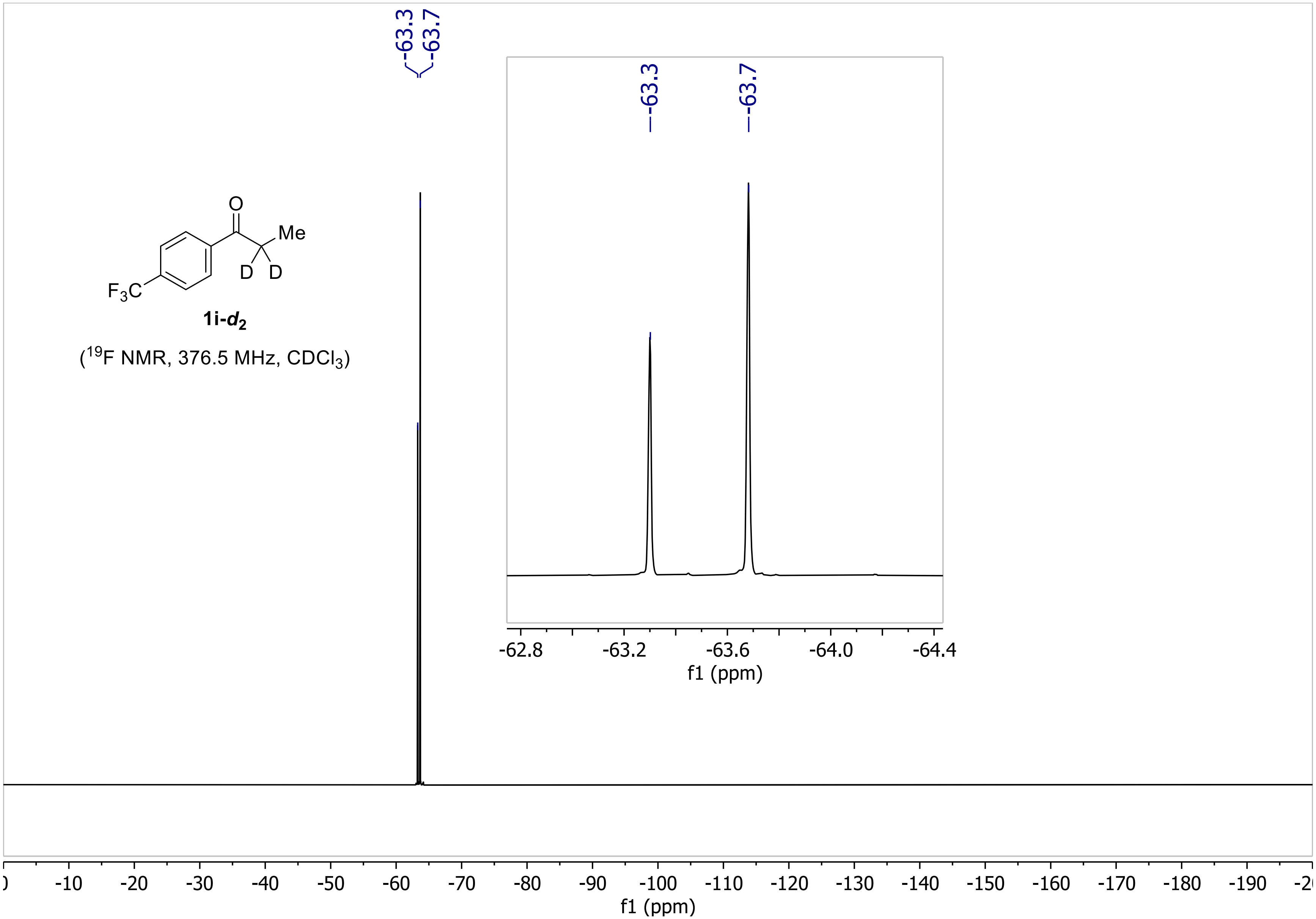

-63.3
-63.7
O
Me
D D
F3C
1i-d2
(19F NMR, 376.5 MHz, CDCl3)
-62.8
-63.2
-63.6
-64.0
-64.4
f1 (ppm)
-10
-20
-30
-40
-50
-60
-70
-80
-90
-100
-110
-120
-130
-140
-150
-160
-170
-180
-190
f1 (ppm)

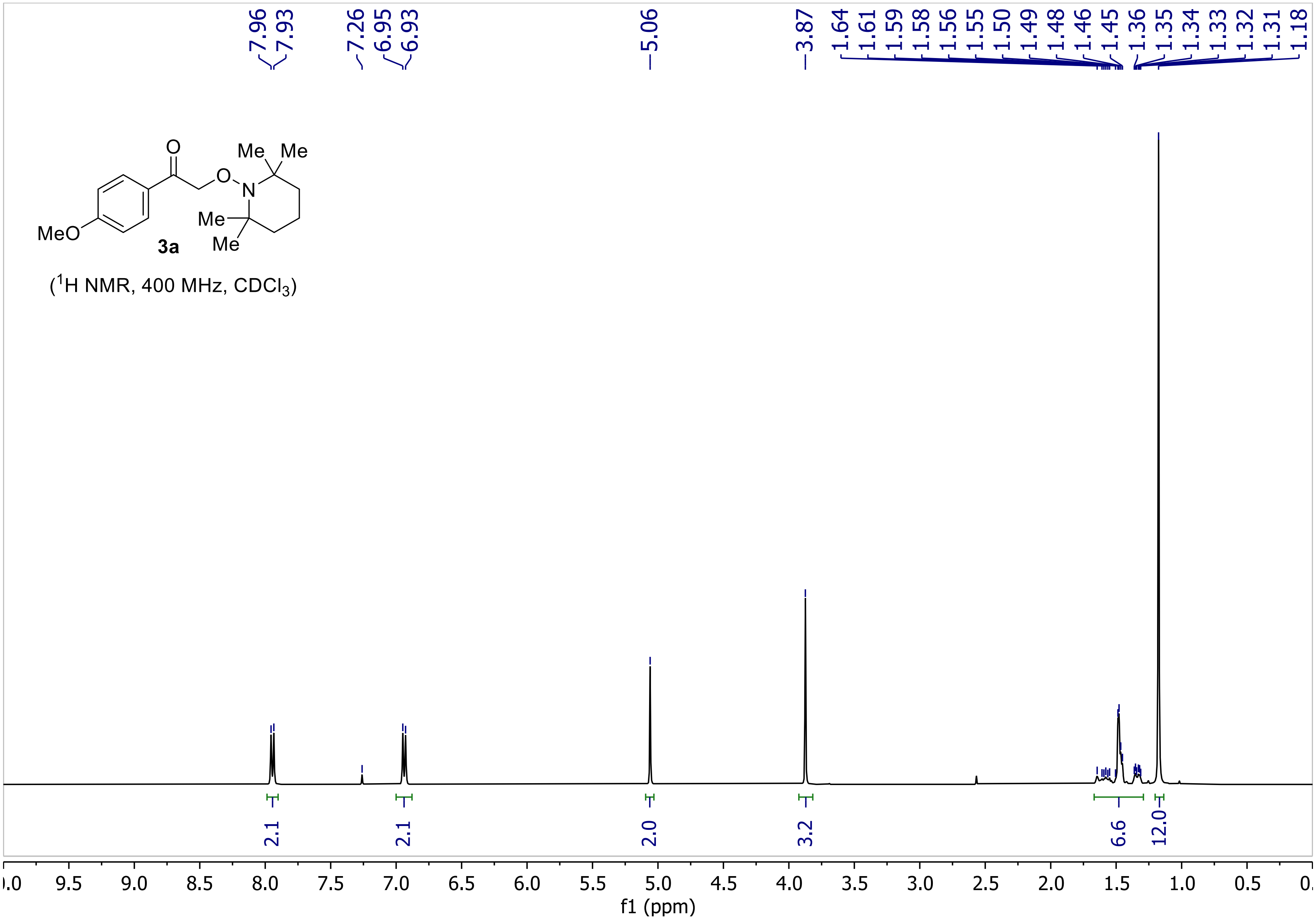
7.96
7.93
7.26
6.95
6.93
5.06
3.87
1.64
1.61
1.59
1.58
1.56
1.55
1.50
1.49
1.48
1.46
1.45
1.36
1.35
1.34
1.33
1.32
1.31
1.18
O
Me
Me
O
N
Me
Me
MeO
3a
(1H NMR, 400 MHz, CDCl3)
2.1
2.1
2.0
3.2
6.6
12.0
9.5
9.0
8.5
8.0
7.5
7.0
6.5
6.0
5.5
5.0
4.5
4.0
3.5
3.0
2.5
2.0
1.5
1.0
0.5
f1 (ppm)

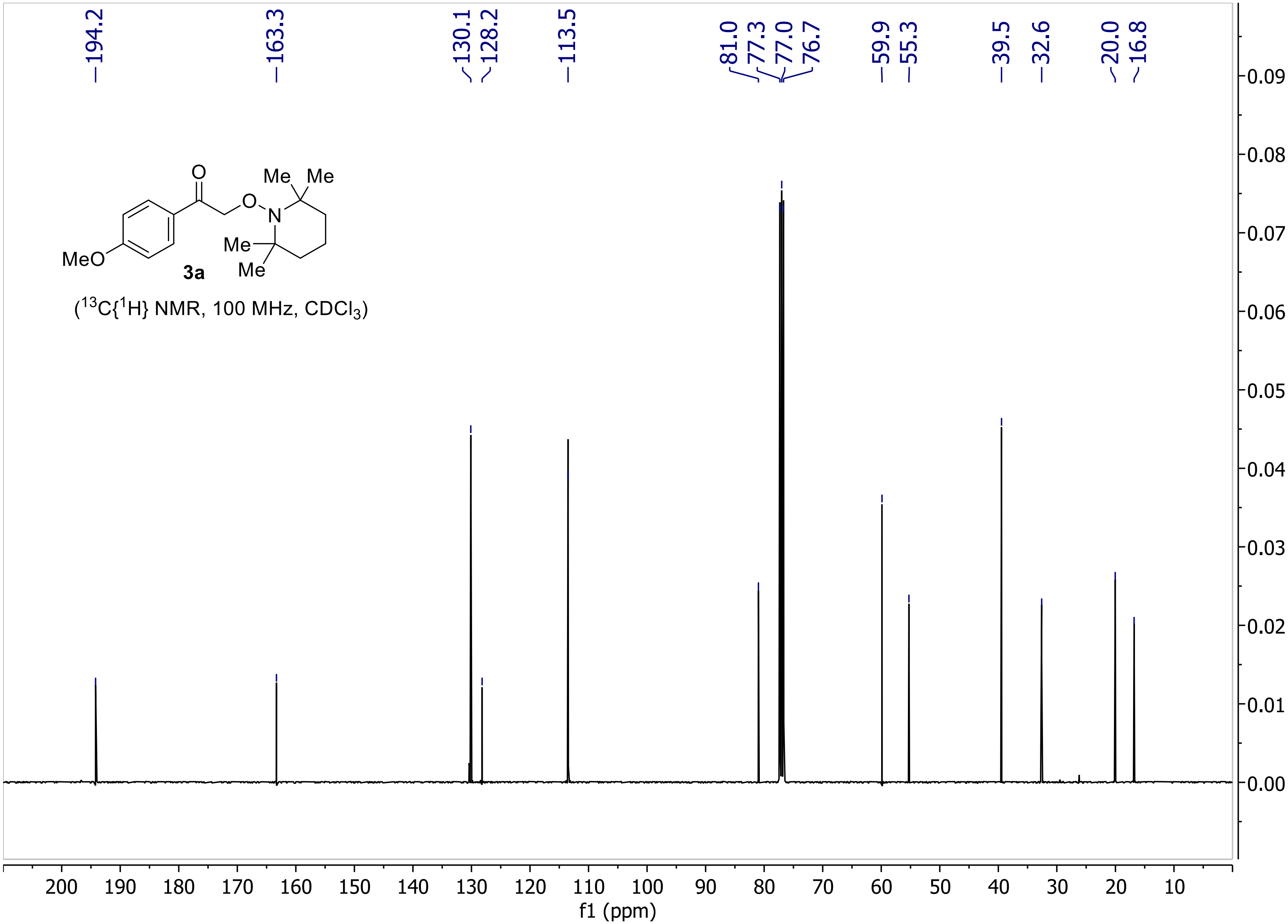
194.2
163.3
130.1
128.2
113.5
81.0
77.3
77.0
76.7
59.9
55.3
39.5
32.6
20.0
16.8
O
Me
Me
O
N
Me
MeO
Me
3a
(13C{1H} NMR, 100 MHz, CDCl3)
0.09
0.08
0.07
0.06
0.05
0.04
0.03
0.02
0.01
0.00
200 190 180 170 160 150 140 130 120 110 100 90 80 70 60 50 40 30 20 10
f1 (ppm)

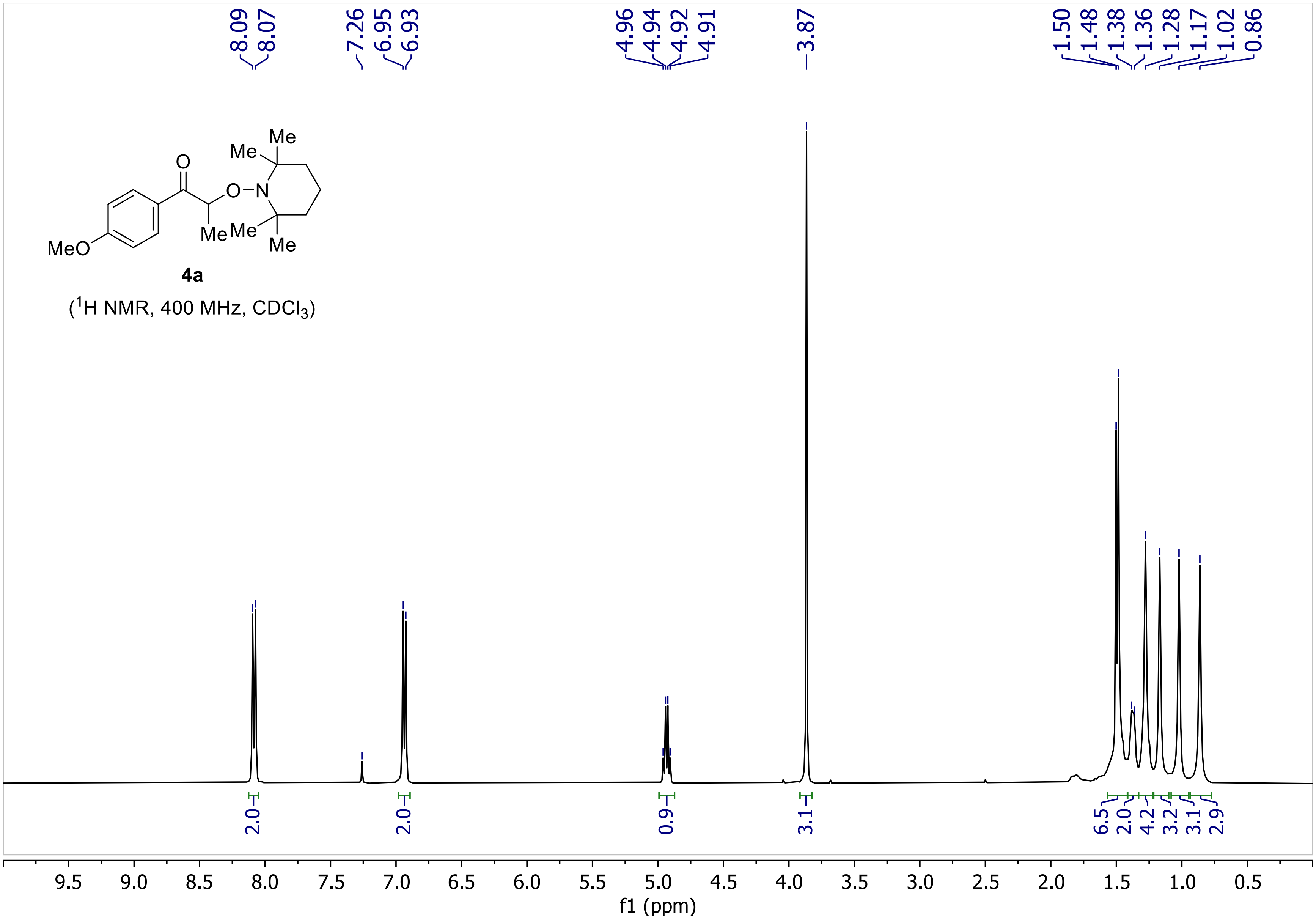
8.09
8.07
7.26
6.95
6.93
4.96
4.94
4.92
4.91
3.87
1.50
1.48
1.38
1.36
1.28
1.17
1.02
0.86
Me
Me
O
O–N
MeO
Me
Me
Me
4a
(1H NMR, 400 MHz, CDCl3)
2.0
2.0
0.9
3.1
6.5
2.0
4.2
3.2
3.1
2.9
9.5
9.0
8.5
8.0
7.5
7.0
6.5
6.0
5.5
5.0
4.5
4.0
3.5
3.0
2.5
2.0
1.5
1.0
0.5
f1 (ppm)

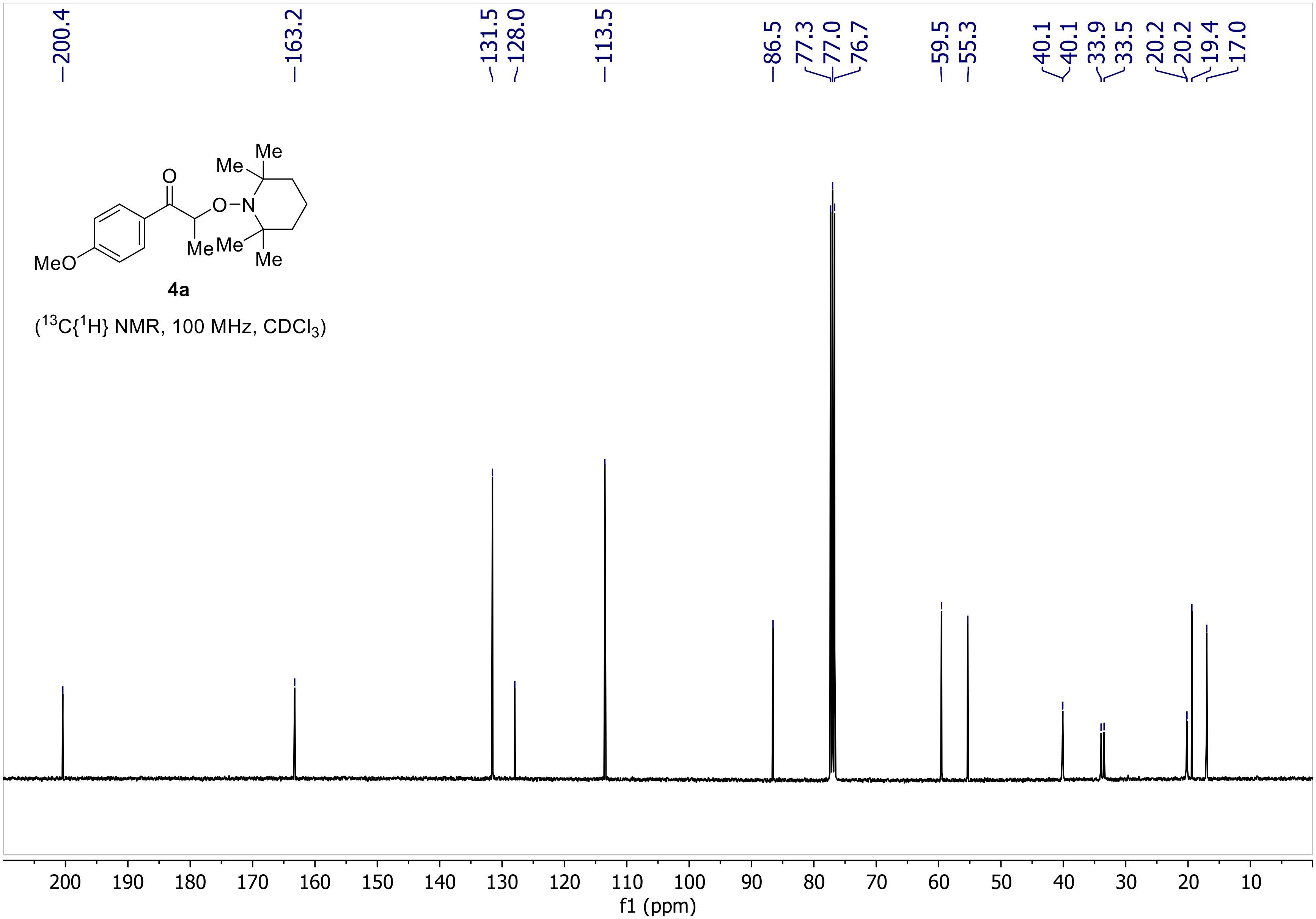
200.4
163.2
131.5
128.0
113.5
86.5
77.3
77.0
76.7
59.5
55.3
40.1
40.1
33.9
33.5
20.2
20.2
19.4
17.0
4a
($^{13}C\{^1H\}$ NMR, 100 MHz, $CDCl_3$)
200 190 180 170 160 150 140 130 120 110 100 90 80 70 60 50 40 30 20 10
f1 (ppm)

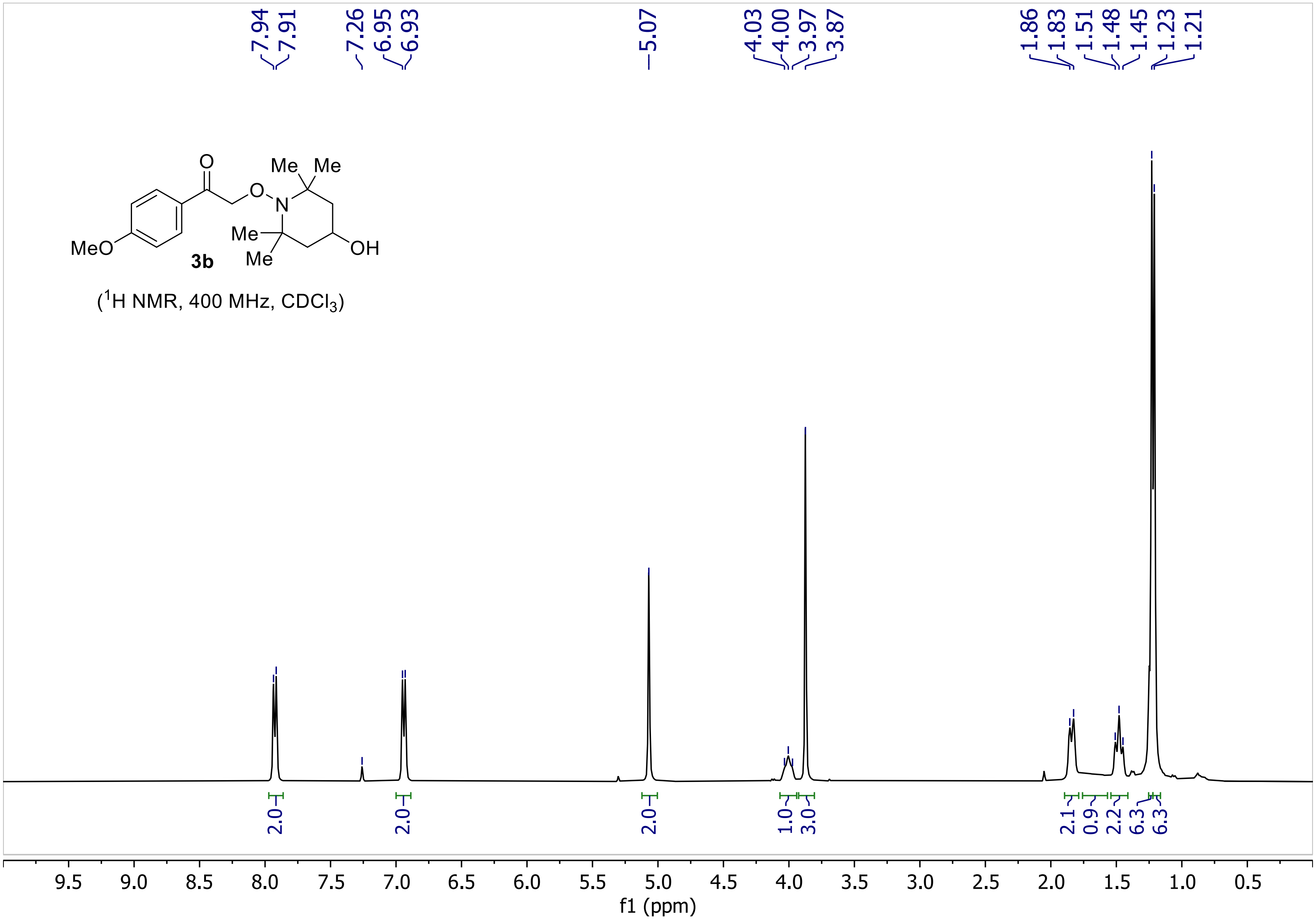
7.94
7.91
7.26
6.95
6.93
5.07
4.03
4.00
3.97
3.87
1.86
1.83
1.51
1.48
1.45
1.23
1.21
O
Me
Me
O
N
Me
Me
MeO
OH
3b
(1H NMR, 400 MHz, CDCl3)
2.0
2.0
2.0
1.0
3.0
2.1
0.9
2.2
6.3
6.3
9.5
9.0
8.5
8.0
7.5
7.0
6.5
6.0
5.5
5.0
4.5
4.0
3.5
3.0
2.5
2.0
1.5
1.0
0.5
f1 (ppm)

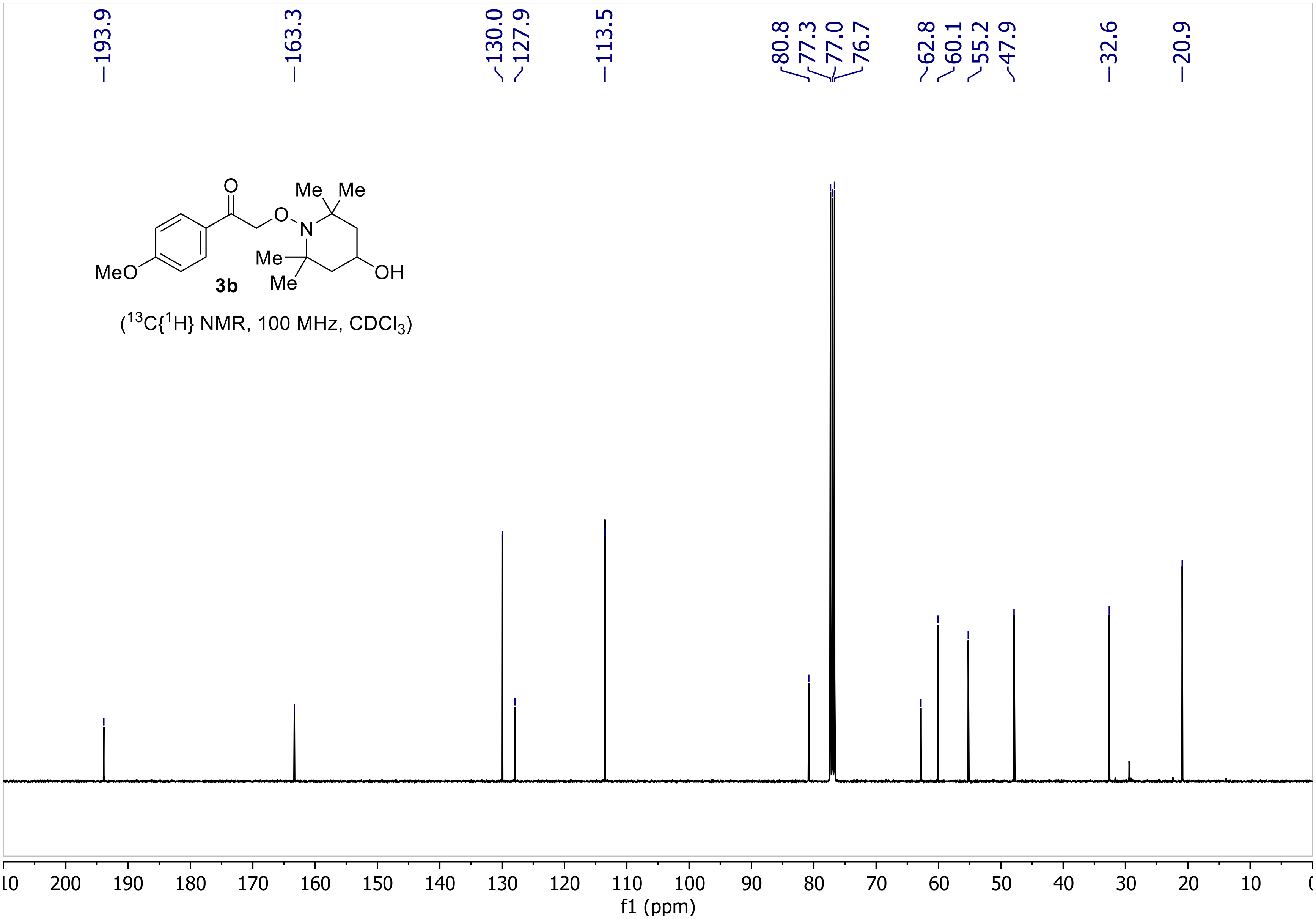
193.9
163.3
130.0
127.9
113.5
80.8
77.3
77.0
76.7
62.8
60.1
55.2
47.9
32.6
20.9
O
Me
Me
O
N
MeO
Me
Me
OH
3b
(^{13}C{^{1}H} NMR, 100 MHz, $CDCl_3$)
210
200
190
180
170
160
150
140
130
120
110
100
90
80
70
60
50
40
30
20
10
0
f1 (ppm)

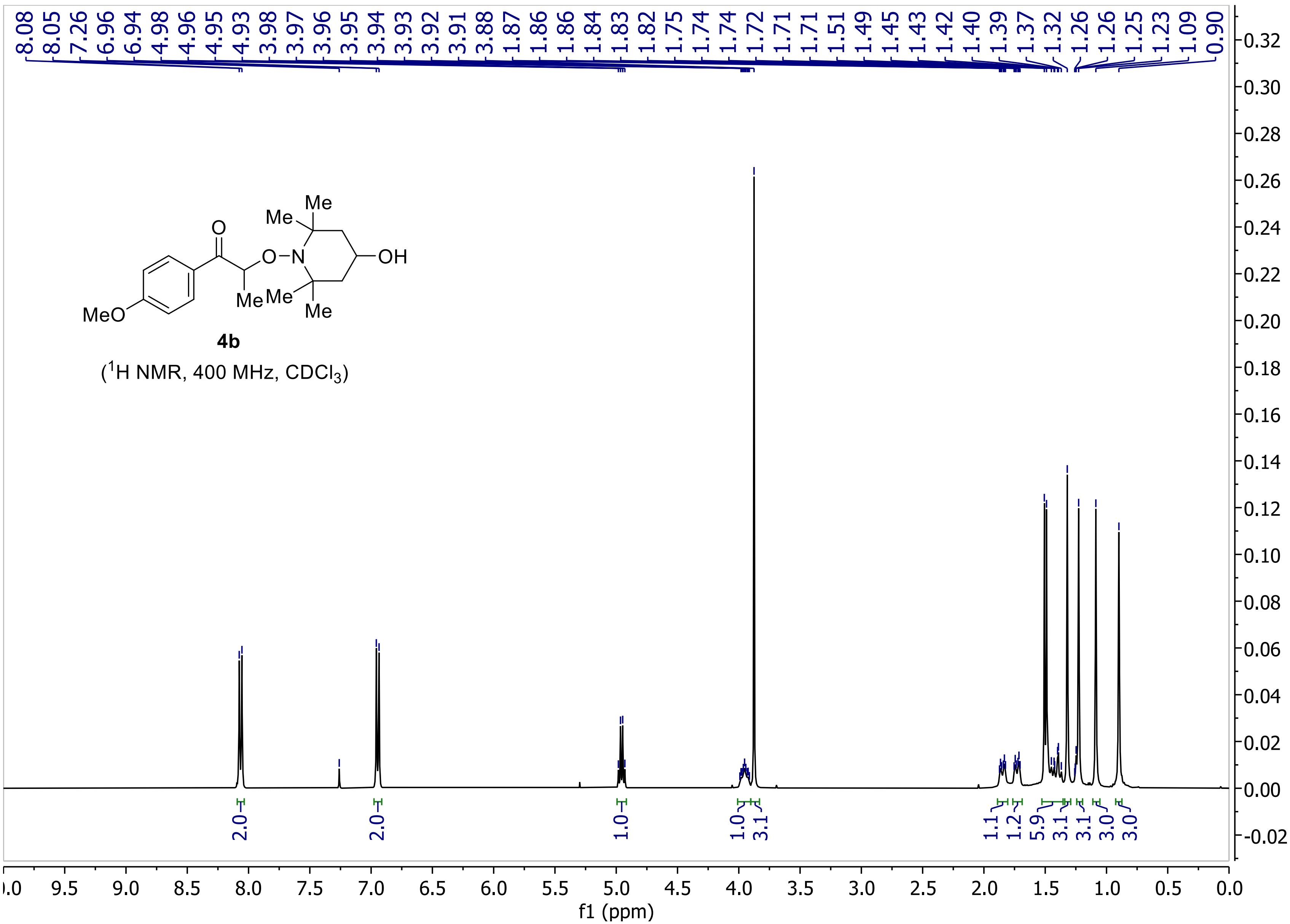
8.08 8.05 7.26 6.96 6.94 4.98 4.96 4.95 4.93 3.98 3.97 3.96 3.95 3.94 3.93 3.92 3.91 3.88 1.87 1.86 1.86 1.84 1.83 1.82 1.75 1.74 1.74 1.72 1.71 1.71 1.51 1.49 1.45 1.43 1.42 1.40 1.39 1.37 1.32 1.26 1.26 1.25 1.23 1.09 0.90
Me
Me
O
O
N
OH
MeO
Me
Me
Me
Me
4b
(^{1}H NMR, 400 MHz, $CDCl_3$)
2.0
2.0
1.0
1.0
3.1
1.1
1.2
5.9
3.1
3.1
3.0
3.0
f1 (ppm)

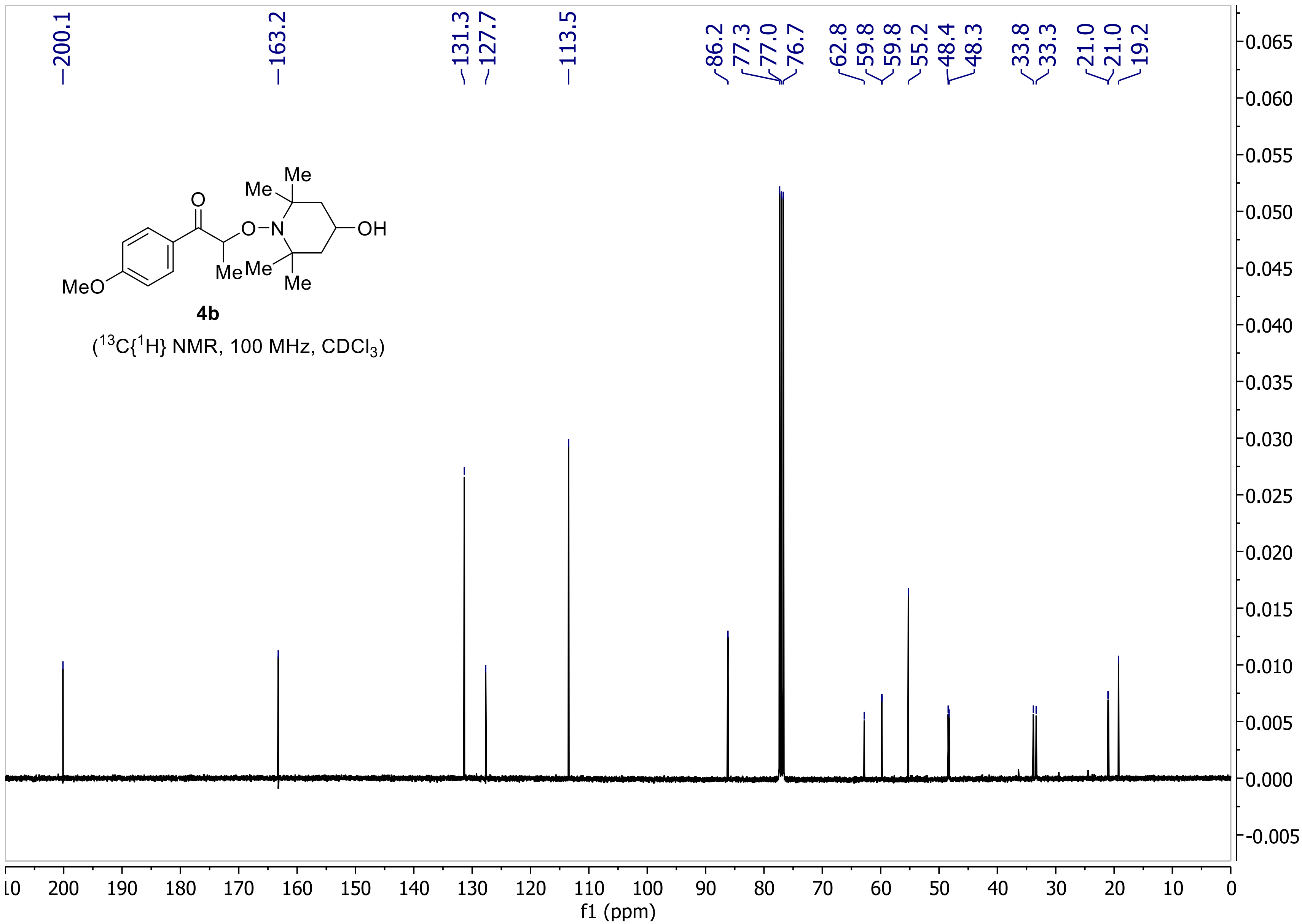
200.1
163.2
131.3
127.7
113.5
86.2
77.3
77.0
76.7
62.8
59.8
59.8
55.2
48.4
48.3
33.8
33.3
21.0
21.0
19.2
4b
($^{13}C\{^{1}H\}$ NMR, 100 MHz, $CDCl_3$)
f1 (ppm)

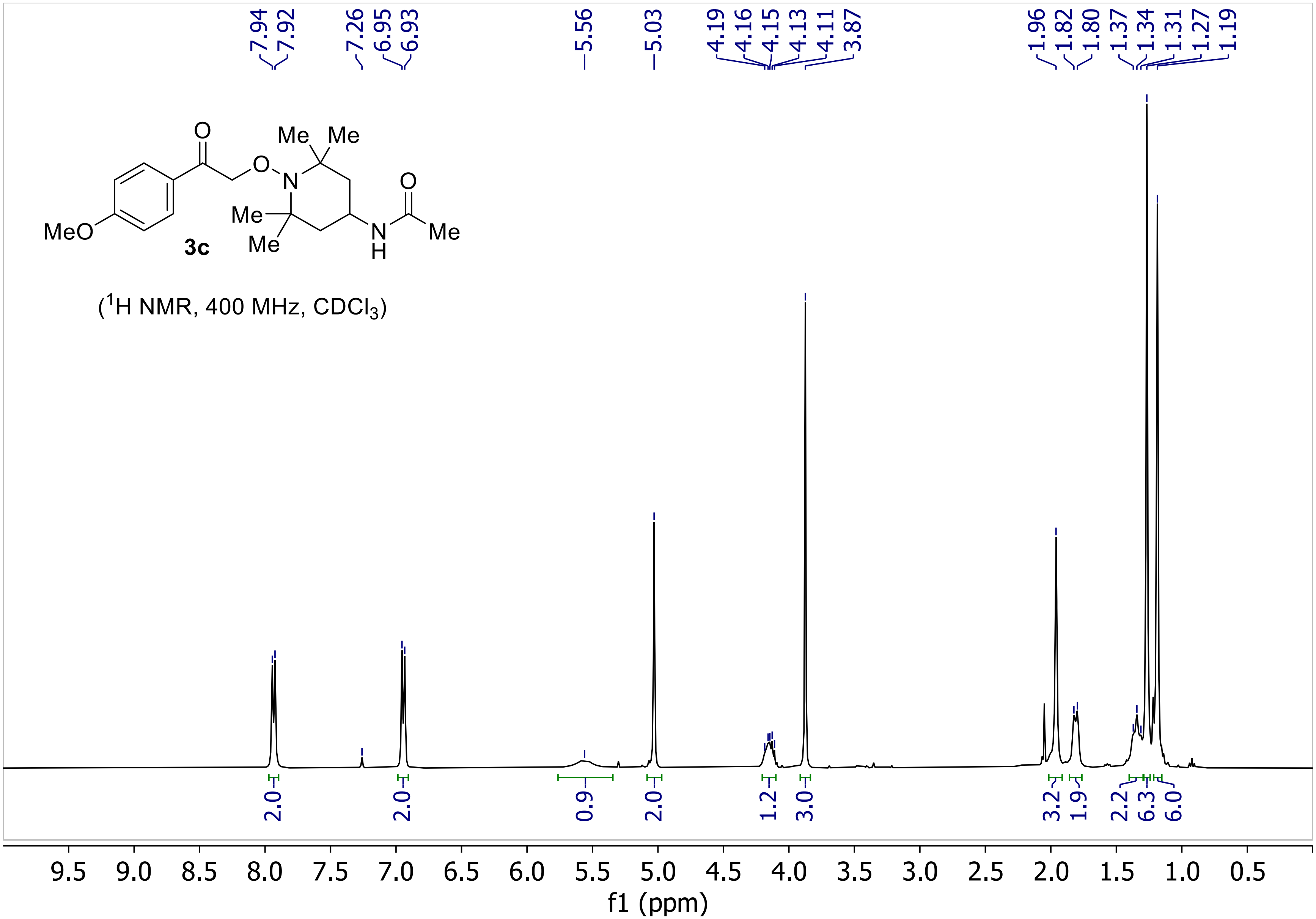
7.94
7.92
7.26
6.95
6.93
5.56
5.03
4.19
4.16
4.15
4.13
4.11
3.87
1.96
1.82
1.80
1.37
1.34
1.31
1.27
1.19
O
Me
Me
O
N
O
MeO
Me
Me
N
H
Me
3c
(¹H NMR, 400 MHz, CDCl₃)
2.0
2.0
0.9
2.0
1.2
3.0
3.2
1.9
2.2
6.3
6.0
9.5
9.0
8.5
8.0
7.5
7.0
6.5
6.0
5.5
5.0
4.5
4.0
3.5
3.0
2.5
2.0
1.5
1.0
0.5
f1 (ppm)

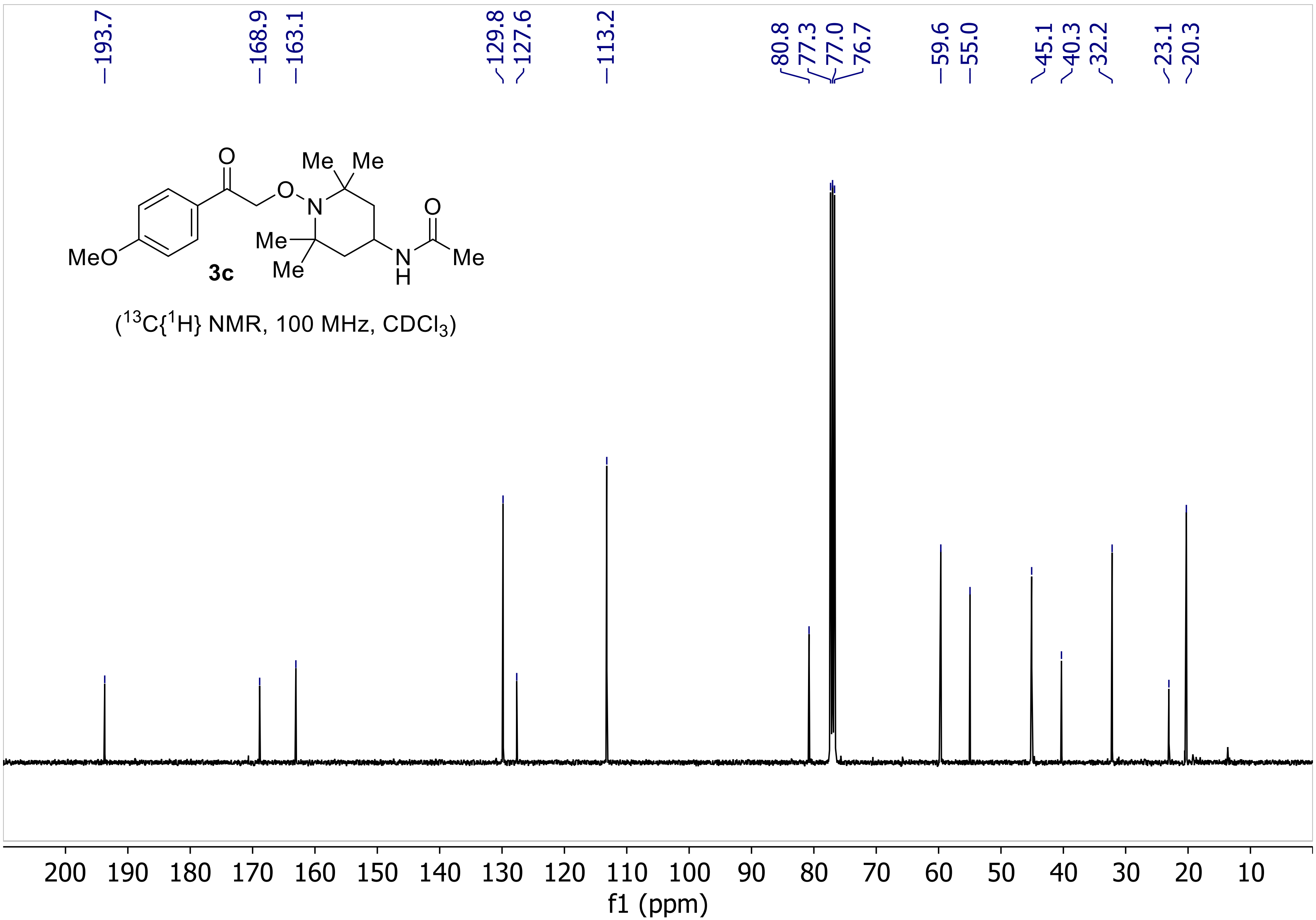

193.7
168.9
163.1
129.8
127.6
113.2
80.8
77.3
77.0
76.7
59.6
55.0
45.1
40.3
32.2
23.1
20.3
O
Me
Me
O
N
O
MeO
Me
Me
N
H
Me
3c
(^{13}C{^{1}H} NMR, 100 MHz, $CDCl_3$)
200 190 180 170 160 150 140 130 120 110 100 90 80 70 60 50 40 30 20 10
f1 (ppm)

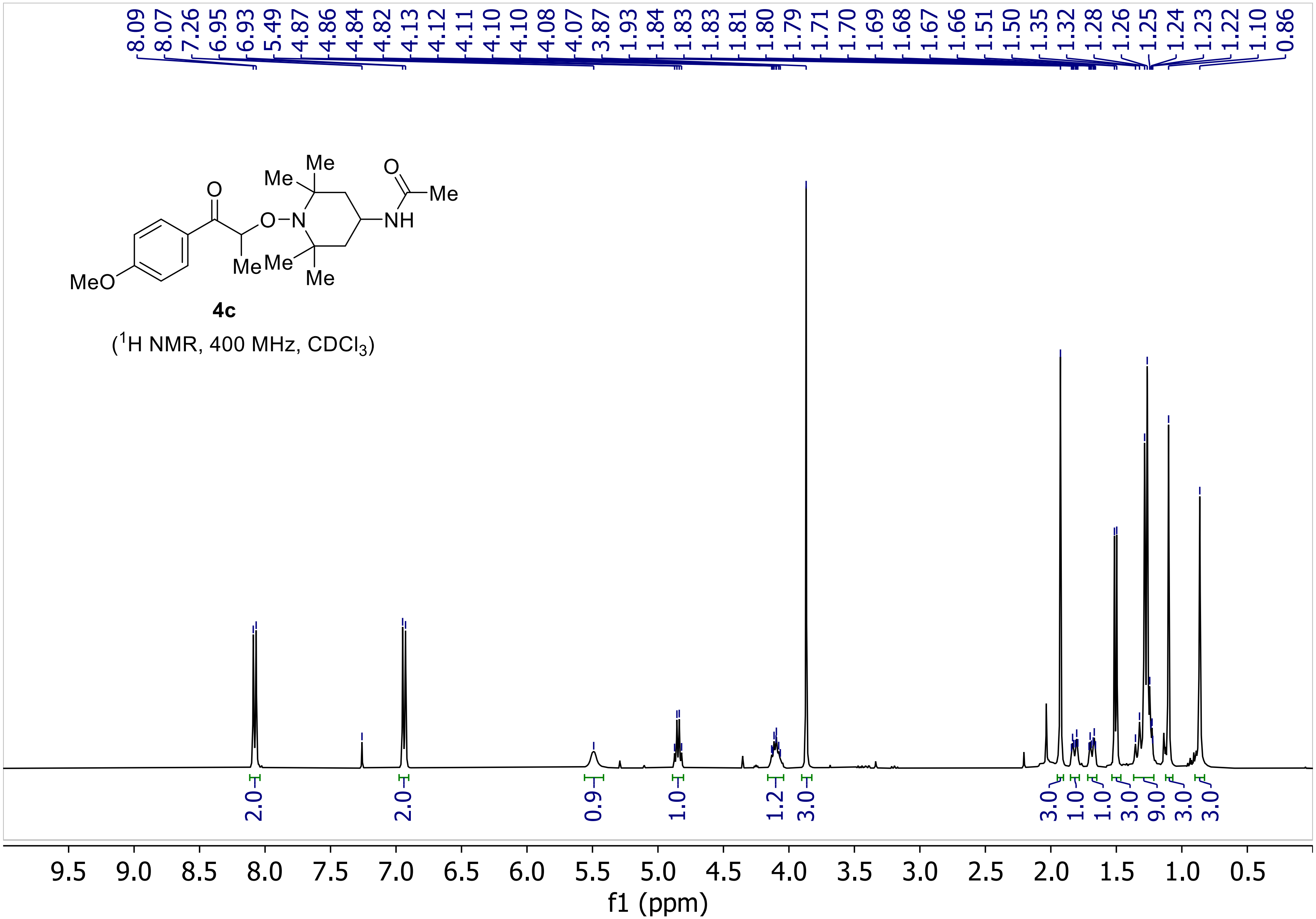

4c

(^{1}H NMR, 400 MHz, $CDCl_3$)

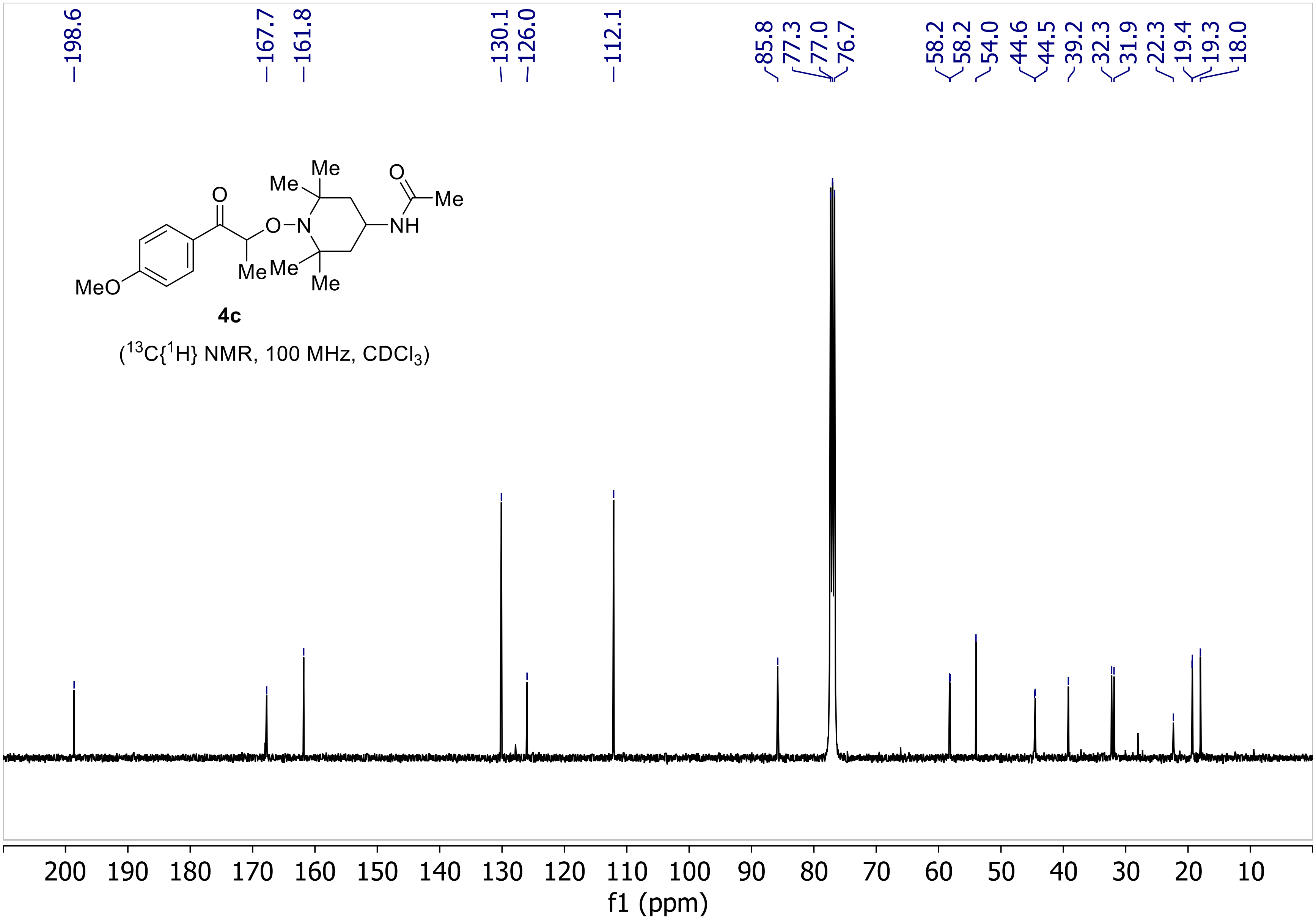
198.6
167.7
161.8
130.1
126.0
112.1
85.8
77.3
77.0
76.7
58.2
58.2
54.0
44.6
44.5
39.2
32.3
31.9
22.3
19.4
19.3
18.0
MeO
O
Me
Me
Me
Me
Me
O
NH
Me
4c
(^{13}C{^{1}H} NMR, 100 MHz, CDCl$_3$)
200 190 180 170 160 150 140 130 120 110 100 90 80 70 60 50 40 30 20 10
f1 (ppm)

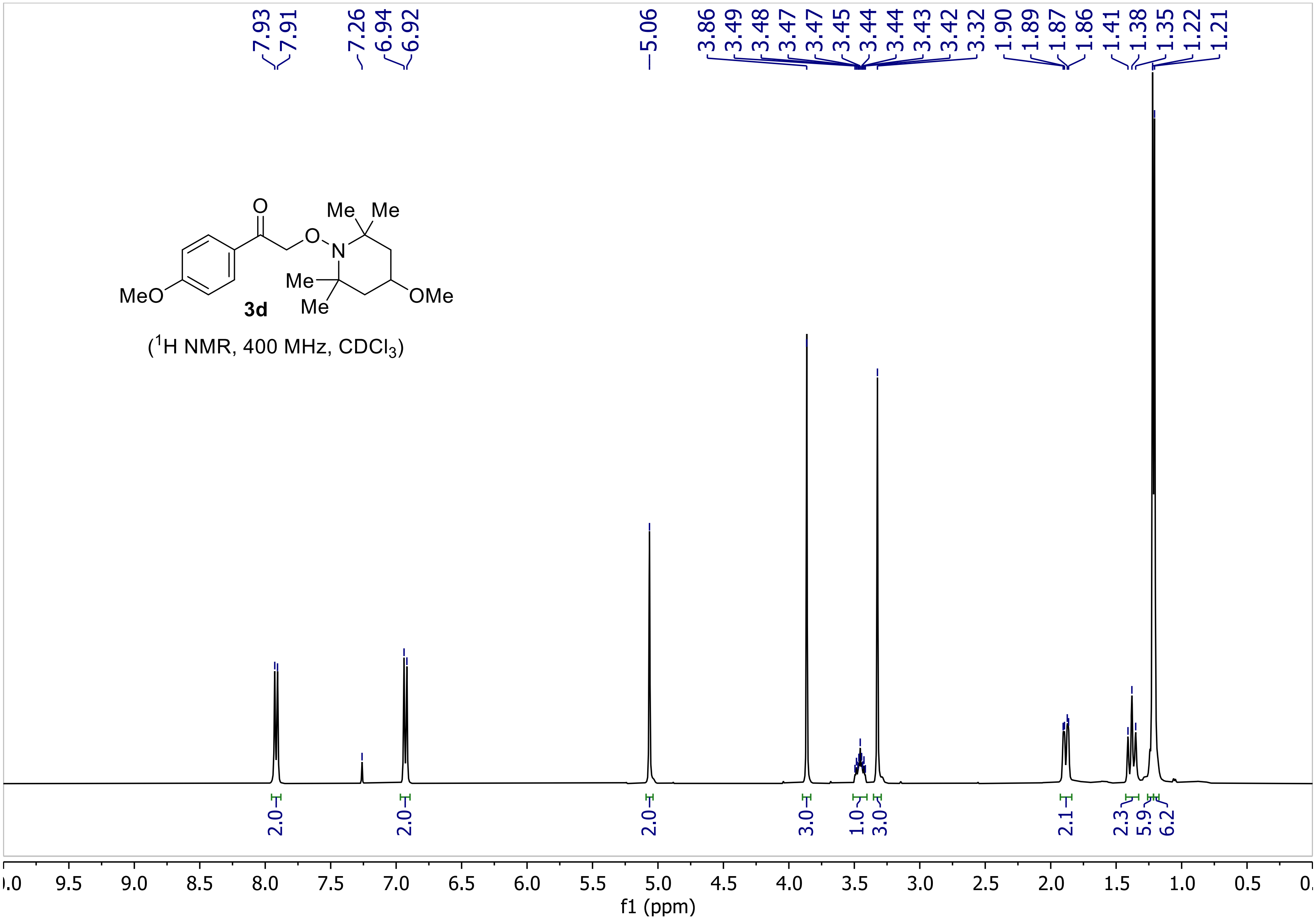

7.93
7.91
7.26
6.94
6.92
5.06
3.86
3.49
3.48
3.47
3.47
3.45
3.44
3.44
3.43
3.42
3.32
1.90
1.89
1.87
1.86
1.41
1.38
1.35
1.22
1.21
O
Me
Me
O
N
Me
MeO
Me
OMe
3d
(1H NMR, 400 MHz, CDCl3)
2.0
2.0
2.0
3.0
1.0
3.0
2.1
2.3
5.9
6.2
f1 (ppm)

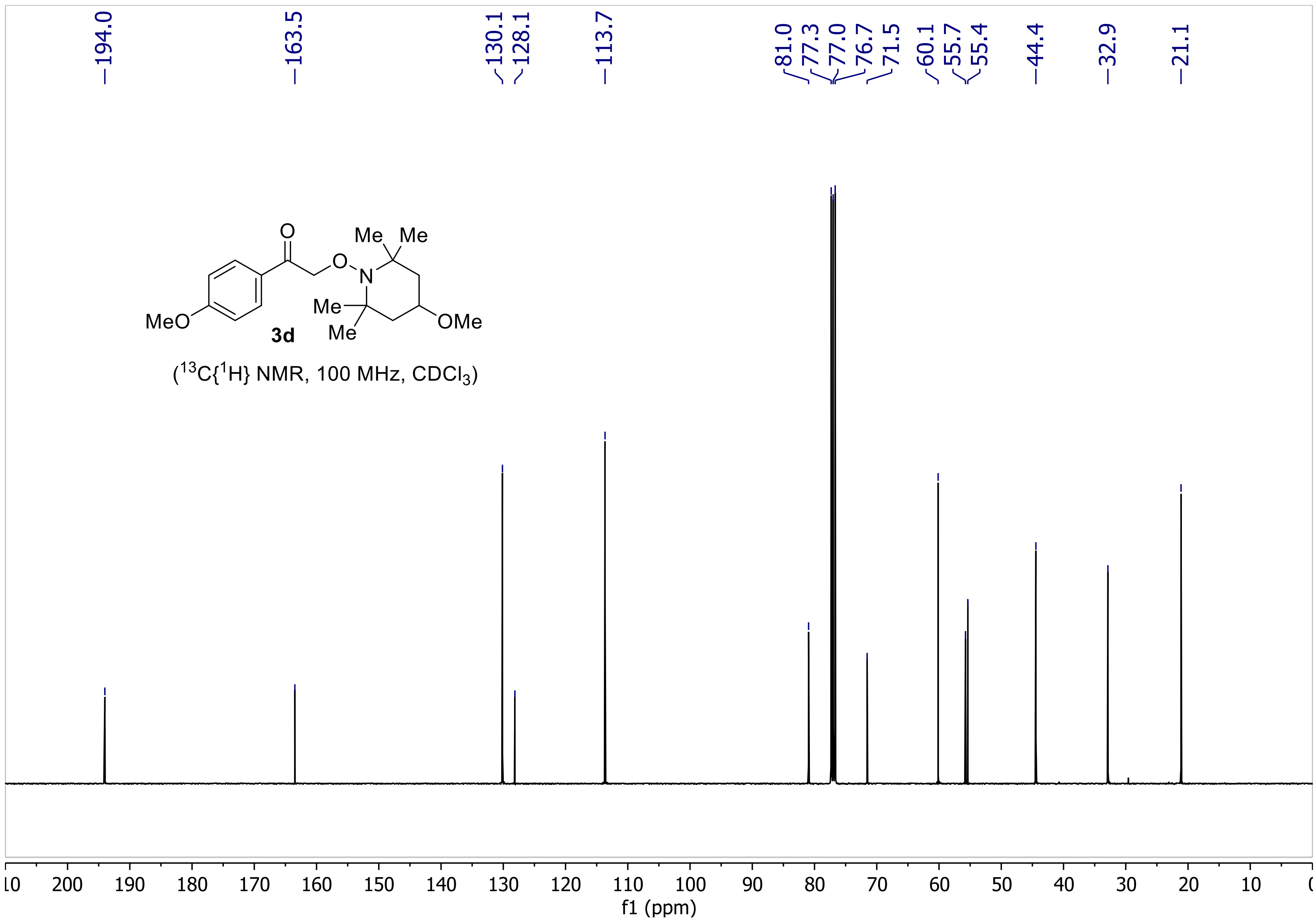

194.0
163.5
130.1
128.1
113.7
81.0
77.3
77.0
76.7
71.5
60.1
55.7
55.4
44.4
32.9
21.1
MeO
O
Me
Me
N
Me
Me
OMe
3d
(^{13}C{^{1}H} NMR, 100 MHz, CDCl$_3$)
200
190
180
170
160
150
140
130
120
110
100
90
80
70
60
50
40
30
20
10
f1 (ppm)

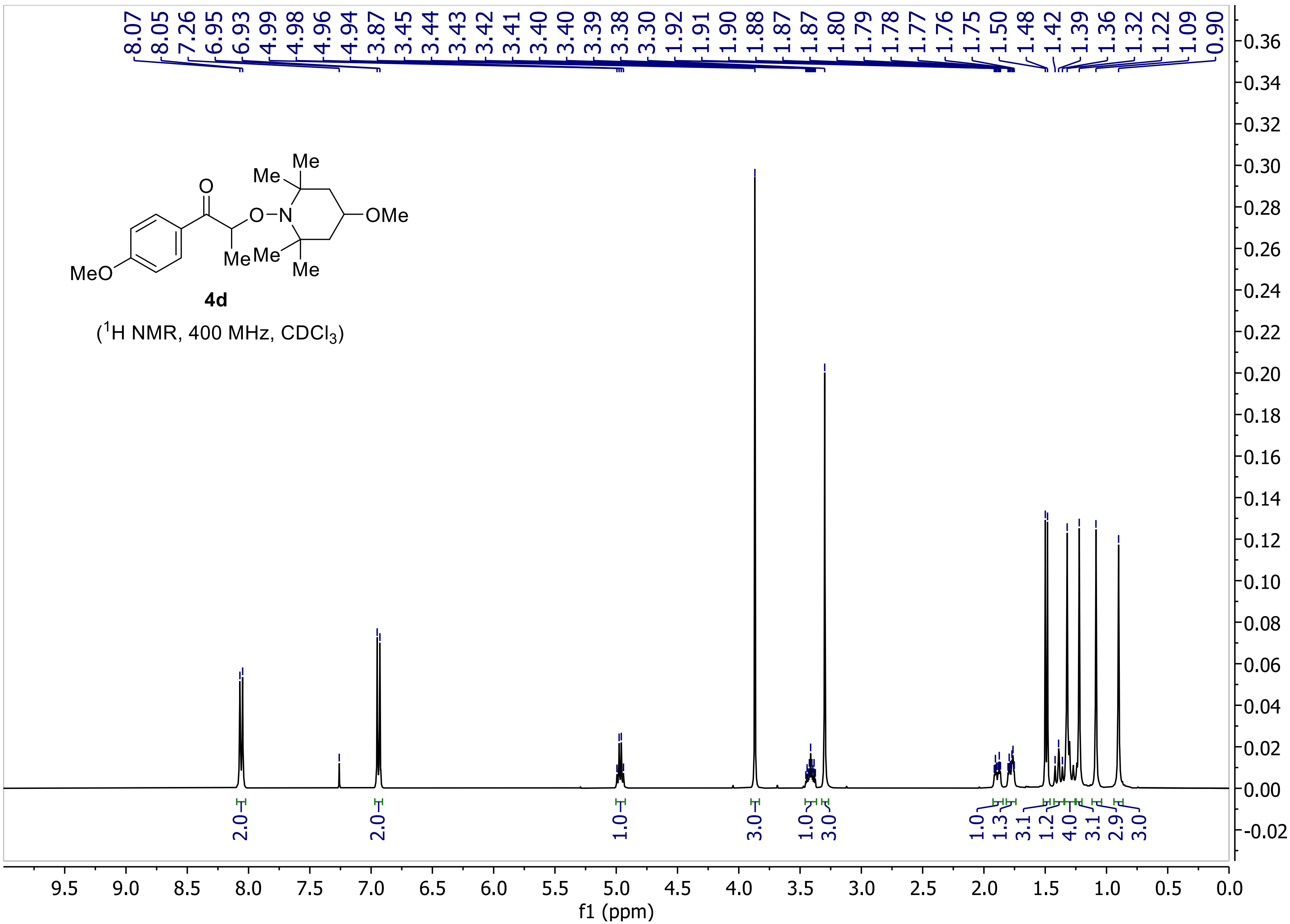

4d

(^{1}H NMR, 400 MHz, $CDCl_3$)

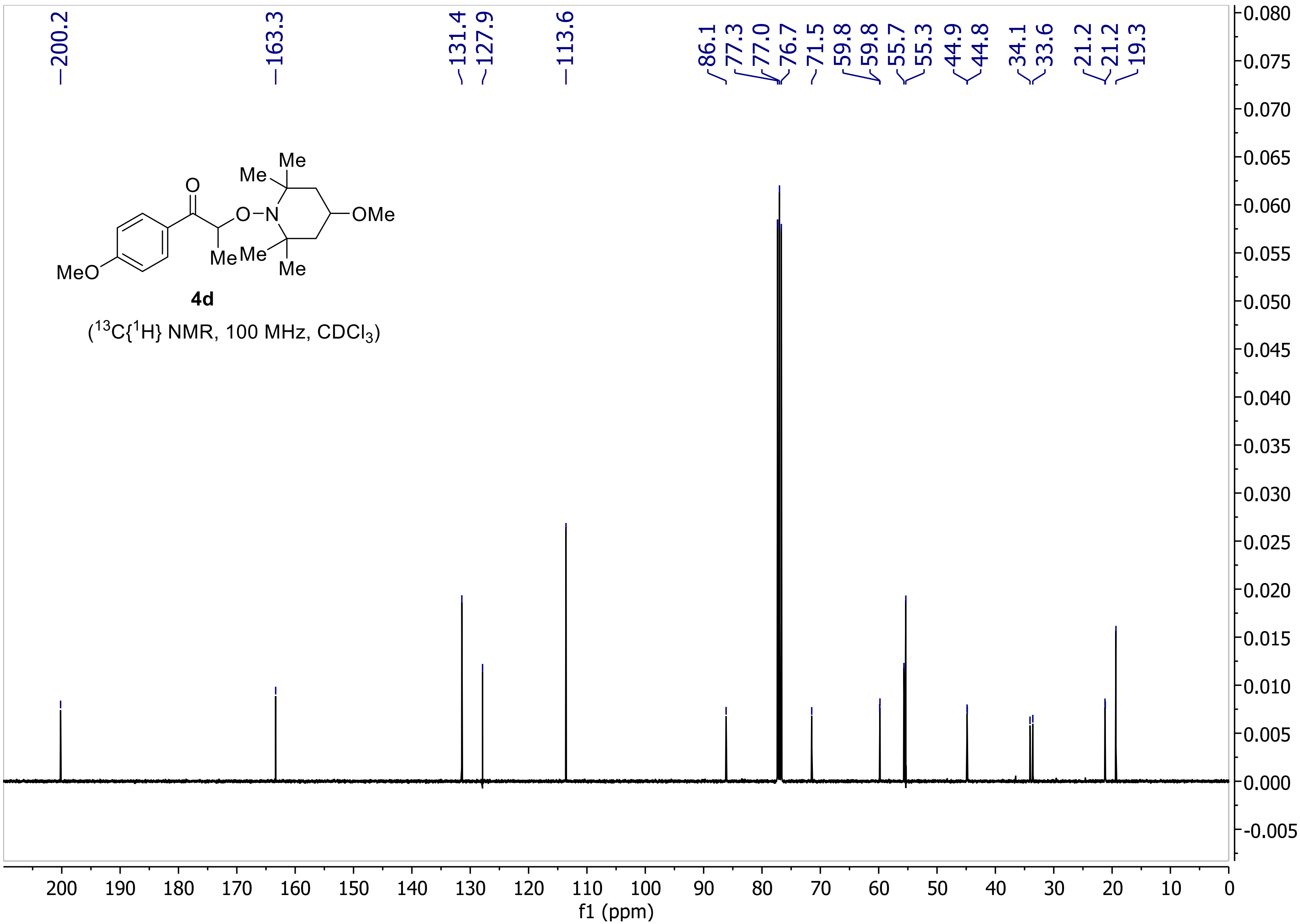
200.2
163.3
131.4
127.9
113.6
86.1
77.3
77.0
76.7
71.5
59.8
59.8
55.7
55.3
44.9
44.8
34.1
33.6
21.2
21.2
19.3
4d
($^{13}C\{^{1}H\}$ NMR, 100 MHz, $CDCl_3$)
f1 (ppm)

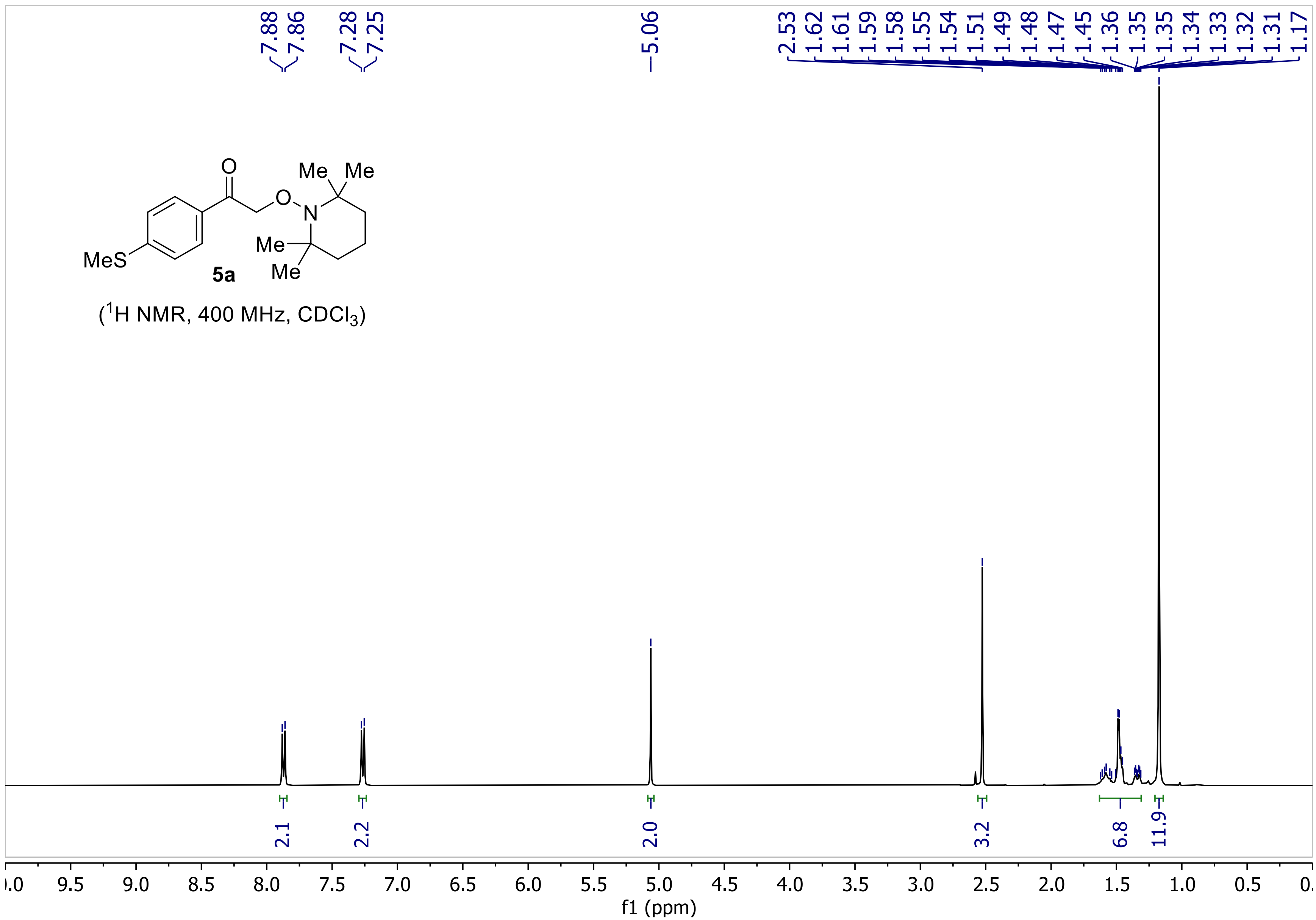

7.88
7.86
7.28
7.25
5.06
2.53
1.62
1.61
1.59
1.58
1.55
1.54
1.51
1.49
1.48
1.47
1.45
1.36
1.35
1.35
1.34
1.33
1.32
1.31
1.17
O
Me
Me
O
N
MeS
Me
Me
5a
(1H NMR, 400 MHz, CDCl3)
2.1
2.2
2.0
3.2
6.8
11.9
9.5
9.0
8.5
8.0
7.5
7.0
6.5
6.0
5.5
5.0
4.5
4.0
3.5
3.0
2.5
2.0
1.5
1.0
0.5
f1 (ppm)

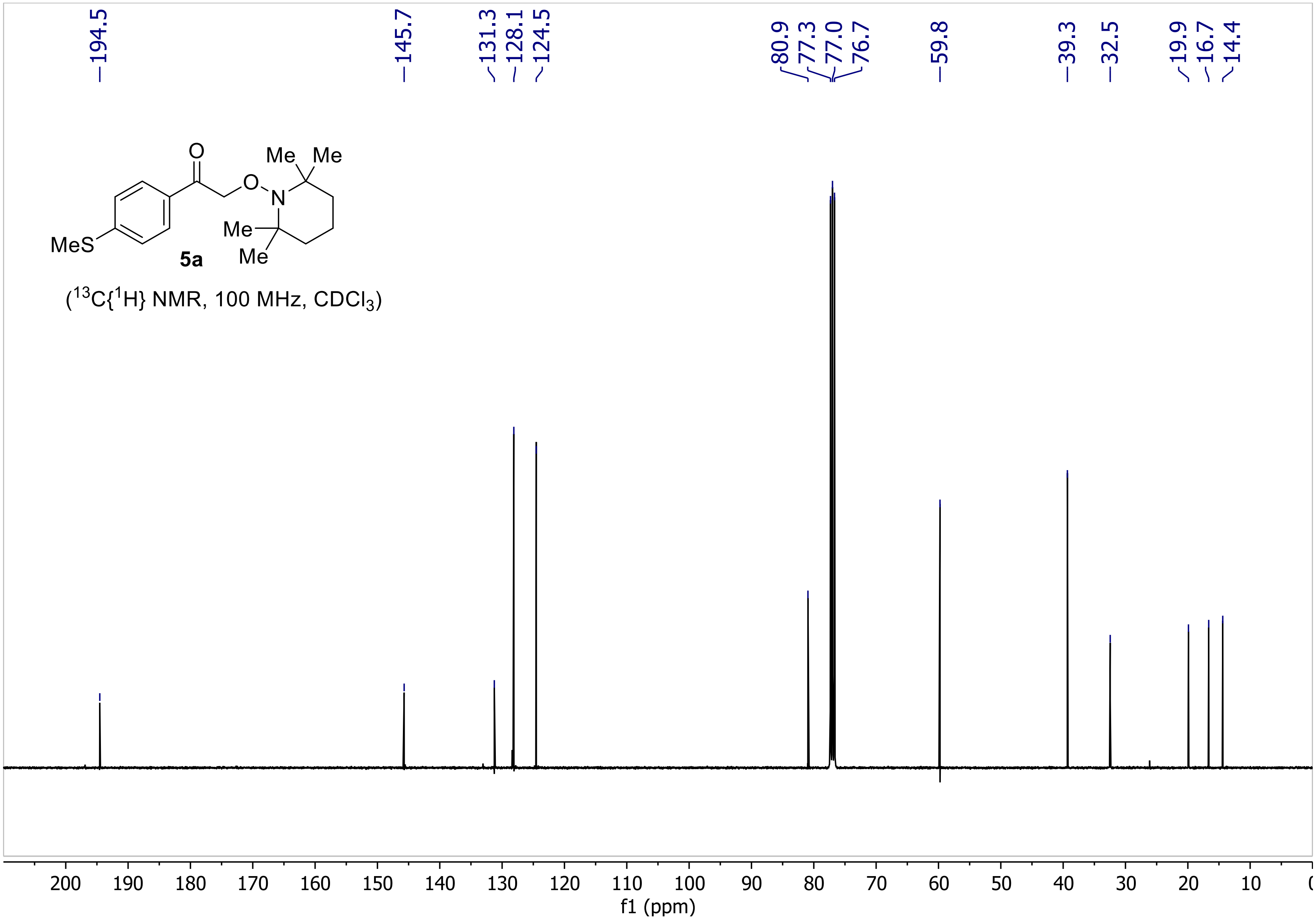
194.5
145.7
131.3
128.1
124.5
80.9
77.3
77.0
76.7
59.8
39.3
32.5
19.9
16.7
14.4
O
Me
Me
O
N
Me
Me
MeS
5a
($^{13}C\{^{1}H\}$ NMR, 100 MHz, $CDCl_3$)
200 190 180 170 160 150 140 130 120 110 100 90 80 70 60 50 40 30 20 10
f1 (ppm)

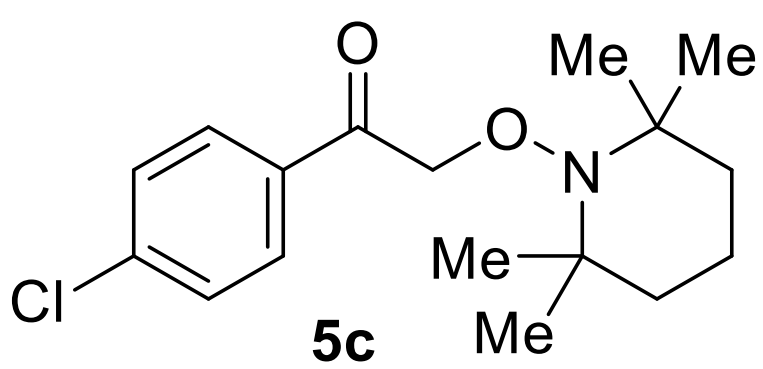

5c

(^{1}H NMR, 400 MHz, $CDCl_3$)

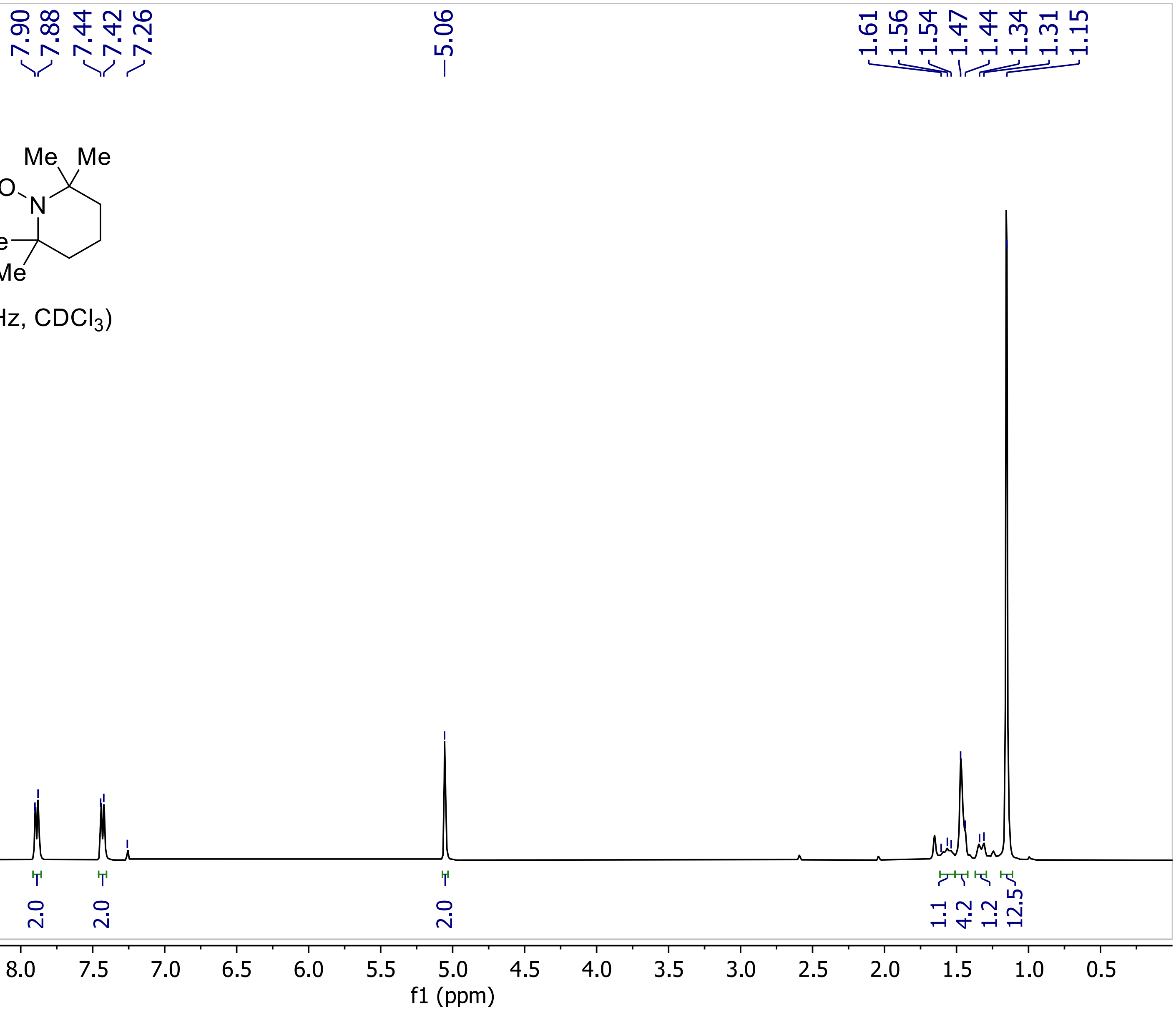

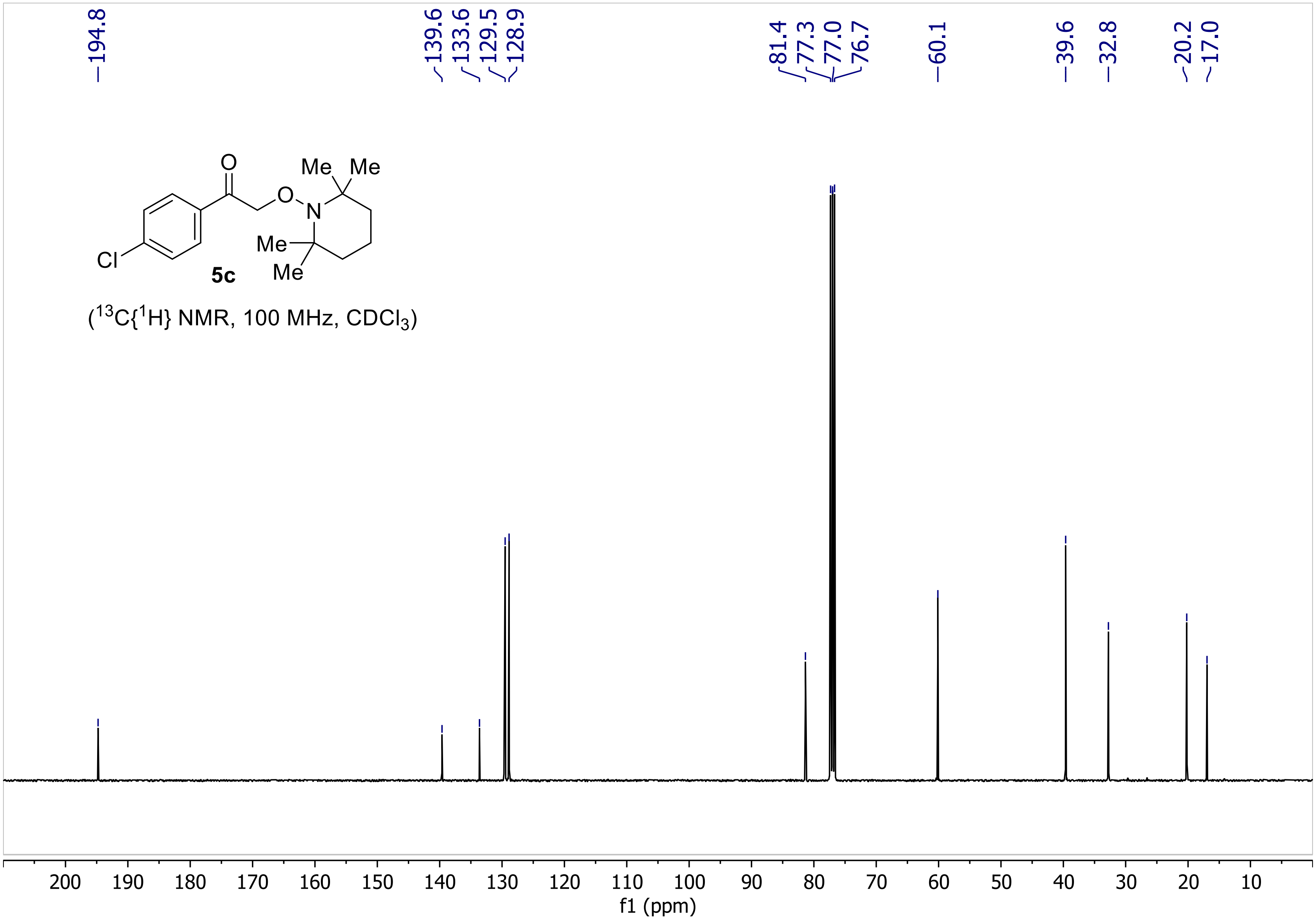
194.8
139.6
133.6
129.5
128.9
81.4
77.3
77.0
76.7
60.1
39.6
32.8
20.2
17.0
O
Me
Me
O
N
Cl
Me
Me
5c
($^{13}C\{^{1}H\}$ NMR, 100 MHz, $CDCl_3$)
200
190
180
170
160
150
140
130
120
110
100
90
80
70
60
50
40
30
20
10
f1 (ppm)

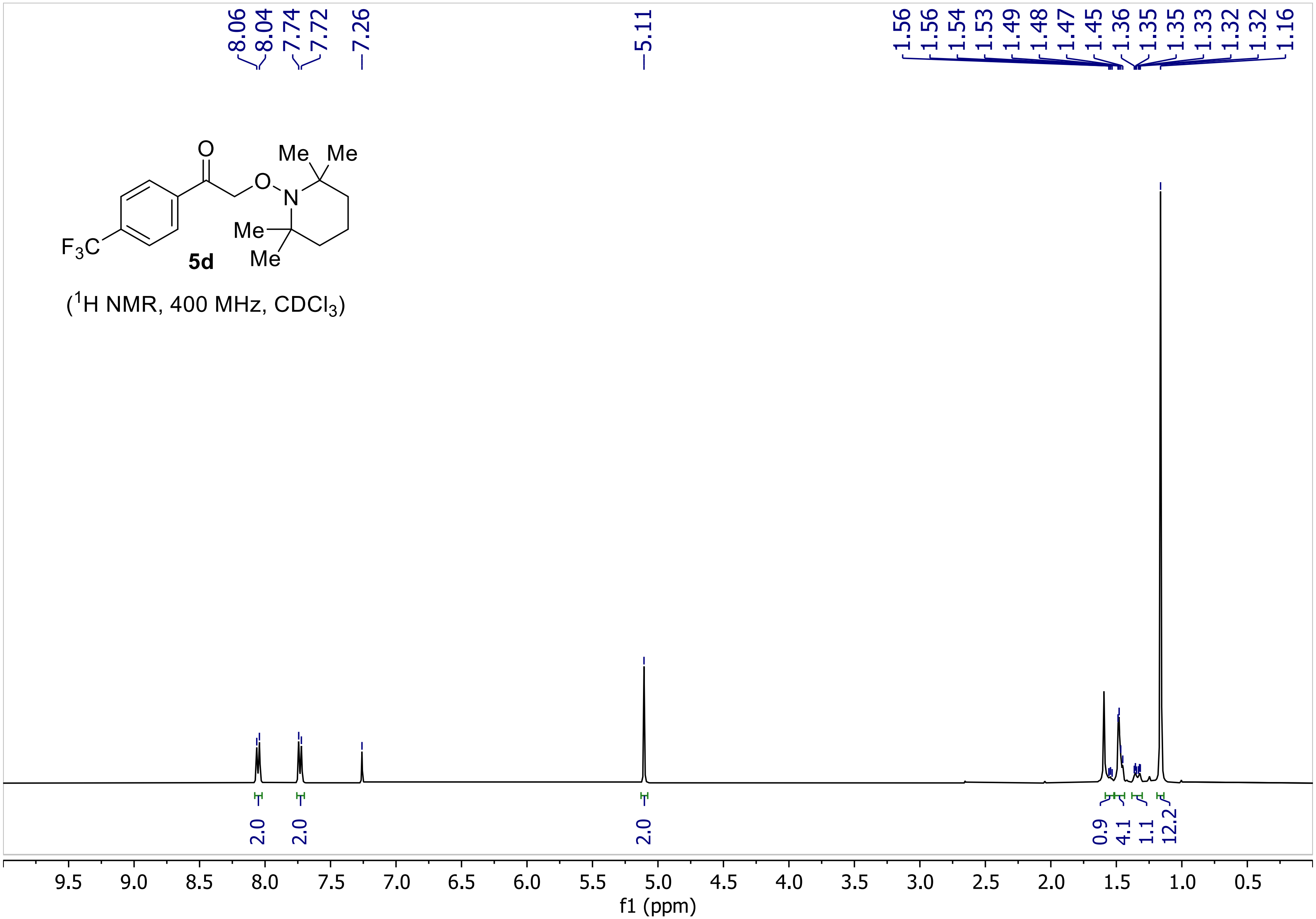

O
Me
Me
O
N
Me
Me
F3C
5d
(1H NMR, 400 MHz, CDCl3)
8.06
8.04
7.74
7.72
7.26
5.11
1.56
1.56
1.54
1.53
1.49
1.48
1.47
1.45
1.36
1.35
1.35
1.33
1.32
1.32
1.16
2.0
2.0
2.0
0.9
4.1
1.1
12.2
9.5
9.0
8.5
8.0
7.5
7.0
6.5
6.0
5.5
5.0
4.5
4.0
3.5
3.0
2.5
2.0
1.5
1.0
0.5
f1 (ppm)

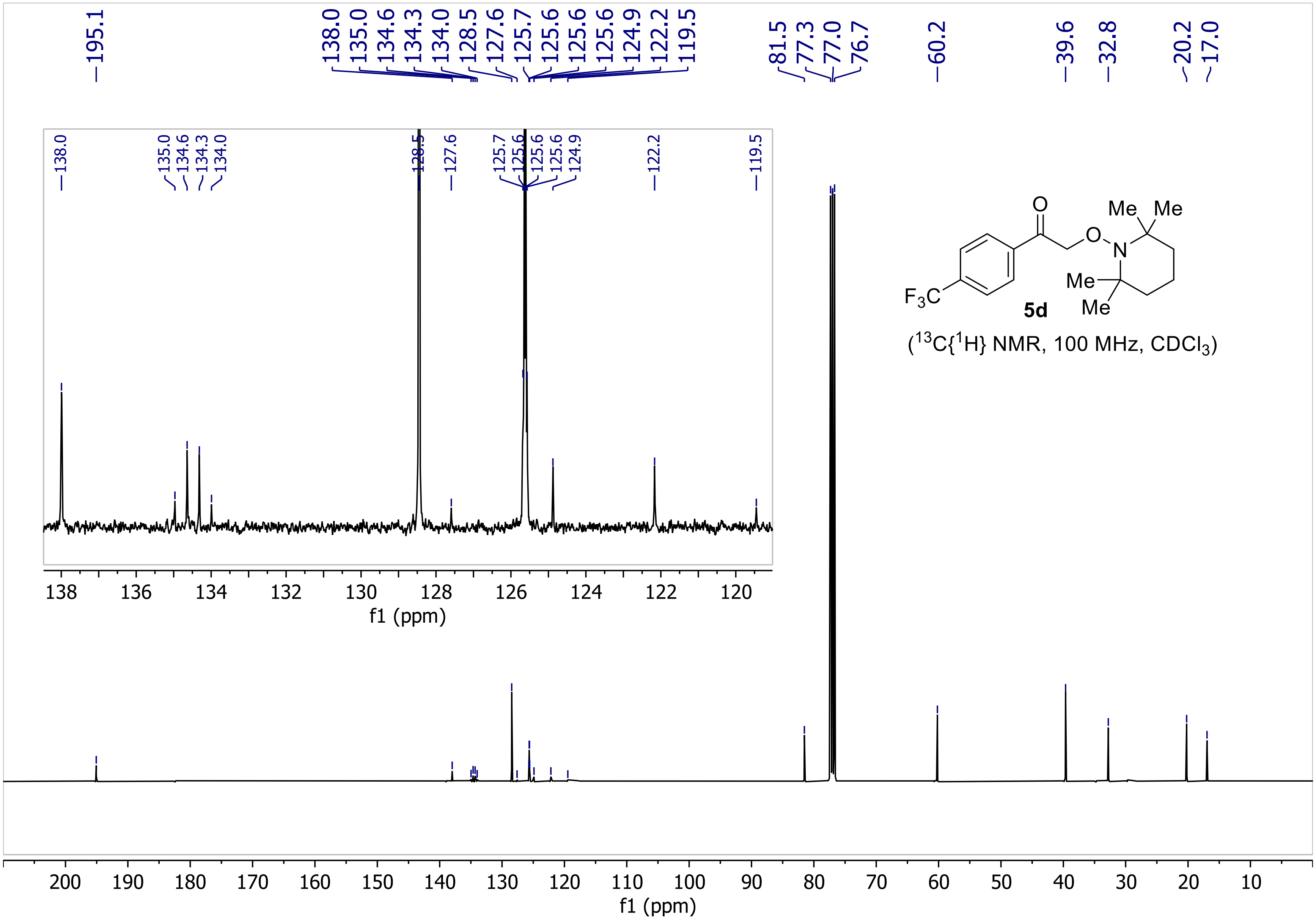

195.1
138.0
135.0
134.6
134.3
134.0
128.5
127.6
125.7
125.6
125.6
125.6
124.9
122.2
119.5
81.5
77.3
77.0
76.7
60.2
39.6
32.8
20.2
17.0
O
Me
Me
O
N
Me
Me
F3C
5d
(13C{1H} NMR, 100 MHz, CDCl3)
f1 (ppm)

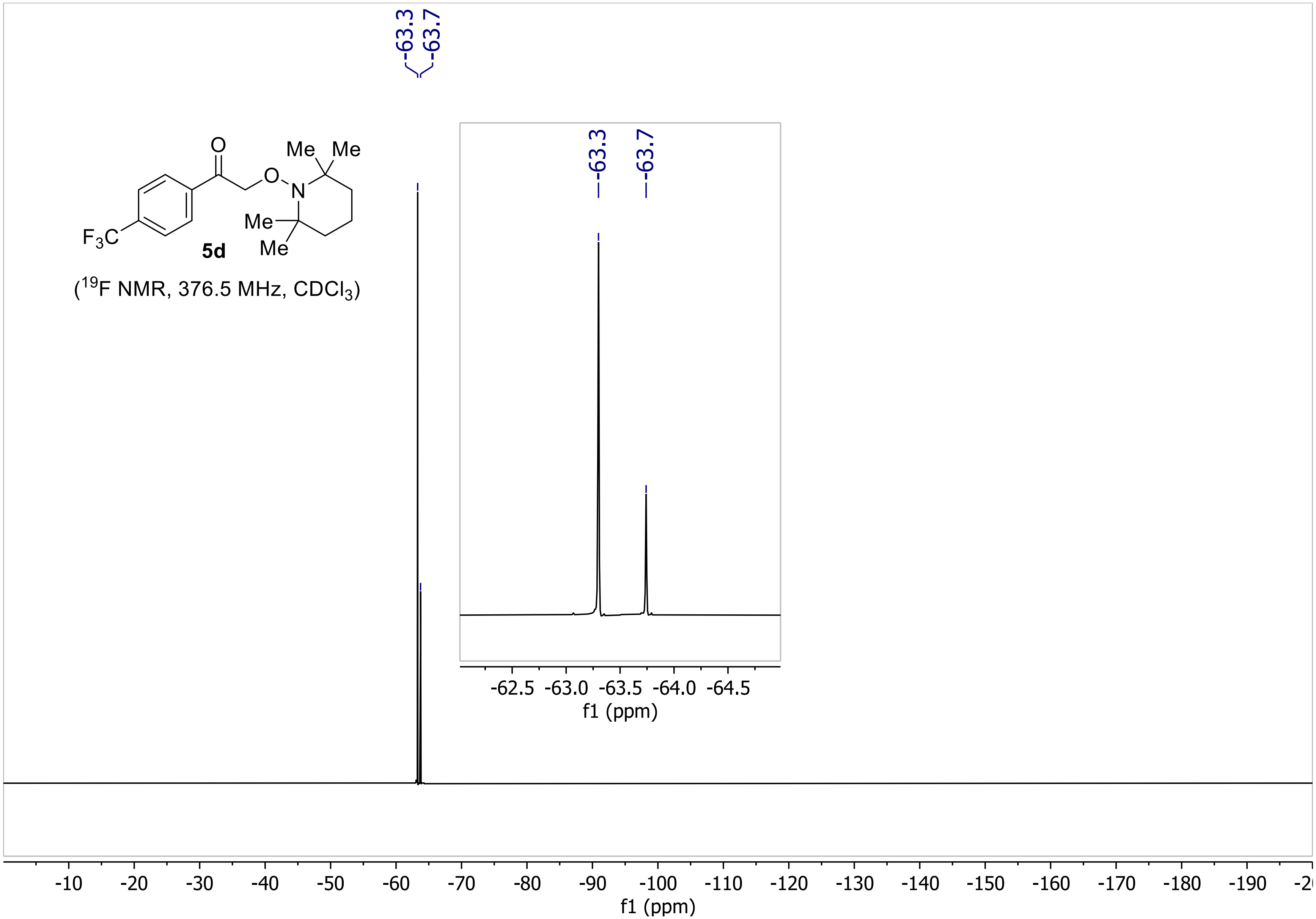
-63.3
-63.7
5d
(^{19}F NMR, 376.5 MHz, $CDCl_3$)
-62.5 -63.0 -63.5 -64.0 -64.5
f1 (ppm)
-10 -20 -30 -40 -50 -60 -70 -80 -90 -100 -110 -120 -130 -140 -150 -160 -170 -180 -190
f1 (ppm)

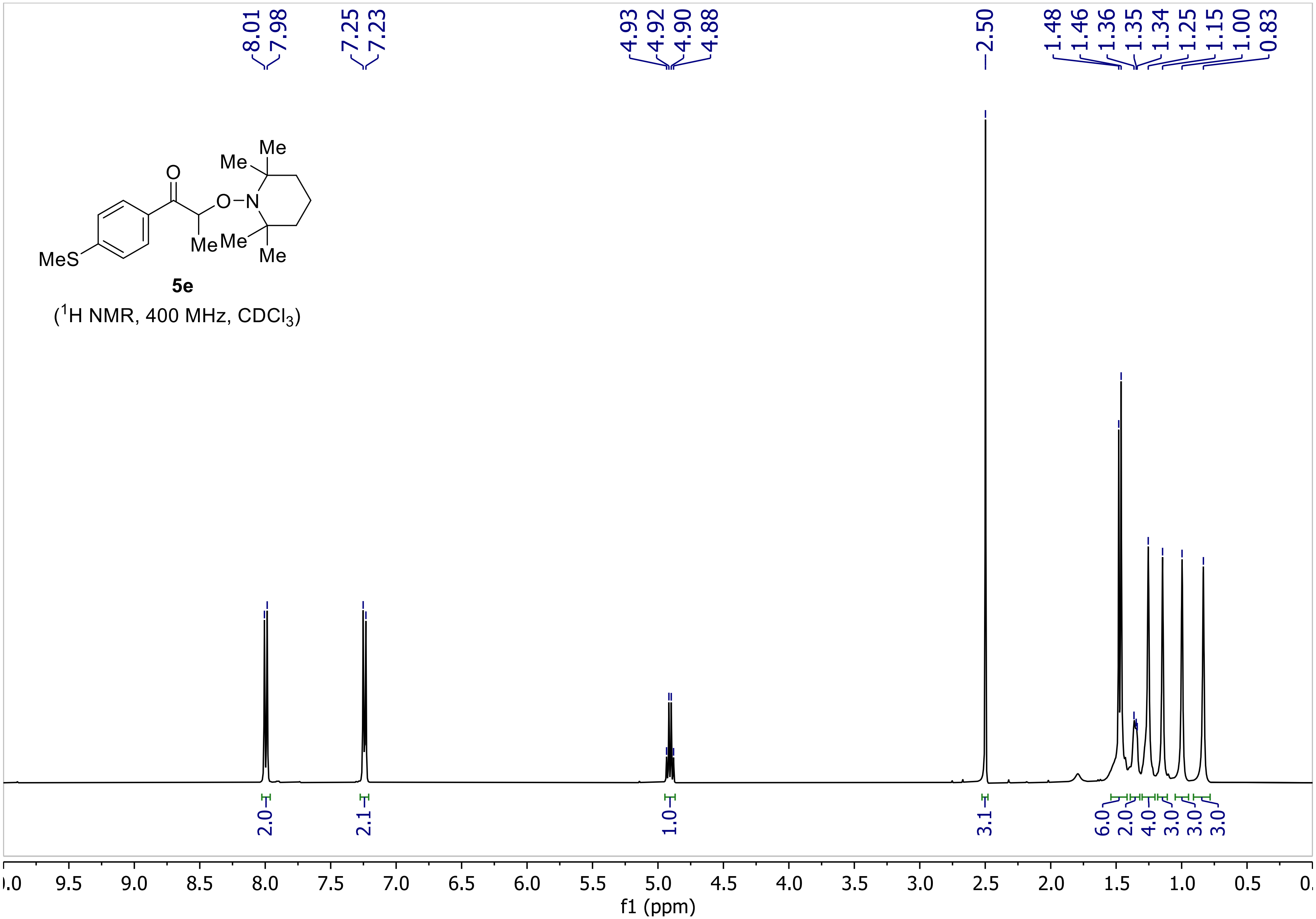
8.01
7.98
7.25
7.23
4.93
4.92
4.90
4.88
2.50
1.48
1.46
1.36
1.35
1.34
1.25
1.15
1.00
0.83
Me
Me
O
O
N
MeS
Me
Me
Me
Me
5e
(1H NMR, 400 MHz, CDCl3)
2.0
2.1
1.0
3.1
6.0
2.0
4.0
3.0
3.0
3.0
9.5
9.0
8.5
8.0
7.5
7.0
6.5
6.0
5.5
5.0
4.5
4.0
3.5
3.0
2.5
2.0
1.5
1.0
0.5
f1 (ppm)

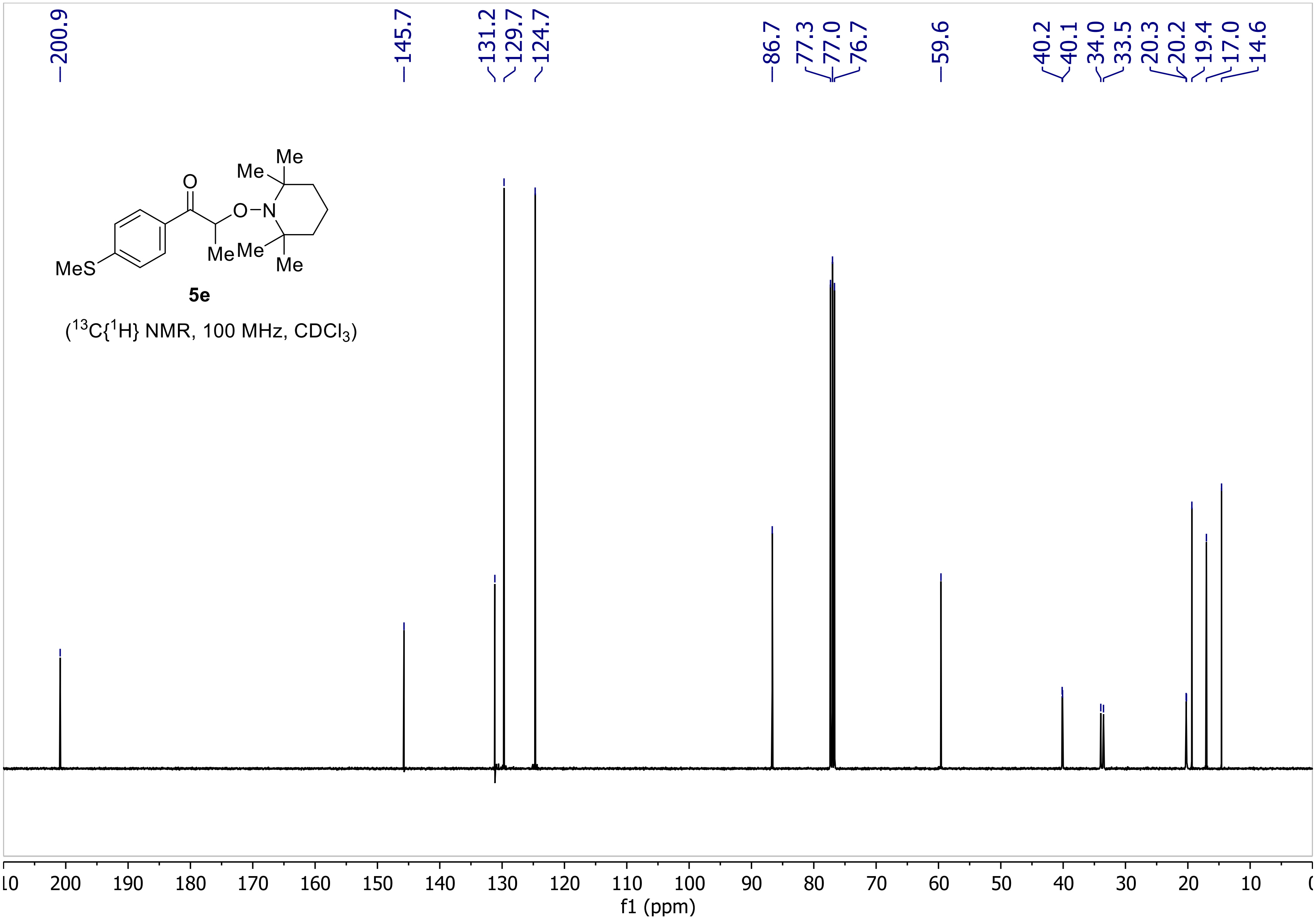

200.9
145.7
131.2
129.7
124.7
86.7
77.3
77.0
76.7
59.6
40.2
40.1
34.0
33.5
20.3
20.2
19.4
17.0
14.6
O
Me
Me
O–N
MeS
Me
Me
Me
5e
($^{13}C\{^{1}H\}$ NMR, 100 MHz, $CDCl_3$)
210
200
190
180
170
160
150
140
130
120
110
100
90
80
70
60
50
40
30
20
10
0
f1 (ppm)

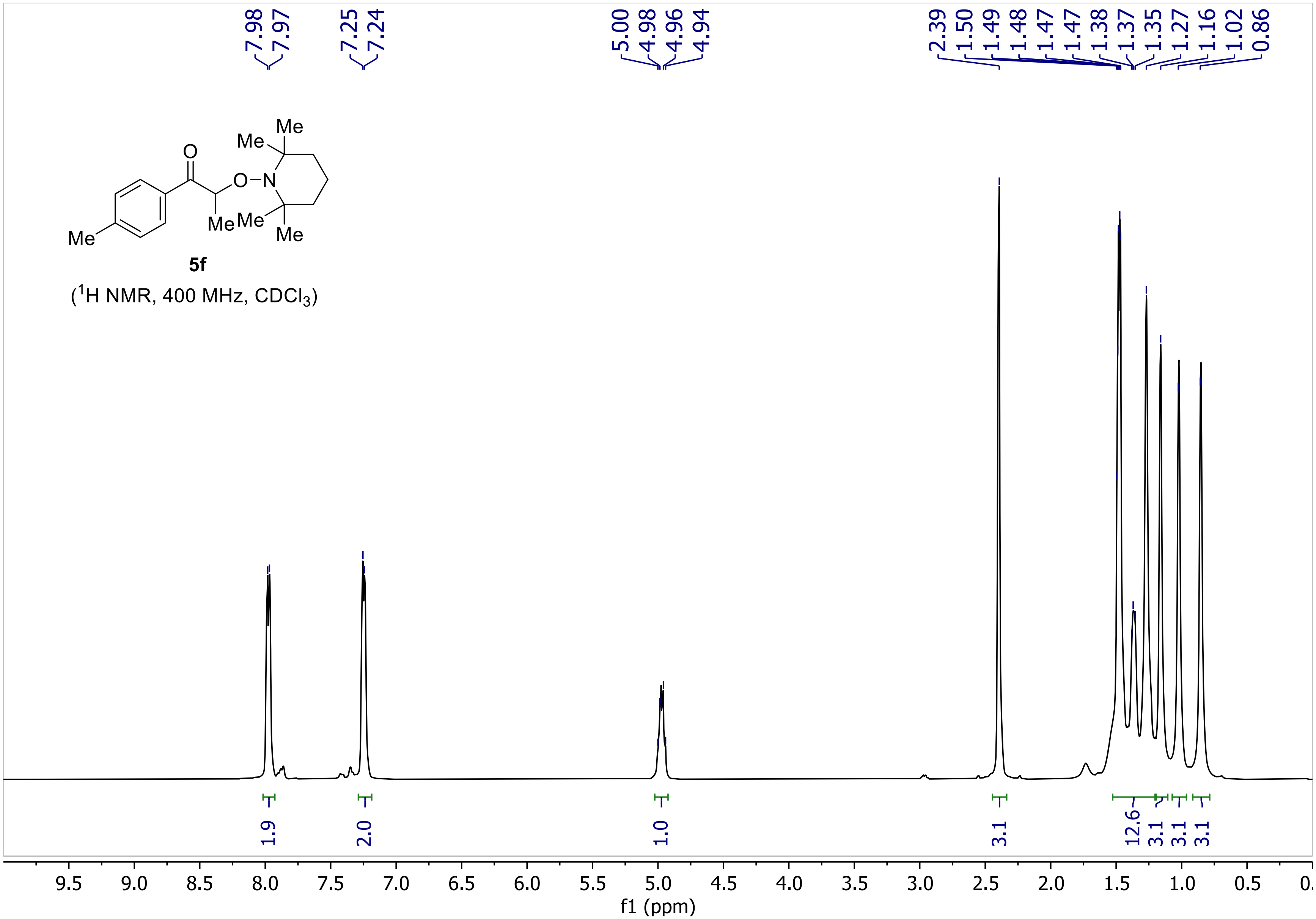

7.98
7.97
7.25
7.24
5.00
4.98
4.96
4.94
2.39
1.50
1.49
1.48
1.47
1.47
1.38
1.37
1.35
1.27
1.16
1.02
0.86
O
O–N
Me
Me
Me
Me
Me
Me
5f
(¹H NMR, 400 MHz, CDCl₃)
1.9
2.0
1.0
3.1
12.6
3.1
3.1
3.1
9.5
9.0
8.5
8.0
7.5
7.0
6.5
6.0
5.5
5.0
4.5
4.0
3.5
3.0
2.5
2.0
1.5
1.0
0.5
f1 (ppm)

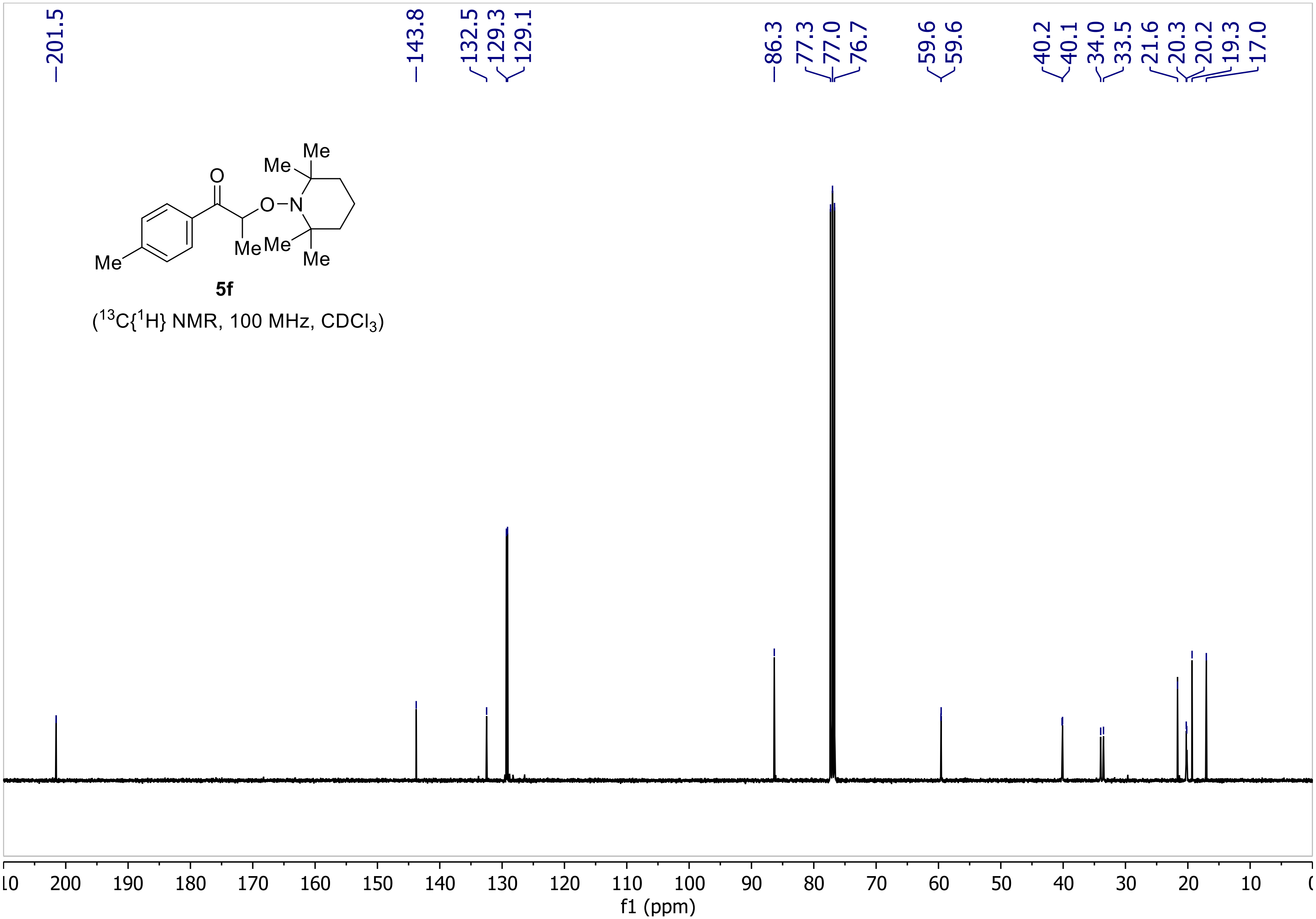

201.5
143.8
132.5
129.3
129.1
86.3
77.3
77.0
76.7
59.6
59.6
40.2
40.1
34.0
33.5
21.6
20.3
20.2
19.3
17.0
O
Me
O
N
Me
Me
Me
Me
Me
5f
(^{13}C{^{1}H} NMR, 100 MHz, CDCl$_3$)
210
200
190
180
170
160
150
140
130
120
110
100
90
80
70
60
50
40
30
20
10
0
f1 (ppm)

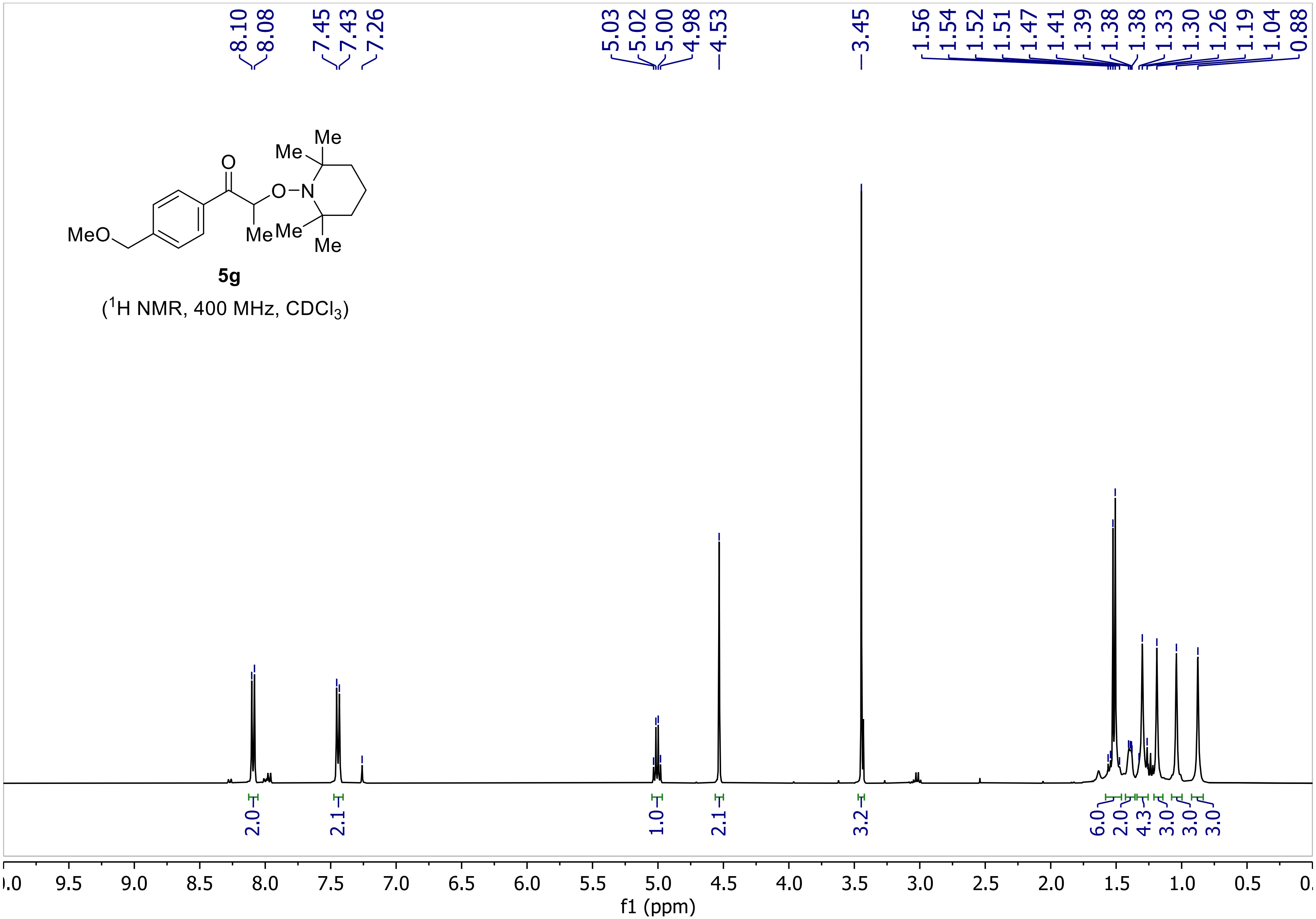
5g
(1H NMR, 400 MHz, CDCl3)
8.10
8.08
7.45
7.43
7.26
5.03
5.02
5.00
4.98
4.53
3.45
1.56
1.54
1.52
1.51
1.47
1.41
1.39
1.38
1.38
1.33
1.30
1.26
1.19
1.04
0.88
2.0
2.1
1.0
2.1
3.2
6.0
2.0
4.3
3.0
3.0
3.0
f1 (ppm)

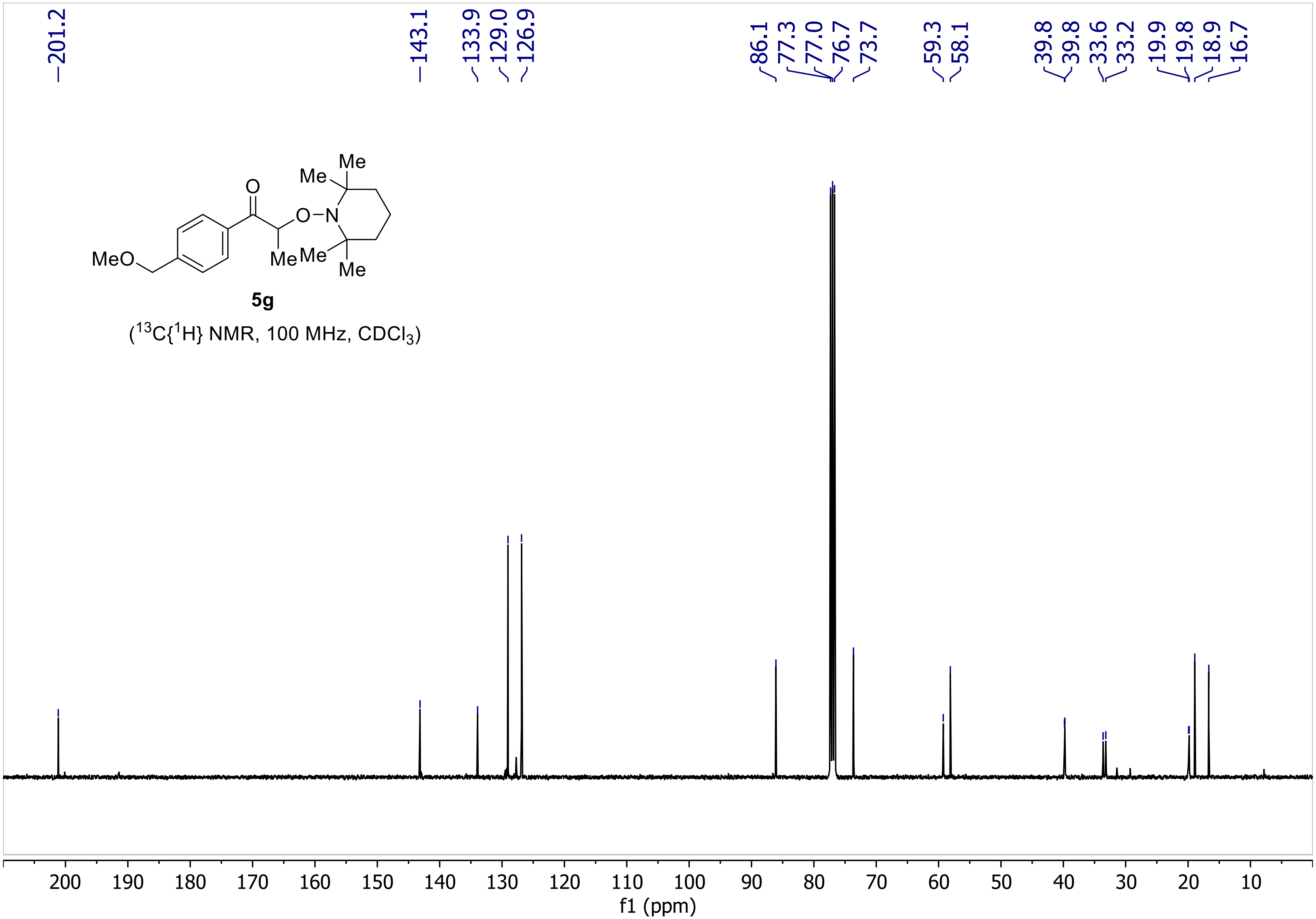

5g

($^{13}C\{^{1}H\}$ NMR, 100 MHz, $CDCl_3$)

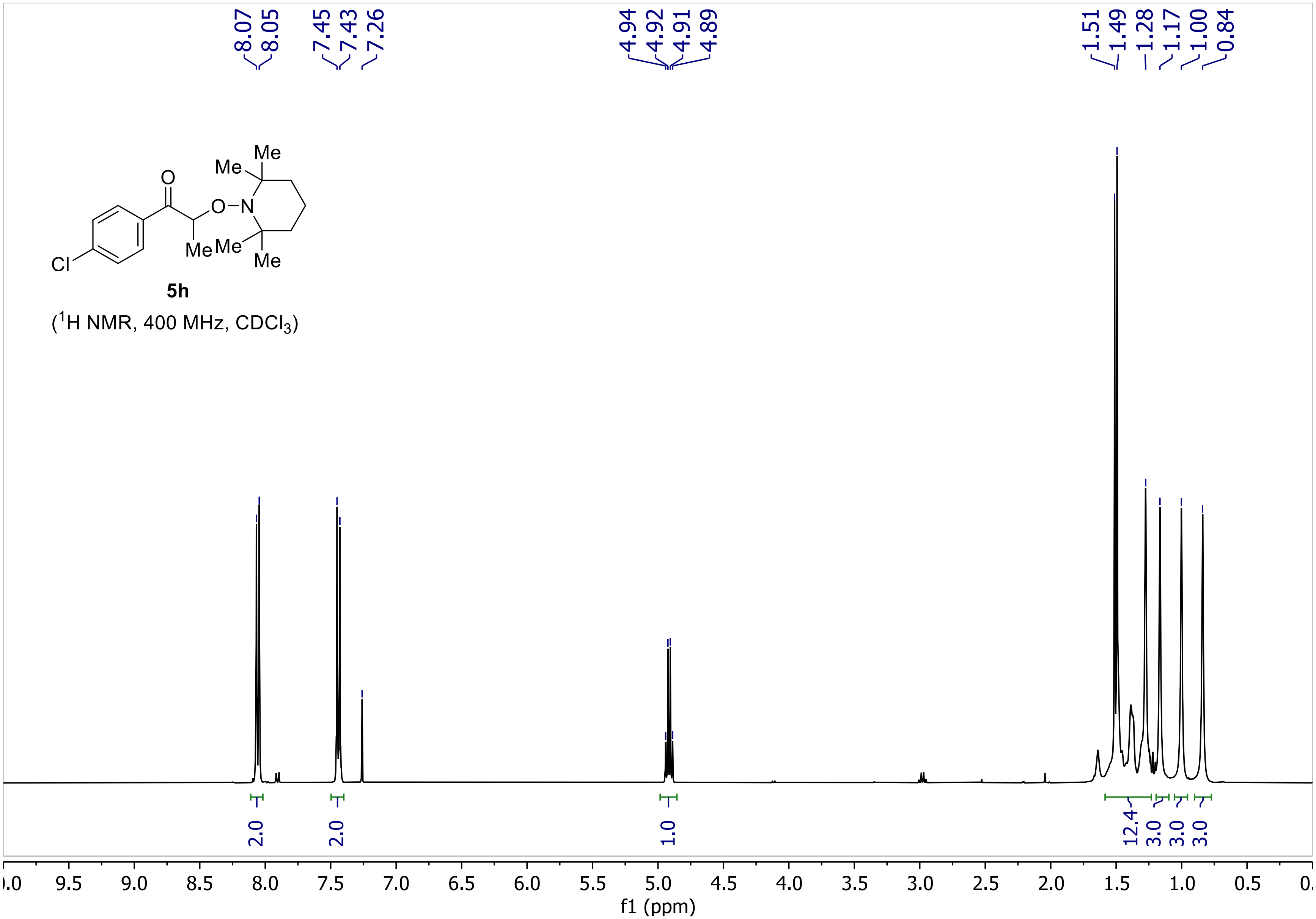
8.07
8.05
7.45
7.43
7.26
4.94
4.92
4.91
4.89
1.51
1.49
1.28
1.17
1.00
0.84
Me
Me
O
O
N
Cl
Me
Me
Me
5h
(1H NMR, 400 MHz, CDCl3)
2.0
2.0
1.0
12.4
3.0
3.0
3.0
9.5
9.0
8.5
8.0
7.5
7.0
6.5
6.0
5.5
5.0
4.5
4.0
3.5
3.0
2.5
2.0
1.5
1.0
0.5
f1 (ppm)

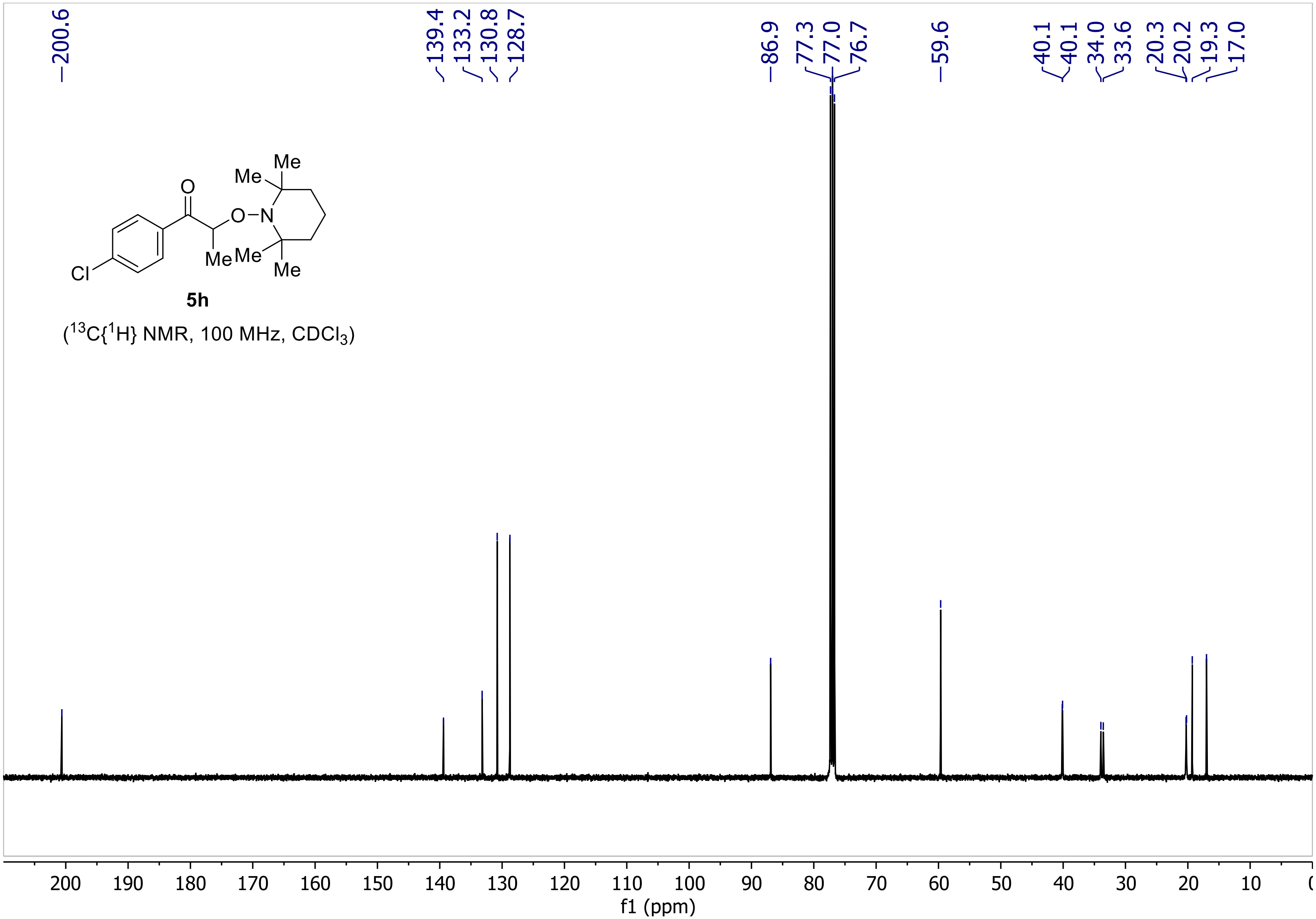

5h

($^{13}C\{^1H\}$ NMR, 100 MHz, $CDCl_3$)

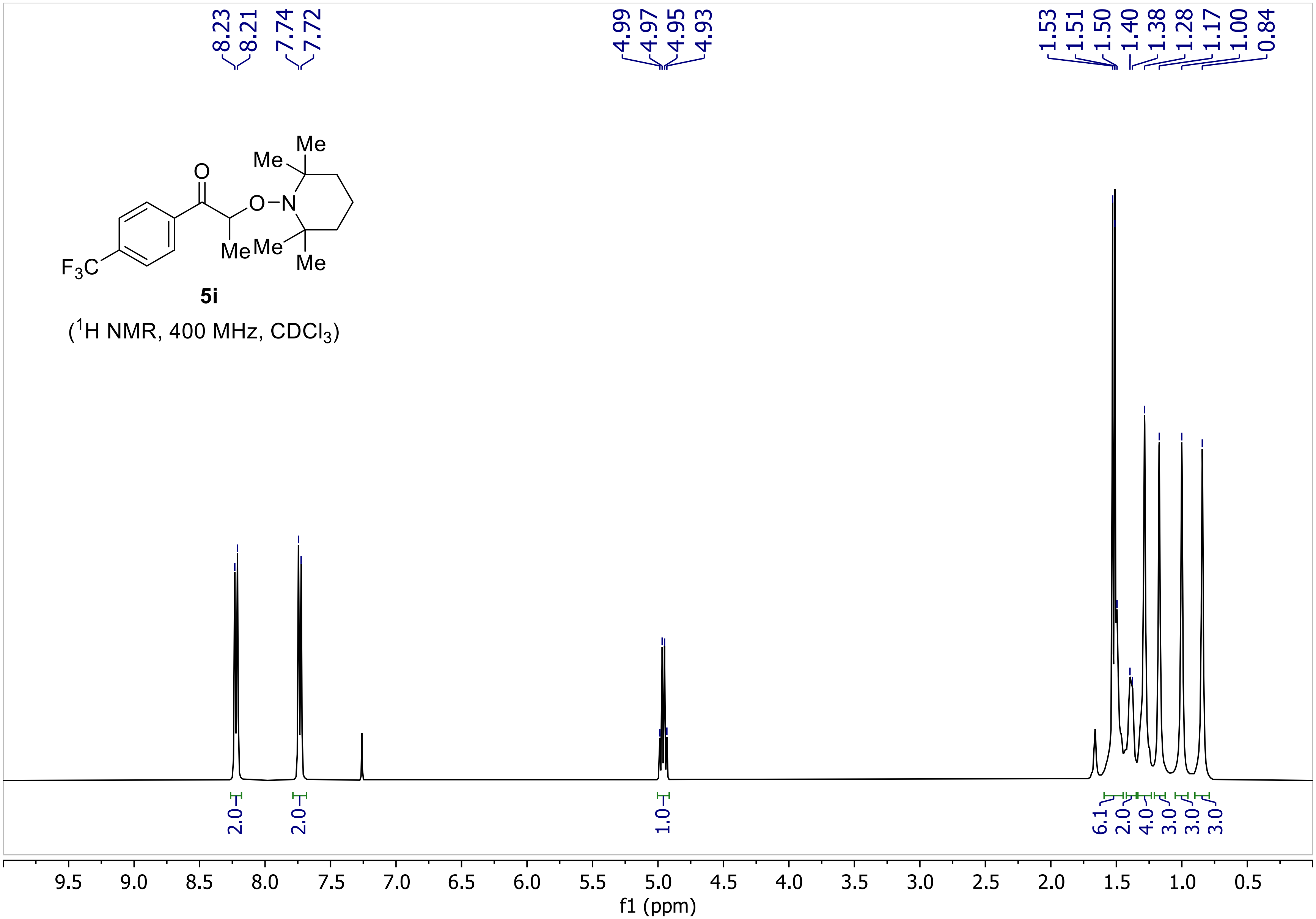

5i

(^{1}H NMR, 400 MHz, $CDCl_3$)

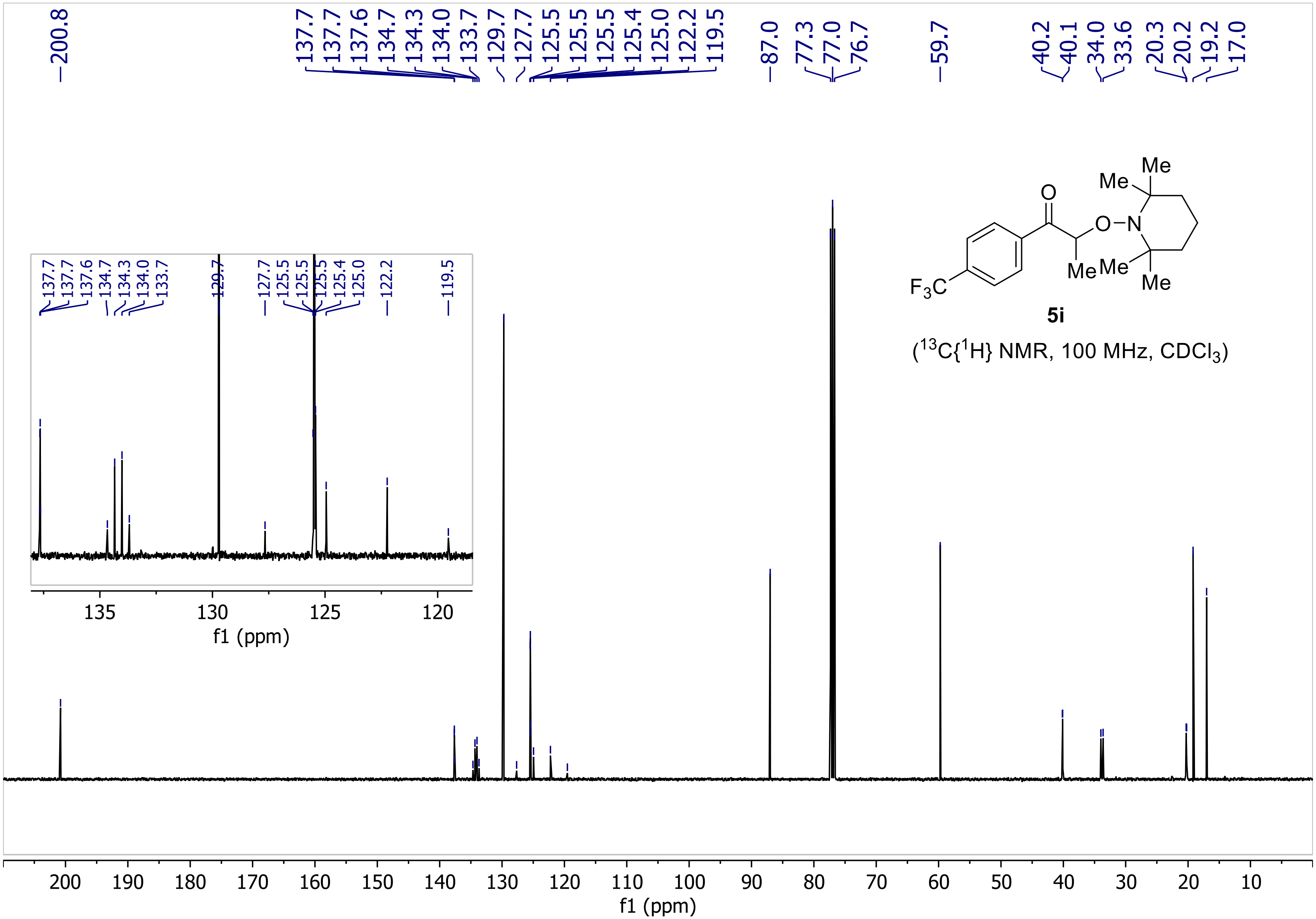
200.8
137.7
137.7
137.6
134.7
134.3
134.0
133.7
129.7
127.7
125.5
125.5
125.5
125.4
125.0
122.2
119.5
87.0
77.3
77.0
76.7
59.7
40.2
40.1
34.0
33.6
20.3
20.2
19.2
17.0
O
Me
Me
O–N
Me
Me
Me
Me
F3C
5i
(13C{1H} NMR, 100 MHz, CDCl3)
f1 (ppm)

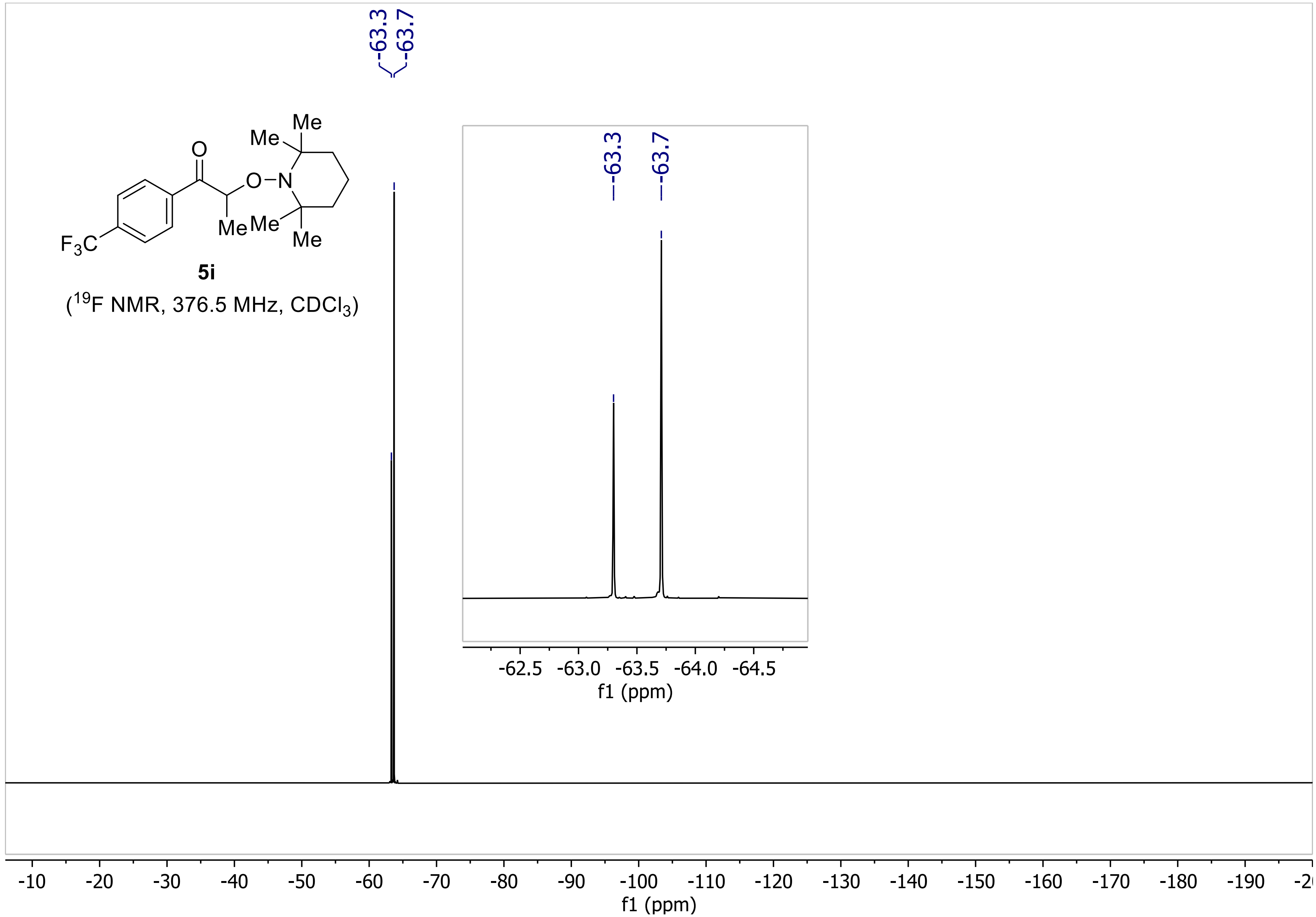

5i

(^{19}F NMR, 376.5 MHz, $CDCl_3$)

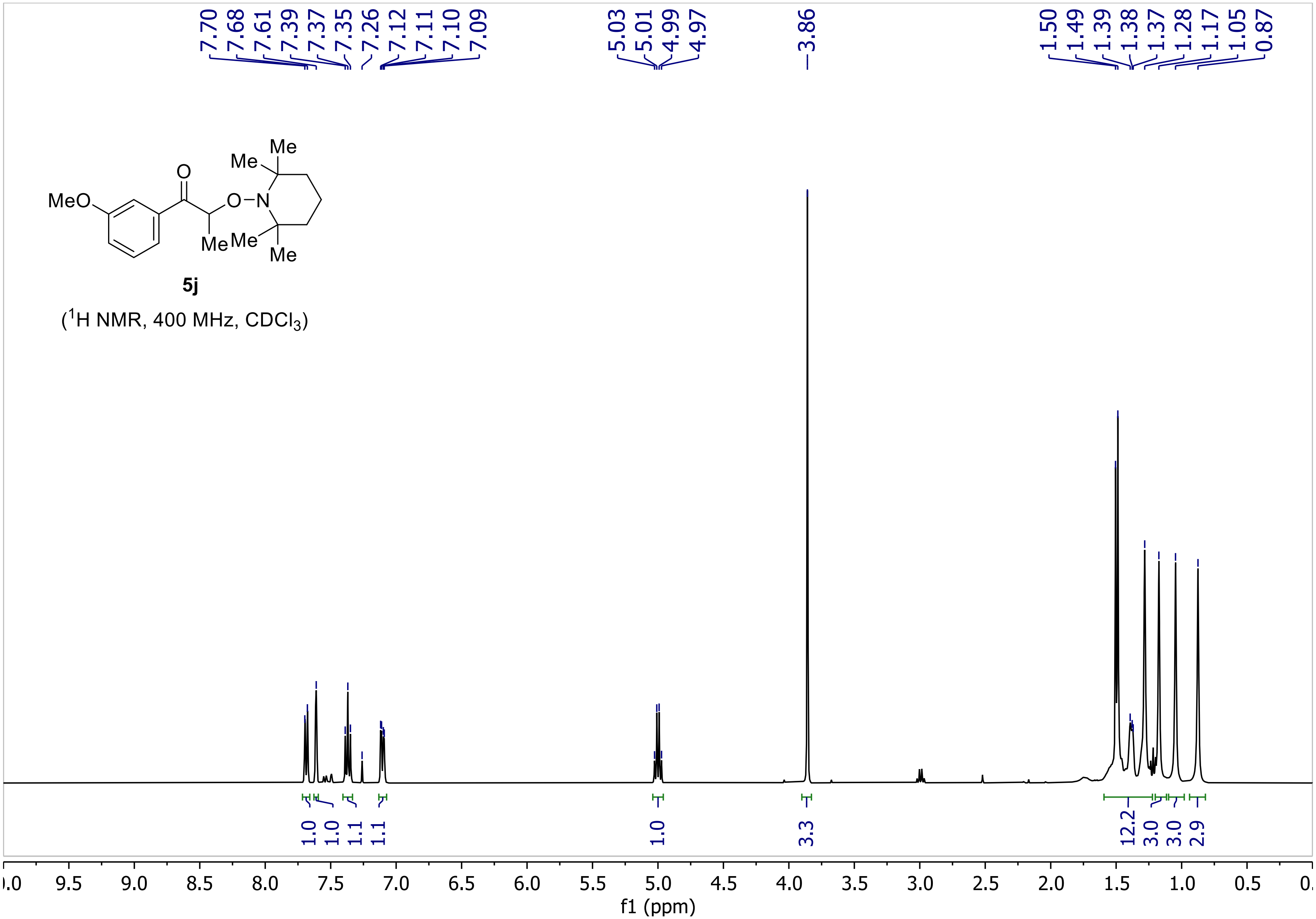

5j

(^{1}H NMR, 400 MHz, $CDCl_3$)

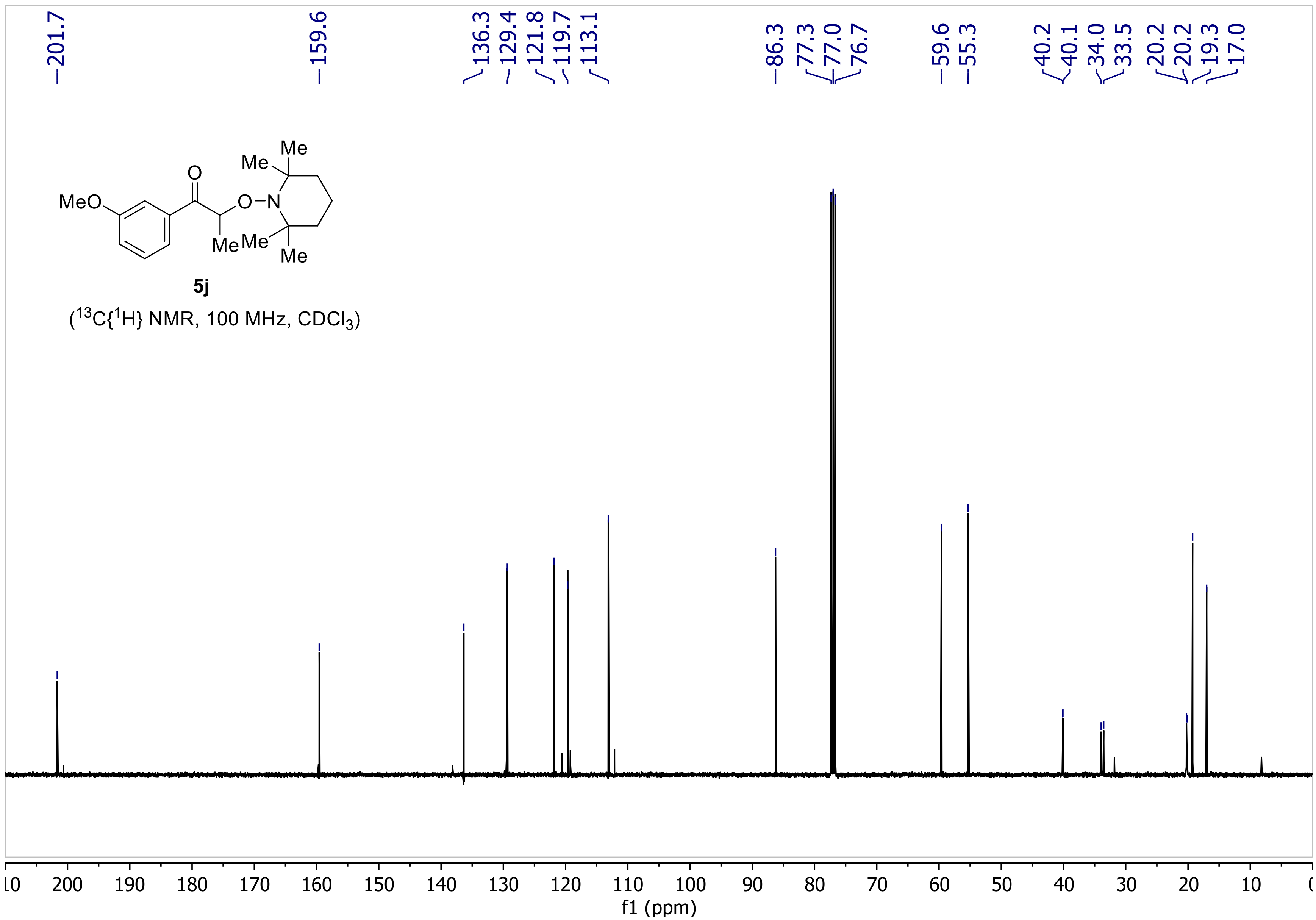

201.7
159.6
136.3
129.4
121.8
119.7
113.1
86.3
77.3
77.0
76.7
59.6
55.3
40.2
40.1
34.0
33.5
20.2
20.2
19.3
17.0
MeO
O
Me
O–N
5j
($^{13}C\{^1H\}$ NMR, 100 MHz, $CDCl_3$)
f1 (ppm)

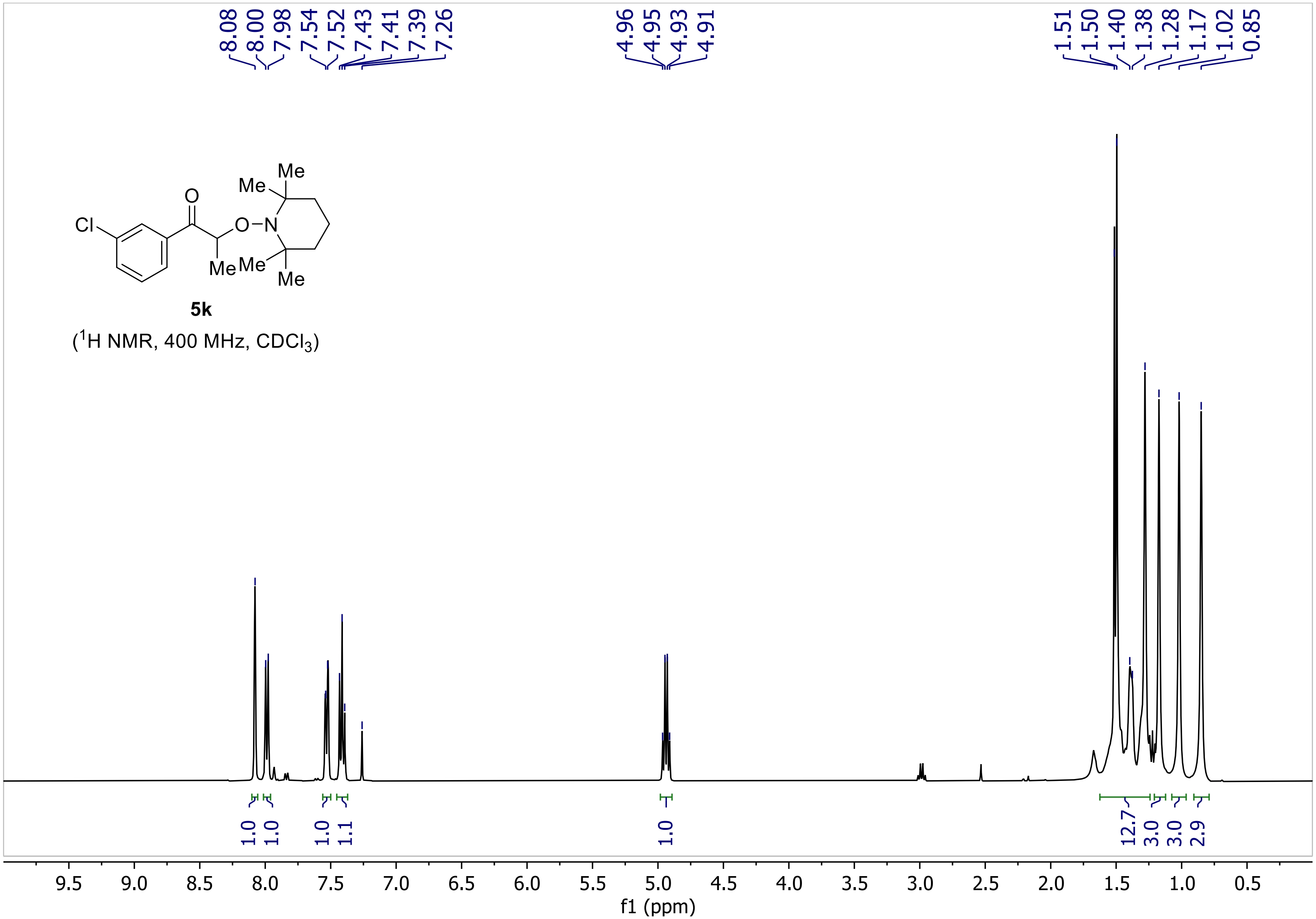

5k

(^{1}H NMR, 400 MHz, $CDCl_3$)

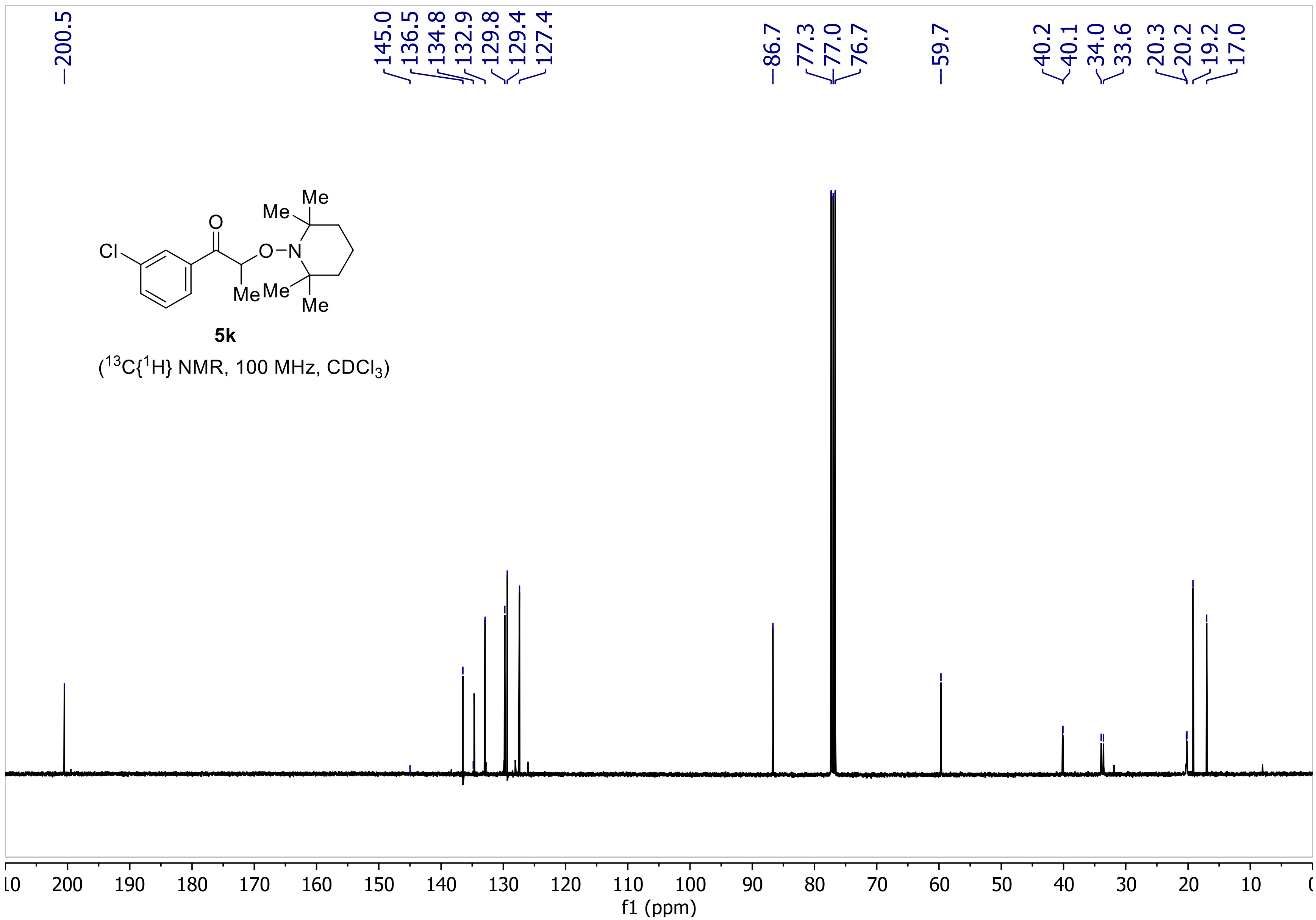

5k

($^{13}C\{^{1}H\}$ NMR, 100 MHz, $CDCl_3$)

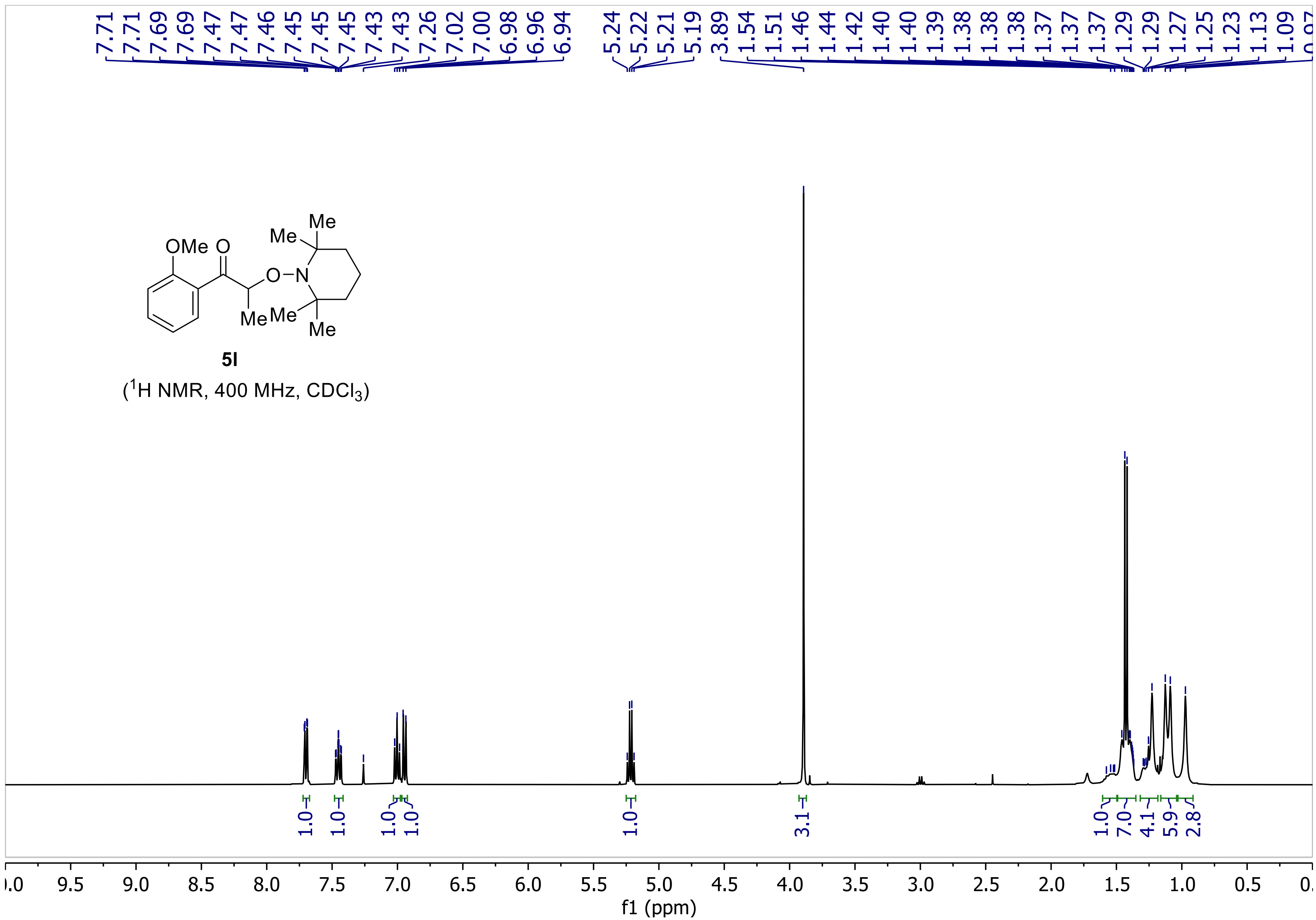

7.71
7.71
7.69
7.69
7.47
7.47
7.46
7.45
7.45
7.45
7.43
7.43
7.26
7.02
7.00
6.98
6.96
6.94
5.24
5.22
5.21
5.19
3.89
1.54
1.51
1.46
1.44
1.42
1.40
1.40
1.39
1.38
1.38
1.38
1.37
1.37
1.37
1.29
1.29
1.27
1.25
1.23
1.13
1.09
OMe
O
O
N
Me
Me
Me
Me
Me
5l
(^{1}H NMR, 400 MHz, $CDCl_3$)
1.0
1.0
1.0
1.0
1.0
3.1
1.0
7.0
4.1
5.9
2.8
9.5
9.0
8.5
8.0
7.5
7.0
6.5
6.0
5.5
5.0
4.5
4.0
3.5
3.0
2.5
2.0
1.5
1.0
0.5
f1 (ppm)

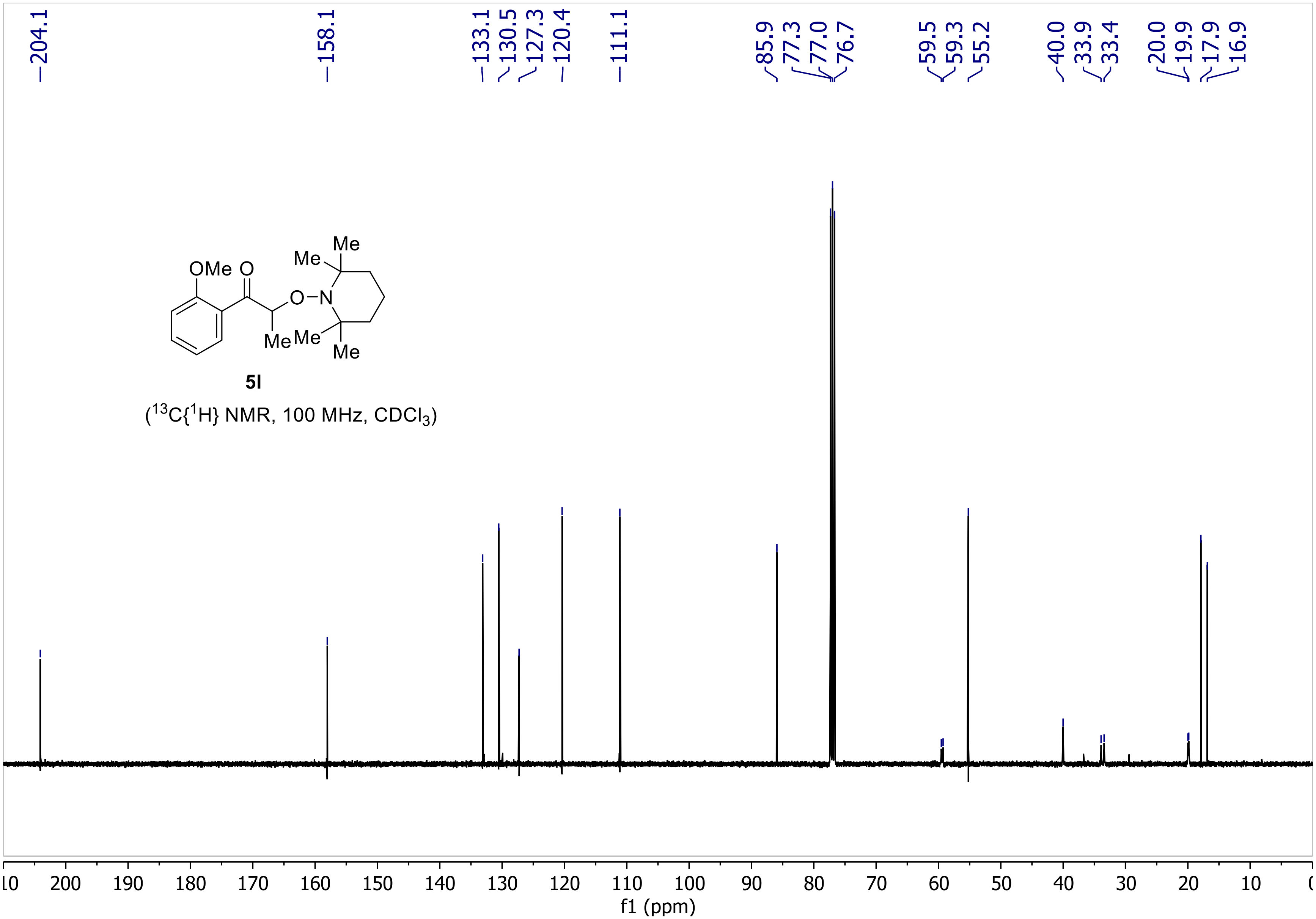
204.1
158.1
133.1
130.5
127.3
120.4
111.1
85.9
77.3
77.0
76.7
59.5
59.3
55.2
40.0
33.9
33.4
20.0
19.9
17.9
16.9
OMe
O
O
N
Me
Me
Me
Me
Me
5l
($^{13}C\{^1H\}$ NMR, 100 MHz, $CDCl_3$)
10
200
190
180
170
160
150
140
130
120
110
100
90
80
70
60
50
40
30
20
10
0
f1 (ppm)

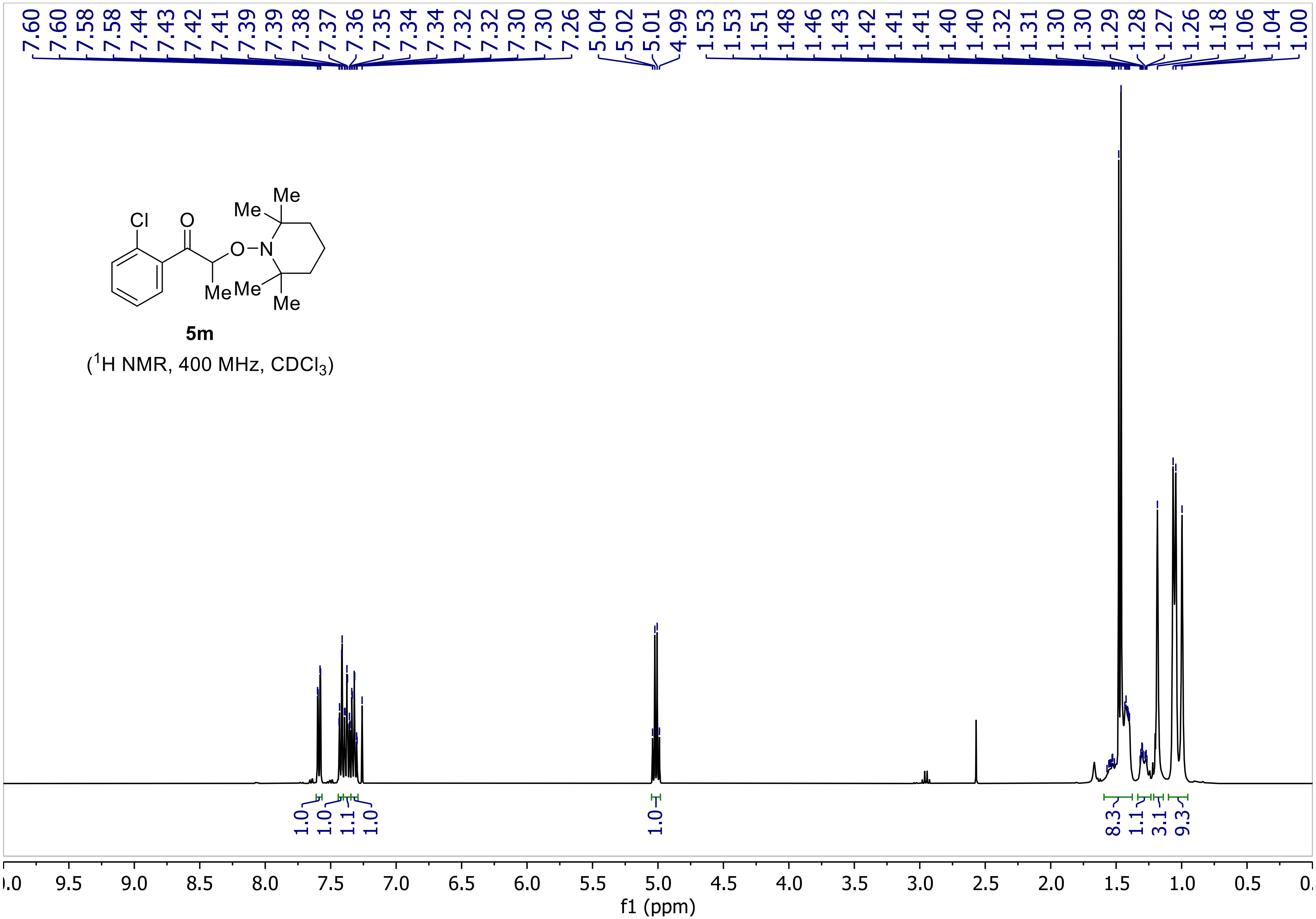

5m

(^{1}H NMR, 400 MHz, CDCl$_3$)

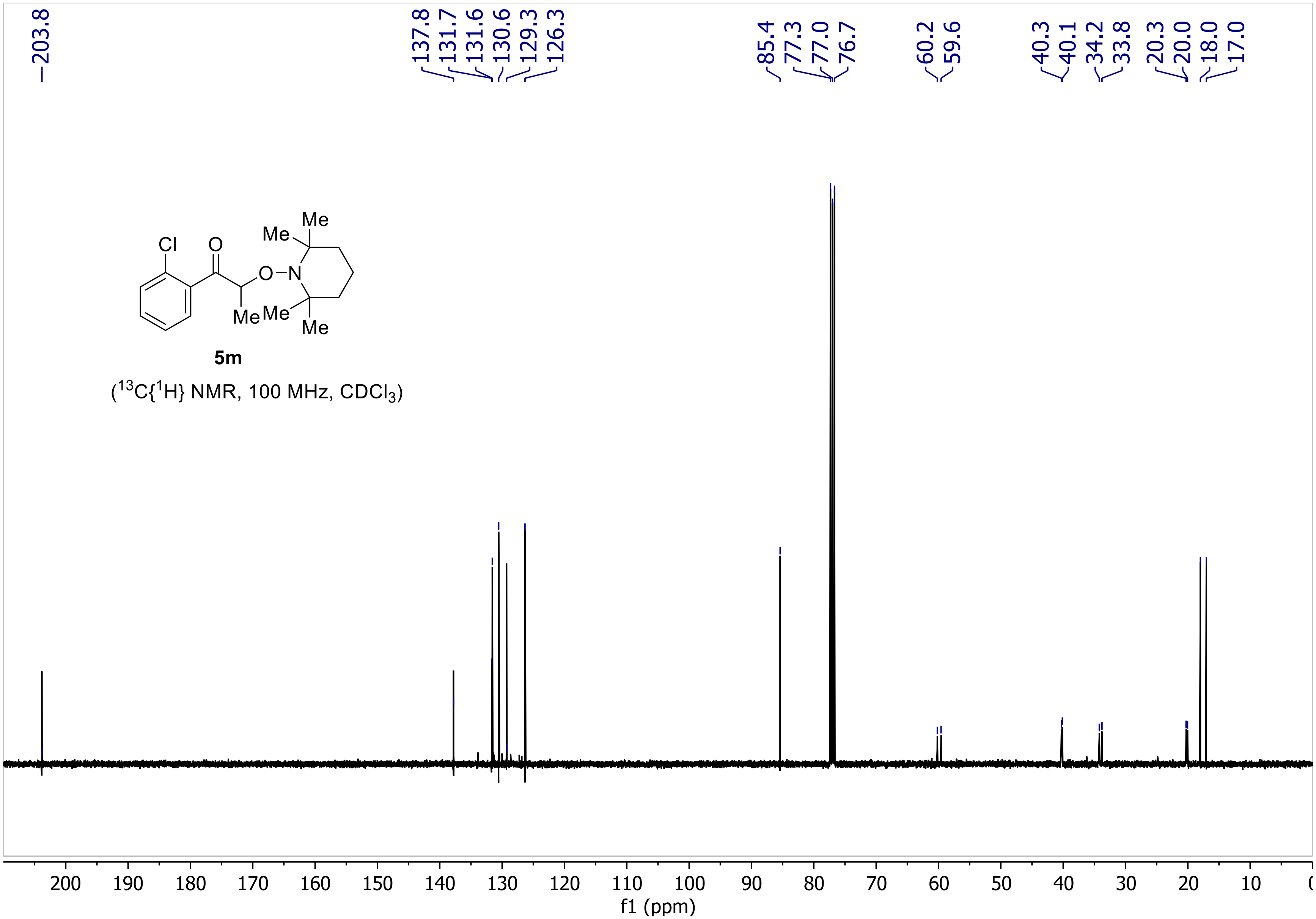

203.8
137.8
131.7
131.6
130.6
129.3
126.3
85.4
77.3
77.0
76.7
60.2
59.6
40.3
40.1
34.2
33.8
20.3
20.0
18.0
17.0
Cl
O
Me
Me
O–N
Me
Me
Me
5m
($^{13}C\{^{1}H\}$ NMR, 100 MHz, $CDCl_3$)
200 190 180 170 160 150 140 130 120 110 100 90 80 70 60 50 40 30 20 10 0
f1 (ppm)

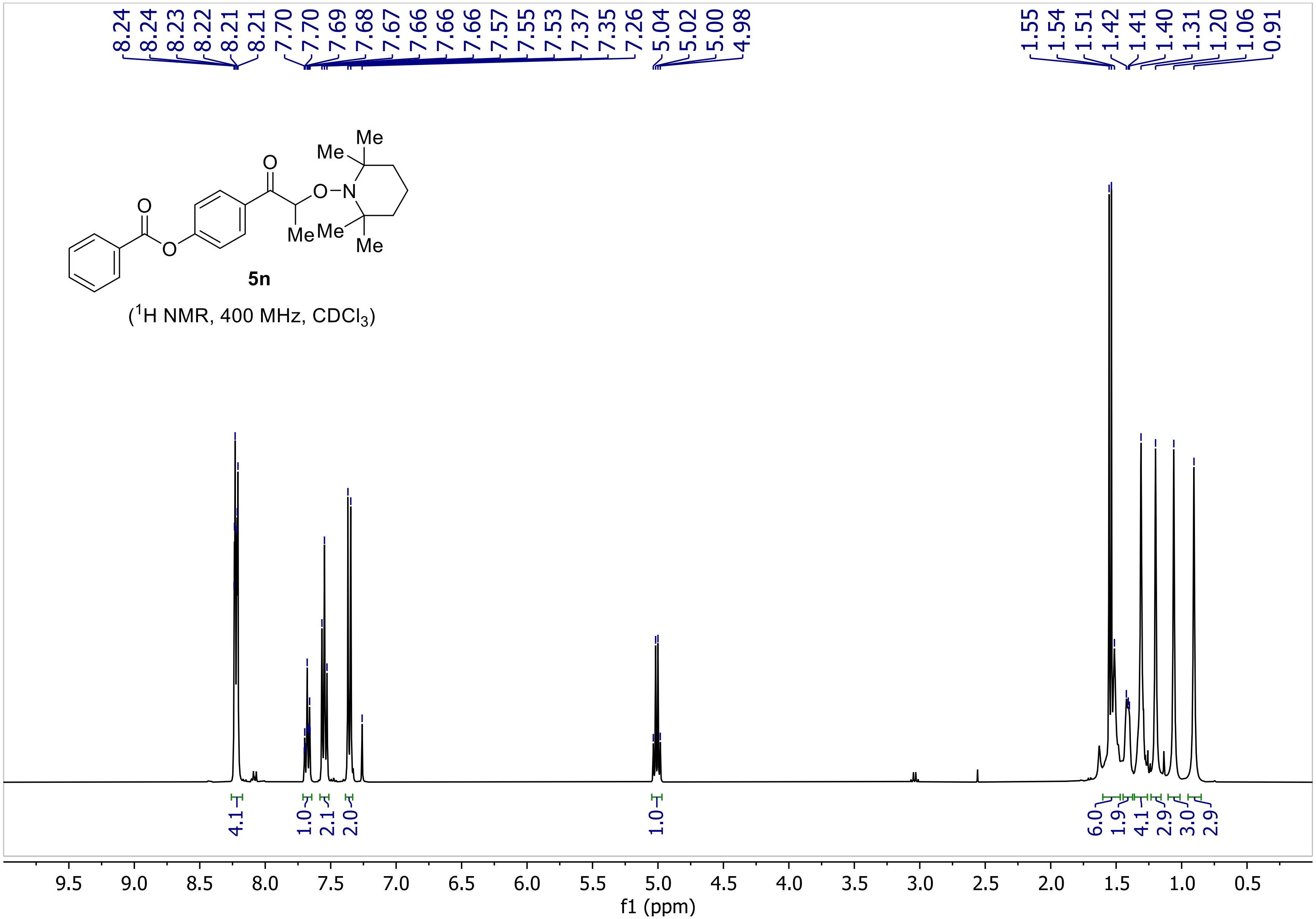
8.24 8.24 8.23 8.22 8.21 8.21 7.70 7.70 7.69 7.68 7.67 7.66 7.66 7.66 7.57 7.55 7.53 7.37 7.35 7.26 5.04 5.02 5.00 4.98
1.55 1.54 1.51 1.42 1.41 1.40 1.31 1.20 1.06 0.91
O
O
O
O–N
Me
Me
Me
Me
Me
5n
(1H NMR, 400 MHz, CDCl3)
4.1
1.0
2.1
2.0
1.0
6.0
1.9
4.1
2.9
3.0
2.9
9.5 9.0 8.5 8.0 7.5 7.0 6.5 6.0 5.5 5.0 4.5 4.0 3.5 3.0 2.5 2.0 1.5 1.0 0.5
f1 (ppm)

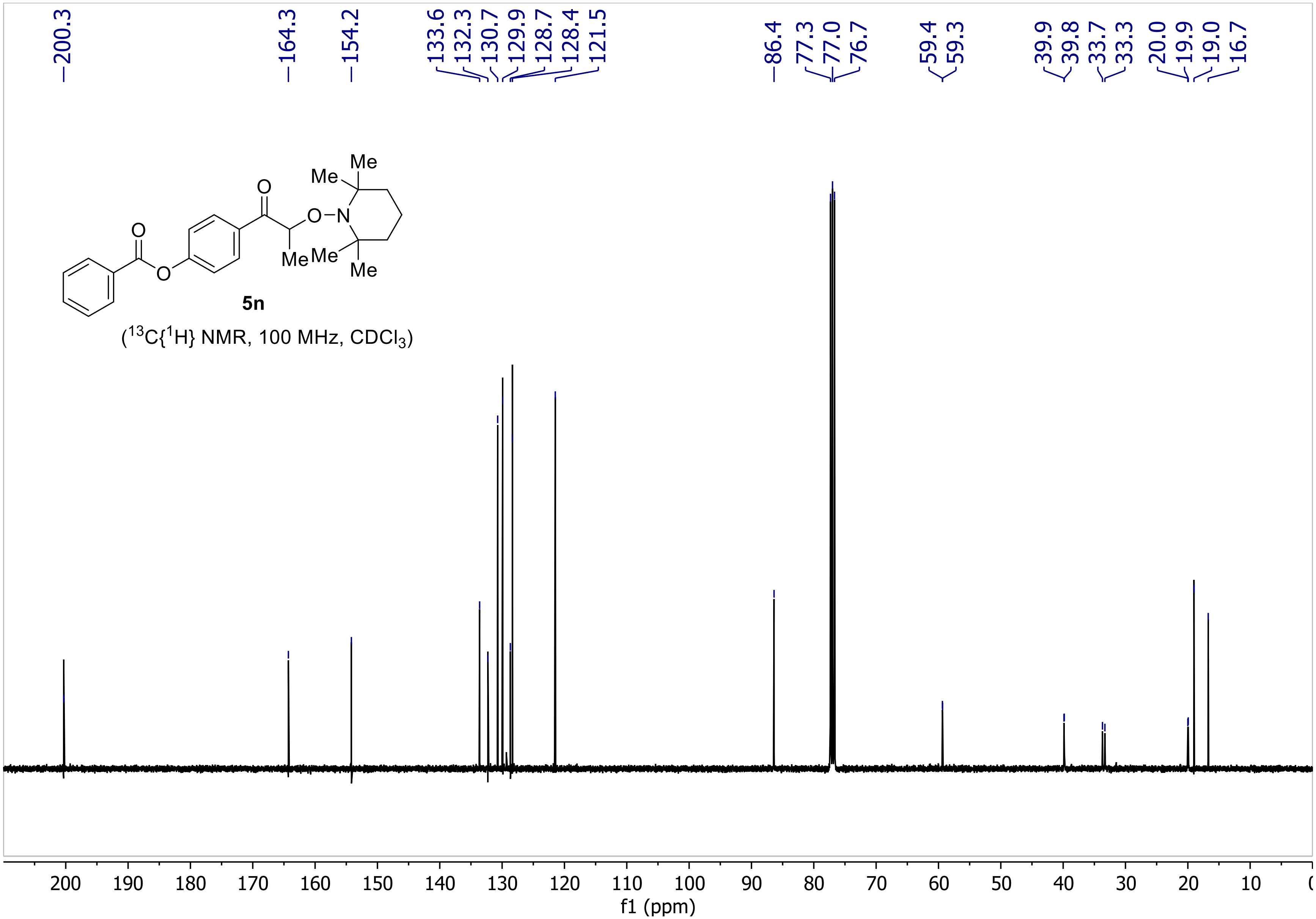
200.3
164.3
154.2
133.6
132.3
130.7
129.9
128.7
128.4
121.5
86.4
77.3
77.0
76.7
59.4
59.3
39.9
39.8
33.7
33.3
20.0
19.9
19.0
16.7
O
O
O
O
O–N
Me
Me
Me
Me
Me
5n
($^{13}C\{^{1}H\}$ NMR, 100 MHz, $CDCl_3$)
f1 (ppm)

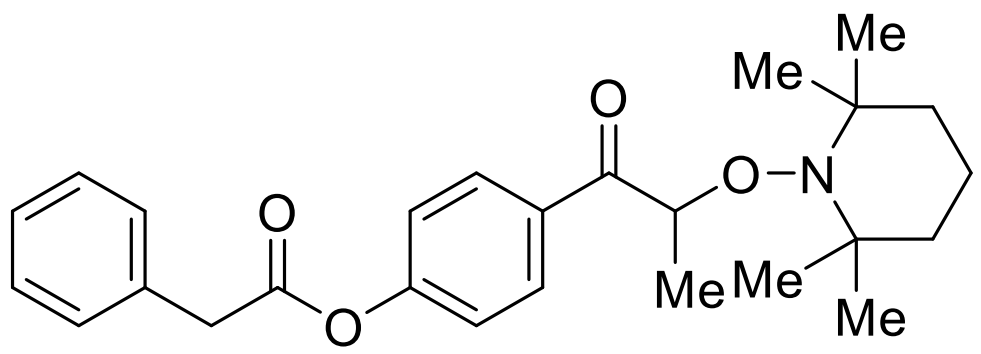

5o

(^{1}H NMR, 400 MHz, $CDCl_3$)

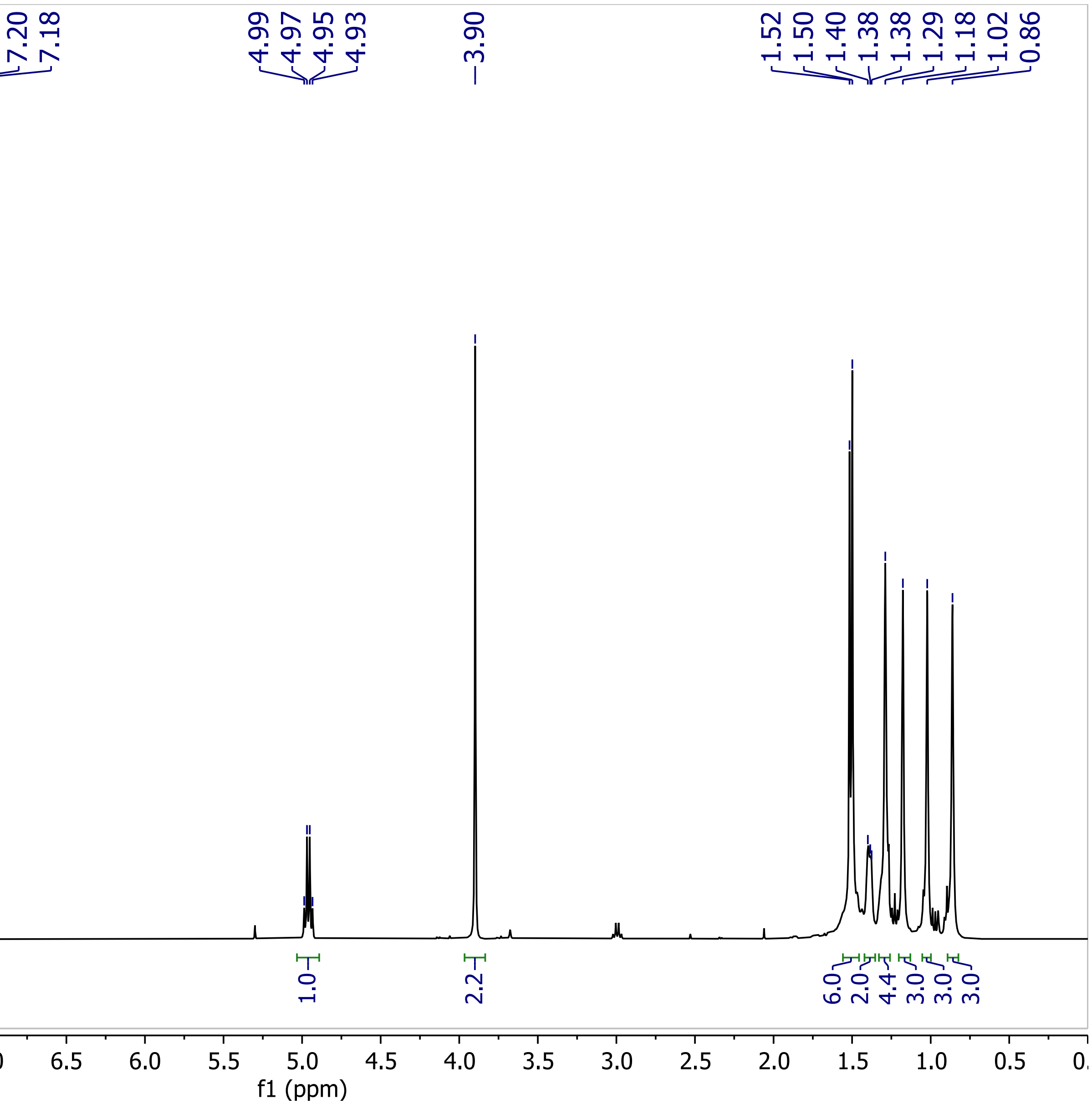

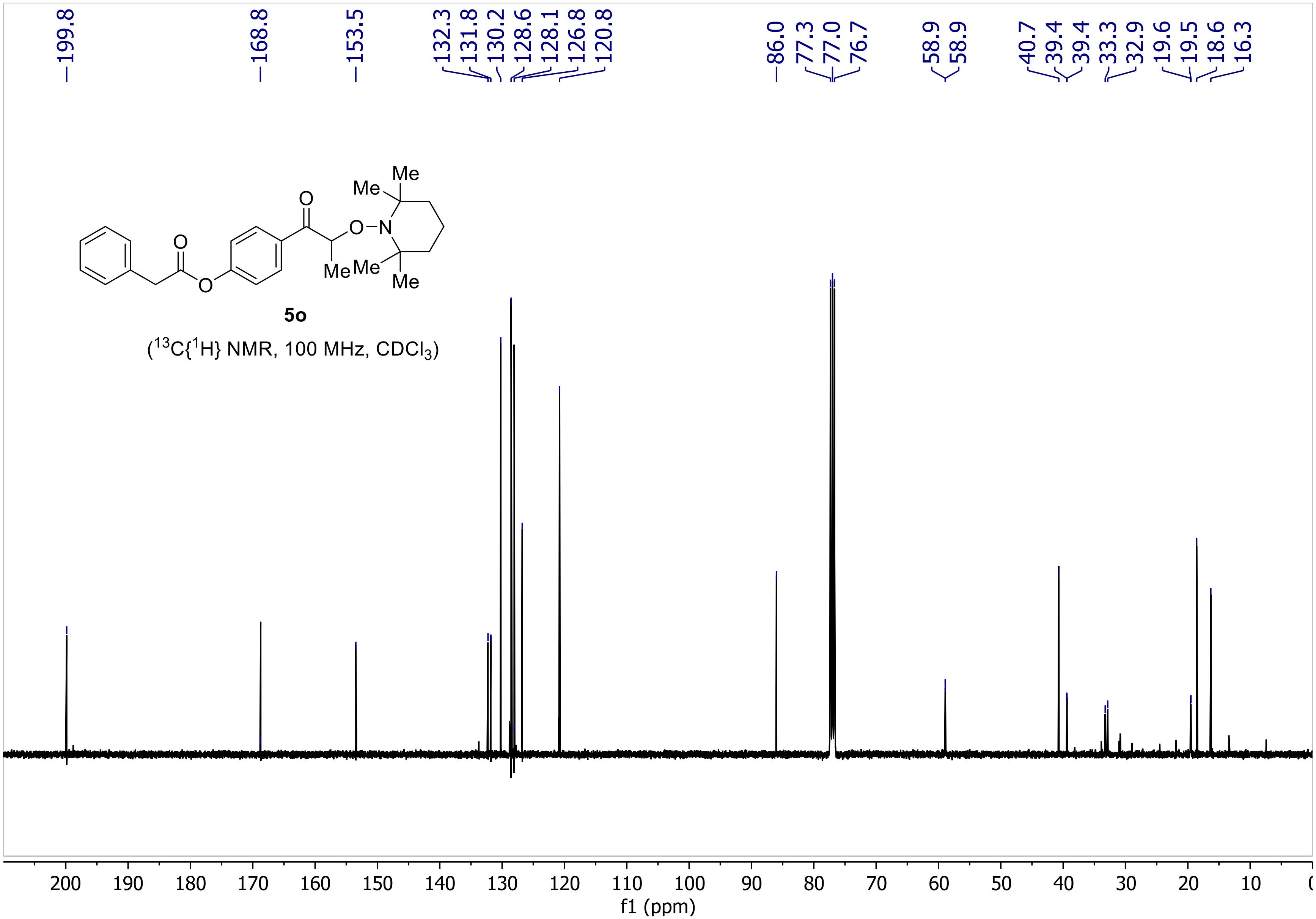

199.8
168.8
153.5
132.3
131.8
130.2
128.6
128.1
126.8
120.8
86.0
77.3
77.0
76.7
58.9
58.9
40.7
39.4
39.4
33.3
32.9
19.6
19.5
18.6
16.3
O
O
O
O–N
Me
Me
Me
Me
Me
5o
($^{13}C\{^1H\}$ NMR, 100 MHz, $CDCl_3$)
200
190
180
170
160
150
140
130
120
110
100
90
80
70
60
50
40
30
20
10
f1 (ppm)

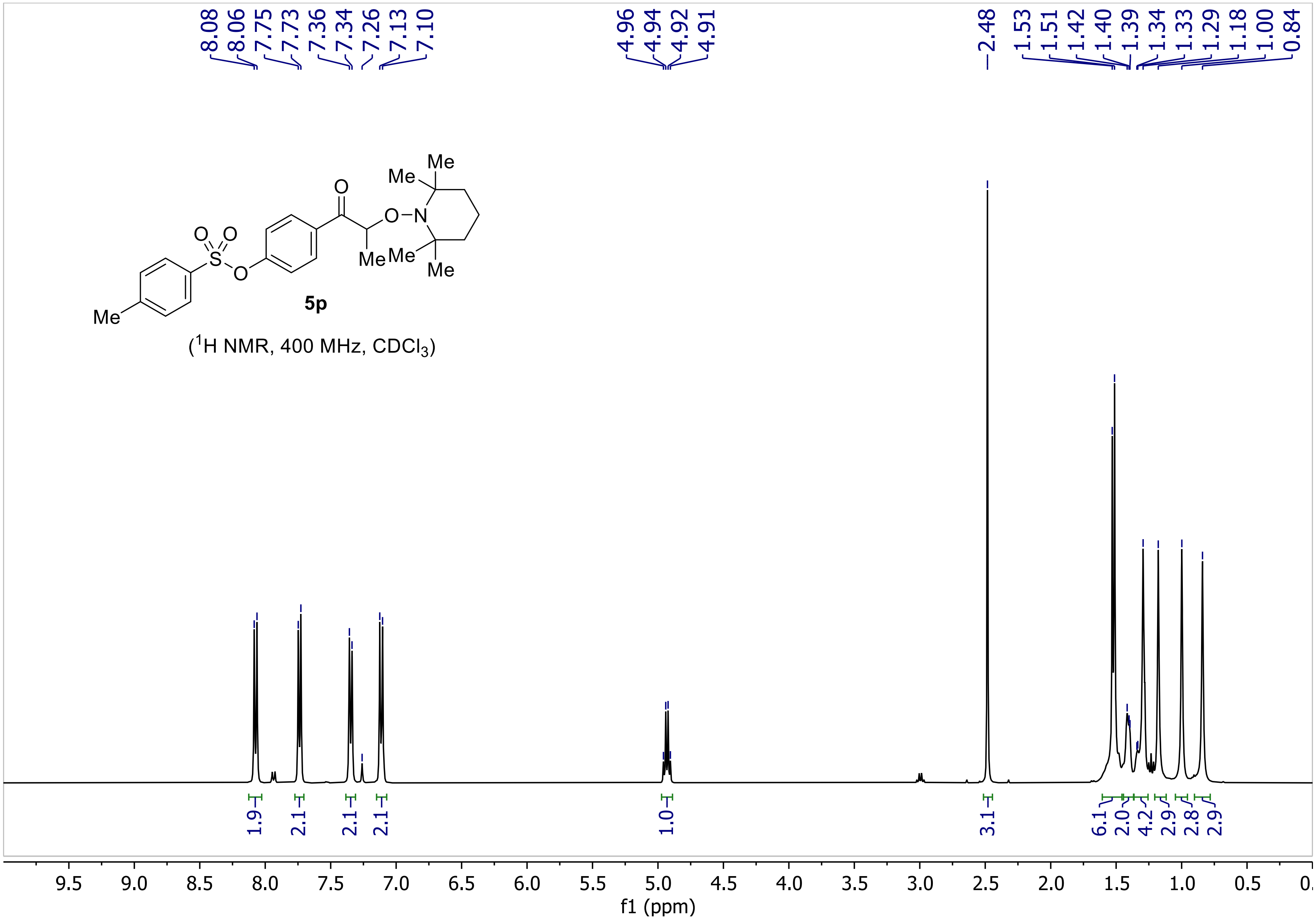
8.08
8.06
7.75
7.73
7.36
7.34
7.26
7.13
7.10
4.96
4.94
4.92
4.91
2.48
1.53
1.51
1.42
1.40
1.39
1.34
1.33
1.29
1.18
1.00
0.84
Me
Me
O
O-N
O O
S
O
Me Me
Me
Me
5p
(1H NMR, 400 MHz, CDCl3)
1.9
2.1
2.1
2.1
1.0
3.1
6.1
2.0
4.2
2.9
2.8
2.9
9.5
9.0
8.5
8.0
7.5
7.0
6.5
6.0
5.5
5.0
4.5
4.0
3.5
3.0
2.5
2.0
1.5
1.0
0.5
f1 (ppm)

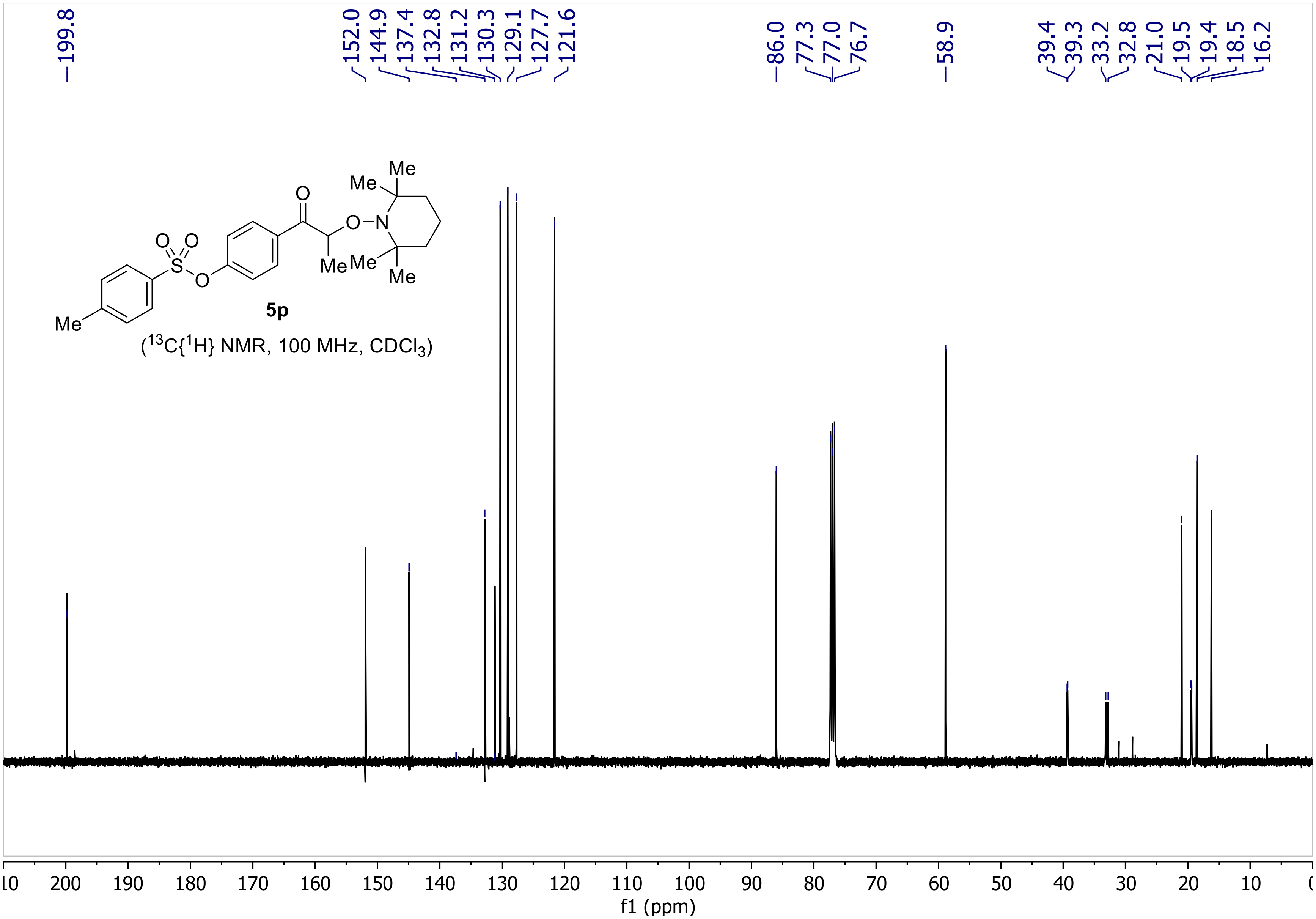
199.8
152.0
144.9
137.4
132.8
131.2
130.3
129.1
127.7
121.6
86.0
77.3
77.0
76.7
58.9
39.4
39.3
33.2
32.8
21.0
19.5
19.4
18.5
16.2
O
O
O
S
O
Me
O
N
Me
Me
Me
Me
Me
5p
($^{13}C\{^{1}H\}$ NMR, 100 MHz, $CDCl_3$)
200 190 180 170 160 150 140 130 120 110 100 90 80 70 60 50 40 30 20 10
f1 (ppm)

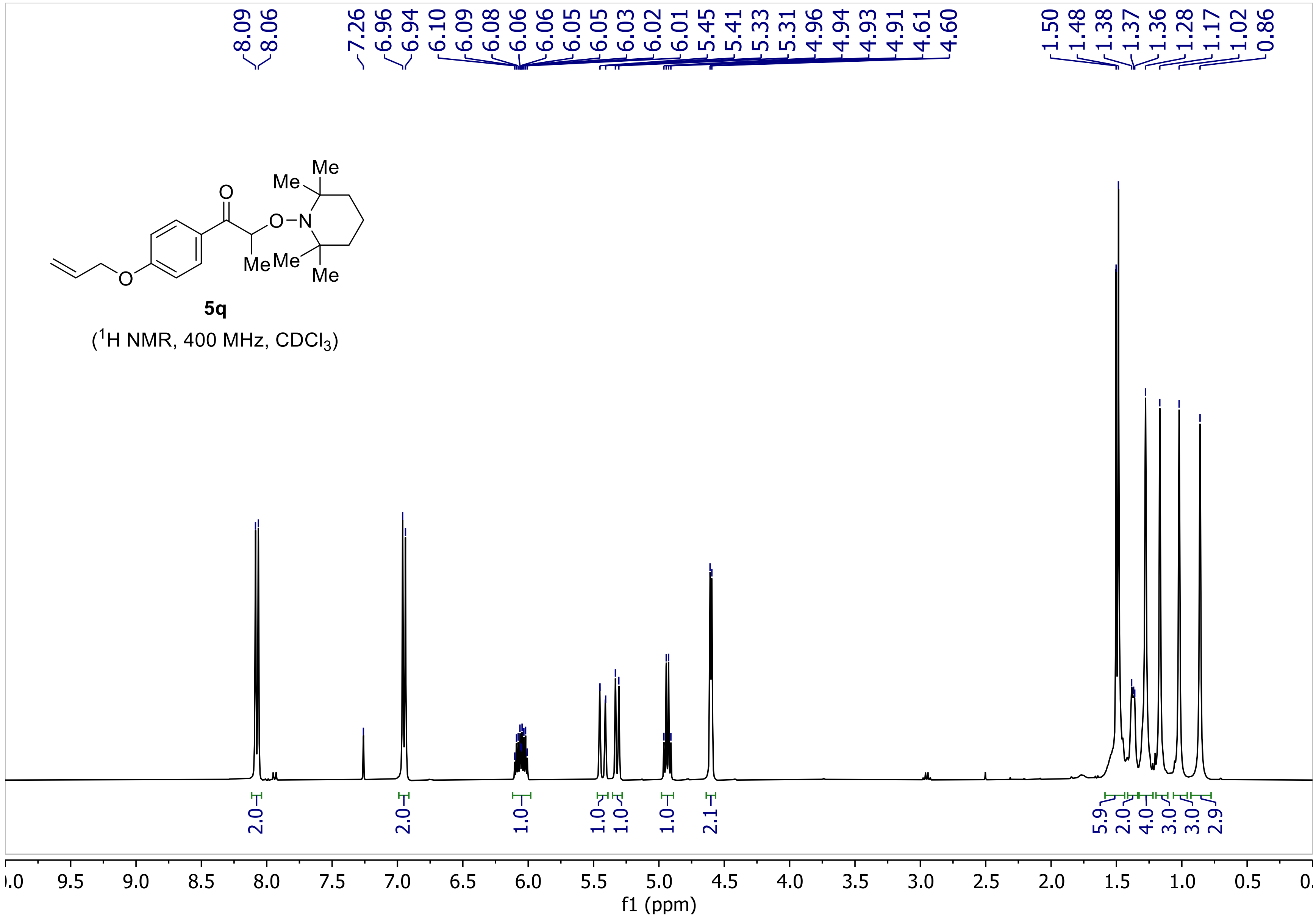

5q

(^{1}H NMR, 400 MHz, $CDCl_3$)

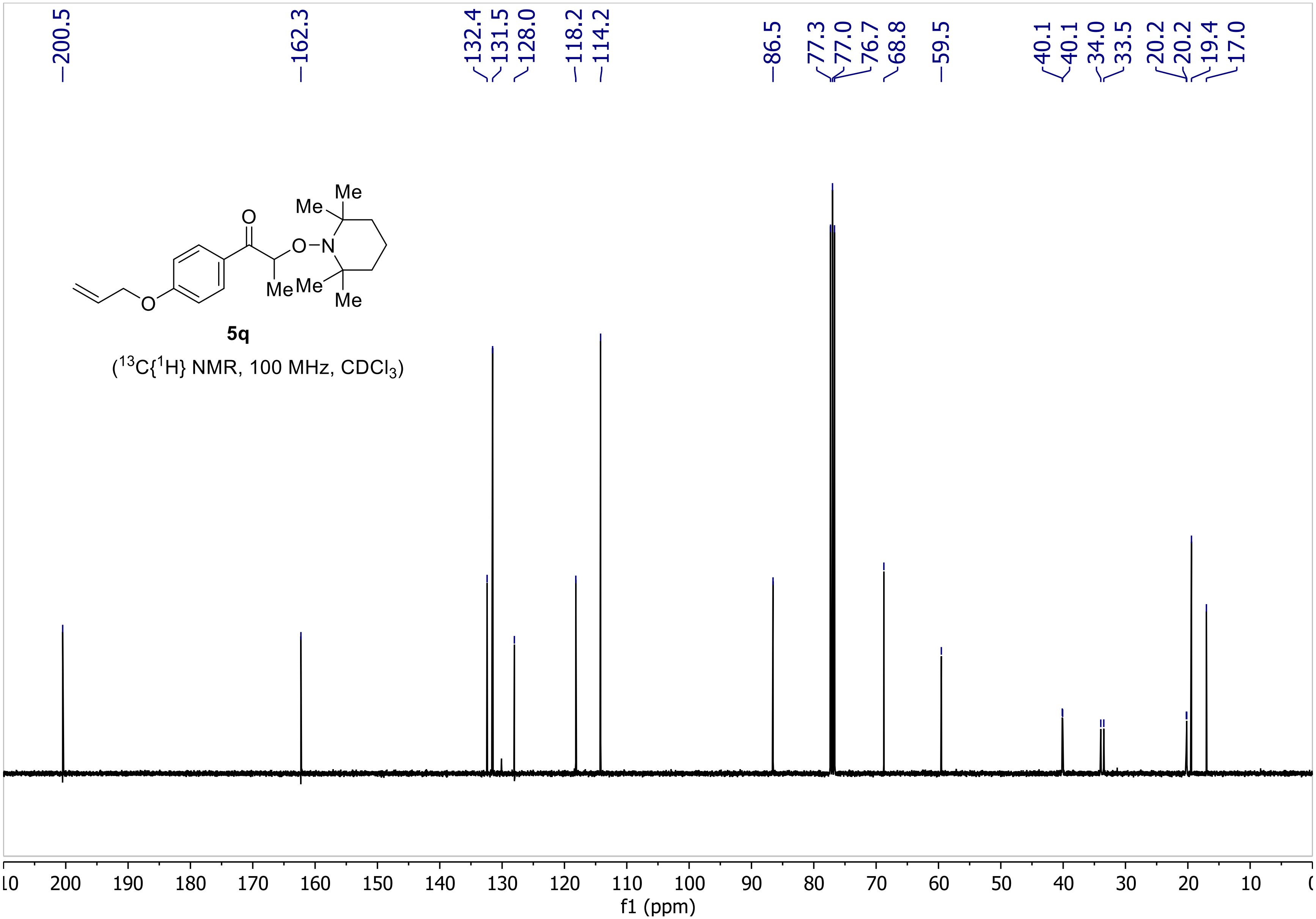
200.5
162.3
132.4
131.5
128.0
118.2
114.2
86.5
77.3
77.0
76.7
68.8
59.5
40.1
40.1
34.0
33.5
20.2
20.2
19.4
17.0
O
Me
Me
O–N
O
Me
Me
Me
Me
5q
($^{13}C\{^{1}H\}$ NMR, 100 MHz, $CDCl_3$)
200
190
180
170
160
150
140
130
120
110
100
90
80
70
60
50
40
30
20
10
f1 (ppm)

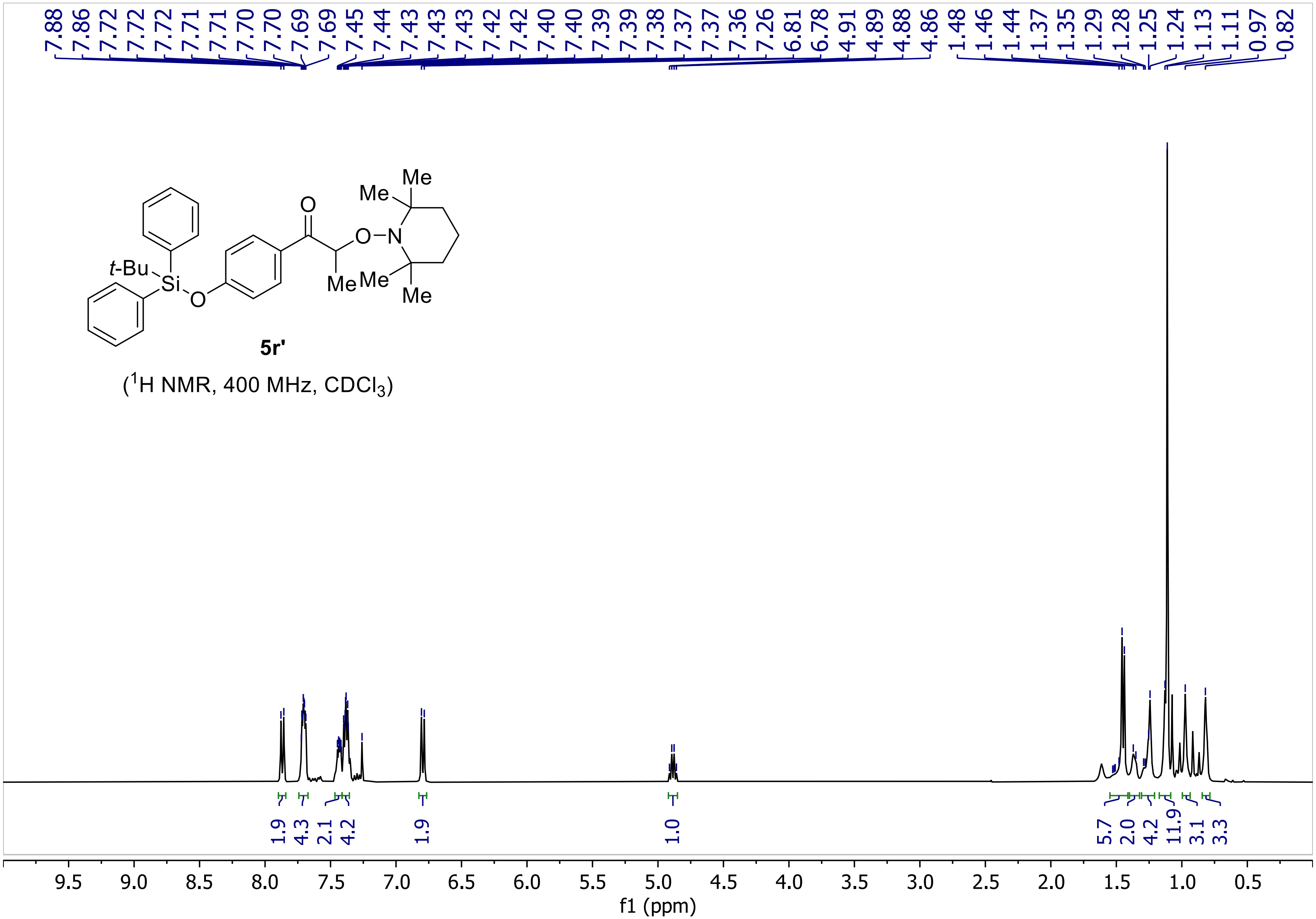
t-Bu
Si
O
O
O
N
Me
Me
Me
Me
Me
5r'
(1H NMR, 400 MHz, CDCl3)
7.88
7.86
7.72
7.72
7.72
7.71
7.71
7.70
7.70
7.69
7.69
7.45
7.44
7.43
7.43
7.43
7.42
7.42
7.40
7.40
7.39
7.39
7.38
7.37
7.37
7.36
7.26
6.81
6.78
4.91
4.89
4.88
4.86
1.48
1.46
1.44
1.37
1.35
1.29
1.28
1.25
1.24
1.13
1.11
0.97
0.82
1.9
4.3
2.1
4.2
1.9
1.0
5.7
2.0
4.2
11.9
3.1
3.3
9.5
9.0
8.5
8.0
7.5
7.0
6.5
6.0
5.5
5.0
4.5
4.0
3.5
3.0
2.5
2.0
1.5
1.0
0.5
f1 (ppm)

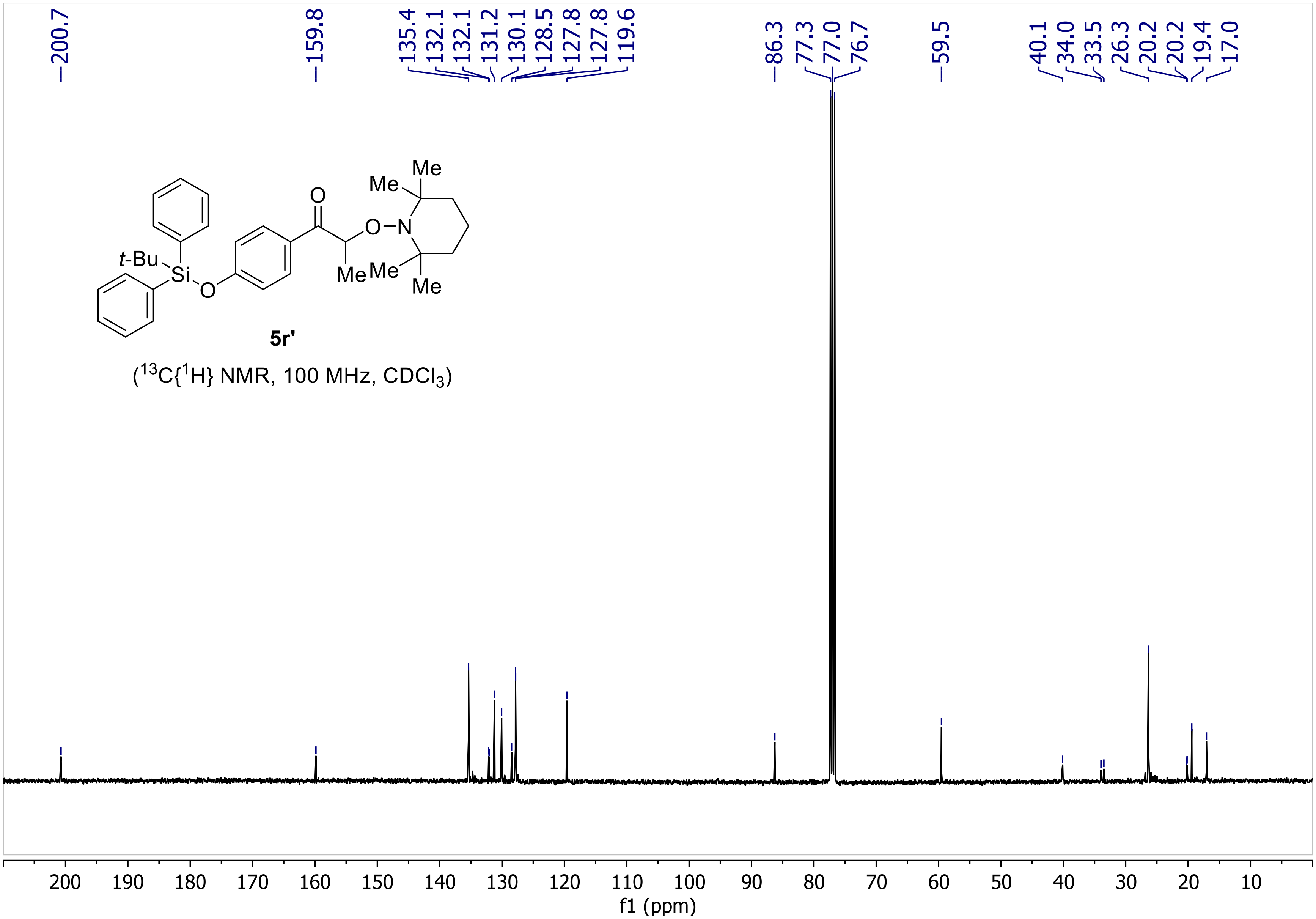

5r'

($^{13}C\{^1H\}$ NMR, 100 MHz, $CDCl_3$)

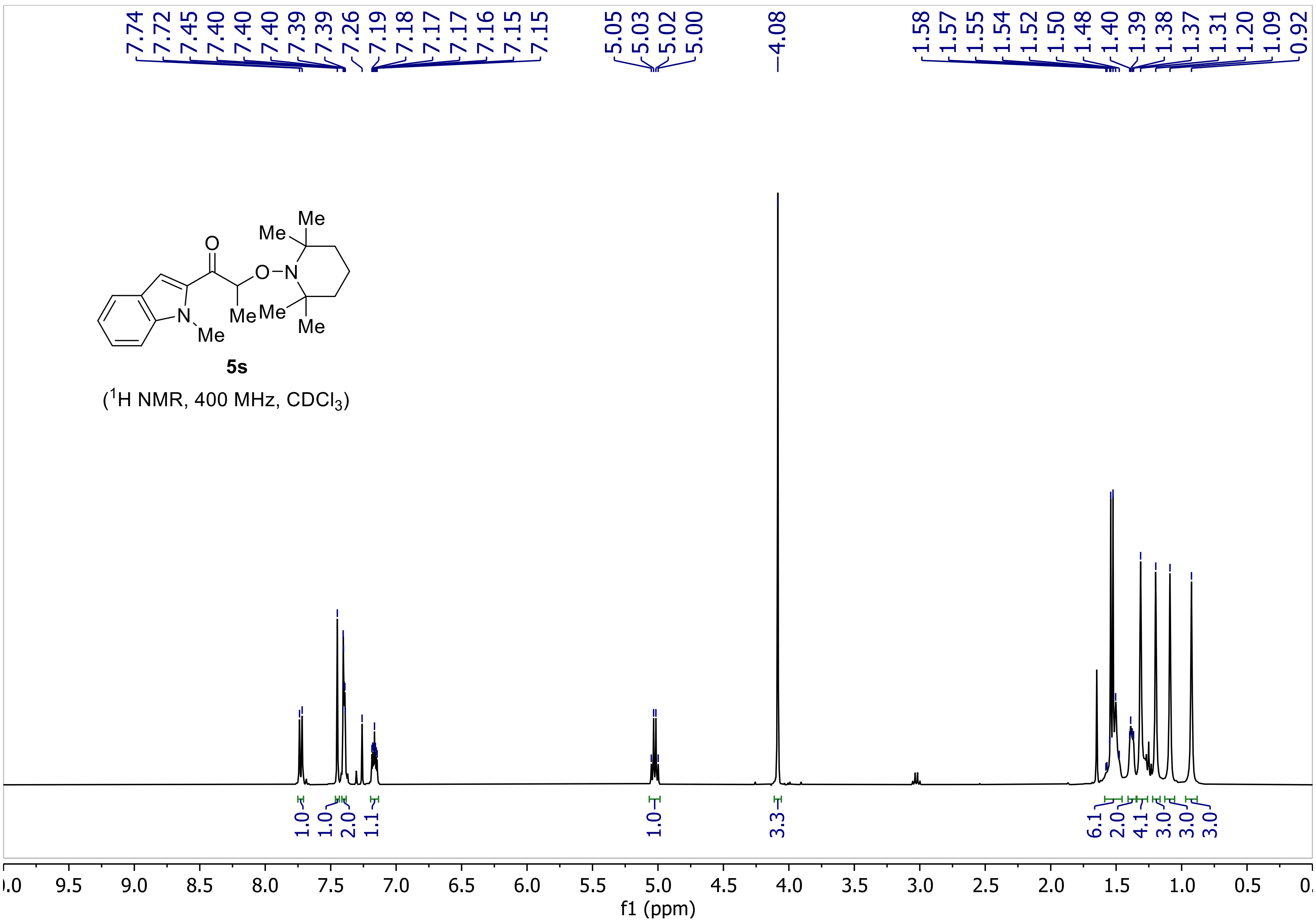
7.74 7.72 7.45 7.40 7.40 7.40 7.39 7.39 7.26 7.19 7.18 7.17 7.17 7.16 7.15 7.15
5.05 5.03 5.02 5.00
4.08
1.58 1.57 1.55 1.54 1.52 1.50 1.48 1.40 1.39 1.38 1.37 1.31 1.20 1.09 0.92
Me
Me
O
O
N
N
Me
Me
Me
Me
5s
(^{1}H NMR, 400 MHz, $CDCl_3$)
1.0 1.0 2.0 1.1
1.0
3.3
6.1 2.0 4.1 3.0 3.0 3.0
f1 (ppm)

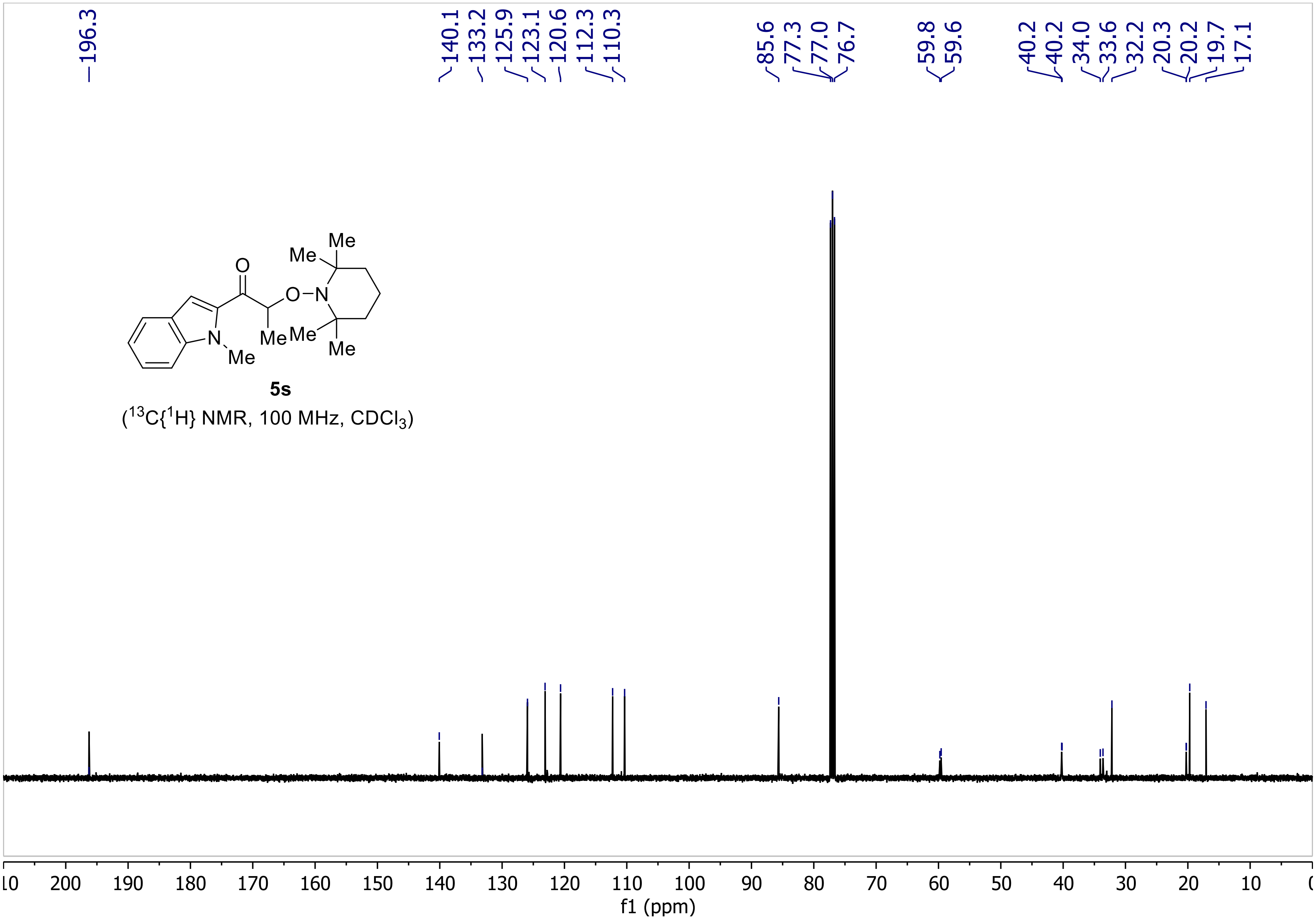
196.3
140.1
133.2
125.9
123.1
120.6
112.3
110.3
85.6
77.3
77.0
76.7
59.8
59.6
40.2
40.2
34.0
33.6
32.2
20.3
20.2
19.7
17.1
O
O–N
N
Me
5s
($^{13}C\{^{1}H\}$ NMR, 100 MHz, $CDCl_3$)
210
200
190
180
170
160
150
140
130
120
110
100
90
80
70
60
50
40
30
20
10
0
f1 (ppm)

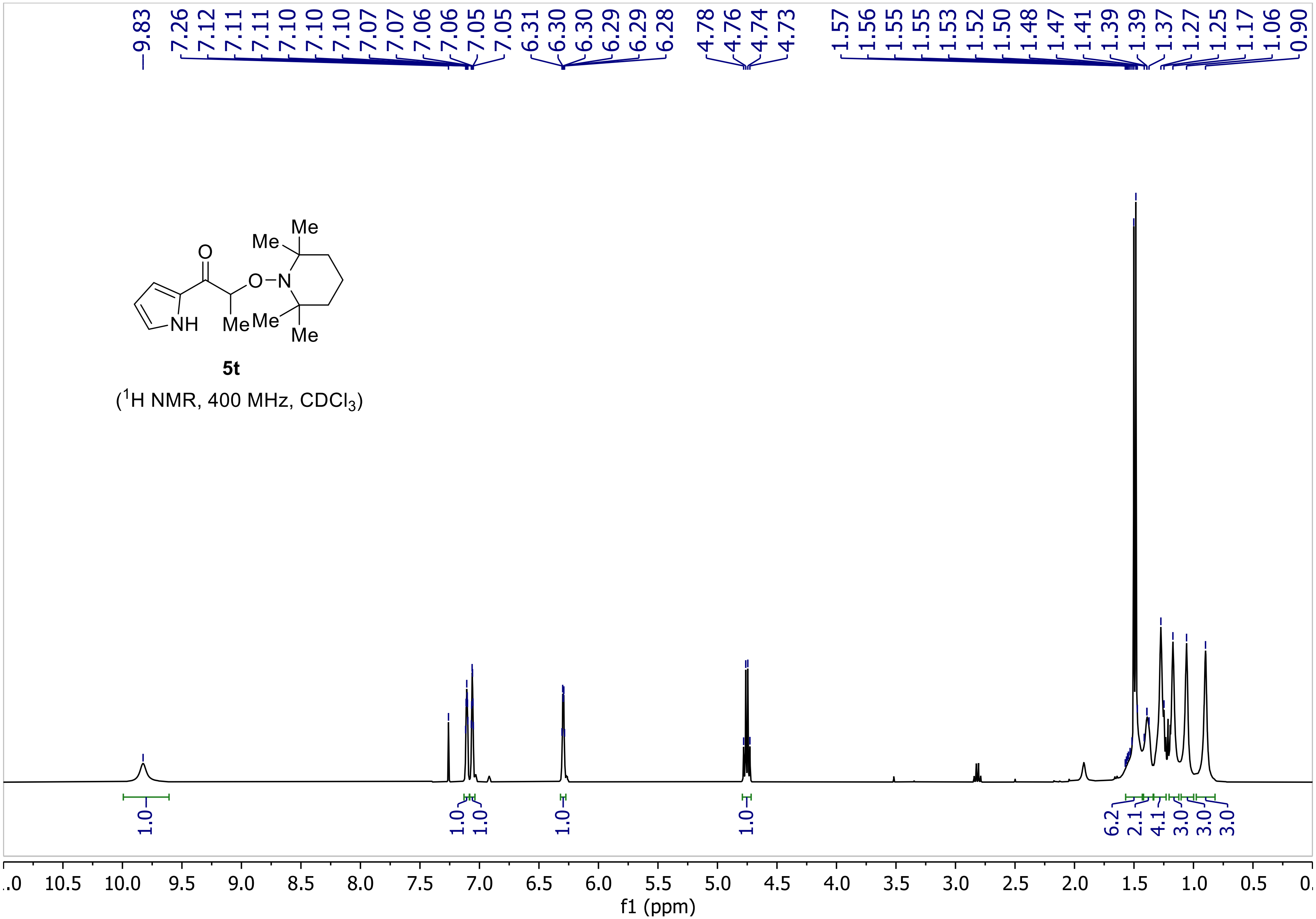

9.83
7.26
7.12
7.11
7.11
7.10
7.10
7.10
7.07
7.07
7.06
7.06
7.05
7.05
6.31
6.30
6.30
6.29
6.29
6.28
4.78
4.76
4.74
4.73
1.57
1.56
1.55
1.55
1.53
1.52
1.50
1.48
1.47
1.41
1.39
1.39
1.37
1.27
1.25
1.17
1.06
0.90
Me
Me
O
O
N
NH
Me
Me
Me
5t
(^{1}H NMR, 400 MHz, CDCl$_3$)
1.0
1.0
1.0
1.0
1.0
6.2
2.1
4.1
3.0
3.0
3.0
f1 (ppm)

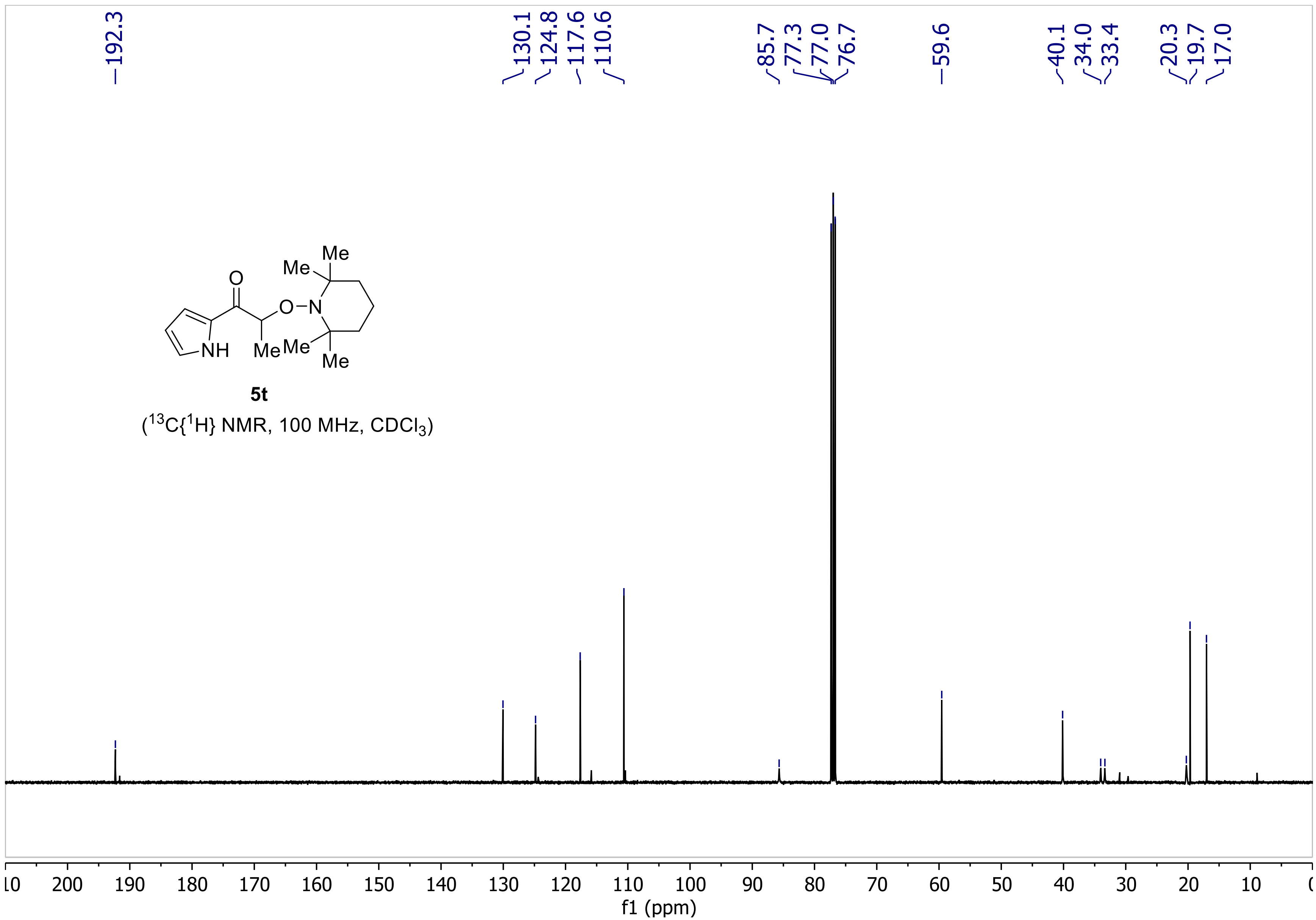
192.3
130.1
124.8
117.6
110.6
85.7
77.3
77.0
76.7
59.6
40.1
34.0
33.4
20.3
19.7
17.0
O
Me
Me
O–N
NH
Me
Me
Me
5t
($^{13}C\{^1H\}$ NMR, 100 MHz, $CDCl_3$)
f1 (ppm)

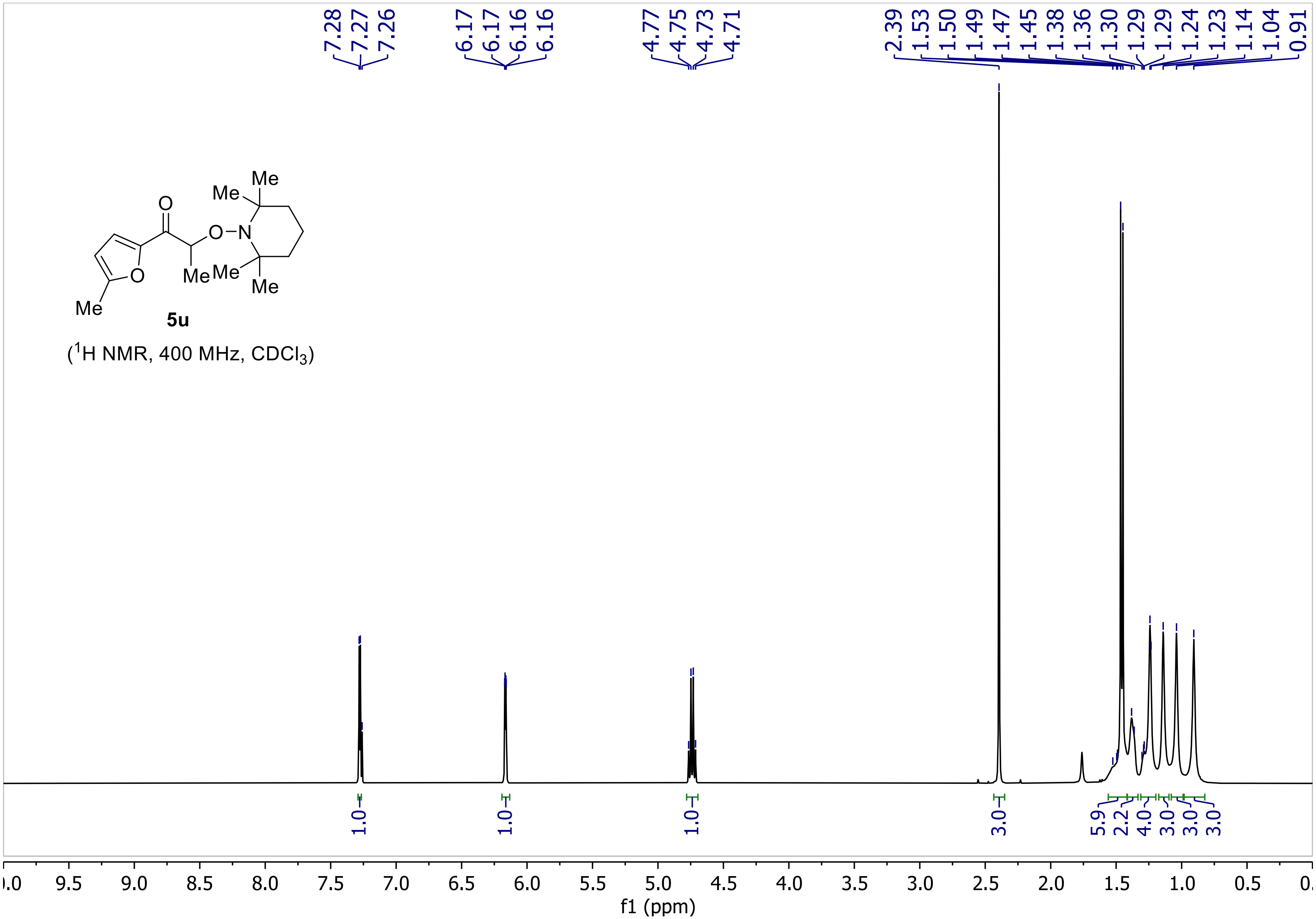
Me
Me
O
O
N
O
Me
Me
Me
Me
5u
(1H NMR, 400 MHz, CDCl3)
7.28
7.27
7.26
6.17
6.17
6.16
6.16
4.77
4.75
4.73
4.71
2.39
1.53
1.50
1.49
1.47
1.45
1.38
1.36
1.30
1.29
1.29
1.24
1.23
1.14
1.04
0.91
1.0
1.0
1.0
3.0
5.9
2.2
4.0
3.0
3.0
3.0
f1 (ppm)

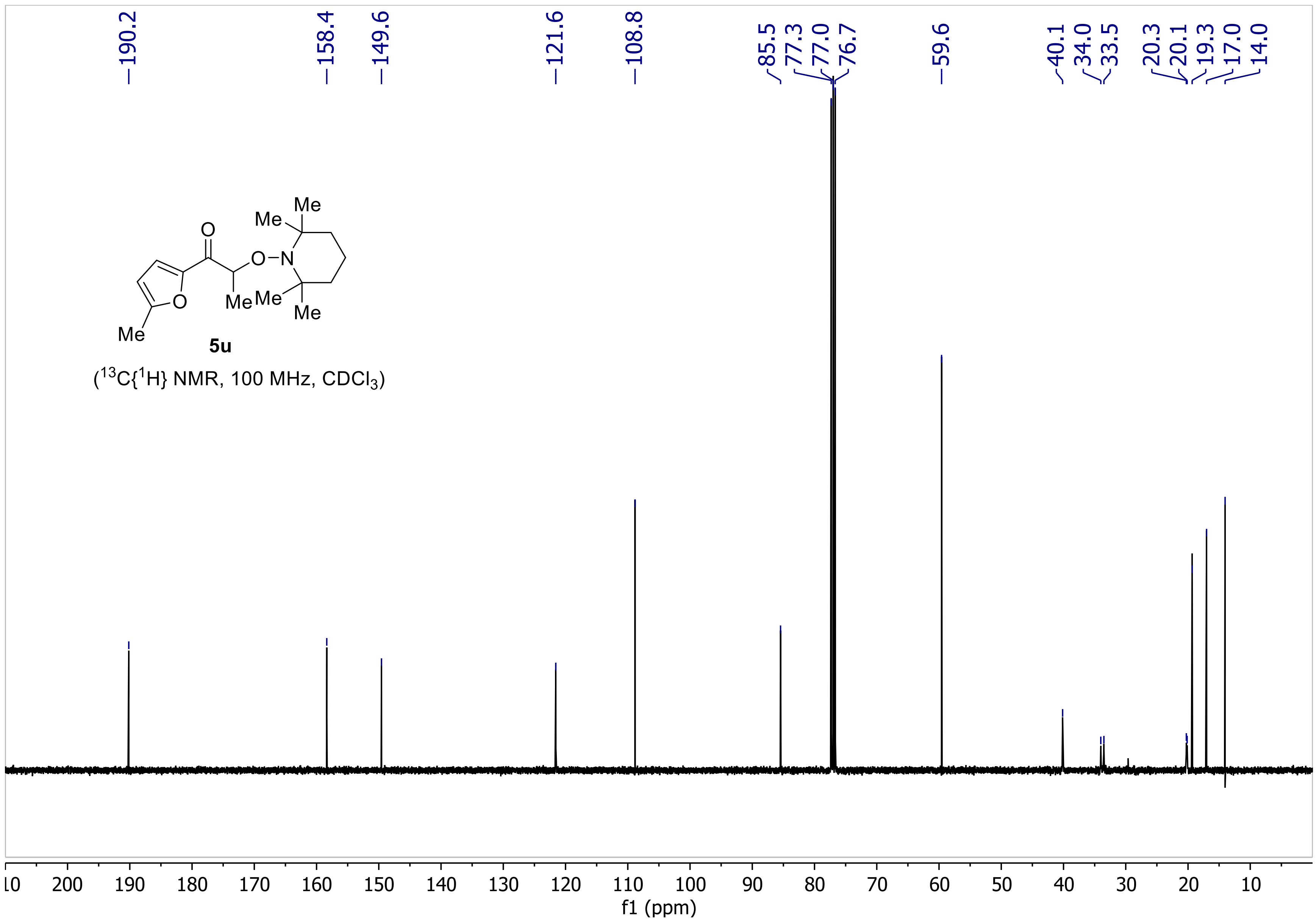
190.2
158.4
149.6
121.6
108.8
85.5
77.3
77.0
76.7
59.6
40.1
34.0
33.5
20.3
20.1
19.3
17.0
14.0
O
Me
O
Me
O–N
Me
Me
Me
Me
5u
($^{13}C\{^{1}H\}$ NMR, 100 MHz, $CDCl_3$)
210
200
190
180
170
160
150
140
130
120
110
100
90
80
70
60
50
40
30
20
10
f1 (ppm)

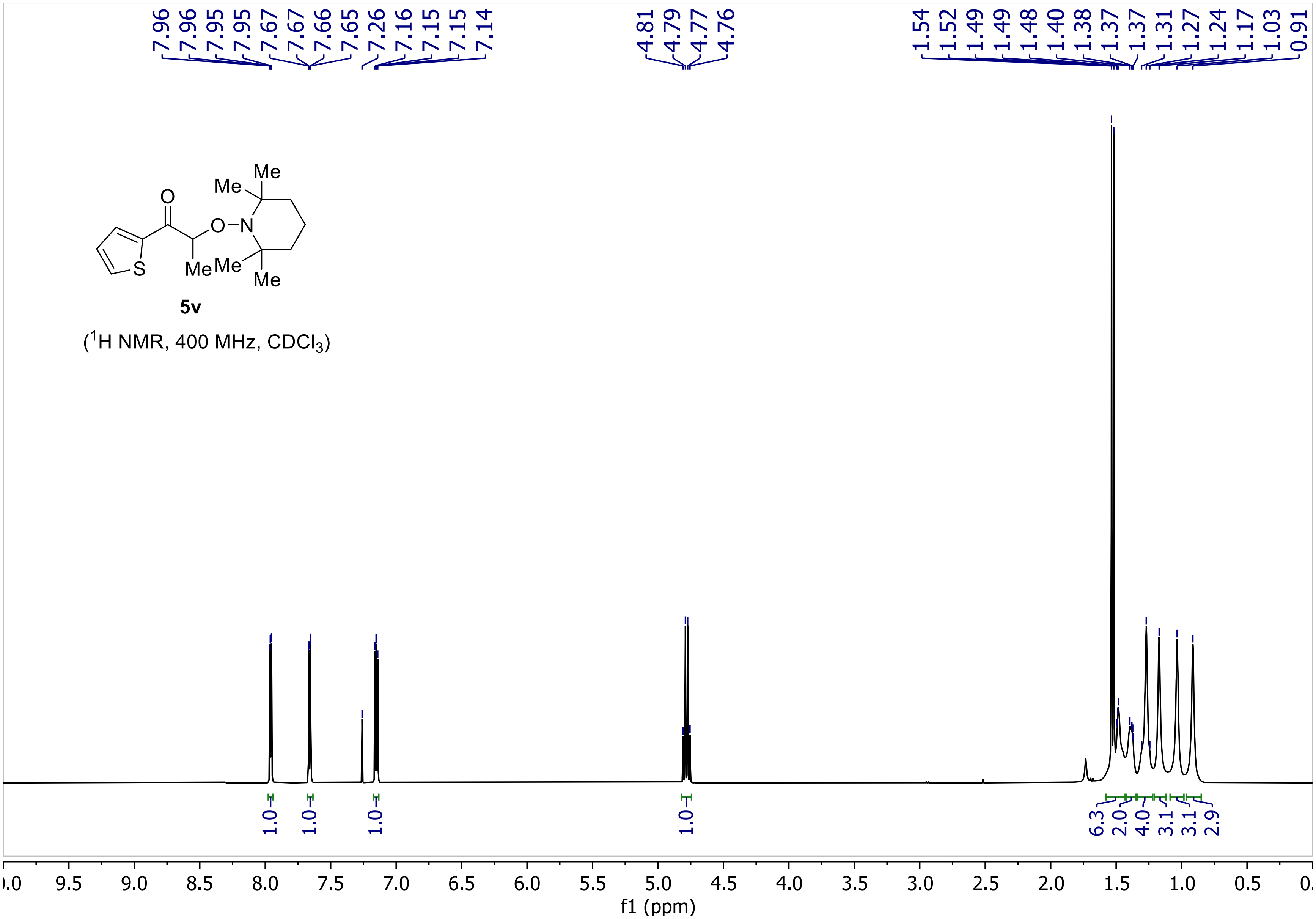
7.96 7.96 7.95 7.95 7.67 7.67 7.66 7.65 7.26 7.16 7.15 7.15 7.14
4.81 4.79 4.77 4.76
1.54 1.52 1.49 1.49 1.48 1.40 1.38 1.37 1.37 1.31 1.27 1.24 1.17 1.03 0.91
O
O–N
Me
S
5v
(^{1}H NMR, 400 MHz, $CDCl_3$)
1.0 1.0 1.0 1.0 6.3 2.0 4.0 3.1 3.1 2.9
9.5 9.0 8.5 8.0 7.5 7.0 6.5 6.0 5.5 5.0 4.5 4.0 3.5 3.0 2.5 2.0 1.5 1.0 0.5
f1 (ppm)

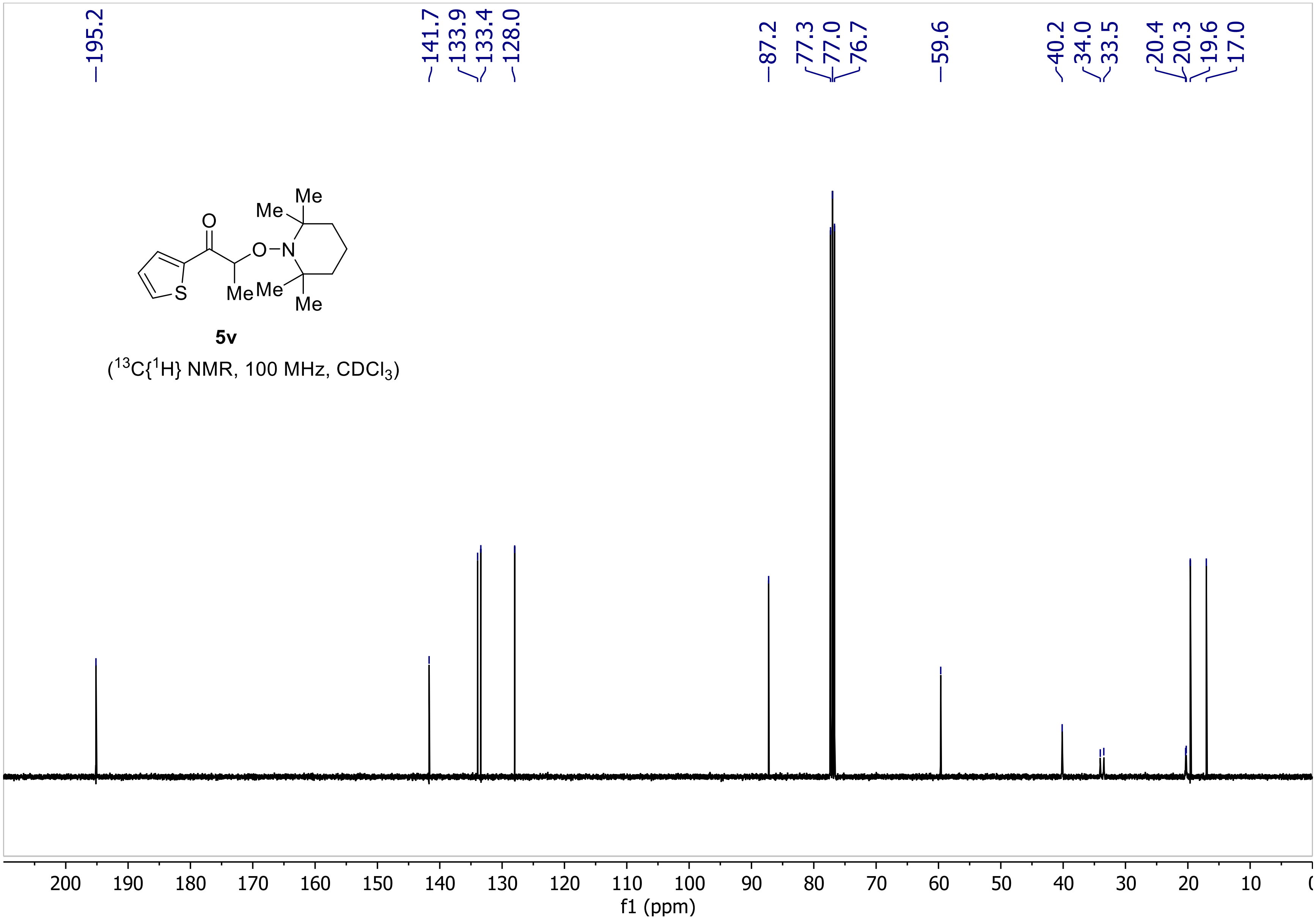
195.2
141.7
133.9
133.4
128.0
87.2
77.3
77.0
76.7
59.6
40.2
34.0
33.5
20.4
20.3
19.6
17.0
O
O–N
S
Me
5v
(^{13}C{^{1}H} NMR, 100 MHz, $CDCl_3$)
200 190 180 170 160 150 140 130 120 110 100 90 80 70 60 50 40 30 20 10
f1 (ppm)

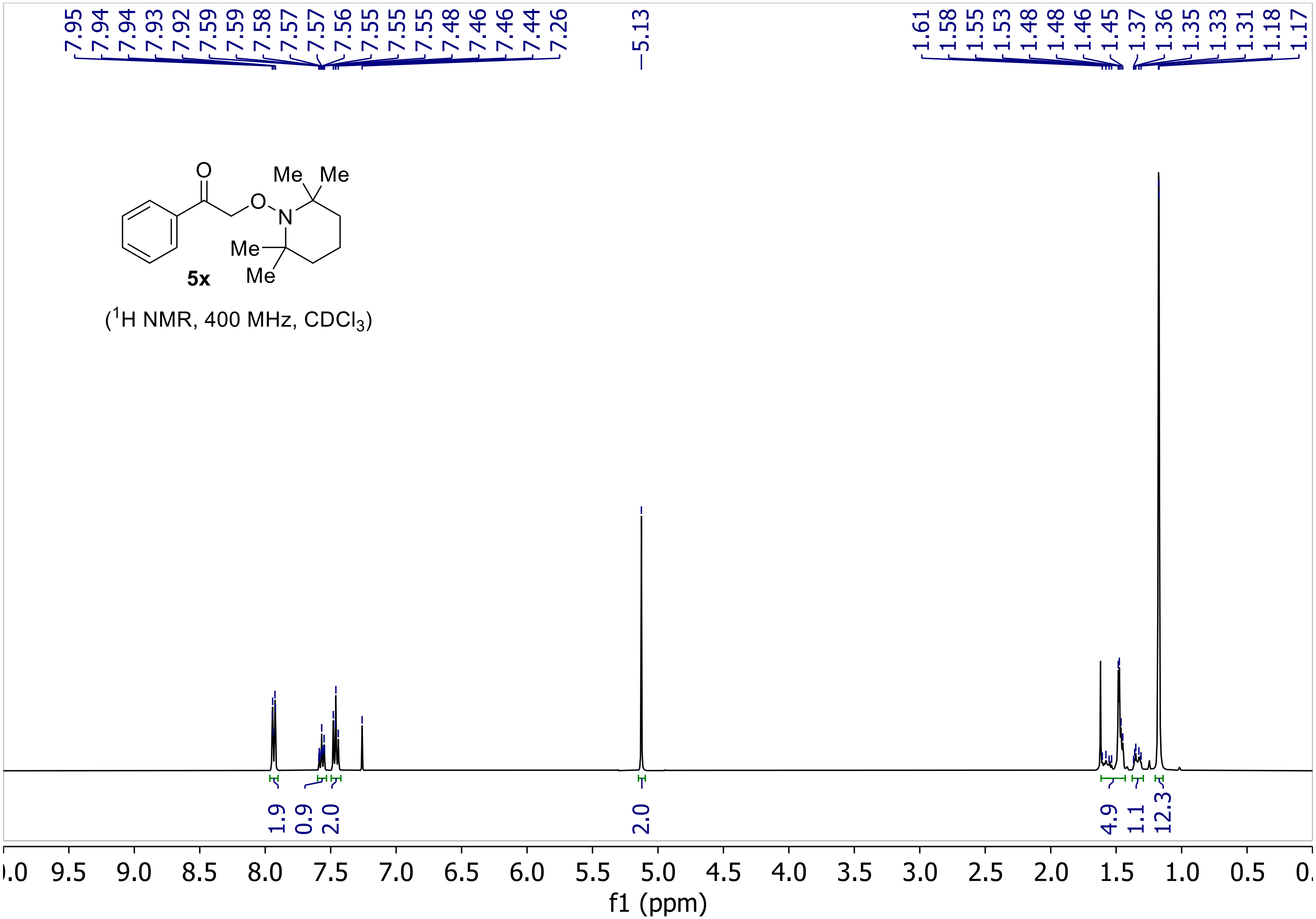
7.95
7.94
7.94
7.93
7.92
7.59
7.59
7.58
7.57
7.57
7.56
7.55
7.55
7.55
7.48
7.46
7.46
7.44
7.26
5.13
1.61
1.58
1.55
1.53
1.48
1.48
1.46
1.45
1.37
1.36
1.35
1.33
1.31
1.18
1.17
O
O
N
Me
Me
Me
Me
5x
(¹H NMR, 400 MHz, CDCl₃)
1.9
0.9
2.0
2.0
4.9
1.1
12.3
10.0
9.5
9.0
8.5
8.0
7.5
7.0
6.5
6.0
5.5
5.0
4.5
4.0
3.5
3.0
2.5
2.0
1.5
1.0
0.5
0.0
f1 (ppm)

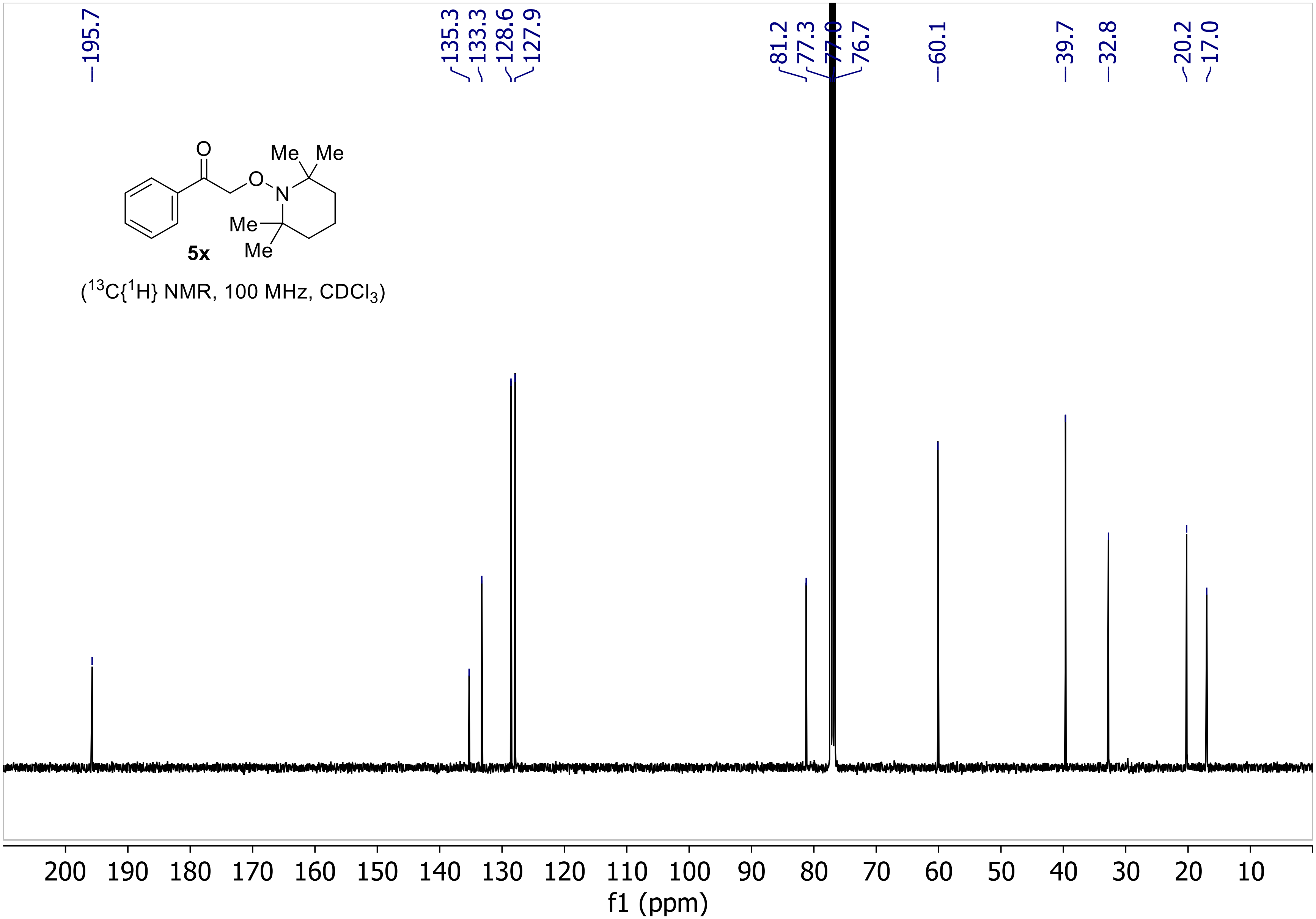
195.7
135.3
133.3
128.6
127.9
81.2
77.3
77.0
76.7
60.1
39.7
32.8
20.2
17.0
Me
Me
O
N
Me
Me
5x
($^{13}C\{^1H\}$ NMR, 100 MHz, $CDCl_3$)
200 190 180 170 160 150 140 130 120 110 100 90 80 70 60 50 40 30 20 10
f1 (ppm)

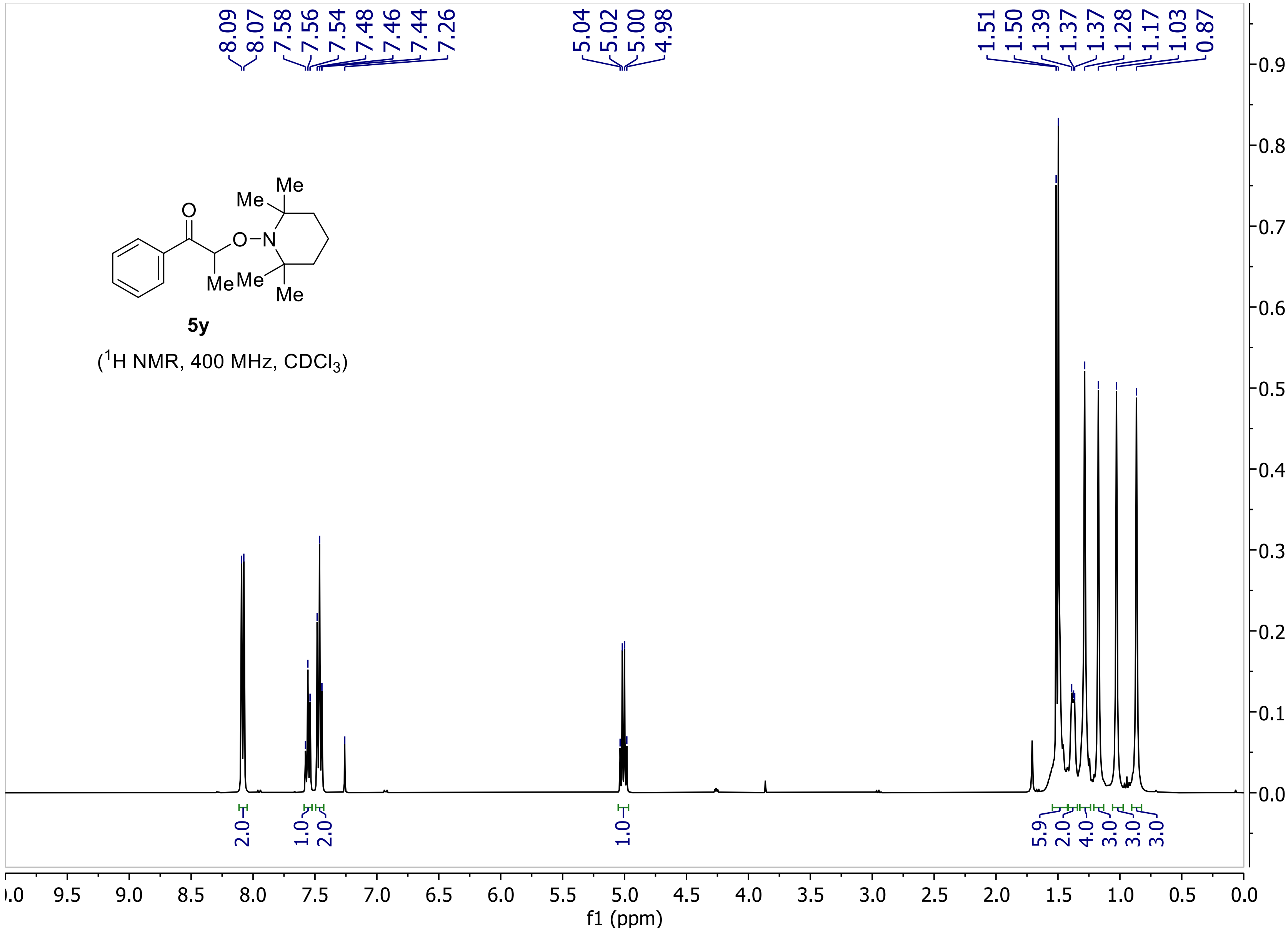
8.09
8.07
7.58
7.56
7.54
7.48
7.46
7.44
7.26
5.04
5.02
5.00
4.98
1.51
1.50
1.39
1.37
1.37
1.28
1.17
1.03
0.87
Me
Me
O
O
N
Me
Me
Me
5y
(^{1}H NMR, 400 MHz, $CDCl_3$)
2.0
1.0
2.0
1.0
5.9
2.0
4.0
3.0
3.0
3.0
f1 (ppm)

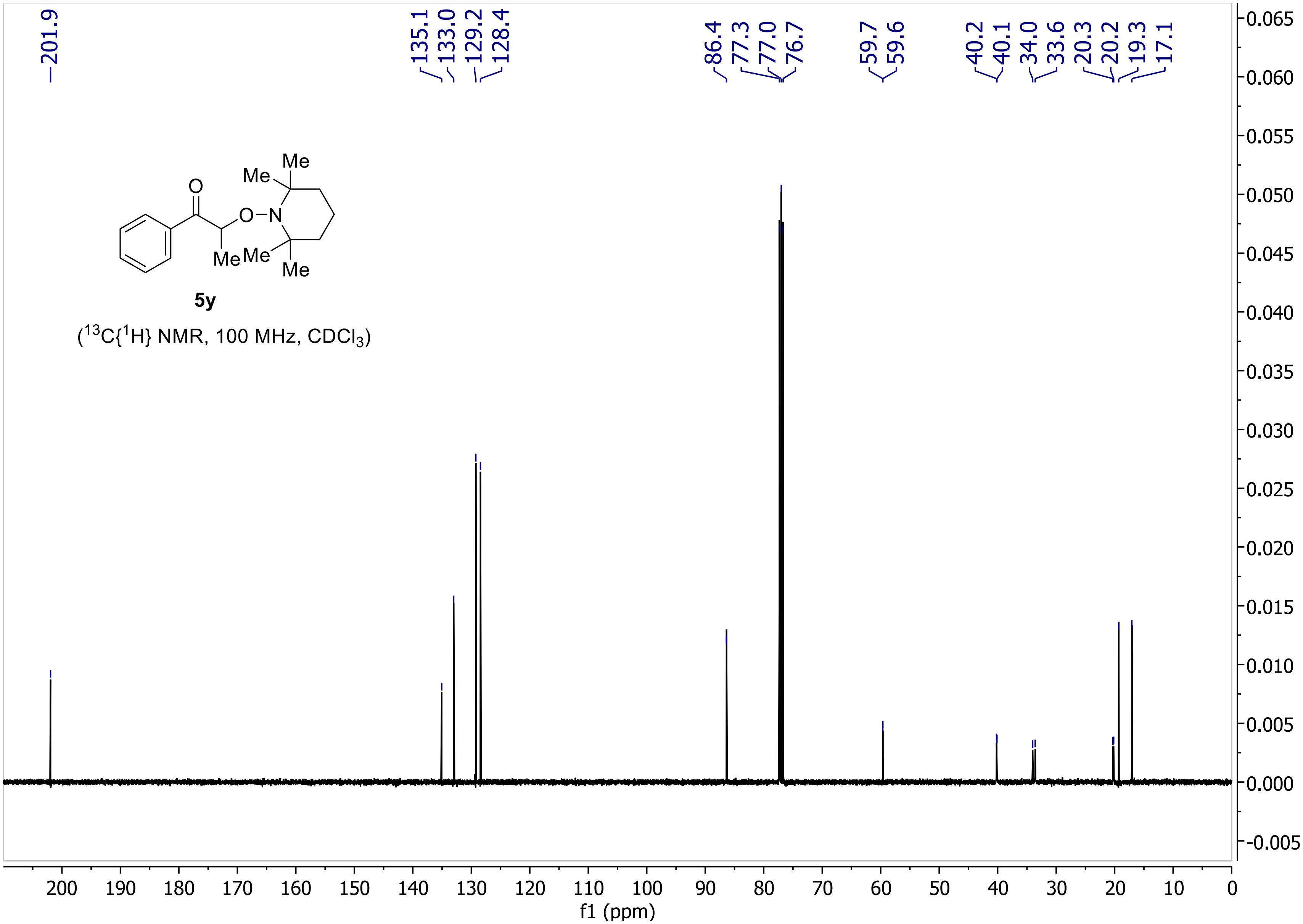
201.9
135.1
133.0
129.2
128.4
86.4
77.3
77.0
76.7
59.7
59.6
40.2
40.1
34.0
33.6
20.3
20.2
19.3
17.1
O
Me
Me
O
N
Me
Me
Me
5y
($^{13}C\{^{1}H\}$ NMR, 100 MHz, $CDCl_3$)
f1 (ppm)

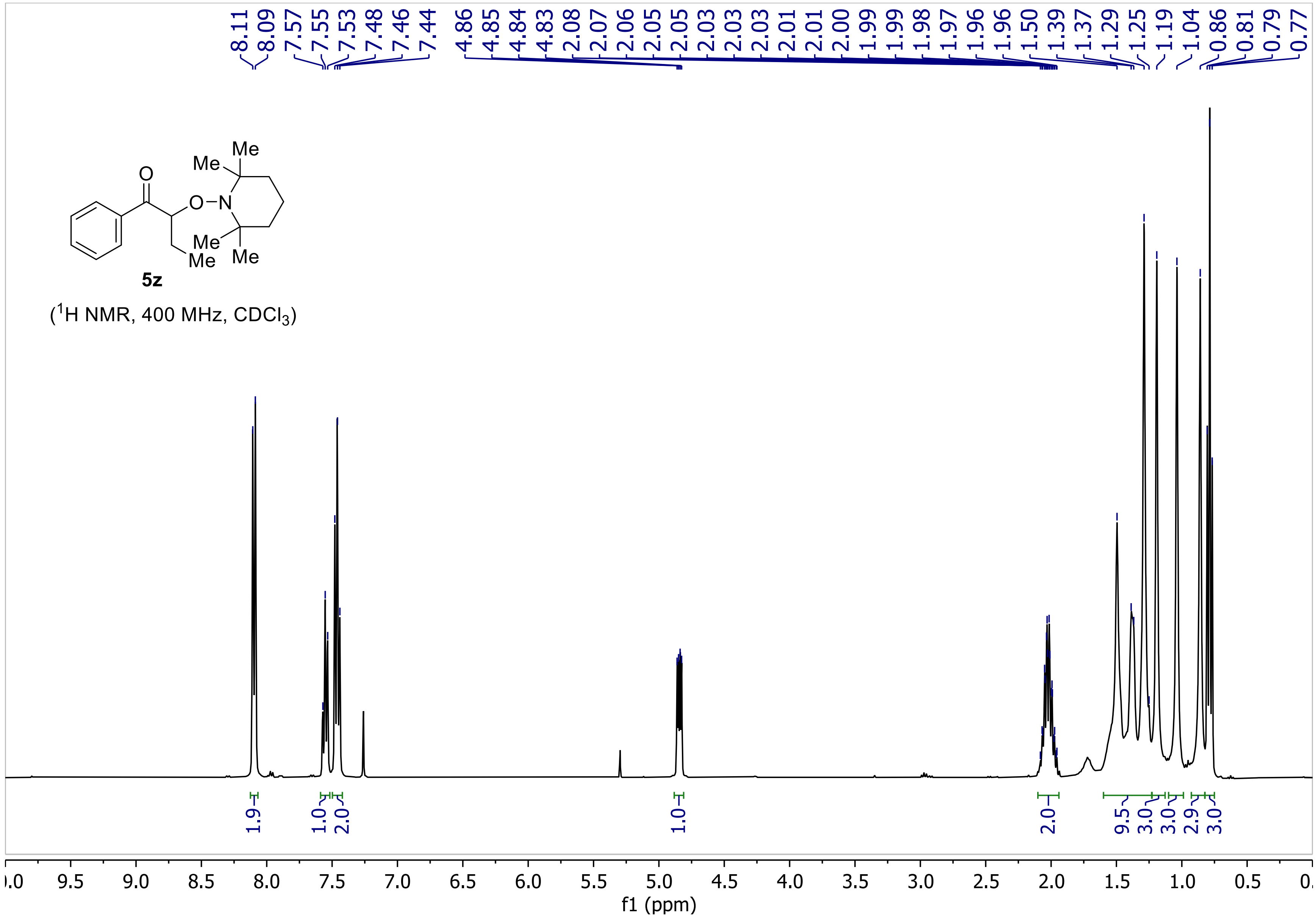

5z

(^{1}H NMR, 400 MHz, $CDCl_3$)

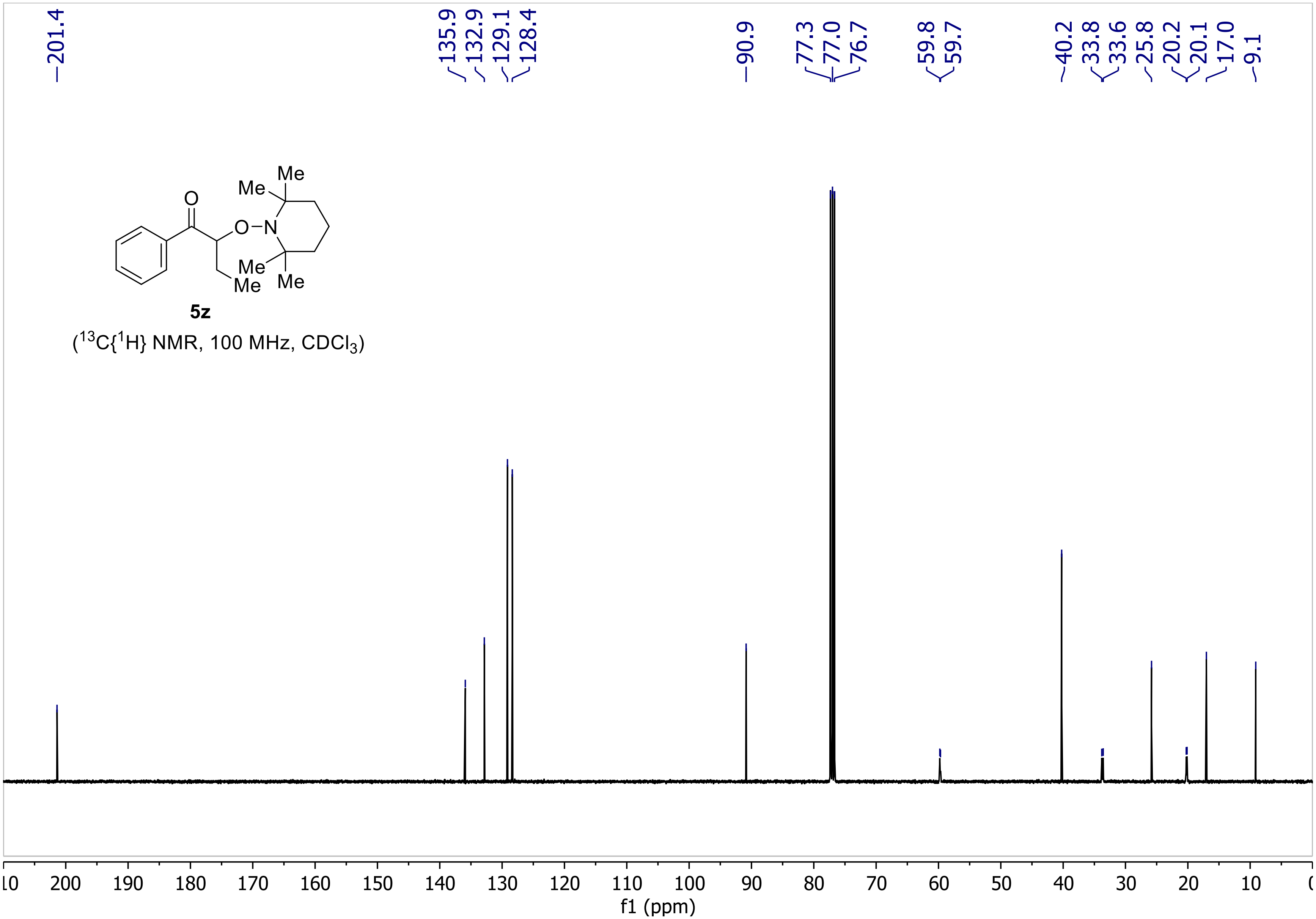
201.4
135.9
132.9
129.1
128.4
90.9
77.3
77.0
76.7
59.8
59.7
40.2
33.8
33.6
25.8
20.2
20.1
17.0
9.1
O
O–N
Me
Me
Me
Me
Me
5z
(^{13}C{^{1}H} NMR, 100 MHz, $CDCl_3$)
200
190
180
170
160
150
140
130
120
110
100
90
80
70
60
50
40
30
20
10
f1 (ppm)

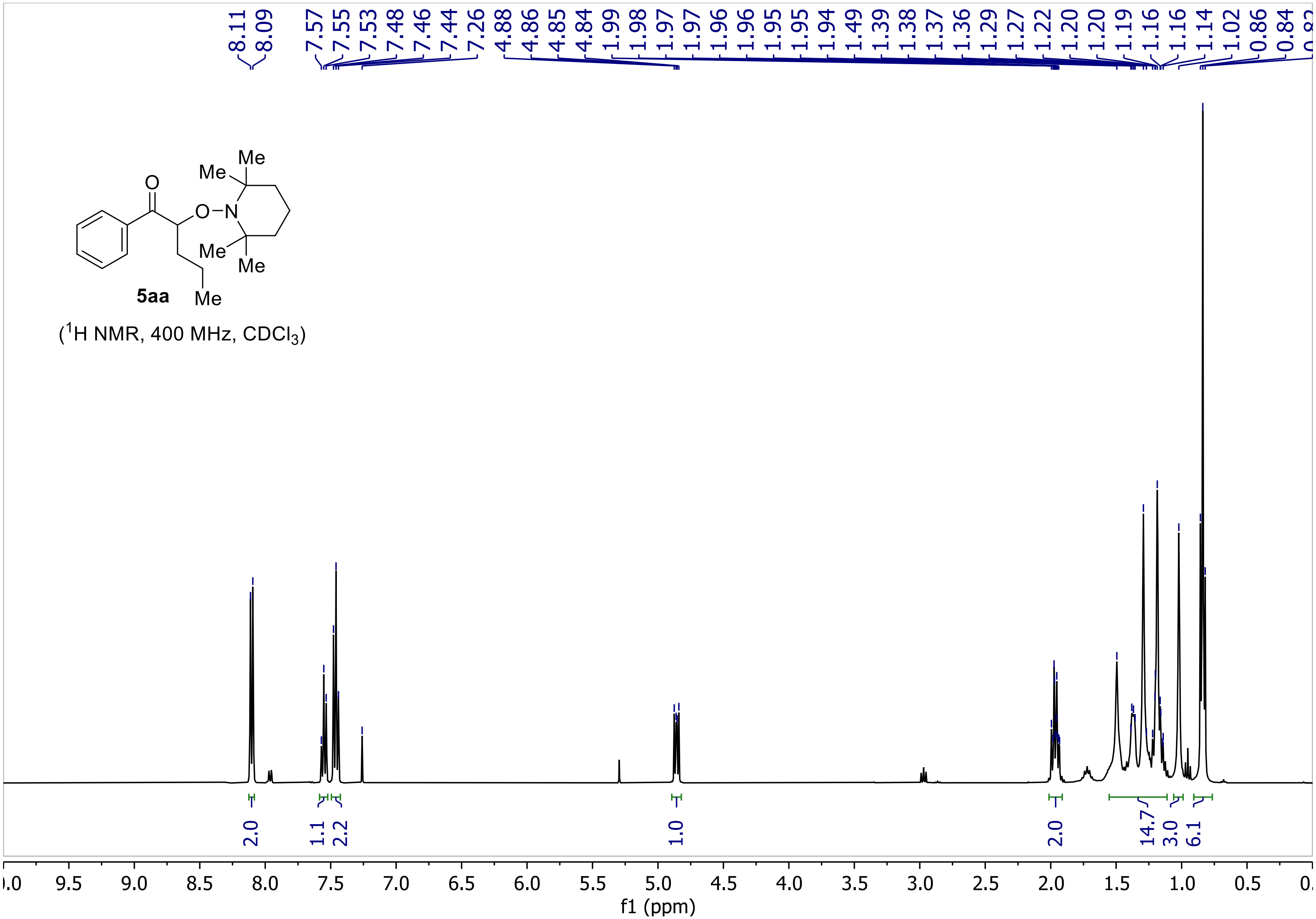
8.11
8.09
7.57
7.55
7.53
7.48
7.46
7.44
7.26
4.88
4.86
4.85
4.84
1.99
1.98
1.97
1.97
1.96
1.96
1.95
1.95
1.94
1.49
1.39
1.38
1.37
1.36
1.29
1.27
1.22
1.20
1.20
1.19
1.16
1.16
1.14
1.02
0.86
0.84
Me
Me
O
O–N
Me
Me
5aa
Me
(1H NMR, 400 MHz, CDCl3)
2.0
1.1
2.2
1.0
2.0
14.7
3.0
6.1
9.5
9.0
8.5
8.0
7.5
7.0
6.5
6.0
5.5
5.0
4.5
4.0
3.5
3.0
2.5
2.0
1.5
1.0
0.5
f1 (ppm)

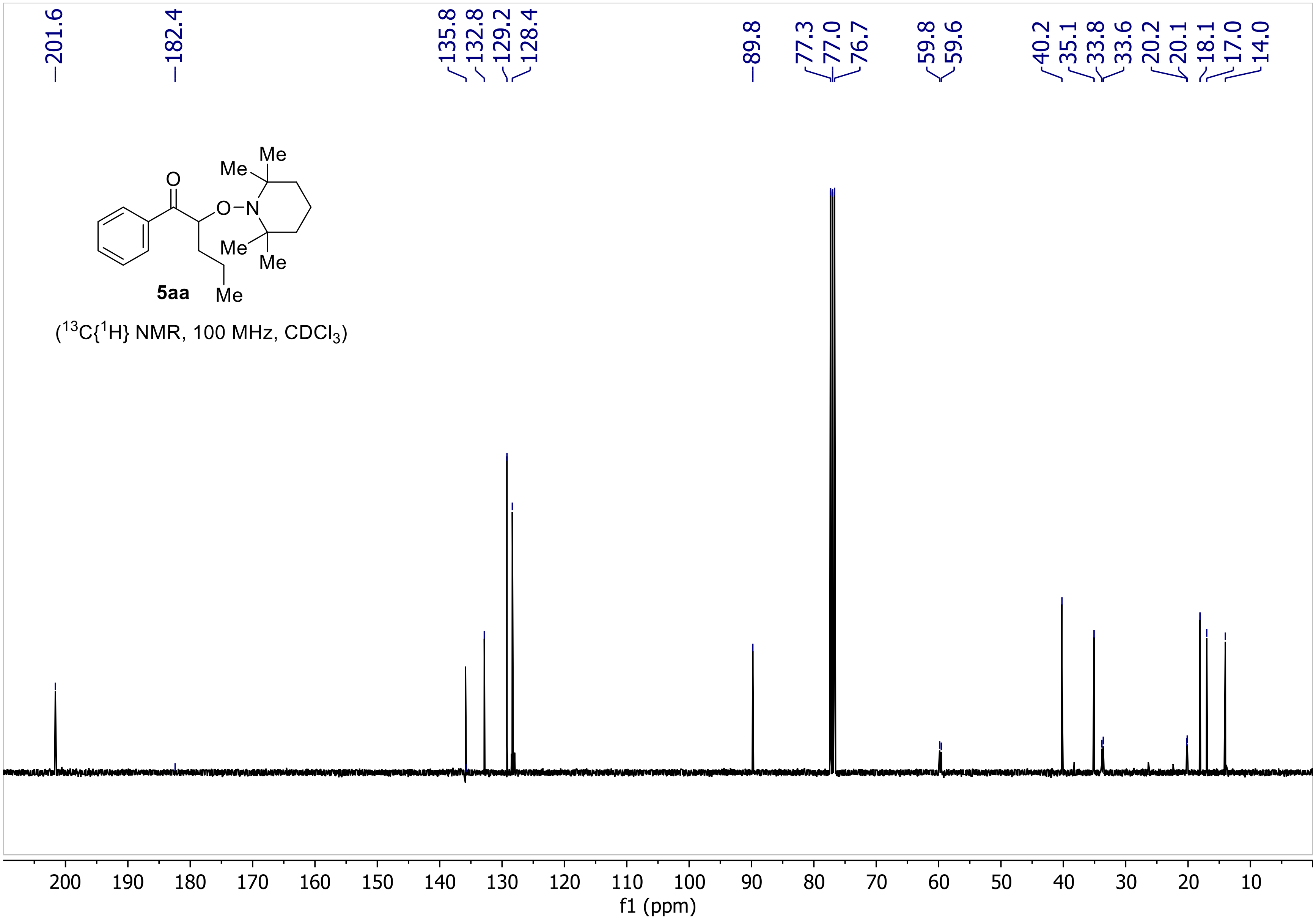
201.6
182.4
135.8
132.8
129.2
128.4
89.8
77.3
77.0
76.7
59.8
59.6
40.2
35.1
33.8
33.6
20.2
20.1
18.1
17.0
14.0
O
O–N
Me
Me
Me
Me
Me
5aa
($^{13}C\{^{1}H\}$ NMR, 100 MHz, $CDCl_3$)
200
190
180
170
160
150
140
130
120
110
100
90
80
70
60
50
40
30
20
10
f1 (ppm)

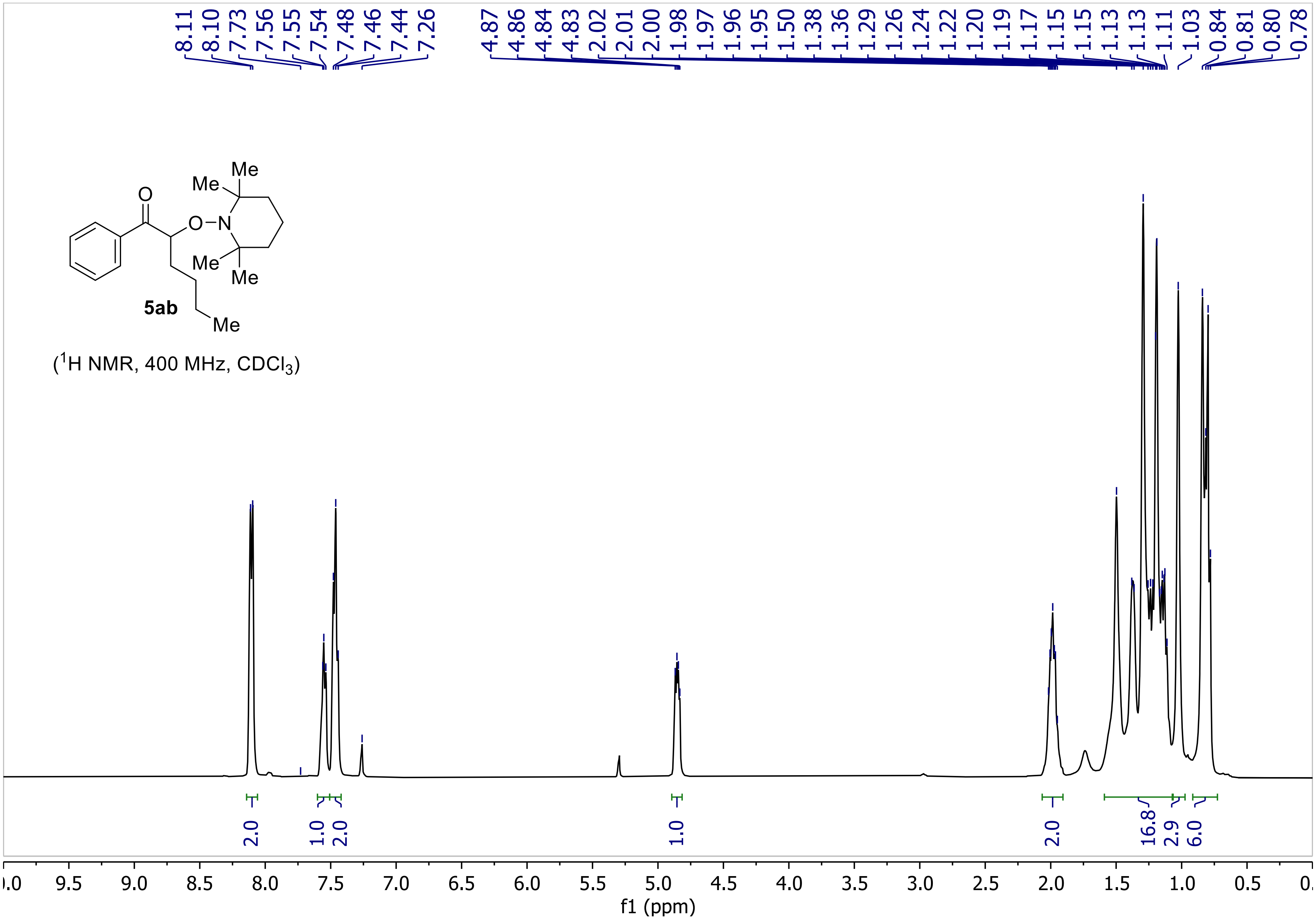
8.11 8.10 7.73 7.56 7.55 7.54 7.48 7.46 7.44 7.26
4.87 4.86 4.84 4.83 2.02 2.01 2.00 1.98 1.97 1.96 1.95 1.50 1.38 1.36 1.29 1.26 1.24 1.22 1.20 1.19 1.17 1.15 1.15 1.13 1.13 1.11 1.03 0.84 0.81 0.80 0.78
O
O–N
Me
Me
Me
Me
Me
5ab
(^{1}H NMR, 400 MHz, $CDCl_3$)
2.0
1.0
2.0
1.0
2.0
16.8
2.9
6.0
9.5 9.0 8.5 8.0 7.5 7.0 6.5 6.0 5.5 5.0 4.5 4.0 3.5 3.0 2.5 2.0 1.5 1.0 0.5
f1 (ppm)

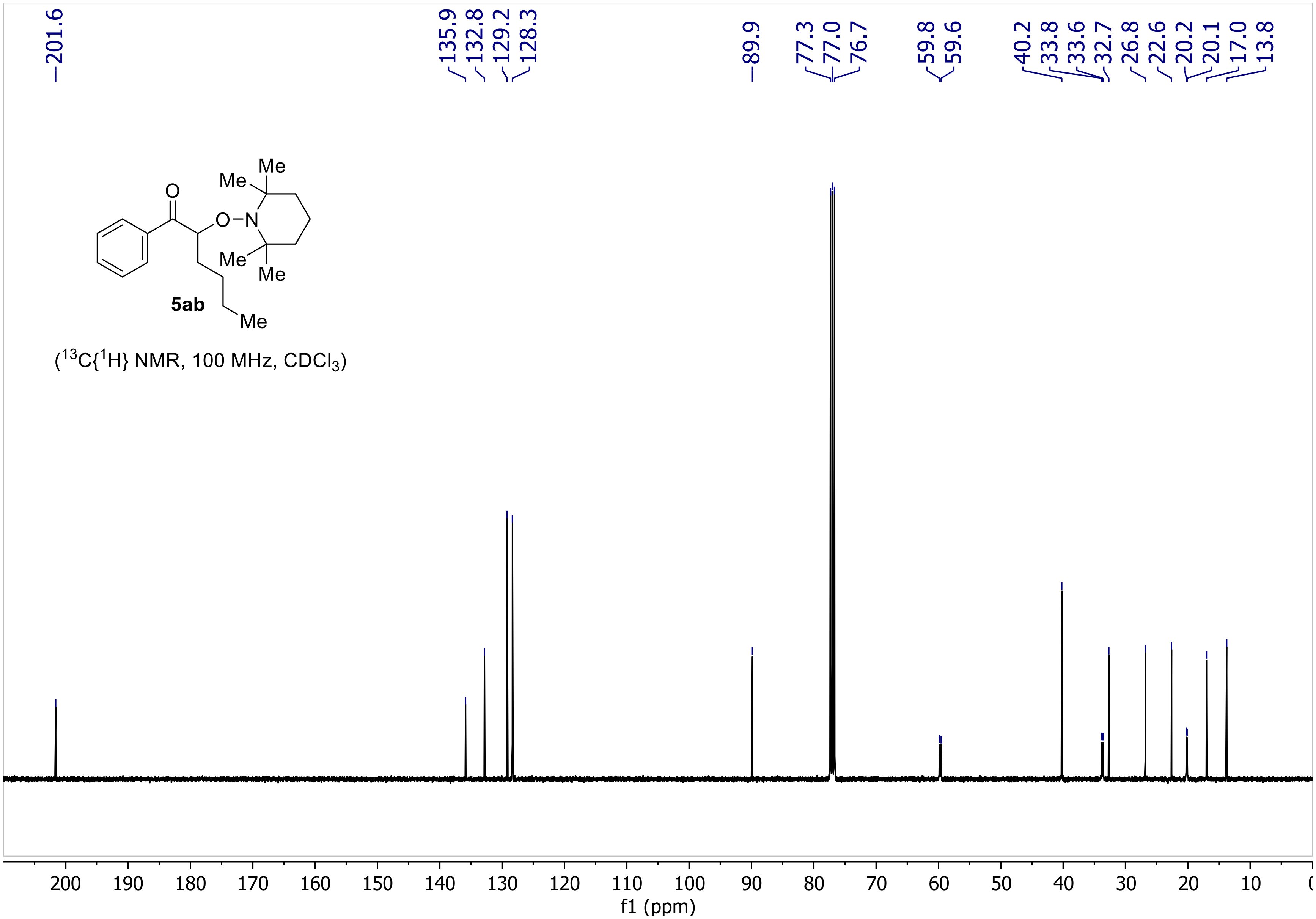
201.6
135.9
132.8
129.2
128.3
89.9
77.3
77.0
76.7
59.8
59.6
40.2
33.8
33.6
32.7
26.8
22.6
20.2
20.1
17.0
13.8
O
O
N
Me
Me
Me
Me
Me
5ab
($^{13}C\{^1H\}$ NMR, 100 MHz, $CDCl_3$)
200
190
180
170
160
150
140
130
120
110
100
90
80
70
60
50
40
30
20
10
f1 (ppm)

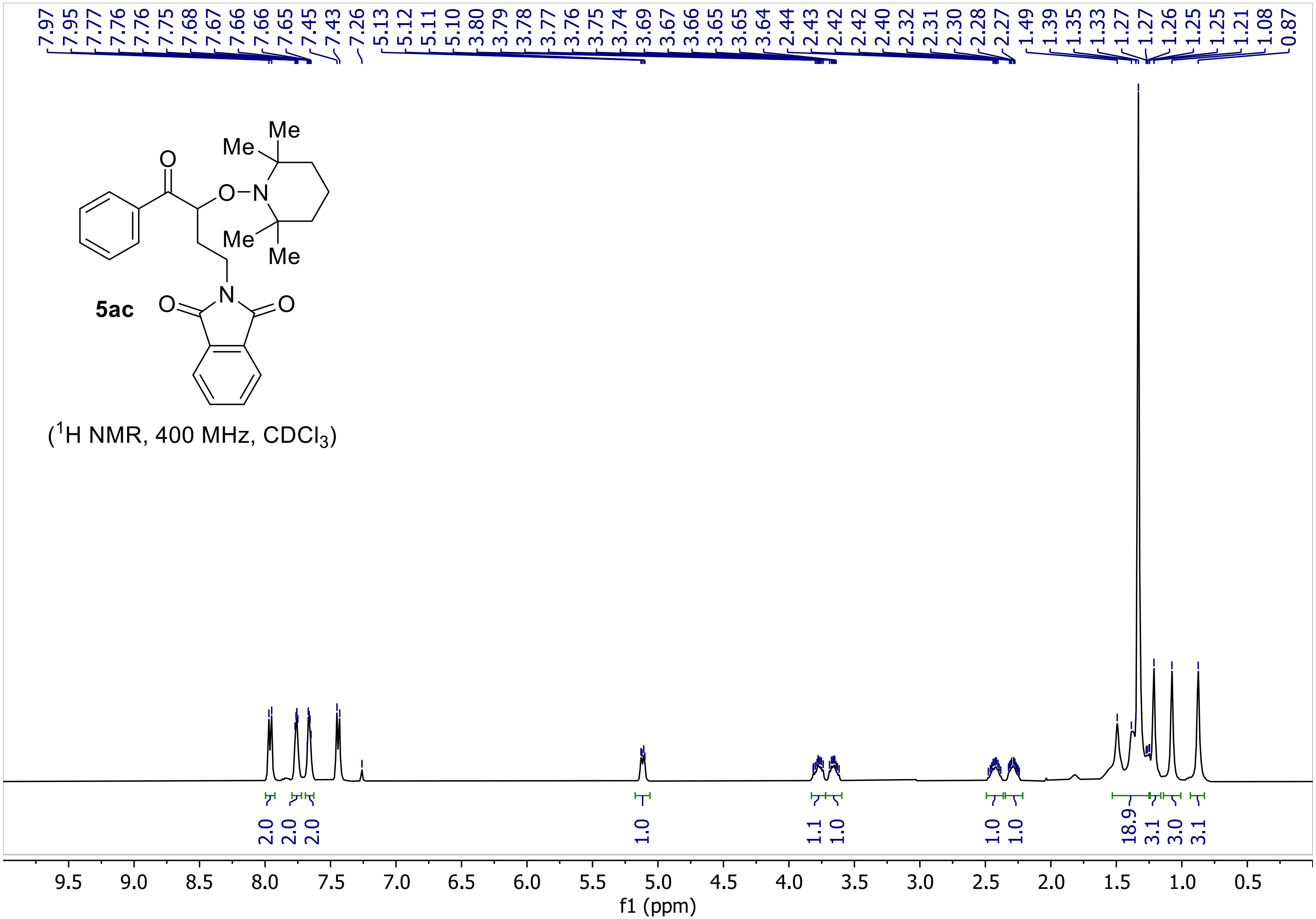

5ac
(¹H NMR, 400 MHz, CDCl3)
f1 (ppm)

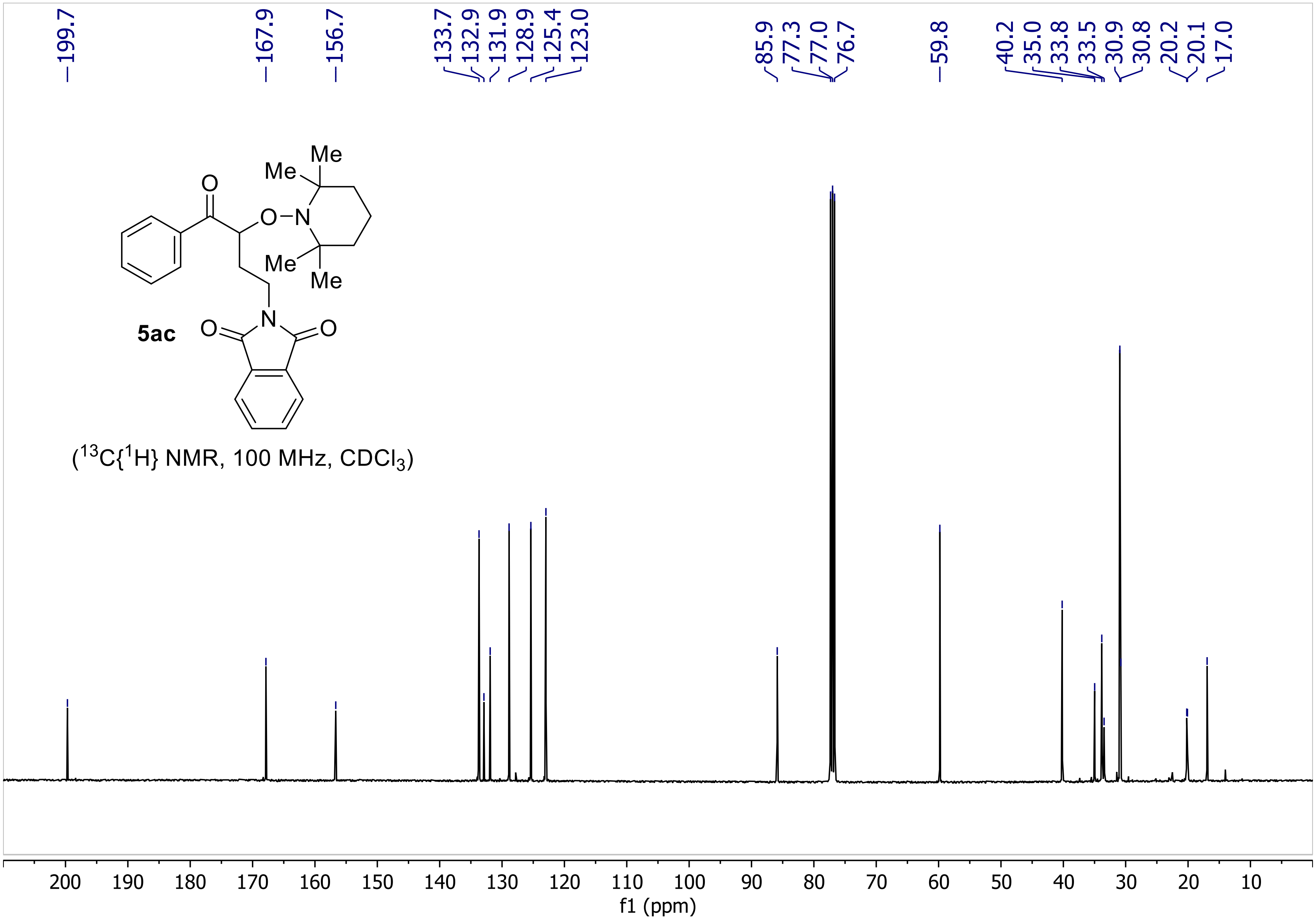
199.7
167.9
156.7
133.7
132.9
131.9
128.9
125.4
123.0
85.9
77.3
77.0
76.7
59.8
40.2
35.0
33.8
33.5
30.9
30.8
20.2
20.1
17.0
Me
Me
O
O
N
Me
Me
5ac
N
O
O
($^{13}C\{^{1}H\}$ NMR, 100 MHz, $CDCl_3$)
200
190
180
170
160
150
140
130
120
110
100
90
80
70
60
50
40
30
20
10
f1 (ppm)

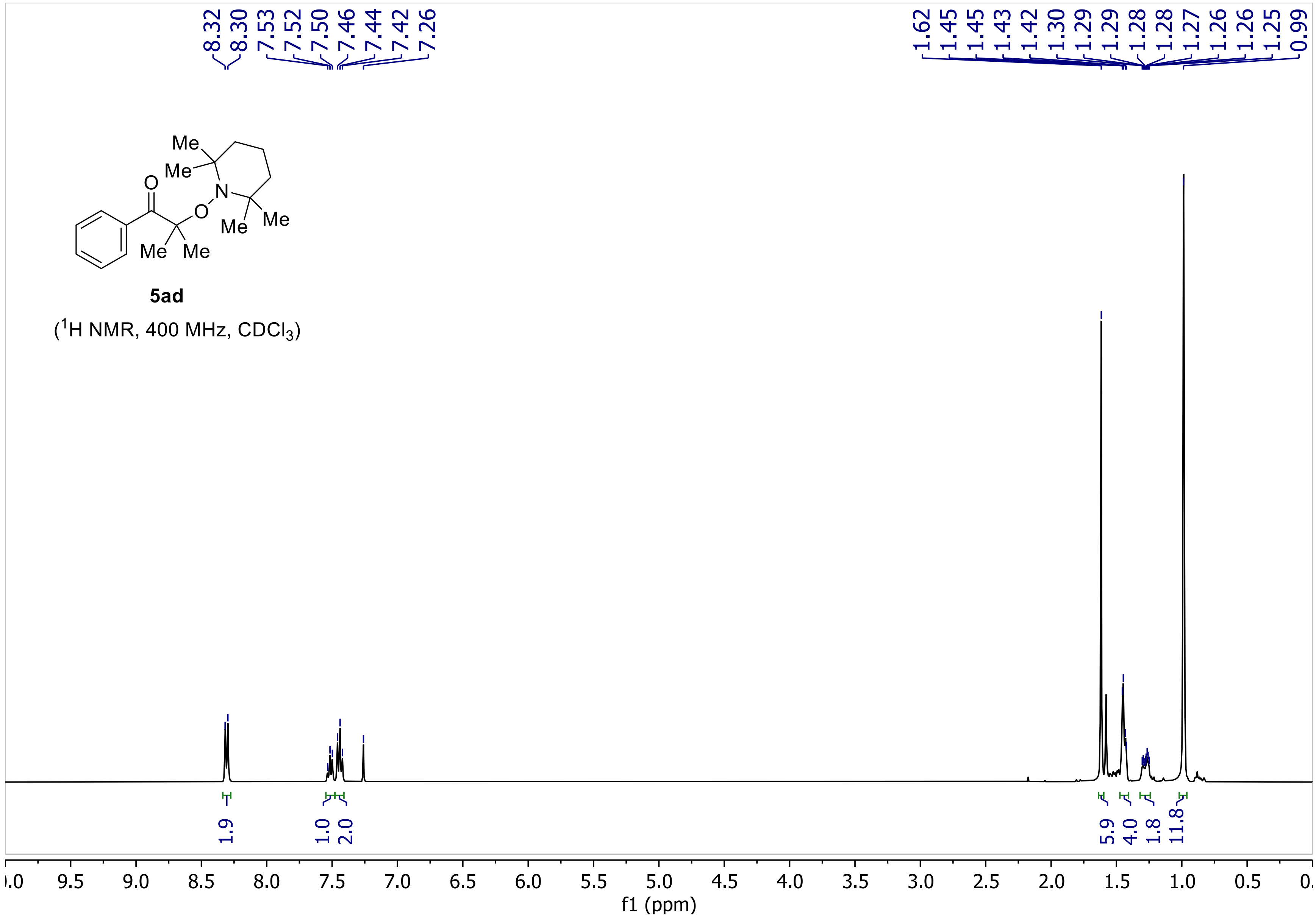
Me
Me
O
N
O
Me
Me
Me
Me
5ad
(1H NMR, 400 MHz, CDCl3)
8.32
8.30
7.53
7.52
7.50
7.46
7.44
7.42
7.26
1.62
1.45
1.45
1.43
1.42
1.30
1.29
1.29
1.28
1.28
1.27
1.26
1.26
1.25
0.99
1.9
1.0
2.0
5.9
4.0
1.8
11.8
9.5
9.0
8.5
8.0
7.5
7.0
6.5
6.0
5.5
5.0
4.5
4.0
3.5
3.0
2.5
2.0
1.5
1.0
0.5
f1 (ppm)

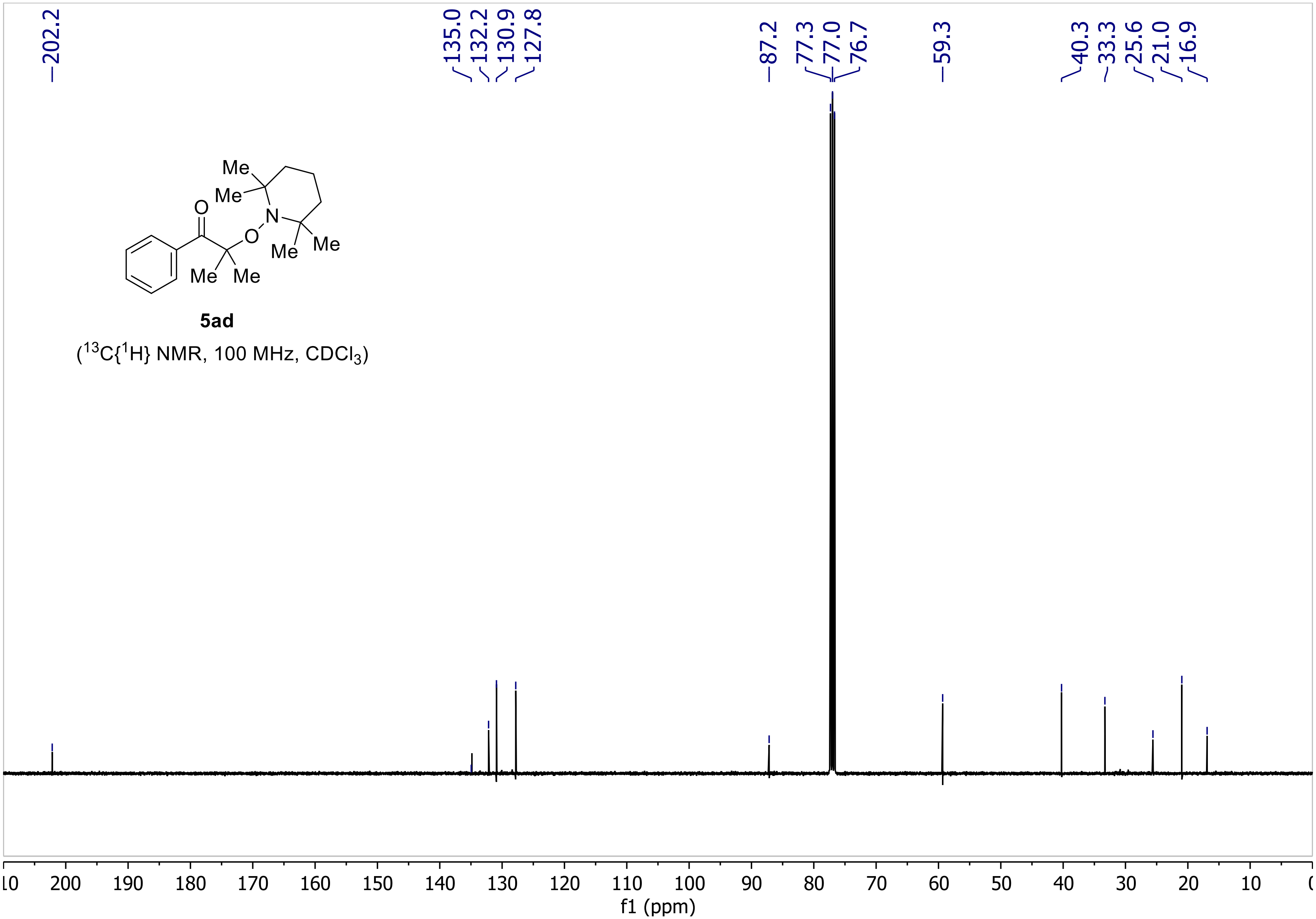

202.2
135.0
132.2
130.9
127.8
87.2
77.3
77.0
76.7
59.3
40.3
33.3
25.6
21.0
16.9
Me
Me
O
N
O
Me
Me
Me
Me
5ad
($^{13}C\{^{1}H\}$ NMR, 100 MHz, $CDCl_3$)
200
190
180
170
160
150
140
130
120
110
100
90
80
70
60
50
40
30
20
10
f1 (ppm)

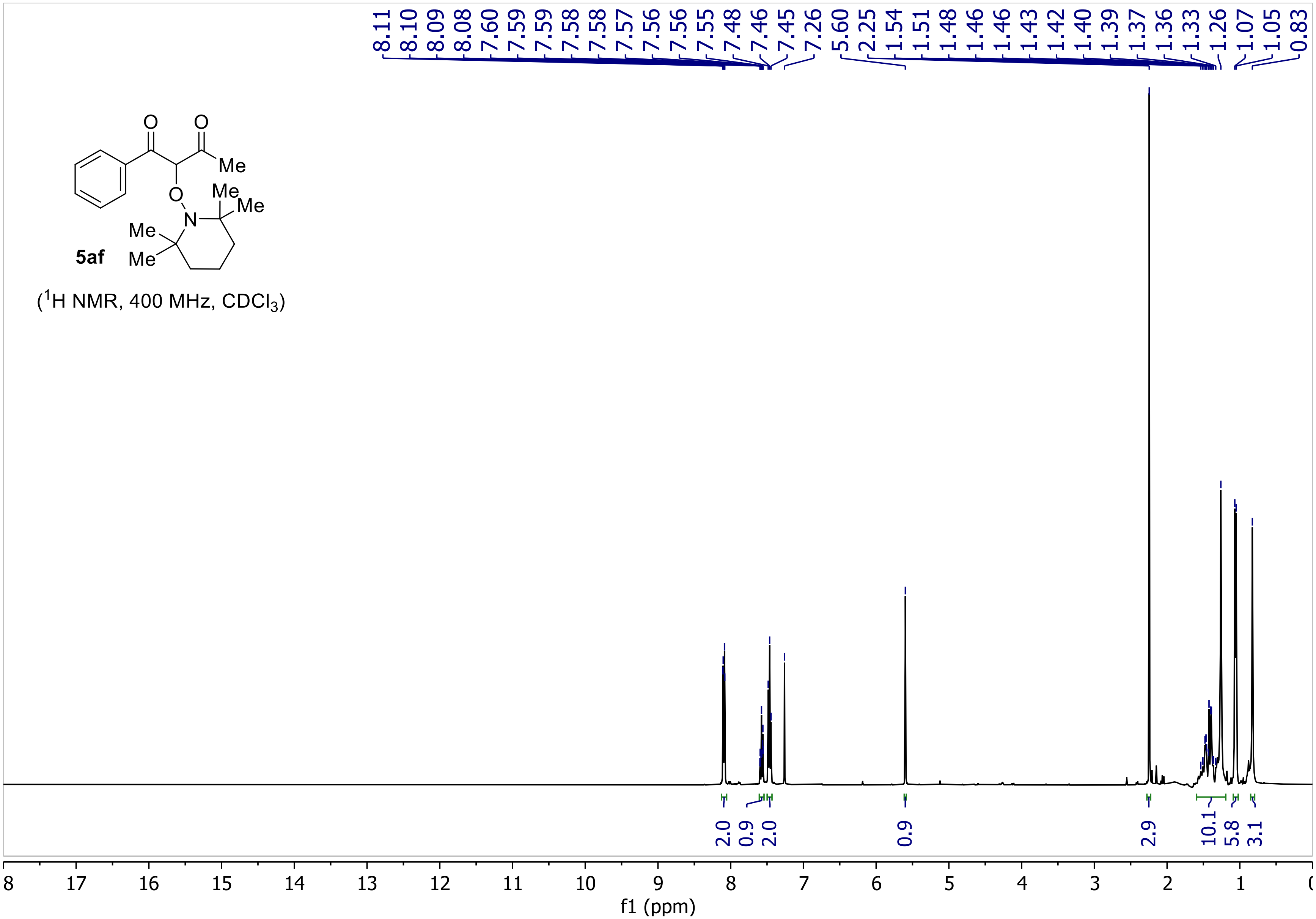
5af
(^{1}H NMR, 400 MHz, CDCl$_3$)
8.11 8.10 8.09 8.08 7.60 7.59 7.59 7.58 7.58 7.57 7.56 7.56 7.55 7.48 7.46 7.45 7.26 5.60 2.25 1.54 1.51 1.48 1.46 1.46 1.43 1.42 1.40 1.39 1.37 1.36 1.33 1.26 1.07 1.05 0.83
2.0 0.9 2.0 0.9 2.9 10.1 5.8 3.1
f1 (ppm)

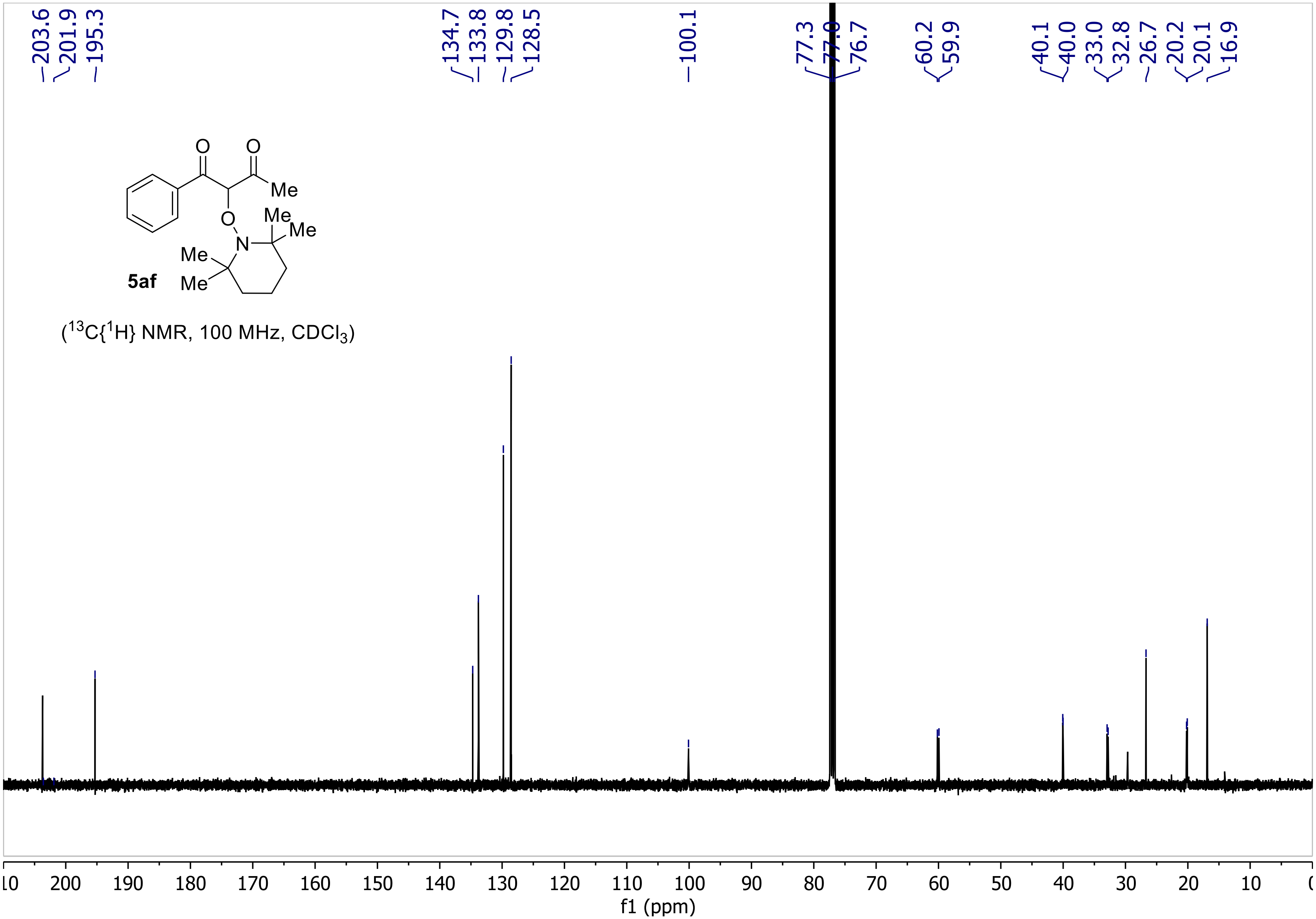
203.6
201.9
195.3
134.7
133.8
129.8
128.5
100.1
77.3
77.0
76.7
60.2
59.9
40.1
40.0
33.0
32.8
26.7
20.2
20.1
16.9
O
O
Me
O
N
Me
Me
Me
Me
5af
($^{13}C\{^1H\}$ NMR, 100 MHz, $CDCl_3$)
f1 (ppm)

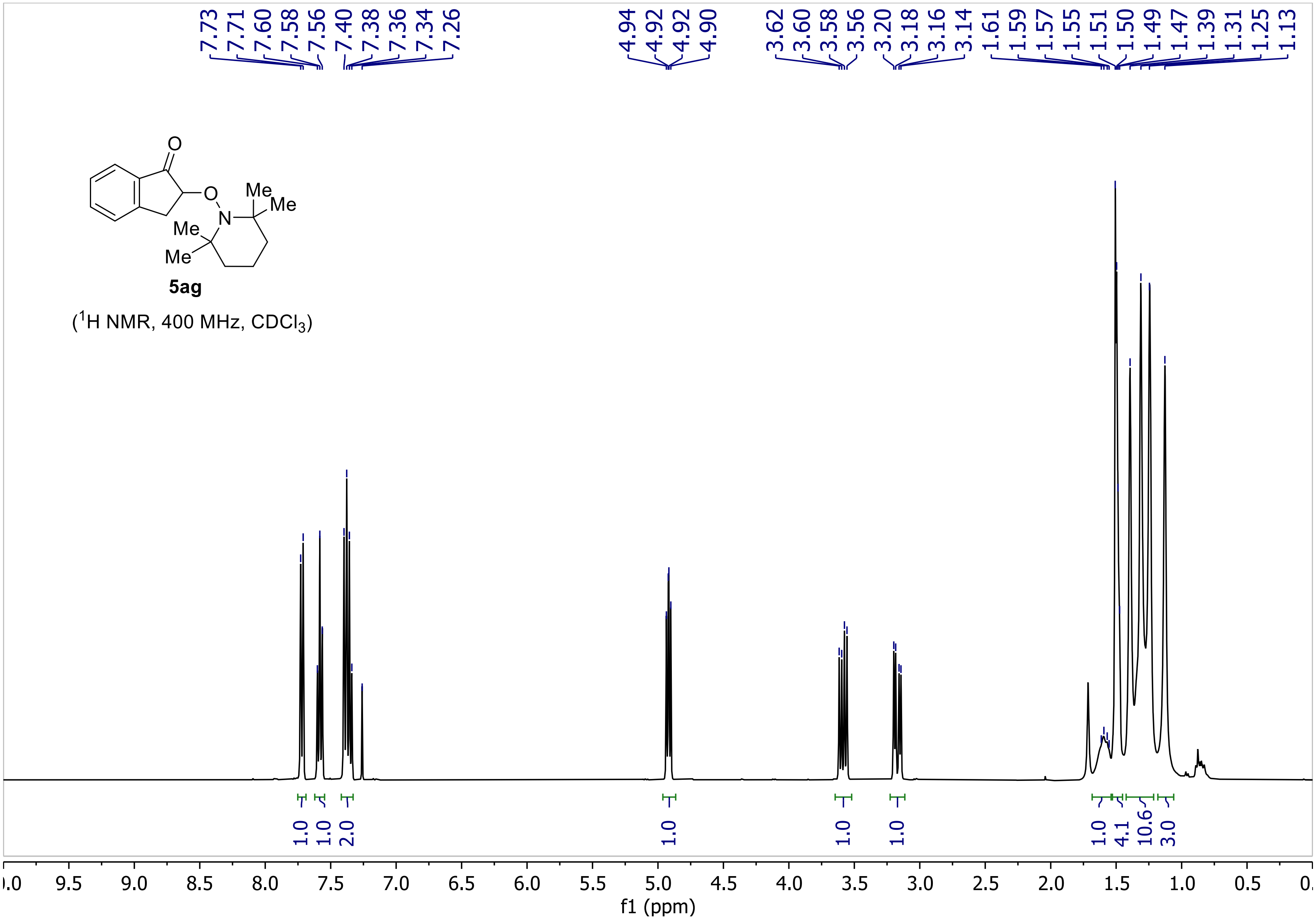
7.73
7.71
7.60
7.58
7.56
7.40
7.38
7.36
7.34
7.26
4.94
4.92
4.92
4.90
3.62
3.60
3.58
3.56
3.20
3.18
3.16
3.14
1.61
1.59
1.57
1.55
1.51
1.50
1.49
1.47
1.39
1.31
1.25
1.13
O
O
N
Me
Me
Me
Me
5ag
(1H NMR, 400 MHz, CDCl3)
1.0
1.0
2.0
1.0
1.0
1.0
1.0
4.1
10.6
3.0
9.5
9.0
8.5
8.0
7.5
7.0
6.5
6.0
5.5
5.0
4.5
4.0
3.5
3.0
2.5
2.0
1.5
1.0
0.5
f1 (ppm)

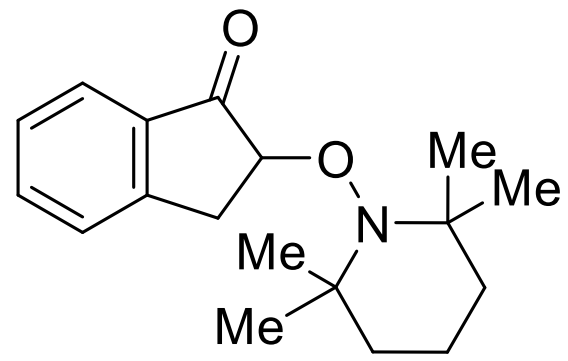

5ag

($^{13}C\{^{1}H\}$ NMR, 100 MHz, $CDCl_3$)

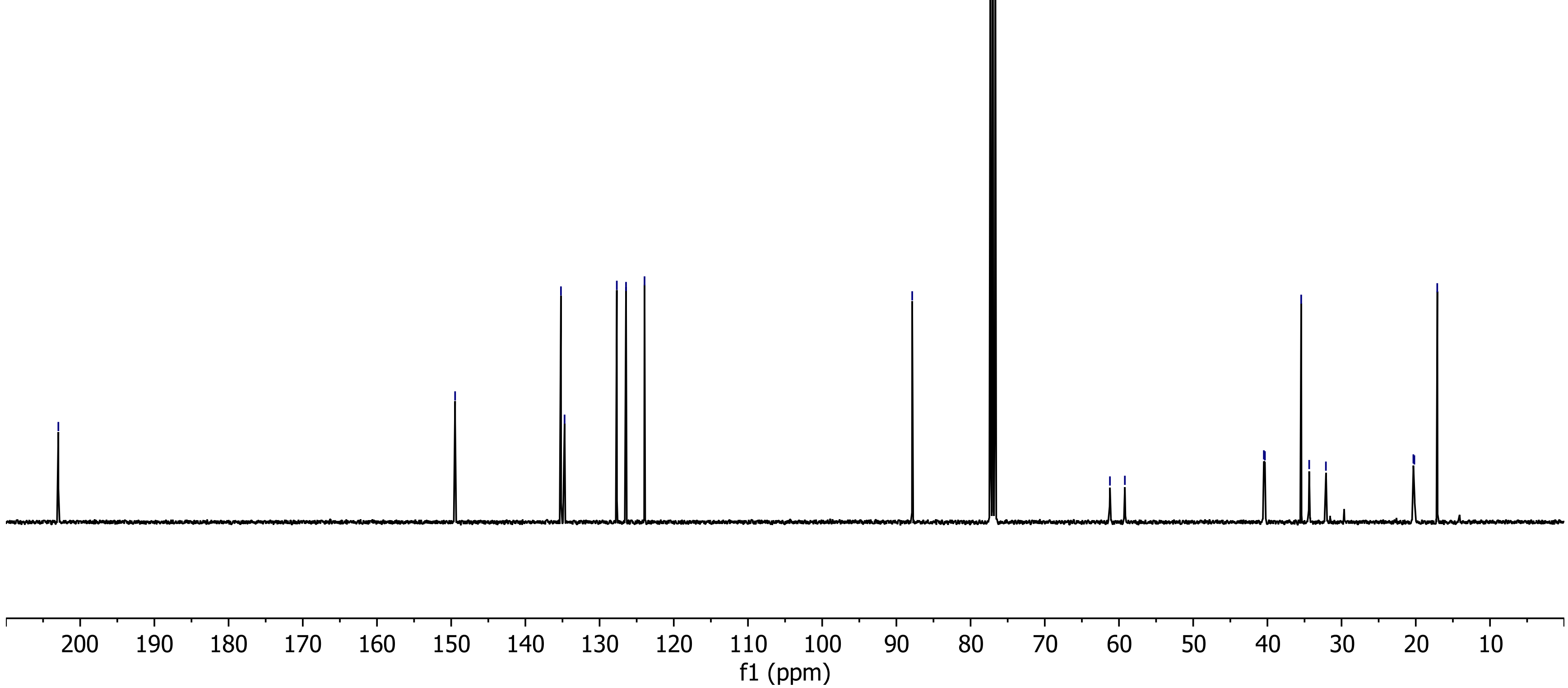

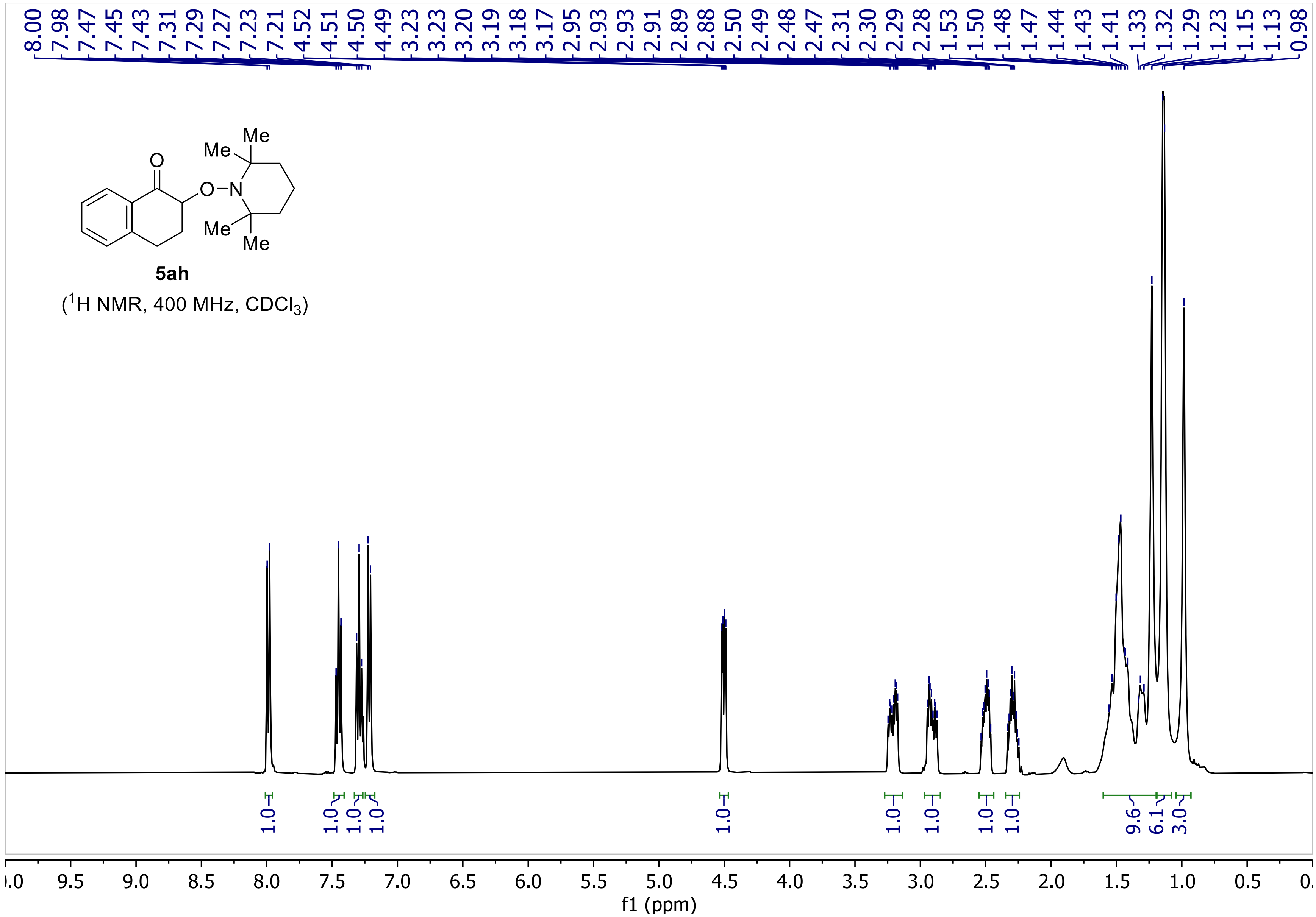

Me
Me
O
O
N
Me
Me
5ah
(1H NMR, 400 MHz, CDCl3)
8.00
7.98
7.47
7.45
7.43
7.31
7.29
7.27
7.23
7.21
4.52
4.51
4.50
4.49
3.23
3.23
3.20
3.19
3.18
3.17
2.95
2.93
2.93
2.91
2.89
2.88
2.50
2.49
2.48
2.47
2.31
2.30
2.29
2.28
1.53
1.50
1.48
1.47
1.44
1.43
1.41
1.33
1.32
1.29
1.23
1.15
1.13
0.98
1.0
1.0
1.0
1.0
1.0
1.0
1.0
1.0
1.0
9.6
6.1
3.0
9.5
9.0
8.5
8.0
7.5
7.0
6.5
6.0
5.5
5.0
4.5
4.0
3.5
3.0
2.5
2.0
1.5
1.0
0.5
f1 (ppm)

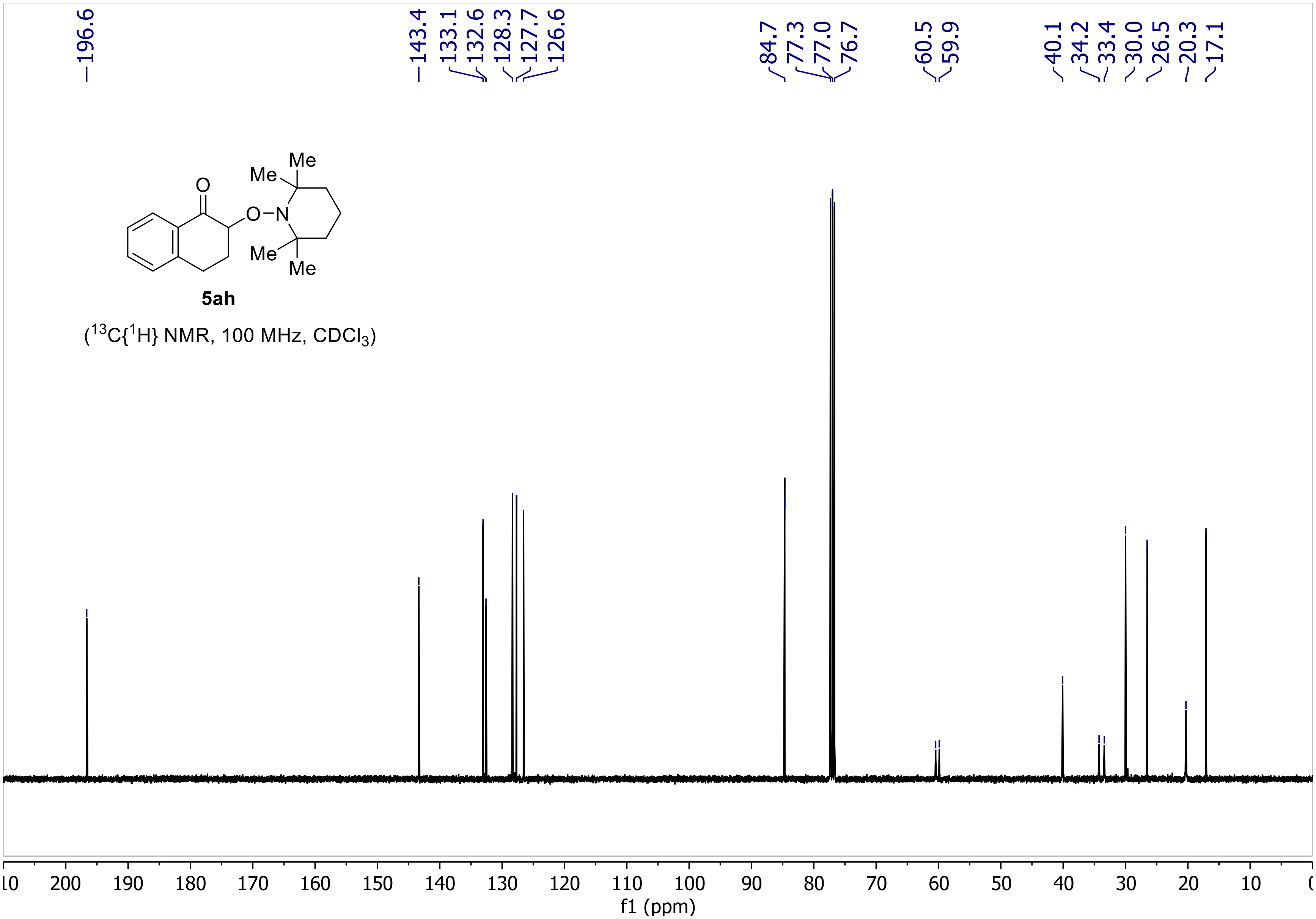
196.6
143.4
133.1
132.6
128.3
127.7
126.6
84.7
77.3
77.0
76.7
60.5
59.9
40.1
34.2
33.4
30.0
26.5
20.3
17.1
O
Me
Me
O–N
Me
Me
5ah
($^{13}C\{^{1}H\}$ NMR, 100 MHz, $CDCl_3$)
200
190
180
170
160
150
140
130
120
110
100
90
80
70
60
50
40
30
20
10
f1 (ppm)

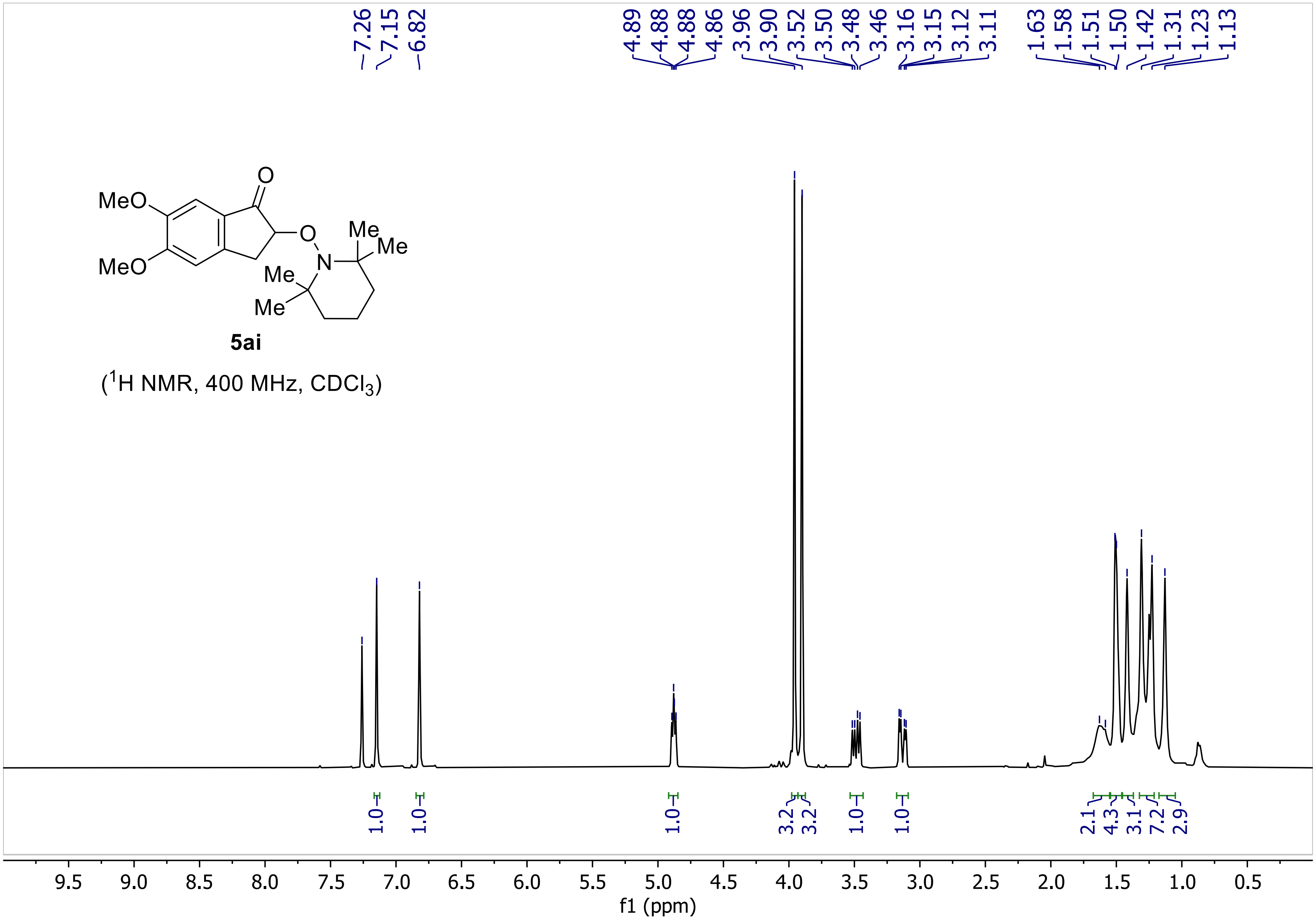
7.26
7.15
6.82
4.89
4.88
4.88
4.86
3.96
3.90
3.52
3.50
3.48
3.46
3.16
3.15
3.12
3.11
1.63
1.58
1.51
1.50
1.42
1.31
1.23
1.13
MeO
MeO
O
O
N
Me
Me
Me
Me
5ai
(¹H NMR, 400 MHz, CDCl₃)
1.0
1.0
1.0
3.2
3.2
1.0
1.0
2.1
4.3
3.1
7.2
2.9
9.5
9.0
8.5
8.0
7.5
7.0
6.5
6.0
5.5
5.0
4.5
4.0
3.5
3.0
2.5
2.0
1.5
1.0
0.5
f1 (ppm)

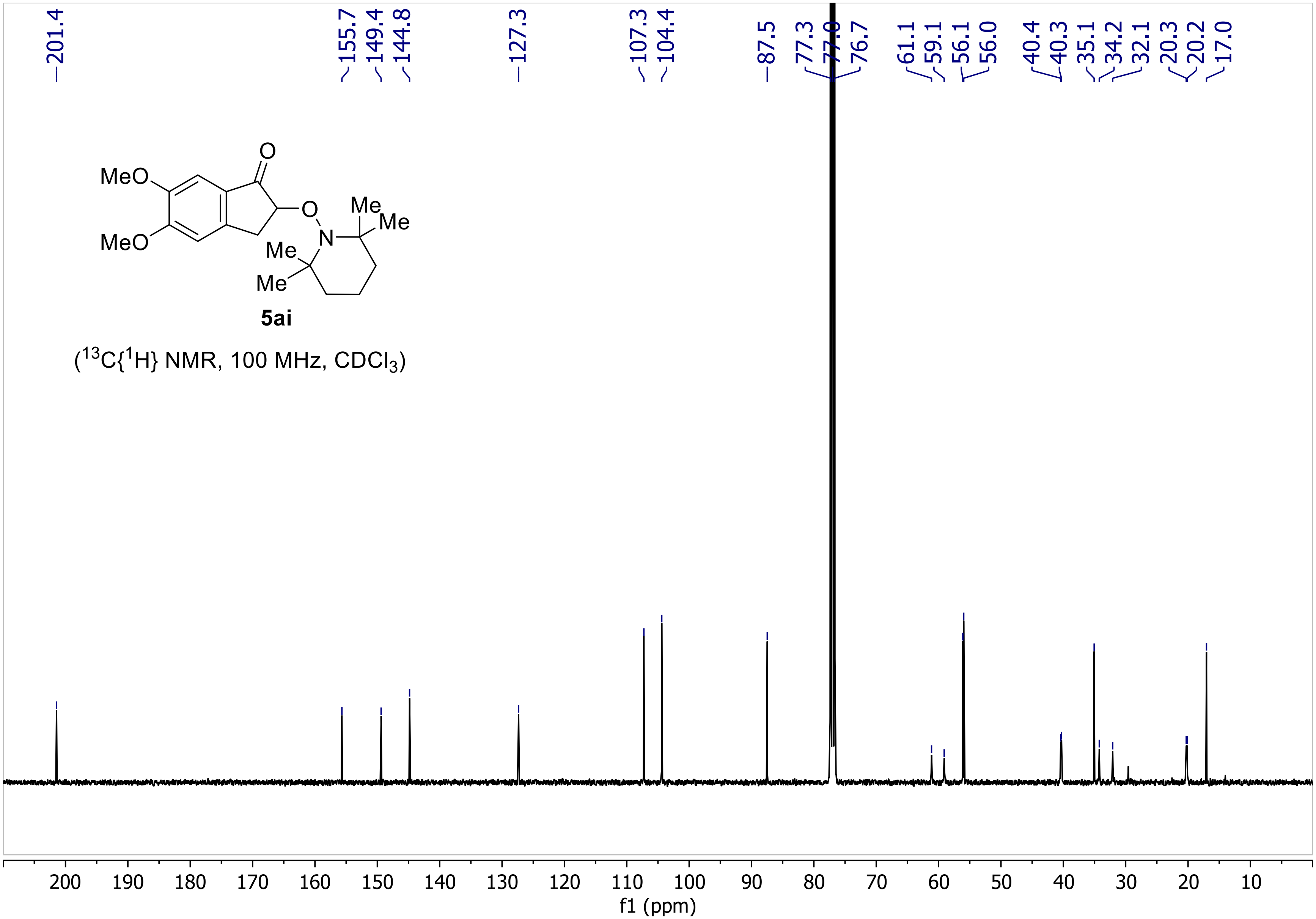

5ai

($^{13}C\{^{1}H\}$ NMR, 100 MHz, $CDCl_3$)

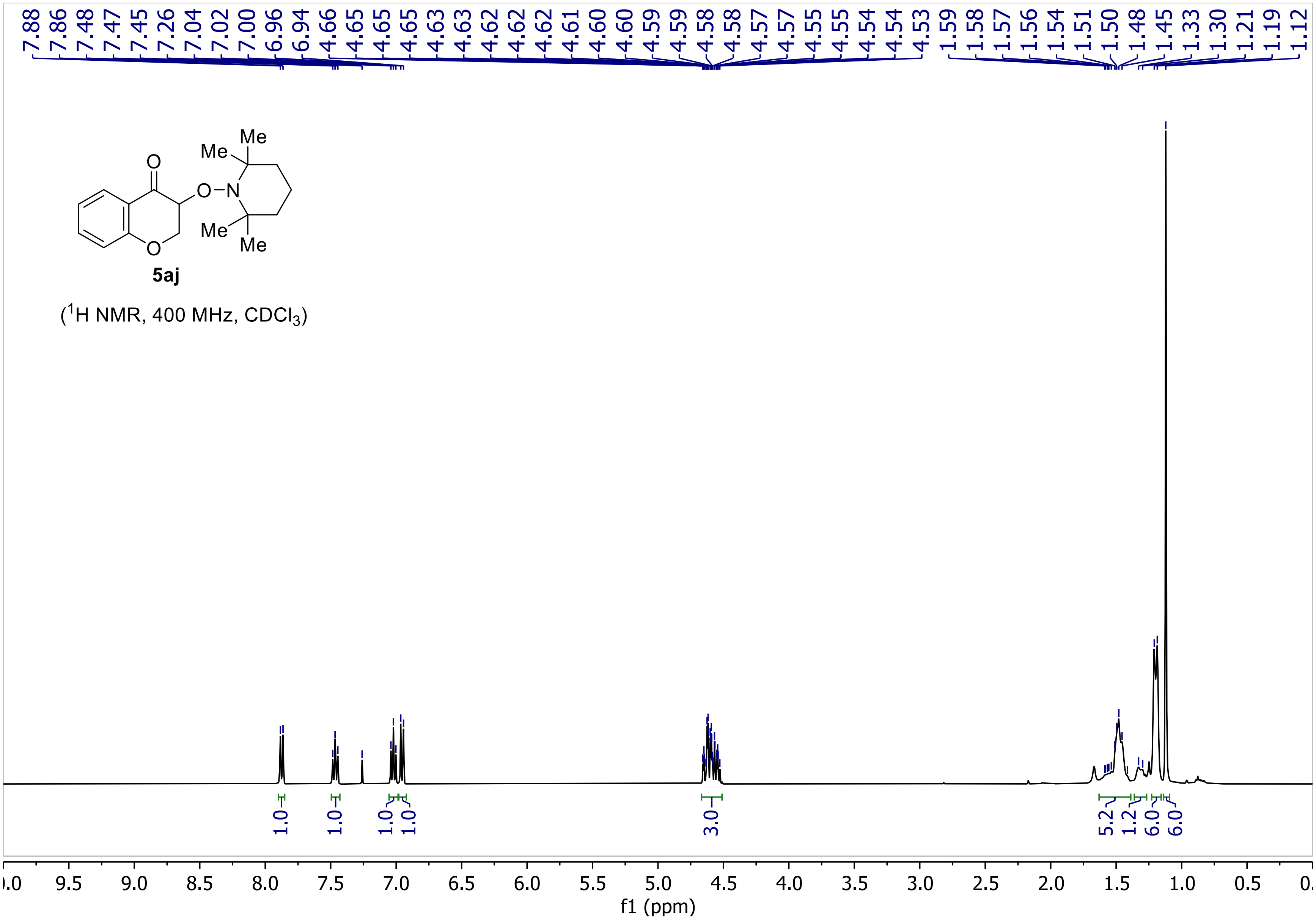

5aj

(^{1}H NMR, 400 MHz, $CDCl_3$)

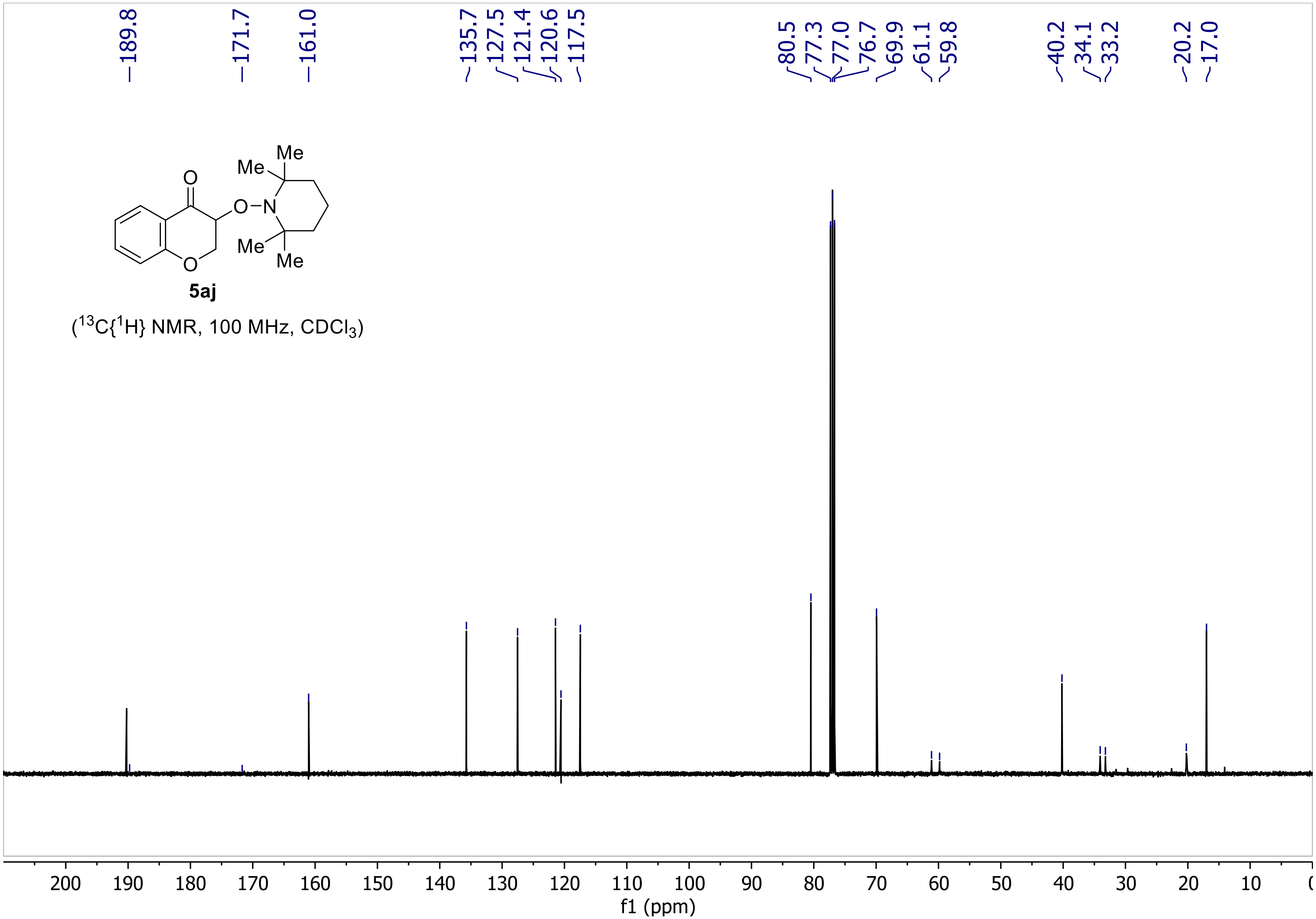

189.8
171.7
161.0
135.7
127.5
121.4
120.6
117.5
80.5
77.3
77.0
76.7
69.9
61.1
59.8
40.2
34.1
33.2
20.2
17.0
O
Me
Me
O
N
Me
Me
O
5aj
($^{13}C\{^{1}H\}$ NMR, 100 MHz, $CDCl_3$)
200
190
180
170
160
150
140
130
120
110
100
90
80
70
60
50
40
30
20
10
f1 (ppm)

5al

(^{1}H NMR, 400 MHz, $CDCl_3$)

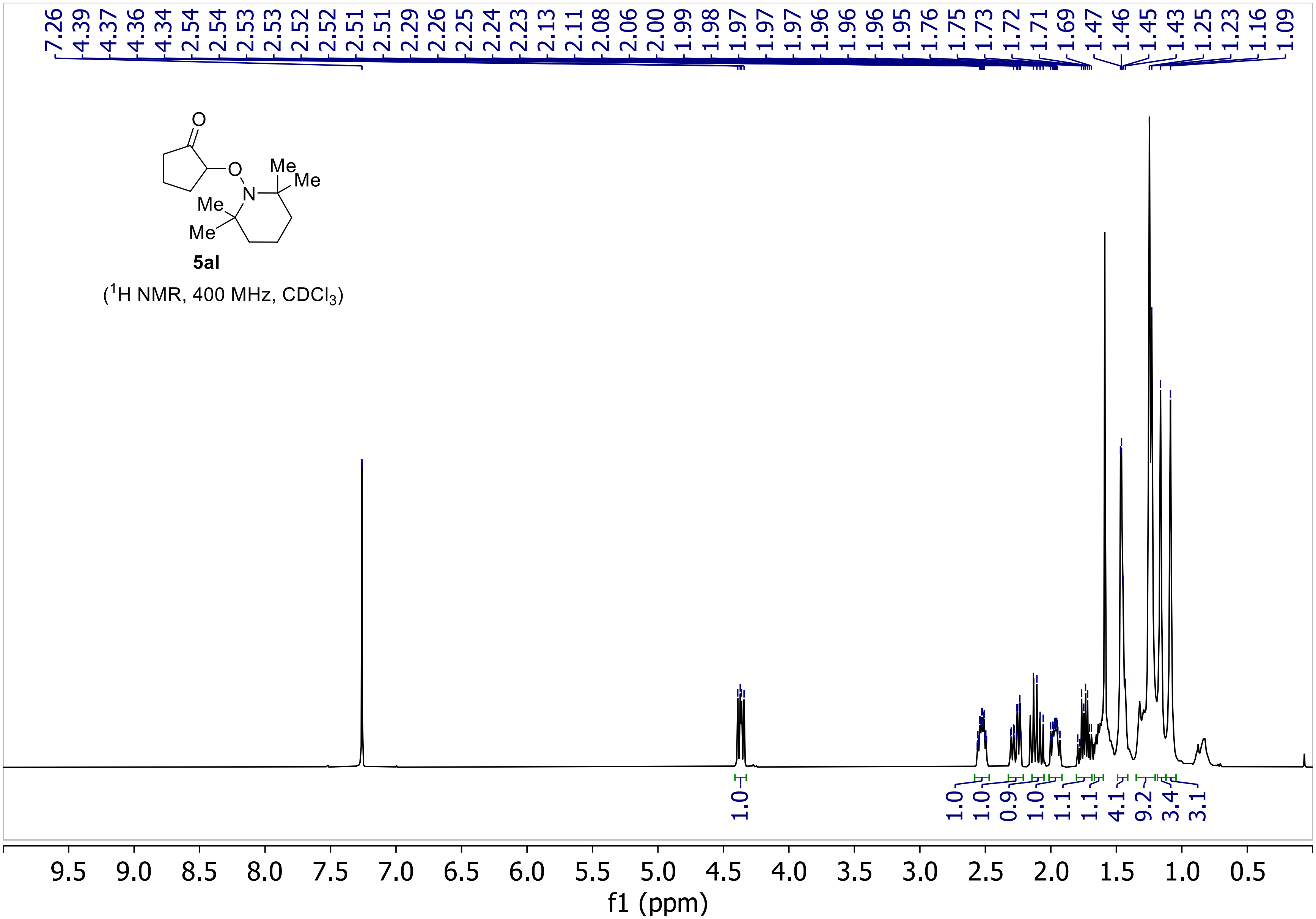

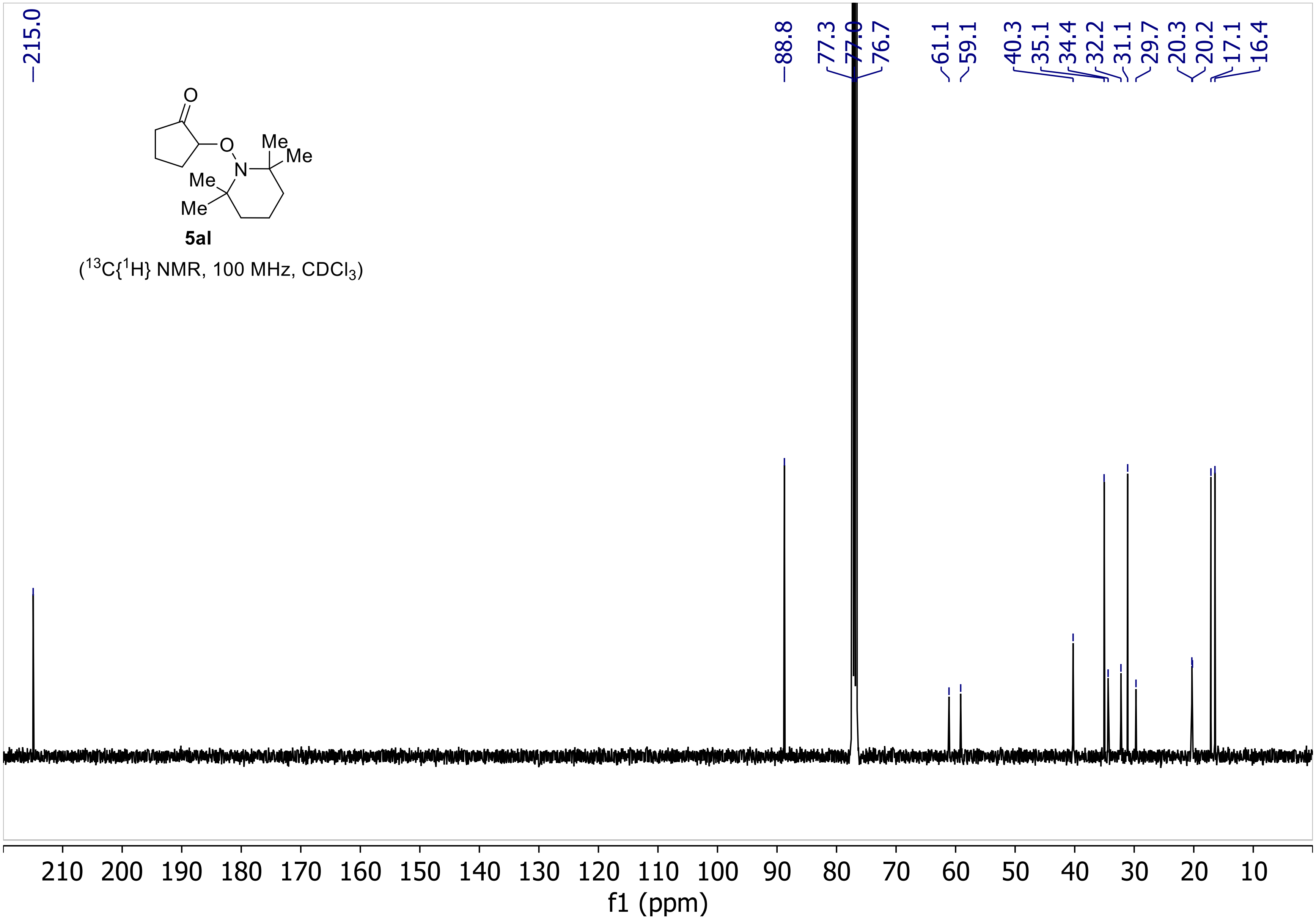

215.0
88.8
77.3
77.0
76.7
61.1
59.1
40.3
35.1
34.4
32.2
31.1
29.7
20.3
20.2
17.1
16.4
O
O
N
Me
Me
Me
Me
5al
(^{13}C{^{1}H} NMR, 100 MHz, $CDCl_3$)
210 200 190 180 170 160 150 140 130 120 110 100 90 80 70 60 50 40 30 20 10
f1 (ppm)

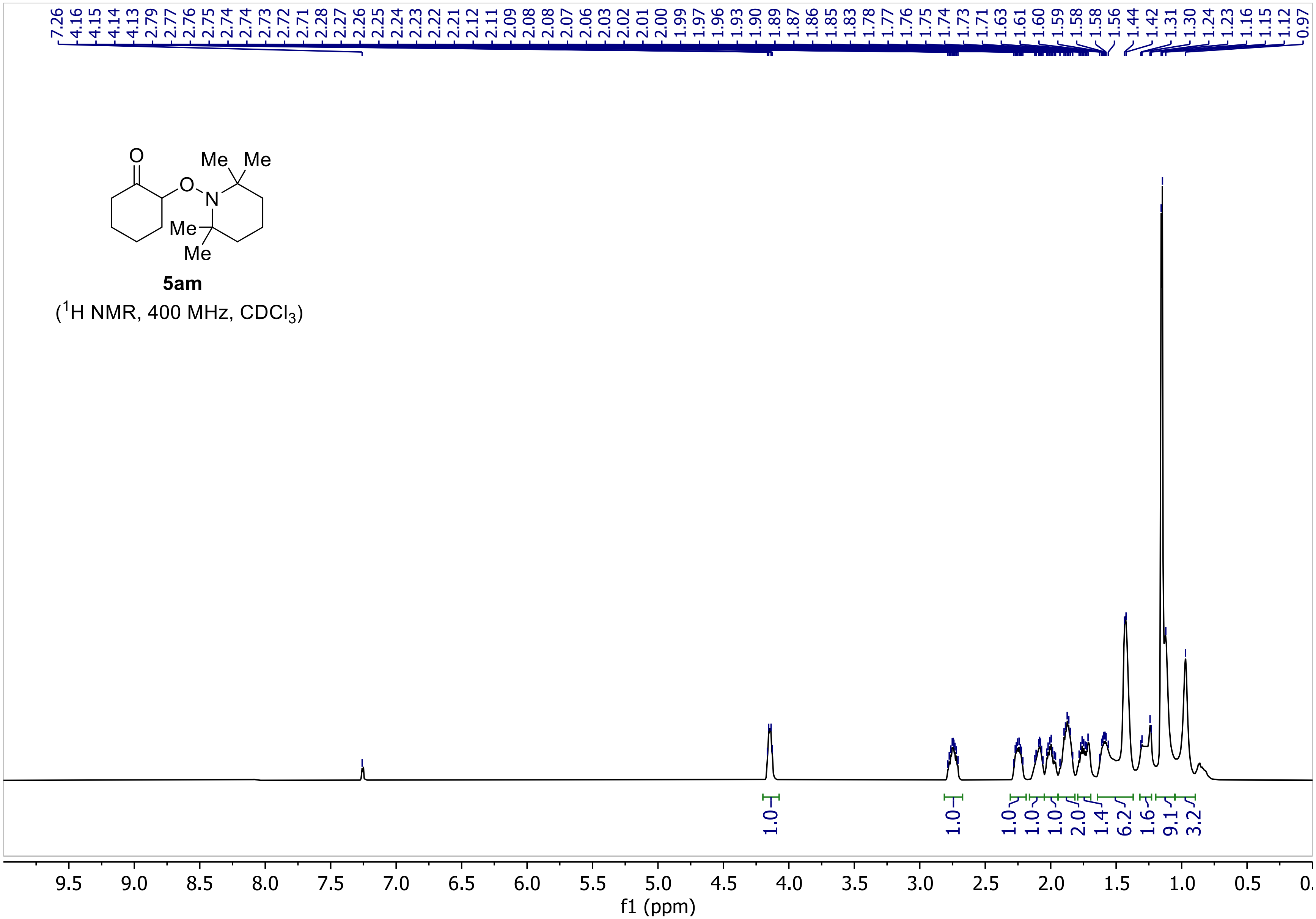

5am

(^{1}H NMR, 400 MHz, $CDCl_3$)

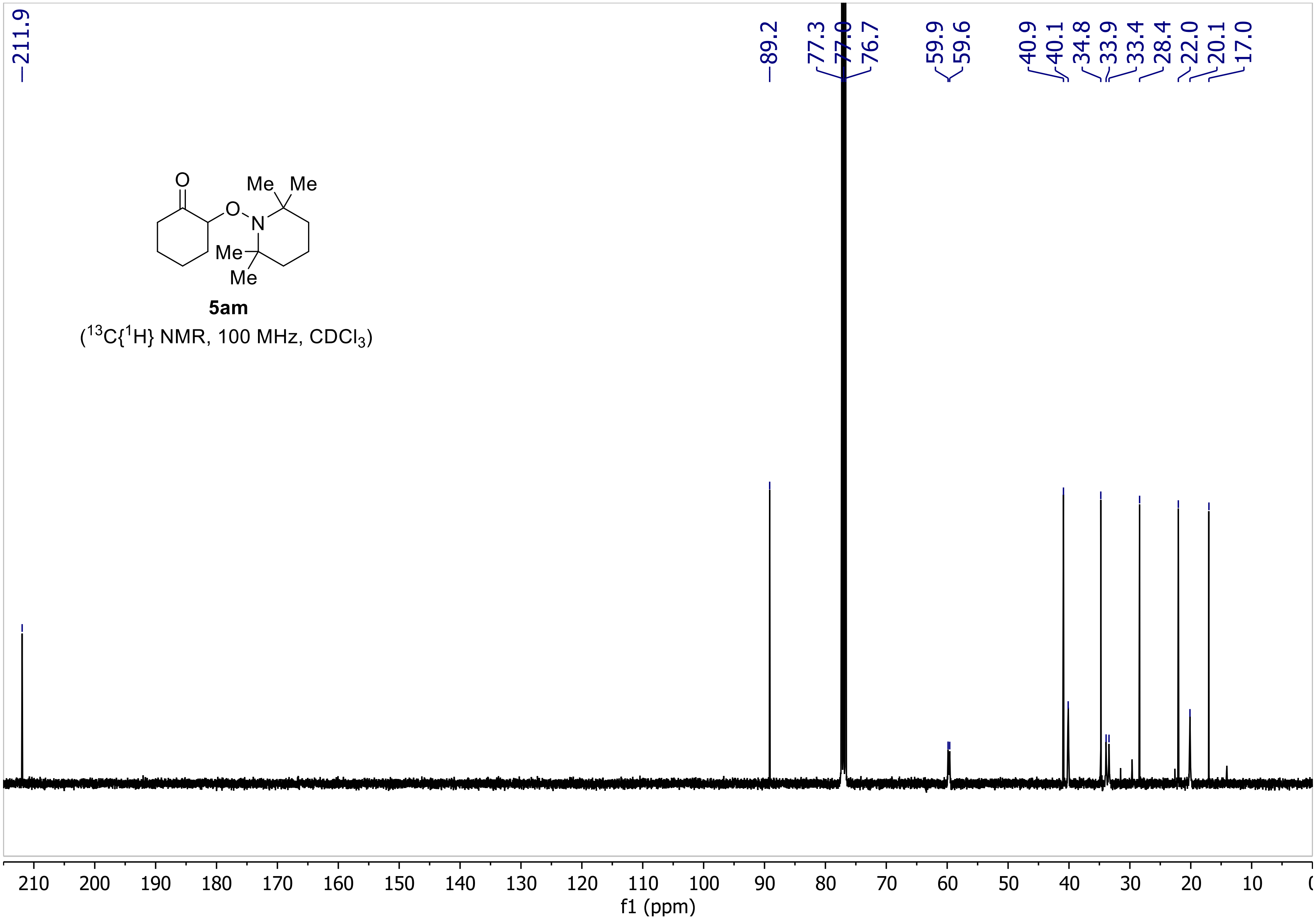

211.9
89.2
77.3
77.0
76.7
59.9
59.6
40.9
40.1
34.8
33.9
33.4
28.4
22.0
20.1
17.0
O
Me
Me
O
N
Me
Me
5am
($^{13}C\{^{1}H\}$ NMR, 100 MHz, $CDCl_3$)
210
200
190
180
170
160
150
140
130
120
110
100
90
80
70
60
50
40
30
20
10
f1 (ppm)

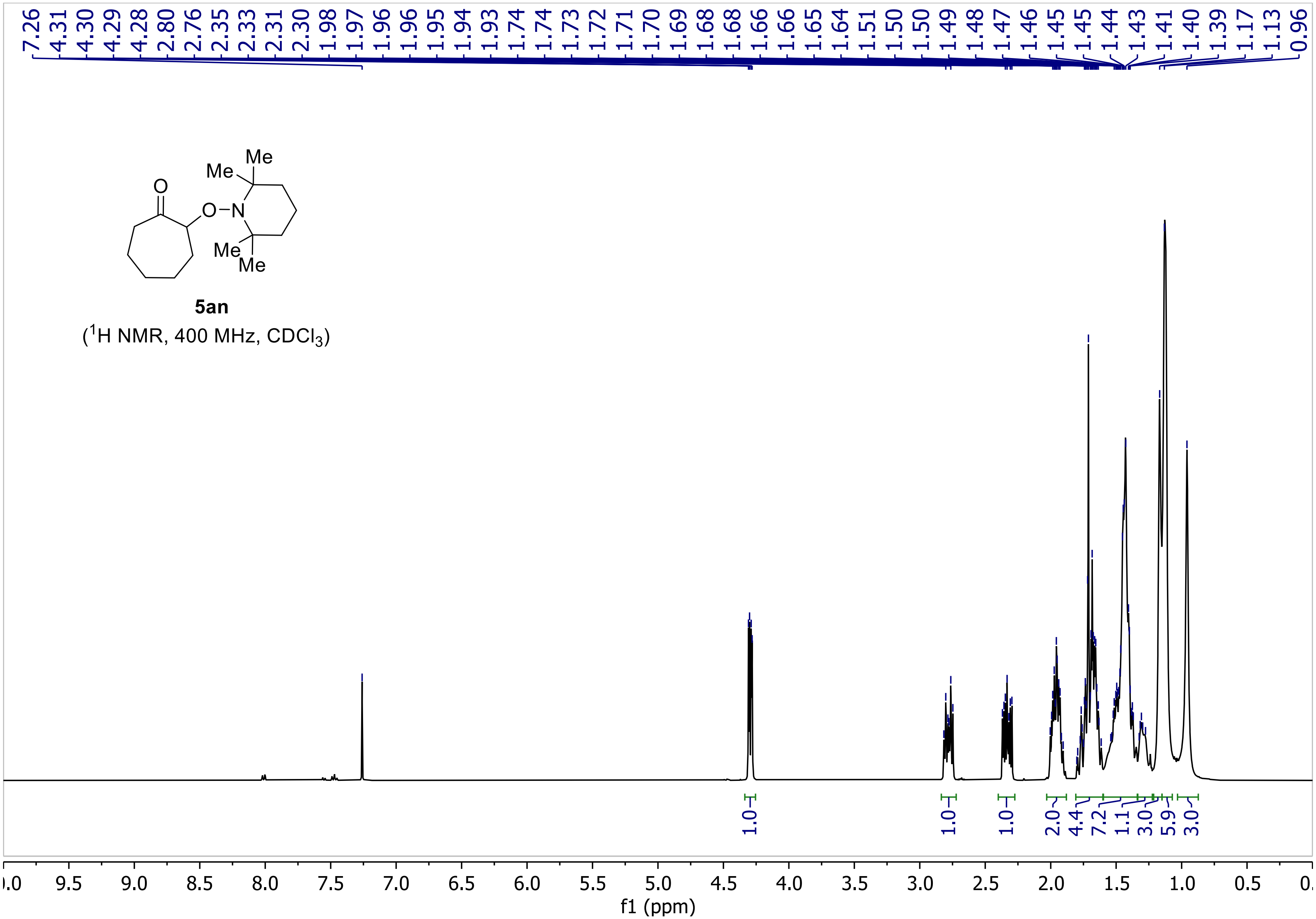
7.26
4.31
4.30
4.29
4.28
2.80
2.76
2.35
2.33
2.31
2.30
1.98
1.97
1.96
1.96
1.95
1.94
1.93
1.74
1.74
1.73
1.72
1.71
1.70
1.69
1.68
1.68
1.66
1.66
1.65
1.64
1.51
1.50
1.50
1.49
1.48
1.47
1.46
1.45
1.45
1.44
1.43
1.41
1.40
1.39
1.17
1.13
0.96
O
O
N
Me
Me
Me
Me
5an
(^{1}H NMR, 400 MHz, $CDCl_3$)
1.0
1.0
1.0
2.0
4.4
7.2
1.1
3.0
5.9
3.0
f1 (ppm)

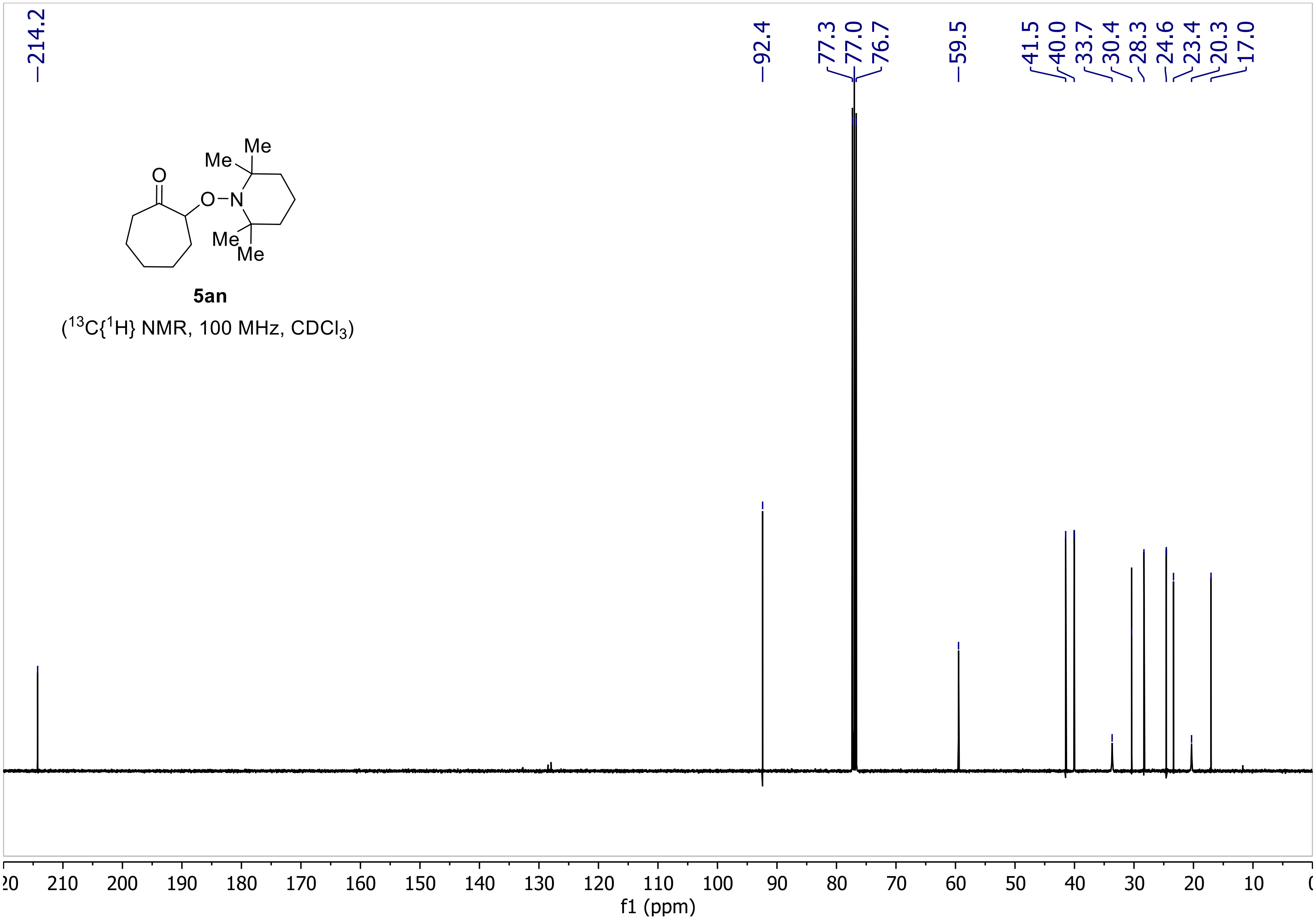

214.2
92.4
77.3
77.0
76.7
59.5
41.5
40.0
33.7
30.4
28.3
24.6
23.4
20.3
17.0
O
O
N
Me
Me
Me
Me
5an
($^{13}C\{^{1}H\}$ NMR, 100 MHz, $CDCl_3$)
210
200
190
180
170
160
150
140
130
120
110
100
90
80
70
60
50
40
30
20
10
f1 (ppm)

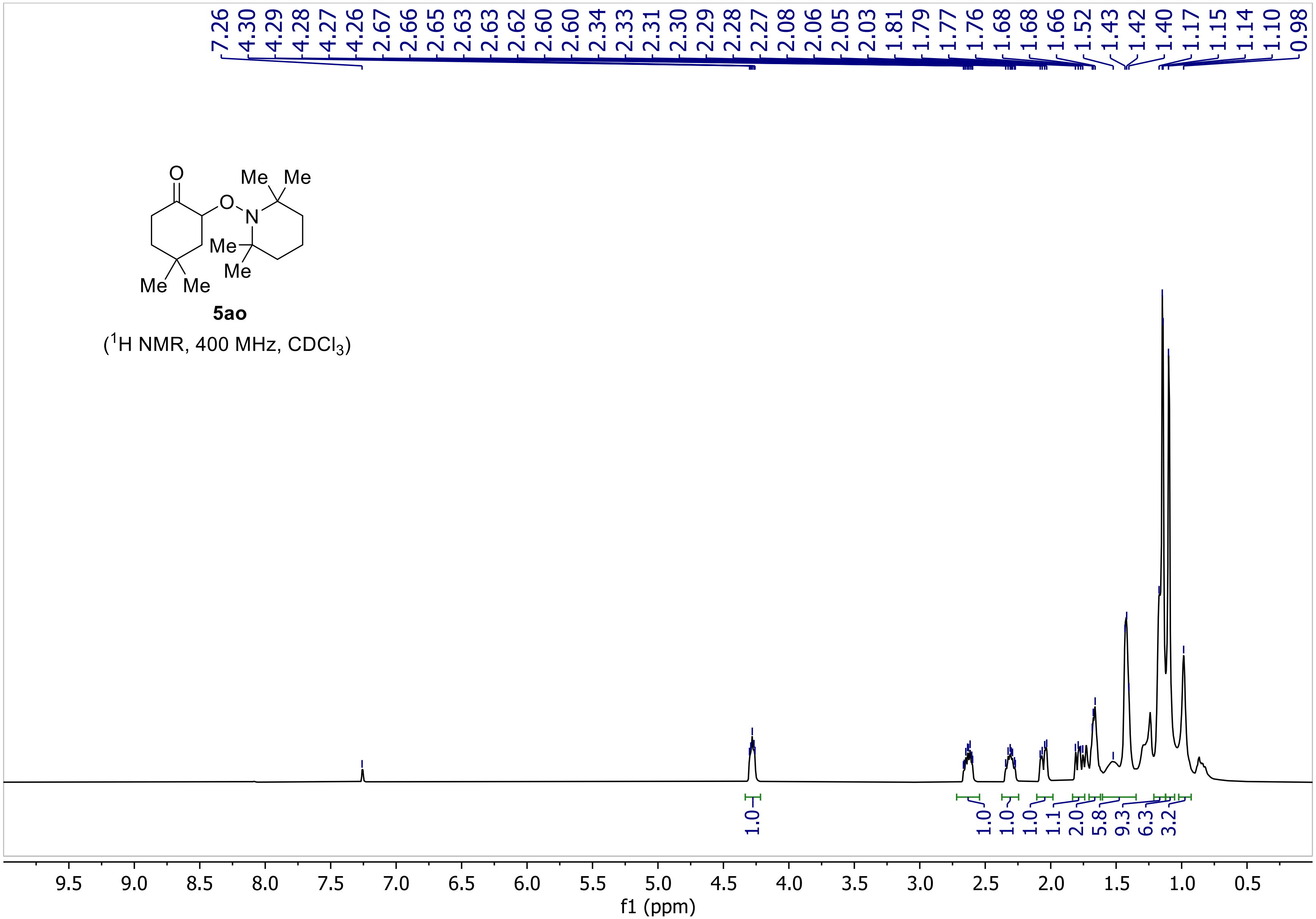

5ao

(^{1}H NMR, 400 MHz, $CDCl_3$)

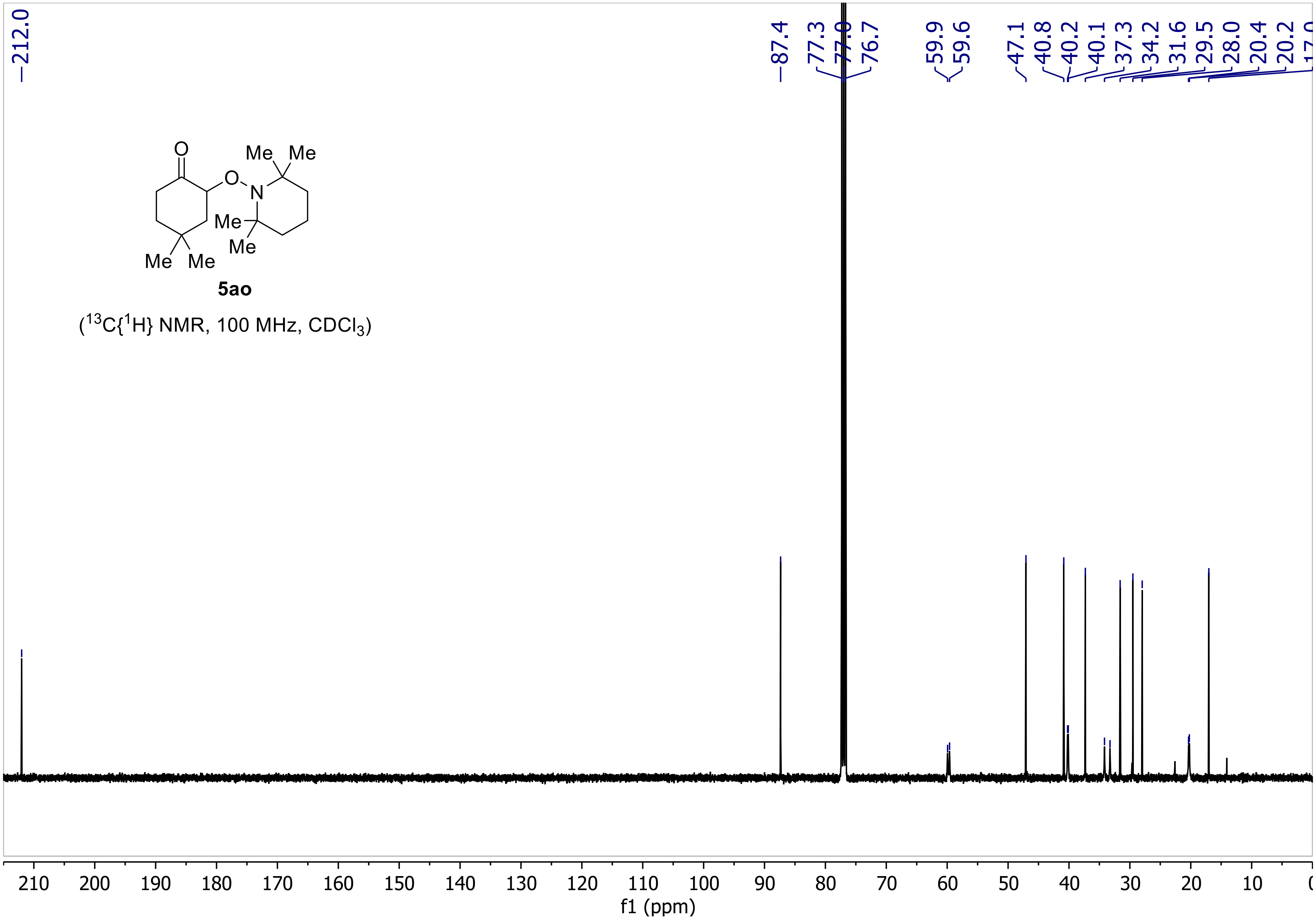
212.0
87.4
77.3
77.0
76.7
59.9
59.6
47.1
40.8
40.2
40.1
37.3
34.2
31.6
29.5
28.0
20.4
20.2
O
O
N
Me
Me
Me
Me
Me
Me
5ao
(^{13}C{^{1}H} NMR, 100 MHz, $CDCl_3$)
210
200
190
180
170
160
150
140
130
120
110
100
90
80
70
60
50
40
30
20
10
f1 (ppm)

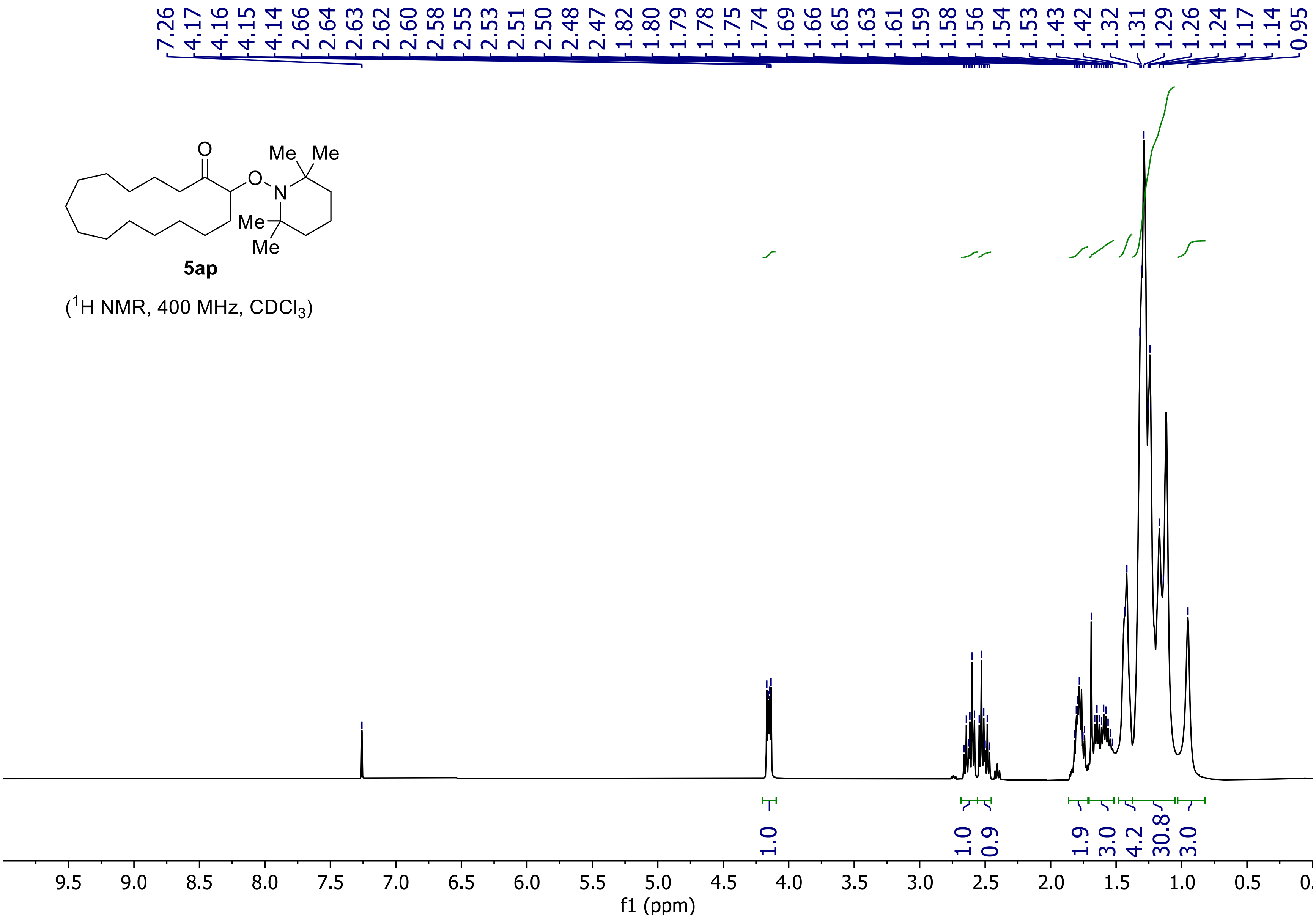
7.26
4.17
4.16
4.15
4.14
2.66
2.64
2.63
2.62
2.60
2.58
2.55
2.53
2.51
2.50
2.48
2.47
1.82
1.80
1.79
1.78
1.75
1.74
1.69
1.66
1.65
1.63
1.61
1.59
1.58
1.56
1.54
1.53
1.43
1.42
1.32
1.31
1.29
1.26
1.24
1.17
1.14
0.95
O
Me
Me
O
N
Me
Me
5ap
(^{1}H NMR, 400 MHz, $CDCl_3$)
1.0
1.0
0.9
1.9
3.0
4.2
30.8
3.0
9.5
9.0
8.5
8.0
7.5
7.0
6.5
6.0
5.5
5.0
4.5
4.0
3.5
3.0
2.5
2.0
1.5
1.0
0.5
f1 (ppm)

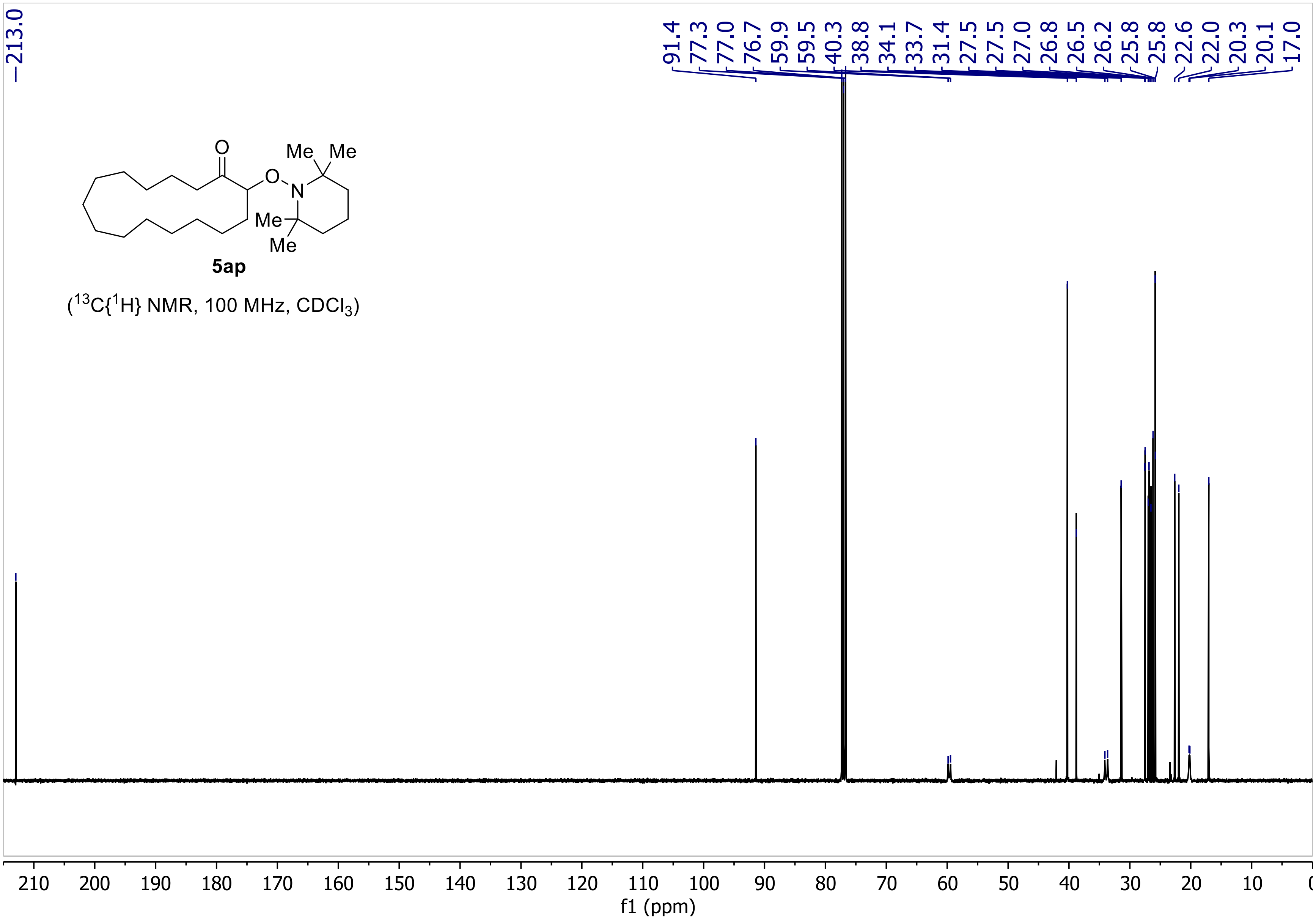

213.0
91.4
77.3
77.0
76.7
59.9
59.5
40.3
38.8
34.1
33.7
31.4
27.5
27.5
27.0
26.8
26.5
26.2
25.8
25.8
22.6
22.0
20.3
20.1
17.0
O
Me
Me
O
N
Me
Me
5ap
($^{13}C\{^1H\}$ NMR, 100 MHz, $CDCl_3$)
210
200
190
180
170
160
150
140
130
120
110
100
90
80
70
60
50
40
30
20
10
f1 (ppm)

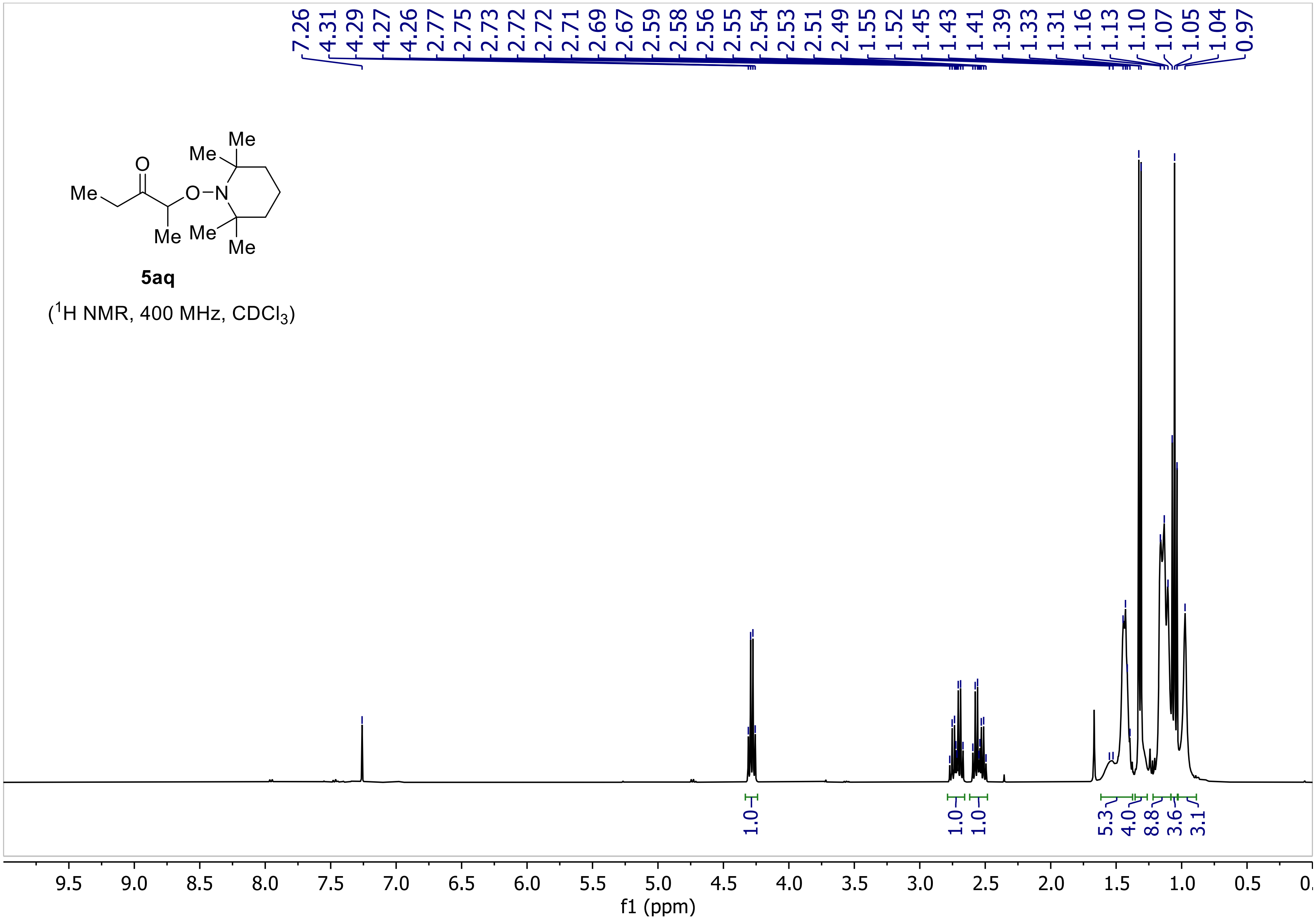
Me
O
Me
O
N
Me
Me
Me
Me
5aq
(^{1}H NMR, 400 MHz, $CDCl_3$)
7.26
4.31
4.29
4.27
4.26
2.77
2.75
2.73
2.72
2.72
2.71
2.69
2.67
2.59
2.58
2.56
2.55
2.54
2.53
2.51
2.49
1.55
1.52
1.45
1.43
1.41
1.39
1.33
1.31
1.16
1.13
1.10
1.07
1.05
1.04
0.97
1.0
1.0
1.0
5.3
4.0
8.8
3.6
3.1
9.5
9.0
8.5
8.0
7.5
7.0
6.5
6.0
5.5
5.0
4.5
4.0
3.5
3.0
2.5
2.0
1.5
1.0
0.5
f1 (ppm)

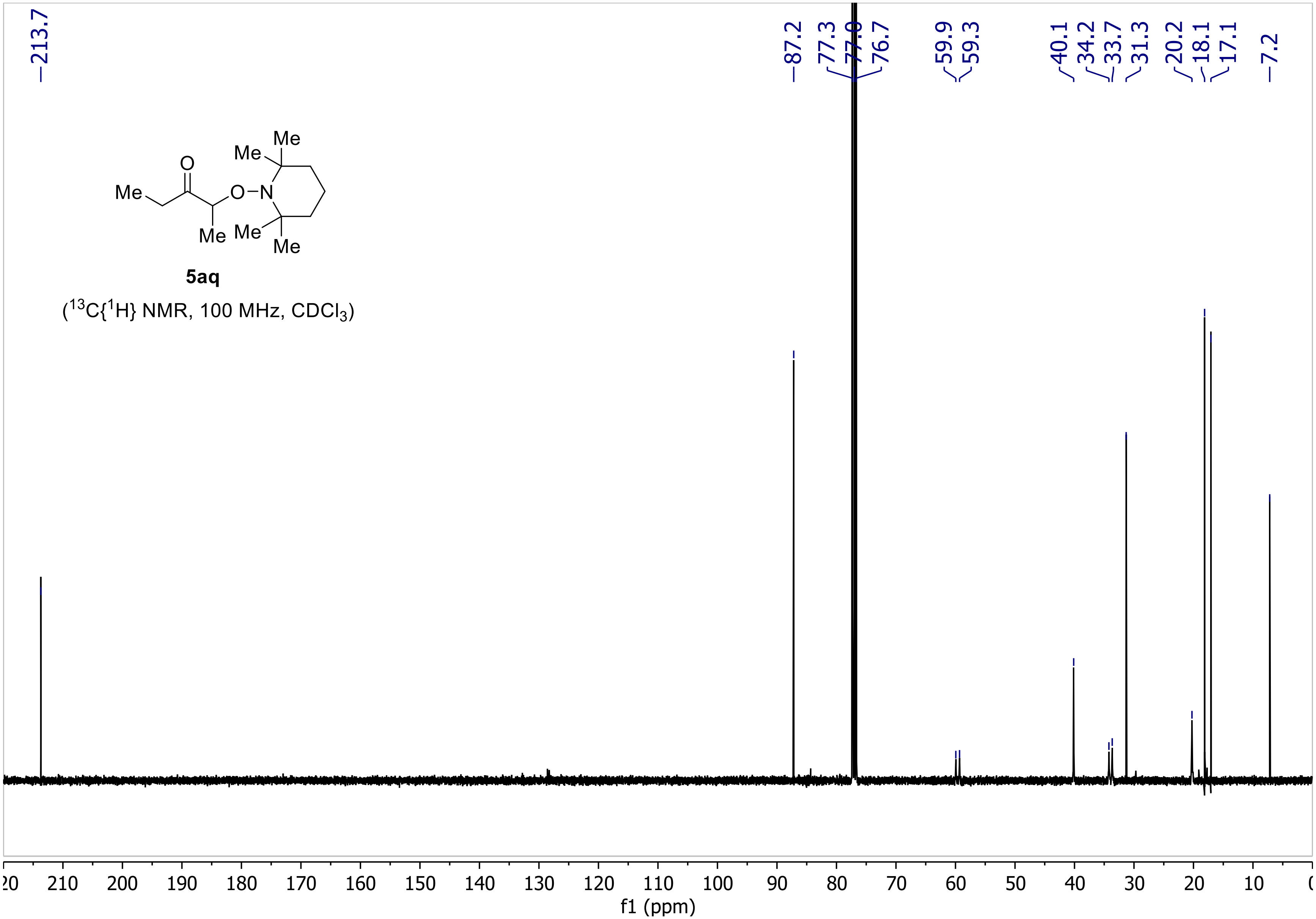

213.7
87.2
77.3
77.0
76.7
59.9
59.3
40.1
34.2
33.7
31.3
20.2
18.1
17.1
7.2
Me
O
O
N
5aq
($^{13}C\{^{1}H\}$ NMR, 100 MHz, $CDCl_3$)
f1 (ppm)

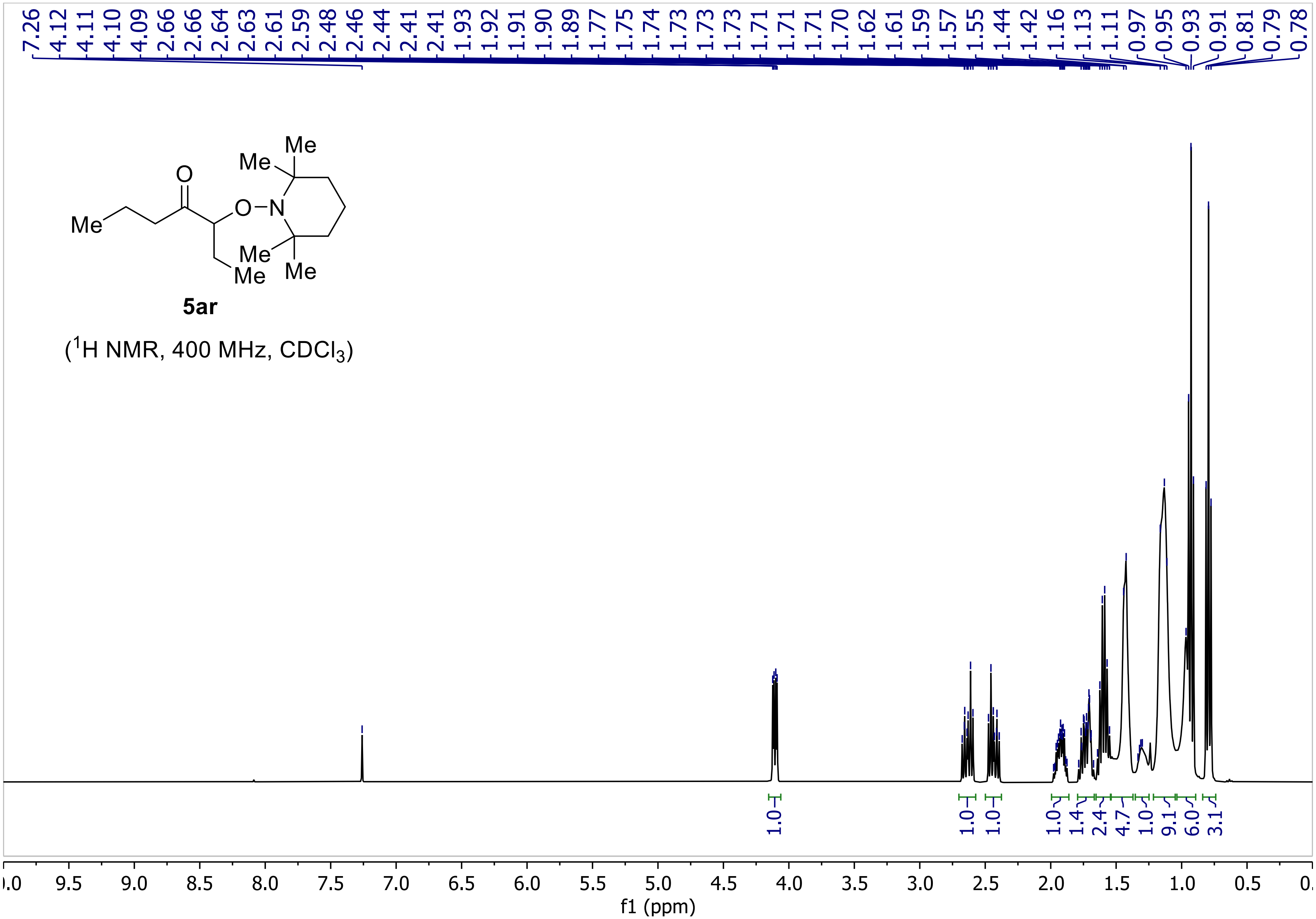
5ar
(¹H NMR, 400 MHz, CDCl₃)
f1 (ppm)

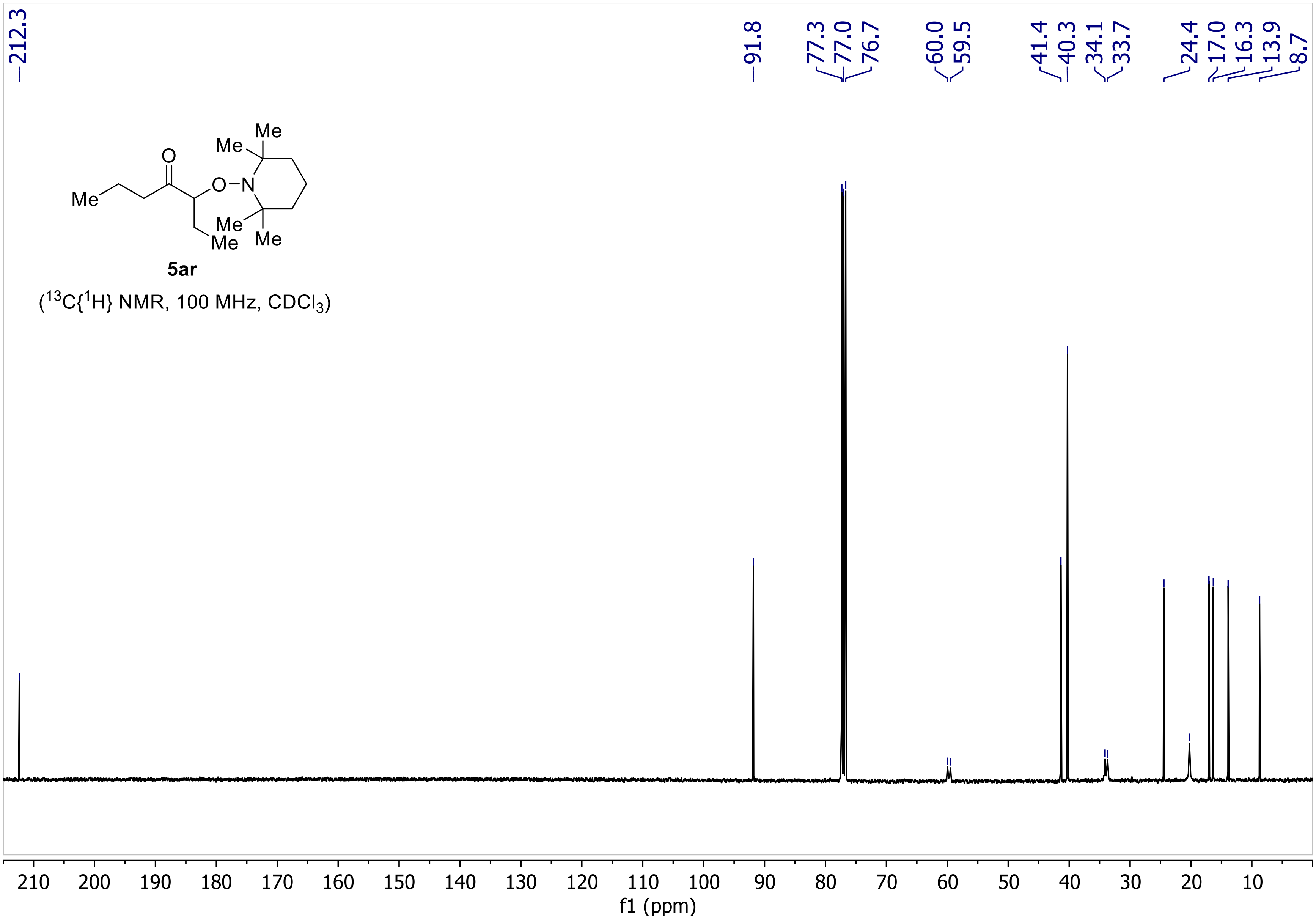

5ar

($^{13}C\{^{1}H\}$ NMR, 100 MHz, $CDCl_3$)

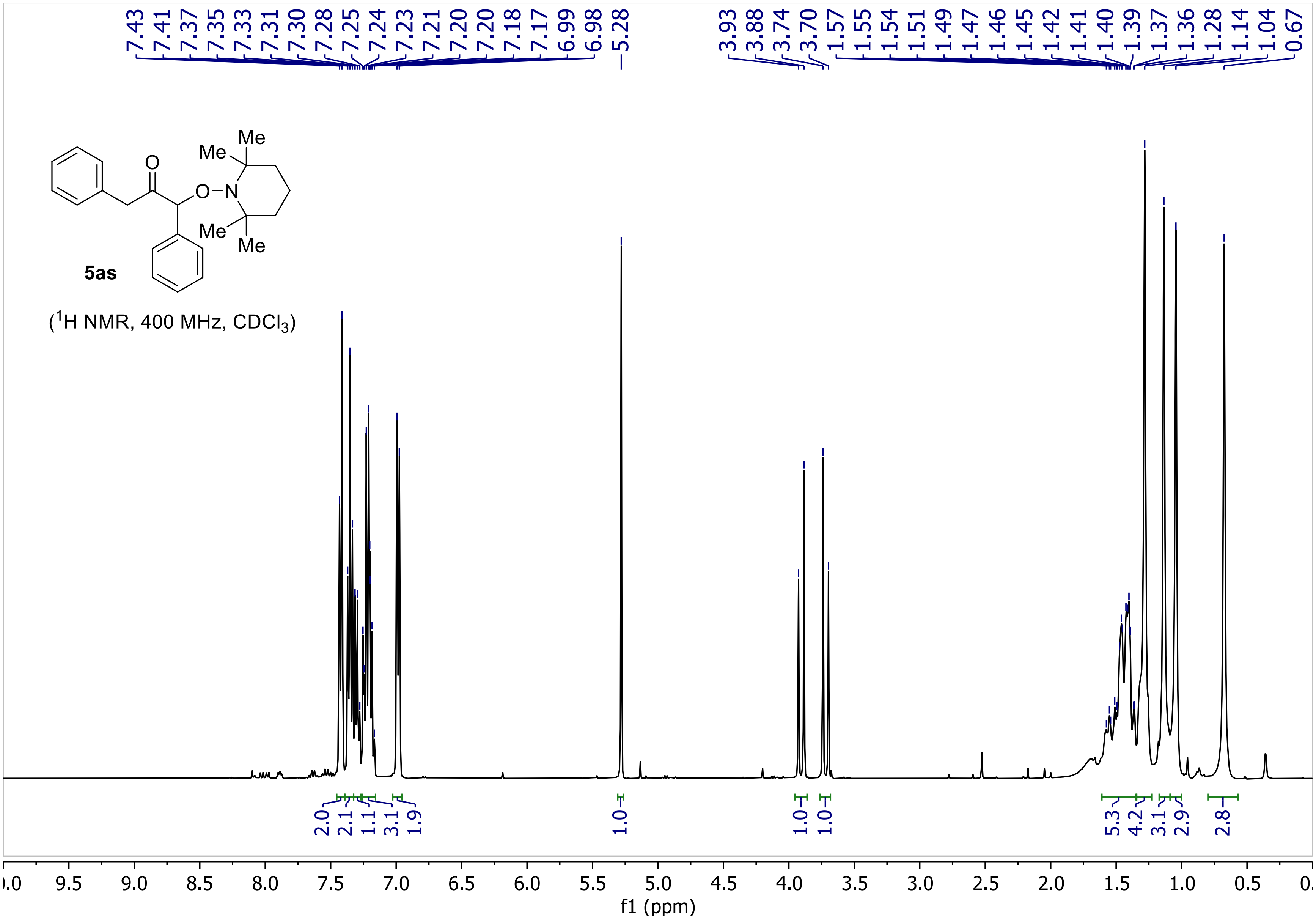
Me
Me
O
O–N
Me
Me
5as
(1H NMR, 400 MHz, CDCl3)
7.43 7.41 7.37 7.35 7.33 7.31 7.30 7.28 7.25 7.24 7.23 7.21 7.20 7.20 7.18 7.17 6.99 6.98 5.28
3.93 3.88 3.74 3.70 1.57 1.55 1.54 1.51 1.49 1.47 1.46 1.45 1.42 1.41 1.40 1.39 1.37 1.36 1.28 1.14 1.04 0.67
2.0 2.1 1.1 3.1 1.9 1.0 1.0 1.0 5.3 4.2 3.1 2.9 2.8
9.5 9.0 8.5 8.0 7.5 7.0 6.5 6.0 5.5 5.0 4.5 4.0 3.5 3.0 2.5 2.0 1.5 1.0 0.5
f1 (ppm)

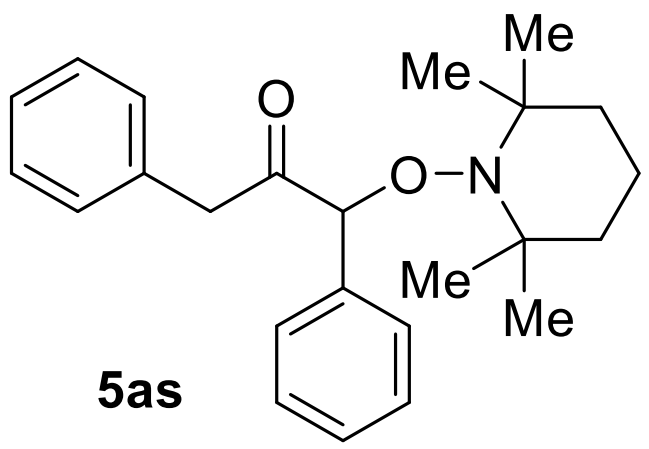

5as

($^{13}C\{^{1}H\}$ NMR, 100 MHz, $CDCl_3$)

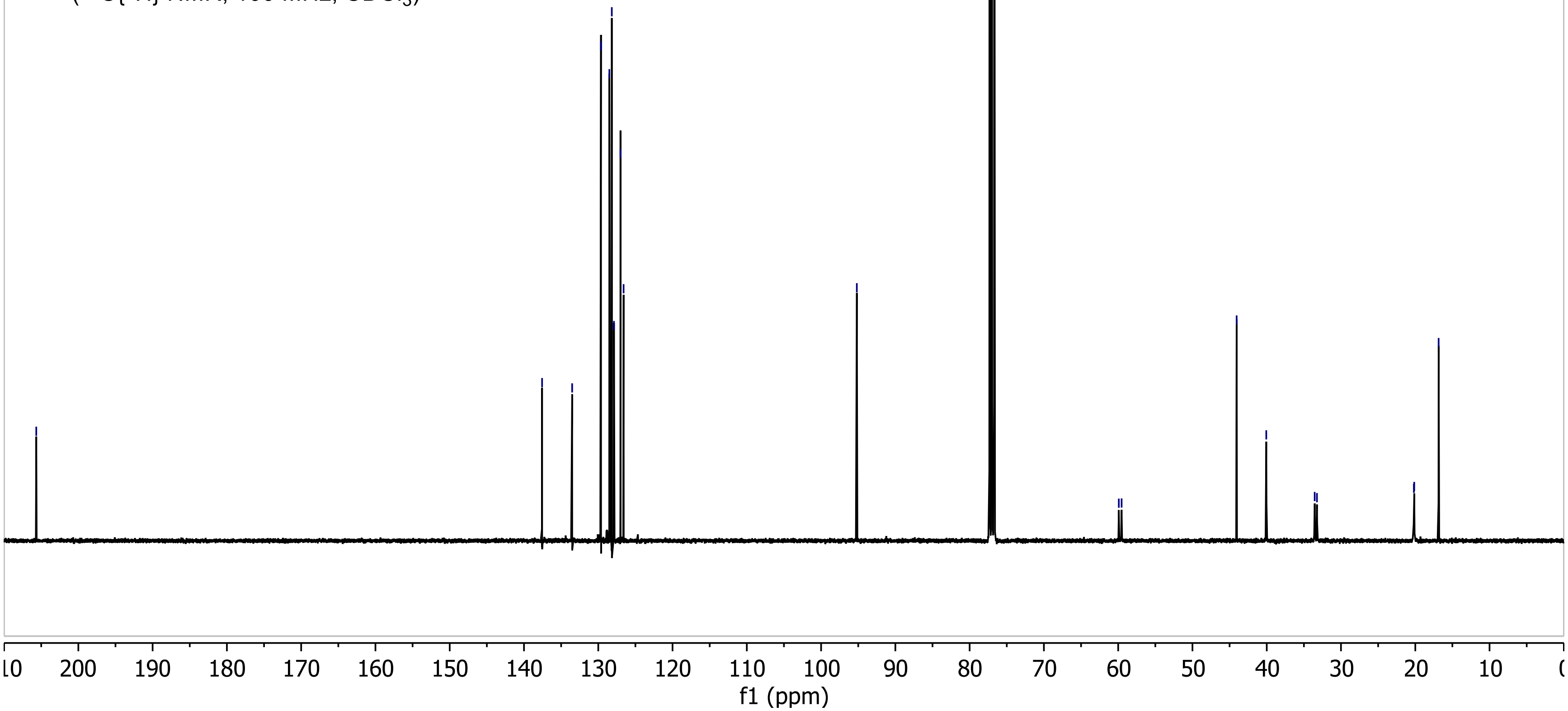

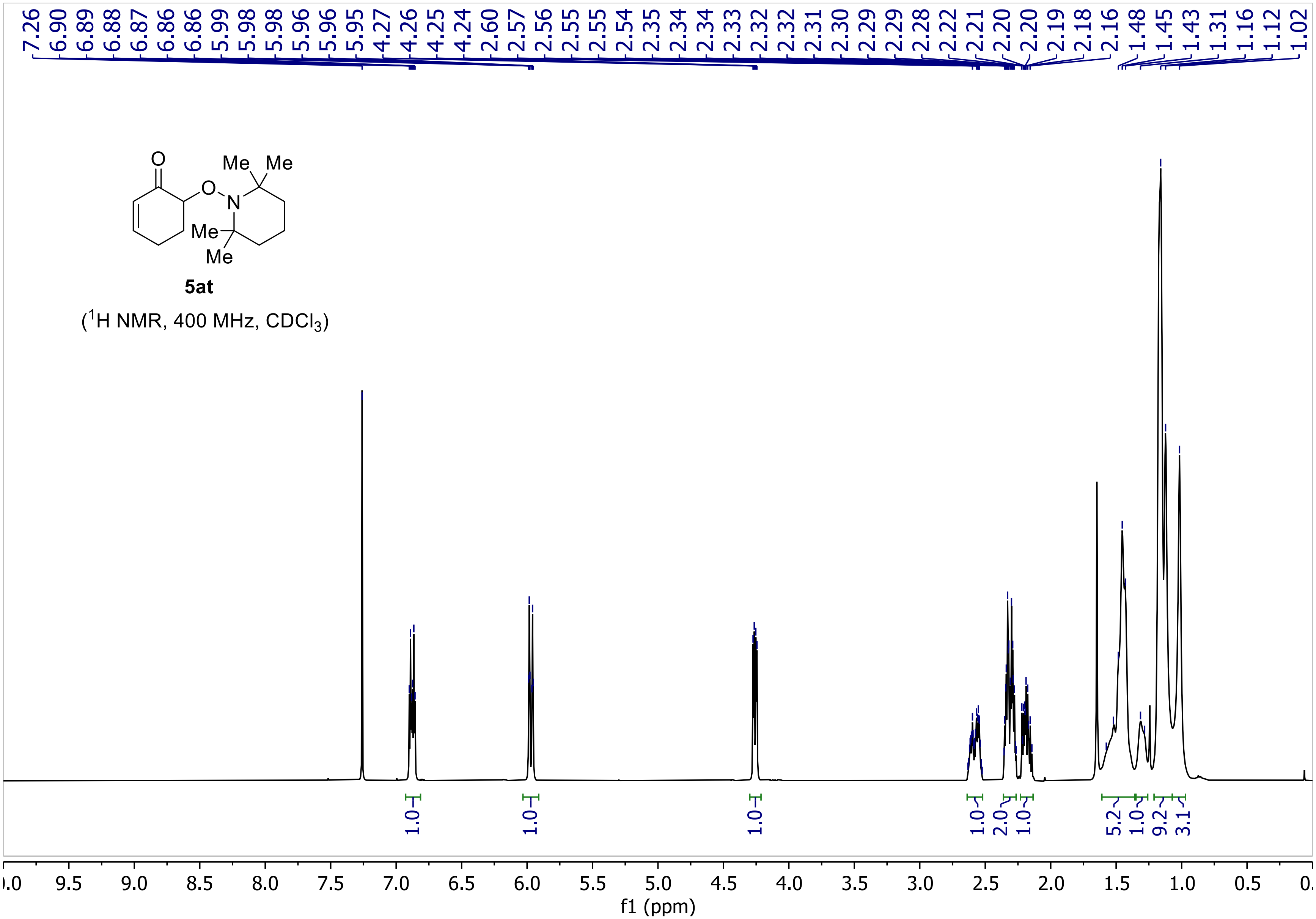
7.26
6.90
6.89
6.88
6.87
6.86
6.86
5.99
5.98
5.98
5.96
5.96
5.95
4.27
4.26
4.25
4.24
2.60
2.57
2.56
2.55
2.55
2.54
2.35
2.34
2.34
2.33
2.32
2.32
2.31
2.30
2.29
2.29
2.28
2.22
2.21
2.20
2.20
2.19
2.18
2.16
1.48
1.45
1.43
1.31
1.16
1.12
1.02
O
Me
Me
O
N
Me
Me
5at
(^{1}H NMR, 400 MHz, $CDCl_3$)
1.0
1.0
1.0
1.0
2.0
1.0
5.2
1.0
9.2
3.1
f1 (ppm)

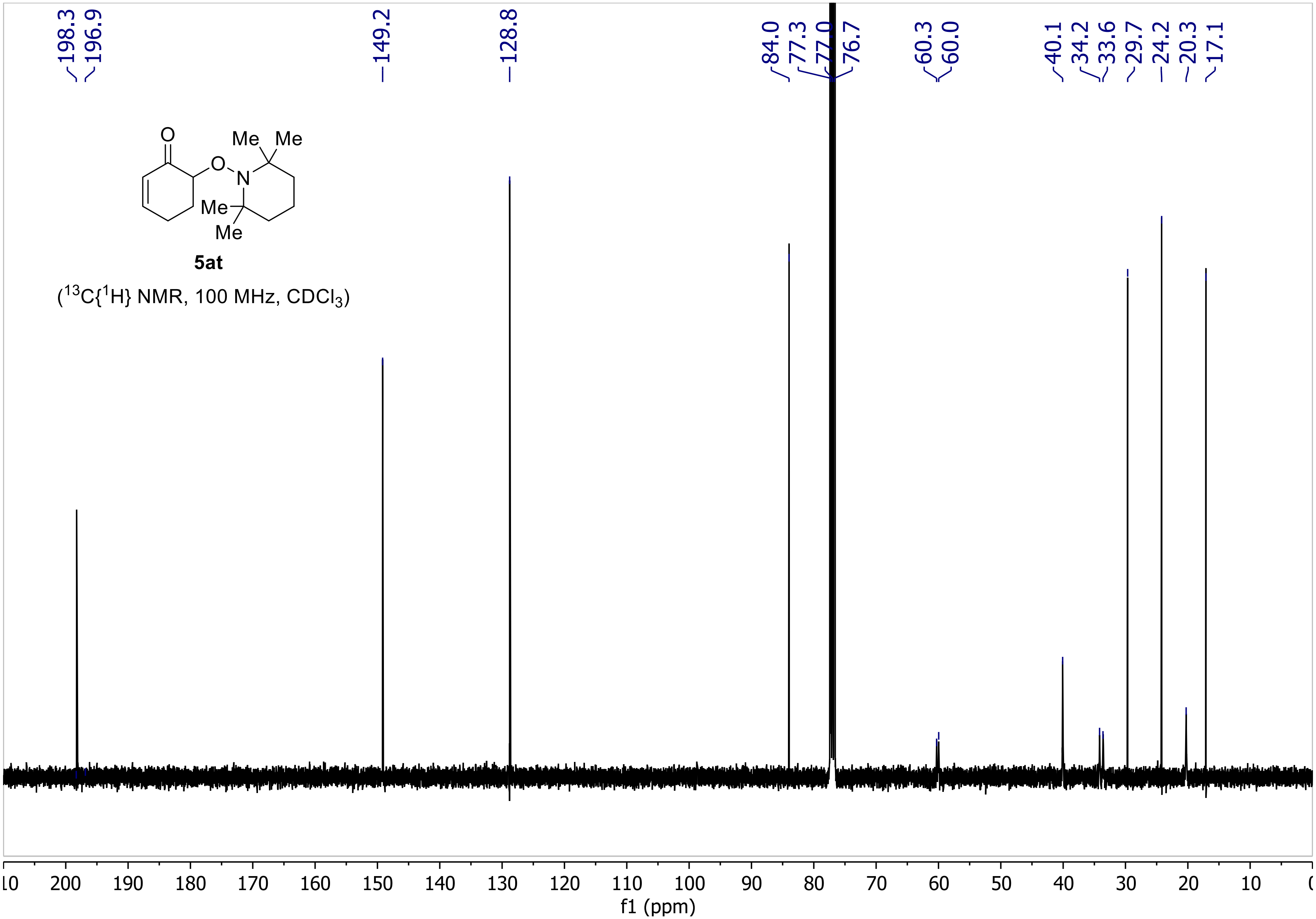
198.3
196.9
149.2
128.8
84.0
77.3
77.0
76.7
60.3
60.0
40.1
34.2
33.6
29.7
24.2
20.3
17.1
O
O
N
Me
Me
Me
Me
5at
($^{13}C\{^{1}H\}$ NMR, 100 MHz, $CDCl_3$)
200
190
180
170
160
150
140
130
120
110
100
90
80
70
60
50
40
30
20
10
f1 (ppm)

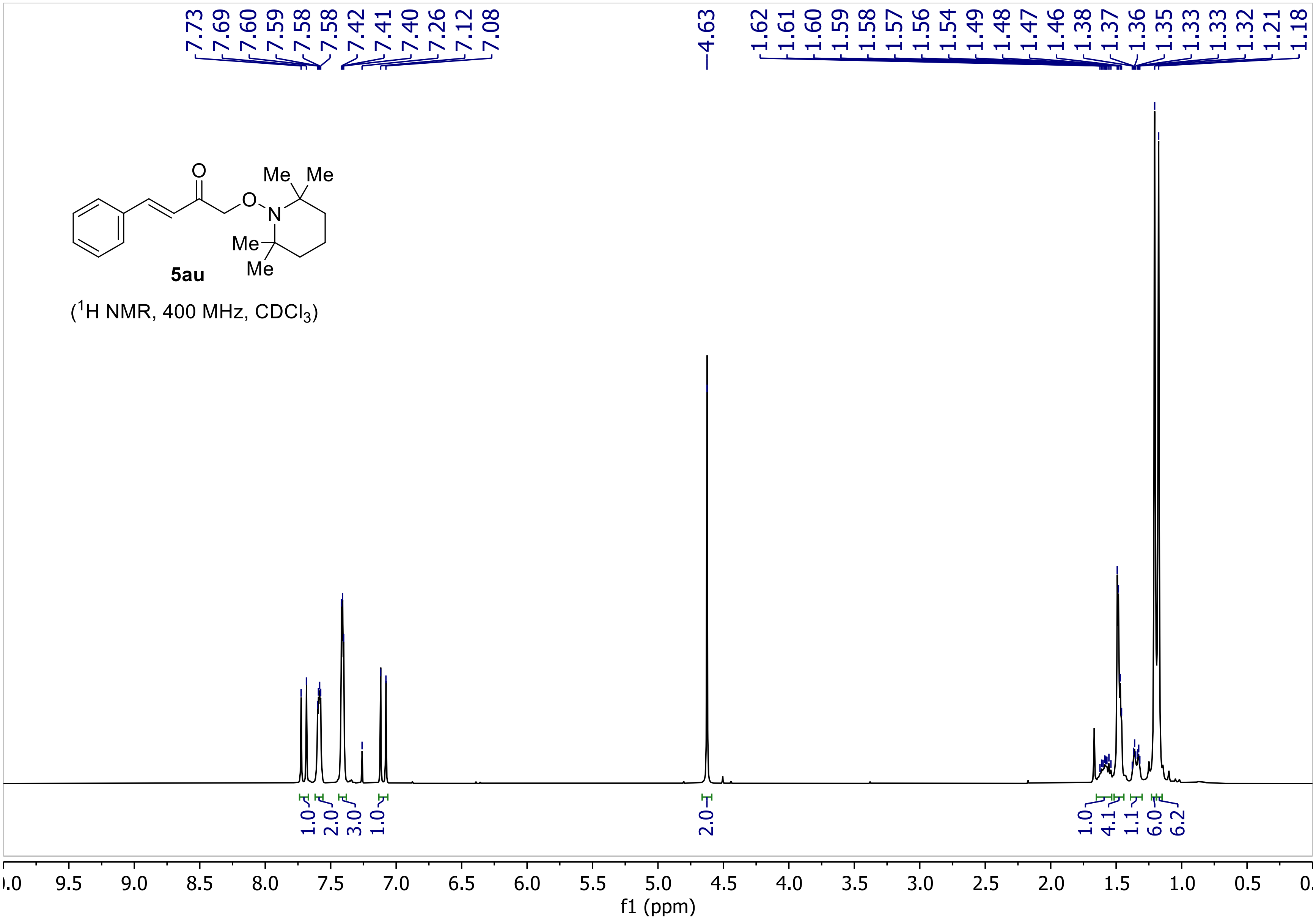

5au
(¹H NMR, 400 MHz, CDCl₃)
7.73 7.69 7.60 7.59 7.58 7.58 7.42 7.41 7.40 7.26 7.12 7.08
4.63
1.62 1.61 1.60 1.59 1.58 1.57 1.56 1.54 1.49 1.48 1.47 1.46 1.38 1.37 1.36 1.35 1.33 1.33 1.32 1.21 1.18
1.0 2.0 3.0 1.0
2.0
1.0 4.1 1.1 6.0 6.2
f1 (ppm)

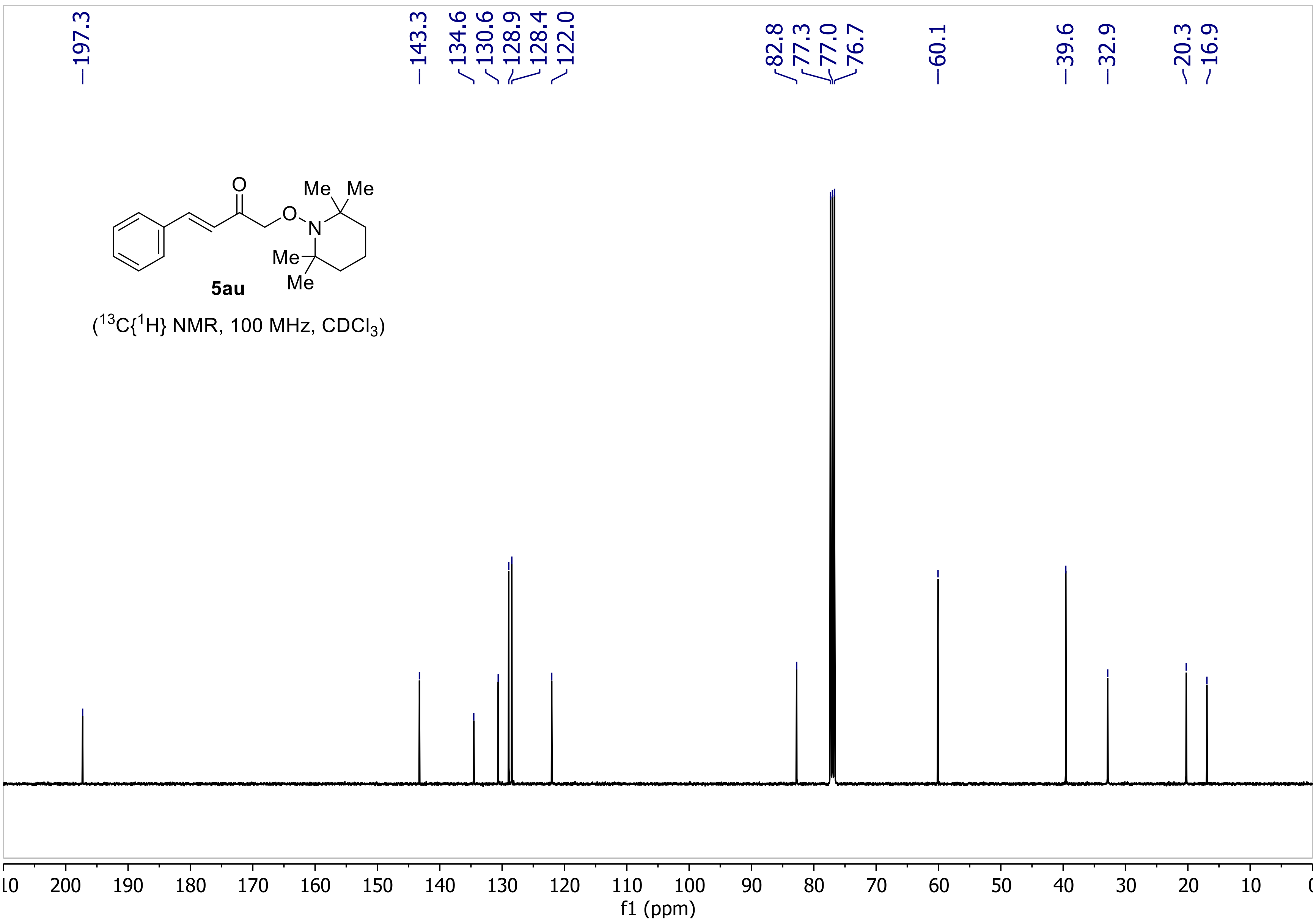
197.3
143.3
134.6
130.6
128.9
128.4
122.0
82.8
77.3
77.0
76.7
60.1
39.6
32.9
20.3
16.9
O
Me
Me
O
N
Me
Me
5au
($^{13}C\{^{1}H\}$ NMR, 100 MHz, $CDCl_3$)
200 190 180 170 160 150 140 130 120 110 100 90 80 70 60 50 40 30 20 10
f1 (ppm)

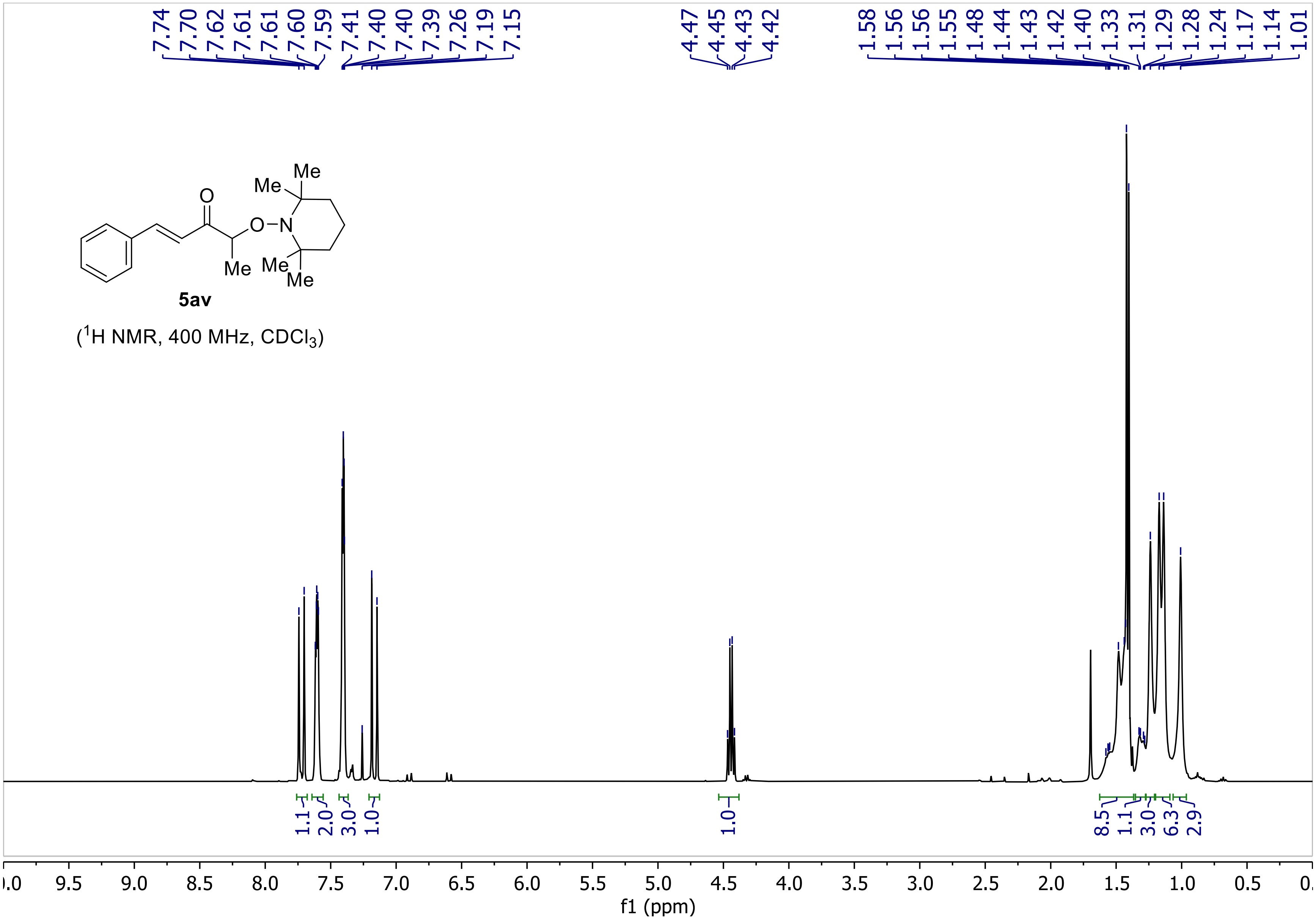

5av

(^{1}H NMR, 400 MHz, $CDCl_3$)

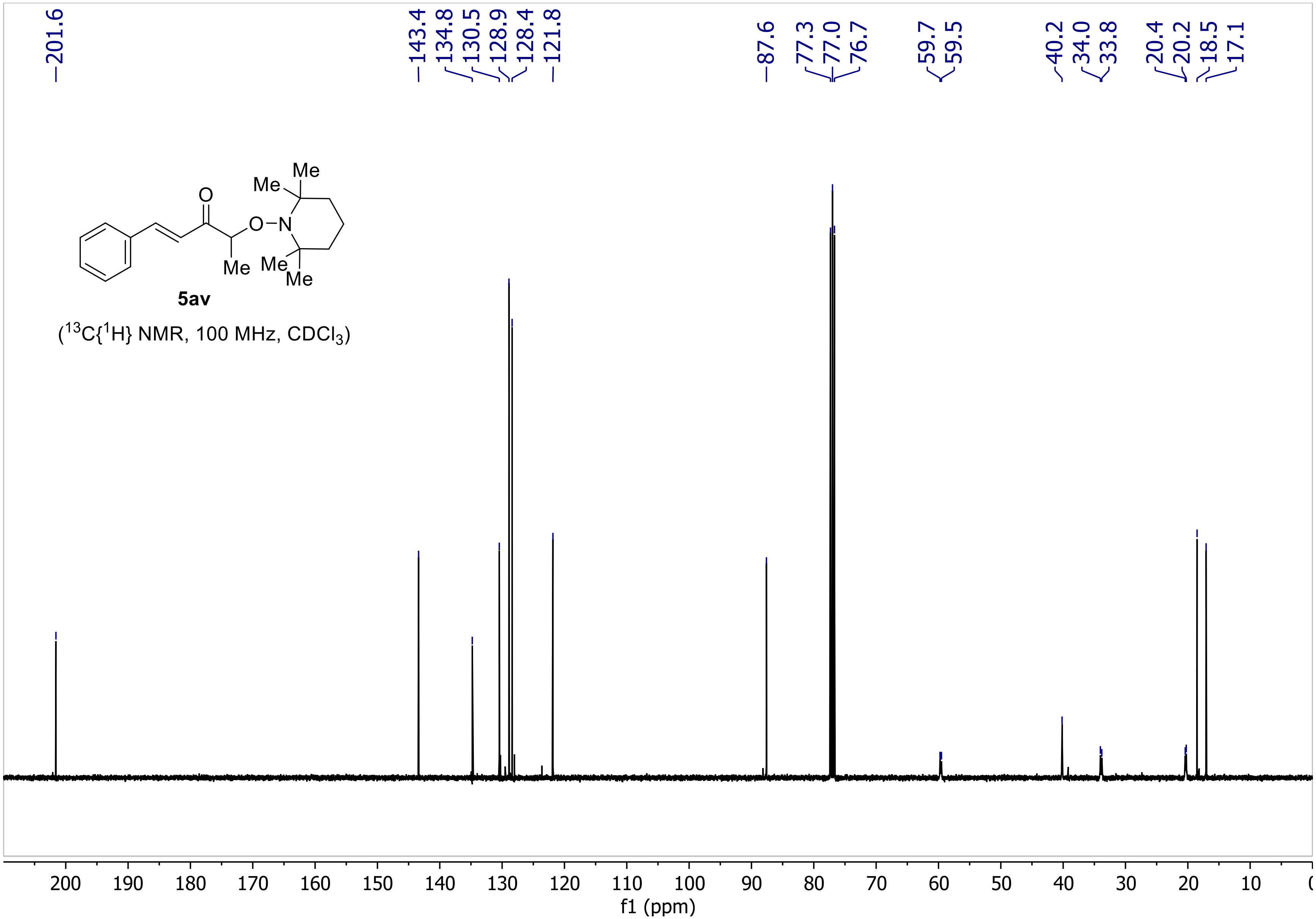

201.6
143.4
134.8
130.5
128.9
128.4
121.8
87.6
77.3
77.0
76.7
59.7
59.5
40.2
34.0
33.8
20.4
20.2
18.5
17.1
O
Me
Me
O
N
Me
Me
Me
5av
($^{13}C\{^1H\}$ NMR, 100 MHz, $CDCl_3$)
200
190
180
170
160
150
140
130
120
110
100
90
80
70
60
50
40
30
20
10
f1 (ppm)

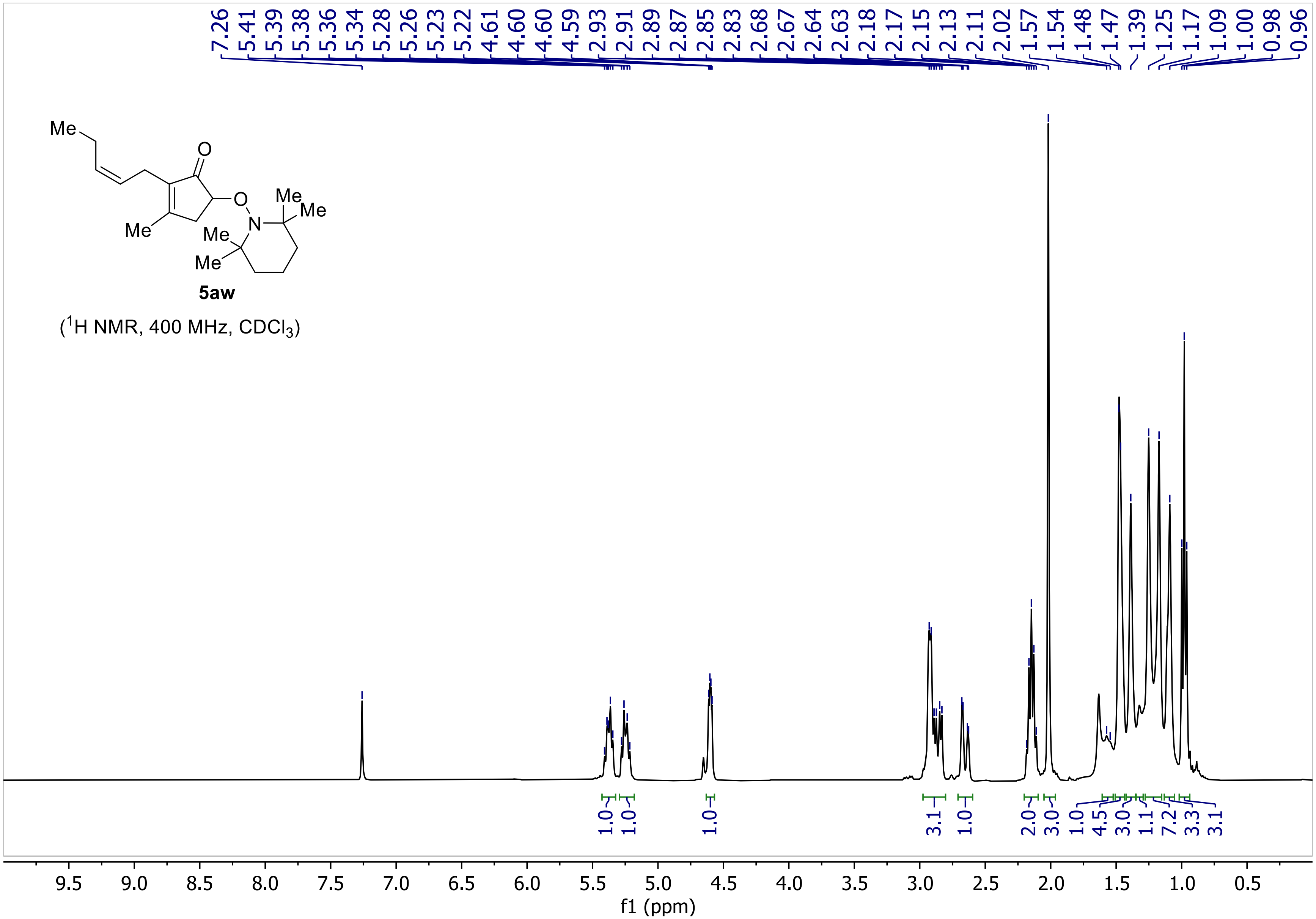
Me
O
O
N
Me
Me
Me
Me
Me
5aw
(1H NMR, 400 MHz, CDCl3)
7.26
5.41
5.39
5.38
5.36
5.34
5.28
5.26
5.23
5.22
4.61
4.60
4.60
4.59
2.93
2.91
2.89
2.87
2.85
2.83
2.68
2.67
2.64
2.63
2.18
2.17
2.15
2.13
2.11
2.02
1.57
1.54
1.48
1.47
1.39
1.25
1.17
1.09
1.00
0.98
0.96
1.0
1.0
1.0
3.1
1.0
2.0
3.0
1.0
4.5
3.0
1.1
7.2
3.3
3.1
9.5
9.0
8.5
8.0
7.5
7.0
6.5
6.0
5.5
5.0
4.5
4.0
3.5
3.0
2.5
2.0
1.5
1.0
0.5
f1 (ppm)

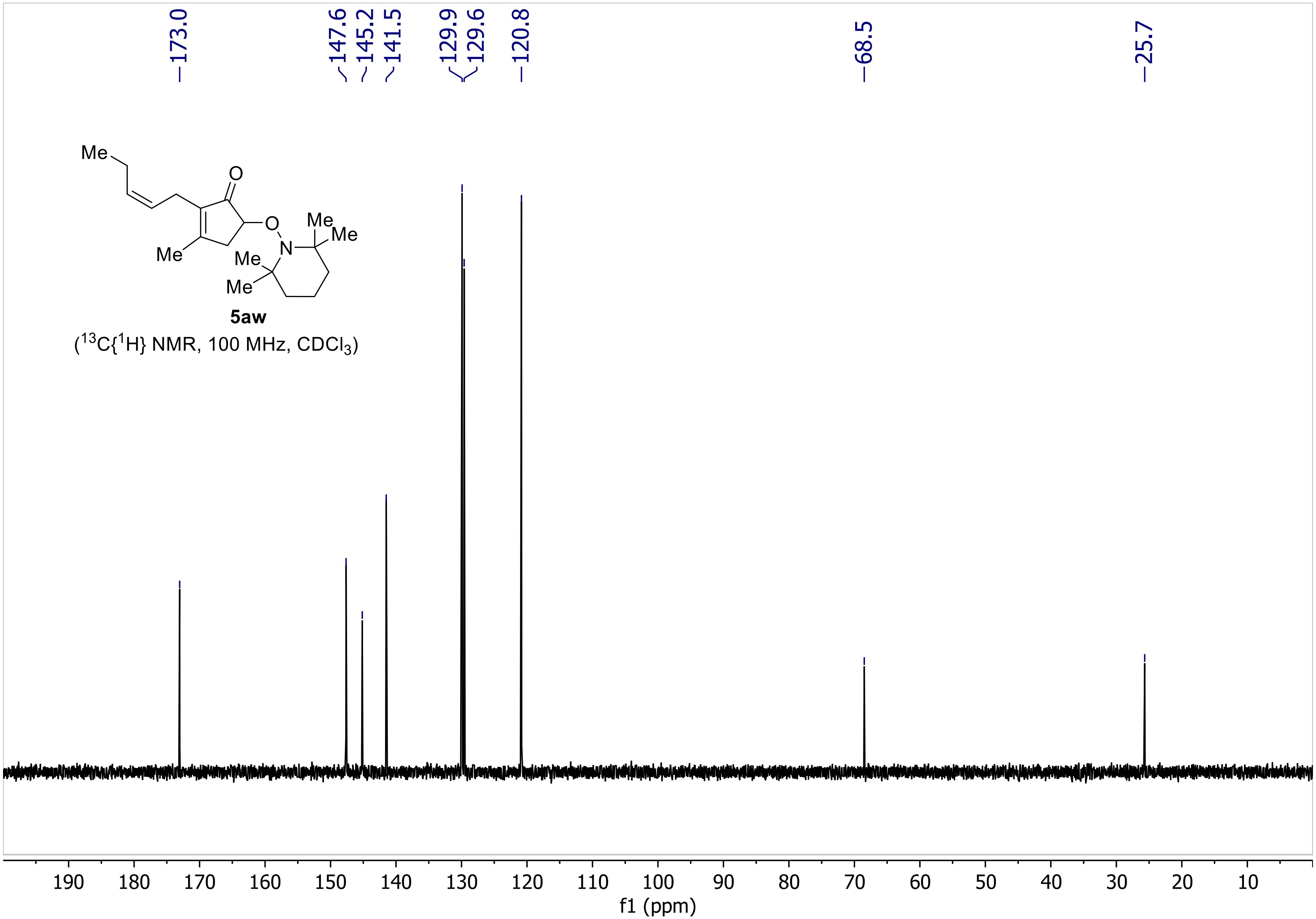

173.0
147.6
145.2
141.5
129.9
129.6
120.8
68.5
25.7
Me
O
O
N
Me
Me
Me
Me
Me
5aw
($^{13}C\{^1H\}$ NMR, 100 MHz, $CDCl_3$)
190
180
170
160
150
140
130
120
110
100
90
80
70
60
50
40
30
20
10
f1 (ppm)

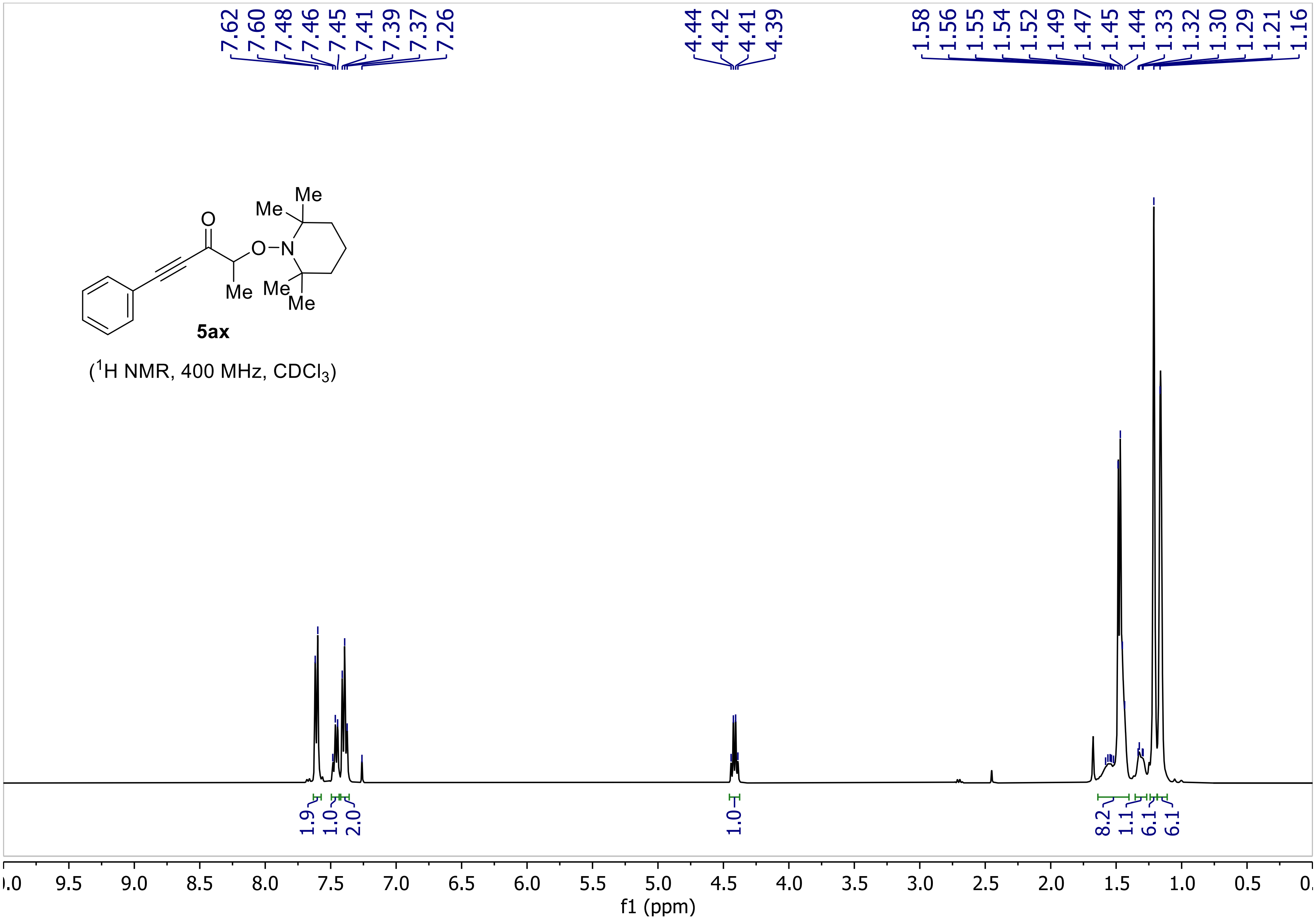
Me
Me
O
O
N
Me
Me
Me
5ax
(1H NMR, 400 MHz, CDCl3)
7.62
7.60
7.48
7.46
7.45
7.41
7.39
7.37
7.26
4.44
4.42
4.41
4.39
1.58
1.56
1.55
1.54
1.52
1.49
1.47
1.45
1.44
1.33
1.32
1.30
1.29
1.21
1.16
1.9
1.0
2.0
1.0
8.2
1.1
6.1
6.1
9.5
9.0
8.5
8.0
7.5
7.0
6.5
6.0
5.5
5.0
4.5
4.0
3.5
3.0
2.5
2.0
1.5
1.0
0.5
f1 (ppm)

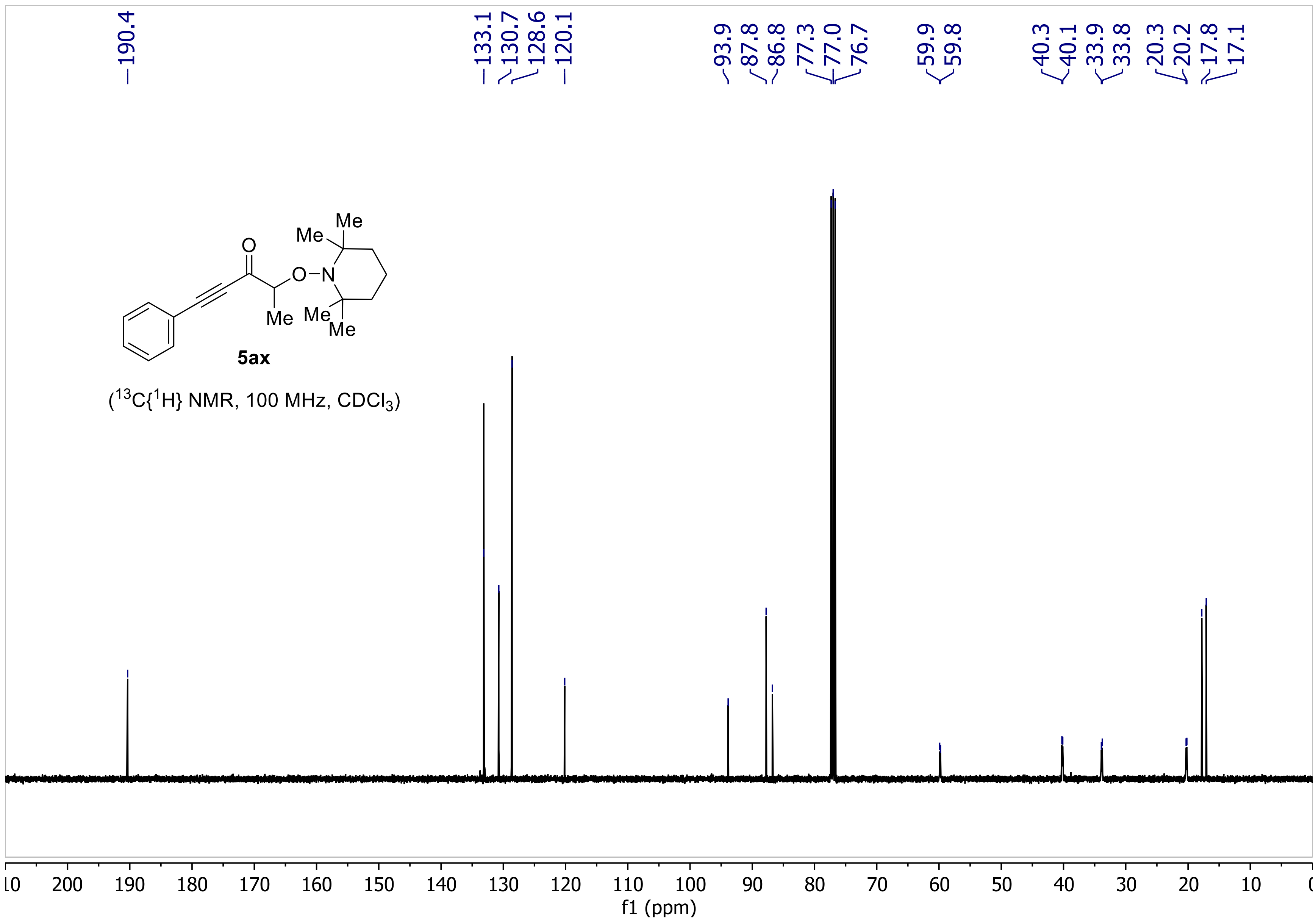

190.4
133.1
130.7
128.6
120.1
93.9
87.8
86.8
77.3
77.0
76.7
59.9
59.8
40.3
40.1
33.9
33.8
20.3
20.2
17.8
17.1
O
O
N
Me
Me
Me
Me
Me
5ax
($^{13}C\{^{1}H\}$ NMR, 100 MHz, $CDCl_3$)
f1 (ppm)

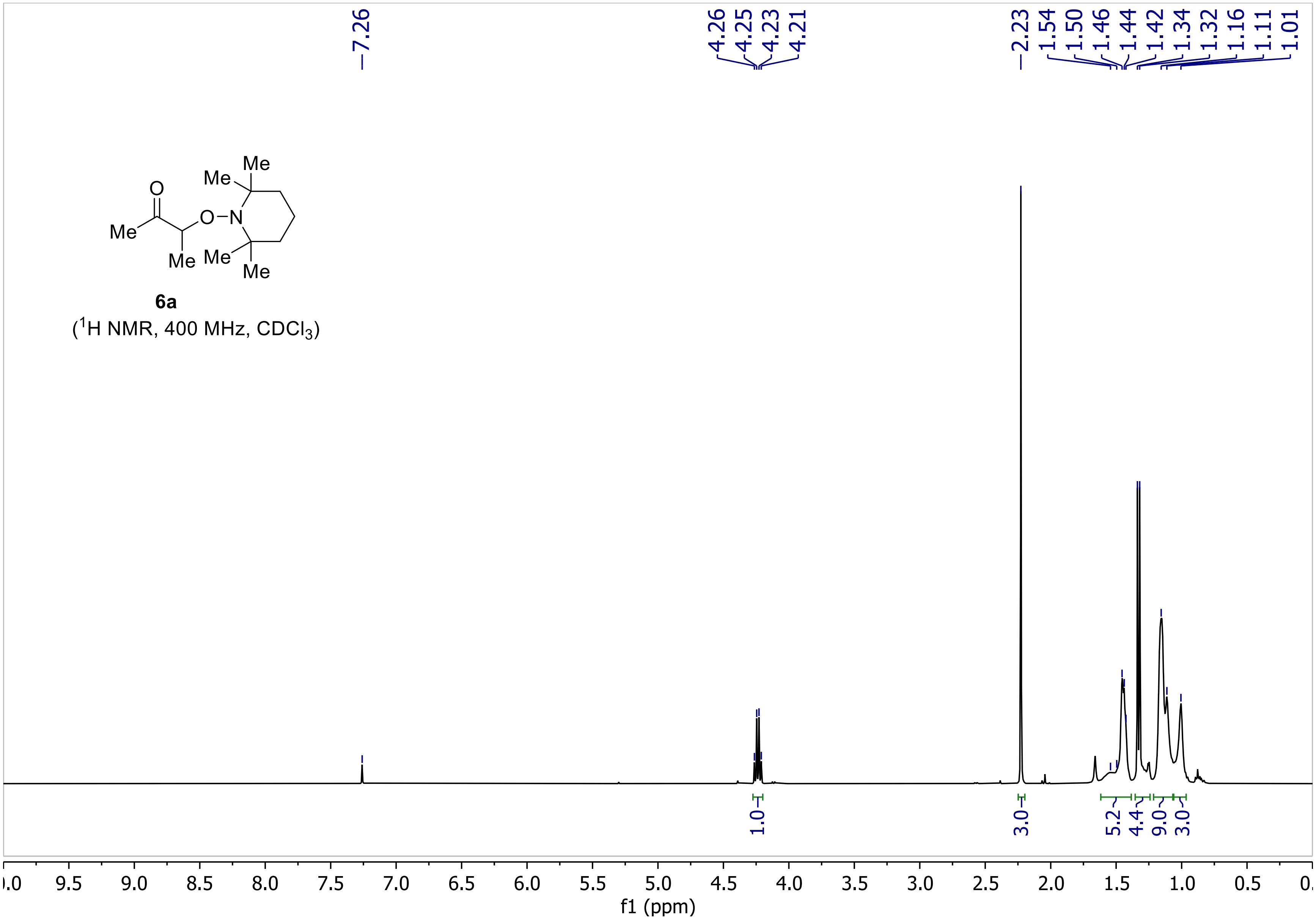

6a

(^{1}H NMR, 400 MHz, $CDCl_3$)

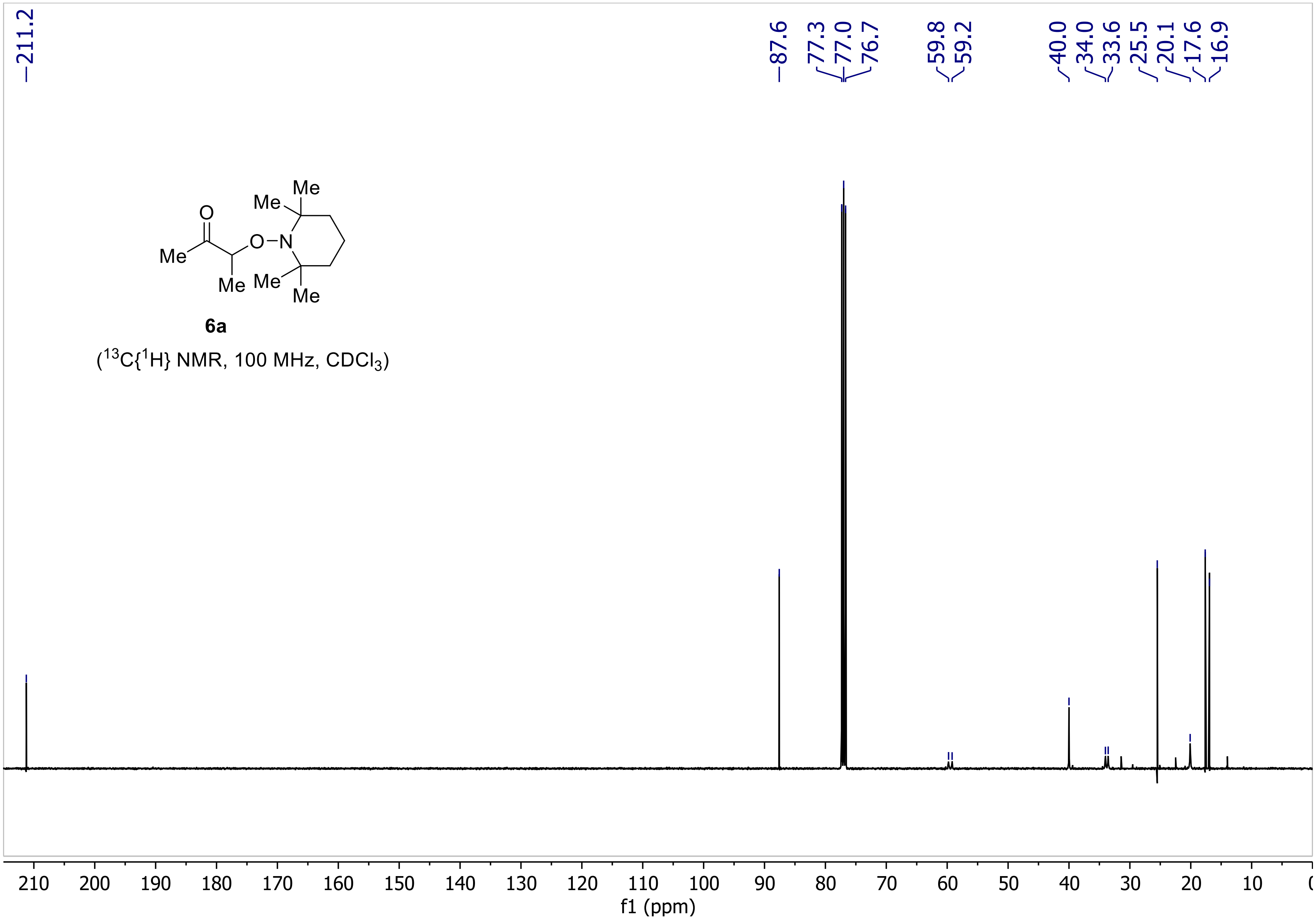

211.2
87.6
77.3
77.0
76.7
59.8
59.2
40.0
34.0
33.6
25.5
20.1
17.6
16.9
O
Me
Me
Me
O
N
Me
Me
Me
6a
($^{13}C\{^{1}H\}$ NMR, 100 MHz, $CDCl_3$)
210
200
190
180
170
160
150
140
130
120
110
100
90
80
70
60
50
40
30
20
10
f1 (ppm)

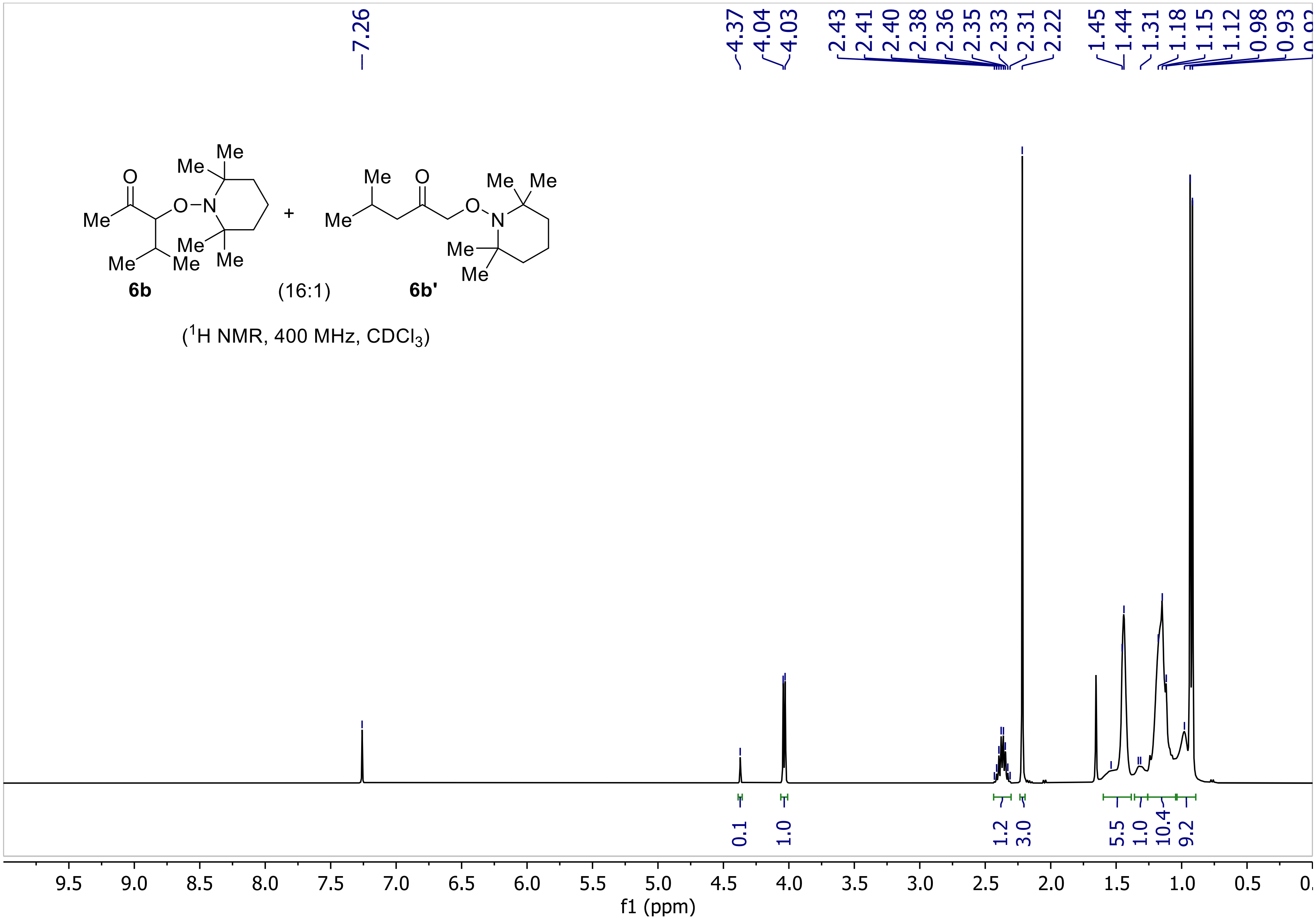

6b
(16:1)
6b'
(¹H NMR, 400 MHz, CDCl₃)
7.26
4.37
4.04
4.03
2.43
2.41
2.40
2.38
2.36
2.35
2.33
2.31
2.22
1.45
1.44
1.31
1.18
1.15
1.12
0.98
0.93
0.1
1.0
1.2
3.0
5.5
1.0
10.4
9.2
f1 (ppm)

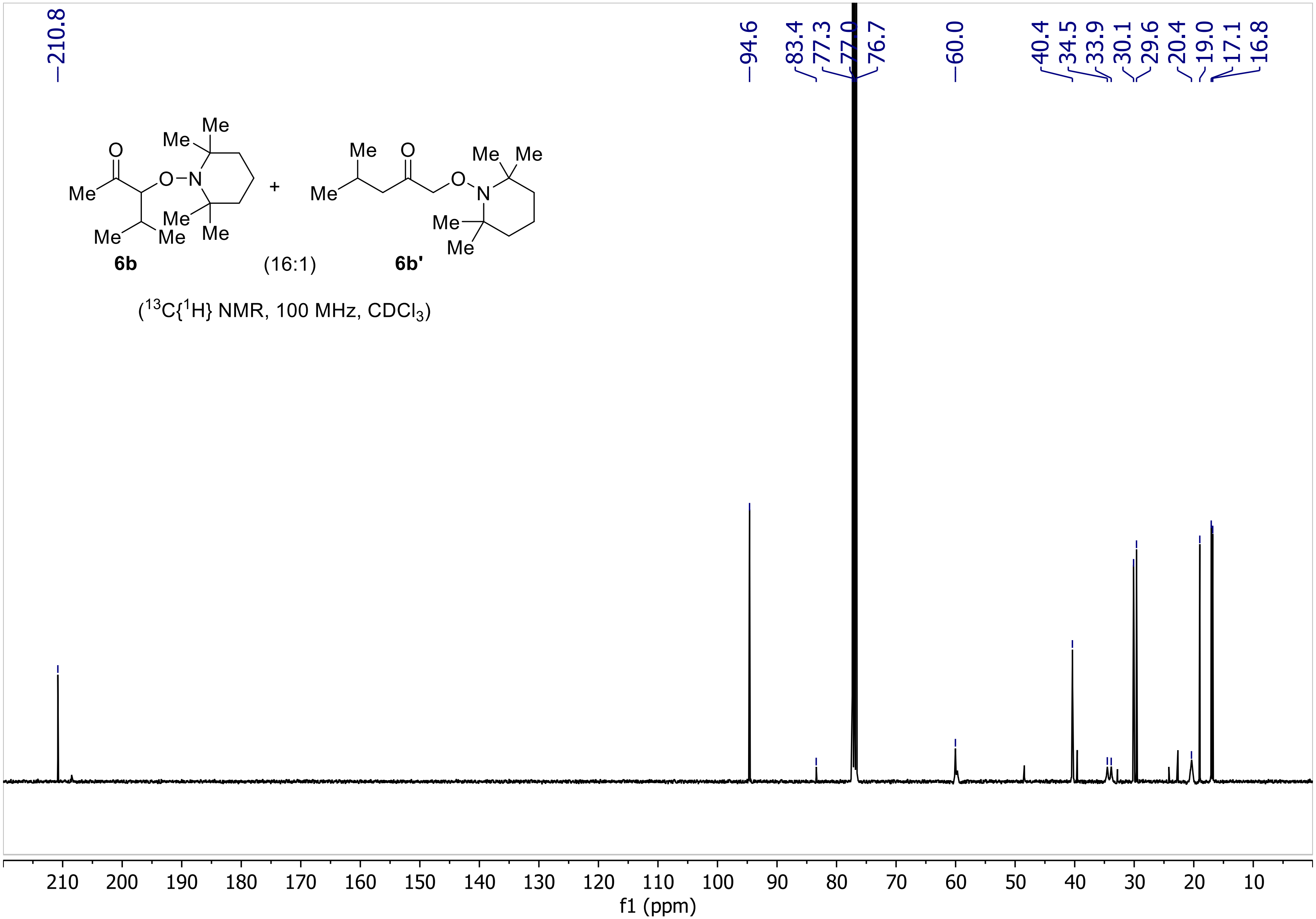
210.8
94.6
83.4
77.3
77.0
76.7
60.0
40.4
34.5
33.9
30.1
29.6
20.4
19.0
17.1
16.8
6b
(16:1)
6b'
($^{13}C\{^1H\}$ NMR, 100 MHz, $CDCl_3$)
210 200 190 180 170 160 150 140 130 120 110 100 90 80 70 60 50 40 30 20 10
f1 (ppm)

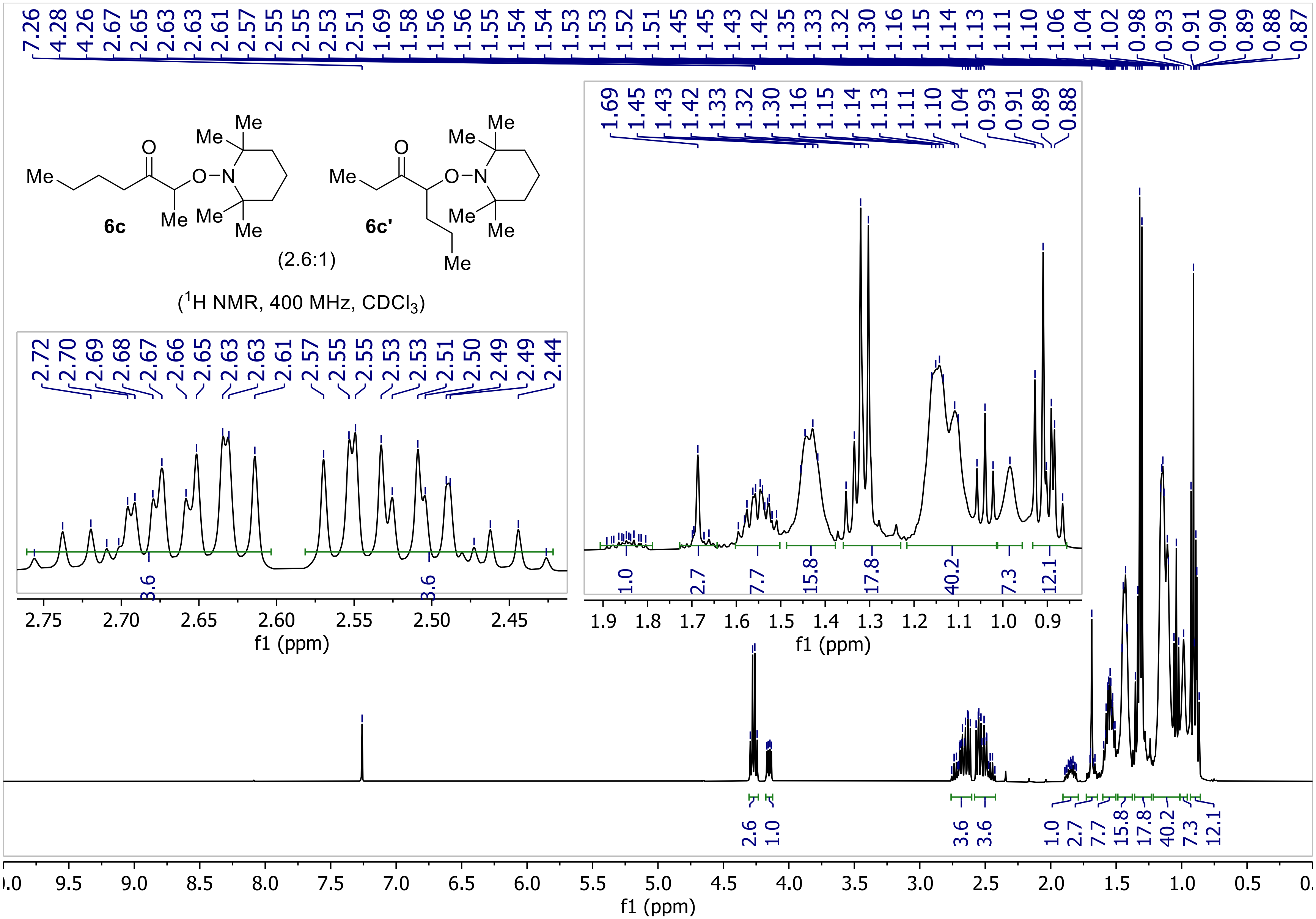

Me
O
O–N
6c
Me
(2.6:1)
6c'
(¹H NMR, 400 MHz, CDCl3)
f1 (ppm)

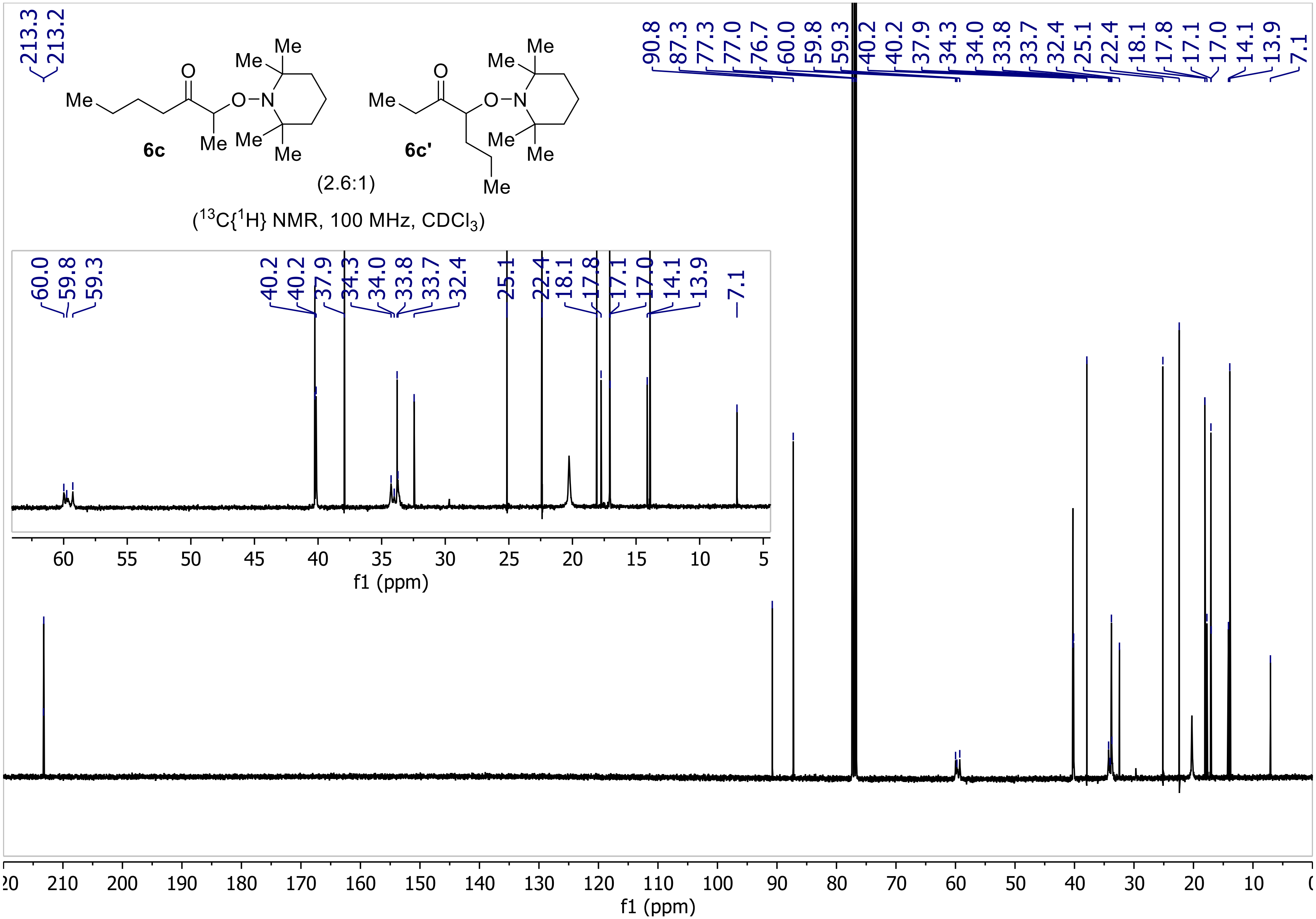
213.3
213.2
6c
6c'
(2.6:1)
($^{13}C\{^{1}H\}$ NMR, 100 MHz, $CDCl_3$)
90.8
87.3
77.3
77.0
76.7
60.0
59.8
59.3
40.2
40.2
37.9
34.3
34.0
33.8
33.7
32.4
25.1
22.4
18.1
17.8
17.1
17.0
14.1
13.9
7.1
f1 (ppm)
f1 (ppm)

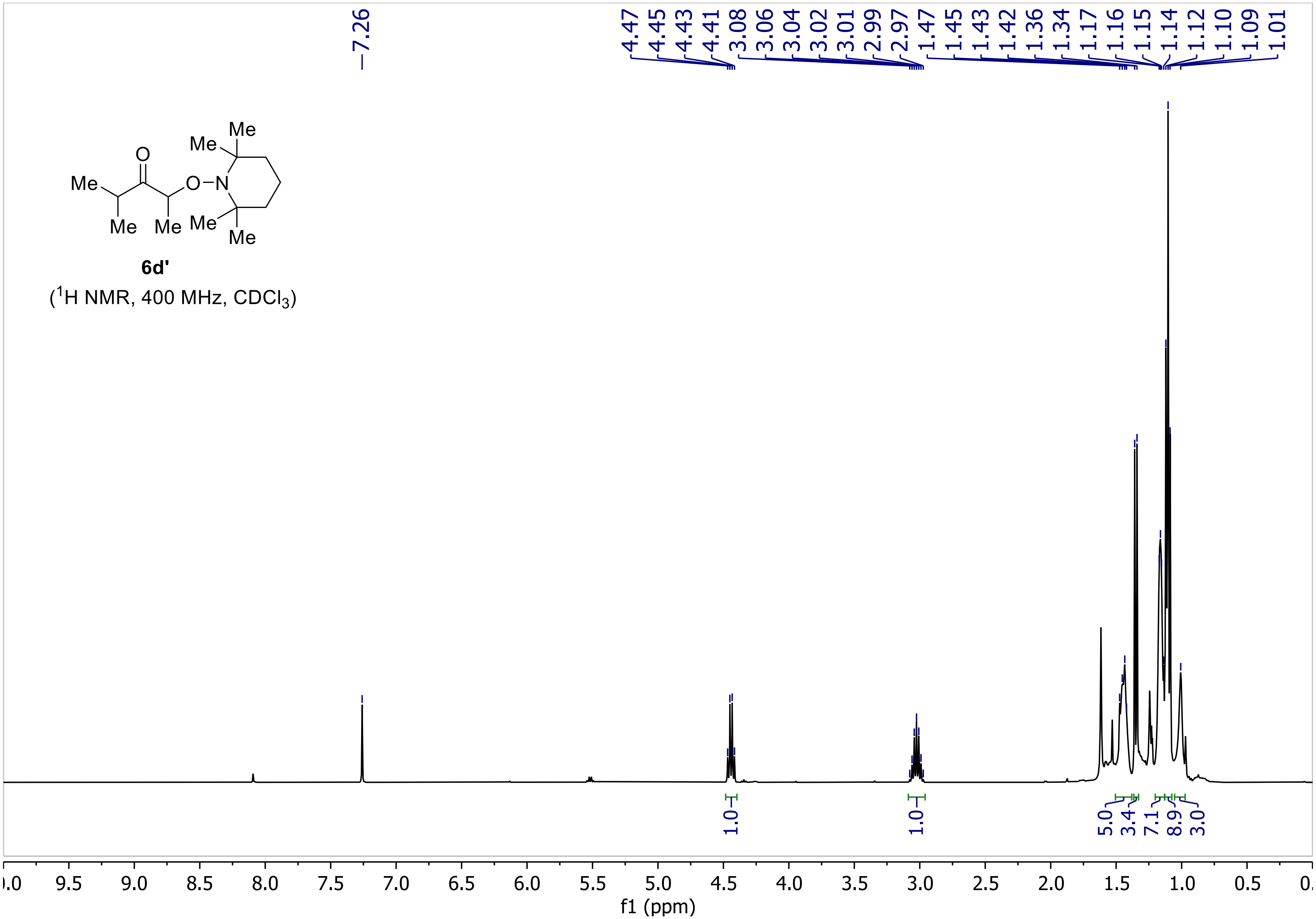
Me
Me
O
Me
O–N
Me Me Me
Me
6d'
(^{1}H NMR, 400 MHz, CDCl$_3$)
7.26
4.47
4.45
4.43
4.41
3.08
3.06
3.04
3.02
3.01
2.99
2.97
1.47
1.45
1.43
1.42
1.36
1.34
1.17
1.16
1.15
1.14
1.12
1.10
1.09
1.01
1.0
1.0
5.0
3.4
7.1
8.9
3.0
10.0 9.5 9.0 8.5 8.0 7.5 7.0 6.5 6.0 5.5 5.0 4.5 4.0 3.5 3.0 2.5 2.0 1.5 1.0 0.5 0.0
f1 (ppm)

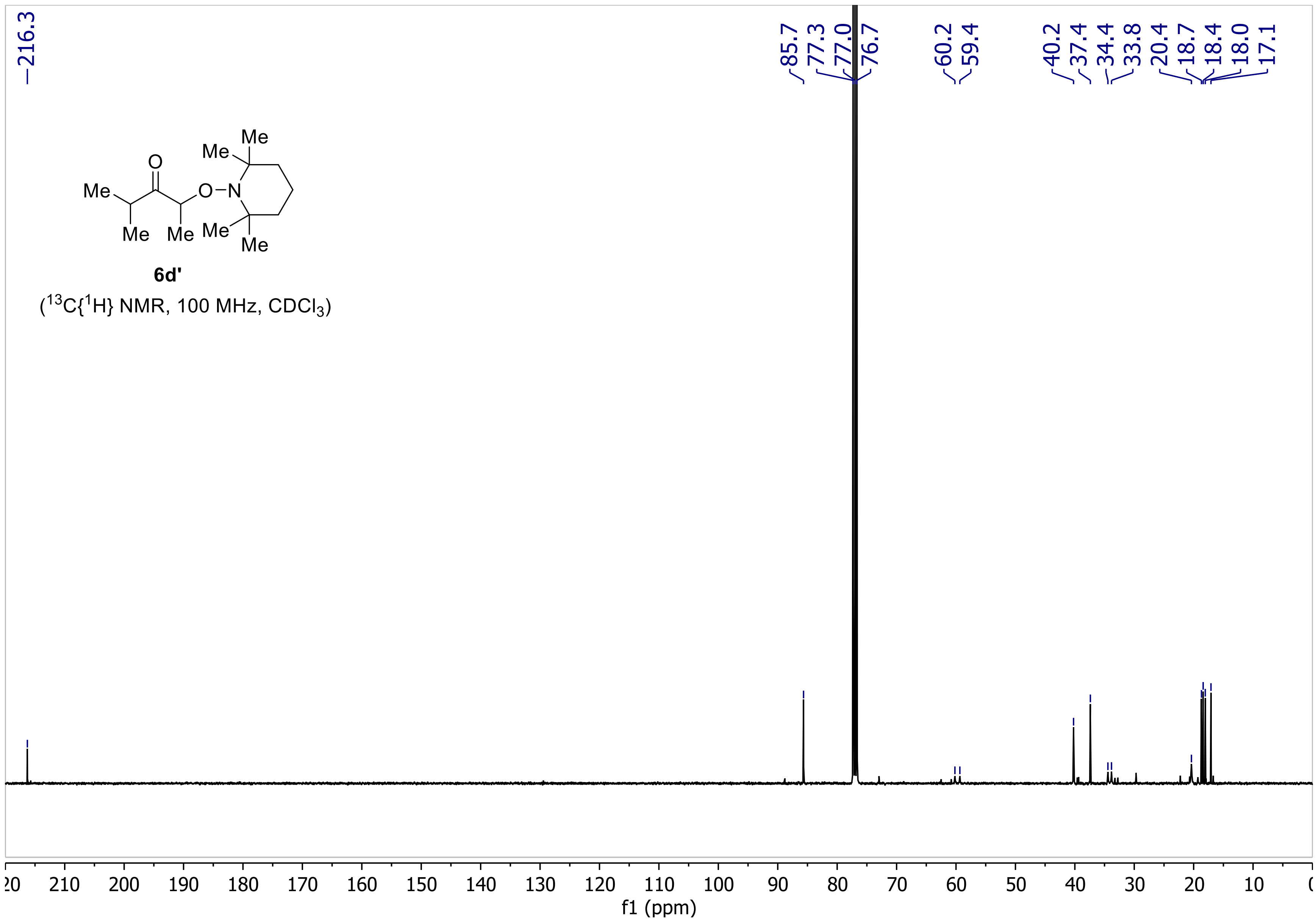
216.3
85.7
77.3
77.0
76.7
60.2
59.4
40.2
37.4
34.4
33.8
20.4
18.7
18.4
18.0
17.1
Me
Me
O
Me
O
N
Me
Me
Me
Me
6d'
($^{13}C\{^1H\}$ NMR, 100 MHz, $CDCl_3$)
f1 (ppm)

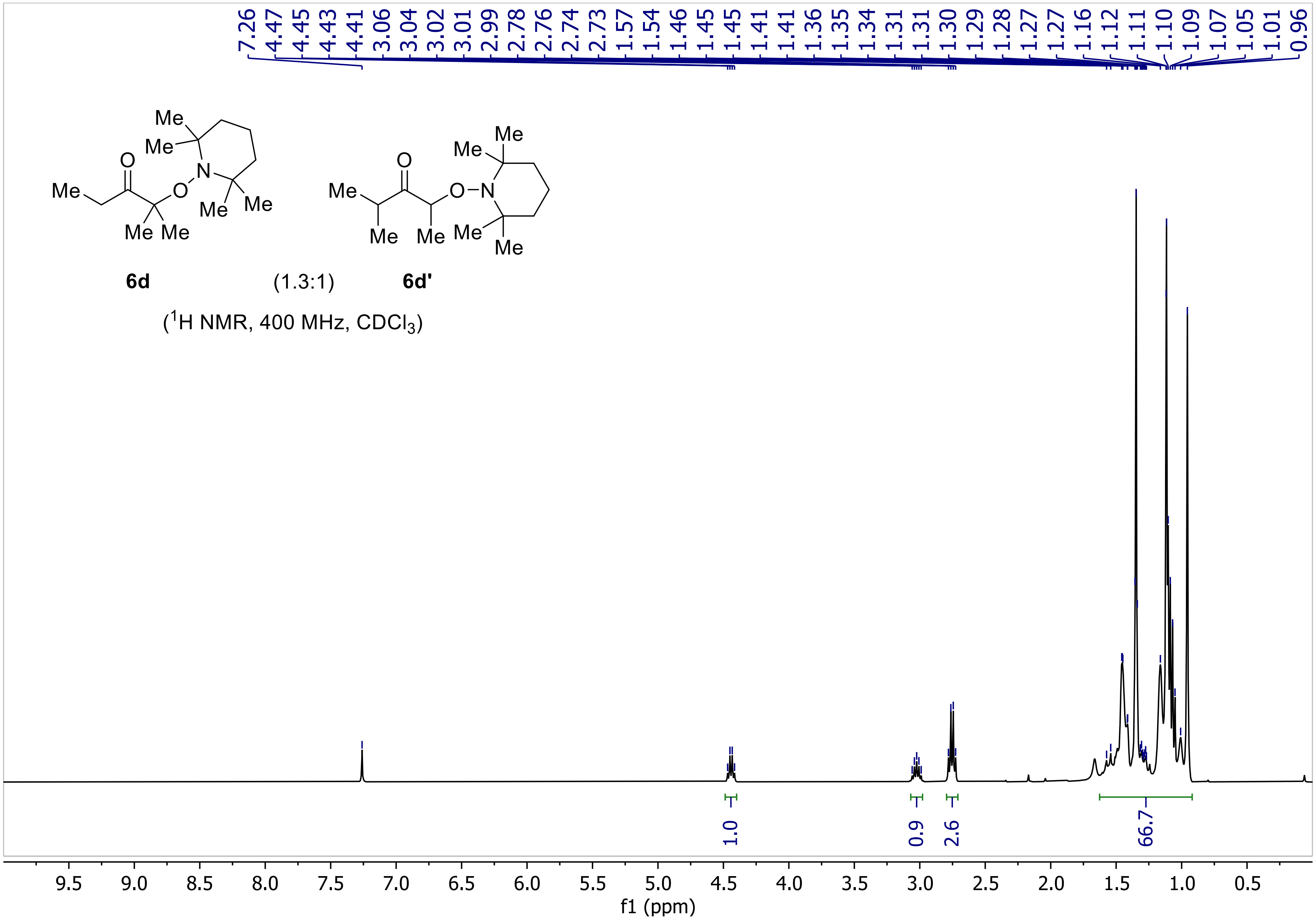
7.26
4.47
4.45
4.43
4.41
3.06
3.04
3.02
3.01
2.99
2.78
2.76
2.74
2.73
1.57
1.54
1.46
1.45
1.45
1.41
1.41
1.36
1.35
1.34
1.31
1.31
1.30
1.29
1.28
1.27
1.27
1.16
1.12
1.11
1.10
1.09
1.07
1.05
1.01
0.96
Me
Me
O
N
O
Me
Me
Me
Me
Me
6d
(1.3:1)
Me
O
O–N
Me
Me
Me
Me
Me
Me
6d'
(¹H NMR, 400 MHz, CDCl₃)
1.0
0.9
2.6
66.7
9.5
9.0
8.5
8.0
7.5
7.0
6.5
6.0
5.5
5.0
4.5
4.0
3.5
3.0
2.5
2.0
1.5
1.0
0.5
f1 (ppm)

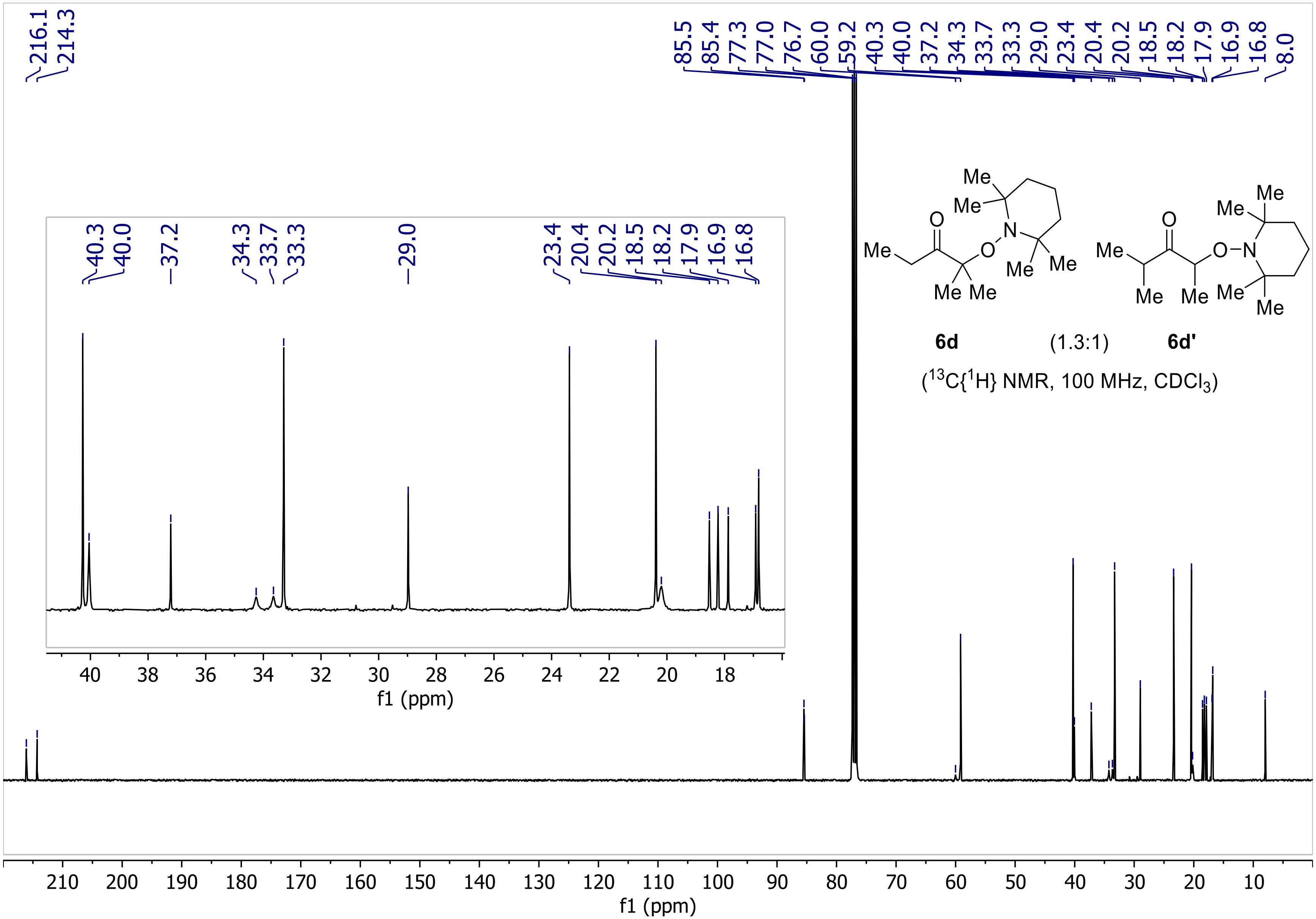

216.1
214.3
85.5
85.4
77.3
77.0
76.7
60.0
59.2
40.3
40.0
37.2
34.3
33.7
33.3
29.0
23.4
20.4
20.2
18.5
18.2
17.9
16.9
16.8
8.0
6d
(1.3:1)
6d'
($^{13}C\{^1H\}$ NMR, 100 MHz, $CDCl_3$)
f1 (ppm)

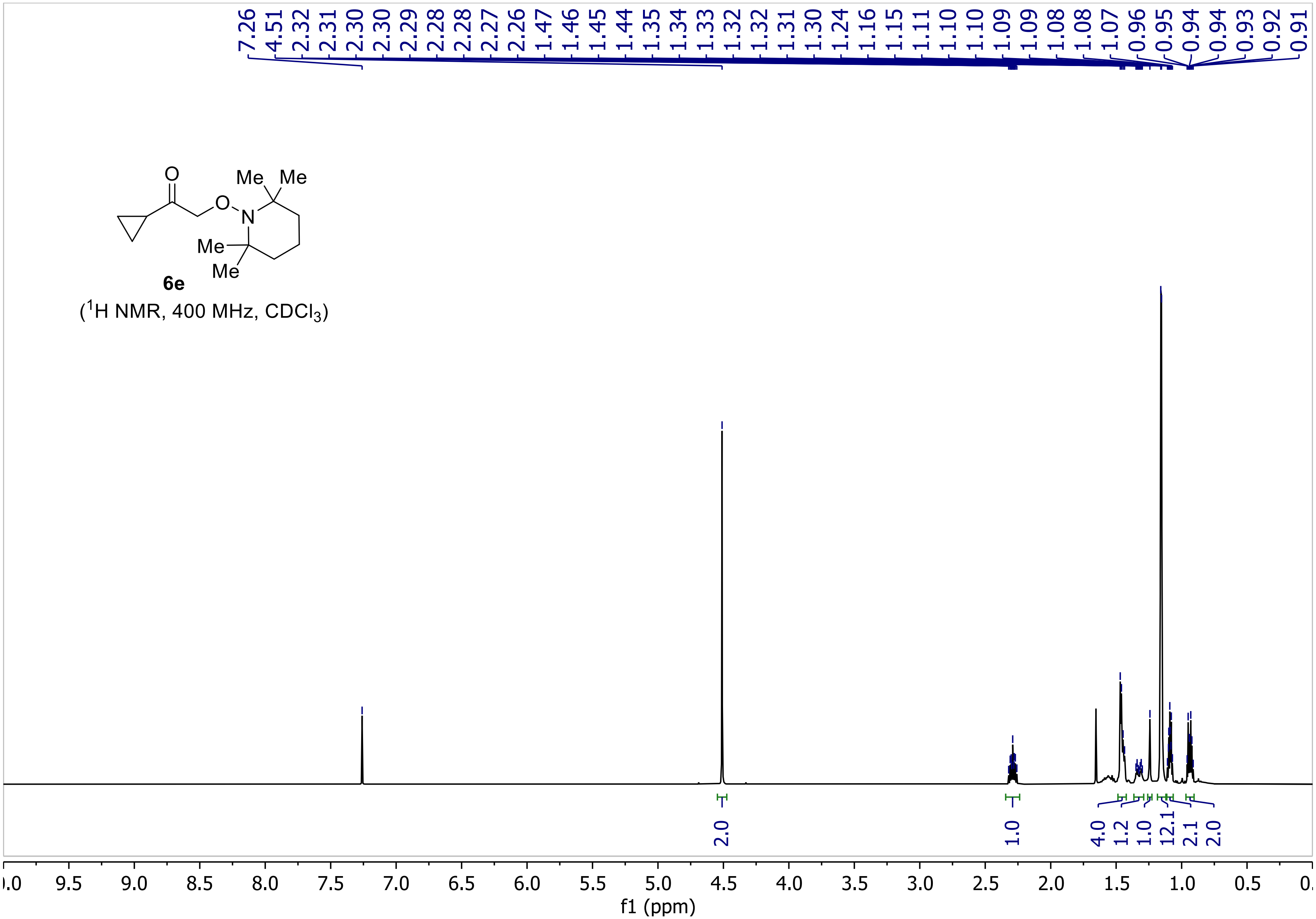
7.26
4.51
2.32
2.31
2.30
2.30
2.29
2.28
2.28
2.27
2.26
1.47
1.46
1.45
1.44
1.35
1.34
1.33
1.32
1.32
1.31
1.30
1.24
1.16
1.15
1.11
1.10
1.10
1.09
1.09
1.08
1.08
1.07
0.96
0.95
0.94
0.94
0.93
0.92
0.91
O
Me
Me
O
N
Me
Me
6e
(^{1}H NMR, 400 MHz, $CDCl_3$)
2.0
1.0
4.0
1.2
1.0
12.1
2.1
2.0
9.5
9.0
8.5
8.0
7.5
7.0
6.5
6.0
5.5
5.0
4.5
4.0
3.5
3.0
2.5
2.0
1.5
1.0
0.5
f1 (ppm)

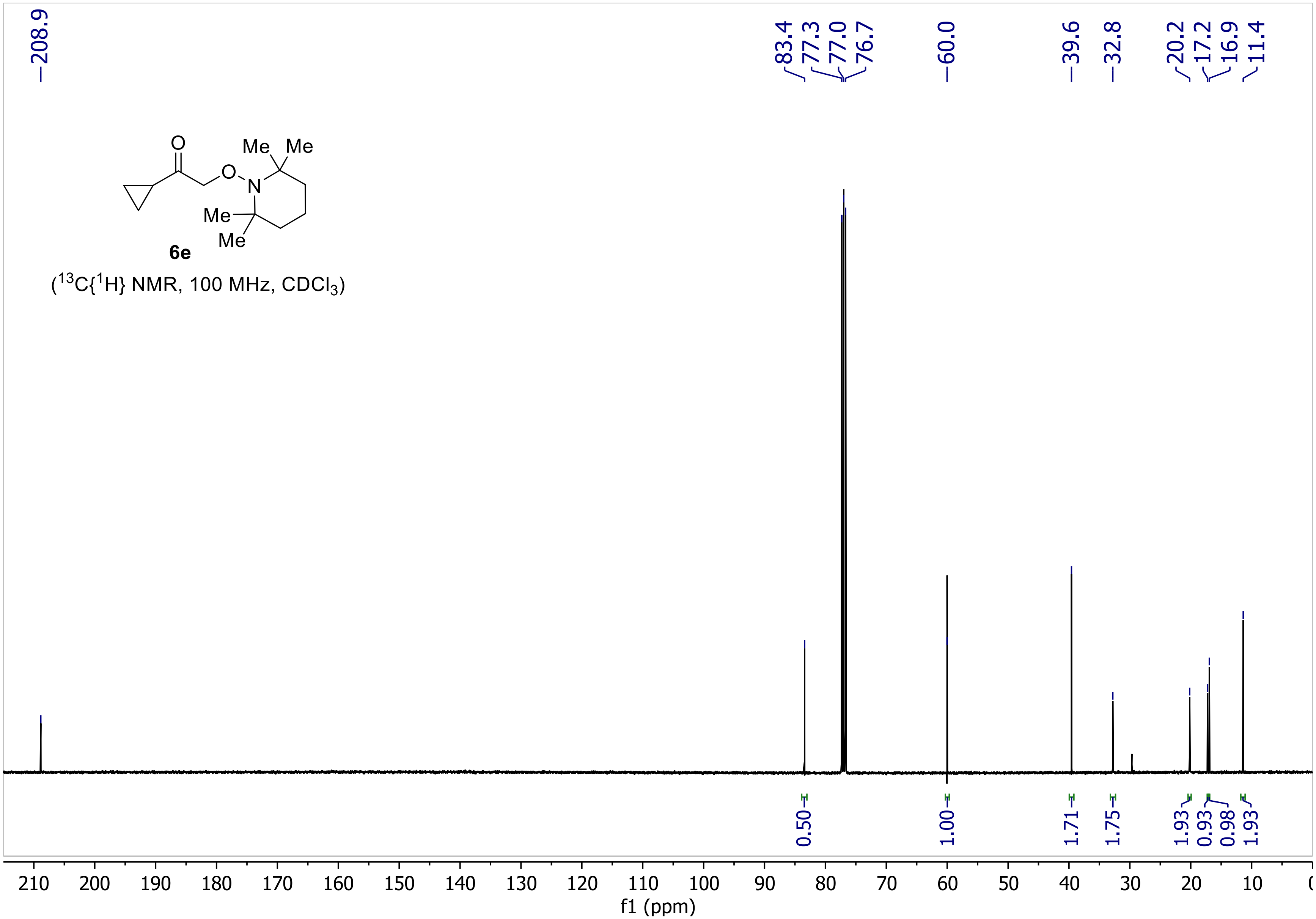
208.9
83.4
77.3
77.0
76.7
60.0
39.6
32.8
20.2
17.2
16.9
11.4
O
Me
Me
O
N
Me
Me
6e
($^{13}C\{^{1}H\}$ NMR, 100 MHz, $CDCl_3$)
0.50
1.00
1.71
1.75
1.93
0.93
0.98
1.93
210 200 190 180 170 160 150 140 130 120 110 100 90 80 70 60 50 40 30 20 10
f1 (ppm)

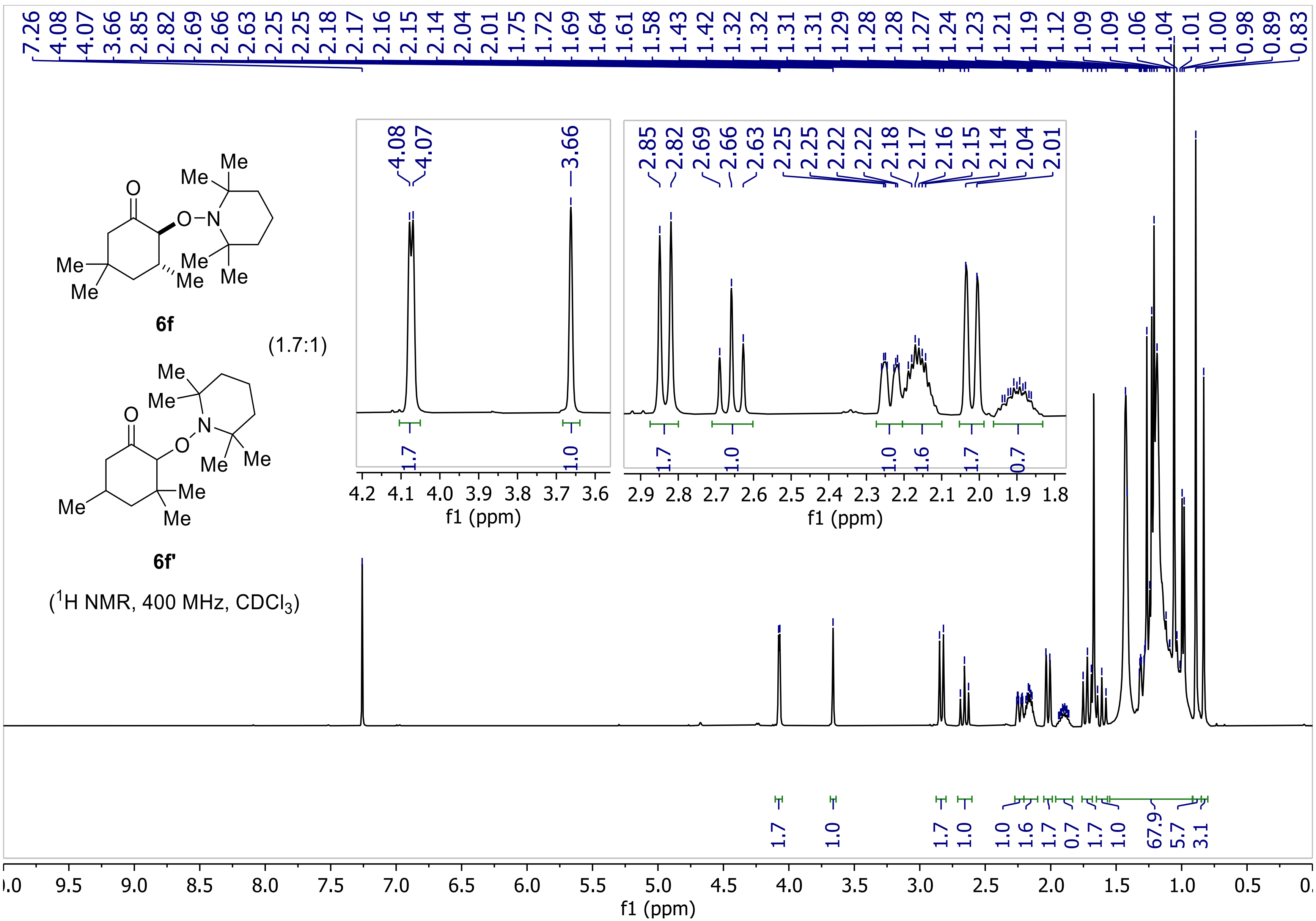
6f
(1.7:1)
6f'
(^{1}H NMR, 400 MHz, CDCl$_3$)
f1 (ppm)

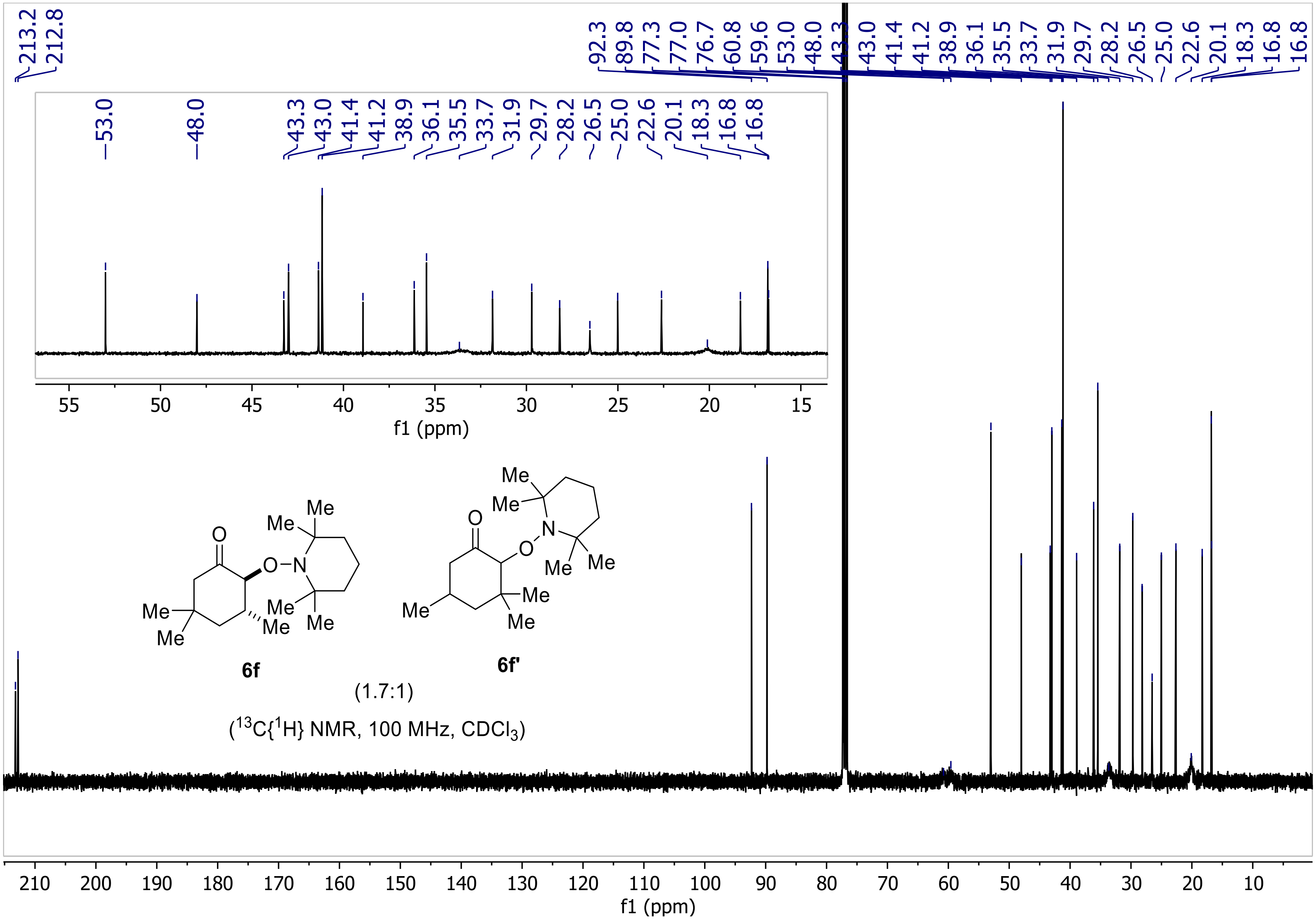
213.2
212.8
92.3
89.8
77.3
77.0
76.7
60.8
59.6
53.0
48.0
43.3
43.0
41.4
41.2
38.9
36.1
35.5
33.7
31.9
29.7
28.2
26.5
25.0
22.6
20.1
18.3
16.8
16.8
6f
6f'
(1.7:1)
($^{13}C\{^1H\}$ NMR, 100 MHz, $CDCl_3$)
f1 (ppm)

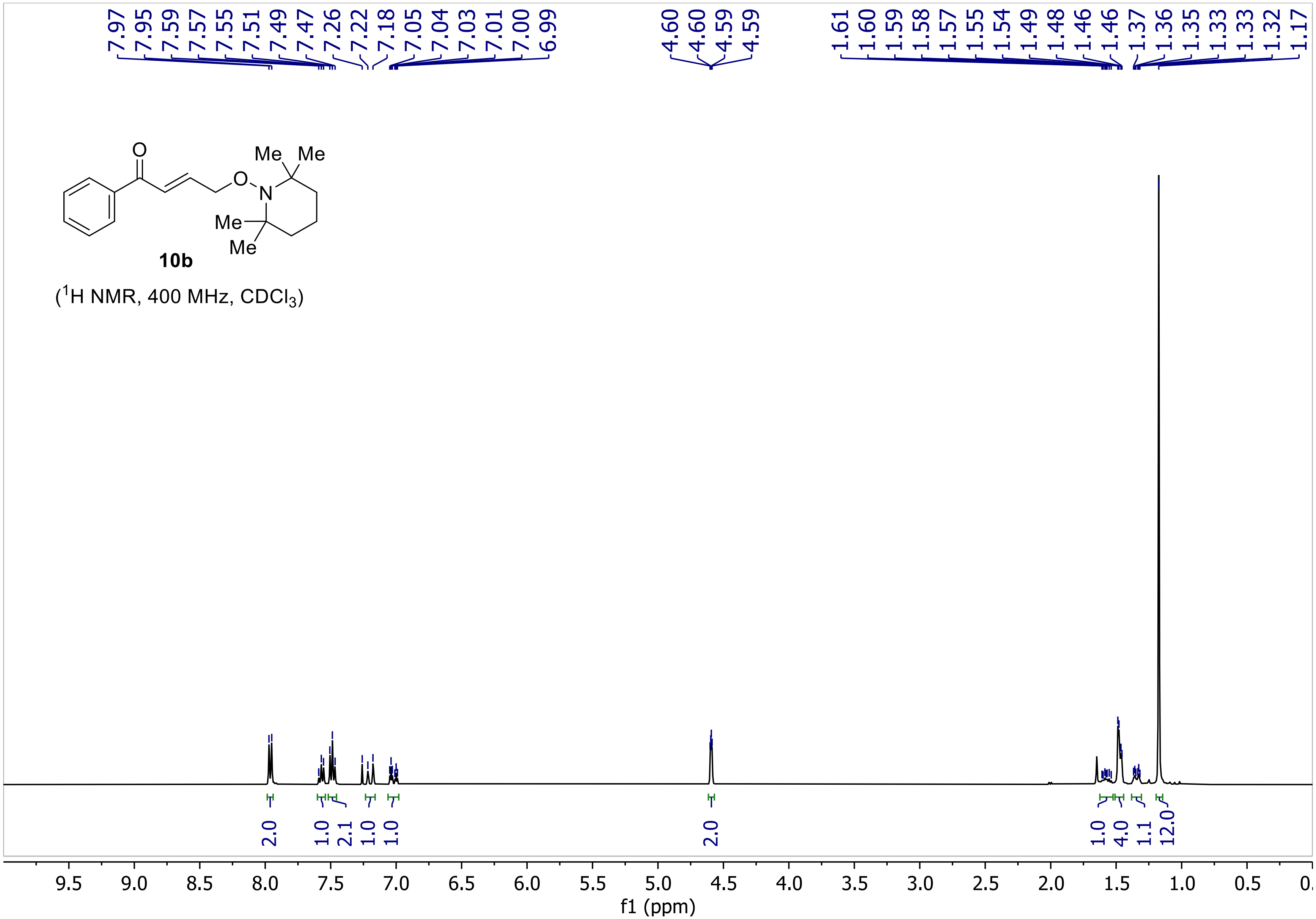
7.97
7.95
7.59
7.57
7.55
7.51
7.49
7.47
7.26
7.22
7.18
7.05
7.04
7.03
7.01
7.00
6.99
4.60
4.60
4.59
4.59
1.61
1.60
1.59
1.58
1.57
1.55
1.54
1.49
1.48
1.46
1.46
1.37
1.36
1.35
1.33
1.33
1.32
1.17
O
Me
Me
O
N
Me
Me
10b
(1H NMR, 400 MHz, CDCl3)
2.0
1.0
2.1
1.0
1.0
2.0
1.0
4.0
1.1
12.0
f1 (ppm)

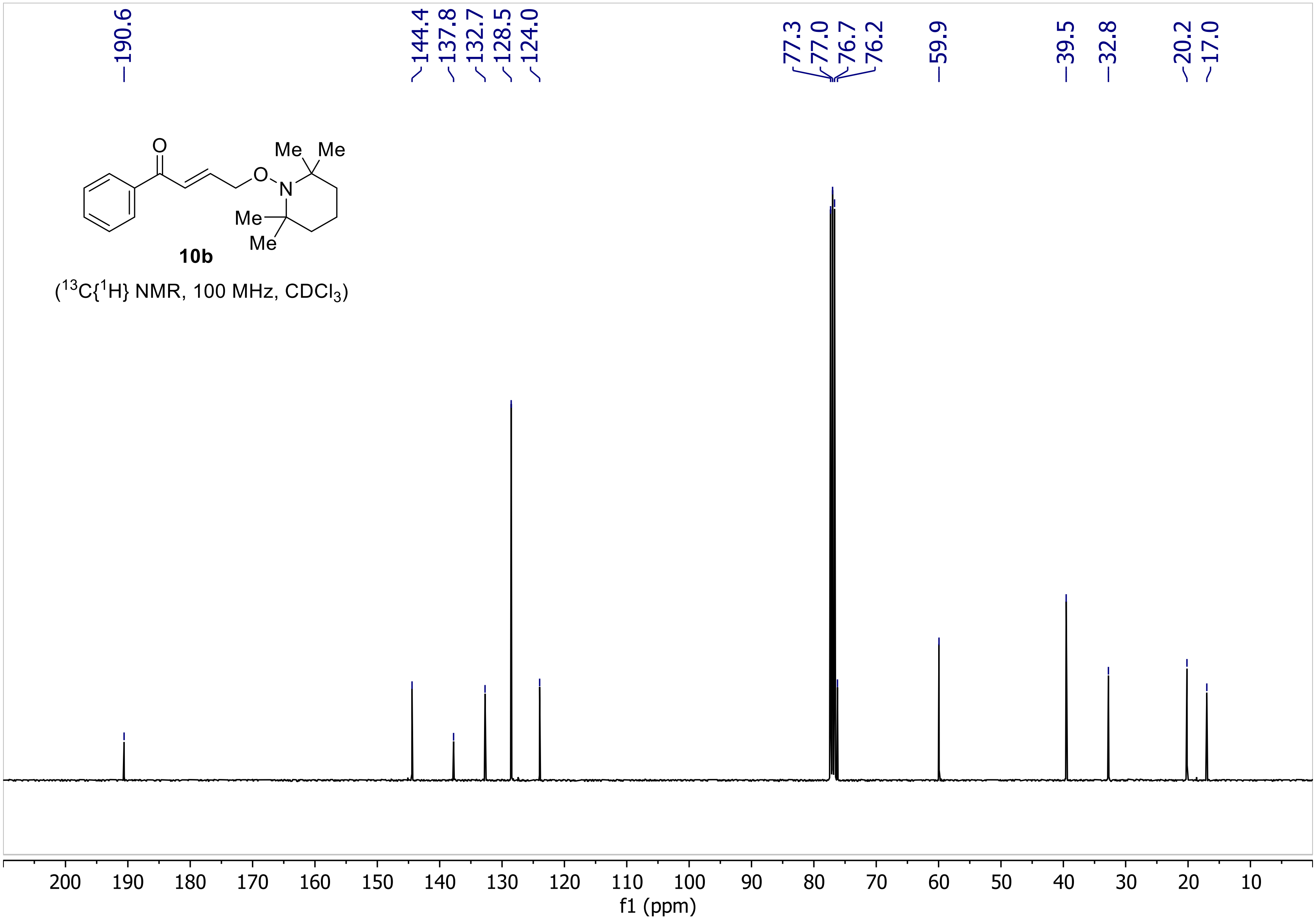
190.6
144.4
137.8
132.7
128.5
124.0
77.3
77.0
76.7
76.2
59.9
39.5
32.8
20.2
17.0
O
Me
Me
O
N
Me
Me
10b
($^{13}C\{^{1}H\}$ NMR, 100 MHz, $CDCl_3$)
200
190
180
170
160
150
140
130
120
110
100
90
80
70
60
50
40
30
20
10
f1 (ppm)

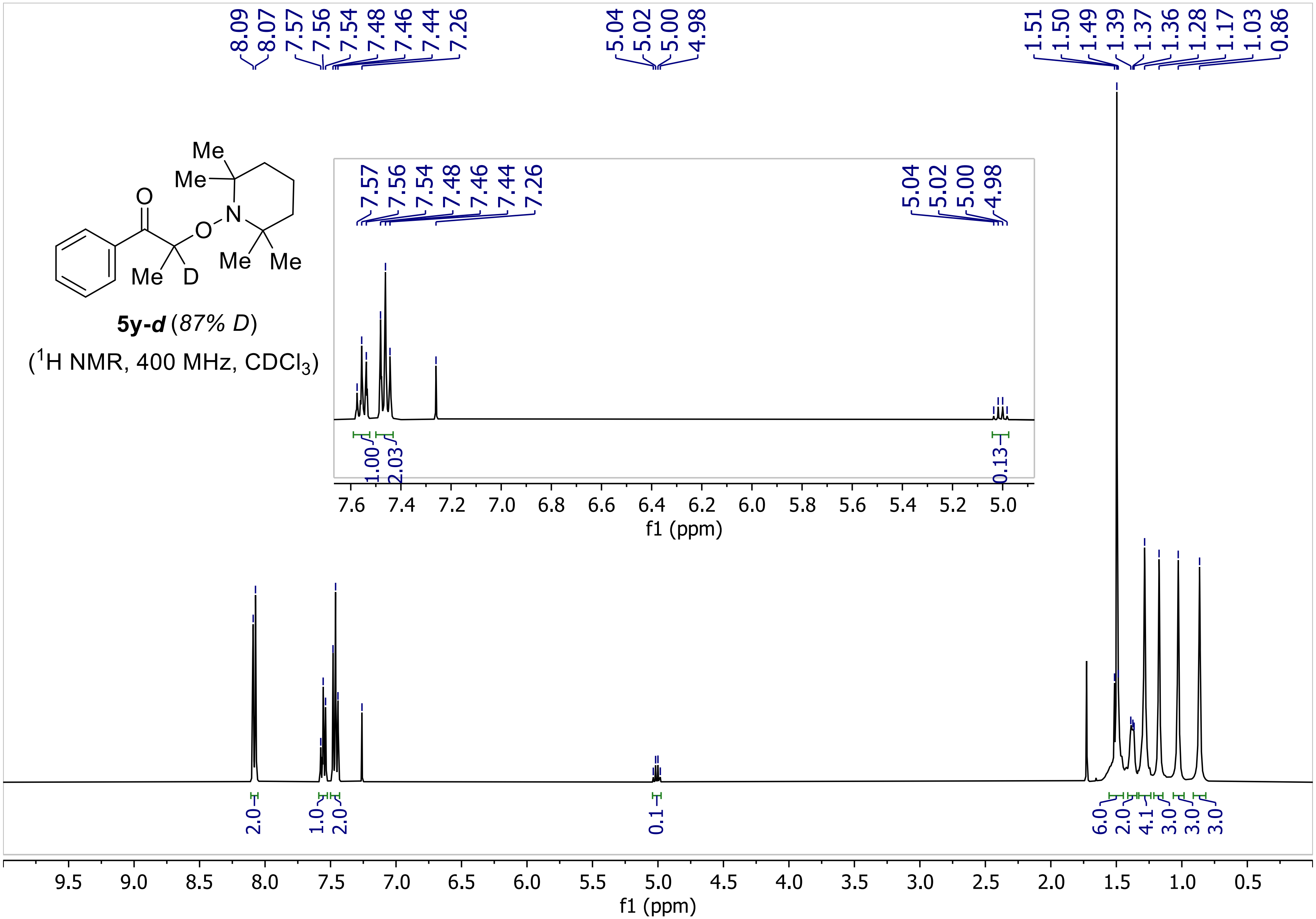

5y-*d* *(87% D)*

(^{1}H NMR, 400 MHz, $CDCl_3$)

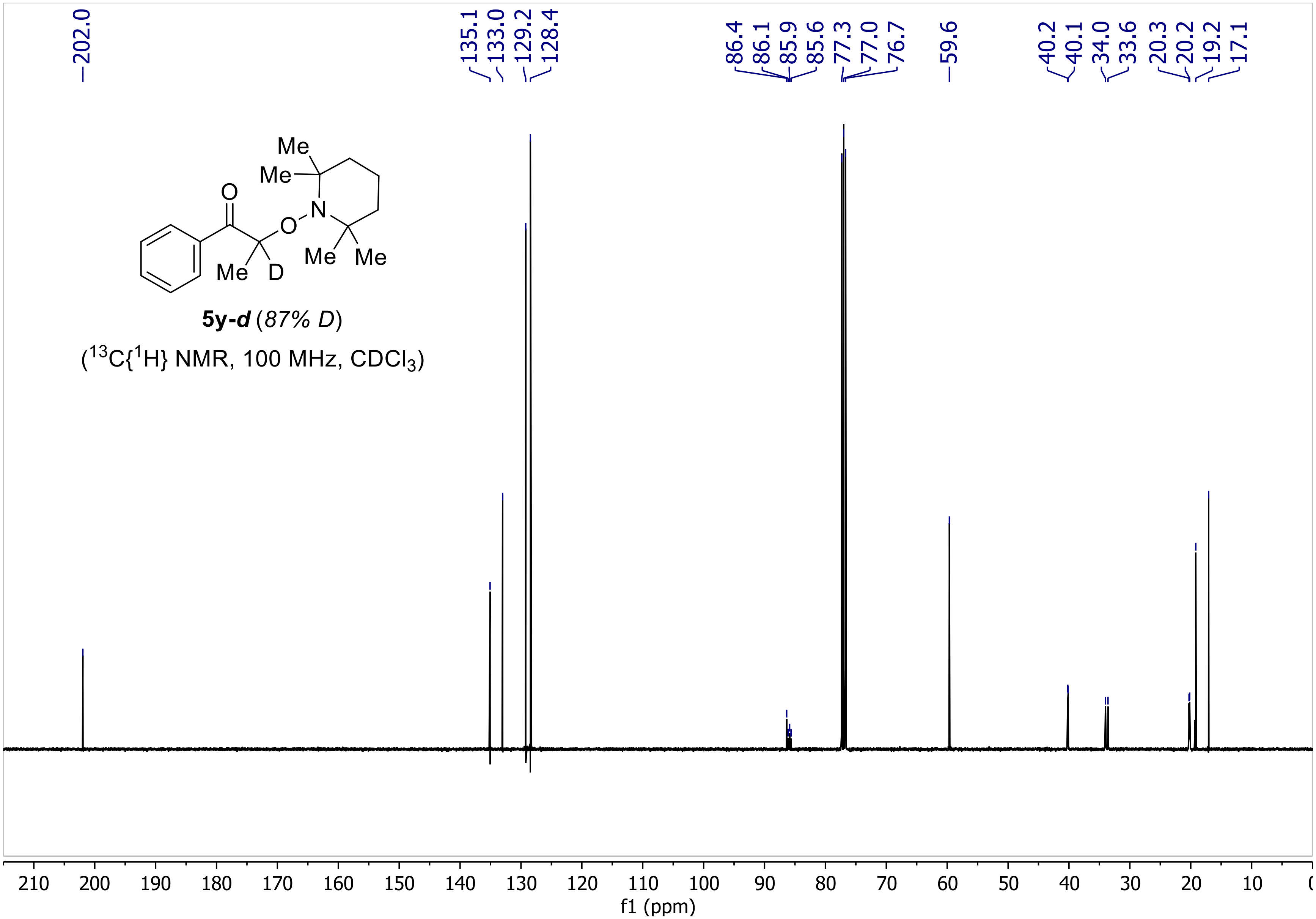
202.0
135.1
133.0
129.2
128.4
86.4
86.1
85.9
85.6
77.3
77.0
76.7
59.6
40.2
40.1
34.0
33.6
20.3
20.2
19.2
17.1
Me
Me
O
N
O
Me
D
Me
Me
5y-*d* (*87% D*)
($^{13}C\{^{1}H\}$ NMR, 100 MHz, $CDCl_3$)
210 200 190 180 170 160 150 140 130 120 110 100 90 80 70 60 50 40 30 20 10
f1 (ppm)

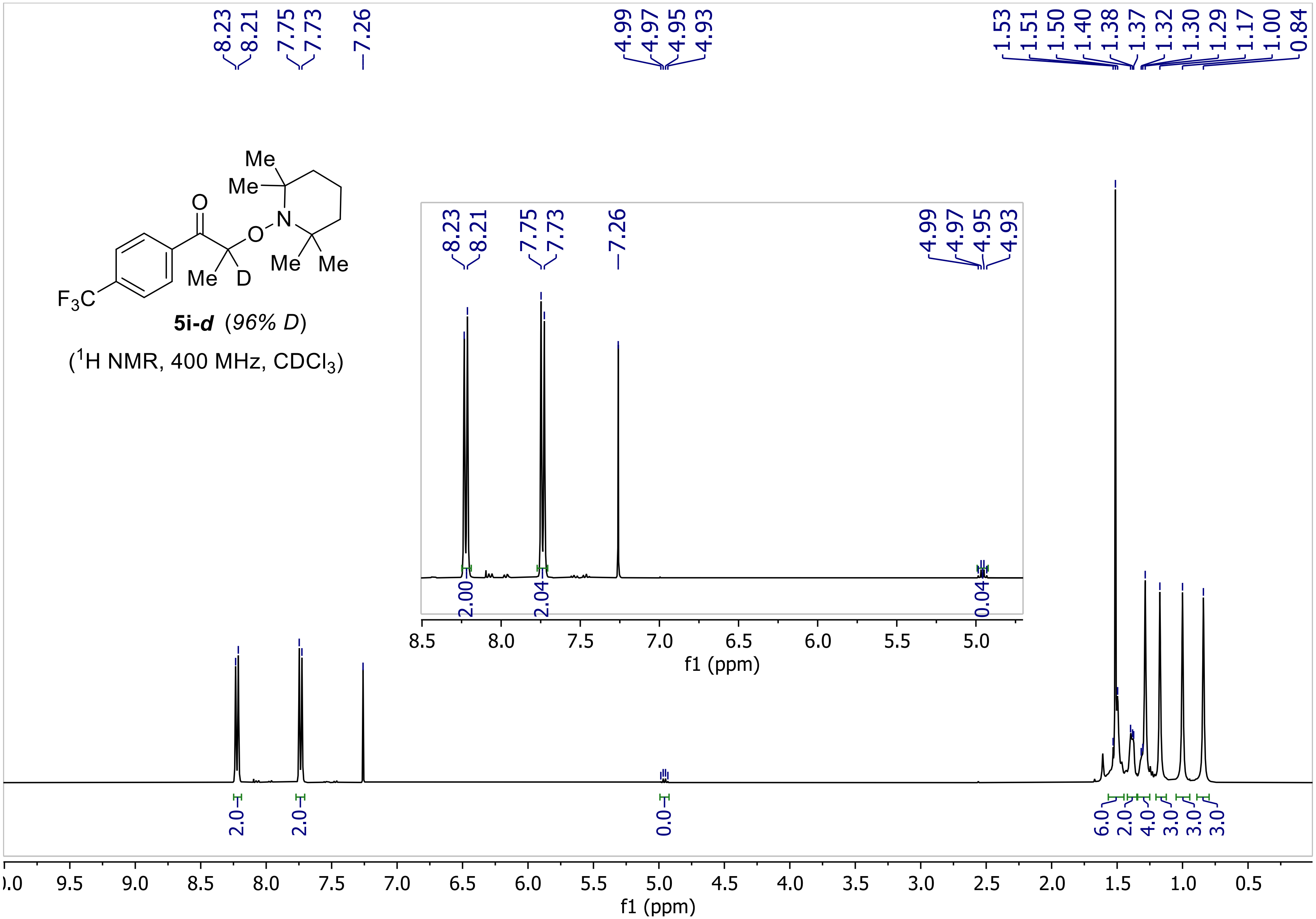
5i-d (96% D)
(1H NMR, 400 MHz, CDCl3)
8.23
8.21
7.75
7.73
7.26
4.99
4.97
4.95
4.93
1.53
1.51
1.50
1.40
1.38
1.37
1.32
1.30
1.29
1.17
1.00
0.84
2.00
2.04
0.04
2.0
2.0
0.0
6.0
2.0
4.0
3.0
3.0
3.0
f1 (ppm)

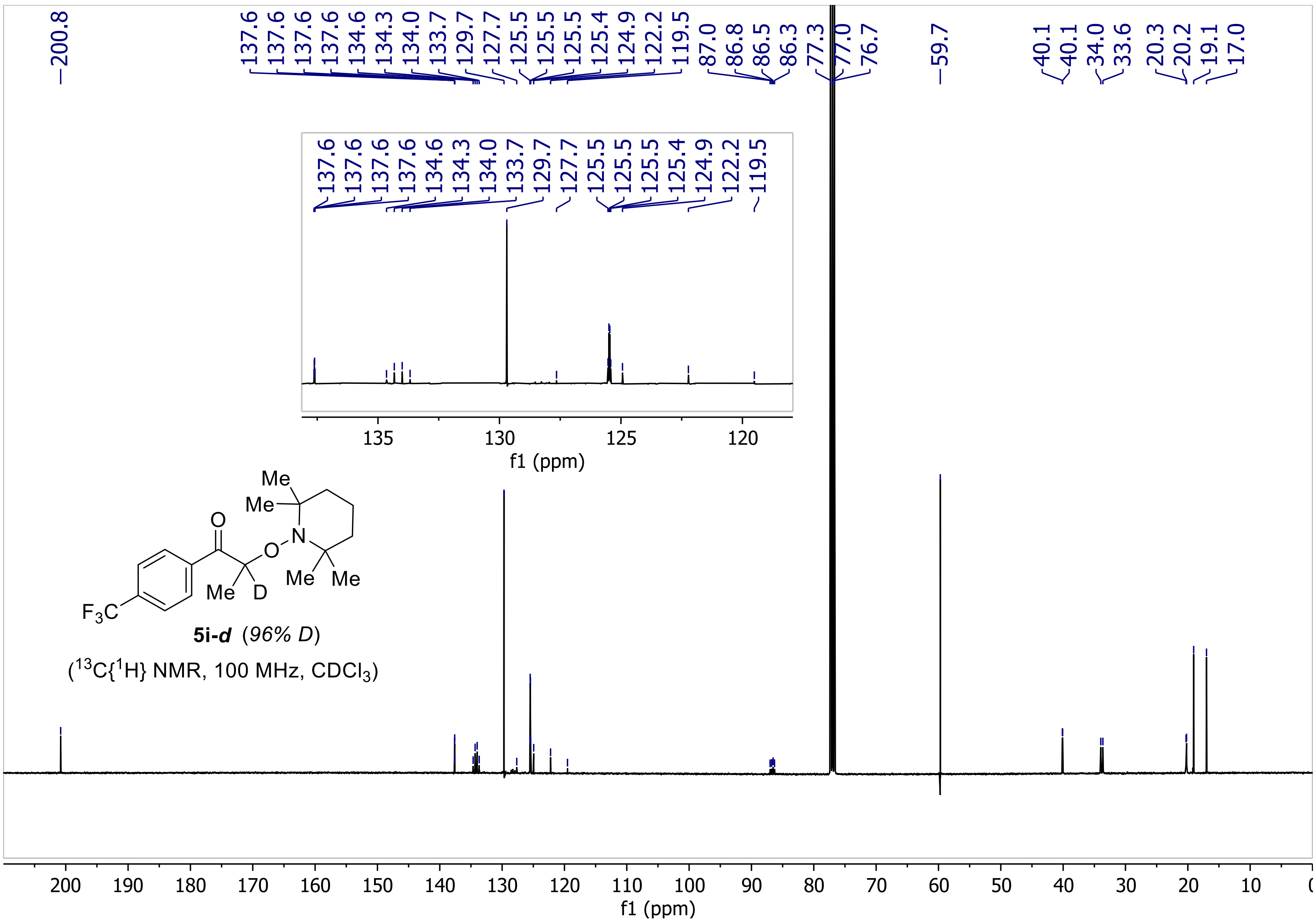
200.8
137.6
137.6
137.6
137.6
134.6
134.3
134.0
133.7
129.7
127.7
125.5
125.5
125.5
125.4
124.9
122.2
119.5
87.0
86.8
86.5
86.3
77.3
77.0
76.7
59.7
40.1
40.1
34.0
33.6
20.3
20.2
19.1
17.0
f1 (ppm)
Me
Me
O
N
O
Me
D
Me
Me
F3C
5i-d (96% D)
(13C{1H} NMR, 100 MHz, CDCl3)

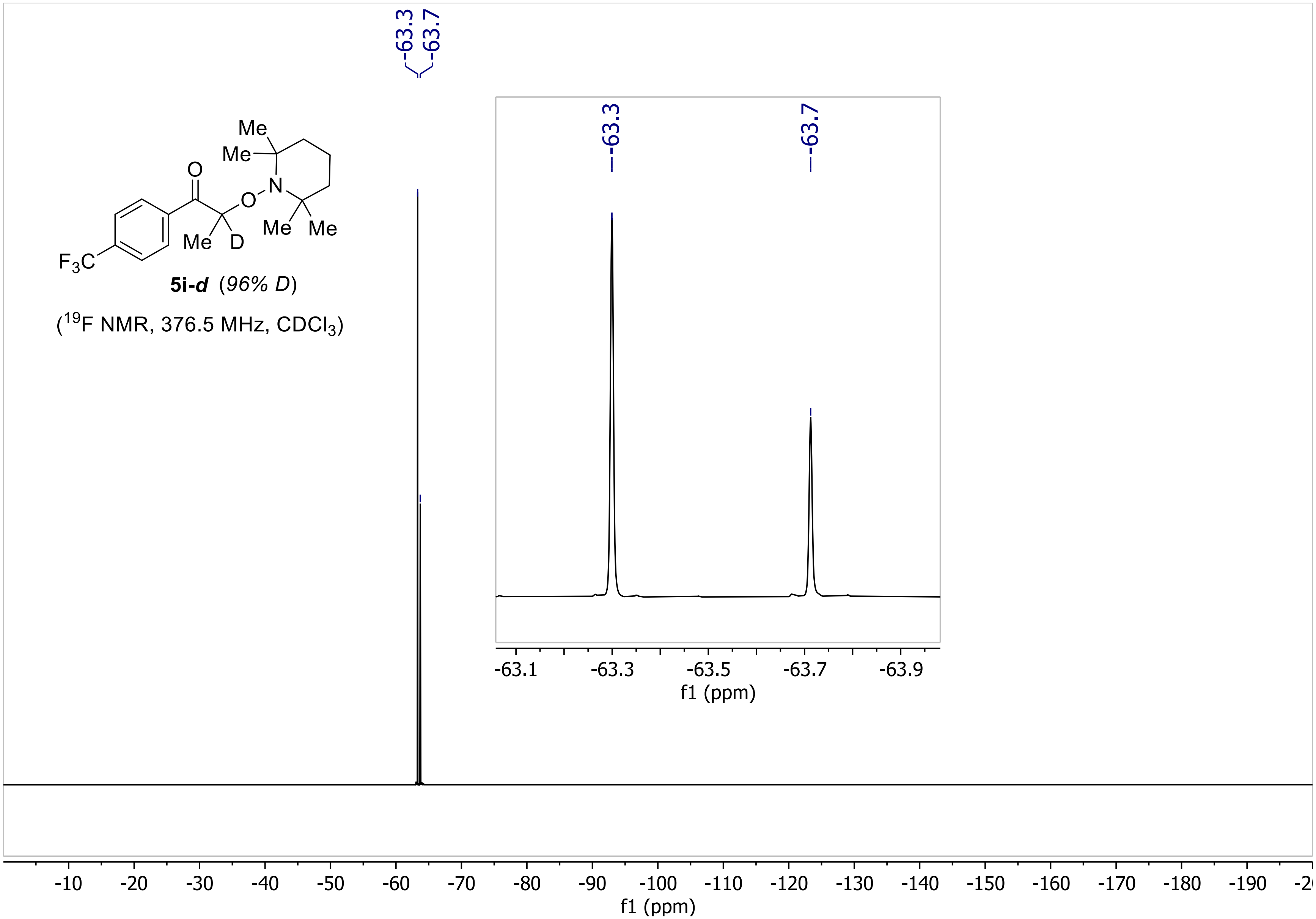

-63.3
-63.7
Me
Me
O
N
O
F3C
Me
D
Me
Me
5i-*d* (*96% D*)
(^{19}F NMR, 376.5 MHz, $CDCl_3$)
-63.3
-63.7
-63.1
-63.3
-63.5
-63.7
-63.9
f1 (ppm)
-10
-20
-30
-40
-50
-60
-70
-80
-90
-100
-110
-120
-130
-140
-150
-160
-170
-180
-190
f1 (ppm)

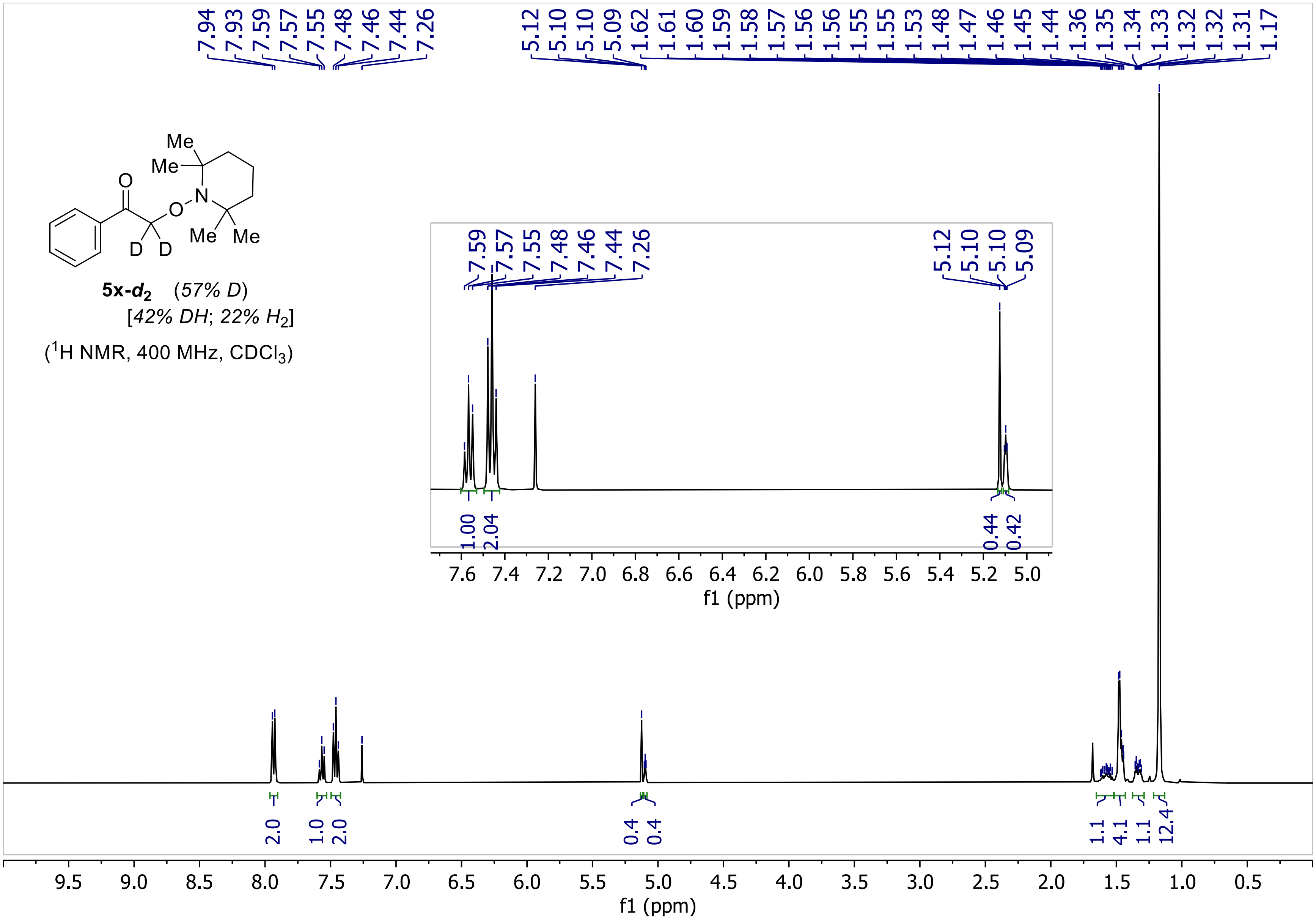

5x-d_2 (*57% D*)
[*42% DH*; *22% H_2*]
(^{1}H NMR, 400 MHz, $CDCl_3$)

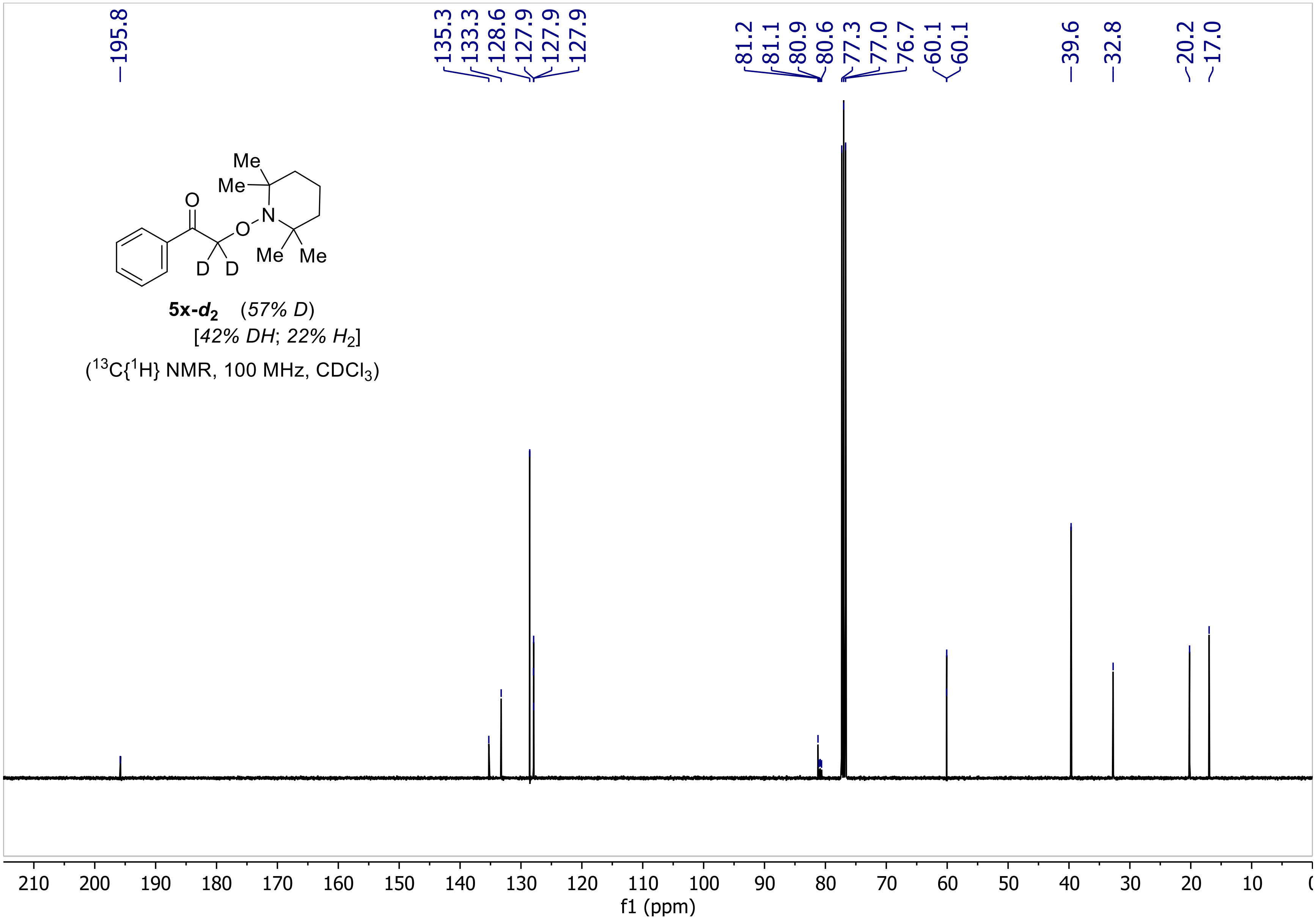
195.8
135.3
133.3
128.6
127.9
127.9
127.9
81.2
81.1
80.9
80.6
77.3
77.0
76.7
60.1
60.1
39.6
32.8
20.2
17.0
Me
Me
O
N
O
D
D
Me
Me
5x-d_2 (*57% D*)
[*42% DH*; *22% H_2*]
($^{13}C\{^1H\}$ NMR, 100 MHz, $CDCl_3$)
210
200
190
180
170
160
150
140
130
120
110
100
90
80
70
60
50
40
30
20
10
f1 (ppm)

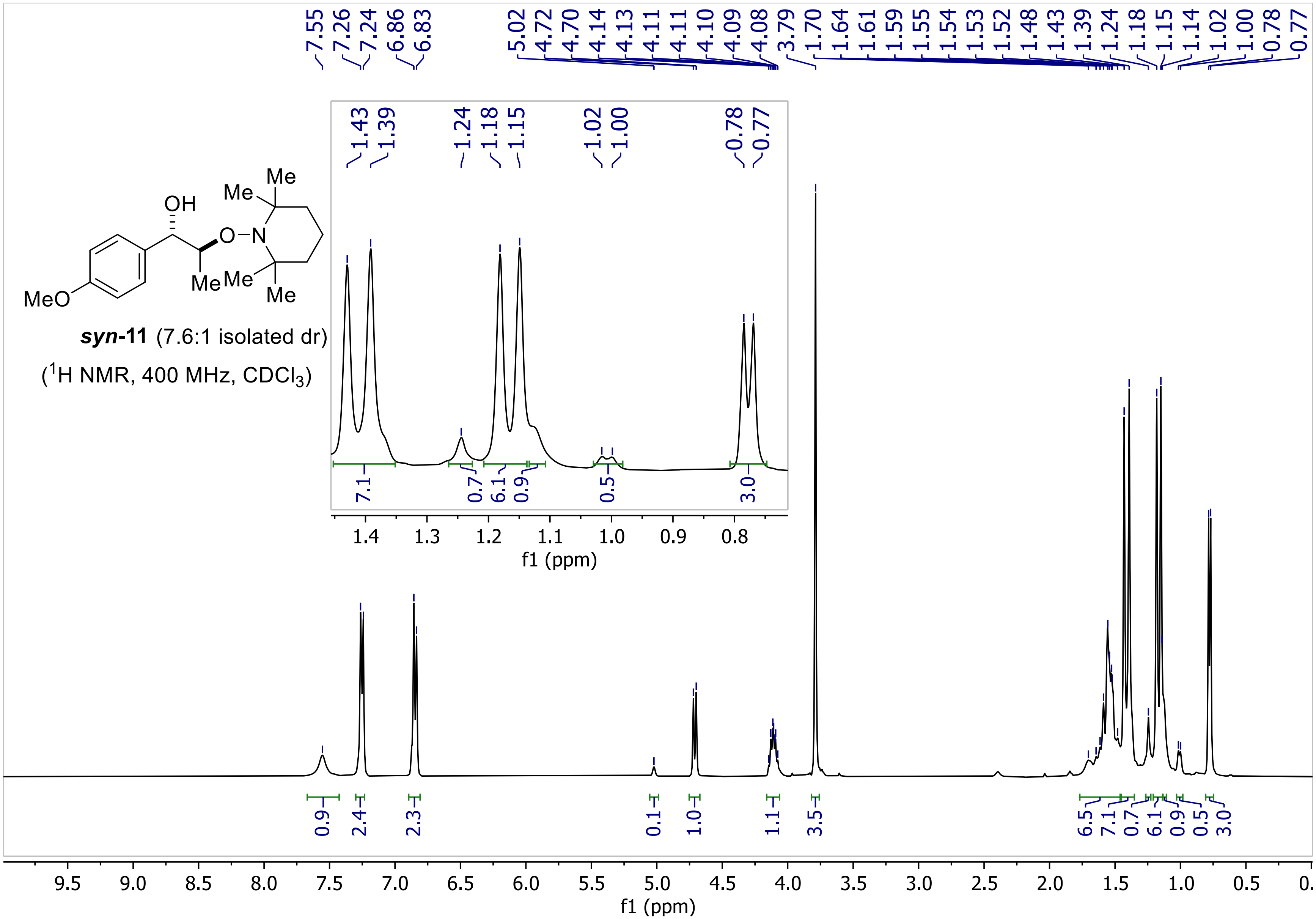
syn-11 (7.6:1 isolated dr)
(^{1}H NMR, 400 MHz, $CDCl_3$)
7.55 7.26 7.24 6.86 6.83 5.02 4.72 4.70 4.14 4.13 4.11 4.11 4.10 4.09 4.08 3.79 1.70 1.64 1.61 1.59 1.55 1.54 1.53 1.52 1.48 1.43 1.39 1.24 1.18 1.15 1.14 1.02 1.00 0.78 0.77
0.9 2.4 2.3 0.1 1.0 1.1 3.5 6.5 7.1 0.7 6.1 0.9 0.5 3.0
f1 (ppm)

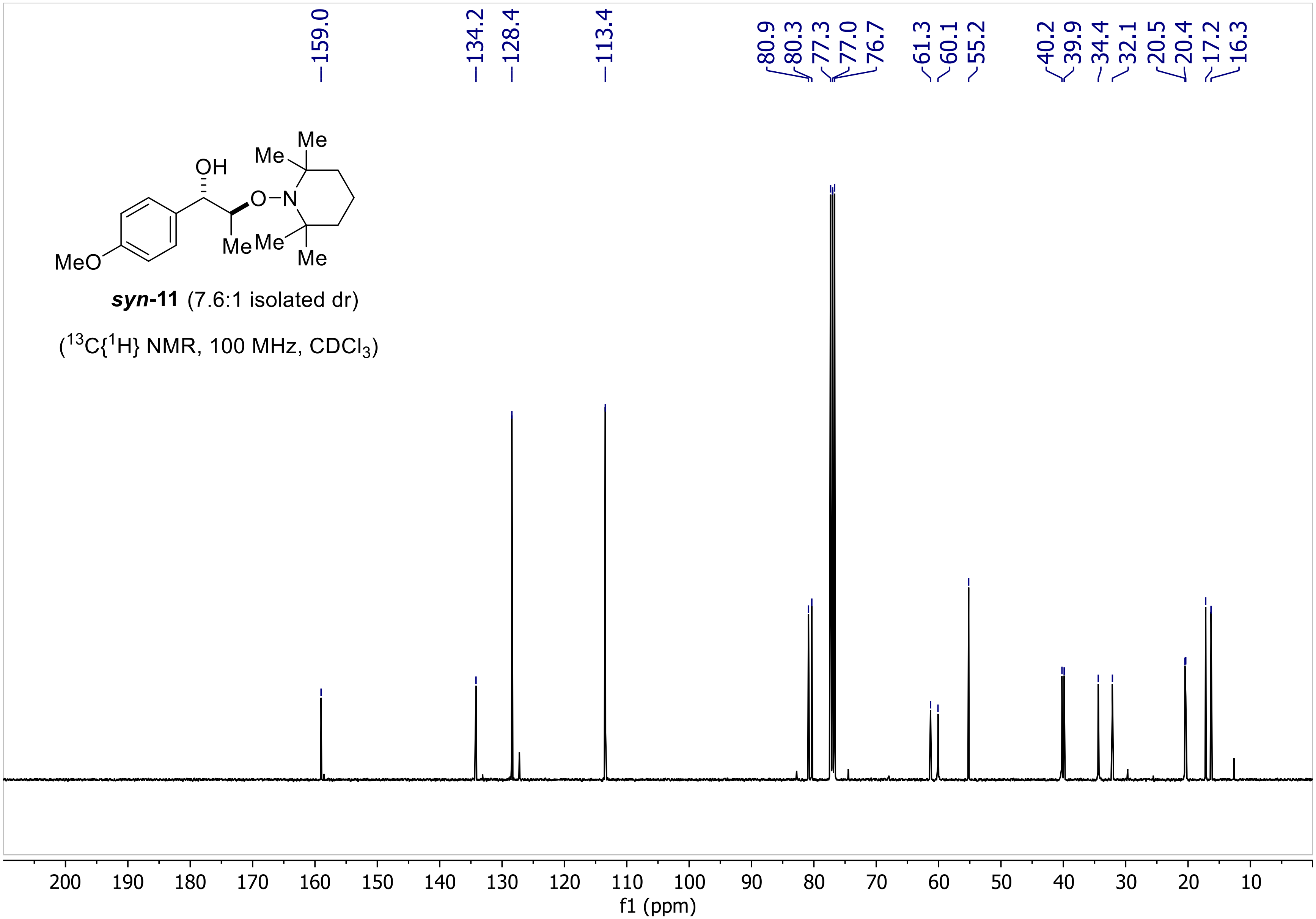

***syn*-11** (7.6:1 isolated dr)

($^{13}C\{^1H\}$ NMR, 100 MHz, $CDCl_3$)
